# Direct Regioselective *para*‐Fluorination via I(I)/I(III) Catalysis

**DOI:** 10.1002/anie.5570530

**Published:** 2026-07-09

**Authors:** Christoph Roblick, Julian D. Bendix, Timothy A. Hilton, Kathrin Bussmann, Constantin G. Daniliuc, Ryan Gilmour

**Affiliations:** ^1^ Institute for Organic Chemistry University of Münster Münster Germany

**Keywords:** catalysis, fluorination, hypervalent iodine, organocatalysis, regioselectivity

## Abstract

Fluorine scanning is an expansive strategy to refine the physicochemical profile of clinically important small molecule drugs. Of the possible regioisomers derived from mono‐substituted benzenes, the *para*‐fluorophenyl motif has emerged as a conspicuous chemotype: Lipitor is a compelling exemplar. When considered through this molecular design lens, the development of effective routes to generate this privileged module, without substrate pre‐functionalization, remains very much in focus. Herein, we report a direct, *para*‐selective fluorination that operates under the auspices of I(I)/I(III) catalysis. This enabling strategy leverages the venerable *Leonard link* inherent to phenylpropanoate and phenylpropanamides to promote a spirocyclization/*para*‐selective activation sequence. Aside from biasing regioselectivity, the C_3_‐side chain of the *Leonard link* maps onto a range of common drug scaffolds and allows the substrate scope to be extended to amino acids and dipeptides (up to 94% yield). X‐ray crystallographic analyses of selected products reveal intermolecular Dunitz–Diederich‐type interactions between the C(sp^2^)‐F bond and the π*_C═O_ orbital of proximal amides. It is envisaged that this organocatalytic platform to enable direct *para*‐fluorination will facilitate the exploration of organo‐fluorine chemical space.

## Graphical abstract

A direct, *para*‐selective fluorination by I(I)/I(III) catalysis is disclosed that does not require substrate pre‐functionalization. This strategy leverages the *Leonard link* inherent to phenylpropanoate and phenylpropanamides to promote a spirocyclization/*para*‐selective sequence. The C_3_‐side chain maps onto a range of common drug scaffolds and allows the substrate scope to be extended to amino acids and dipeptides (up to 94%).

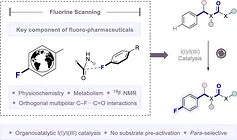

## 1 Introduction

**FIGURE 1:**
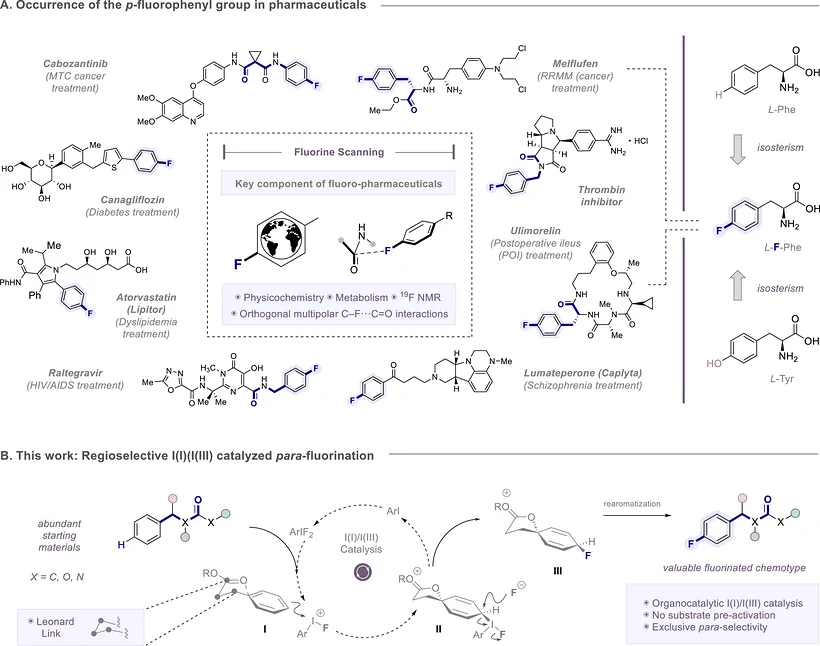
(A) The occurrence and importance of the *p*‐fluorophenyl group in leading pharmaceuticals. (B) An I(I)/I(III) catalysis‐based platform for direct, regioselective *para*‐fluorination.

Fluorine scanning has become a foundational pillar of contemporary drug discovery by enabling physicochemical refinement through the precise introduction of fluorine atoms [1, 2, 3, 4]. Often executed as part of a wider analysis of structure‐function interplay, the success of this particular approach is manifest in the ever‐expanding arsenal of blockbuster drugs that contain fluorinated signatures [5, 6, 7, 8, 9, 10, 11]. As organofluorine biochemistry is extremely limited in nature [12, 13, 14, 15], this inimitable strategy has no biological parallels and is a triumph of successfully leveraging the modest steric demand and high electronegativity of this substituent [16, 17, 18, 19, 20, 21, 22, 23, 24, 25]. Sustained innovation is, however, conditional on the development of enabling methods to expedite the synthesis of privileged chemotypes [25, 26, 27] to advance molecular design hypotheses [10, 28, 29, 30, 31]. Whilst remarkable progress has been made in the field of C(sp^3^)‐based fluorination patterns [32, 33, 34, 35, 36], most notably O´Hagan´s seminal contributions to multi‐*vicinal* fluoroalkanes [37, 38, 39, 40, 41, 42], the impact of fluorine scanning can be most keenly felt in tailoring the properties of aryl groups. The impact of C(sp^2^)‐F introduction is often multifaceted and may (i) improve metabolic stability [43, 44], (ii) facilitate structural analysis by bio‐NMR [45, 46, 47, 48, 49, 50, 51, 52], and/or (iii) enable fluorine‐specific interactions to be leveraged [53, 54]. A structural survey of fluorinated chemotypes by Shibata and co‐workers has revealed that of the possible regioisomers derived from fluorination of mono‐substituted benzenes, the *para*‐derivative is most conspicuous (Figure 1A) [55, 56]. The importance of this motif in a societal context is reflected across a very broad clinical spectrum that ranges from oncology (cabozantinib) to virology (raltegravir), psychiatry (Caplyta), obesity (Lipitor) and diabetes (canagliflozin) [1, 2, 3, 4, 5, 6, 7, 8, 9, 10, 11, 28, 29, 30, 31, 55, 56]. Moreover, the *p*‐fluorophenyl group continues to find widespread application in peptide drugs (e.g., oncology – melflufen; post‐operative ileus – ulimorelin) [57] and protein design and may be considered as an isostere of phenylalanine and tyrosine (Figure 1A, right) [46, 58, 59, 60, 61]. Finally, no discussion of this motif in drug discovery would be complete without reference to the venerable Dunitz–Diederich interaction [53, 54, 62, 63, 64, 65], in which the C_Ar_(sp^2^)‐F bond interacts with the π*_C═O_ orbital of proximal amides; this has been used to great effect in thrombin inhibitor design [66, 67]. Consequently, catalysis‐based strategies to expedite the introduction of the *para*‐C(sp^2^)‐F substituent, without the need for substrate pre‐functionalization [2, 68, 69, 70, 71, 72, 73, 74, 75, 76, 77, 78, 79, 80, 81, 82, 83, 84, 85, 86], remains a core objective in contemporary organofluorine chemistry.

**FIGURE 2:**
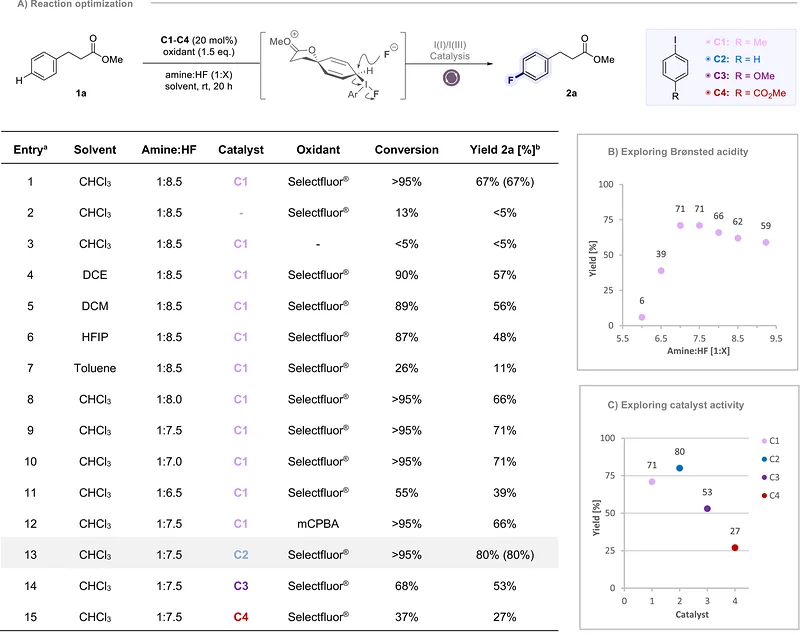
Reaction development. (A) Optimization of transformation **1a** to product **2a**. ^a^Standard reaction conditions: **1a** (0.2 mmol), catalyst (20 mol%), oxidant (1.5 eq.), amine:HF mixture (0.5 mL), solvent (0.5 mL), rt, 20h. ^b^NMR yields are determined by ^19^F NMR using 4‐fluorotoluene as the internal standard. Isolated yields are given in parentheses. (B) Influence of the amine:HF ratio on the reaction. Conditions: **1a** (0.2 mmol), *p*‐TolI (**C1**) (20 mol%), Selectfluor (1.5 eq.), amine:HF mixture (0.5 mL), CHCl_3_ (0.5 mL). (C) Catalyst screening experiments using inexpensive aryliodide catalysts **C1**–**C4** and a 1:7.5 amine:HF ratio.

To contribute to this field, it was envisaged that an organocatalytic I(I)/I(III) catalysis manifold [87, 88, 89, 90, 91, 92, 93] may provide an enabling solution (Figure 1B). The conceptual reaction design was inspired by seminal work by Miyamoto and co‐workers who established that a pre‐formed hydroxy(phenyl)‐λ^3^‐iodane•18‐crown‐6 complex could enable selective fluorination of phenyl groups in the presence of a Lewis acid activator (BF_3_•OEt_2_) [94]. This reagent was prepared from iodosylbenzene and HBF_4_•Me_2_O in the presence of equimolar 18‐crown‐6 following a procedure previously reported by the same group [95]. Moreover, Meng, Li and co‐workers have reported that stoichiometric amounts of PhI(OAc)_2_ and HF enable the fluorination of pivaloyl‐based anilides via a postulated *N*‐activation strategy [96]. To transition to a catalysis paradigm, it was reasoned that mono‐substituted aryl substrates might regioselectively engage a bulky, transient electrophile (λ^3^‐ArIF_2_ generated in situ) [97, 98, 99] to forge an intermediate cation (**I** → **II**). A key consideration was the installation of a heteroatom‐containing (C_3_)‐*Leonard link* [100, 101] in the substrate to forge a transient spirocyclic cation (**II**) and also ensure that the product would map onto a range of common drug discovery modules (highlighted in bold in Figure 1). It is pertinent to note that the trimethylene bridge ‐(CH_2_)_3_‐ is conspicuous in Kita‐type spirocyclization precursors, which provided further confidence for this approach [102, 103, 104]. Displacement at the doubly allylic C(sp^3^)‐I(III) centre was expected to be facile, with rearomatization forging the desired *p*‐fluorinated product and re‐generating the ArI(I) catalyst. A systematic investigation of oxidants, amine:HF ratios and reaction media would then converge on a general I(I)/I(III) catalysis platform to enable the title transformation. Reaction development was initiated by exploring the conversion of substrate **1a** to the *para*‐fluorinated product **2a** in the presence of simply aryl iodide catalysts (Figure 2).

## 2 Results and Discussion

To complement existing stoichiometric variants, mitigate the need for reagent preparation, and validate a general approach to *para*‐fluorination, it was envisaged that an I(I)/I(III) redox cycle [105, 106, 107] starting from inexpensive aryl iodide organocatalysts would be enabling. If successful, this strategy would expand the scope of I(I)/I(III) catalysis beyond simple π‐bonds [108, 109, 110, 111, 112, 113, 114, 115, 116, 117, 118, 119, 120, 121, 122, 123] to aromatic substrates [54]. To that end, aryl iodides **C1**‐**C4** were used in combination with Selectfluor and amine•HF complexes to explore the conversion of **1a** → **2a**. An initial hit using *p*‐iodotoluene and an amine:HF ratio of 1:8.5 in CHCl_3_ furnished the desired product in 67% yield, 95% conversion (Figure 2A, Entry 1). Control reactions in the absence of the catalyst or oxidant did not yield the desired product, thereby supporting the involvement of an I(I)/I(III) cycle (Entries 2 and 3, respectively). Altering the reaction medium did not enhance catalysis efficiency (Entries 4–7), and so attention was turned to the impact of Brønsted acidity on the outcome of the reaction [107, 124, 125]. Systematically lowering the amine:HF ratio from 1:8.0 to 1:6.5 (Entries 8–11) identified an optimal value of 1:7.5 (Entry 9, 71%; please see insert B: plot of yield as determined by ^19^F NMR spectroscopy versus Brønsted acidity). As with all reactions that employ amine•HF complexes, caution should be exercised when handling these reagents (please see the Supporting Information). Replacing Selectfluor by *m*‐CPBA [126, 127] did not enhance the yield of the reaction (Entry 12), and so minor alternations to the catalyst were considered. As summarized in Figure 2C, switching from *p*‐iodotoluene (**C1**) to iodobenzene (**C2**) enhanced the yield to 80% (Entry 13). However, the introduction of strong electron‐donating or withdrawing groups (**C3** and **C4**, respectively) proved detrimental to the outcome. For full details on reaction optimization, please see the Supporting Information.

**SCHEME 1:**
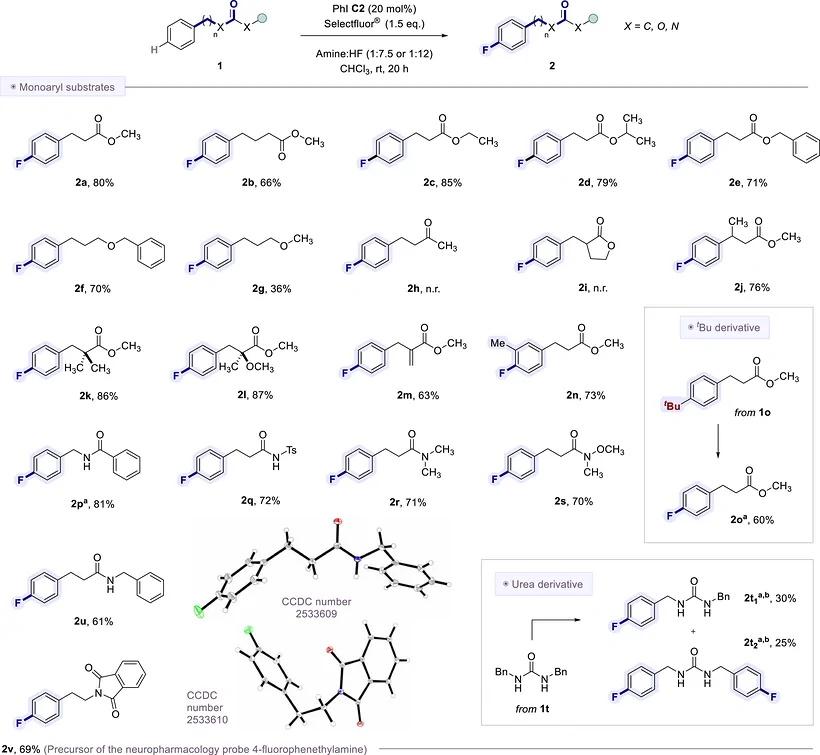
Scope of regioselective *para*‐fluorination. Standard reaction conditions: substrate **1** (0.2 mmol, 1 eq.), iodobenzene **C2** (20 mol%), Selectfluor (1.5 eq.), amine:HF (1:7.5, 0.5 mL), CHCl_3_ (0.5 mL), rt, 20h. Isolated yields are given. ^a^DMPU:HF (1:12) was used instead of an amine:HF ratio of (1:7.5). ^b^3 equivalents of Selectfluor were used instead of 1.5 eq. *nr* = no reaction. Full experimental details are provided in the Supporting Information. Bottom: X‐ray structures of **2u** [143] and **2v** [144].

With these optimized conditions in hand, attention was then turned to establishing a scope that would fully leverage the *Leonard link* as a conformational tool to pre‐organize the scaffold [128, 129, 130, 131] towards the formation of a key spirocyclic intermediate (Scheme 1). Initially, simple mono‐arylated substrates were explored with a view to establishing the impact of spacer length relative to compound **2a** (80%). Extending the spacer by one carbon atom had a marginal impact on efficiency, but the desired product could be isolated in 66% yield. Modifying the ester group was generally well tolerated (**2c, 2d** and **2e**, up to 85%), and ethers such as **2f** and **2g** were also compatible (up to 70%), indicating that carbonyl group deletion was unproblematic. An interesting limitation of the method was the ketone **2h**, which could not be detected under the standard conditions, and this was also the case for the lactone **2i**. Although speculative, this may be a consequence of competing reactivity via enolium *Umpolung* [132, 133, 134, 135]. When returning to the parent C3 scaffold, substitution at the benzylic position proved unproblematic (**2j**, 76%), and α,α‐substitution was found to be highly beneficial as illustrated by entries **2k** (86%) and **2l** (87%): this is possibly due to the Thorpe‐Ingold effect [136, 137, 138, 139] facilitating formation of the postulated spirocyclic intermediate (see Figure 1B). The α,β‐unsaturated ester proved unproblematic in **2m**, enabling regioselective *para*‐fluorination to occur chemoselectively with no 1,2‐difluorination observed [111]. Addition of a *meta*‐methyl group on the substrate did not notably suppress efficiency (**2n**, 73%), but this was not investigated further as the focus of the study was on the title chemotype. Inspired by the work of Gouverneur and colleagues on *N*‐arylsulfonamides [140], the *t*Bu substrate **1o** was explored in the hope that direct conversion to the fluoride might be observed. To our delight, the expected product **2o** could be generated in 60% yield.

Given the importance of amides in synthetic research, compounds **2p** to **2v** were prepared. Benzylic amides proved to be compatible with the general conditions, enabling compound **2p** to be generated in (81%): this is a prominent structural motif in the anti‐HIV drug Raltegravir (Figure 1A). The tosyl derivative **2q** (72%) was also formed, as was the dimethyl species **2r** (71%). To demonstrate the tolerance of Weinreb amides, compound **2s** was generated in 70% yield. Furthermore, the benzyl protected derivatives **2u** could be synthesized (61%) as well as the phthalimide protected phenethylamine **2v** (69%), a thrombin inhibitor analog [141].

**SCHEME 2:**
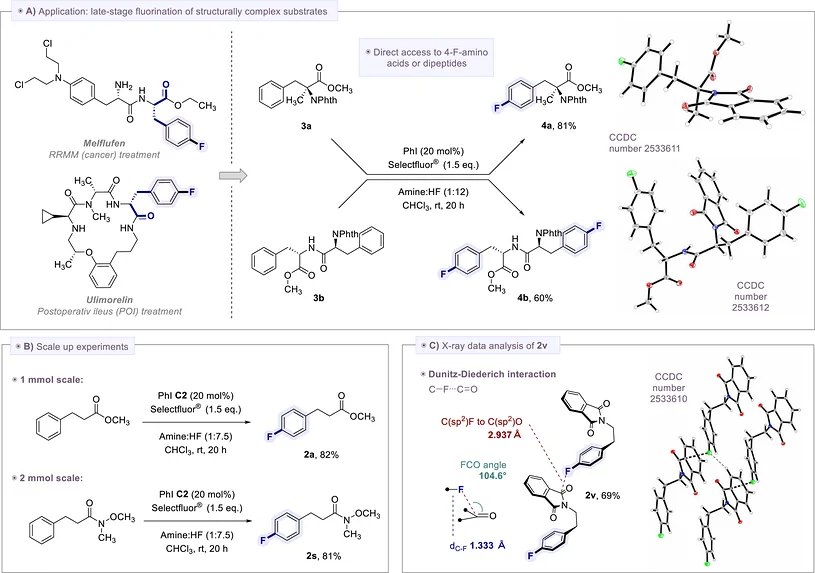
(A) Extension of applicability to late‐stage fluorination of bio‐relevant amino acids and peptides and *para*‐fluorination protocol for **3a**/**3b** to **4a**/**4b** and its x‐ray structural analysis, **4a** [147] and **4b** [148]. Conditions: substrate **3** (0.2 mmol, 1 eq.), iodobenzene **C2** (20 mol%), Selectfluor (1.5 eq.), DMPU:HF (1:12, 0.5 mL), CHCl_3_ (0.5 mL), rt, 20h. Isolated yields are given. (B) Scale‐up experiment on a 1 mmol (**2a**) and on a 2 mmol (**2s**) scale using the standard reaction conditions: substrate **1** (1 eq.), iodobenzene **C2** (20 mol%), Selectfluor (1.5 eq.), amine:HF (1:7.5), CHCl_3_, rt, 20h. Isolated yields are given. (C) X‐ray crystallographic analysis of product **2v**, observation of a C(sp^2^)‒F···C=O interaction (2.937 Å) [144].

Further structural exploration of phenethylamine scaffolds was not conducted owing to their similarity to bioactive amphetamines [142]. As indicated in the lower left section of Scheme 1, it was possible to isolate crystals of compounds **2u** [143] and **2v** [144] that were suitable for X‐ray diffraction analysis (Scheme 1, bottom). These data unequivocally establish the proof of structure. Attention was then turned to a model urea and the possibility of bidirectional modification. Substrate **1t** was subjected to the general catalysis conditions, enabling the mono‐ (**2t_1_**, 30%) and difluorinated (**2t_2_**, 25%) products to be generated (these compounds could be separated by column chromatography). This is an encouraging finding given the challenge that Lewis‐basic groups often present, acting as competing ligands, in I(I)/I(III) catalysis [117]. This provided a foundation to explore the suitability of the approach in modifying amino acid derivatives that are common building blocks in peptide‐based pharmaceuticals such as Ulimorelin or Melflufen (Scheme 2A) [57, 145, 146]. Initially, (*S*)‐α‐methylphenylalanine derivative **3a** was submitted to the catalysis conditions. The inclusion of the methyl group was deemed prudent to ensure that no potential complications arose from enolium *Umpolung* activation pathways [54, 132, 133, 134, 135]. To our delight, this enabled the desired *para*‐fluorinated product **4a** to be forged in 81% yield. Moreover, it was possible to gain additional structural support by X‐ray analysis [147]. To explore the compatibility of the method in bidirectional peptide modification and explore the effect of removing the α‐Me group, the diphenylalanine derivative **3b** was investigated.

*Para*‐selective di‐fluorination proved facile, enabling the desired product **4b** to be isolated in 60% yield. Once again, X‐ray crystallographic analysis established the molecular connectivity of the product [148].

To demonstrate the scalability of the method, products **2a** and **2s** were generated on a 1 mmol and 2 mmol scale without a loss in catalysis efficiency (Scheme 2B). Indeed, in the case of the Weinreb amide **2s**, a 10% increase in yield was noted. To return to the venerable Dunitz‐Diederich‐type interactions of *p*‐fluorophenyl groups with amides [53, 62, 63, 64, 65], a detailed inspection of the extended crystal of structure **2v** was conducted (Scheme 2C) [144]. Immediately obvious was the F‐C‐O angle in which the C(sp^2^)‐F substituent is oriented towards a neighbouring carbonyl group (104.6°). Importantly, this contact was less than the sum of the van der Waals radii [*d*(F···C═O) = 2.94 Å]. It is important to highlight that the fluorine atom is in close proximity to the neighbouring C(sp^2^) of the fused π‐system (2.826 Å), suggesting that both interactions contribute to packing.

**SCHEME 3:**
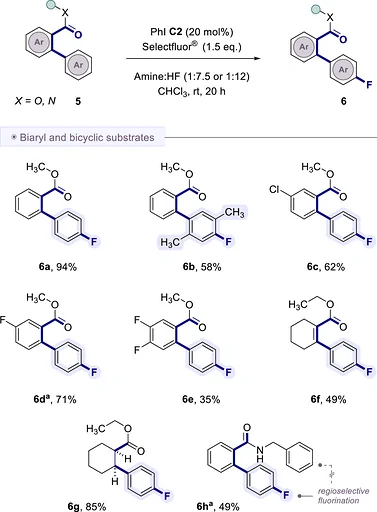
Scope of regioselective *para*‐fluorination. Standard reaction conditions: substrate **5** (0.2 mmol, 1 eq.), iodobenzene **C2** (20 mol%), Selectfluor (1.5 eq.), amine:HF (1:7.5, 0.5 mL), CHCl_3_ (0.5 mL), rt, 20h. ^a^DMPU:HF (1:12) was used instead of an amine:HF ratio of (1:7.5). Isolated yields are given. Full experimental details are provided in the supporting information.

To further extend this approach to direct *para*‐fluorination, integrating the C3‐link within a biaryl framework was considered (Scheme 3). This approach would further support the directing influence of the carbonyl group and offer a convenient strategy to enable selective mono‐fluorination. Gratifyingly, compound **6a** was generated in the highest yield of the study (94%). The introduction of 2,5‐dimethyl groups had an impact on efficiency, but the product **6b** could be isolated in 58%. The introduction of halogen atoms on the benzoic acid component also proved unproblematic (Entries **6c‐e**, up to 71%). In the case of the cyclic styrenyl system, fluorination proceeded smoothly (**6f**, 49%), and the yield could be markedly enhanced by elimination of the alkene (**6g**, 85%). As a final control, the amide **6h** was prepared: this reaction proceeded smoothly in 49% with regioselectivity for the expected aryl ring observed.

## 3 Conclusion

Motivated by the prominence of the *para*‐fluorophenyl chemotype in leading pharmaceuticals, a main group I(I)/I(III) catalysis‐based method has been developed that expedites the direct, regioselective fluorination of abundant precursors. This organocatalytic approach mitigates the need for substrate pre‐functionalization, enabling a formal C(sp^2^)‐H → C(sp^2^)‐F process. Integrating the phenylpropane‐oate/‐amide motif allows structural pre‐organization via the venerable *Leonard link* to favour the formation of a transient spirocyclic intermediate: this reinforces *para*‐activation upon engagement with in situ generated PhIF_2_. Importantly, this C_3_‐unit maps on to a range of common drugs, and the tolerance of Lewis basic groups provides an additional degree of generality to the substrate scope.

Proof of concept in urea and amino acid modification complements the mono‐arylated scope, and application in the regioselective modification of biaryl substrates highlights the synthetic utility of the strategy and the value of the *Leonard link* in ensuring directionality. It is envisaged that this operationally simple method will find application in fluorine scanning as part of wider drug discovery campaigns.

## Author Contributions

**Christoph Roblick**: conceptualization, investigation, methodology, validation, writing – review and editing, formal analysis, writing – original draft. **Julian D. Bendix**: investigation, methodology, formal analysis. **Timothy A. Hilton**: investigation, methodology, formal analysis. **Kathrin Bussmann**: investigation, methodology, formal analysis. **Constantin G. Daniliuc**: investigation, formal analysis, methodology, writing – review and editing. **Ryan Gilmour**: conceptualization, funding acquisition, writing – review and editing, writing – original draft, formal analysis, supervision, resources, project administration.

## Conflicts of Interest

The authors declare no conflicts of interest.

## Supporting information




**Supporting File 1**: The Supporting Information is available free of charge on the Wiley VCH GmbH Publications website: Experimental protocols, nuclear magnetic resonance spectra and x‐ray crystallographic data. Additional references are cited within the Supporting Information [149, 150, 151, 152, 153, 154, 155, 156, 157, 158, 159, 160, 161, 162, 163, 164, 165, 166, 167, 168, 169, 170, 171, 172, 173, 174, 175, 176, 177, 178, 179, 180, 181, 182, 183, 184, 185, 186, 187, 188, 189, 190, 191, 192, 193, 194, 195, 196, 197, 198, 199, 200, 201, 202, 203, 204, 205, 206, 207, 208, 209].

**Supporting File 2**: anie73475‐sup‐0002.cif

## Data Availability

The data that support the findings of this study are available in the Supporting Information of this article. Deposition numbers 2533609 (for **2u**), 2533610 (for **2v**), 2533611 (for **4a**), and 2533612 (for **4b**) contain the supplementary crystallographic data for this paper. These data are provided free of charge by the joint Cambridge Crystallo‐graphic Data Centre and Fachinformationszentrum Karlsruhe Access Structures service.
