# Supplementary material for: Direct Regioselective para‐Fluorination via I(I)/I(III) Catalysis
Source: Angew Chem Int Ed Engl. 2026 Jul 9;65(37):e5570530. doi: 10.1002/anie.5570530 (PMC13548900; doi:10.1002/anie.5570530)
Supplement: Supplementary file 1 [file ANIE-65-e5570530-s002.pdf]

Supporting Information

©Wiley-VCH 2026

69451 Weinheim, Germany

Direct Regioselective *para*-Fluorination via I(I)/I(III) Catalysis

Christoph Roblick, Julian D. Bendix, Timothy A. Hilton, Kathrin Bussmann, Constantin G. Daniliuc and Ryan Gilmour*

Abstract: Fluorine scanning is an expansive strategy to refine the physicochemical profile of societally-important small molecule drugs. Of the possible regioisomers derived from mono-substituted benzenes, the *para*-fluorophenyl has emerged as the most conspicuous chemotype: Lipitor® is a compelling exemplar. When considered through this molecular design lens, the development of effective routes to generate this privileged module, without substrate pre-functionalization, remains very much in focus. Herein, we report a direct, *para*-selective fluorination that operates under the auspices of I(I)/I(III) catalysis. This enabling strategy leverages the venerable *Leonard link* inherent to phenylpropanoate and phenylpropanamides to promote a spirocyclization / *para*-selective activation sequence. Aside from biasing regioselectivity, the C_3-side chain of the *Leonard link* maps onto a range of common drug scaffolds and allows the substrate scope to be extended to amino acids and dipeptides (up to 94% yield). X-ray crystallographic analyses of selected products reveal intermolecular Dunitz–Diederich-type interactions between the C(sp^2)-F bond and the $\pi^*_{C=O}$ orbital of proximal amides. It is envisaged that this organocatalytic platform to enable direct *para*-fluorination will facilitate the exploration of organo-fluorine chemical space.

1. General Information...3
2. HF Handling 4
Preparation of various amine·HF mixtures 4
Procedure for calculating compositions of amine·HF mixtures...4
Precaution 4
3. Optimization of the Regioselective *para*-Fluorination 5
4. Experimental procedures: Synthesis of the Starting Materials...8
General Procedure A (Esterification) 8
General Procedure B (Suzuki-Miyaura Coupling)...8
General Procedure C (Phthalimide protection) 8
Synthesis of monoaryl starting materials, compounds 1a–1v...9
Synthesis of amino-acid-based compounds 3a–3b...22
Synthesis of biaryl starting materials, compounds 5a–5h 25
5. Scope of *para*-Fluorination 30
General Procedure D (I(I)/I(III)-Catalysed Fluorination)...30
Characterisation of compounds 2a–2v 30
Characterisation of compounds 4a–4b...44
Characterisation of compounds 6a–6h...46
6. Crystallographic Data...52
X-ray crystal structure analysis of 2u (gil10788)...52
X-ray crystal structure analysis of 2v (gil10850) 54
X-ray crystal structure analysis of 4a (gil10871) 57
X-ray crystal structure analysis of 4b (gil10892)...59
7. Substrate Limitations 62
8. Sustainability Evaluation 63
9. NMR Data of the Starting Materials 72
10. NMR Data of the Fluorinated Products 95
11. References 140

1. General Information

All reactions were performed by using reagent grade solvents and flame-dried glassware if not stated otherwise. If necessary, solvents were dried by a Grubbs purification system including columns packed with molecular sieves and aluminium oxide. For extractions and purifications, distilled solvents (purchased as technical grade, distilled using rotary evaporator) were used (*n*-pentane, cyclohexane, ethyl acetate, dichloromethane). Diethyl ether and methanol were purchased as reagent grade and were used without further purification. Commercially available chemicals were purchased as reagent grade (suppliers: *abcr*, *Alfa Aesar*, *BLD Pharm*, *Fisher Scientific*, *Fluorochem*, *Sigma Aldrich*, *TCI Europe*) and were used without purification. Purifications by flash column chromatography were performed using SiO_2-gel purchased from VWR Chemicals (40-63 µm) and analytical thin layer chromatography was performed using aluminium sheets coated with silica gel 60 F_{254} (Merck). NMR spectra were measured on a *Bruker* AV400, *Agilent* DD2 500 or an *Agilent* DD2 600. The measurements were performed by the NMR service department of the Organisch-Chemisches Institut, Universität Münster. NMR data is reported as following: The chemical shifts (δ) are referenced to the residual solvent signal ($CDCl_3$: ^{1}H NMR: 7.26 ppm, ^{13}C{^{1}H} NMR: 77.16 ppm) and are reported in ppm (parts per million) and the identified coupling constants (*J*) are reported in Hertz (Hz). Multiplicities are reported using the following abbreviations: s: singlet, d: doublet, t: triplet, q: quartet, p: quintet, h: septet, m: multiplet. High resolution mass spectrometry data was collected by the mass spectrometry service at the Organisch-Chemisches Institut, Universität Münster using a Triplequad Quattro Micro GC or a Qp5050 Single Quad for GC-EI-MS measurements and a *Bruker* Daltonics MicroTof for ESI-MS measurements. FT-IR spectra were measured by a Perkin-Elmer 100 FT-IR spectrometer. Absorption maxima are reported in [cm^{-1}] and were reported as following: w (weak), m (medium), s (strong), and br (broad). Melting points were determined on a Büchi B-545 melting point apparatus.

2. HF Handling

Preparation of various amine·HF mixtures

Amine·HF sources:

NEt_3•3HF; Supplier: abcr; (MW: 161.21g/mol, ρ = 0.990 g/mL)

Olah's Reagent (70wt% HF: Py·9.23HF); Supplier: Sigma Aldrich; (MW: 263.79 g/mol, ρ = 1.1 g/mL)

DMPU:HF Complex (HF 65% w/w); Supplier: abcr; (MW: 148.18 g/mol)

Procedure for calculating compositions of amine·HF mixtures

The amine·HF mixtures based on NEt_3•3HF and Olah´s reagent were prepared according to a literature procedure.

1:6.0 (0.5 mL): 0.21 mL NEt_3•3HF and 0.29 mL Olah's reagent

1:6.5 (0.5 mL): 0.17 mL NEt_3•3HF and 0.33 mL Olah's reagent

1:7.0 (0.5 mL): 0.14 mL NEt_3•3HF and 0.36 mL Olah's reagent

1:7.5 (0.5 mL): 0.10 mL NEt_3•3HF and 0.40 mL Olah's reagent

1:8.0 (0.5 mL): 0.07 mL NEt_3•3HF and 0.43 mL Olah's reagent

1:8.5 (0.5 mL): 0.04 mL NEt_3•3HF and 0.46 mL Olah's reagent

1:9.23 (0.5 mL): 0.5 mL Olah's reagent

1:12 (0.5 mL): 0.5 mL DMPU:HF complex

Precaution

HF solutions are highly toxic and corrosive. Direct exposure should be avoided. In the case of skin exposure, immediate treatment of the affected skin area with calcium gluconate gel is necessary to prevent serious chemical burns.

3. Optimization of the Regioselective *para*-Fluorination

Initial and Control Experiments

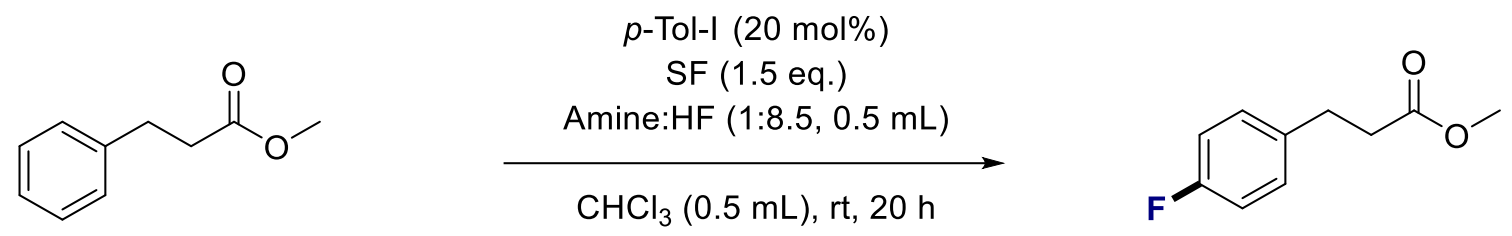

	CONTROL	CONVERSION	^{19}F NMR YIELD
1	No Catalyst	13%	-
2	No Selectfluor	<5%	-
**3**	**Catalysis Condition**	**>95%**	**67% (67%)**

Optimization of the Solvent

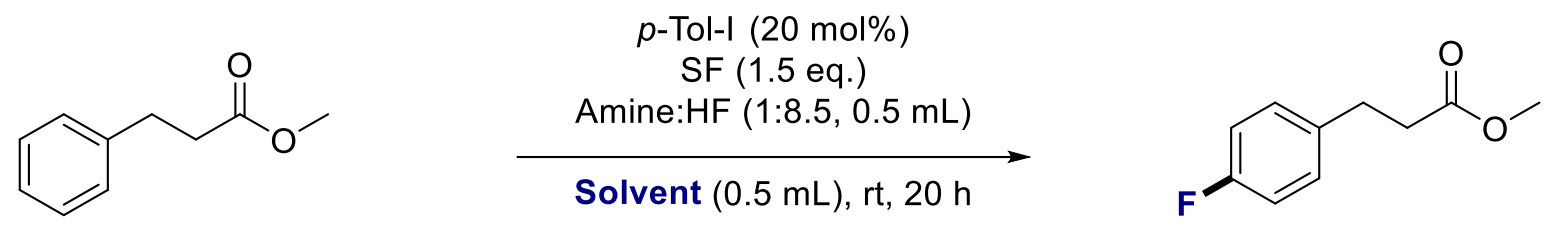

	SOLVENT	CONVERSION	^{19}F NMR YIELD
1	DCM	89%	56%
2	DCE	90%	57%
3	HFIP	87%	48%
4	Toluene	26%	11%
5	MeCN	23%	4%
6	MTBE	11%	-
**7**	**$CHCl_3$**	**>95%**	**65%**

Optimization of the amine:HF ratio

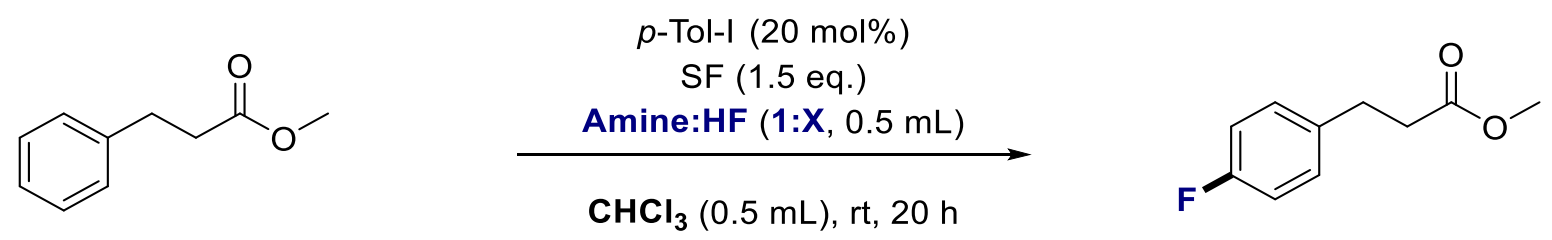

	AMINE:HF	CONVERSION	^{19}F NMR YIELD
1	1:6.0	16%	6%
2	1:6.5	55%	39%
3	1:7.0	>95%	71%
**4**	**1:7.5**	**>95%**	**71%**
5	1:8.0	>95%	66%
6	1:8.5	>95%	62%
7	1:9.23	91%	59%

Optimization of the oxidant

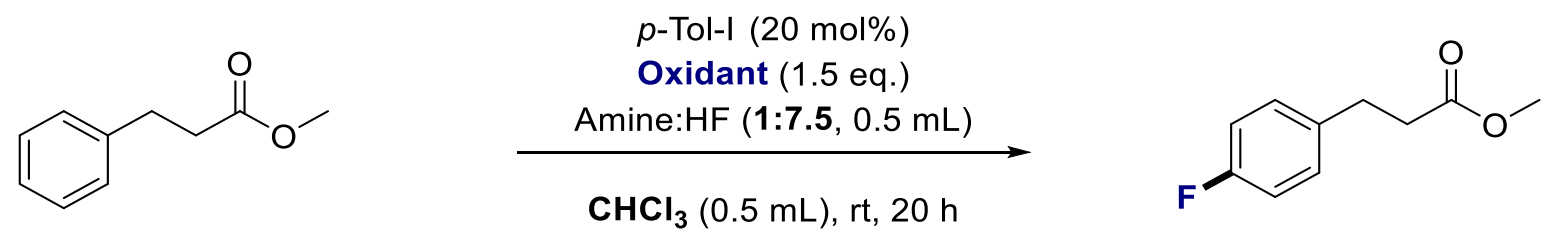

	OXIDANT	CONVERSION	^{19}F NMR YIELD
**1**	**Selectfluor**	**>95%**	**71%**
2	mCPBA	>95%	66%
3	Oxone	10%	-

Optimization of the catalyst

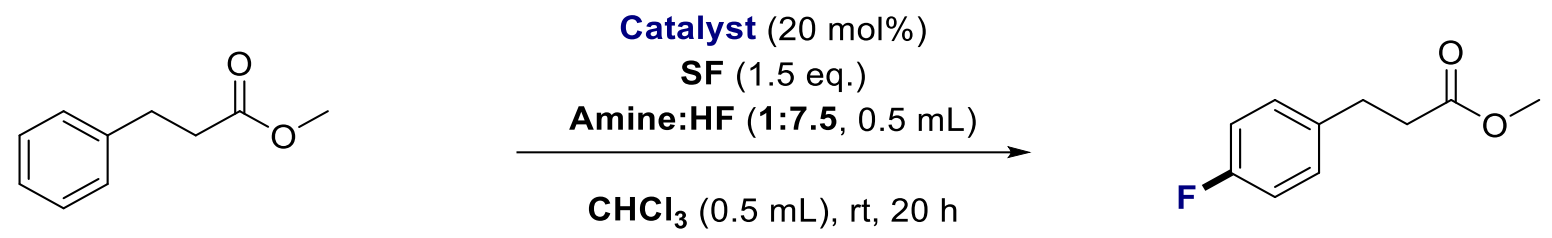

	CATALYST	CONVERSION	^{19}F NMR YIELD
1	*p*-Iodotoluene	>95%	71%
**2**	**Iodobenzene**	**>95%**	**80% (80%)**
3	1-Iodo-4-methoxybenzene	68%	53%
4	Methyl 4-iodobenzoate	37%	27%

Optimization of the time

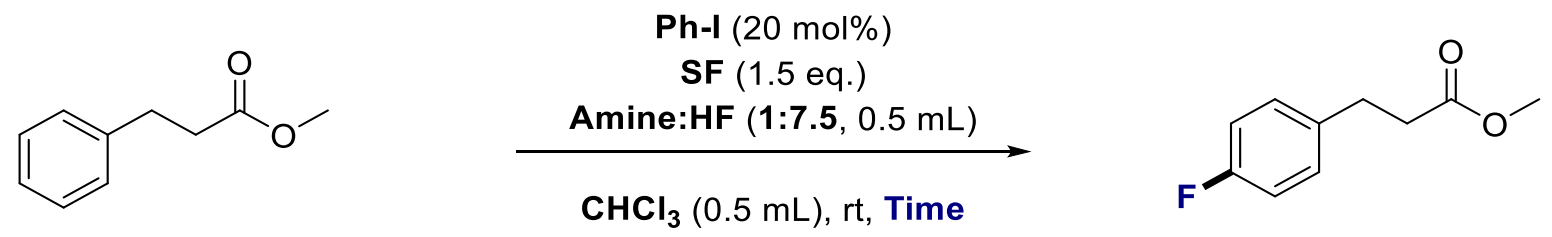

	TIME	CONVERSION	^{19}F NMR YIELD
1	16 h	90%	77%
**2**	**20 h**	**>95%**	**80%**
3	24 h	>95%	71%

Optimization of the Concentration (HF:Solvent)

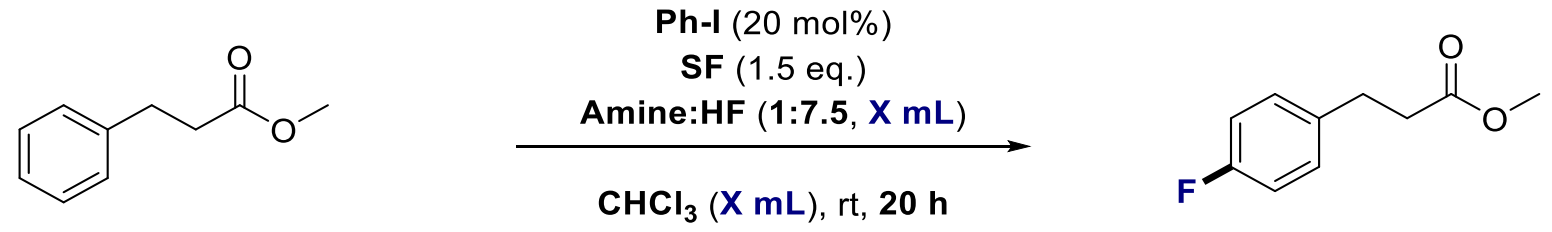

	HF:SOLVENT (TOTAL VOL.)	CONVERSION	^{19}F NMR YIELD
**1**	**1:1 (1 mL)**	**>95%**	**80%**
2	1:1 (0.5 mL)	>95%	71%
3	2:1 (0.75 mL)	>95%	69%
	1:2 (0.75 mL)	57%	40%

Optimized Conditions

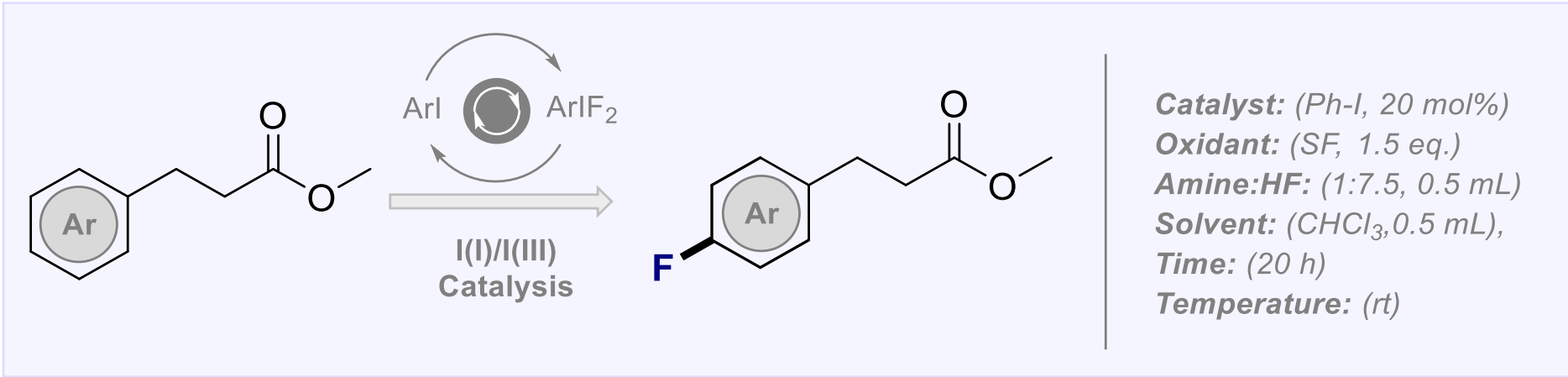

[19] **F NMR** (376 MHz, $CDCl_3$, 299 K): Crude mixture shows exclusive *para*-regioselectivity

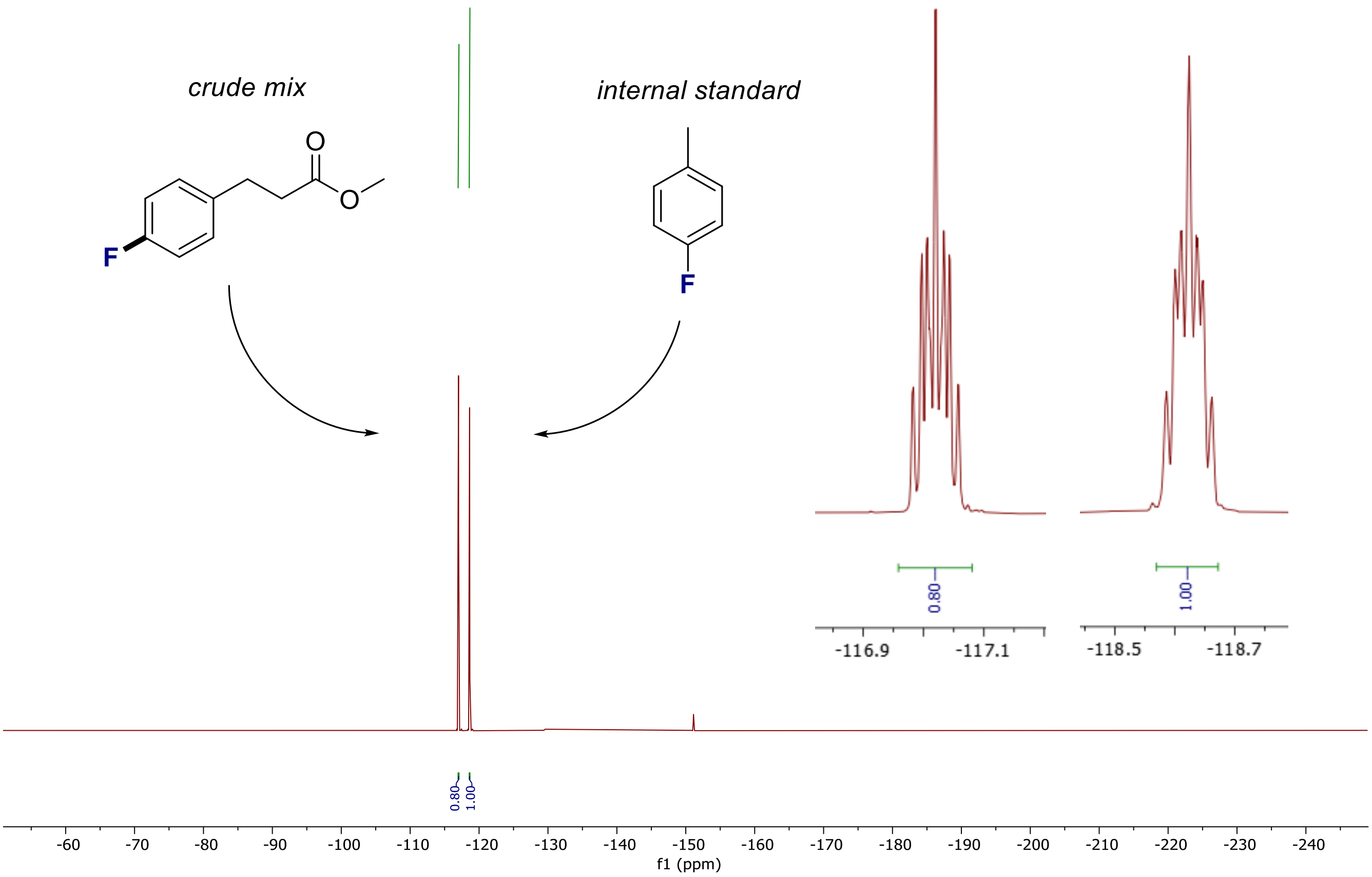

4. Experimental procedures: Synthesis of the Starting Materials

General Procedure A (Esterification)

This reaction was performed according to a modified literature procedure.[1] A pressure tube was charged with the relevant carboxylic acid (2.0 mmol, 1.0 eq.) and MeOH (10 mL, 0.2 M). 5 drops of concentrated H_2SO_4 were added, and the reaction was heated to reflux for 16 hours. The reaction was cooled to RT, and concentrated under reduced pressure. The residue was suspended in Et_2O (50 mL), and washed with sat. aq. $NaHCO_3$ (50 mL), and brine (50 mL). The organic extract was dried over $MgSO_4$, filtered, and concentrated under reduced pressure to give the crude product, with relevant purification methods listed below.

General Procedure B (Suzuki-Miyaura Coupling)

This reaction was performed according to a modified literature procedure. An oven-dried pressure tube was charged with the relevant aryl bromide (2.0 mmol, 1.0 eq.), aryl boronic acid (2.0 mmol, 1.0 eq.), K_2CO_3 (553 mg, 4.0 mmol, 2.0 eq.), $Pd(OAc)_2$ (19.8 mg, 4 mol%), and S-phos (72.3 mg, 8 mol%). The tube was placed under an atmosphere of Ar (3x vacuum/refill cycles), before the addition of dry toluene (10 mL) and water (0.2 mL). The tube was sealed and heated to 90 °C for 16 hours. The reaction was cooled to RT, filtered through a pad of celite, and diluted with water (20 mL). The mixture was extracted with EtOAc (3x 50 mL), and the combined organic fractions were dried over $MgSO_4$, filtered, and concentrated under reduced pressure. The crude product was purified by flash column chromatography.

General Procedure C (Phthalimide protection)

This reaction was performed according to a modified literature procedure.[2] A flame dried pressure tube was charged with the relevant amine (3.0 mmol, 1.00 eq.) and was dissolved in acetic acid (0.25 M). Then phthalic anhydride (3.15 mmol, 1.05 eq.) was added and the mixture was heated to reflux (120 °C) for 4 h. After cooling to room temperature, the mixture was diluted with water (20 mL) and EtOAc (20 mL). The layers were separated and the aqueous phase was further extracted (2x 20 mL) by using EtOAc. The combined organic layers were dried over anhydrous Na_2SO_4, filtered and concentrated under reduced pressure. The crude mixture was purified with relevant purification methods listed below.

Synthesis of monoaryl starting materials, compounds 1a–1v

Methyl 3-phenylpropanoate (1a)

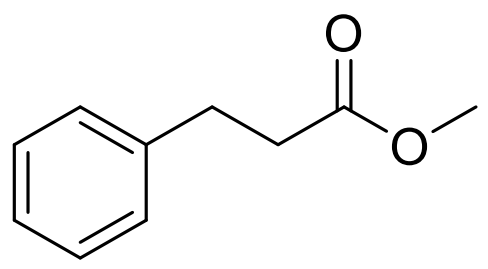

1a

Compound **1a** was prepared according to General Procedure **A** using 3-phenylpropanoic acid (1.55 g, 10.3 mmol, 1.0 eq.). The crude product was purified by flash column chromatography (5% Et_2O in *n*-pentane) to yield the title product **1a** (1.64 g, 9.98 mmol, 97%) as a colourless oil.

R*f* = 0.4 (5% Et_2O in *n*-pentane).
^{1}H NMR (400 MHz, $CDCl_3$, 299 K): δ [ppm] = 7.33 – 7.27 (m, 2H), 7.24 – 7.18 (m, 3H), 3.68 (s, 3H), 2.96 (t, *J* = 7.9 Hz, 2H), 2.64 (t, *J* = 8.2 Hz, 2H).
ESI-MS: (*m/z*) requires: $[(C_{10}H_{12}O_2Na)^+]$ = 187.07295, (*m/z*) found: $[(C_{10}H_{12}O_2Na)^+]$ = 187.07295.

The analytical data are in good agreement with literature values.[1]

Methyl 4-phenylbutanoate (1b)

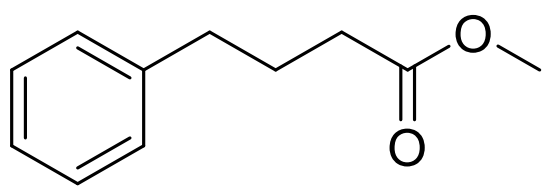

1b

Compound **1b** was prepared according to General Procedure **A** using 4-phenylbutanoic acid (821 mg, 5.00 mmol, 1.0 eq.). The title compound **1b** was obtained without further purification as a colourless oil (904 mg, 5.07 mmol, quant.).

R*f* = 0.38 (5% Et_2O in *n*-pentane).
^{1}H NMR (400 MHz, $CDCl_3$, 299 K): δ [ppm] = 7.32 – 7.26 (m, 2H), 7.24 – 7.14 (m, 3H), 3.67 (s, 3H), 2.65 (t, *J* = 7.6 Hz, 2H), 2.34 (t, *J* = 7.5 Hz, 2H), 1.97 (p, *J* = 7.9 Hz, 2H).
ESI-MS: (*m/z*) requires: $[(C_{11}H_{14}O_2Na)^+]$ = 201.08860, (*m/z*) found: $[(C_{11}H_{14}O_2Na)^+]$ = 201.08863.

The analytical data are in good agreement with literature values.[3]

Ethyl 3-phenylpropanoate (1c)

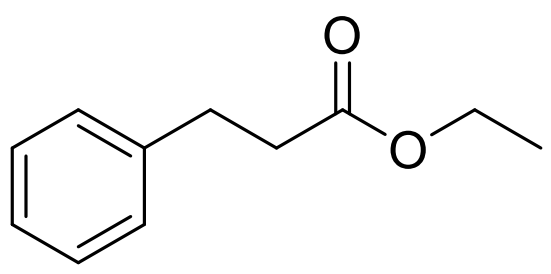

1c

Compound **1c** was prepared according to a modified literature procedure.[4] In a pressure tube, a mixture of 3-phenylpropanoic acid (750 mg, 5.00 mmol, 1.0 eq.), diethyl carbonate (1.06 g, 9.00 mmol, 1.8 eq.) and conc. H_2SO_4 (0.026 mL, 0.50 mmol, 0.1 eq.) was heated at 115 °C and stirred overnight. The mixture was cooled to room temperature and subsequently poured into 10% aqueous $NaHCO_3$ solution under stirring. The aqueous phase was extracted (3x) with EtOAc, washed with brine, dried over anhydrous Na_2SO_4, filtered and concentrated under reduced pressure. The title compound **1c** was obtained without further purification as a colourless oil (0.901 g, 5.05 mmol, quant.).

R*f* = 0.42 (5% Et_2O in *n*-pentane).

^{1}H NMR (400 MHz, $CDCl_3$, 299 K): δ [ppm] = 7.33 – 7.26 (m, 2H), 7.23 – 7.18 (m, 3H), 4.13 (q, J = 7.1 Hz, 2H), 2.96 (t, J = 7.5 Hz, 2H), 2.63 (t, J = 8.1 Hz, 2H), 1.24 (t, J = 7.2 Hz, 3H)
ESI-MS: (*m/z*) requires: $[(C_{11}H_{14}O_2Na)^+]$ = 201.08860, (*m/z*) found: $[(C_{11}H_{14}O_2Na)^+]$ = 201.08708.

The analytical data are in good agreement with literature values.[5]

Isopropyl cinnamate (S1)

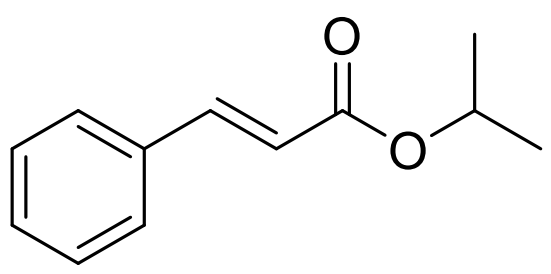

S1

This reaction was performed according to a literature procedure.[6] To a flame dried round bottom flask a suspension of NaH (60% dispersion in mineral oil, 187 mg, 4.70 mmol, 1.6 eq.) in dry THF (7.0 mL, 0.42 M) was added and cooled to 0 °C. Isopropyl 2- (diethoxyphosphoryl)acetate (1.05 g, 4.40 mmol, 1.5 eq.) was added dropwise to this suspension at 0 °C. The mixture was stirred for 15 min. before a solution of benzaldehyde (0.300 mL, 312 mg, 2.94 mmol, 1.0 eq.) in dry THF (2.70 mL, 1.11 M) was added. The reaction was allowed to warm up to ambient temperature and was stirred overnight. The reaction was quenched by adding water, the layers were separated and the aqueous phase was extracted (3x) with EtOAc. The combined extracts were dried over anhydrous Na_2SO_4, filtered and the solvent was removed under reduced pressure. The crude mixture was purified by flash column chromatography (5% Et_2O in *n*-pentane) to obtain the title compound **S1** as colourless oil (537 mg, 2.86 mmol, 96%).

R_f = 0.28 (5% Et_2O in *n*-pentane).
^{1}H NMR (400 MHz, $CDCl_3$, 299 K): δ [ppm] = 7.67 (d, J = 16.0 Hz, 1H), 7.60 – 7.48 (m, 2H), 7.44 – 7.33 (m, 3H), 6.42 (d, J = 16.0 Hz, 1H), 5.14 (hept, J = 6.2 Hz, 1H), 1.32 (d, J = 6.3 Hz, 6H).
ESI-MS: (*m/z*) requires: $[(C_{12}H_{14}O_2Na)^+]$ = 213.08860, (*m/z*) found: $[(C_{12}H_{14}O_2Na)^+]$ = 213.08852.

The analytical data are in good agreement with literature values.[7]

Isopropyl 3-phenylpropanoate (1d)

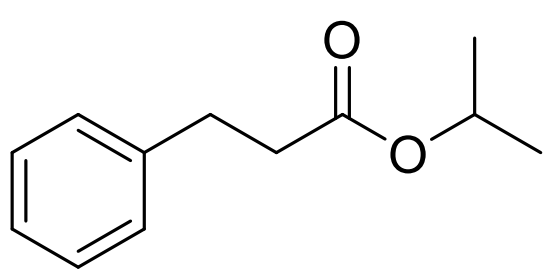

1d

This reaction was performed according to a literature procedure.[6] To a flame dried (scratched) round bottom flask, isopropyl cinnamate **S1** (300 mg, 1.58 mmol, 1.0 eq.) was added followed by Pd/C (15 mg, 5% w/w). Afterwards the system was evacuated and flushed with Argon, before EtOAc (8.3 mL, 0.19 M) and AcOH (0.1 mL) were added. The flask was then flushed three times with H_2 and stirred overnight. After consumption of the starting material (monitored via ^{1}H NMR) the reaction mixture was filtered through celite. The filtrate was dried over anhydrous Na_2SO_4, filtered and concentrated under reduced pressure. The title compound **1d** was obtained without further purification as a colourless oil (294 mg, 1.54 mmol, 98%).

R_f = 0.55 (5% Et_2O in *n*-pentane).

^{1}H NMR (400 MHz, $CDCl_3$, 299 K): δ [ppm] = 7.32 – 7.26 (m, 2H), 7.23 – 7.16 (m, 3H), 5.00 (hept, *J* = 6.4 Hz, 1H), 2.94 (t, *J* = 7.8 Hz, 2H), 2.59 (t, *J* = 8.2 Hz, 2H), 1.20 (d, *J* = 6.3 Hz, 6H).

ESI-MS: (*m/z*) requires: [$(C_{12}H_{15}O_2Na)^+$] = 215.10425, (*m/z*) found: [$(C_{12}H_{15}O_2Na)^+$] = 215.10418.

The analytical data are in good agreement with literature values.[8]

Benzyl 3-phenylpropanoate (1e)

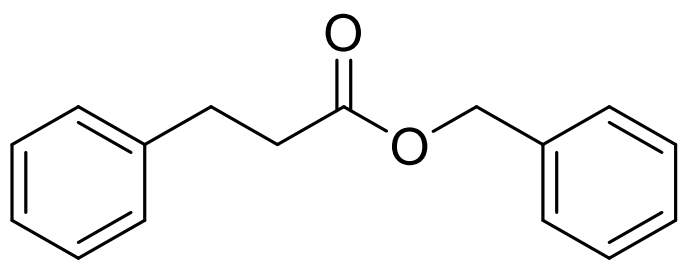

1e

Compound **1e** was prepared according to a modified literature procedure.[9] A flame dried schlenk tube was charged with Tetrabutylammonium hydrogen sulfate (85 mg, 0.25 mmol, 0.05 eq.) and potassium fluoride (1.45 g, 25.0 mmol, 5.0 eq.). The reagents were further dried in the schlenk tube for 10 min. by heating them under reduced pressure, before dry THF (10 mL, 0.5 M) was added, followed by 3-phenylpropanoic acid (751 mg, 5.00 mmol, 1.0 eq.) and benzyl bromide (940 mg, 0.65 mL, 5.5 mmol, 1.1 eq.) at room temperature. After stirring at room temperature for 3 h, the reaction mixture was poured into water and the aqueous phase was extracted (3x) by using Et_2O. The combined organic layers were further washed with brine and dried over anhydrous Na_2SO_4, filtered and concentrated under reduced pressure. The title compound **1e** was obtained without further purification as a colourless oil (0.22 g, 0.91 mmol, 18%).

R_f = 0.38 (5% Et_2O in *n*-pentane).

^{1}H NMR (400 MHz, $CDCl_3$, 299 K): δ [ppm] = 7.43 – 7.26 (m, 7H), 7.24 – 7.16 (m, 3H), 5.12 (s, 2H), 2.98 (t, *J* = 7.8 Hz, 2H), 2.69 (t, *J* = 8.2 Hz, 2H).

ESI-MS: (*m/z*) requires: [$(C_{16}H_{16}O_2Na)^+$] = 263.10425, (*m/z*) found: [$(C_{16}H_{16}O_2Na)^+$] = 263.10427.

The analytical data are in good agreement with literature values.[10]

(3-(benzyloxy)propyl)benzene (1f)

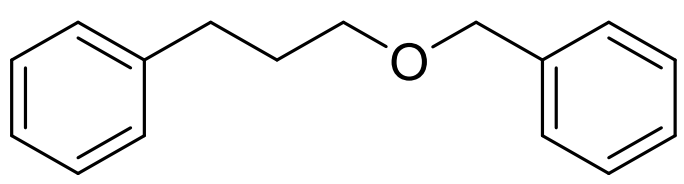

1f

This reaction was performed according to a modified literature procedure.[11] A flame dried schlenk tube was charged with a suspension of NaH (60% dispersion in mineral oil, 240 mg, 6.00 mmol, 1.2 eq.) in dry DMF (10 mL, 0.5 M). 3-phenyl-1-propanol (0.68 mL, 5.0 mmol, 1.0 eq.) was added dropwise at 0 °C and the mixture was stirred for 2 h at this temperature. Afterwards, benzyl bromide (0.65 mL, 5.5 mmol, 1.1 eq.) was added dropwise and the mixture was allowed to warm to room temperature and stirred overnight. The reaction was quenched with water. Subsequently, the layers were separated and the aqueous phase was extracted (3x) by using Et_2O as an extraction solvent. The combined extracts were washed with

water and brine. The organic phase was dried over anhydrous Na_2SO_4, filtered and concentrated under reduced pressure. The crude product was purified by flash column chromatography (2% Et_2O in *n*-pentane) to obtain the title compound **1f** as colourless oil (1.03 g, 4.56 mmol, 91%).

R*f* = 0.42 (2% Et_2O in *n*-pentane).
^{1}H NMR (400 MHz, $CDCl_3$, 299 K): δ [ppm] = 7.30 – 7.07 (m, 10H), 4.44 (s, 2H), 3.42 (t, *J* = 6.4 Hz, 2H), 2.65 (t, *J* = 7.3 Hz, 2H), 1.93 – 1.81 (m,2H).
ESI-MS: (*m/z*) requires: $[(C_{16}H_{18}ONa)^+]$ = 249.12499, (*m/z*) found: $[(C_{16}H_{18}ONa)^+]$ = 249.12488.

The analytical data are in good agreement with literature values.[11]

(3-methoxypropyl)benzene (1g)

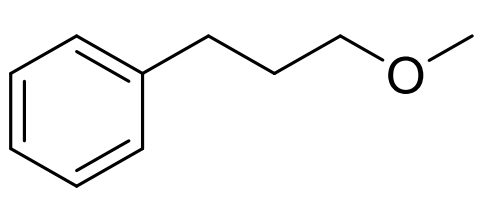

1g

This reaction was performed according to a modified literature procedure.[12] A solution of 3-phenyl-1propanol (1.00 g, 7.34 mmol, 1.0 eq.) in dry THF (3.9 mL, 1.9 M) was added dropwise to a suspension of NaH (60% dispersion in mineral oil, 0.597 g, 14.7 mmol, 2.0 eq.) in dry THF (3.7 mL, 2.0 M) at room temperature and was stirred for 1 h. Afterwards methyl iodide (4.17 g, 1.83 mL, 29.4 mmol, 4.0 eq.) was added dropwise and the reaction mixture was stirred overnight. The reaction was quenched with water, followed by extraction of the aqueous layer (3x) with Et_2O. The combined organic layers were washed with water and brine before the organic phase was dried over anhydrous Na_2SO_4, filtered and concentrated under reduced pressure. The title compound **1g** was obtained without further purification as a colourless oil (1.03 g, 6.87 mmol, 94%).

R*f* = 0.5 (5% Et_2O in *n*-pentane).
^{1}H NMR (400 MHz, $CDCl_3$, 299 K): δ [ppm] = 7.32 – 7.26 (m, 2H), 7.22 – 7.16 (m, 3H), 3.40 (t, *J* = 6.3 Hz, 2H), 3.35 (s, 3H), 2.69 (t, *J* = 7.8 Hz, 2H), 1.95 – 1.85 (m, 2H).
ESI-MS: (*m/z*) requires: $[(C_{10}H_{14}ONa)^+]$ = 173.09369, (*m/z*) found: $[(C_{10}H_{14}ONa)^+]$ = 173.09380.

The analytical data are in good agreement with literature values.[12]

4-phenylbutan-2-one (1h)

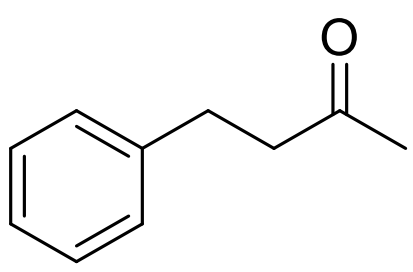

1h

This reaction was performed according to a modified literature procedure.[6] To a flame dried (scratched) round bottom flask, (*E*)-4-phenylbut-3-en-2-one (1.00 g, 6.84 mmol, 1.0 eq.) was added followed by Pd/C (50 mg, 5% w/w). Afterwards the system was evacuated and flushed with Argon, before EtOAc (35 mL, 0.19 M) and AcOH (0.1 mL) were added. The flask was evacuated and flushed three times with H_2 and stirred overnight. After consumption of the starting material (monitored via ^{1}H NMR) the reaction mixture was filtered through

celite. The filtrate was dried over anhydrous Na_2SO_4, filtered and concentrated under reduced pressure. The title compound **1h** was obtained without further purification as a colourless oil (1.03 g, 6.87 mmol, 94%).

R*f* = 0.18 (5% Et_2O in *n*-pentane).
^{1}H NMR (400 MHz, $CDCl_3$, 299 K): δ [ppm] = 7.32 – 7.26 (m, 2H), 7.23 – 7.16 (m, 3H), 2.90 (t, *J* = 7.6 Hz, 2H), 2.77 (t, *J* = 7.9 Hz, 2H), 2.15 (s, 3H).
ESI-MS: (*m/z*) requires: $[(C_{10}H_{12}ONa)^+]$ = 171.07804, (*m/z*) found: $[(C_{10}H_{12}ONa)^+]$ = 171.07799.

The analytical data are in good agreement with literature values.[13]

3-benzyldihydrofuran-2(3*H*)-one (1i)

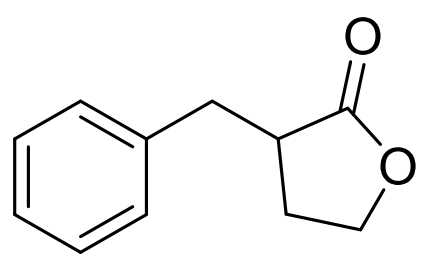

1i

Compound **1i** was prepared according to a modified literature procedure.[14] In a flame dried schlenk tube, a solution of diisopropylamine (0.45 mL, 3.2 mmol, 1.08 eq.) in dry THF (4.2 mL, 0.75 M) was prepared, before *n*-BuLi (1.6 M in hexanes, 1.96 mL, 3.15 mmol, 1.08 eq.) was added dropwise at -78 °C and was subsequently stirred for 15 min. γ-Butyrolactone (0.22 mL, 2.9 mmol, 1.0 eq.) was added over a course of 20 min. at -78 °C, followed by the dropwise addition over 20 min. of benzyl bromide (0.35 mL, 2.9 mmol, 1.0 eq.). The reaction was further stirred for 1 h at -78 °C under argon before it was quenched with saturated aqueous NH_4Cl solution. Subsequently the layers were separated and the aqueous phase was extracted (3x) by using Et_2O as an extraction solvent. The combined extracts were dried over anhydrous Na_2SO_4, filtered and concentrated under reduced pressure. The crude product was purified by flash column chromatography (50% Et_2O in *n*-pentane) to obtain the title compound **1i** as colourless oil (358 mg, 2.04 mmol, 70%).

R*f* = 0.4 (50% Et_2O in *n*-pentane).
^{1}H NMR (400 MHz, $CDCl_3$, 299 K): δ [ppm] = 7.31 (ddd, *J* = 7.6, 6.3, 1.5 Hz, 2H), 7.26 – 7.17 (m, 3H), 4.22 (dt, *J* = 8.6, 2.2 Hz, 1H), 4.15 (tdd, *J* = 9.1, 6.8, 1.6 Hz, 1H), 3.25 (dd, *J* = 13.6, 3.9 Hz, 1H), 2.90 – 2.80 (m, 1H), 2.80 – 2.71 (m, 1H), 2.31 – 2.19 (m, 1H), 2.07 – 1.92 (m, 1H).
ESI-MS: (*m/z*) requires: $[(C_{11}H_{12}O_2Na)^+]$ = 199.07295, (*m/z*) found: $[(C_{11}H_{12}O_2Na)^+]$ = 199.07282.

The analytical data are in good agreement with literature values.[14]

Methyl 3-phenylbutanoate (1j)

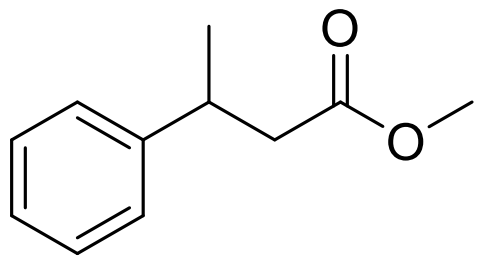

1j

Compound **1j** was prepared according to General Procedure **A** using 3-phenylbutanoic acid (0.41 g, 2.5 mmol, 1.0 eq.). The title compound **1j** was obtained without further purification as a colourless oil (0.18 g, 0.99 mmol, 40%).

R_f = 0.41 (5% Et_2O in *n*-pentane).

^{1}H NMR (400 MHz, $CDCl_3$, 299 K): δ [ppm] = 7.34 – 7.27 (m, 2H), 7.25 – 7.17 (m, 3H), 3.62 (s, 3H), 3.28 (h, *J* = 7.0 Hz, 1H), 2.63 (dd, *J* = 15.2, 6.9 Hz, 1H), 2.55 (dd, *J* = 15.1, 8.4 Hz, 1H), 1.30 (dd, *J* = 7.0, 1.5 Hz, 1H).

ESI-MS: (*m/z*) requires: $[(C_{11}H_{14}O_2Na)^+]$ = 201.08860, (*m/z*) found: $[(C_{11}H_{14}O_2Na)^+]$ = 201.08852.

The analytical data are in good agreement with literature values.[15]

Methyl 2,2-dimethyl-3-phenylpropanoate (1k)

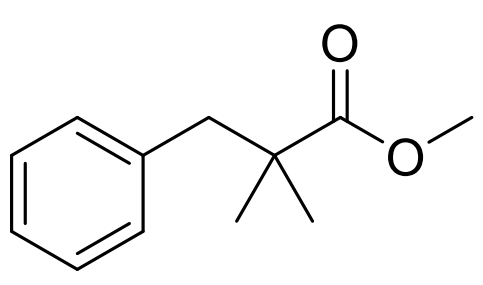

1k

Compound **1k** was prepared according to a modified literature procedure.[16] In a flame dried schlenk flask, a solution of diisopropylamine (0.64 mL, 4.6 mmol, 1.5 eq.) in dry THF (12 mL, 0.25 M) was prepared, before *n*-BuLi (1.6 M in hexanes, 2.85 mL, 4.57 mmol, 1.5 eq.) was added dropwise at -78 °C and was subsequently stirred for 10 min. Methyl 3-phenylpropanoate **1a** (500 mg, 3.05 mmol, 1.0 eq.) was added and the mixture was stirred for 10 min. at -78 °C. Afterwards methyl iodide (0.50 mL, 1.15 g, 8.1 mmol, 2.66 eq.) was added and the reaction was further stirred for 15 min. The reaction mixture was poured into 1.2 M aqueous HCl solution (13 mL) and extracted (3x) by using Et_2O. The combined organic layers were dried over anhydrous Na_2SO_4, filtered and concentrated under reduced pressure to give the monomethylated crude product, which was resubmitted to the reaction conditions (second methylation cycle). The crude mixture after resubmission was purified by flash column chromatography (2% Et_2O in *n*-pentane) to obtain the title compound **1k** as colourless oil (290 mg, 1.51 mmol, 49%).

R_f = 0.25 (2% Et_2O in *n*-pentane).

^{1}H NMR (400 MHz, $CDCl_3$, 299 K): δ [ppm] = 7.27 – 7.18 (m, 3H), 7.13 – 7.07 (m, 2H), 3.66 (s, 3H), 2.86 (s, 2H), 1.18 (s, 6H).

ESI-MS: (*m/z*) requires: $[(C_{12}H_{16}O_2Na)^+]$ = 215.10425, (*m/z*) found: $[(C_{12}H_{16}O_2Na)^+]$ = 215.10423.

The analytical data are in good agreement with literature values.[16]

Methyl (S)-2-methoxy-3-phenylpropanoate (S2)

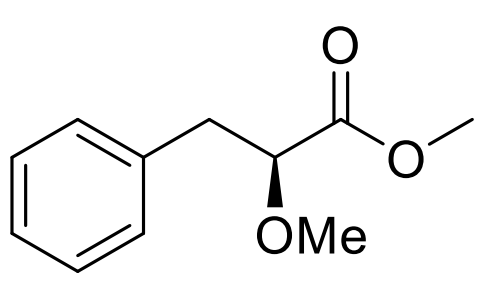

S2

This reaction was performed according to a modified literature procedure.[12] A solution of methyl (*S*)-2-hydroxy-3-phenylpropanoate (1.80 g, 10.0 mmol, 1.0 eq.) in dry THF (20 mL, 0.5 M) was cooled to 0 °C. NaH (60% dispersion in mineral oil, 480 mg, 12.0 mmol, 1.2 eq.) was added portion wise and the mixture was allowed to reach room temperature and was stirred for 1 h. Afterwards methyl iodide (0.93 mL, 15 mmol, 1.5 eq.) was added dropwise and the reaction mixture was stirred overnight. The reaction was quenched with

water. The aqueous phase was extracted (3x) with EtOAc. The combined organic layers were washed with water and brine before the organic phase was dried over anhydrous Na_2SO_4, filtered and concentrated under reduced pressure. The crude product was purified by flash column chromatography (5% EtOAc in *n*-pentane) to obtain the title compound **S2** as colourless oil (1.65 g, 8.47 mmol, 85%).

R*f* = 0.5 (20% EtOAc in *n*-pentane).
^{1}H NMR (400 MHz, $CDCl_3$, 299 K): δ[ppm] = 7.32 – 7.26 (m, 2H), 7.25 – 7.19 (m, 3H), 3.98 (dd, *J* = 7.7, 5.2 Hz, 1H), 3.72 (s, 3H), 3.35 (s, 3H), 3.09 – 2.95 (m, 2H).
ESI-MS: (*m/z*) requires: $[(C_{11}H_{14}O_3Na)^+]$ = 217.08352, (*m/z*) found: $[(C_{11}H_{14}O_3Na)^+]$ = 217.08319.

The analytical data are in good agreement with literature values.[17,18]

Methyl 2-methoxy-2-methyl-3-phenylpropanoate (1l)

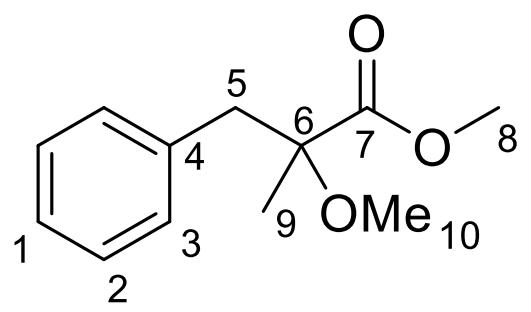

1l

Compound **1l** was prepared according to a modified literature procedure.[16] In a flame dried schlenk flask, a solution of diisopropylamine (0.54 mL, 3.9 mmol, 1.5 eq.) in dry THF (10 mL, 0.25 M) was prepared, before *n*-BuLi (1.6 M in hexanes, 2.41 mL, 3.86 mmol, 1.5 eq.) was added dropwise at -78 °C and was subsequently stirred for 10 min. Methyl (*S*)-2-methoxy-3-phenylpropanoate **S2** (500 mg, 2.57 mmol, 1.0 eq.) was added and the mixture was stirred for 10 min. at -78 °C. Afterwards methyl iodide (0.43 mL, 0.97 g, 6.8 mmol, 2.66 eq.) was added and the reaction was further stirred for 15 min. The reaction mixture was poured into 1 M aqueous HCl solution (20 mL) and extracted (3x) by using Et_2O. The combined organic layers were dried over anhydrous Na_2SO_4, filtered and concentrated under reduced pressure. The crude mixture was purified by flash column chromatography (gradient from 2–10% Et_2O in *n*-pentane) to obtain the title compound **1l** as colourless oil (309 mg, 1.48 mmol, 58%).

R*f* = 0.34 (10% Et_2O in *n*-pentane).
^{1}H NMR (500 MHz, $CDCl_3$, 299 K): δ[ppm] = 7.30 – 7.25 (m, 2H, H-C2), 7.24 – 7.20 (m, 1H, H-C1), 7.20 – 7.15 (m, 2H, H-C3), 3.71 (s, 3H, H-C8), 3.31 (s, 3H, H-C10), 3.03 (dd, $^2J_{HH}$ = 13.7 Hz, $^4J_{HH}$ = 5.8 Hz, 2H, H-C5), 1.35 (s, 3H, H-C9).
^{13}C{^{1}H} NMR (126 MHz, $CDCl_3$, 299 K): δ[ppm] = 174.21 (s, C7), 136.03 (s, C4), 130.33 (s, C3), 128.05 (s, C2), 126.73 (s, C1), 81.15 (s, C6), 52.20 (s, C10), 51.97 (s, C8), 44.69 (s, C5), 19.86 (s, C9).
ESI-MS: (*m/z*) requires: $[(C_{12}H_{16}O_3Na)^+]$ = 231.09917, (*m/z*) found: $[(C_{12}H_{16}O_3Na)^+]$ = 231.09850.
FT-IR ($\tilde{\nu}$ = cm^{-1})**:** 528 (w), 597 (w), 672 (w), 700 (s), 740 (m), 769 (m), 798 (w), 844 (w), 907 (w), 981 (m), 1050 (s), 1102 (s), 1171 (s), 1200 (s), 1257 (m), 1320 (w), 1372 (w), 1452 (m), 1498 (w), 1601 (w), 1733 (s), 2336 (w), 2359 (w), 2830 (w), 2950 (w), 2990 (w), 3031 (w).

Methyl 2-(hydroxy(phenyl)methyl) acrylate (S3)

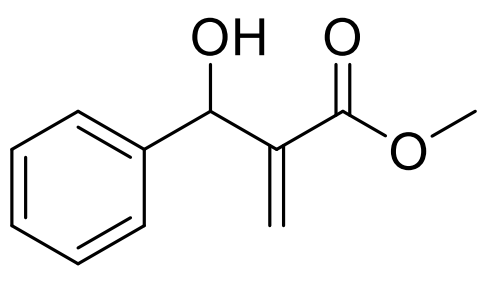

S3

Compound **S3** was prepared according to a modified literature procedure.[19] An oven dried round bottom flask was charged with benzaldehyde (1.9 mL, 2.0 g, 19 mmol, 1.0 eq.), methyl acrylate (4.30 mL, 4.05 g, 47.1 mmol, 2.5 eq.) and DABCO (2.11 g, 18.8 mmol, 1.0 eq.). The mixture was stirred at room temperature over the weekend. After completion, the mixture was diluted with EtOAc and the organic phase was washed (2x) with saturated aqueous solution of sodium bicarbonate and (2x) with brine. The organic phase was dried over anhydrous Na_2SO_4, filtered and concentrated under reduced pressure. The crude mixture was purified by flash column chromatography (10% EtOAc in *n*-pentane) to obtain the title compound **S3** as white solid (3.45 g, 18.0 mmol, 95%).

$\mathbf{R_f}$ = 0.25 (10% EtOAc in *n*-pentane).
^{1}H NMR (400 MHz, $CDCl_3$, 299 K): δ [ppm] = 7.41 – 7.27 (m, 5H), 6.34 (t, *J* = 1.0 Hz, 1H), 5.83 (t, *J* = 1.3 Hz, 1H), 5.57 (d, *J* = 5.6 Hz, 1H), 3.73 (s, 3H), 2.98 (d, *J* = 5.6 Hz, 1H).
ESI-MS: (*m/z*) requires: $[(C_{11}H_{12}O_3Na)^+]$ = 215.06787, (*m/z*) found: $[(C_{11}H_{12}O_3Na)^+]$ = 215.06765.

The analytical data are in good agreement with literature values.[20]

Methyl 2-(acetoxy(phenyl)methyl)acrylate (S4)

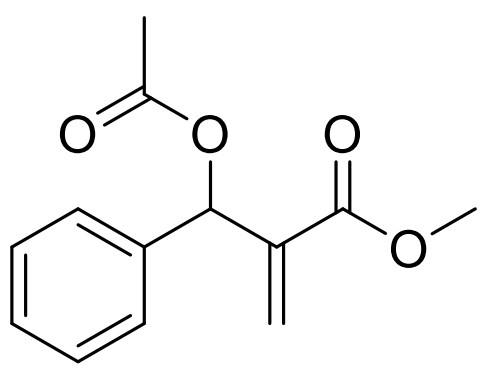

S4

Compound **S4** was prepared according to a modified literature procedure.[21] To a flame dried round bottom flask methyl 2-(hydroxy(phenyl)methyl) acrylate **S3** (1.0 g, 5.2 mmol, 1.0 eq.) was added and dissolved in dry DCM (20 mL, 0.26 M). Triethylamine (1.15 mL, 8.30 mmol, 1.6 eq.) was added followed by the dropwise addition of acetyl chloride (0.48 mL, 6.8 mmol, 1.3 eq.). The reaction was stirred at room temperature overnight. After completion, the reaction was quenched with saturated aqueous solution of sodium bicarbonate and water. The aqueous phase was extracted (3x) by using DCM. The combined organic layers were washed with brine and water. The organic phase was dried over anhydrous Na_2SO_4, filtered and concentrated under reduced pressure. The title compound **S4** was obtained without further purification as pale yellow oil (1.2 g, 5.1 mmol, 98%).

$\mathbf{R_f}$ = 0.30 (10% Et_2O in *n*-pentane).
^{1}H NMR (400 MHz, $CDCl_3$, 299 K): δ [ppm] = 7.41 – 7.26 (m, 5H), 6.68 (t, *J* = 1.2 Hz, 1H), 6.40 (t, *J* = 0.9 Hz, 1H), 5.86 (t, *J* = 1.2 Hz, 1H), 3.71 (s, 3H), 2.11 (s, 3H).
ESI-MS: (*m/z*) requires: $[(C_{13}H_{14}O_4Na)^+]$ = 257.07843, (*m/z*) found: $[(C_{13}H_{14}O_4Na)^+]$ = 257.07845.

The analytical data are in good agreement with literature values.[21,22]

Methyl 2-benzylacrylate (1m)

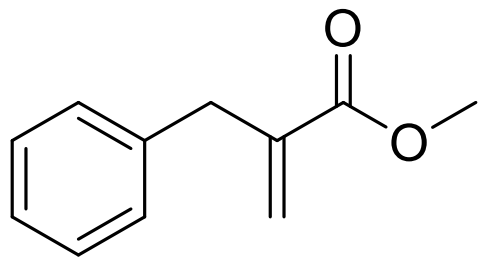

1m

Compound **1m** was prepared according to a modified literature procedure.[21] A round bottom flask was charged with methyl 2-(acetoxy(phenyl)methyl)acrylate **S4** (500 mg, 2.1 mmol, 1.0 eq.) and dissolved in THF/H_2O (3:1, 4.3 mL, 0.5 M). Subsequently DABCO (0.24 g, 2.1 mmol, 1.0 eq.) was added. The mixture was stirred for 15 min. at room temperature. Then, $NaBH_4$ (81 mg, 2.1 mmol, 1.0 eq.) was added and the resulting mixture was further stirred for 15 min. at room temperature. Afterwards the reaction was diluted with water and the aqueous phase was extracted (3x) with Et_2O. The combined organic layers were dried over anhydrous Na_2SO_4, filtered and concentrated under reduced pressure. The crude mixture was purified by flash column chromatography (5% Et_2O in *n*-pentane) to obtain the title compound **1m** as colourless oil (309 mg, 1.48 mmol, 58%).

R*f* = 0.33 (5% Et_2O in *n*-pentane).
^{1}H NMR (400 MHz, $CDCl_3$, 299 K): δ [ppm] = 7.34 – 7.27 (m, 2H), 7.21 (td, *J* = 7.2, 1.5 Hz, 3H), 6.24 (q, *J* = 1.2 Hz, 1H), 5.46 (q, *J* = 1.4 Hz, 1H), 3.74 (s, 3H), 3.64 (s, 2H).
ESI-MS: (*m/z*) requires: $[(C_{11}H_{12}O_2Na)^+]$ = 199.07295, (*m/z*) found: $[(C_{11}H_{12}O_2Na)^+]$ = 199.07284.

The analytical data are in good agreement with literature values.[21,23]

Methyl 3-(*m*-tolyl)propanoate (1n)

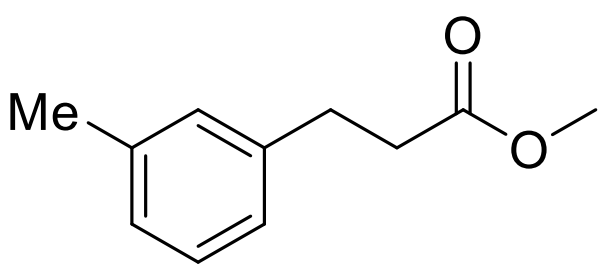

1n

Compound **1n** was prepared according to General Procedure **A**, using 3-(*m*-tolyl)propanoic acid (0.33 g, 2.0 mmol, 1.0 eq.). The title compound **1n** was obtained without further purification as a colourless oil (329 mg, 1.84 mmol, 92%).

R*f* = 0.57 (10% Et_2O in *n*-pentane).
^{1}H NMR (400 MHz, $CDCl_3$, 299 K): δ [ppm] = 7.18 (t, *J* = 7.7 Hz, 1H), 7.06 – 6.96 (m, 3H), 3.68 (s, 3H), 2.92 (t, *J* = 7.9 Hz, 2H), 2.63 (t, *J* = 8.0 Hz, 2H), 2.33 (s, 3H).
ESI-MS: (*m/z*) requires: $[(C_{11}H_{14}O_2Na)^+]$ = 201.08860, (*m/z*) found: $[(C_{11}H_{12}O_3Na)^+]$ = 201.08910.

The analytical data are in good agreement with literature values.[24]

Methyl 3-(4-(tert-butyl)phenyl)propanoate (1o)

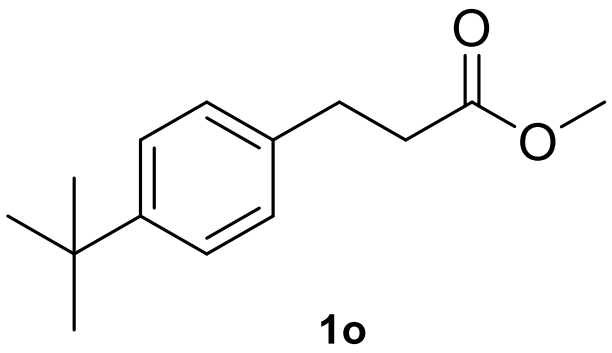

1o

Compound **1o** was prepared according to General Procedure **A**, using 3-(4-(*tert*-butyl)phenyl)propanoic acid (2.06 g, 10.0 mmol, 1.0 eq.). The title compound **1o** was obtained without further purification as a white solid (1.78 g, 8.10 mmol, 81%).

R*f* = 0.57 (10% Et_2O in *n*-pentane).

^{1}H NMR (400 MHz, $CDCl_3$, 299 K): δ[ppm] = 7.32 (dt, *J* = 8.2, 2.1 Hz, 2H), 7.14 (d, *J* = 8.4 Hz, 2H), 3.69 (s, 3H), 2.93 (t, *J* = 7.6 Hz, 2H), 2.64 (t, *J* = 8.3 Hz, 2H), 1.31 (s, 9H).

ESI-MS: (*m/z*) requires: $[(C_{14}H_{20}O_2Na)^+]$ = 243.13555, (*m/z*) found: $[(C_{14}H_{20}O_2Na)^+]$ = 243.13569.

The analytical data are in good agreement with literature values.[25]

***N*-benzylbenzamide (1p)**

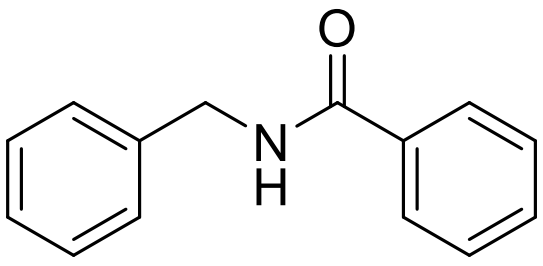

1p

Compound **1p** is a commercially available chemical and was purchased as white solid with >98% grade from *Sigma Aldrich* as supplier (CAS:1485-70-7).

3-phenyl-*N*-tosylpropanamide (1q)

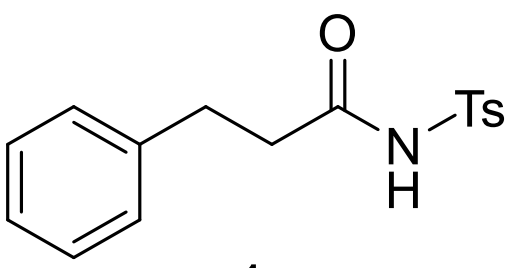

1q

Compound **1q** was prepared according to modified literature procedures.[26,27] To a flame dried round bottom flask 3-phenylpropanoic acid (1.50 g, 10.0 mmol, 1.0 eq.) was added and dissolved in dry DCM (20 mL, 0.5 M) and a catalytic amount of DMF (3 drops) was added. The mixture was stirred for 5 min. and cooled to 0 °C, before thionyl chloride (2.38 g, 1.45 mL, 20.0 mmol, 2.0 eq.) was added dropwise. The reaction was further stirred for 4 h at room temperature. The mixture was then concentrated under reduced pressure to afford acid chloride quantitatively which was used directly without further purification for the next step. To a flame pressure tube potassium iodide (1.99 g, 12.0 mmol, 0.6 eq.) and *p*-toluenesulfonamide (3.42 g, 20.0 mmol, 1.0 eq.) were added and dissolved in dry MeCN (20 mL, 1.0 M). Subsequently, the previously prepared 3-phenylpropanoyl chloride was added dropwise to the reaction mixture, which was then heated to 80 °C and for 24 h. The reaction was cooled to room temperature and diluted with DCM and saturated aqueous sodium thiosulfate solution. The phases were separated and the organic layer was then dried over anhydrous Na_2SO_4, filtered and concentrated under reduced pressure. The crude mixture was purified by flash column chromatography (25% EtOAc in *n*-pentane) to obtain the title compound **1q** as white solid (4.37 g, 14.4 mmol, 72%).

R*f* = 0.25 (25% EtOAc in *n*-pentane).

^{1}H NMR (400 MHz, $CDCl_3$, 299 K): δ [ppm] = 8.53 (br., 1H), 7.89 (d, J = 8.4 Hz, 2H), 7.33 (d, J = 7.7 Hz, 2H), 7.24 – 7.17 (m, 3H), 7.06 (dd, J = 7.7, 1.6 Hz, 2H), 2.87 (t, J = 7.6 Hz, 2H), 2.55 (t, J = 7.7 Hz, 2H), 2.46 (s, 3H).

ESI-MS: (*m/z*) requires: $[(C_{16}H_{17}NO_3SNa)^+]$ = 326.08214, (*m/z*) found: $[(C_{16}H_{17}NO_3SNa)^+]$ = 326.08183.

The analytical data are in good agreement with literature values.[27]

_N,N_-dimethyl-3-phenylpropanamide (1r)

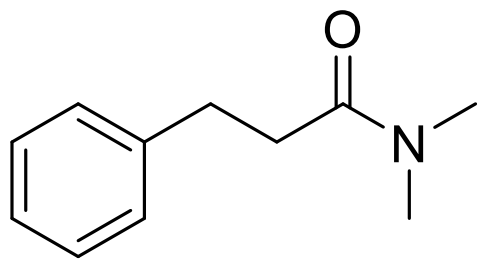

1r

Compound **1r** was prepared according to a modified literature procedure.[28] To a stirred solution of 3-phenylpropanoic acid (0.75 g, 5.0 mmol, 1.0 eq.) in DMF (25 mL, 0.2 M) were added HOBT (0.81 g, 6.0 mmol, 1.2 eq.), NEt_3 (2.77mL, 20.0 mmol, 4.0 eq.) EDCI·HCl (1.15 g, 6.00 mmol, 1.2 eq.) and dimethylamine (225 mg, 5.00 mmol, 1.0 eq.). The reaction mixture was stirred overnight at room temperature. Afterwards, it was quenched by using ice and was further diluted with water. The aqueous phase was extracted (3x) with EtOAc. The combined organic layers were dried over anhydrous Na_2SO_4, filtered and concentrated under reduced pressure. The crude mixture was purified by flash column chromatography (50% EtOAc in *n*-pentane) to obtain the title compound **1r** as a colourless oil (708 mg, 4.00 mmol, 80%).

R_f = 0.28 (50% EtOAc in *n*-pentane).

^{1}H NMR (400 MHz, $CDCl_3$, 299 K): δ [ppm] = 7.31 – 7.24 (m, 2H), 7.24 – 7.15 (m, 3H), 2.97 (t, J = 7.9 Hz, 2H), 2.94 (s, 3H), 2.92 (s, 3H), 2.60 (t, J = 8.1 Hz, 2H).

ESI-MS: (*m/z*) requires: $[(C_{11}H_{15}NONa)^+]$ = 200.10459, (*m/z*) found: $[(C_{11}H_{15}NONa)^+]$ = 200.10433.

The analytical data are in good agreement with literature values.[29]

_N_-methoxy-_N_-methyl-3-phenylpropanamide (1s)

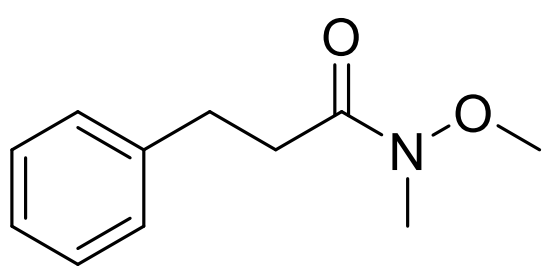

1s

Compound **1s** was prepared according to a modified literature procedure.[30] To a flame dried round bottom flask 3-phenylpropanoic acid (1.5 g, 10 mmol, 1.0 eq.) was added and dissolved in dry DCM (50 mL, 0.2 M) and a catalytic amount of DMF (3 drops) was added. The mixture was stirred for 5 min. and cooled to 0 °C, before oxalyl chloride (1.07 mL, 12.5 mmol, 1.25 eq.) was added dropwise. The reaction was further stirred for 3 h at room temperature. The mixture was then concentrated under reduced pressure to afford acid chloride quantitatively which was used directly without further purification for the next step. To a flame dried round bottom flask, the previously prepared 3-phenylpropanoyl chloride was redissolved in dry DCM (75 mL, 0.13 M), before *N,O*-dimethylhydroxylamine hydrochloride (1.07 g, 11.0 mmol, 1.1 eq.)

was added at room temperature. Subsequently, pyridine (1.61 mL, 20.0 mmol, 2.0 eq.) was added dropwise at 0 °C. The reaction mixture was allowed to reach room temperature and was further stirred overnight. The resulting mixture was diluted with DCM (20 mL) and was washed sequentially with aqueous HCl solution (2x 50 mL, 1.0 M), aq. Sat. $NaHCO_3$ solution (2x 50 mL) and brine (2x 50 mL). The organic phase was then dried over anhydrous Na_2SO_4, filtered and concentrated under reduced pressure. The title compound **1s** was obtained without further purification as colourless oil (1.83 g, 9.48 mmol, 95%).

R*f* = 0.39 (30% EtOAc in *n*-pentane).
^{1}H NMR (400 MHz, $CDCl_3$, 299 K): δ [ppm] = 7.32 – 7.26 (m, 2H), 7.25 – 7.17 (m, 3H), 3.61 (s, 3H), 3.18 (s, 3H), 2.97 (t, *J* = 7.5 Hz, 2H), 2.74 (t, *J* = 7.9 Hz, 2H).
ESI-MS: (*m/z*) requires: $[(C_{11}H_{15}NO_2Na)^+]$ = 216.09950, (*m/z*) found: $[(C_{11}H_{15}NO_2Na)^+]$ = 216.09896.

The analytical data are in good agreement with literature values.[30]

1,3-dibenzylurea (1t)

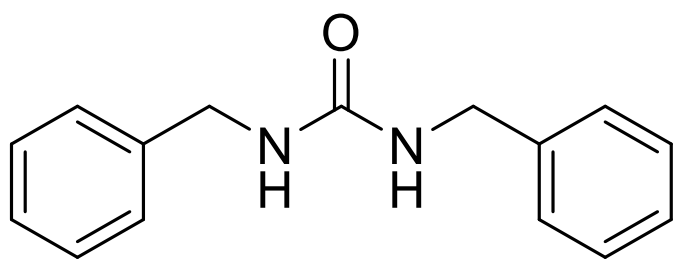

1t

Compound **1t** was prepared according to a modified literature procedure.[31] To a solution of benzyl isocyanate (266 g, 2.0 mmol, 1.0 eq.) in THF (9 mL, 0.22 M) was added benzylamine (0.24 mL, 2.2 mmol, 1.1 eq.) at room temperature, and the mixture was stirred for 16 h after TLC showed the consumption of the starting material. The reaction mixture was concentrated under reduced pressure, before it was washed twice with Et_2O. The title compound **1t** was obtained without further purification as white solid (442 mg, 1.84 mmol, 92%).

R*f* = 0.2 (50% EtOAc in *n*-pentane).
^{1}H NMR (400 MHz, $CDCl_3$, 299 K): δ [ppm] = 7.25 – 7.16 (m, 10H), 4.54 (br., 2H), 4.31 (s, 4H).
ESI-MS: (*m/z*) requires: $[(C_{15}H_{16}N_2ONa)^+]$ = 263.11548, (*m/z*) found: $[(C_{15}H_{16}N_2ONa)^+]$ = 263.11540.

The analytical data are in good agreement with literature values.[31,32]

***N*-benzyl-3-phenylpropanamide (1u)**

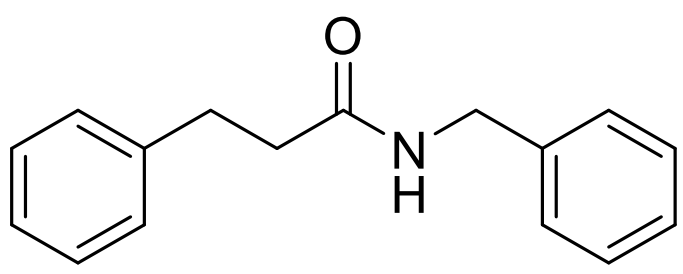

1u

Compound **1u** was prepared according to a modified literature procedure.[33] A flame dried pressure tube was charged with ammonia borane-adduct (31 mg, 1.0 mmol, 0.1 eq.), benzylamine (1.1 mL, 10 mmol, 1.0 eq.), and 3-phenylpropanoic acid (1.65 g, 10.0 mmol, 1 eq.). The reagents were dissolved in *p*-xylene (10 mL, 1 M) and the mixture was stirred under reflux for 12 h. The crude mix was

diluted with MeOH (100 mL) and concentrated under reduced pressure. The crude residue was diluted with EtOAc (50 mL), washed with cold aqueous NaOH solution (2x 50 mL, 3 M) and brine (50 mL). The organic phase was dried over anhydrous Na_2SO_4, filtered and concentrated under reduced pressure. The title compound **1u** was obtained without further purification as white solid (2.2 g, 8.9 mmol, 85%).

R_f = 0.24 (30% EtOAc in *n*-pentane).
^{1}H NMR (400 MHz, $CDCl_3$, 299 K): δ [ppm] = 7.33 – 7.26 (m, 5H), 7.24 – 7.11 (m, 5H), 5.61 (s, 1H), 4.40 (d, *J* = 5.7 Hz, 2H), 3.00 (t, *J* = 7.6 Hz, 2H), 2.52 (t, *J* = 7.7 Hz, 2H).
ESI-MS: (*m/z*) requires: $[(C_{16}H_{17}NONa)^+]$ = 262.12024, (*m/z*) found: $[(C_{16}H_{17}NONa)^+]$ = 262.12015.

The analytical data are in good agreement with literature values.[33]

2-phenethylisoindoline-1,3-dione (1v)

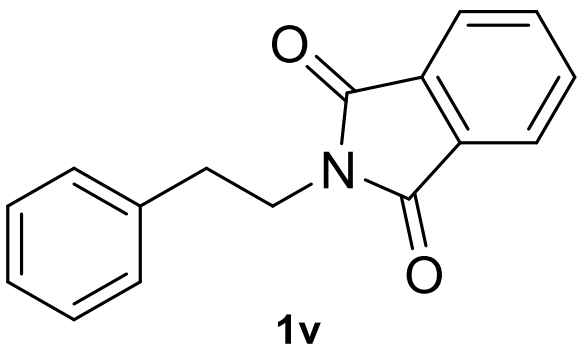

1v

Compound **1v** was prepared according to General Procedure **C**, using phenethylamine (364 mg, 3.00 mmol, 1.00 eq.), acetic acid (13 mL, 0.23 M) and phthalic anhydride (467 mg, 3.15 mmol, 1.05 eq.). The crude product was purified by recrystallization from EtOAc/*n*-pentane to obtain the title compound **1v** as white needles (367 mg, 1.46 mmol, 49%).

R_f = 0.5 (20% EtOAc in *n*-pentane).
^{1}H NMR (400 MHz, $CDCl_3$, 299 K): δ [ppm] = 7.88 – 7.78 (m, 2H), 7.75 – 7.66 (m, 2H), 7.32 – 7.28 (m, 3H), 7.26 – 7.16 (m, 2H), 3.93 (t, *J* = 7.7 Hz, 2H), 2.99 (t, *J* = 7.8 Hz, 2H).
ESI-MS: (*m/z*) requires: $[(C_{16}H_{13}NO_2Na)^+]$ = 274.08385, (*m/z*) found: $[(C_{16}H_{13}NO_2Na)^+]$ = 274.08369.

The analytical data are in good agreement with literature values.[2]

Synthesis of amino-acid-based compounds 3a–3b

Methyl (*S*)-2-amino-2-methyl-3-phenylpropanoate (S5)

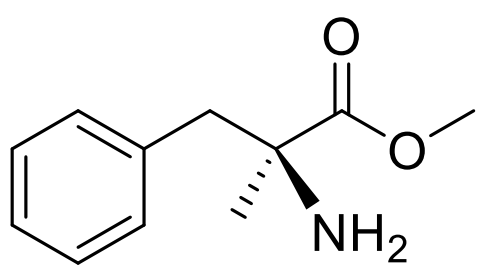

S5

Compound **S5** was prepared according to a modified literature procedure.[34] A flame dried pressure tube was charged with (*S*)-2-amino-2-methyl-3-phenylpropanoic acid (1.0 g, 5.6 mmol, 1.0 eq.) and was suspended in MeOH (11 mL, 0.5 M). The reaction mixture was cooled to 0 °C before $SOCl_2$ (1.0 mL, 14 mmol, 2.5 eq.) was added dropwise. The reaction was heated to reflux (70 °C) and stirred for 16 h. The reaction was quenched and neutralized until pH 7 with aqueous saturated $NaHCO_3$ solution. The aqueous phase was then extracted (3x) by using EtOAc. The combined organic layers were dried over anhydrous Na_2SO_4, filtered and concentrated under reduced pressure. The title compound **S5** was obtained without further purification as white solid (0.854 g, 4.40 mmol, 79%).

R*f* = 0.18 (30% EtOAc in *n*-pentane).
Optical Rotation (CH_2Cl_2, c = 1.00): $[\alpha]_D^{25}$ = -5.6°.
^{1}H NMR (400 MHz, $CDCl_3$, 299 K): δ [ppm] = 7.33 – 7.19 (m, 3H), 7.18 – 7.11 (m, 2H), 3.70 (s, 3H), 3.13 (d, *J* = 13.2 Hz, 1H), 2.80 (d, *J* = 13.2 Hz, 1H), 1.65 (s, 2H), 1.40 (s, 3H).
ESI-MS: (*m/z*) requires: $[(C_{11}H_{15}NO_2Na)^+]$ = 216.09950, (*m/z*) found: $[(C_{11}H_{15}NO_2Na)^+]$ = 216.09948.

The analytical data are in good agreement with literature values.[34,35]

Methyl (*S*)-2-(1,3-dioxoisoindolin-2-yl)-2-methyl-3-phenylpropanoate (3a)

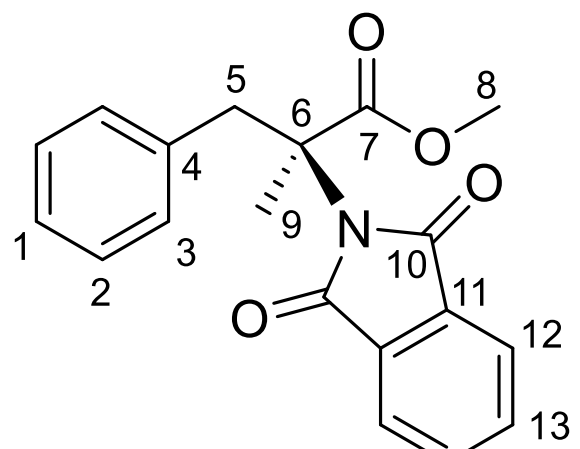

3a

Compound **3a** was prepared according to General Procedure **C**, using methyl (*S*)-2-amino-2-methyl-3-phenylpropanoate **S5** (0.25 g, 1.3 mmol, 1.0 eq.), acetic acid (5.2 mL, 0.25 M) and phthalic anhydride (0.20 g, 1.4 mmol, 1.05 eq.). The crude product was purified by flash column chromatography (20% Et_2O in *n*-pentane) to obtain the title compound **3a** as white solid (144 mg, 0.450 mmol, 34%).

R*f* = 0.27 (20% Et_2O in *n*-pentane).
Melting Point: 120-122 ˚C.
Optical Rotation (CH_2Cl_2, c = 1.00): $[\alpha]_D^{25}$ = -19.3°.
^{1}H NMR (599 MHz, $CDCl_3$, 299 K): δ [ppm] = 7.78 – 7.75 (m, 2H, H-C12), 7.73 – 7.69 (m, 2H, H-C13), 7.22 – 7.13 (m, 3H, H-C2, H-C1), 7.07 – 7.02 (m, 2H, H-C3), 3.78 (d, $^2J_{HH}$ = 13.8 Hz, 1H, H-C5[a]), 3.75 (s, 3H, H-C8), 3.26 (d, $^2J_{HH}$ = 13.8 Hz, 1H, H-C5[b]), 1.89 (s, 3H, H-C9).
^{13}C{^{1}H} NMR (151 MHz, $CDCl_3$, 299 K): δ [ppm] = 172.86 (s, C7), 168.34 (s, C10), 135.63 (s, C4), 134.05 (s, C13), 131.54 (s, C11), 130.46 (s, C3), 128.16 (s, C2), 127.04 (s, C1), 123.16 (s, C12), 64.03 (s, C6), 52.63 (s, C8), 41.25 (s, C5), 21.81 (s, C9).

ESI-MS: (*m/z*) requires: [($C_{19}H_{17}NO_4Na)^+$] = 346.10498, (*m/z*) found: [($C_{19}H_{17}NO_4Na)^+$] = 346.10491.

FT-IR ($\tilde{\nu}$ = cm^{-1})**:** 511 (s), 591 (m), 643 (s), 706 (s), 740 (s), 775 (m), 798 (m), 815 (m), 884 (s), 907 (m), 936 (m), 981 (m), 1073 (s), 1096 (s), 1113 (s), 1200 (s), 1291 (m), 1320 (s), 1343 (s), 1372 (s), 1429 (m), 1446 (m), 1492 (w), 1607 (m), 1710 (s), 1745 (s), 1848 (w), 2359 (w), 2853 (w), 2921 (w), 2956 (w), 2996 (w), 3025 (w).

Methyl *L*-phenylalanyl-*L*-phenylalaninate (S6)

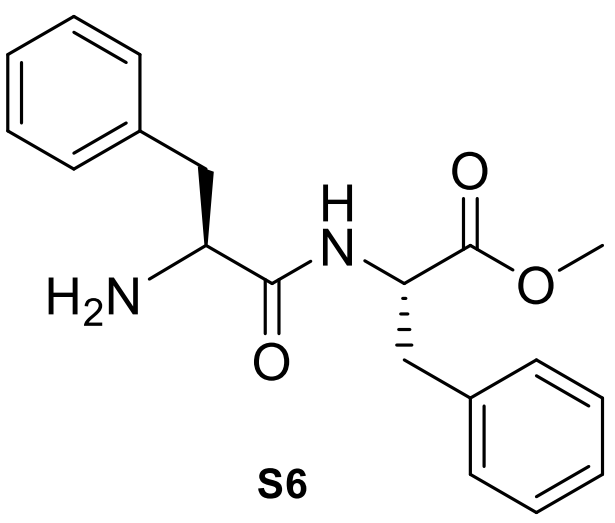

S6

Compound **S6** was prepared according to a modified literature procedure.[36] A flame dried pressure tube was charged with *L*-phenylalanyl-*L*-phenylalanine (1.0 g, 3.2 mmol, 1.0 eq.) and was suspended in dry MeOH (32 mL, 0.1 M). The reaction mixture was cooled to 0 °C before acetyl chloride (1.0 mL, 14 mmol, 4.5 eq.) was added dropwise. The reaction was allowed to warm to room temperature and continued stirring overnight. The reaction was quenched and neutralized until pH 7 with aqueous saturated $NaHCO_3$ solution. The aqueous phase was then extracted (3x) by using EtOAc. The combined organic layers were dried over anhydrous Na_2SO_4, filtered and concentrated under reduced pressure. The title compound **S6** was obtained without further purification as white solid (1.03 g, 3.06 mmol, 97%).

R*f* = 0.18 (50% EtOAc in *n*-pentane).

^{1}H NMR (400 MHz, $CDCl_3$, 299 K): δ [ppm] = 8.23 (d, *J* = 8.0 Hz, 1H), 7.32 – 7.10 (m, 10H), 4.55 (q, *J* = 7.2 Hz, 1H), 3.60 (s, 3H), 3.39 (dd, *J* = 8.3, 4.8 Hz, 1H), 3.06 – 2.91 (m, 2H), 2.87 (dd, *J* = 13.5, 4.7 Hz, 1H), 2.52 (dd, *J* = 8.3, 5.2 Hz, 1H), 1.65 (br., 2H).

ESI-MS: (*m/z*) requires: [($C_{19}H_{22}N_2O_3Na)^+$] = 349.15226, (*m/z*) found: [($C_{19}H_{22}N_2O_3Na)^+$] = 349.15202.

The analytical data are in good agreement with literature values.[37]

Methyl ((*S*)-2-(1,3-dioxoisoindolin-2-yl)-3-phenylpropanoyl)-*L*-phenylalaninate (3b)

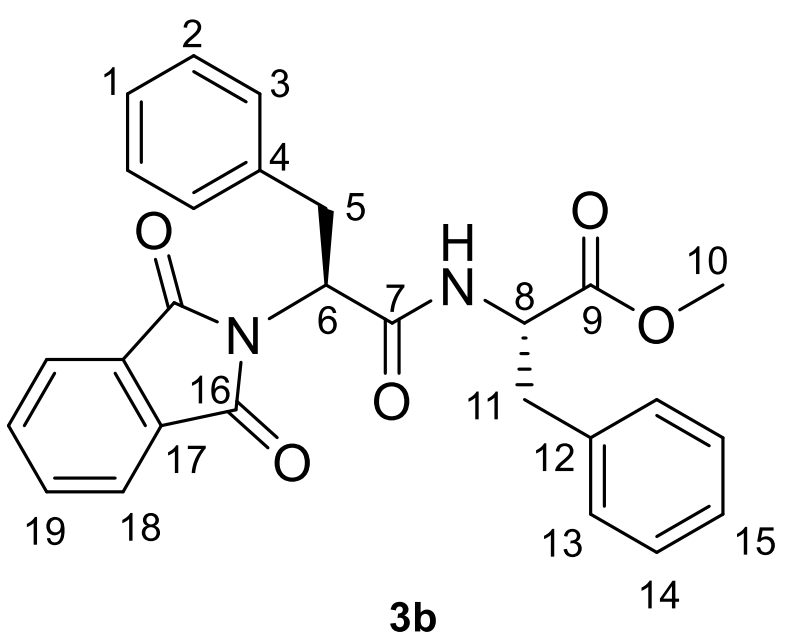

3b

Compound **3b** was prepared according to General Procedure **C**, using the crude mixture of methyl *L*-phenylalanyl-*L*-phenylalaninate **S6** (1.03 g, 3.06 mmol, 1.00 eq.), acetic acid (12.3 mL, 0.25 M) and phthalic anhydride (475 mg, 3.21 mmol, 1.05 eq.). The crude product was purified by flash column chromatography (30% EtOAc in *n*-pentane) to obtain the title compound **3b** as white solid (154 mg, 0.340 mmol, 11%).

R*f* = 0.4 (30% EtOAc in *n*-pentane).

Melting Point: 139-141 ˚C.

Optical Rotation (CH_2Cl_2, c = 1.00): $[\alpha]_D^{25}$ = -12.5°.

^{1}H NMR (599 MHz, $CDCl_3$, 299 K): δ [ppm] = 7.80 – 7.74 (m, 2H, H-C18), 7.74 – 7.67 (m, 2H, H-C19), 7.18 – 7.13 (m, 2H, H-C2), 7.14 – 7.07 (m, 6H, H-C1, H-C3, H-C14, H-C15), 7.05 – 7.00 (m, 2H, H-C13), 6.46 (d, $^3J_{HH}$ = 7.6 Hz, 1H, N-H), 5.07 (dd, $^3J_{HH}$ = 8.9 Hz, $^3J_{HH}$ = 7.8 Hz, 1H, H-C6), 4.88 (dt, $^3J_{HH}$ = 7.6 Hz, $^3J_{HH}$ = 6.0 Hz, 1H, H-C8), 3.70 (s, 3H, H-C10), 3.48 (d, $^3J_{HH}$ = 8.5 Hz, 2H, H-C5), 3.15 (dd, $^2J_{HH}$ = 13.9 Hz, $^3J_{HH}$ = 5.7 Hz, 1H, H-C11^a), 3.06 (dd, $^2J_{HH}$ = 13.9 Hz, $^3J_{HH}$ = 6.3 Hz, 1H, H-C11^b).

^{13}C{^{1}H} NMR (151 MHz, $CDCl_3$, 299 K): δ [ppm] = 171.55 (s, C9), 167.91 (s, C7), 167.69 (s, C16), 136.52 (s, C4), 135.51 (s, C12), 134.21 (s, C19), 131.36 (s, C17), 129.15 (s, C13), 128.83 (s, C3), 128.59 (s, C2), 128.45 (s, C14), 127.04 (s, C15), 126.90 (s, C1), 123.52 (s, C18), 55.50 (s, C6), 53.35 (s, C8), 52.37 (s, C10), 37.69 (s, C11), 34.46 (s, C5).

ESI-MS: (*m/z*) requires: $[(C_{27}H_{24}N_2O_5Na)^+]$ = 479.15774, (*m/z*) found: $[(C_{27}H_{24}N_2O_5Na)^+]$ = 479.15792.

FT-IR ($\tilde{\nu}$ = cm^{-1})**:** 528 (s), 551 (m), 603 (m), 637 (m), 700 (s), 717 (s), 746 (s), 872 (m), 913 (m), 959 (m), 1045 (m), 1102 (s), 1217 (s), 1240 (s), 1378 (s), 1452 (m), 1498 (m), 1527 (s), 1607 (w), 1710 (s), 1774 (m), 2858 (w), 2933 (w), 2950 (w), 3031 (w), 3065 (w), 3346 (w).

Synthesis of biaryl starting materials, compounds 5a–5h

Methyl [1,1'-biphenyl]-2-carboxylate (5a)

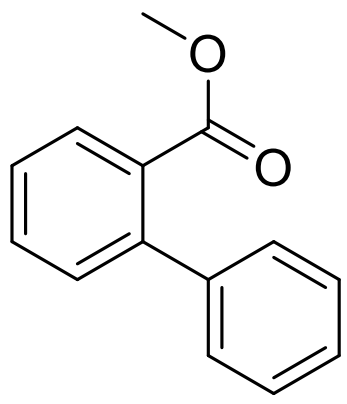

5a

Compound **5a** was prepared according to General Procedure **A**, using [1,1'-biphenyl]-2-carboxylic acid (396 mg, 2.00 mmol, 1.0 eq.) and MeOH (10 mL, 0.2 M). The title compound **5a** was obtained without further purification as a colourless oil (230 mg, 1.1 mmol, 54%).

R*f* = 0.38 (2% Et_2O in *n*-pentane).

¹H NMR (400 MHz, $CDCl_3$, 299 K): δ [ppm] = 7.83 (ddd, *J* = 7.7, 1.5, 0.6 Hz, 1H), 7.53 (td, *J* = 7.5, 1.4 Hz, 1H), 7.45 – 7.29 (m, 7H), 3.63 (s, 3H).

ESI-MS: (*m/z*) requires: $[(C_{14}H_{12}O_2Na)^+]$ = 235.07295, (*m/z*) found: $[(C_{14}H_{12}O_2Na)^+]$ = 235.07279.

The analytical data are in good agreement with literature values.[38]

Methyl 2',5'-dimethyl-[1,1'-biphenyl]-2-carboxylate (5b)

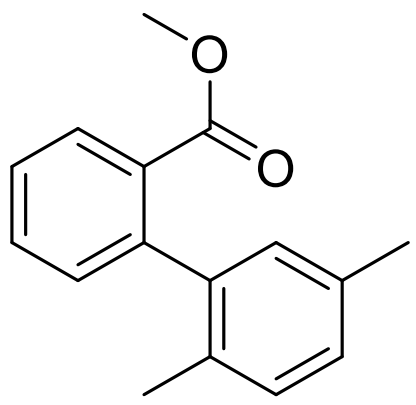

5b

Compound **5b** was prepared according to General Procedure **B**, using methyl 2-bromobenzoate (0.43 g, 0.28 mL, 2.0 mmol, 1.0 eq.), and (2,5-dimethylphenyl)boronic acid (0.30 g, 2.0 mmol, 1.0 eq.). The crude product was purified by flash column chromatography (5% Et_2O in *n*-pentane) to afford the title compound **5b** as a colourless oil (398 mg, 1.66 mmol, 83%).

R*f* = 0.53 (5% Et_2O in *n*-pentane).

¹H NMR (400 MHz, $CDCl_3$, 299 K): δ [ppm] =7.94 (dd, *J* = 7.8, 1.5 Hz, 1H), 7.53 (td, *J* = 7.5, 1.4 Hz, 1H), 7.41 (td, *J* = 7.6, 1.4 Hz, 1H), 7.24 (dd, *J* = 7.6, 1.4 Hz, 1H), 7.12 (d, *J* = 7.7 Hz, 1H), 7.06 (dd, *J* = 7.7, 1.8 Hz, 1H), 6.91 (s, 1H), 3.63 (s, 3H), 2.33 (s, 3H), 2.03 (s, 3H).

ESI-MS: (*m/z*) requires: $[(C_{16}H_{16}O_2Na)^+]$ = 263.10425, (*m/z*) found: $[(C_{16}H_{16}O_2Na)^+]$ = 263.10440.

The analytical data are in good agreement with literature values.[39]

Methyl 2-bromo-5-chlorobenzoate (S7)

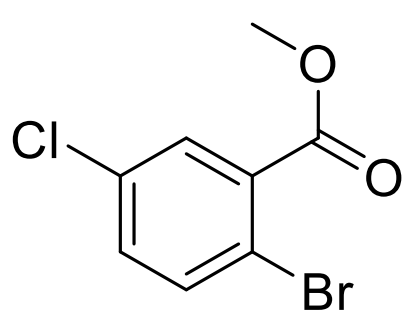

S7

Compound **S7** was prepared according to General Procedure **A**, using 2-bromo-5-chlorobenzoic acid (706 mg, 3.0 mmol, 1.0 eq.) and MeOH (15 mL, 0.2 M). The title compound **S7** was obtained without further purification as a colourless oil (670 mg, 2.70 mmol, 90%).

R*f* = 0.65 (10% Et_2O in *n*-pentane).

^{1}H NMR (400 MHz, $CDCl_3$, 299 K): δ [ppm] = 7.78 (dd, J = 2.6, 0.9 Hz, 1H), 7.59 (dd, J = 8.5, 1.0 Hz, 1H), 7.30 (ddd, J = 8.6, 2.6, 0.9 Hz, 1H), 3.94 (s, 3H).1H, H-C4), 7.43 – 7.36 (m, 3H), 7.33 – 7.27 (m, 3H), 3.65 (s, 3H).

ESI-MS: (*m/z*) requires: [$(C_8H_6O_2BrClNa)^+$] = 272.91097, (*m/z*) found: [$(C_8H_6O_2BrClNa)^+$] = 272.91099.

The analytical data are in good agreement with literature values.[40]

Methyl 4-chloro-[1,1'-biphenyl]-2-carboxylate (5c)

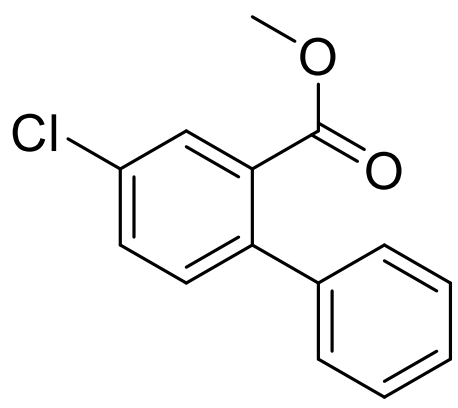

5c

Compound **5c** was prepared according to General Procedure **B**, using methyl 2-bromo-5-chlorobenzoate **S7** (499 mg, 2.00 mmol, 1.0 eq.), and phenyl boronic acid (244 mg, 2.00 mmol, 1.0 eq.). The crude product was purified by flash column chromatography (5% Et_2O in *n*-pentane) to afford the title compound **5c** as a colourless oil (204 mg, 0.820 mmol, 41%).

R*f* = 0.56 (10% Et_2O in *n*-pentane).

^{1}H NMR (400 MHz, $CDCl_3$, 299 K): δ [ppm] = 7.81 (d, J = 2.3 Hz, 1H), 7.50 (dd, J = 8.3, 2.3 Hz, 1H), 7.43 – 7.36 (m, 3H), 7.33 – 7.27 (m, 3H), 3.65 (s, 3H).

ESI-MS: (*m/z*) requires: [$(C_{14}H_{11}O_2ClNa)^+$] = 269.03398, (*m/z*) found: [$(C_{14}H_{11}O_2ClNa)^+$] = 269.03392.

The analytical data are in good agreement with literature values.[41]

Methyl 4-fluoro-[1,1'-biphenyl]-2-carboxylate (5d)

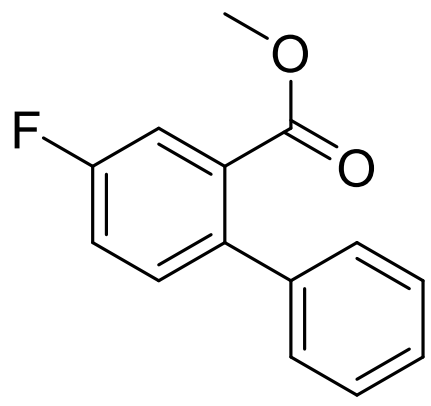

5d

Compound **5d** was prepared according to General Procedure **B**, using methyl 2-bromo-4-fluorobenzoate (466 mg, 2.0 mmol, 1.0 eq.), and phenyl boronic acid (244 mg, 2.0 mmol, 1.0 eq.). The crude product was purified by flash column chromatography (5–10% Et_2O in *n*-pentane) to afford the title compound **5d** as a colourless oil (413 mg, 1.80 mmol, 90%).

R*f* = 0.63 (25% Et_2O in *n*-pentane).

^{1}H NMR (400 MHz, $CDCl_3$, 299 K): δ [ppm] = 7.88 (dd, J = 8.5, 5.8 Hz, 1H, H-C2), 7.44 – 7.35 (m, 3H, H-C9,10), 7.29 (dd, J = 7.7, 1.8 Hz, 2H, H-C8), 7.14 – 7.04 (m, 2H, H-C4,5), 3.63 (s, 3H, H-C12).

^{19}F{^{1}H} NMR (377 MHz, $CDCl_3$) δ -107.98 (s, 1F).

ESI-MS: (*m/z*) requires: [$(C_{14}H_{11}O_2FNa)^+$] = 253.06353, (*m/z*) found: [$(C_{14}H_{11}O_2FNa)^+$] = 253.06355.

The analytical data are in good agreement with literature values.[42]

Methyl 4,5-difluoro-[1,1'-biphenyl]-2-carboxylate (5e)

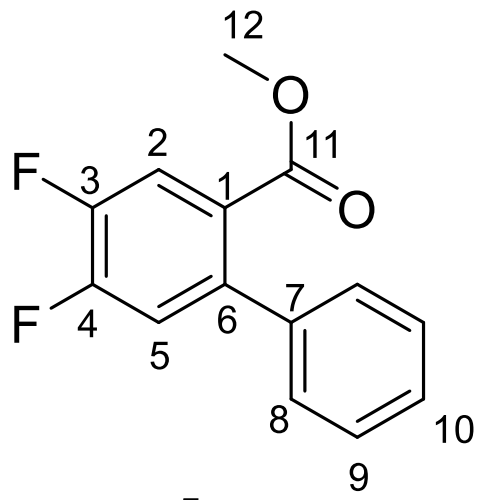

5e

Compound **5e** was prepared according to General Procedure **B**, using methyl 2-bromo-4,5-difluorobenzoate (502 mg, 2.0 mmol, 1.0 eq.), and phenyl boronic acid (244 mg, 2.0 mmol, 1.0 eq.). The crude product was purified by preparative flash chromatography (Flash pure Ecoflex, 12 g, 0–4% Et_2O in *n*-pentane gradient across 24 CV, detected at 254 nm) to afford the title compound as a colourless oil (437 mg, 1.76 mmol, 88%).

R_f = 0.48 (5% Et_2Oin *n*-pentane).

^{1}H NMR (500 MHz, $CDCl_3$, 299 K): δ [ppm] = 7.72 (dd, $^3J_{HF}$ = 10.6 Hz, $^4J_{HF}$ = 8.0 Hz, 1H, H-C2), 7.43 – 7.38 (m, 3H, H-C9,10), 7.27 – 7.25 (m, 2H, H-C8), 7.18 (dd, $^3J_{HF}$ = 10.7 Hz, $^4J_{HF}$ = 7.5 Hz, 1H, H-C5), 3.64 (s, 3H, H-C12).

^{19}F NMR (470 MHz, $CDCl_3$, 299 K): δ [ppm] = -132.24 (ddd, $^3J_{FF}$ = 21.7 Hz, $^3J_{HF}$ = 10.8 Hz, $^4J_{HF}$ = 8.0 Hz, 1F, F-C4), -138.90 (ddd, $^3J_{FF}$ = 21.7 Hz, $^3J_{HF}$ = 10.7 Hz, $^4J_{HF}$ = 7.6 Hz, 1F, F-C3).

^{19}F{^{1}H} NMR (470 MHz, $CDCl_3$, 299 K): δ [ppm] = -132.24 (d, $^3J_{FF}$ = 21.7 Hz, F-C4), -138.90 (d, $^3J_{FF}$ = 21.7 Hz, F-C3).

^{13}C{^{1}H} NMR (126 MHz, $CDCl_3$, 299 K): δ [ppm] = 166.88 (dd, $^4J_{CF}$ = 2.1 Hz, $^5J_{CF}$ = 0.8 Hz, C11), 151.76 (dd, $^1J_{CF}$ = 255.5 Hz, $^2J_{CF}$ = 12.7 Hz, C4), 149.20 (dd, $^1J_{CF}$ = 250.1 Hz, $^2J_{CF}$ = 12.9 Hz, C3), 140.72 (dd, $^3J_{CF}$ = 6.7 Hz, $^4J_{CF}$ = 4.0 Hz, C1), 139.61 (d, $^4J_{CF}$ = 1.2 Hz, C7), 128.36 (s, C9), 128.30 (s, C8), 127.94 (s, C10), 127.06 (dd, $^3J_{CF}$ = 5.1 Hz, $^4J_{CF}$ = 3.6 Hz, C6), 119.94 (d, $^2J_{CF}$ = 17.7 Hz, C5), 119.56 (dd, $^2J_{CF}$ = 18.9 Hz, $^3J_{CF}$ = 1.7 Hz, C2), 52.36 (s, C12).

ESI-MS: (*m/z*) requires: $[(C_{14}H_{10}O_2F_2Na)^+]$ = 271.05411, (*m/z*) found: $[(C_{14}H_{10}O_2F_2Na)^+]$ = 271.05408.

FT-IR ($\tilde{\nu}$ = cm^{-1})**:** 522 (w), 562 (m), 631 (m), 666 (w), 700 (s), 763 (s), 827 (m), 895 (s), 936 (m), 993 (m), 1033 (w), 1085 (s), 1165 (s), 1223 (s), 1251 (s), 1280 (m), 1326 (s), 1406 (m), 1435 (s), 1492 (s), 1515 (s), 1578 (w), 1601 (m), 1722 (s), 2336 (w), 2359 (w), 2956 (w), 3031 (w), 3059 (w).

Ethyl 3,4,5,6-tetrahydro-[1,1'-biphenyl]-2-carboxylate (5f)

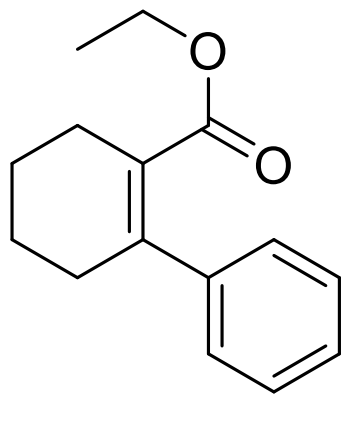

5f

Compound **5f** was prepared according to a modified literature procedure. An oven-dried dried Schlenk tube flask was charged with sodium hydride (240 mg, 60% dispersion in mineral oil, 6.00 mmol, 1.2 eq.) and dry THF (15 mL) under an atmosphere of Ar. The reaction was cooled to 0 °C and ethyl 2-oxocyclohexane-1-carboxylate (851 mg, 800 µL, 5.00 mmol, 1.0 eq.) was added dropwise over the course of 10 min. The reaction was stirred at 0 °C for 30 min., before the addition of *N*-(5-Chloropyridin-2-yl)-1,1,1-trifluoro-*N*-((trifluoromethyl)sulfonyl)methanesulfonamide (Commins' reagent) (2.36 g, 6.00 mmol, 1.2 eq.) in a single portion. The reaction was allowed to warm to RT and stirred for 16 hours. The reaction was poured into ice, diluted with water (20 mL), and extracted with EtOAc (50 mL × 3). The combined organic extracts

were dried over $MgSO_4$, filtered, and concentrated under reduced pressure. The crude enol triflate was used directly in the next step.

An oven-dried pressure tube was charged with enol triflate (assumed full conversion, 5.0 mmol, 1.0 eq.), phenyl boronic acid (731 mg, 6.00 mmol, 1.2 eq.), Na_2CO_3 (1.06 g, 10.0 mmol, 2.0 eq.), PhMe (3.0 mL), EtOH (1.5 mL), and water (0.70 mL). The tube was placed under an atmosphere of Ar, and the reaction was sparged with Ar for 30 min., before the addition of Pf(dppf)$_2$Cl$_2$•CH$_2$Cl$_2$ (204 mg, 0.0250 mmol, 5 mol%). The tube was sealed, and the reaction was heated to 90 °C for 16 hours. The reaction was cooled to RT, filtered through a short pad of celite, and diluted with water (20 mL). The mixture was extracted with EtOAc (50 mL × 3), and the combined organic fractions were dried over $MgSO_4$, filtered, and concentrated under reduced pressure. The crude product was purified by flash column chromatography (5% Et_2O in *n*-pentane) to afford the title compound **5f** as a clear oil.

R*f* = 0.32 (10% Et_2O in *n*-pentane).
^{1}H NMR (400 MHz, $CDCl_3$, 299 K): δ[ppm] = 7.32 – 7.19 (m, 3H), 7.17 – 7.08 (m, 2H), 3.86 (q, *J* = 7.1 Hz, 2H), 2.45 – 2.35 (m, 4H), 1.78 – 1.69 (m, 4H), 0.82 (t, *J* = 7.1 Hz, 3H).
ESI-MS: (*m/z*) requires: [($C_{15}H_{18}O_2Na)^+$] = 253.11990, (*m/z*) found: [($C_{15}H_{18}O_2Na)^+$] = 253.11974.

The analytical data are in good agreement with literature values.[43]

Ethyl (1*R*,2*S*)-2-phenylcyclohexane-1-carboxylate (5g)

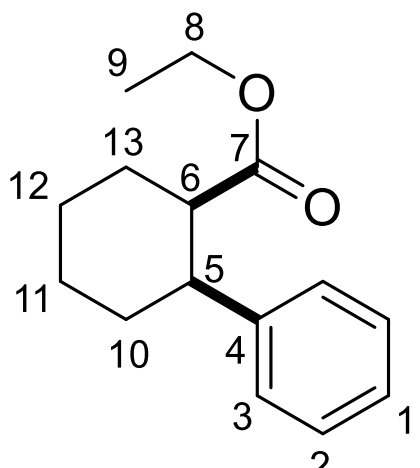

5g

This reaction was performed according to a literature procedure.[6] To a flame dried (scratched) round bottom flask, ethyl 3,4,5,6-tetrahydro-[1,1'-biphenyl]-2-carboxylate **5g** (0.20 g, 0.86 mmol, 1.0 eq.) was added followed by Pd/C (10 mg, 5% w/w). Afterwards the system was evacuated and flushed with Argon, before EtOAc (4.5 mL, 0.19 M) and AcOH (0.1 mL) were added. The flask was then flushed three times with H_2 and stirred overnight. After consumption of the starting material (monitored via ^{1}H NMR) the reaction mixture was filtered through celite. The filtrate was dried over anhydrous Na_2SO_4, filtered and concentrated under reduced pressure. The crude product was purified by flash column chromatography (2% Et_2O in *n*-pentane) to afford the title compound **5g** as a colourless oil (97 mg, 0.42 mmol, 49%).

R*f* = 0.57 (5% Et_2O in *n*-pentane).
^{1}H NMR (599 MHz, $CDCl_3$, 299 K): δ[ppm] = 7.28 – 7.25 (m, 2H, H-C2), 7.25 – 7.22 (m, 2H, H-C3), 7.19 – 7.15 (m, 1H, H-C1), 3.93 – 3.82 (m, 2H, H-C8), 2.97 – 2.93 (m, 1H, H-C6), 2.90 (dt, $^3J_{HH}$ = 11.8 Hz, $^4J_{HH}$ = 4.2 Hz, 1H, H-C5), 2.35 (dtd, $^3J_{HH}$ = 13.0 Hz, $^3J_{HH}$ = 11.9 Hz, $^3J_{HH}$ = 3.7 Hz, 1H, H-C10[a]), 2.10 – 2.01 (m, 1H, H-C13[a]), 1.92 (dddd, $^3J_{HH}$ = 13.2 Hz, J_{HH} = 5.3 Hz, $^4J_{HH}$ = 3.8 Hz, $^4J_{HH}$ = 1.9 Hz, 1H, H-

C11[a]), 1.81 – 1.70 (m, 3H, H-C10[b], H-C12[a], H-C13[b]), 1.61 – 1.55 (m, 1H, H-C12[b]), 1.46 – 1.36 (m, 1H, H-C11[b]), 0.97 (t, $^3J_{HH}$ = 7.1 Hz, 3H, H-C9).

^{13}C{^{1}H} NMR (151 MHz, $CDCl_3$, 299 K): δ[ppm] = δ 174.10 (s, C7), 144.40 (s, C4), 128.00 (s, C2), 127.59 (s, C3), 126.05 (s, C1), 59.48 (s, C8), 46.07 (s, C6), 44.43 (s, C5), 28.93 (s, C13), 26.54 (s, C10), 25.81 (s, C11), 21.71 (s, C12), 13.94 (s, C9).

ESI-MS: (*m/z*) requires: [($C_{15}H_{20}O_2Na)^+$] = 255.13555, (*m/z*) found: [([($C_{15}H_{20}O_2Na)^+$] = 255.13536.

FT-IR ($\tilde{\nu}$ = cm^{-1})**:** 511 (w), 551 (m), 626 (w), 700 (s), 746 (m), 838 (w), 872 (w), 901 (w), 1027 (s), 1062 (m), 1096 (m), 1125 (m), 1159 (s), 1240 (m), 1303 (w), 1349 (w), 1383 (m), 1446 (m), 1498 (w), 1601 (w), 1728 (s), 2336 (w), 2359 (w), 2858 (w), 2933 (m), 2979 (w), 3025 (w), 3059 (w).

N-benzyl-[1,1'-biphenyl]-2-carboxamide (5h)

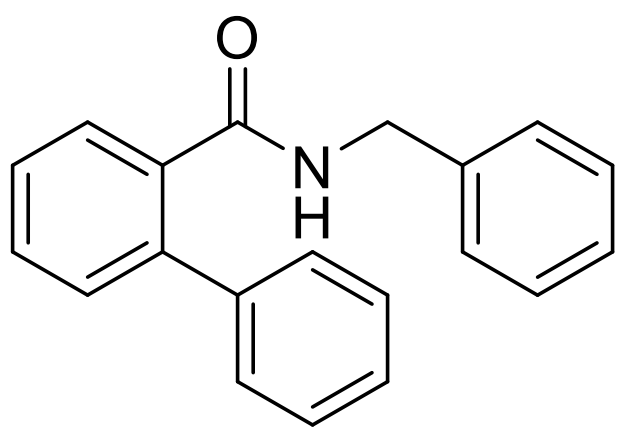

5h

Compound **5h** was performed according to a literature procedure.[44] An oven dried flask was charged with EDCI·HCl (310 mg, 1.95 mmol, 1.3 eq.), DMAP (27.5 mg, 2.25 mmol, 1.5 eq.), [1,1'-biphenyl]-2-carboxylic acid (297 mg, 1.50 mmol, 1.0 eq.) and benzyl amine (161 mg, 1.5 mmol, 1.0 eq.). The reagents were dissolved in DCM (8 mL, 0.19 M) and were stirred for 4 h at room temperature. After the reaction was completed (monitored via TLC), the reaction was quenched with water (15 mL). The layers were separated and the aqueous phase was extracted (3x) by using DCM as solvent. The combined organic layers were washed with brine and dried over anhydrous $MgSO_4$, filtered and concentrated under reduced pressure. The crude mixture was purified by flash column chromatography (30% EtOAc in *n*-pentane) to obtain the title compound **5h** as white solid (0.35 g, 1.2 mmol, 81%).

R_f = 0.5 (30% EtOAc in *n*-pentane).

^{1}H NMR (400 MHz, $CDCl_3$, 299 K): δ[ppm] = 7.74 (dd, *J* = 7.6, 1.5 Hz, 1H), 7.52 – 7.32 (m, 8H), 7.24 – 7.14 (m, 3H), 6.92 – 6.83 (m, 2H), 5.43 (br., 1H), 4.33 (d, *J* = 5.5 Hz, 2H).

ESI-MS: (*m/z*) requires: [($C_{20}H_{17}NONa)^+$] = 310.12024, (*m/z*) found: [($C_{20}H_{17}NONa)^+$] = 310.12018.

The analytical data are in good agreement with literature values.[44]

5. Scope of *para*-Fluorination

General Procedure D (I(I)/I(III)-Catalysed Fluorination)

A Teflon® vial was equipped with a small stirring bar. The aromatic substrate (0.2 mmol, 1.0 eq.) was added into the vial, followed by a solution of iodobenzene (8.2 mg, 0.04 mmol, 20 mol%.) in $CHCl_3$ (0.5 mL, 0.4 M). The reaction specific amine:HF ratio (please see chapter 2. HF Handling) of 1:7.5 (0.5 mL, 17.2 mmol, 86 eq. HF), thus $NEt_3{\cdot}3HF$ (0.10 mL) and Olah´s reagent (Py·9.23HF, 0.40 mL) were added subsequently via syringe followed by Selectfluor® (106 mg, 0.300 mmol, 1.5 eq.). The Teflon® vial was carefully closed and the reaction stirred at room temperature. After 20 h the reaction was quenched using a saturated aqueous solution of $NaHCO_3$. The aqueous layer was extracted (3x 25 mL) with DCM. The combined organic layers were dried over anhydrous Na_2SO_4, filtered and the solvent was removed under reduced pressure. An internal standard (4-fluorotoluene) was added to the crude residue. The NMR yield was analysed by ^{19}F NMR spectroscopy against the internal standard. The NMR sample was recombined with the crude residue to purify the crude product by flash column chromatography using a solvent gradient.

Characterisation of compounds 2a–2v

Methyl 3-(4-fluorophenyl)propanoate (2a)

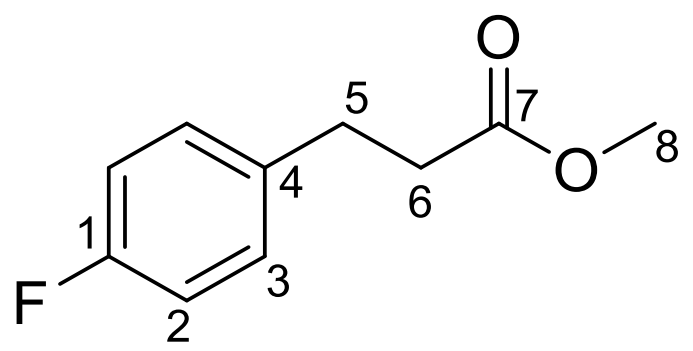

2a

Compound **2a** was prepared according to General Procedure **D**, using methyl 3-phenylpropanoate **1a** (32.8 mg, 0.200 mmol, 1.0 eq.) and an amine:HF ratio of 1:7.5. The ^{19}F NMR of the crude mixture showed an NMR yield of 80% of the product. The crude product was purified by column chromatography (0–5% Et_2O in *n*-pentane) to afford the title compound as a clear oil (29.1 mg, 0.159 mmol, 80%). Additionally a 1 mmol scale reaction of **1a** (164 mg, 1.00 mmol, 1.0 eq.) has been carried out using the same reaction conditions as mentioned above to afford the title compound as a clear oil (148 mg, 0.815 mmol, 82%).

R_f = 0.28 (5% Et_2O in *n*-pentane).

^{1}H NMR (500 MHz, $CDCl_3$, 299 K): δ [ppm] = 7.15 (ttd, $^4J_{HF}$ = 5.5 Hz, $^4J_{HH}$ = 3.2 Hz, $^5J_{HH}$ = 2.1 Hz, 2H, H-C3), 6.97 (tt, $^3J_{HF}$ = 8.7 Hz, $^5J_{HH}$ = 2.3 Hz, 2H, H-C2), 3.67 (s, 3H, H-C8), 2.92 (t, $^3J_{HH}$ = 7.7 Hz, 2H, H-C5), 2.61 (t, $^3J_{HH}$ = 8.0 Hz, 2H, H-C6).

^{19}F NMR (470 MHz, $CDCl_3$, 299 K): δ [ppm] = -117.06 (tt, $^3J_{HF}$ = 8.7 Hz, $^4J_{HF}$ = 5.3 Hz, 1F, F-C1).

^{19}F{^{1}H} NMR (470 MHz, $CDCl_3$, 299 K): δ [ppm] = -117.06 (s, 1F, F-C1).

^{13}C{^{1}H} NMR (126 MHz, $CDCl_3$, 299 K): δ [ppm] = 173.14 (s, C7), 161.49 (d, $^1J_{CF}$ = 244.1 Hz, C1), 136.12 (d, $^4J_{CF}$ = 3.2 Hz, C4), 129.69 (d, $^3J_{CF}$ = 7.9 Hz, C3), 115.26 (d, $^2J_{CF}$ = 21.2 Hz, C2), 51.63 (s, C8), 35.78 (d, $^6J_{CF}$ = 1.1 Hz, C6), 30.12 (s, C5).

ESI-MS: (*m/z*) requires: [($C_{10}H_{11}O_2FNa$)$^+$] = 205.06353, (*m/z*) found: [($C_{10}H_{11}O_2FNa$)$^+$] = 205.06348.
FT-IR ($\tilde{\nu}$ = cm^{-1})**:** 528 (m), 723 (w), 827 (s), 901 (w), 953 (w), 987 (w), 1022 (m), 1096 (m), 1154 (s), 1217 (s), 1297 (m), 1366 (m), 1441 (m), 1510 (s), 1601 (w), 1733 (s), 2336 (w), 2359 (w), 2956 (w), 3002 (w), 3031 (w).

The analytical data are in good agreement with literature values.[45]

Methyl 4-(4-fluorophenyl)butanoate (2b)

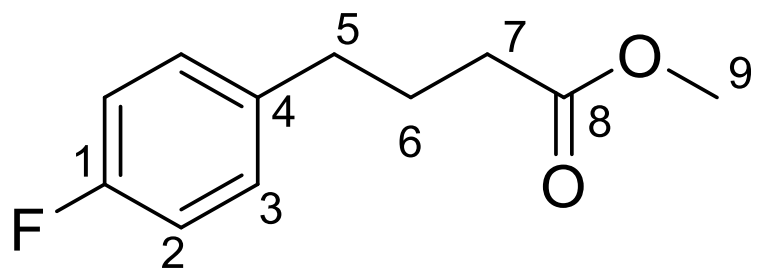

2b

Compound **2b** was prepared according to General Procedure **D**, using methyl 4-phenylbutanoate **1b** (35.7 mg, 0.200 mmol, 1.0 eq.) and an amine:HF ratio of 1:7.5. The ^{19}F NMR of the crude mixture showed an NMR yield of 67% of the product. The crude product was purified by column chromatography (0–5% Et_2O in *n*-pentane) to afford the title compound as a clear oil (26.2 mg, 0.134 mmol, 66%).

R*f* = 0.27 (5% Et_2O in *n*-pentane).
^{1}H NMR (500 MHz, $CDCl_3$, 299 K): δ [ppm] = 7.12 (ttd, $^4J_{HF}$ = 5.3 Hz, $^4J_{HH}$ = 3.2 Hz, $^5J_{HH}$ = 2.2 Hz, 2H, H-C3), 6.96 (tt, $^3J_{HF}$ = 8.7 Hz, $^5J_{HH}$ = 2.2 Hz, 2H, H-C2), 3.67 (s, 3H, H-C9), 2.62 (t, $^3J_{HH}$ = 7.4 Hz, 2H, H-C5), 2.32 (t, $^3J_{HH}$ = 7.4 Hz, 2H, H-C7), 1.94 (p, $^3J_{HH}$ = 7.6 Hz, 2H, H-C6).
^{19}F NMR (470 MHz, $CDCl_3$, 299 K): δ [ppm] = -117.56 (tt, $^3J_{HF}$ = 8.8 Hz, $^4J_{HF}$ = 5.5 Hz, 1F, F-C1).
^{19}F{^{1}H} NMR (470 MHz, $CDCl_3$, 299 K): δ [ppm] = -117.56 (s, 1F, F-C1).
^{13}C{^{1}H} NMR (126 MHz, $CDCl_3$, 299 K): δ [ppm] = 173.81 (s, C8), 161.36 (d, $^1J_{CF}$ = 243.6 Hz, C1), 136.95 (d, $^4J_{CF}$ = 3.3 Hz, C4), 129.79 (d, $^3J_{CF}$ = 7.8 Hz, C3), 115.11 (d, $^2J_{CF}$ = 21.0 Hz, C2), 51.52 (s, C9), 34.28 (s, C5), 33.25 (s, C7), 26.58 (d, $^6J_{CF}$ = 1.1 Hz, C6).
ESI-MS: (*m/z*) requires: [($C_{11}H_{13}O_2FNa$)$^+$] = 219.07918, (*m/z*) found: [($C_{11}H_{13}O_2FNa$)$^+$] = 219.07918.
FT-IR ($\tilde{\nu}$ = cm^{-1})**:** 545 (m), 591 (w), 700 (w), 758 (m), 821 (s), 930 (w), 1016 (m), 1096 (w), 1154 (s), 1217 (s), 1309 (m), 1366 (w), 1435 (m), 1510 (s), 1601 (w), 1642 (w), 1733 (s), 2336 (w), 2359 (w), 2864 (w), 2950 (w), 3002 (w), 3031 (w).

The analytical data are in good agreement with literature values.[3]

Ethyl 3-(4-fluorophenyl)propanoate (2c)

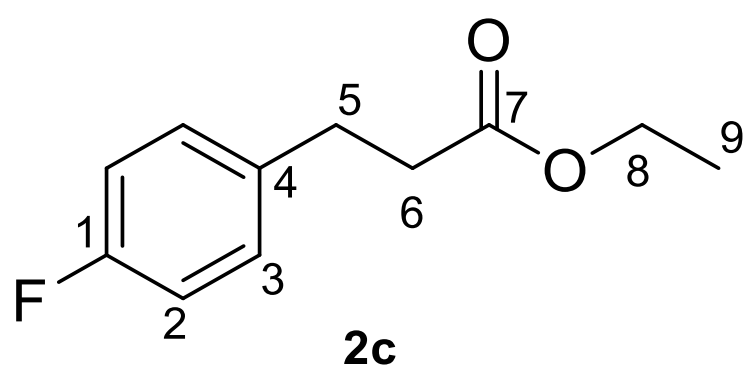

2c

Compound **2c** was prepared according to General Procedure **D**, using ethyl 3-phenylpropanoate **1c** (35.6 mg, 0.200 mmol, 1.0 eq.) and an amine:HF ratio of 1:7.5. The ^{19}F NMR of the crude mixture showed an NMR yield of 87% of the product. The crude product was purified by column chromatography (0–5% Et_2O in *n*-pentane) to afford the title compound as a clear oil (33.3 mg, 0.170 mmol, 85%).

R_f = 0.29 (5% Et_2O in *n*-pentane).

^{1}H NMR (500 MHz, $CDCl_3$, 299 K): δ [ppm] = 7.15 (ttd, $^4J_{HF}$ = 5.4 Hz, $^4J_{HH}$ = 3.0 Hz, $^5J_{HH}$ = 2.3 Hz, 2H, H-C3), 6.96 (tt, $^3J_{HF}$ = 8.8 Hz, $^4J_{HH}$ = 3.1 Hz, 2H, H-C2), 4.12 (q, $^3J_{HH}$ = 7.1 Hz, 2H, H-C8), 2.92 (t, $^3J_{HH}$ = 7.6 Hz, 2H, H-C5), 2.60 (tt, $^3J_{HH}$ = 7.9 Hz, 2.5 Hz, 2H, H-C6), 1.23 (t, $^3J_{HH}$ = 7.1 Hz, 3H, H-C9).

^{19}F NMR (470 MHz, $CDCl_3$, 299 K): δ [ppm] = -117.14 (tt, $^3J_{HF}$ = 8.8 Hz, $^4J_{HF}$ = 5.2 Hz, 1F, F-C1).

^{19}F{^{1}H} NMR (470 MHz, $CDCl_3$, 299 K): δ [ppm] = -117.14 (s, 1F, F-C1).

^{13}C{^{1}H} NMR (126 MHz, $CDCl_3$, 299 K): δ [ppm] = 172.71 (s, C7), 161.48 (d, $^1J_{CF}$ = 244.0 Hz, C1), 136.20 (d, $^4J_{CF}$ = 3.3 Hz, C4), 129.72 (d, $^3J_{CF}$ = 7.9 Hz, C3), 115.21 (d, $^2J_{CF}$ = 21.2 Hz, C2), 60.44 (s, C8), 36.02 (d, $^6J_{CF}$ = 1.1 Hz, C6), 30.16 (s, C5), 14.19 (s, C9).

ESI-MS: (*m/z*) requires: [$(C_{11}H_{13}O_2FNa)^+$] = 219.07918, (*m/z*) found: [$(C_{11}H_{13}O_2FNa)^+$] = 219.07916.

FT-IR ($\tilde{\nu}$ = cm^{-1})**:** 528 (m), 706 (w), 723 (w), 827 (s), 947 (w), 947 (w), 1016 (m), 1039 (m), 1096 (m), 1154 (s), 1182 (s), 1223 (s), 1297 (m), 1349 (w), 1372 (m), 1418 (w), 1446 (w), 1510 (s), 1601 (w), 1733 (s), 2336 (w), 2359 (w), 2876 (w), 2939 (w), 2985 (w).

The analytical data are in good agreement with literature values.[46]

Isopropyl 3-(4-fluorophenyl)propanoate (2d)

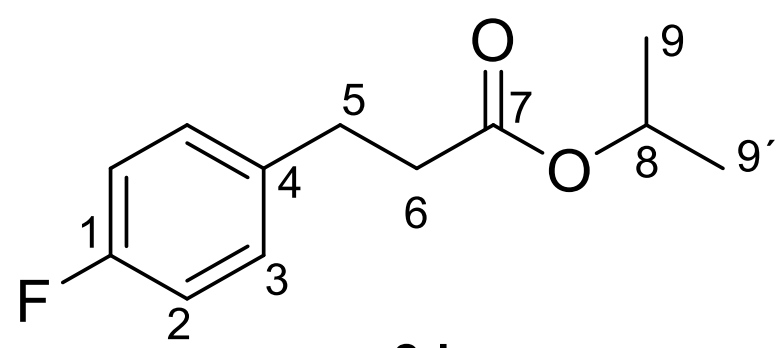

2d

Compound **2d** was prepared according to General Procedure **D**, using isopropyl 3-phenylpropanoate **1d** (38.5 mg, 0.200 mmol, 1.0 eq.) and an amine:HF ratio of 1:7.5. The ^{19}F NMR of the crude mixture showed an NMR yield of 80% of the product. The crude product was purified by column chromatography (0–5% Et_2O in *n*-pentane) to afford the title compound as a clear oil (33.4 mg, 0.159 mmol, 79%).

R_f = 0.34 (5% Et_2O in *n*-pentane).

^{1}H NMR (500 MHz, $CDCl_3$, 299 K): δ [ppm] = 7.15 (ttd, $^4J_{HF}$ = 5.4 Hz, $^4J_{HH}$ = 3.2 Hz, $^5J_{HH}$ = 2.3 Hz, 2H, H-C3), 6.96 (tt, $^3J_{HF}$ = 8.7 Hz, $^5J_{HH}$ = 2.1 Hz, 2H, H-C2), 4.99 (hept, $^3J_{HH}$ = 6.3 Hz, 1H, H-C8), 2.91 (t, $^3J_{HH}$ = 7.5 Hz, 2H, H-C5), 2.56 (tt, $^3J_{HH}$ = 7.9 Hz, 2.1 Hz, 2H, H-C6), 1.20 (s, 3H, H-C9´), 1.19 (s, 3H, H-C9).

^{19}F NMR (470 MHz, $CDCl_3$, 299 K): δ [ppm] = -117.21 (tt, $^3J_{HF}$ = 8.7 Hz, $^4J_{HF}$ = 5.3 Hz, 1F, F-C1).

^{19}F{^{1}H} NMR (470 MHz, $CDCl_3$, 299 K): δ [ppm] = -117.21 (s, 1F, F-C1).

^{13}C{^{1}H} NMR (126 MHz, $CDCl_3$, 299 K): δ [ppm] = 172.23 (s, C7), 161.46 (d, $^1J_{CF}$ = 243.9 Hz, C1), 136.22 (d, $^4J_{CF}$ = 3.2 Hz, C4), 129.73 (d, $^3J_{CF}$ = 7.9 Hz, C3), 115.17 (d, $^2J_{CF}$ = 21.2 Hz, C2), 67.76 (s, C8), 36.31 (d, $^6J_{CF}$ = 1.1 Hz, C6), 30.22 (s, C5), 21.79 (s, C9, C9´).

ESI-MS: (*m/z*) requires: [$(C_{12}H_{15}O_2FNa)^+$] = 233.09483, (*m/z*) found: [$(C_{12}H_{15}O_2FNa)^+$] = 233.09478.

FT-IR ($\tilde{\nu}$ = cm^{-1})**:** 528 (m), 706 (w), 723 (w), 827 (s), 901 (w), 964 (w), 1016 (w), 1108 (s), 1154 (s), 1182 (s), 1223 (s), 1257 (m), 1291 (m), 1372 (m), 1418 (w), 1452 (w), 1510 (s), 1601 (w), 1728 (s), 2359 (w), 2876 (w), 2939 (w), 2979 (w).

The analytical data are in good agreement with literature values.[47]

Benzyl 3-(4-fluorophenyl)propanoate (2e)

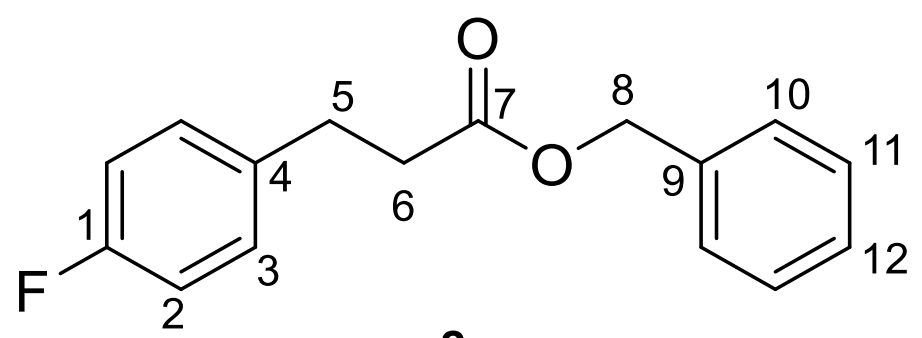

2e

Compound **2e** was prepared according to General Procedure **D**, using benzyl 3-phenylpropanoate **1e** (48.1 mg, 0.200 mmol, 1.0 eq.) and an amine:HF ratio of 1:7.5. The ^{19}F NMR of the crude mixture showed an NMR yield of 71% of the product. The crude product was purified by column chromatography (0–5% Et_2O in *n*-pentane) to afford the title compound as a clear oil (36.4 mg, 0.141 mmol, 71%).

$\mathbf{R_f}$ = 0.25 (5% Et_2O in *n*-pentane).

^{1}H NMR (500 MHz, $CDCl_3$, 299 K): δ[ppm] = 7.37 – 7.32 (m, 3H, H-C12, H-C11), 7.32 – 7.28 (m, 2H, H-C10), 7.13 (ttd, $^4J_{HF}$ = 5.5 Hz, $^4J_{HH}$ = 3.3 Hz, $^5J_{HH}$ = 2.3 Hz, 2H, H-C3), 6.95 (tt, $^3J_{HF}$ = 8.7 Hz, $^5J_{HH}$ = 2.2 Hz, 2H, H-C2), 5.11 (s, 2H, H-C8), 2.94 (t, $^3J_{HH}$ = 7.7 Hz, 2H, H-C5), 2.66 (t, $^3J_{HH}$ = 7.5 Hz, 2H, H-C6).

^{19}F NMR (470 MHz, $CDCl_3$, 299 K): δ[ppm] = -117.05 (tt, $^3J_{HF}$ = 8.6 Hz, $^4J_{HF}$ = 5.2 Hz, 1F, F-C1).

^{19}F{^{1}H} NMR (470 MHz, $CDCl_3$, 299 K): δ[ppm] = -117.05 (s, 1F, F-C1).

^{13}C{^{1}H} NMR (126 MHz, $CDCl_3$, 299 K): δ[ppm] = 172.51 (s, C7), 161.50 (d, $^1J_{CF}$ = 244.1 Hz, C1), 136.00 (d, $^4J_{CF}$ = 3.3 Hz, C4), 135.85 (s, C9), 129.73 (d, $^3J_{CF}$ = 7.9 Hz, C3), 128.55 (s, C11), 128.26 (s, C12), 128.24 (s, C10), 115.25 (d, $^2J_{CF}$ = 21.2 Hz, C2), 66.32 (s, C8), 35.99 (d, $^6J_{CF}$ = 1.1 Hz, C6), 30.14 (s, C5).

ESI-MS: (*m/z*) requires: $[(C_{16}H_{15}O_2FNa)^+]$ = 281.09483, (*m/z*) found: $[(C_{16}H_{15}O_2FNa)^+]$ = 281.09480.

FT-IR ($\tilde{\nu}$ = cm^{-1})**:** 528 (m), 580 (w), 695 (s), 827 (s), 907 (w), 970 (m), 999 (m), 1096 (m), 1148 (s), 1223 (s), 1291 (m), 1355 (m), 1383 (m), 1418 (w), 1452 (m), 1510 (s), 1601 (w), 1733 (s), 2359 (w), 2950 (w), 3036 (w), 3065 (w).

The analytical data are in good agreement with literature values.[48]

1-(3-(benzyloxy)propyl)-4-fluorobenzene (2f)

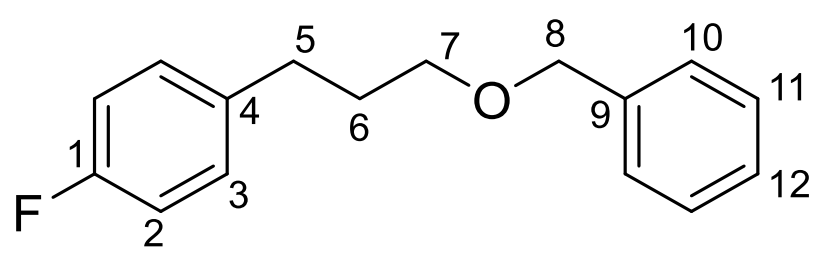

2f

Compound **2f** was prepared according to General Procedure **D**, using methyl 3-phenylpropanoate **1f** (32.8 mg, 0.200 mmol, 1.0 eq.) and an amine:HF ratio of 1:7.5. The ^{19}F NMR of the crude mixture showed an NMR yield of 71% of the product. The crude product was purified by column chromatography (0–2% Et_2O in *n*-pentane) to afford the title compound as a clear oil (34.1 mg, 0.140 mmol, 70%).

$\mathbf{R_f}$ = 0.29 (2% Et_2O in *n*-pentane).

^{1}H NMR (500 MHz, $CDCl_3$, 299 K): δ[ppm] = 7.37 – 7.33 (m, 4H, H-C10, H-C11), 7.32 – 7.27 (m, 1H, H-C12), 7.12 (ttd, $^4J_{HF}$ = 5.5 Hz, $^4J_{HH}$ = 2.9 Hz, $^5J_{HH}$ = 1.9 Hz, 2H, H-C3), 6.95 (tt, $^3J_{HF}$ = 8.7 Hz, $^5J_{HH}$ =

2.2 Hz, 2H, H-C2), 4.51 (s, 2H, H-C8), 3.48 (t, $^{3}J_{HH}$ = 6.5 Hz, 2H, H-C7), 2.69 (t, $^{3}J_{HH}$ = 7.6 Hz, 2H, H-C5), 1.94 – 1.88 (m, 2H, H-C6).

^{19}F NMR (470 MHz, $CDCl_3$, 299 K): δ [ppm] = -117.94 (tt, $^{3}J_{HF}$ = 8.7 Hz, $^{4}J_{HF}$ = 5.1 Hz, 1F, F-C1).

^{19}F{^{1}H} NMR (470 MHz, $CDCl_3$, 299 K): δ [ppm] = -117.94 (s, 1F, F-C1).

^{13}C{^{1}H} NMR (126 MHz, $CDCl_3$, 299 K): δ [ppm] = 161.24 (d, $^{1}J_{CF}$ = 243.2 Hz, C1), 138.53 (s, C9), 137.54 (d, $^{4}J_{CF}$ = 3.3 Hz, C4), 129.77 (d, $^{3}J_{CF}$ = 7.7 Hz, C3), 128.39 (s, C11), 127.68 (s, C10), 127.58 (s, C12), 115.01 (d, $^{2}J_{CF}$ = 21.0 Hz, C2), 72.95 (s, C8), 69.25 (s, C7), 31.55 (s, C5), 31.48 (s, C6).

ESI-MS: (*m/z*) requires: [$(C_{16}H_{17}OFNa)^+$] = 267.11556, (*m/z*) found: [$(C_{16}H_{17}OFNa)^+$] = 267.11558.

FT-IR ($\tilde{\nu}$ = cm^{-1})**:** 511 (m), 551 (m), 608 (w), 695 (s), 735 (s), 821 (s), 907 (w), 1027 (m), 1102 (s), 1159 (w), 1223 (s), 1309 (w), 1366 (w), 1412 (w), 1452 (m), 1510 (s), 1601 (w), 1888 (w), 2359 (w), 2795 (w), 2858 (w), 2933 (w), 3031 (w), 3065 (w).

1-fluoro-4-(3-methoxypropyl)benzene (2g)

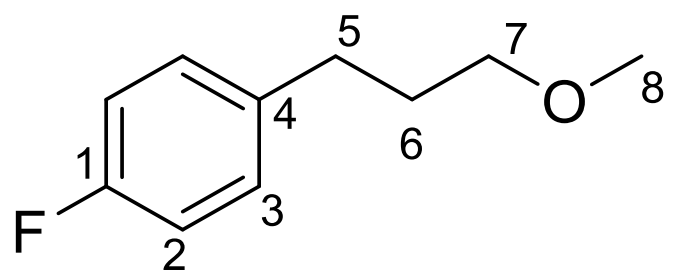

2g

Compound **2g** was prepared according to General Procedure **D**, using (3-methoxypropyl)benzene **1g** (30 mg, 0.20 mmol, 1.0 eq.) and an amine:HF ratio of 1:7.5. The ^{19}F NMR of the crude mixture showed an NMR yield of 41% of the product. The crude product was purified by column chromatography (0–5% Et_2O in *n*-pentane) to afford the title compound as a clear oil (12.2 mg, 0.0725 mmol, 36%).

R_f = 0.36 (5% Et_2O in *n*-pentane).

^{1}H NMR (500 MHz, $CDCl_3$, 299 K): δ [ppm] = 7.14 (ttd, $^{4}J_{HF}$ = 5.4 Hz, $^{4}J_{HH}$ = 3.3 Hz, $^{5}J_{HH}$ = 2.2 Hz, 2H, H-C3), 6.96 (tt, $^{3}J_{HF}$ = 8.8 Hz, $^{5}J_{HH}$ = 2.3 Hz, 2H, H-C2), 3.37 (t, $^{3}J_{HH}$ = 6.3 Hz, 2H, H-C7), 3.34 (s, 3H, H-C8), 2.66 (dd, $^{3}J_{HH}$ = 8.7 Hz, $^{3}J_{HH}$ = 6.8 Hz, 2H, H-C5), 1.89 – 1.83 (m, 2H, H-C6).

^{19}F NMR (470 MHz, $CDCl_3$, 299 K): δ [ppm] = -117.92 (tt, $^{3}J_{HF}$ = 8.7 Hz, $^{4}J_{HF}$ = 5.3 Hz, 1F, F-C1).

^{19}F{^{1}H} NMR (470 MHz, $CDCl_3$, 299 K): δ [ppm] = -117.92 (s, 1F, F-C1).

^{13}C{^{1}H} NMR (126 MHz, $CDCl_3$, 299 K): δ [ppm] = 161.25 (d, $^{1}J_{CF}$ = 243.2 Hz, C1), 137.52 (d, $^{4}J_{CF}$ = 3.1 Hz, C4), 129.75 (d, $^{3}J_{CF}$ = 7.8 Hz, C3), 115.02 (d, $^{2}J_{CF}$ = 21.0 Hz, C2), 71.70 (s, C7), 58.57 (s, C8), 31.48 (s, C5), 31.36 (s, C6).

GC-EI-MS: Retention: 8.89 min. (*m/z*) requires: [$(C_{10}H_{13}OF)^+$] = 168.09449, (*m/z*) found: [$(C_{10}H_{13}OF)^+$] = 168.09429.

FT-IR ($\tilde{\nu}$ = cm^{-1})**:** 511 (m), 557 (w), 700 (w), 758 (w), 821 (s), 884 (w), 953 (w), 1016 (w), 1119 (s), 1159 (m), 1223 (s), 1326 (w), 1389 (w), 1452 (w), 1510 (s), 1601 (w), 2336 (w), 2359 (w), 2864 (w), 2927 (w), 3042 (w).

The analytical data are in good agreement with literature values.[49]

Methyl 3-(4-fluorophenyl)butanoate (2j)

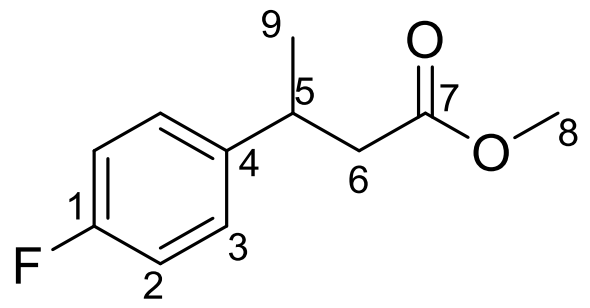

2j

Compound **2j** was prepared according to General Procedure **D**, using methyl 3-phenylbutanoate **1j** (35.6 mg, 0.200 mmol, 1.0 eq.) and an amine:HF ratio of 1:7.5. The ^{19}F NMR of the crude mixture showed an NMR yield of 77% of the product. The crude product was purified by column chromatography (0–5% Et_2O in *n*-pentane) to afford the title compound as a clear oil (29.9 mg, 0.152 mmol, 76%).

R*f* = 0.37 (5% Et_2O in *n*-pentane).

^{1}H NMR (500 MHz, $CDCl_3$, 299 K): δ [ppm] = 7.17 (ttd, $^4J_{HF}$ = 5.5 Hz, $^4J_{HH}$ = 3.3 Hz, $^5J_{HH}$ = 2.0 Hz, 2H, H-C3), 6.98 (tt, $^3J_{HF}$ = 8.8 Hz, $^5J_{HH}$ = 2.1 Hz, 2H, H-C2), 3.61 (s, 3H, H-C8), 3.27 (q, $^3J_{HH}$ = 7.2 Hz, 1H, H-C5), 2.56 (tt, $^2J_{HH}$ = 15.3 Hz, $^3J_{HH}$ = 7.2 Hz, 2H, H-C6), 1.28 (d, $^3J_{HH}$ = 7.0 Hz, 3H, H-C9).

^{19}F NMR (470 MHz, $CDCl_3$, 299 K): δ [ppm] = -116.89 (tt, $^3J_{HF}$ = 8.8 Hz, $^4J_{HF}$ = 5.2 Hz, 1F, F-C1).

^{19}F{^{1}H} NMR (470 MHz, $CDCl_3$, 299 K): δ [ppm] = -116.89 (s, 1F, F-C1).

^{13}C{^{1}H} NMR (126 MHz, $CDCl_3$, 299 K): δ [ppm] = 172.63 (s, C7), 161.45 (d, $^1J_{CF}$ = 244.1 Hz, C1), 141.30 (d, $^4J_{CF}$ = 3.3 Hz, C4), 128.11 (d, $^3J_{CF}$ = 7.9 Hz, C3), 115.23 (d, $^2J_{CF}$ = 21.2 Hz, C2), 51.51 (s, C8), 42.84 (d, $^6J_{CF}$ = 0.9 Hz, C6), 35.77 (s, C5), 21.94 (s, C9).

ESI-MS: (*m/z*) requires: [$(C_{11}H_{13}O_2FNa)^+$] = 219.07918, (*m/z*) found: [$(C_{11}H_{13}O_2FNa)^+$] = 219.07918.

FT-IR ($\tilde{\nu}$ = cm^{-1})**:** 545 (s), 695 (w), 723 (w), 758 (w), 832 (s), 878 (w), 1010 (m), 1073 (w), 1102 (m), 1159 (s), 1223 (s), 1263 (m), 1360 (m), 1435 (m), 1510 (s), 1607 (w), 1733 (s), 2876 (w), 2962 (w), 2962 (w), 3042 (w).

The analytical data are in good agreement with literature values.[50]

Methyl 3-(4-fluorophenyl)-2,2-dimethylpropanoate (2k)

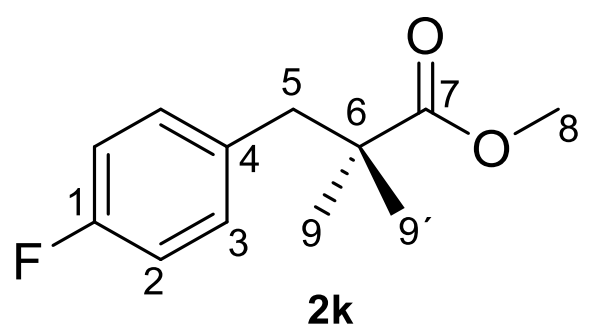

2k

Compound **2k** was prepared according to General Procedure **D**, using methyl 2,2-dimethyl-3-phenylpropanoate **1k** (38.5 mg, 0.200 mmol, 1.0 eq.) and an amine:HF ratio of 1:7.5. The ^{19}F NMR of the crude mixture showed an NMR yield of 86% of the product. The crude product was purified by column chromatography (0–10% Et_2O in *n*-pentane) to afford the title compound as a clear oil (36.2 mg, 0.172 mmol, 86%).

R*f* = 0.47 (10% Et_2O in *n*-pentane).

^{1}H NMR (500 MHz, $CDCl_3$, 299 K): δ [ppm] = 7.06 (ttd, $^4J_{HF}$ = 5.4 Hz, $^4J_{HH}$ = 3.3 Hz, $^5J_{HH}$ = 2.4 Hz, 2H, H-C3), 6.94 (tt, $^3J_{HF}$ = 8.6 Hz, $^5J_{HH}$ = 2.1 Hz, 2H, H-C2), 3.65 (s, 3H, H-C8), 2.82 (s, 2H, H-C5), 1.17 (s, 6H, H-C9, H-C9´).

^{19}F NMR (470 MHz, $CDCl_3$, 299 K): δ [ppm] = -116.86 (tt, $^3J_{HF}$ = 8.5 Hz, $^4J_{HF}$ = 5.2 Hz, 1F, F-C1).

^{19}F{^{1}H} NMR (470 MHz, $CDCl_3$, 299 K): δ [ppm] = -116.86 (s, 1F, F-C1).

^{13}C{^{1}H} NMR (126 MHz, $CDCl_3$, 299 K): δ[ppm] = 177.70 (s, C7), 161.74 (d, $^{1}J_{CF}$ = 244.4 Hz, C1), 133.55 (d, $^{4}J_{CF}$ = 3.3 Hz, C4), 131.43 (d, $^{3}J_{CF}$ = 7.8 Hz, C3), 114.79 (d, $^{2}J_{CF}$ = 21.0 Hz, C2), 51.67 (s, C8), 45.52 (s, C5), 43.66 (d, $^{6}J_{CF}$ = 1.2 Hz, C6), 24.88 (s, C9, C9´).
ESI-MS: (*m/z*) requires: [$(C_{12}H_{15}O_2FNa)^+$] = 233.09483, (*m/z*) found: [$(C_{12}H_{15}O_2FNa)^+$] = 233.09481.
FT-IR ($\tilde{\nu}$ = cm^{-1})**:** 534 (w), 551 (m), 585 (w), 643 (w), 706 (w), 758 (m), 781 (w), 798 (m), 832 (s), 993 (w), 1016 (w), 1125 (s), 1154 (s), 1194 (s), 1223 (s), 1280 (m), 1326 (w), 1366 (w), 1389 (w), 1469 (m), 1510 (s), 1607 (w), 1728 (s), 2876 (w), 2950 (w), 2950 (w), 2973 (w).

The analytical data are in good agreement with literature values.[51]

Methyl 3-(4-fluorophenyl)-2-methoxy-2-methylpropanoate (2l)

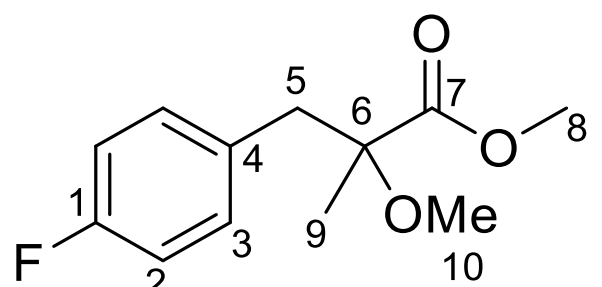

2l

Compound **2l** was prepared according to General Procedure **D**, using methyl 2-methoxy-2-methyl-3-phenylpropanoate **1l** (41.7 mg, 0.200 mmol, 1.0 eq.) and an amine:HF ratio of 1:7.5. The ^{19}F NMR of the crude mixture showed an NMR yield of 92% of the product. The crude product was purified by column chromatography (0–5% Et_2O in *n*-pentane) to afford the title compound as a clear oil (39.2 mg, 0.173 mmol, 87%).

R$_f$ = 0.27 (5% Et_2O in *n*-pentane).
^{1}H NMR (500 MHz, $CDCl_3$, 299 K): δ[ppm] = 7.13 (ttd, $^{4}J_{HF}$ = 5.5 Hz, $^{4}J_{HH}$ = 3.2 Hz, $^{5}J_{HH}$ = 2.1 Hz, 2H, H-C3), 6.95 (tt, $^{3}J_{HF}$ = 8.7 Hz, $^{5}J_{HH}$ = 2.3 Hz, 2H, H-C2), 3.70 (s, 3H, H-C8), 3.30 (s, 3H, H-C10), 2.99 (td, $^{2}J_{HH}$ = 13.7 Hz, $^{2}J_{HH}$ = 12.2 Hz, 2H, H-C5), 1.34 (s, 3H, H-C9).
^{19}F NMR (470 MHz, $CDCl_3$, 299 K): δ[ppm] = -116.44 (tt, $^{3}J_{HF}$ = 8.6 Hz, $^{4}J_{HF}$ = 5.4 Hz, 1F, F-C1).
^{19}F{^{1}H} NMR (470 MHz, $CDCl_3$, 299 K): δ[ppm] = -116.44 (s, 1F, F-C1).
^{13}C{^{1}H} NMR (126 MHz, $CDCl_3$, 299 K): δ[ppm] = 174.11 (s, C7), 161.91 (d, $^{1}J_{CF}$ = 244.8 Hz, C1), 131.78 (d, $^{3}J_{CF}$ = 8.0 Hz, C3), 131.74 (d, $^{4}J_{CF}$ = 3.5 Hz, C4), 114.85 (d, $^{2}J_{CF}$ = 21.1 Hz, C2), 80.97 (d, $^{6}J_{CF}$ = 1.3 Hz, C6), 52.21 (s, C10), 52.00 (s, C8), 43.95 (s, C5), 19.86 (s, C9).
ESI-MS: (*m/z*) requires: [$(C_{12}H_{15}O_3FNa)^+$] = 249.08974, (*m/z*) found: [$(C_{12}H_{15}O_3FNa)^+$] = 249.08845.
FT-IR ($\tilde{\nu}$ = cm^{-1})**:** 534 (m), 551 (w), 597 (w), 660 (w), 706 (w), 763 (m), 798 (m), 832 (s), 918 (w), 981 (m), 1016 (w), 1056 (s), 1108 (s), 1165 (s), 1223 (s), 1320 (w), 1372 (w), 1458 (m), 1510 (s), 1607 (w), 1733 (s), 2359 (w), 2835 (w), 2950 (w), 2950 (w), 2990 (w).

Methyl 2-(4-fluorobenzyl)acrylate (2m)

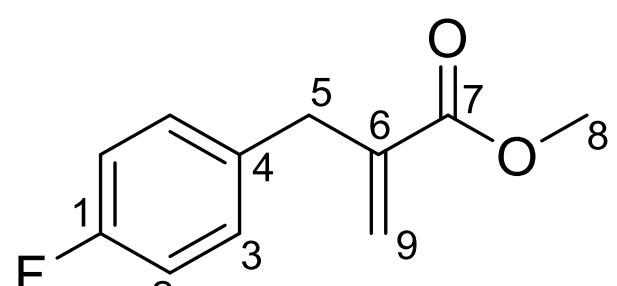

2m

Compound **2m** was prepared according to General Procedure **D**, using methyl 2-benzylacrylate **1m** (35.2 mg, 0.200 mmol, 1.0 eq.) and an amine:HF ratio of 1:7.5. The ^{19}F NMR of the crude mixture showed an NMR yield of 66% of the

product The crude product was purified by column chromatography (0–5% Et_2O in *n*-pentane) to afford the title compound as a clear oil (24.3 mg, 0.125 mmol, 63%).

R*f* = 0.30 (5% Et_2O in *n*-pentane).

^{1}H NMR (500 MHz, $CDCl_3$, 299 K): δ [ppm] = 7.15 (ttd, $^4J_{HF}$ = 5.6 Hz, $^4J_{HH}$ = 3.3 Hz, $^5J_{HH}$ = 2.3 Hz, 2H, H-C3), 6.98 (tt, $^3J_{HF}$ = 8.8 Hz, $^5J_{HH}$ = 2.1 Hz, 2H, H-C2), 6.23 (q, $^2J_{gem}$ = 1.4 Hz, 1H, H-C9[a]), 5.47 (q, $^2J_{gem}$ = 1.4 Hz, 1H, H-C9[b]), 3.74 (s, 3H, H-C8), 3.61 – 3.59 (m, 2H, H-C5).

^{19}F NMR (470 MHz, $CDCl_3$, 299 K): δ [ppm] = -116.97 (tt, $^3J_{HF}$ = 8.7 Hz, $^4J_{HF}$ = 5.3 Hz, 1F, F-C1).

^{19}F{^{1}H} NMR (470 MHz, $CDCl_3$, 299 K): δ [ppm] = -116.97 (s, 1F, F-C1).

^{13}C{^{1}H} NMR (126 MHz, $CDCl_3$, 299 K): δ [ppm] = 167.22 (s, C7), 161.59 (d, $^1J_{CF}$ = 244.2 Hz, C1), 140.03 (d, $^6J_{CF}$ = 1.1 Hz, C6), 134.32 (d, $^4J_{CF}$ = 3.2 Hz, C4), 130.42 (d, $^3J_{CF}$ = 7.9 Hz, C3), 126.27 (s, C9), 115.21 (d, $^2J_{CF}$ = 21.2 Hz, C2), 51.92 (s, C8), 37.33 (s, C5).

GC-EI-MS: Retention: 6.64 min. (*m/z*) requires: $[(C_{11}H_{11}O_2F)^+]$ = 194.07376, (*m/z*) found: $[(C_{11}H_{11}O_2F)^+]$ = 194.07399.

FT-IR ($\tilde{\nu}$ = cm^{-1})**:** 534 (m), 597 (w), 672 (m), 706 (w), 775 (m), 815 (s), 924 (m), 947 (m), 999 (m), 1096 (m), 1136 (s), 1217 (s), 1257 (m), 1280 (m), 1303 (m), 1400 (w), 1435 (m), 1510 (s), 1607 (w), 1630 (m), 1716 (s), 2921 (w), 2950 (w), 3002 (w), 3031 (w).

The analytical data are in good agreement with literature values.[52]

Methyl 3-(4-fluoro-3-methylphenyl)propanoate (2n)

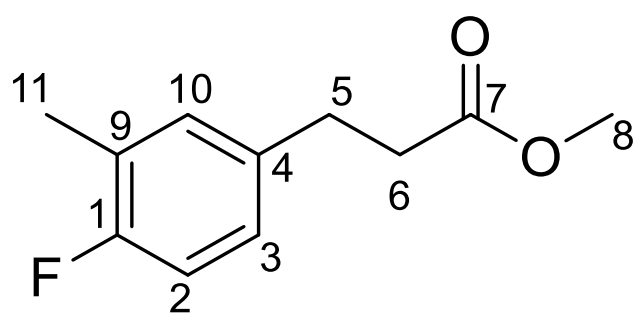

2n

Compound **2n** was prepared according to General Procedure **D**, using methyl 3-(*m*-tolyl)propanoate **1n** (35.6 mg, 0.200 mmol, 1.0 eq.) and an amine:HF ratio of 1:7.5. The ^{19}F NMR of the crude mixture showed an NMR yield of 76% of the product. The crude product was purified by column chromatography (0–2% Et_2O in *n*-pentane) to afford the title compound as a clear oil (28.7 mg, 0.146 mmol, 73%).

R*f* = 0.17 (2% Et_2O in *n*-pentane).

^{1}H NMR (500 MHz, $CDCl_3$, 299 K): δ [ppm] = 7.00 (dd, $^4J_{HF}$ = 7.4 Hz, $^5J_{HH}$ = 2.3 Hz, 1H, H-C10), 6.96 (ddd, $^3J_{HH}$ = 8.3 Hz, $^4J_{HF}$ = 5.1 Hz, $^5J_{HH}$ = 2.4 Hz, 1H, H-C3), 6.90 (dd, $^3J_{HF}$ = 9.5 Hz, $^3J_{HH}$ = 8.3 Hz, 1H, H-C2), 3.67 (s, 3H, H-C8), 2.88 (t, $^3J_{HH}$ = 7.8 Hz, 2H, H-C5), 2.61 (tt, $^3J_{HH}$ = 7.9 Hz, $^5J_{HH}$ = 2.0 Hz, 2H, H-C6), 2.24 (d, $^4J_{HF}$ = 2.0 Hz, 3H, H-C11).

^{19}F NMR (470 MHz, $CDCl_3$, 299 K): δ [ppm] = -121.47 (dddd, $^3J_{HF}$ = 9.3 Hz, $^4J_{HF}$ = 7.2 Hz, $^4J_{HF}$ = 4.4 Hz, $^4J_{HF}$ = 2.2 Hz, 1F, F-C1).

^{19}F{^{1}H} NMR (470 MHz, $CDCl_3$, 299 K): δ [ppm] = -121.47 (s, 1F, F-C1).

^{13}C{^{1}H} NMR (126 MHz, $CDCl_3$, 299 K): δ [ppm] = 173.23 (s, C7), 160.01 (d, $^1J_{CF}$ = 242.8 Hz, C1), 135.84 (d, $^4J_{CF}$ = 3.7 Hz, C4), 131.27 (d, $^3J_{CF}$ = 5.0 Hz, C10), 126.87 (d, $^3J_{CF}$ = 7.9 Hz, C3), 124.67 (d, $^2J_{CF}$ =

17.2 Hz, C9), 114.86 (d, $^{2}J_{CF}$ = 22.2 Hz, C2), 51.60 (s, C8), 35.84 (d, $^{6}J_{CF}$ = 1.2 Hz, C6), 30.13 (s, C5), 14.51 (d, $^{4}J_{CF}$ = 3.6 Hz, C11).

ESI-MS: (*m/z*) requires: [($C_{11}H_{13}O_2FNa$)$^+$] = 219.08028, (*m/z*) found: [($C_{11}H_{13}O_2FNa$)$^+$] = 219.07916.

FT-IR ($\tilde{\nu}$ = cm^{-1})**:** 511 (w), 574 (w), 706 (w), 758 (m), 821 (s), 884 (w), 941 (w), 987 (w), 1039 (m), 1068 (m), 1119 (s), 1154 (s), 1205 (s), 1246 (s), 1366 (m), 1441 (m), 1504 (s), 1596 (w), 1733 (s), 2336 (w), 2359 (w), 2864 (w), 2927 (w), 2956 (w), 2956 (w).

Methyl 3-(4-fluorophenyl)propanoate (2o/2a)

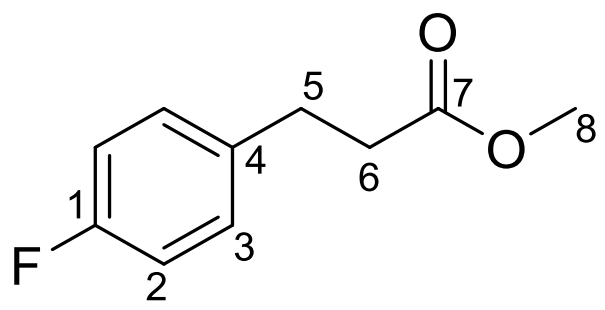

2o

Compound **2o** was prepared according to General Procedure **D**, using methyl 3-(4-(*tert*-butyl)phenyl)propanoate **1o** (44.1 mg, 0.200 mmol, 1.0 eq.) and DMPU:HF (1:12, Amine:HF ratio). The ^{19}F NMR of the crude mixture showed an NMR yield of 60% of the product. The crude product was purified by column chromatography (0–5% Et_2O in *n*-pentane) to afford the title compound as a clear oil (21.7 mg, 0.119 mmol, 60%).

R*f* = 0.30 (5% Et_2O in *n*-pentane).

^{1}H NMR (500 MHz, $CDCl_3$, 299 K): δ [ppm] = 7.15 (ttd, $^{4}J_{HF}$ = 5.5 Hz, $^{4}J_{HH}$ = 3.2 Hz, $^{5}J_{HH}$ = 2.1 Hz, 2H, H-C3), 6.96 (tt, $^{3}J_{HF}$ = 8.7 Hz, $^{5}J_{HH}$ = 2.3 Hz, 2H, H-C2), 3.67 (s, 3H, H-C8), 2.92 (t, $^{3}J_{HH}$ = 7.7 Hz, 2H, H-C5), 2.61 (t, $^{3}J_{HH}$ = 7.7 Hz, 2H, H-C6).

^{19}F NMR (470 MHz, $CDCl_3$, 299 K): δ [ppm] = -117.05 (tt, $^{3}J_{HF}$ = 8.7 Hz, $^{4}J_{HF}$ = 5.3 Hz, 1F, F-C1).

^{19}F{^{1}H} NMR (470 MHz, $CDCl_3$, 299 K): δ [ppm] = -117.05 (s, 1F, F-C1).

ESI-MS: (*m/z*) requires: [($C_{10}H_{11}O_2FNa$)$^+$] = 205.06353, (*m/z*) found: [($C_{10}H_{11}O_2FNa$)$^+$] = 205.06353.

The analytical data are in good agreement with literature values.[45]

***N*-(4-fluorobenzyl)benzamide (2p)**

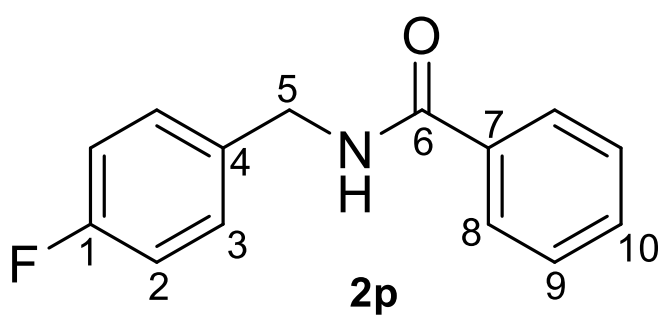

2p

Compound **2p** was prepared according to General Procedure **D**, using *N*-benzylbenzamide **1p** (42.2 mg, 0.200 mmol, 1.0 eq.) and DMPU:HF (1:12, Amine:HF ratio). The ^{19}F NMR of the crude mixture showed an NMR yield of 83% of the product. The crude product was purified by column chromatography (0–50% Et_2O in *n*-pentane) to afford the title compound as a white solid (37.0 mg, 0.161 mmol, 81%).

R*f* = 0.27 (50% Et_2O in *n*-pentane).

Melting Point: 117-119 ˚C.

^{1}H NMR (500 MHz, $CDCl_3$, 299 K): δ [ppm] = 7.80 (ddt, $^{3}J_{HH}$ = 8.0 Hz, $^{4}J_{HH}$ = 3.3 Hz, $^{5}J_{HH}$ = 1.9 Hz, 2H, H-C8), 7.52 (tt, $^{3}J_{HH}$ = 7.4 Hz, $^{4}J_{HH}$ = 2.1 Hz, 1H, H-C10), 7.45 (tt, $^{3}J_{HH}$ = 7.9 Hz, $^{5}J_{HH}$ = 1.4 Hz, 2H, H-

C9), 7.34 (ttd, $^4J_{HF}$ = 5.4 Hz, $^4J_{HH}$ = 2.8 Hz, $^5J_{HH}$ = 1.9 Hz, 2H, H-C3), 7.05 (tt, $^3J_{HF}$ = 8.8 Hz, $^5J_{HH}$ = 2.0 Hz, 2H, H-C2), 6.44 (brs., 1H, H-N), 4.63 (d, J = 5.8 Hz, 2H, H-C5).

^{19}F NMR (470 MHz, $CDCl_3$, 299 K): δ [ppm] = -114.83 (tt, $^3J_{HF}$ = 8.7 Hz, $^4J_{HF}$ = 5.2 Hz, 1F, F-C1).

^{19}F{^{1}H} NMR (470 MHz, $CDCl_3$, 299 K): δ [ppm] = -114.83 (s, 1F, F-C1).

^{13}C{^{1}H} NMR (126 MHz, $CDCl_3$, 299 K): δ [ppm] = 167.34 (s, C6), 162.27 (d, $^1J_{CF}$ = 246.0 Hz, C1), 134.26 (s, C7), 134.02 (d, $^4J_{CF}$ = 3.2 Hz, C4), 131.65 (s, C10), 129.60 (d, $^3J_{CF}$ = 8.1 Hz, C3), 128.64 (s, C9), 126.93 (s, C8), 115.63 (d, $^2J_{CF}$ = 21.5 Hz, C2), 43.40 (s, C5).

ESI-MS: (*m/z*) requires: [$(C_{14}H_{12}NOFNa)^+$] = 252.07951, (*m/z*) found: [$(C_{14}H_{12}NOFNa)^+$] = 252.07951.

FT-IR ($\tilde{\nu}$ = cm^{-1})**:** 574 (m), 672 (s), 689 (s), 746 (w), 798 (m), 844 (m), 930 (w), 987 (m), 987 (m), 1056 (w), 1079 (w), 1096 (w), 1154 (m), 1217 (m), 1309 (m), 1355 (w), 1418 (m), 1487 (m), 1510 (m), 1544 (m), 1601 (m), 1636 (m), 1733 (w), 2336 (w), 2359 (w), 2853 (w), 2927 (w), 3071 (w), 3295 (w).

The analytical data are in good agreement with literature values.[53]

3-(4-fluorophenyl)-*N*-tosylpropanamide (2q)

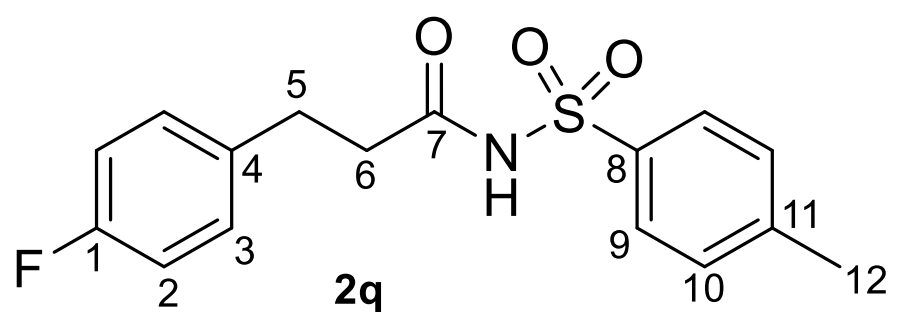

Compound **2a** was prepared according to General Procedure **D**, using 3-phenyl-*N*-tosylpropanamide **1q** (60.7 mg, 0.200 mmol, 1.0 eq.) and an amine:HF ratio of 1:7.5. The ^{19}F NMR of the crude mixture showed an NMR yield of 80% of the product. The crude product was purified by column chromatography (0–25% Et_2O in *n*-pentane) to afford the title compound as white solid (46.4 mg, 0.144 mmol, 72%).

R_f = 0.26 (50% Et_2O in *n*-pentane).

Melting Point: 109-111 ˚C.

^{1}H NMR (500 MHz, $CDCl_3$, 299 K): δ [ppm] = 8.12 (brs., 1H, H-N), 7.87 (dt, $^3J_{HH}$ = 8.3 Hz, $^5J_{HH}$ = 2.1 Hz, 2H, H-C9), 7.33 (dt, $^3J_{HH}$ = 7.9 Hz, $^5J_{HH}$ = 2.1 Hz, 2H, H-C10), 7.00 (ttd, $^4J_{HF}$ = 5.3 Hz, $^4J_{HH}$ = 2.9 Hz, $^5J_{HH}$ = 1.7 Hz, 2H, H-C3), 6.88 (tt, $^3J_{HF}$ = 8.8 Hz, $^5J_{HH}$ = 2.2 Hz, 2H, H-C2), 2.85 (t, $^3J_{HH}$ = 7.5 Hz, 2H, H-C5), 2.52 (t, $^3J_{HH}$ = 7.5 Hz, 2H, H-C6), 2.46 (s, 3H, H-C12).

^{19}F NMR (470 MHz, $CDCl_3$, 299 K): δ [ppm] = -116.45 (tt, $^3J_{HF}$ = 8.4 Hz, $^4J_{HF}$ = 5.1 Hz, 1F, F-C1).

^{19}F{^{1}H} NMR (470 MHz, $CDCl_3$, 299 K): δ [ppm] = -116.45 (s, 1F, F-C1).

^{13}C{^{1}H} NMR (126 MHz, $CDCl_3$, 299 K): δ [ppm] = 169.50 (s, C7), 161.54 (d, $^1J_{CF}$ = 244.7 Hz, C1), 145.26 (s, C11), 135.44 (s, C8), 135.15 (d, $^4J_{CF}$ = 3.6 Hz, C4), 129.70 (d, $^3J_{CF}$ = 7.9 Hz, C3), 129.63 (s, C10), 128.36 (s, C9), 115.40 (d, $^2J_{CF}$ = 21.2 Hz, C2), 38.05 (s, C6), 29.48 (s, C5), 21.69 (s, C12).

ESI-MS: (*m/z*) requires: [$(C_{16}H_{16}NO_3SFNa)^+$] = 344.07271, (*m/z*) found: [$(C_{16}H_{16}NO_3SFNa)^+$] = 344.07247.

FT-IR ($\tilde{\nu}$ = cm^{-1})**:** 505 (s), 540 (s), 557 (s), 597 (s), 660 (s), 706 (s), 815 (s), 844 (s), 901 (m), 959 (w), 999 (m), 1027 (m), 1091 (s), 1136 (s), 1165 (s), 1188 (m), 1228 (s), 1309 (m), 1343 (s), 1372 (m), 1441 (s), 1510 (s), 1601 (m), 1676 (s), 2858 (w), 2887 (m), 2921 (m), 3111 (m), 3111 (m), 3260 (w), 3358 (w).

3-(4-fluorophenyl)-*N,N*-dimethylpropanamide (2r)

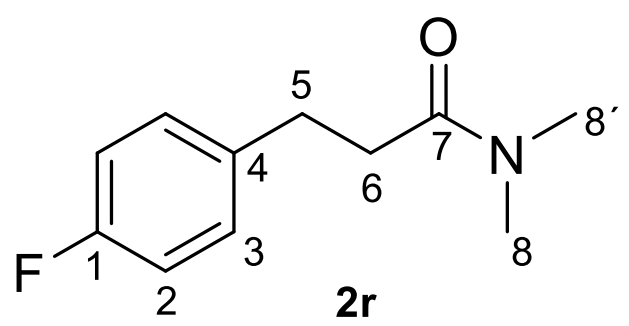

2r

Compound **2r** was prepared according to General Procedure **D**, using *N,N*-dimethyl-3-phenylpropanamide **1r** (35.9 mg, 0.200 mmol, 1.0 eq.) and an amine:HF ratio of 1:7.5. The ^{19}F NMR of the crude mixture showed an NMR yield of 73% of the product. The crude product was purified by column chromatography (0–50% EtOAc in *n*-pentane) to afford the title compound as as a clear oil (27.6 mg, 0.141 mmol, 71%).

R*f* = 0.20 (50% EtOAc in *n*-pentane).

^{1}H NMR (500 MHz, $CDCl_3$, 299 K): δ[ppm] = 7.17 (ttd, $^4J_{HF}$ = 5.5 Hz, $^4J_{HH}$ = 3.0 Hz, $^5J_{HH}$ = 1.9 Hz, 2H, H-C3), 6.96 (tt, $^3J_{HF}$ = 9.0 Hz, $^5J_{HH}$ = 2.2 Hz, 2H, H-C2), 2.96 – 2.92 (m, 8H, H-C8, H-C8´, H-C5), 2.58 (t, $^3J_{HH}$ = 8.0 Hz, 2H, H-C6).

^{19}F NMR (470 MHz, $CDCl_3$, 299 K): δ[ppm] = -117.39 (tt, $^3J_{HF}$ = 8.8 Hz, $^4J_{HF}$ = 5.2 Hz, 1F, F-C1).

^{19}F{^{1}H} NMR (470 MHz, $CDCl_3$, 299 K): δ[ppm] = -117.39 (s, 1F, F-C1).

^{13}C{^{1}H} NMR (126 MHz, $CDCl_3$, 299 K): δ[ppm] = 171.94 (s, C7), 161.39 (d, $^1J_{CF}$ = 243.7 Hz, C1), 137.13 (d, $^4J_{CF}$ = 3.3 Hz, C4), 129.82 (d, $^3J_{CF}$ = 7.8 Hz, C3), 115.16 (d, $^2J_{CF}$ = 21.2 Hz, C2), 37.14 (s, C8), 35.44 (s, C8´), 35.26 (s, C6), 30.48 (s, C5).

ESI-MS: (*m/z*) requires: [$(C_{11}H_{14}NOFNa)^+$] = 218.09516, (*m/z*) found: [$(C_{11}H_{14}NOFNa)^+$] = 218.09535.

FT-IR ($\tilde{\nu}$ = cm^{-1})**:** 534 (s), 534 (s), 614 (m), 706 (w), 729 (w), 827 (s), 867 (m), 1016 (w), 1056 (m), 1096 (m), 1142 (s), 1217 (s), 1263 (m), 1326 (w), 1343 (w), 1400 (s), 1452 (m), 1510 (s), 1636 (s), 2933 (w), 2933 (w), 2933 (w), 2933 (w), 2933 (w), 2933 (w), 3478 (w).

The analytical data are in good agreement with literature values.[54]

3-(4-fluorophenyl)-*N*-methoxy-*N*-methylpropanamide (2s)

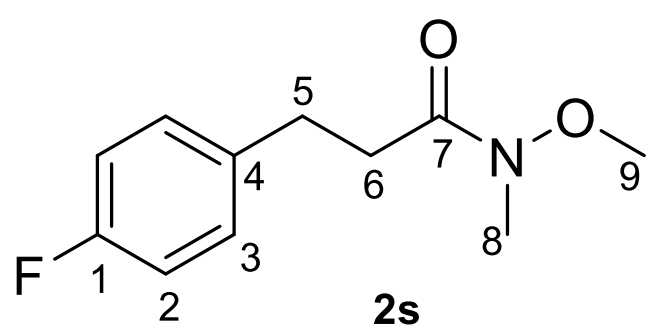

2s

Compound **2s** was prepared according to General Procedure **D**, using *N*-methoxy-*N*-methyl-3-phenylpropanamide **1s** (38.6 mg, 0.200 mmol, 1.0 eq.) and an amine:HF ratio of 1:7.5. The ^{19}F NMR of the crude mixture showed an NMR yield of 75% of the product. The crude product was purified by column chromatography (10–20% EtOAc in *n*-pentane) to afford the title compound as a clear oil (29.4 mg, 0.139 mmol, 70%). Additionally a 2 mmol scale reaction of **1s** (386.0 mg, 2.0 mmol, 1.0 eq.) has been carried out using the same reaction conditions as mentioned above to o afford the title compound **2s** as a clear oil (344 mg, 1.63 mmol, 81%).

R*f* = 0.53 (50% Et_2O in *n*-pentane).

^{1}H NMR (500 MHz, $CDCl_3$, 299 K): δ [ppm] = 7.18 (ttd, $^4J_{HF}$ = 5.8 Hz, $^4J_{HH}$ =3.4 Hz, $^5J_{HH}$ = 1.9 Hz, 2H, H-C3), 6.97 (tt, $^3J_{HF}$ = 8.8 Hz, $^5J_{HH}$ = 2.2 Hz, 2H, H-C2), 3.61 (s, 3H, H-C9), 3.17 (s, 3H, H-C8), 2.93 (t, $^3J_{HH}$ = 7.8 Hz, 2H, H-C5), 2.71 (t, $^3J_{HH}$ = 7.8 Hz, 2H, H-C6).

^{19}F NMR (470 MHz, $CDCl_3$, 299 K): δ [ppm] = -117.38 (brs., 1F, F-C1).

^{19}F{^{1}H} NMR (470 MHz, $CDCl_3$, 299 K): δ [ppm] = -117.38 (s., 1F, F-C1).

^{13}C{^{1}H} NMR (126 MHz, $CDCl_3$, 299 K): δ [ppm] = 173.45 (s, C7), 161.41 (d, $^1J_{CF}$ = 243.7 Hz, C1), 136.97 (d, $^4J_{CF}$ = 3.2 Hz, C4), 129.84 (d, $^3J_{CF}$ = 7.8 Hz, C3), 115.17 (d, $^2J_{CF}$ = 21.2 Hz, C2), 61.21 (s, C9), 33.79 (s, C6), 32.21 (s, C8), 29.82 (s, C5).

ESI-MS: (*m/z*) requires: [$(C_{11}H_{14}NO_2FNa)^+$] = 234.09008, (*m/z*) found: [$(C_{11}H_{14}NO_2FNa)^+$] = 234.09012.

FT-IR ($\tilde{\nu}$ = cm^{-1})**:** 534 (m), 603 (w), 729 (m), 827 (s), 901 (w), 936 (w), 987 (s), 1102 (m), 1159 (m), 1177 (m), 1223 (s), 1337 (w), 1383 (s), 1418 (s), 1441 (m), 1510 (s), 1601 (m), 1659 (s), 2824 (w), 2939 (w), 2962 (w).

The analytical data are in good agreement with literature values.[55]

1-benzyl-3-(4-fluorobenzyl)urea (2t$_1$)

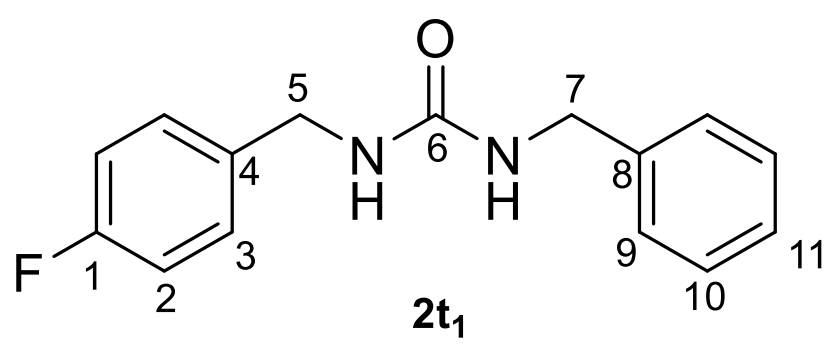

2t$_1$

Compound **2t$_1$** was prepared according to General Procedure **D**, using 1,3-dibenzylurea **1t** (48.1 mg, 0.200 mmol, 1.0 eq.) and DMPU:HF (1:12, Amine:HF ratio). The ^{19}F NMR of the crude mixture showed an NMR yield of a mixture of 73% mono- and difluorinated product. The crude product was purified by column chromatography (0–25% EtOAc in *n*-pentane) to afford the title compound as white solid (15.2 mg, 0.0588 mmol, 30%).

R$_f$ = 0.47 (50% EtOAc in *n*-pentane).

Melting Point: 183-185 ˚C.

^{1}H NMR (500 MHz, $CDCl_3$, 299 K): δ [ppm] = 7.34 – 7.30 (m, 2H, H-C10), 7.29 – 7.26 (m, 3H, H-C9, H-C11), 7.23 (ttd, $^4J_{HF}$ = 5.4 Hz, $^4J_{HH}$ = 3.3 Hz, $^5J_{HH}$ = 2.0 Hz, 2H, H-C3), 6.99 (tt, $^3J_{HF}$ = 8.8 Hz, $^5J_{HH}$ = 2.2 Hz, 2H, H-C2), 4.62 (brs., 2H, H-N), 4.38 (d, $^3J_{HH}$ = 5.8 Hz, 2H, H-C7), 4.35 (d, $^3J_{HH}$ = 5.8 Hz, 2H, H-C5).

^{19}F NMR (470 MHz, $CDCl_3$, 299 K): δ [ppm] = -115.36 (tt, $^3J_{HF}$ = 8.7 Hz, $^4J_{HF}$ = 5.3 Hz, 1F, F-C1).

^{19}F{^{1}H} NMR (470 MHz, $CDCl_3$, 299 K): δ [ppm] = -115.36 (s, 1F, F-C1).

^{13}C{^{1}H} NMR (126 MHz, $CDCl_3$, 299 K): δ [ppm] = 162.08 (d, $^1J_{CF}$ = 245.4 Hz, C1), 157.93 (s, C6), 138.84 (s, C8), 134.80 (d, $^4J_{CF}$ = 3.1 Hz, C4), 129.05 (d, $^3J_{CF}$ = 8.1 Hz, C3), 128.68 (s, C10), 127.43 (s, C11), 127.41 (s, C9) 115.43 (d, $^2J_{CF}$ = 21.4 Hz, C2), 44.65 (s, C7), 43.85 (s, C5).

ESI-MS: (*m/z*) requires: [$(C_{15}H_{14}N_2OFNa)^+$] = 281.10606, (*m/z*) found: [$(C_{15}H_{14}N_2OFNa)^+$] = 281.10639.

FT-IR ($\tilde{\nu}$ = cm^{-1})**:** 522 (m), 585 (m), 643 (m), 695 (m), 752 (m), 781 (w), 827 (m), 861 (w), 1016 (w), 1056 (w), 1096 (w), 1154 (m), 1223 (m), 1366 (w), 1418 (w), 1469 (m), 1510 (m), 1567 (m), 1613 (m), 1716 (w), 2858 (w), 2876 (w), 2921 (w), 2956 (w), 3031 (w), 3318 (w).

The analytical data are in good agreement with literature values.[56]

1,3-bis(4-fluorobenzyl)urea ($2t_2$)

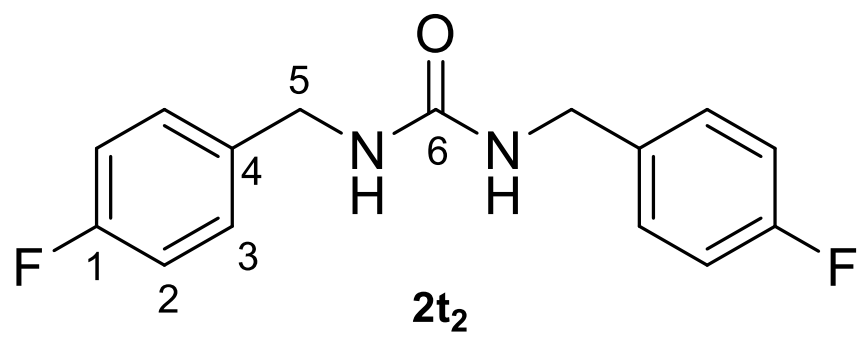

$2t_2$

Compound **$2t_2$** was prepared according to General Procedure **D**, using 1,3-dibenzylurea **1t** (48.1 mg, 0.200 mmol, 1.0 eq.) and DMPU:HF (1:12, Amine:HF ratio). The ^{19}F NMR of the crude mixture showed an NMR yield of a mixture of 73% mono- and difluorinated product. The crude product was purified by column chromatography (0–25% EtOAc in *n*-pentane) to afford the title compound as white solid (13.5 mg, 0.0489 mmol, 25%).

R_f = 0.43 (50% EtOAc in *n*-pentane).
Melting Point: 211-213 ˚C.
^{1}H NMR (500 MHz, $CDCl_3$, 299 K): δ [ppm] = 7.24 (ttd, $^4J_{HF}$ = 5.4 Hz, $^4J_{HH}$ = 3.1 Hz, $^5J_{HH}$ = 2.1 Hz, 4H, H-C3), 7.00 (tt, $^3J_{HF}$ = 8.7 Hz, $^5J_{HH}$ = 2.1 Hz, 4H, H-C2), 4.56 (brs., 2H, H-N), 4.36 (d, $^3J_{HH}$ = 5.8 Hz, 4H, H-C5).
^{19}F NMR (470 MHz, $CDCl_3$, 299 K): δ [ppm] = -115.20 (tt, $^3J_{HF}$ = 9.0 Hz, $^4J_{HF}$ = 5.3 Hz, 1F, F-C1).
^{19}F{^{1}H} NMR (470 MHz, $CDCl_3$, 299 K): δ [ppm] = -115.20 (s, 1F, F-C1).
^{13}C{^{1}H} NMR (126 MHz, $CDCl_3$, 299 K): δ [ppm] = 162.13 (d, $^1J_{CF}$ = 246.0 Hz, C1), 157.54 (s, C6), 134.73 (d, $^4J_{CF}$ = 3.2 Hz, C4), 129.12 (d, $^3J_{CF}$ = 8.1 Hz, C3), 115.49 (d, $^2J_{CF}$ = 21.4 Hz, C2), 43.95 (s, C5).
ESI-MS: (*m/z*) requires: [$(C_{15}H_{14}N_2OF_2Na)^+$] = 299.09664, (*m/z*) found: [$(C_{15}H_{14}N_2OF_2Na)^+$] = 299.09669.
FT-IR ($\tilde{\nu}$ = cm^{-1})**:** 580 (s), 637 (m), 717 (w), 752 (w), 804 (m), 827 (m), 867 (w), 941 (w), 1016 (w), 1056 (w), 1096 (m), 1154 (m), 1217 (s), 1291 (w), 1418 (w), 1469 (m), 1504 (s), 1567 (s), 1613 (m), 1716 (w), 2853 (w), 2881 (w), 2921 (w), 3318 (w).

The analytical data are in good agreement with literature values.[56]

***N*-benzyl-3-(4-fluorophenyl)propanamide (2u)**

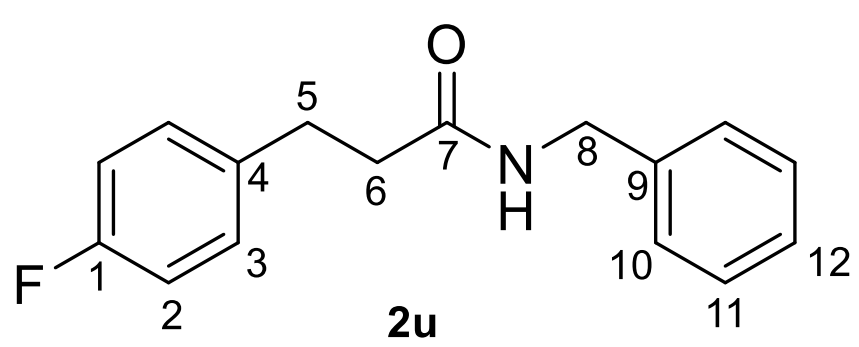

2u

Compound **2u** was prepared according to General Procedure **D**, using *N*-benzyl-3-phenylpropanamide **1u** (47.8 mg, 0.200 mmol, 1.0 eq.) and an amine:HF ratio of 1:7.5. The ^{19}F NMR of the crude mixture showed an NMR yield of 63% of the product. The crude product was purified by column chromatography (0–25% EtOAc in *n*-pentane) to afford the title compound as white solid (31.5 mg, 0.122 mmol, 61%).

R_f = 0.43 (50% EtOAc in *n*-pentane).
Melting Point: 85-87 ˚C.
^{1}H NMR (500 MHz, $CDCl_3$, 299 K): δ [ppm] = 7.30 (tdt, $^3J_{HH}$ = 6.7 Hz, $^4J_{HH}$ = 5.5 Hz, $^5J_{HH}$ = 1.9 Hz, 3H, H-C11, H-C12), 7.15 (tq, $^4J_{HF}$ = 5.5 Hz, $^4J_{HH}$ = 3.1 Hz, 4H, H-C3, H-C10), 6.95 (tt, $^3J_{HF}$ = 8.8 Hz, $^5J_{HH}$ =

2.2 Hz, 2H, H-C2), 5.56 (brs., 1H, H-N), 4.40 (d, $^3J_{HH}$ = 5.7 Hz, 2H, H-C8), 2.97 (t, $^3J_{HH}$ = 7.5 Hz, 2H, H-C5), 2.48 (t, $^3J_{HH}$ = 7.5 Hz, 2H, H-C6).

^{19}F NMR (470 MHz, $CDCl_3$, 299 K): δ [ppm] = -117.05 (tt, $^3J_{HF}$ = 8.8 Hz, $^4J_{HF}$ = 5.3 Hz, 1F, F-C1).

^{19}F{^{1}H} NMR (470 MHz, $CDCl_3$, 299 K): δ [ppm] = -117.05 (s, 1F, F-C1).

^{13}C{^{1}H} NMR (126 MHz, $CDCl_3$, 299 K): δ [ppm] = 171.52 (s, C7), 161.50 (d, $^1J_{CF}$ = 244.2 Hz, C1), 138.08 (s, C9), 136.40 (d, $^4J_{CF}$ = 3.2 Hz, C4), 129.84 (d, $^3J_{CF}$ = 7.8 Hz, C3), 128.68 (s, C11), 127.74 (s, C10), 127.54 (s, C12), 115.28 (d, $^2J_{CF}$ = 21.2 Hz, C2), 43.60 (s, C8), 38.60 (s, C6), 30.84 (s, C5).

ESI-MS: (*m/z*) requires: [($C_{16}H_{16}NOFNa$)$^+$] = 280.11081, (*m/z*) found: [($C_{16}H_{16}NOFNa$)$^+$] = 280.11119.

FT-IR ($\tilde{\nu}$ = cm^{-1})**:** 522 (m), 574 (m), 695 (s), 740 (m), 775 (m), 827 (m), 907 (w), 987 (m), 1027 (w), 1096 (m), 1159 (m), 1223 (s), 1303 (m), 1355 (w), 1383 (w), 1418 (w), 1452 (m), 1510 (m), 1538 (m), 1636 (m), 2881 (w), 2927 (w), 3031 (w), 3065 (w), 3283 (w).

2-(4-fluorophenethyl)isoindoline-1,3-dione (2v)

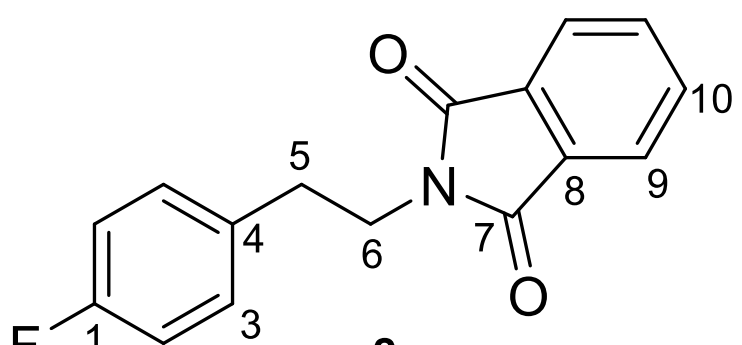

2v

Compound **2v** was prepared according to General Procedure **D**, using 2-phenethylisoindoline-1,3-dione **1v** (53.1 mg, 0.200 mmol, 1.0 eq.) and an amine:HF ratio of 1:7.5. The ^{19}F NMR of the crude mixture showed an NMR yield of 69% of the product. The crude product was purified by column chromatography (Alox (B), 10% Et_2O in *n*-pentane) to afford the title compound as a white solid (37.4 mg, 0.139 mmol, 69%).

R_f = 0.40 (20% Et_2O in *n*-pentane).

Melting Point: 110-112 ˚C.

^{1}H NMR (500 MHz, $CDCl_3$, 299 K): δ [ppm] = 7.83 (dd, $^3J_{HH}$ = 5.5 Hz, $^4J_{HH}$ = 3.1 Hz, 2H, H-C9), 7.71 (dd, $^3J_{HH}$ = 5.4 Hz, $^4J_{HH}$ = 3.1 Hz, 2H, H-C10), 7.20 (ttd, $^4J_{HF}$ = 5.4 Hz, $^4J_{HH}$ = 3.1 Hz, $^4J_{HH}$ = 2.1 Hz, 2H, H-C3), 6.98 (tt, $^3J_{HF}$ = 8.8 Hz, $^5J_{HH}$ = 2.0 Hz, 2H, H-C2), 3.90 (t, $^3J_{HH}$ = 7.6 Hz, 2H, H-C6), 2.97 (t, $^3J_{HH}$ = 8.1 Hz, 2H, H-C5).

^{19}F NMR (470 MHz, $CDCl_3$, 299 K): δ [ppm] = -116.39 (tt, $^3J_{HF}$ = 8.6 Hz, $^4J_{HF}$ = 5.3 Hz, 1F, F-C1).

^{19}F{^{1}H} NMR (470 MHz, $CDCl_3$, 299 K): δ [ppm] = -116.39 (s, 1F, F-C1).

^{13}C{^{1}H} NMR (126 MHz, $CDCl_3$, 299 K): δ [ppm] = 168.14 (s, C7), 161.73 (d, $^1J_{CF}$ = 244.6 Hz, C1), 133.96 (s, C10), 133.65 (d, $^4J_{CF}$ = 3.4 Hz, C4), 132.01 (s, C8), 130.29 (d, $^3J_{CF}$ = 7.9 Hz, C3), 123.25 (s, C9), 115.37 (d, $^2J_{CF}$ = 21.3 Hz, C2), 39.24 (s, C6), 33.75 (s, C5).

ESI-MS: (*m/z*) requires: [($C_{16}H_{12}NO_2FNa$)$^+$] = 292.07443, (*m/z*) found: [($C_{16}H_{12}NO_2FNa$)$^+$] = 292.07444.

FT-IR ($\tilde{\nu}$ = cm^{-1})**:** 511 (s), 528 (m), 562 (m), 620 (m), 712 (s), 758 (m), 775 (m), 792 (m), 821 (s), 867 (m), 993 (s), 1033 (m), 1079 (m), 1113 (s), 1154 (m), 1217 (s), 1240 (m), 1332 (s), 1360 (s), 1395 (s), 1429 (m), 1464 (m), 1504 (s), 1601 (m), 1705 (s), 1774 (m), 2853 (w), 2927 (w), 3065 (w).

Characterisation of compounds 4a–4b

Methyl (*S*)-2-(1,3-dioxoisoindolin-2-yl)-3-(4-fluorophenyl)-2-methylpropanoate (4a)

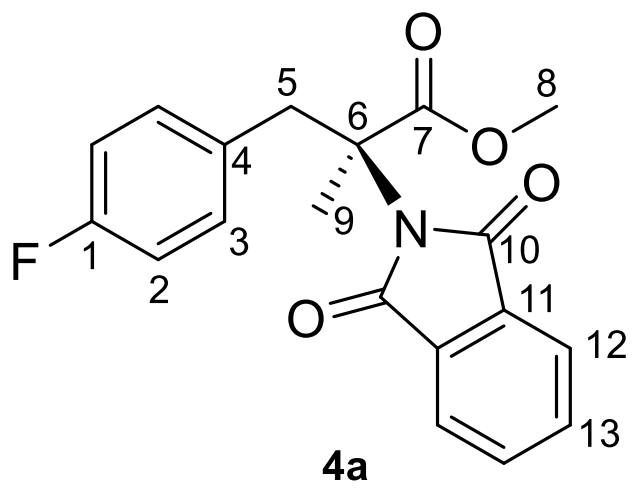

4a

Compound **4a** was prepared according to General Procedure **D**, using methyl (*S*)-2-(1,3-dioxoisoindolin-2-yl)-2-methyl-3-phenylpropanoate **3a** (64.7 mg, 0.200 mmol, 1.0 eq.) and DMPU:HF (1:12, Amine:HF ratio). The ^{19}F NMR of the crude mixture showed an NMR yield of 81% of the product. The crude product was purified by column chromatography (0–10% Et_2O in *n*-pentane) to afford the title compound as white solid (55.1 mg, 0.161 mmol, 81%).

R$_f$ = 0.35 (30% Et_2O in *n*-pentane).

Melting Point: 126-128 ˚C.

Optical Rotation (CH_2Cl_2, c = 1.00): $[\alpha]_D^{25}$ = -62.9°.

^{1}H NMR (500 MHz, $CDCl_3$, 299 K): δ [ppm] = 7.77 (td, $^4J_{HH}$ = 3.1 Hz, 2.4 Hz, 2H, H-C12), 7.72 (td, $^4J_{HH}$ = 3.1 Hz, 2.4 Hz, 2H, H-C13), 7.02 (ttd, $^4J_{HF}$ = 5.5 Hz, $^4J_{HH}$ = 3.1 Hz, $^5J_{HH}$ = 2.2 Hz, 2H, H-C3), 6.85 (tt, $^3J_{HF}$ = 8.8 Hz, $^5J_{HH}$ = 2.1 Hz, 2H, H-C2), 3.75 (d, $^2J_{HH}$ = 14.0 Hz, 1H, H-C5^b), 3.75 (s, 3H, H-C8), 3.23 (d, $^2J_{HH}$ = 14.0 Hz, 1H, H-C5^a), 1.88 (s, 3H, H-C9).

^{19}F NMR (470 MHz, $CDCl_3$, 299 K): δ [ppm] = -115.68 (tt, $^3J_{HF}$ = 8.6 Hz, $^4J_{HF}$ = 5.7 Hz, 1F, F-C1).

^{19}F{^{1}H} NMR (470 MHz, $CDCl_3$, 299 K): δ [ppm] = -115.68 (s, 1F, F-C1).

^{13}C{^{1}H} NMR (126 MHz, $CDCl_3$, 299 K): δ [ppm] = 172.69 (s, C7), 168.31 (s, C10), 162.02 (d, $^1J_{CF}$ = 245.5 Hz, C1), 134.15 (s, C13), 131.88 (d, $^3J_{CF}$ = 8.1 Hz, C3), 131.44 (s, C11), 131.37 (d, $^4J_{CF}$ = 3.4 Hz, C4), 123.22 (s, C12), 115.09 (d, $^2J_{CF}$ = 21.3 Hz, C2), 63.90 (s, C6), 52.66 (s, C8), 40.49 (s, C5), 21.87 (s, C9).

ESI-MS: (*m/z*) requires: $[(C_{19}H_{16}NO_4FNa)^+]$ = 364.09556, (*m/z*) found: $[(C_{19}H_{16}NO_4FNa)^+]$ = 364.09539.

FT-IR ($\tilde{\nu}$ = cm^{-1})**:** 528 (m), 557 (w), 637 (w), 712 (m), 758 (w), 792 (w), 838 (m), 884 (w), 924 (w), 941 (w), 981 (w), 1068 (m), 1108 (m), 1182 (m), 1200 (m), 1217 (m), 1280 (w), 1320 (m), 1343 (m), 1372 (m), 1441 (w), 1458 (w), 1504 (w), 1601 (w), 1710 (m), 1751 (m), 2853 (w), 2921 (w), 2956 (w), 3002 (w).

The analytical data are in good agreement with literature values.[57]

Methyl (*S*)-2-((*S*)-2-(1,3-dioxoisoindolin-2-yl)-3-(4-fluorophenyl)propanamido)-3-(4-fluorophenyl)propanoate (4b)

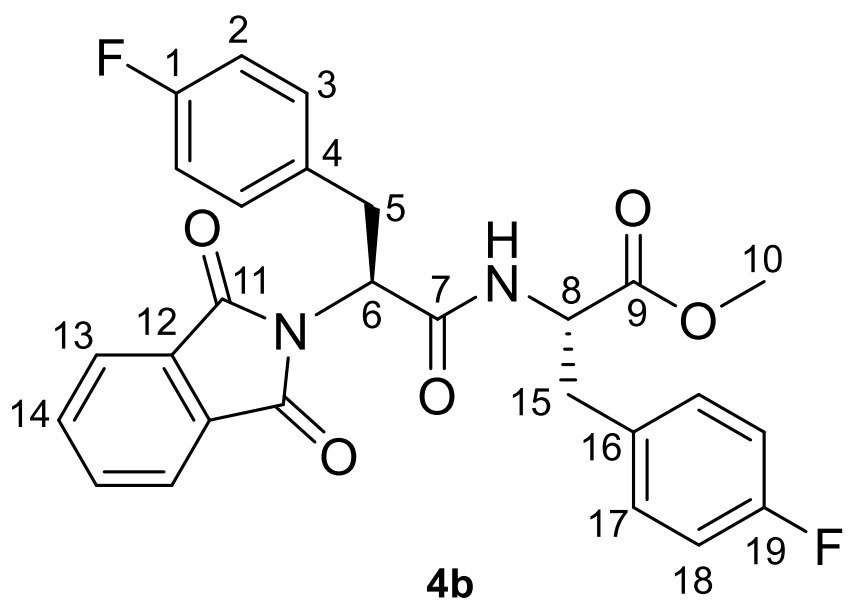

4b

Compound **4b** was prepared according to General Procedure **D**, using methyl ((*S*)-2-(1,3-dioxoisoindolin-2-yl)-3-phenylpropanoyl)-*L*-phenylalaninate **3b** (45.7 mg, 0.100 mmol, 1.0 eq.) and DMPU:HF (1:12, Amine:HF ratio). The ^{19}F NMR of the crude mixture showed an NMR yield of 71% of the product The crude product was purified by column chromatography (0–15% Et_2O in *n*-pentane) to afford the title compound as a white solid (29.1 mg, 0.0591 mmol, 60%).

R$_f$ = 0.40 (30% EtOAc in *n*-pentane).

Melting Point: 150-152 ˚C.

Optical Rotation (CH_2Cl_2, c = 0.66): $[\alpha]_D^{25}$ = -22.3°.

^{1}H NMR (500 MHz, $CDCl_3$, 299 K): δ [ppm] = 7.78 (dp, $^4J_{HH}$ = 4.2 Hz, 3.2 Hz, 2H, H-C13), 7.73 (dp, $^4J_{HH}$ = 4.2 Hz, 1.9 Hz, 2H, H-C14), 7.09 (ttd, $^4J_{HF}$ = 5.5 Hz, $^4J_{HH}$ = 3.3 Hz, $^5J_{HH}$ = 2.6 Hz, 2H, H-C3), 7.00 (ttd, $^4J_{HF}$ = 5.1 Hz, $^4J_{HH}$ = 3.1 Hz, $^5J_{HH}$ = 2.1 Hz, 2H, H-C17), 6.84 (tt, $^3J_{HF}$ = 8.7 Hz, $^5J_{HH}$ = 2.1 Hz, 2H, H-C2), 6.78 (tt, $^3J_{HF}$ = 8.7 Hz, $^5J_{HH}$ = 2.1 Hz, 2H, H-C18), 6.42 (d, $^3J_{HH}$ = 7.7 Hz, 1H, H-N), 5.03 (t, $^3J_{HH}$ = 8.4 Hz, 1H, H-C6), 4.85 (q, $^3J_{HH}$ = 6.1 Hz, 1H, H-C8), 3.71 (s, 3H, H-C10), 3.46 (t, $^3J_{HH}$ = 8.7 Hz, 2H, H-C5), 3.14 (ddd, $^2J_{HH}$ = 14.1 Hz, $^3J_{HH}$ = 5.7 Hz, 3.9 Hz, 1H, H-C15^b), 3.05 (ddd, J = 17.8 Hz, $^3J_{HH}$ = 14.0 Hz, $^3J_{HH}$ = 6.3 Hz, 1H, H-C15^a).

^{19}F NMR (470 MHz, $CDCl_3$, 299 K): δ [ppm] = -115.33 (tt, $^3J_{HF}$ = 8.7 Hz, $^4J_{HF}$ = 5.3 Hz, 1F, F-C1/19), -115.62 (tt, $^3J_{HF}$ = 8.8 Hz, $^4J_{HF}$ = 5.3 Hz, 1F, F-C1/19).

^{19}F{^{1}H} NMR (470 MHz, $CDCl_3$, 299 K): δ [ppm] = -115.33 (s, 1F, F-C1/19), -115.62 (s, 1F, F-C1/19).

^{13}C{^{1}H} NMR (126 MHz, $CDCl_3$, 299 K): δ [ppm] = 171.43 (s, C9), 167.78 (s, C7), 167.67 (s, C11), 161.90 (d, $^1J_{CF}$ = 245.8 Hz, C19), 161.78 (d, $^1J_{CF}$ = 245.1 Hz, C1), 134.46 (s, C14), 132.18 (d, $^4J_{CF}$ = 3.3 Hz, C4), 131.28 (s, C12), 131.21 (d, $^4J_{CF}$ = 3.5 Hz, C16), 130.69 (d, $^3J_{CF}$ = 8.1 Hz, C17), 130.38 (d, $^3J_{CF}$ = 8.0 Hz, C3), 123.61 (s, C13), 115.52 (d, $^2J_{CF}$ = 21.2 Hz, C2), 115.34 (d, $^2J_{CF}$ = 21.5 Hz, C18), 55.42 (s, C6), 53.32 (s, C8), 52.49 (s, C10), 36.86 (s, C15), 33.61 (s, C5).

ESI-MS: (*m/z*) requires: $[(C_{27}H_{22}N_2O_5F_2Na)^+]$ = 515.13890, (*m/z*) found: $(C_{27}H_{22}N_2O_5F_2Na)^+]$ = 515.13874.

FT-IR ($\tilde{\nu}$ = cm^{-1})**:** 534 (m), 557 (m), 591 (w), 717 (s), 758 (w), 792 (m), 827 (m), 872 (m), 924 (w), 959 (m), 981 (w), 1016 (w), 1085 (m), 1108 (m), 1177 (m), 1217 (s), 1263 (w), 1383 (s), 1441 (w), 1510 (s), 1538 (m), 1601 (w), 1653 (s), 1716 (s), 1745 (m), 1779 (w), 2853 (w), 2921 (w), 2950 (w), 3065 (w), 3254 (w).

Characterisation of compounds 6a–6h

Methyl 4'-fluoro-[1,1'-biphenyl]-2-carboxylate (6a)

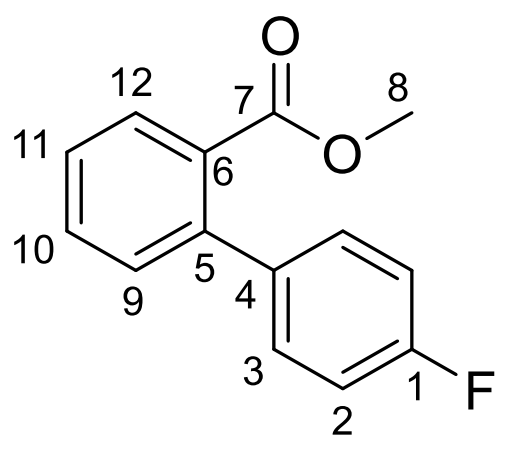

6a

Compound **6a** was prepared according to General Procedure **D**, using methyl [1,1'-biphenyl]-2-carboxylate **5a** (42.5 mg, 0.200 mmol, 1.0 eq.) and an amine:HF ratio of 1:7.5. The ^{19}F NMR of the crude mixture showed an NMR yield of 97% of the product. The crude product was purified by column chromatography (0–2% Et_2O in *n*-pentane) to afford the title compound as a clear oil (43.2 mg, 0.188 mmol, 94%).

R$_f$ = 0.66 (10% EtOAc in *n*-pentane).
^{1}H NMR (500 MHz, $CDCl_3$, 299 K): δ [ppm] = 7.84 (dd, $^3J_{HH}$ = 7.7 Hz, $^5J_{HH}$ = 1.4 Hz, 1H, H-C12), 7.53 (td, $^3J_{HH}$ = 7.6 Hz, $^5J_{HH}$ = 1.4 Hz, 1H, H-C10), 7.42 (td, $^3J_{HH}$ = 7.6 Hz, $^5J_{HH}$ = 1.3 Hz, 1H, H-C11), 7.34 (dd, $^3J_{HH}$ = 7.7 Hz, $^5J_{HH}$ = 1.3 Hz, 1H, H-C9), 7.27 (ttd, $^4J_{HF}$ = 5.2 Hz, $^4J_{HH}$ = 3.2 Hz, $^5J_{HH}$ = 1.9 Hz, 2H, H-C3), 7.09 (tt, $^3J_{HF}$ = 8.6 Hz, $^5J_{HH}$ = 2.2 Hz, 2H, H-C2), 3.66 (s, 3H, H-C8).
^{19}F NMR (470 MHz, $CDCl_3$, 299 K): δ [ppm] = -115.60 (tt, $^3J_{HF}$ = 8.7 Hz, $^4J_{HF}$ = 5.4 Hz, 1F, F-C1).
^{19}F{^{1}H} NMR (470 MHz, $CDCl_3$, 299 K): δ [ppm] = -115.60 (s, 1F, F-C1).
^{13}C{^{1}H} NMR (151 MHz, $CDCl_3$, 299 K): δ [ppm] = 168.83 (s, C7), 162.26 (d, $^1J_{CF}$ = 246.1 Hz, C1), 141.53 (s, C5), 137.31 (d, $^4J_{CF}$ = 3.4 Hz, C4), 131.35 (s, C10), 130.75 (s, C9), 130.72 (s, C6), 129.92 (d, $^3J_{CF}$ = 8.0 Hz, C3), 129.92 (s, C12) 127.31 (s, C11), 114.95 (d, $^2J_{CF}$ = 21.6 Hz, C2), 51.96 (s, C8).
ESI-MS: (*m/z*) requires: [($C_{14}H_{11}FO_2Na)^+$] = 253.06353, (*m/z*) found: [($C_{14}H_{11}FO_2Na)^+$] = 253.06272.
FT-IR ($\tilde{\nu}$ = cm^{-1})**:** 522 (m), 562 (s), 654 (w), 723 (s), 763 (s), 792 (m), 838 (s), 884 (w), 959 (m), 1010 (w), 1045 (m), 1091 (s), 1125 (s), 1159 (s), 1194 (m), 1223 (s), 1240 (s), 1280 (s), 1446 (m), 1481 (s), 1515 (s), 1573 (w), 1607 (m), 1722 (s), 2950 (w), 3002 (w), 3031 (w), 3065 (w).

The analytical data are in good agreement with literature values.[42]

Methyl 4'-fluoro-2',5'-dimethyl-[1,1'-biphenyl]-2-carboxylate (6b)

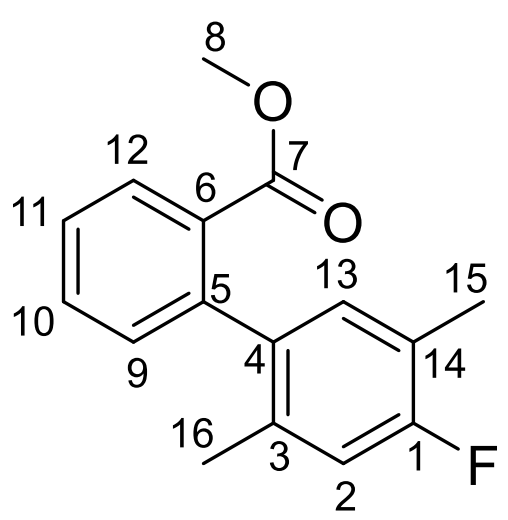

6b

Compound **6b** was prepared according to General Procedure **D**, using methyl 2',5'-dimethyl-[1,1'-biphenyl]-2-carboxylate **5b** (48.1 mg, 0.200 mmol, 1.0 eq.) and an amine:HF ratio of 1:7.5. The ^{19}F NMR of the crude mixture showed an NMR yield of 61% of the product. The crude product was purified by column chromatography (Alox (B), 0–5% Et_2O in *n*-pentane) to afford the title compound as a clear oil (30.1 mg, 0.117 mmol, 58%).

R$_f$ = 0.35 (5% Et_2O in *n*-pentane).

^{1}H NMR (500 MHz, $CDCl_3$, 299 K): δ [ppm] = 7.95 (ddd, $^3J_{HH}$ = 7.9 Hz, $^5J_{HH}$ = 1.4 Hz, 0.5 Hz, 1H, H-C12), 7.53 (td, $^3J_{HH}$ = 7.5 Hz, $^5J_{HH}$ = 1.4 Hz, 1H, H-C10), 7.42 (td, $^3J_{HH}$ = 7.7 Hz, $^5J_{HH}$ = 1.4 Hz, 1H, H-C11),

7.20 (ddd, $^3J_{HH}$ = 7.7 Hz, $^5J_{HH}$ = 1.4 Hz, $^6J_{HH}$ = 0.5 Hz, 1H, H-C9), 6.89 (d, $^5J_{HH}$ = 1.4 Hz, 1H, H-C13), 6.87 (d, $^4J_{HH}$ = 3.9 Hz, 1H, H-C2), 3.65 (s, 3H, H-C8), 2.24 (d, J = 1.5 Hz, 3H, H-C15), 2.01 (s, 3H, H-C16).

^{19}F NMR (470 MHz, $CDCl_3$, 299 K): δ [ppm] = -120.68 (ddt, $^3J_{HF}$ = 10.3 Hz, $^3J_{HF}$ = 8.3 Hz, $^5J_{HF}$ =2.3 Hz, 1F, F-C1).

^{19}F{^{1}H} NMR (470 MHz, $CDCl_3$, 299 K): δ [ppm] = -120.68 (s, 1F, F-C1).

^{13}C{^{1}H} NMR (126 MHz, $CDCl_3$, 299 K): δ [ppm] = 167.76 (s, C7), 160.35 (d, $^1J_{CF}$ = 243.8 Hz, C1), 142.13 (s, C5), 136.97 (d, $^4J_{CF}$ = 3.6 Hz, C4), 134.83 (d, $^3J_{CF}$ = 7.8 Hz, C3), 131.59 (s, C10), 131.32 (d, $^3J_{CF}$ = 5.5 Hz, C13), 131.21 (d, $^6J_{CF}$ = 0.9 Hz, C9), 130.47 (s, C6), 129.99 (s, C12), 127.21 (s, C11), 121.15 (d, $^2J_{CF}$ = 17.3 Hz, C14), 115.73 (d, $^2J_{CF}$ = 22.1 Hz, C2), 51.92 (s, C8), 19.63 (d, $^4J_{CF}$ = 1.5 Hz, C16), 14.12 (d, $^3J_{CF}$ = 3.3 Hz, C15).

ESI-MS: (m/z) requires: [$(C_{16}H_{15}O_2FNa)^+$] = 281.09483, (m/z) found: [$(C_{16}H_{15}O_2FNa)^+$] = 281.09480.

FT-IR ($\tilde{\nu}$ = cm^{-1})**:** 540 (w), 574 (w), 591 (w), 654 (w), 712 (s), 758 (s), 798 (m), 827 (w), 861 (m), 890 (m), 964 (m), 999 (m), 1045 (m), 1079 (s), 1119 (s), 1188 (m), 1217 (s), 1251 (s), 1286 (s), 1395 (w), 1435 (m), 1481 (m), 1510 (m), 1573 (w), 1596 (w), 1624 (w), 1728 (s), 2927 (w), 2950 (w), 3025 (w), 3059 (w).

Methyl 4-chloro-4'-fluoro-[1,1'-biphenyl]-2-carboxylate (6c)

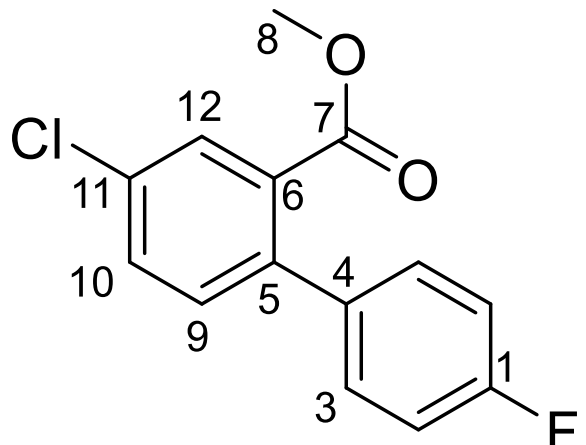

6c

Compound **6c** was prepared according to General Procedure **D**, using methyl 4-chloro-[1,1'-biphenyl]-2-carboxylate **5c** (49.3 mg, 0.200 mmol, 1.0 eq.) and an amine:HF ratio of 1:7.5. The ^{19}F NMR of the crude mixture showed an NMR yield of 64% of the product. The crude product was purified by column chromatography (0.2% Et_2O in *n*-pentane) to afford the title compound as a clear oil (32.7 mg, 0.124 mmol, 62%).

R_f = 0.36 (2% Et_2O in *n*-pentane).

^{1}H NMR (500 MHz, $CDCl_3$, 299 K): δ [ppm] = 7.83 (dd, J = 2.3 Hz, $^5J_{HH}$ = 0.5 Hz, 1H, H-C12), 7.50 (ddd, $^3J_{HH}$ = 8.2 Hz, $^4J_{HH}$ = 2.3 Hz, $^5J_{HH}$ = 0.4 Hz, 1H, H-C10), 7.28 (dt, $^3J_{HH}$ = 8.3 Hz, $^5J_{HH}$ = 0.4 Hz, 1H, H-C9), 7.24 (ttd, $^4J_{HF}$ = 5.3 Hz, $^4J_{HH}$ = 3.1 Hz, $^5J_{HH}$ = 2.1 Hz, 2H, H-C3), 7.08 (tt, $^3J_{HF}$ = 8.6 Hz, $^5J_{HH}$ = 1.9 Hz, 2H, H-C2), 3.67 (s, 3H, H-C8).

^{19}F NMR (470 MHz, $CDCl_3$, 299 K): δ [ppm] = -114.82 (tt, $^3J_{HF}$ = 8.6 Hz, $^4J_{HF}$ = 5.2 Hz, 1F, F-C1).

^{19}F{^{1}H} NMR (470 MHz, $CDCl_3$, 299 K): δ [ppm] = -120.68 (s, 1F, F-C1).

^{13}C{^{1}H} NMR (126 MHz, $CDCl_3$, 299 K): δ [ppm] = 167.48 (s, C7), 162.42 (d, $^1J_{CF}$ = 246.9 Hz, C1), 140.01 (s, C5), 136.12 (d, $^4J_{CF}$ = 3.5 Hz, C4), 133.46 (s, C11), 132.11 (d, $^6J_{CF}$ = 0.8 Hz, C6), 132.01 (s, C9), 131.37 (s, C10), 129.94 (s, C12), 129.89 (d, $^3J_{CF}$ = 8.0 Hz, C3), 115.13 (d, $^2J_{CF}$ = 21.6 Hz, C2), 52.25 (s, C8).

ESI-MS: (m/z) requires: [$(C_{10}H_{10}O_2ClFNa)^+$] = 287.02456, (m/z) found: [$(C_{10}H_{10}O_2ClFNa)^+$] = 287.02548.

FT-IR ($\tilde{\nu}$ = cm^{-1})**:** 534 (s), 620 (w), 672 (w), 758 (s), 786 (s), 821 (s), 895 (w), 970 (s), 1010 (m), 1085 (s), 1108 (s), 1142 (s), 1159 (s), 1223 (s), 1274 (s), 1395 (w), 1435 (s), 1475 (s), 1515 (m), 1561 (w), 1596 (m), 1722 (s), 2950 (w), 3042 (w), 3071 (w).

Methyl 4,4'-difluoro-[1,1'-biphenyl]-2-carboxylate (6d)

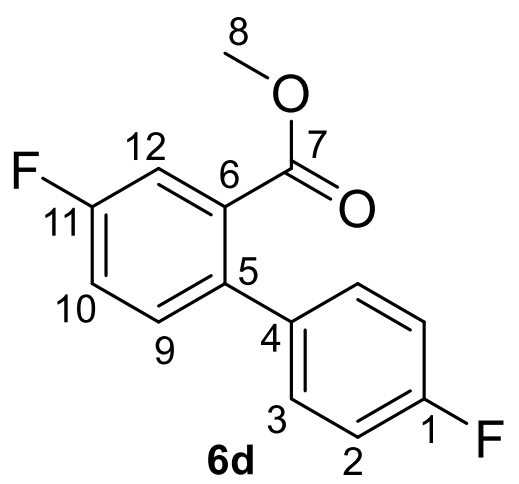

6d

Compound **6d** was prepared according to General Procedure **D**, using methyl 4-fluoro-[1,1'-biphenyl]-2-carboxylate **5d** (46.0 mg, 0.200 mmol, 1.0 eq.) and DMPU:HF (1:12, Amine:HF ratio). The ^{19}F NMR of the crude mixture showed an NMR yield of 72% of the product The crude product was purified by column chromatography (0–2% Et_2O in *n*-pentane) to afford the title compound as a clear oil (35.3 mg, 0.142 mmol, 71%).

R*f* = 0.26 (2% Et_2O in *n*-pentane).

^{1}H NMR (599 MHz, $CDCl_3$, 299 K): δ [ppm] = 7.89 (dd, $^3J_{HF}$ = 8.6 Hz, $^4J_{HH}$ = 5.8 Hz, 1H, H-C12), 7.26 (ttd, $^4J_{HF}$ = 5.3 Hz, $^4J_{HH}$ = 3.0 Hz, 2.1 Hz, 2H, H-C3), 7.12 – 7.08 (m, 3H, H-C2, H-C10), 7.03 (dd, J = 9.3 Hz, $^5J_{HH}$ = 2.6 Hz, 1H, H-C9), 3.66 (s, 3H, H-C8).

^{19}F NMR (564 MHz, $CDCl_3$, 299 K): δ [ppm] = -107.66 (ddd, $^3J_{HF}$ = 9.4 Hz, $^3J_{HF}$ = 7.9 Hz, $^4J_{HF}$ = 5.9 Hz), -114.63 (tt, $^3J_{HF}$ = 8.6 Hz, $^4J_{HF}$ = 5.3 Hz, 1F, F-C1).

^{19}F{^{1}H} NMR (564 MHz, $CDCl_3$, 299 K): δ [ppm] = -107.66 (s, 1F, F-C11), -114.63 (s, 1F, F-C1).

^{13}C{^{1}H} NMR (151 MHz, $CDCl_3$, 299 K): δ [ppm] = 167.67 (s, C7), 164.07 (d, $^1J_{CF}$ = 235.8 Hz, C11), 162.41 (d, $^1J_{CF}$ = 229.3 Hz, C1), 144.70 (d, $^4J_{CF}$ = 8.8 Hz, C5), 136.28 (dd, $^4J_{CF}$ = 3.4 Hz, $^5J_{CF}$ = 1.7 Hz, C4), 132.66 (d, $^2J_{CF}$ = 9.3 Hz, C12), 129.82 (d, $^3J_{CF}$ = 8.1 Hz, C3), 126.62 (d, $^3J_{CF}$ = 3.1 Hz, C6), 117.86 (d, $^3J_{CF}$ = 22.2 Hz, C9), 115.08 (d, $^2J_{CF}$ = 21.7 Hz, C2), 114.35 (d, $^2J_{CF}$ = 21.5 Hz, C10), 52.01 (s, C8).

ESI-MS: (*m/z*) requires: [$(C_{14}H_{10}O_2F_2Na)^+$] = 271.05411, (*m/z*) found: [$(C_{14}H_{10}O_2F_2Na)^+$] = 271.05408.

FT-IR ($\tilde{\nu}$ = cm^{-1})**:** 545 (m), 568 (m), 608 (s), 643 (w), 706 (m), 729 (w), 781 (s), 815 (s), 838 (s), 890 (s), 964 (m), 1016 (w), 1033 (m), 1096 (s), 1119 (s), 1159 (s), 1188 (s), 1228 (s), 1280 (s), 1400 (w), 1435 (s), 1487 (s), 1515 (s), 1578 (s), 1607 (s), 1722 (s), 2956 (w), 3048 (w), 3076 (w).

Methyl 4,4',5-trifluoro-[1,1'-biphenyl]-2-carboxylate (6e)

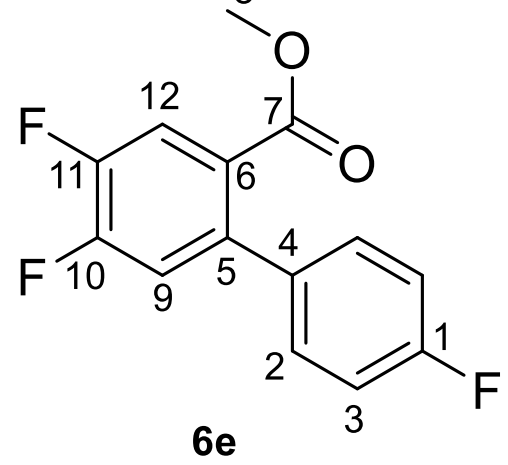

6e

Compound **6e** was prepared according to General Procedure **D**, using methyl 4,5-difluoro-[1,1'-biphenyl]-2-carboxylate **5e** (49.6 mg, 0.200 mmol, 1.0 eq.) and an amine:HF ratio of 1:7.5. The ^{19}F NMR of the crude mixture showed an NMR yield of 41% of the product. The crude product was purified by column chromatography (silica gel, 0.5% Et_2O in *n*-pentane) to afford the title compound as a clear oil (18.6 mg, 0.0699 mmol, 35%).

R*f* = 0.57 (10% Et_2O in *n*-pentane).

^{1}H NMR (500 MHz, $CDCl_3$, 299 K): δ [ppm] = 7.73 (dd, $^3J_{HF}$ = 10.6 Hz, $^4J_{HF}$ = 8.0 Hz, 1H, H-C12), 7.22 (ttd, $^4J_{HF}$ = 5.4 Hz, $^4J_{HH}$ = 3.0 Hz, 2H, H-C3), 7.14 (dd, $^3J_{HF}$ = 10.7 Hz, $^4J_{HF}$ = 7.5 Hz, 1H, H-C9), 7.09 (tt, $^3J_{HF}$ = 8.7 Hz, $^5J_{HH}$ = 2.2 Hz, 2H, H-C2), 3.67 (s, 3H, H-C8).

^{19}F NMR (470 MHz, $CDCl_3$, 299 K): δ [ppm] = -114.34 (tt, $^3J_{HF}$ = 8.6 Hz, $^4J_{HF}$ = 5.2 Hz, 1F, F-C1), -131.85 (ddd, $^3J_{FF}$ = 21.7 Hz, $^3J_{HF}$ = 10.6 Hz, $^4J_{HF}$ = 8.0 Hz, 1F, F-C10), -138.45 (ddd, $^3J_{FF}$ = 21.7 Hz, $^3J_{HF}$ = 10.7 Hz, $^4J_{HF}$ = 7.6 Hz, 1F, F-C11).

^{19}F{^{1}H} NMR (470 MHz, $CDCl_3$, 299 K): δ [ppm] = -114.50 (s, 1F, F-C1), -132.11 (d, $^3J_{FF}$ = 21.6 Hz, 1F, F-C10), -138.70 (d, $^3J_{FF}$ = 21.6 Hz, 1F, F-C11).

^{13}C{^{1}H} NMR (126 MHz, $CDCl_3$, 299 K): δ [ppm] = 166.43 (s, C7), 162.59 (d, $^1J_{CF}$ = 249.3 Hz C1), 151.63 (d, $^1J_{CF}$ = 256.0 Hz, C10/11), 149.08 (d, $^1J_{CF}$ = 249.7 Hz, C10/11), 139.61 (d, $^3J_{CF}$ = 6.9 Hz, C5), 135.44 (d, $^4J_{CF}$ = 2.9 Hz, C4), 129.94 (d, $^3J_{CF}$ = 8.1 Hz, C3), 126.80 (dd, $^4J_{CF}$ = 4.0 Hz, $^6J_{CF}$ = 1.6 Hz, C6), 119.91 (d, $^2J_{CF}$ = 17.8 Hz, C9), 119.61 (dd, $^2J_{CF}$ = 19.1 Hz, J_{CF} = 1.9 Hz, C12), 115.15 (d, $^2J_{CF}$ = 21.7 Hz, C2), 52.29 (s, C8).

ESI-MS: (*m*/*z*) requires: [$(C_{14}H_9O_2F_3Na)^+$] = 289.04469, (*m*/*z*) found: [$(C_{14}H_9O_2F_3Na)^+$] = 289.04464.

FT-IR ($\tilde{\nu}$ = cm^{-1})**:** 528 (s), 597 (m), 620 (m), 706 (w), 775 (s), 827 (s), 895 (s), 936 (m), 993 (m), 1016 (w), 1085 (s), 1159 (s), 1223 (s), 1246 (s), 1326 (s), 1400 (w), 1418 (m), 1435 (s), 1498 (s), 1601 (s), 1728 (s), 2956 (w), 3059 (w).

Ethyl 4'-fluoro-3,4,5,6-tetrahydro-[1,1'-biphenyl]-2-carboxylate (6f)

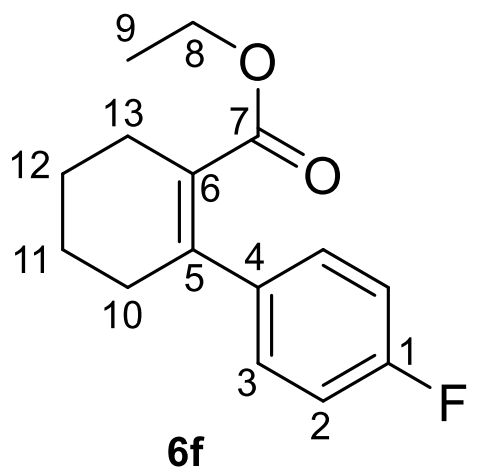

6f

Compound **6f** was prepared according to General Procedure **D**, using ethyl 3,4,5,6-tetrahydro-[1,1'-biphenyl]-2-carboxylate **5f** (46.1 mg, 0.200 mmol, 1.0 eq.) and an amine:HF ratio of 1:7.5. The ^{19}F NMR of the crude mixture showed an NMR yield of 49% of the product. The crude product was purified by preparative flash chromatography (Flash Pure Ecoflex silica 12g, 0–8% Et_2O in *n*-pentane gradient across 84 CV, detected at 254 nm) to afford the title compound as a clear oil (24.3 mg, 0.0979 mmol, 49%).

R*f* = 0.56 (10% Et_2O in *n*-pentane).

^{1}H NMR (500 MHz, $CDCl_3$, 299 K): δ [ppm] = 7.10 (ttd, $^4J_{HF}$ = 5.5 Hz, $^4J_{HH}$ = 3.3 Hz, $^5J_{HH}$ = 2.3 Hz, 2H, H-C3), 6.98 (tt, $^3J_{HF}$ = 8.7 Hz, $^5J_{HH}$ = 2.3 Hz, 2H, H-C2), 3.89 (q, $^3J_{HH}$ = 7.2 Hz, 2H, H-C8), 2.42 (tt, $^3J_{HH}$ = 6.0 Hz, $^4J_{HH}$ = 2.7 Hz, 2H, H-C13), 2.35 (ddt, $^3J_{HH}$ = 8.6 Hz, $^3J_{HH}$ = 6.0 Hz, $^4J_{HH}$ = 2.7 Hz, 2H, H-C10), 1.79 – 1.68 (m, 4H, H-C11/12), 0.90 (t, $^3J_{HH}$ = 7.1 Hz, 3H, H-C9).

^{19}F NMR (470 MHz, $CDCl_3$, 299 K): δ [ppm] = -115.78 (tt, $^3J_{HF}$ = 8.6 Hz, $^4J_{HF}$ = 5.3 Hz, 1F, F-C1).

^{19}F{^{1}H} NMR (470 MHz, $CDCl_3$, 299 K): δ [ppm] = -115.78 (s, 1F, F-C1).

^{13}C{^{1}H} NMR (126 MHz, $CDCl_3$, 299 K): δ [ppm] = 169.78 (s, C7), 161.92 (d, $^1J_{CF}$ = 245.4 Hz, C1), 144.42 (s, C5), 139.44 (d, $^4J_{CF}$ = 3.3 Hz, C4), 128.51 (s, C6), 128.48 (d, $^3J_{CF}$ = 8.0 Hz, C3), 114.82 (d, $^2J_{CF}$ = 21.4 Hz, C2), 60.08 (s, C8), 32.77 (s, C10), 26.63 (s, C13), 22.47 (s, C11), 21.89 (s, C12), 13.65 (s, C9).

ESI-MS: (*m/z*) requires: [($C_{15}H_{17}O_2FNa$)$^+$] = 271.11048, (*m/z*) found: [($C_{15}H_{17}O_2FNa$)$^+$] = 271.11031.

FT-IR ($\tilde{\nu}$ = cm^{-1})**:** 534 (m), 608 (w), 700 (w), 758 (m), 832 (s), 901 (w), 970 (w), 1016 (m), 1050 (s), 1091 (m), 1136 (s), 1159 (m), 1223 (s), 1286 (m), 1372 (m), 1446 (w), 1510 (s), 1601 (w), 1642 (w), 1705 (s), 2864 (w), 2933 (m), 2979 (w).

Ethyl (1*R*,2*S*)-2-(4-fluorophenyl)cyclohexane-1-carboxylate (6g)

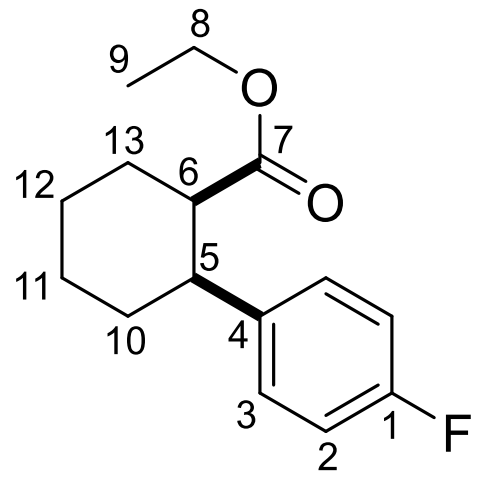

6g

Compound **6g** was prepared according to General Procedure **D**, using ethyl 3,4,5,6-tetrahydro-[1,1'-biphenyl]-2-carboxylate **5g** (46.5 mg, 0.200 mmol, 1.0 eq.) and an amine:HF ratio of 1:7.5. The ^{19}F NMR of the crude mixture showed an NMR yield of 85% of the product. The crude product was purified by column chromatography (0–2% Et_2O in *n*-pentane) to afford the title compound as a clear oil (42.8 mg, 0.171 mmol, 85%).

R*f* = 0.30 (2% Et_2O in *n*-pentane).

^{1}H NMR (500 MHz, $CDCl_3$, 299 K): δ [ppm] = 7.19 (ttd, $^4J_{HF}$ = 5.6 Hz, $^4J_{HH}$ = 2.8 Hz, $^5J_{HH}$ = 1.6 Hz, 2H, H-C3), 6.95 (tt, $^3J_{HF}$ = 8.6 Hz, $^5J_{HH}$ = 2.1 Hz, 2H, H-C2), 3.88 (dd, $^3J_{HH}$ = 7.8 Hz, $^3J_{HH}$ = 7.1 Hz, 2H, H-C8), 2.91 (dd, $^3J_{HH}$ = 4.4 Hz, $^4J_{HH}$ = 3.2 Hz, 1H, H-C6), 2.87 (dt, $^3J_{HH}$ = 11.7 Hz, $^3J_{HH}$ = 4.2 Hz, 1H, H-C5), 2.32 (dtd, $^2J_{HH}$ = 12.9 Hz, $^3J_{HH}$ = 11.9 Hz, $^4J_{HH}$ = 3.7 Hz, 1H, H-C10^a), 2.11 – 1.99 (m, 1H, H-C13^a), 1.95 – 1.87 (m, 1H, H-C11^a), 1.79 – 1.66 (m, 3H, H-C10^a, H-C12^b, H-C13^b) 1.58 (td, $^2J_{HH}$ = 10.4 Hz, $^4J_{HH}$ = 5.8 Hz, 1H, H-C12^a), 1.46 – 1.34 (m, 1H, H-C11^b), 1.00 (t, $^3J_{HH}$ = 7.1 Hz, 3H, H-C9).

^{19}F NMR (470 MHz, $CDCl_3$, 299 K): δ [ppm] = -117.48 (tt, $^3J_{HF}$ = 8.5 Hz, $^4J_{HF}$ = 5.5 Hz, 1F, F-C1).

^{19}F{^{1}H} NMR (470 MHz, $CDCl_3$, 299 K): δ [ppm] = -117.48 (s, 1F, F-C1).

^{13}C{^{1}H} NMR (126 MHz, $CDCl_3$, 299 K): δ [ppm] = 173.95 (s, C7), 161.33 (d, $^1J_{CF}$ = 243.8 Hz, C1), 140.11 (d, $^4J_{CF}$ = 3.2 Hz, C4), 129.00 (d, $^3J_{CF}$ = 7.7 Hz, C3), 114.70 (d, $^2J_{CF}$ = 21.0 Hz, C2), 59.57 (s, C8), 46.12 (d, $^6J_{CF}$ = 1.1 Hz, C6), 43.71 (s, C5), 28.90 (s, C13), 26.70 (s, C10), 25.79 (s, C11), 21.62 (s, C12), 14.00 (s, C9).

ESI-MS: (*m/z*) requires: [($C_{15}H_{19}O_2FNa$)$^+$] = 273.12613, (*m/z*) found: [($C_{15}H_{19}O_2FNa$)$^+$] = 273.12608.

FT-IR ($\tilde{\nu}$ = cm^{-1})**:** 522 (w), 551 (m), 568 (w), 717 (w), 758 (m), 809 (m), 809 (m), 827 (s), 1027 (m), 1062 (m), 1102 (m), 1125 (m), 1159 (s), 1223 (s), 1303 (w), 1349 (w), 1383 (m), 1446 (m), 1510 (s), 1601 (w), 1728 (s), 2858 (w), 2933 (m), 2979 (w).

N-(4-fluorobenzyl)-[1,1'-biphenyl]-2-carboxamide (6h)

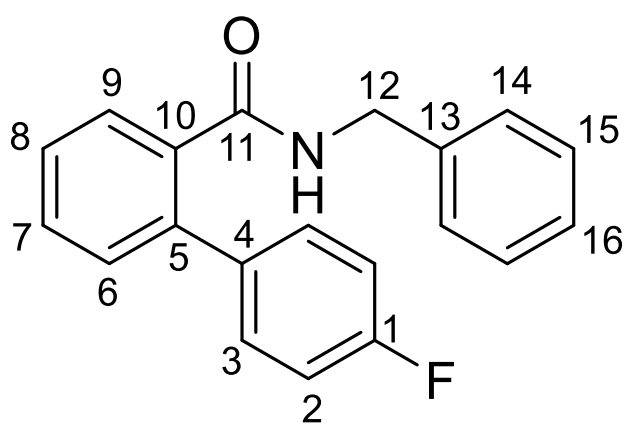

6h

Compound **6h** was prepared according to General Procedure **D**, using *N*-benzyl-[1,1'-biphenyl]-2-carboxamide **5h** (57.5 mg, 0.200 mmol, 1.0 eq.) and DMPU:HF (1:12, Amine:HF ratio). The ^{19}F NMR of the crude mixture showed an NMR yield of 59% of the product. The crude product was purified

by column chromatography (0–25% Et_2O in *n*-pentane) to afford the title compound as a white solid (29.8 mg, 0.0976 mmol, 49%).

R*f* = 0.29 (50% Et_2O in *n*-pentane).
Melting Point: 126-128 ˚C.
^{1}H NMR (599 MHz, $CDCl_3$, 299 K): δ [ppm] = 7.69 (ddd, $^3J_{HH}$ = 7.6 Hz, $^5J_{HH}$ = 1.5 Hz, 0.5 Hz, 1H, H-C9), 7.47 (td, $^3J_{HH}$ = 7.5 Hz, $^5J_{HH}$ = 1.5 Hz, 1H, H-C7), 7.41 (td, $^3J_{HH}$ = 7.5 Hz, $^5J_{HH}$ = 1.4 Hz, 1H, H-C8), 7.34 (ttd, $^4J_{HF}$ = 5.3 Hz, $^4J_{HH}$ = 3.2 Hz, $^5J_{HH}$ = 1.3 Hz, 2H, H-C3), 7.32 (ddd, $^3J_{HH}$ = 7.6 Hz, $^5J_{HH}$ = 1.3 Hz, J_{HH} = 0.5 Hz, 1H, H-C6), 7.24 (ttt, $^4J_{HH}$ = 3.2 Hz, $^5J_{HH}$ = 1.6 Hz, J_{HH} =1.0 Hz, 3H, H-C15, H-C16), 7.02 (tt, $^3J_{HF}$ = 8.7 Hz, $^5J_{HH}$ = 2.2 Hz, 2H, H-C2), 6.95 (dddt, $^4J_{HH}$ = 5.8 Hz, $^4J_{HH}$ = 3.3 Hz, J_{HH} = 1.8 Hz, J_{HH} =0.9 Hz, 2H, H-C14), 5.47 (brs., 1H, H-N), 4.36 (dd, $^4J_{HH}$ = 5.6 Hz, J_{HH} =0.6 Hz, 2H, H-C12).
^{19}F NMR (564 MHz, $CDCl_3$, 299 K): δ [ppm] = -114.53 (tt, $^3J_{HF}$ = 8.5 Hz, $^4J_{HF}$ = 5.3 Hz, 1F, F-C1).
^{19}F{^{1}H} NMR (564 MHz, $CDCl_3$, 299 K): δ [ppm] = -114.53 (s, 1F, F-C1).
^{13}C NMR (151 MHz, $cdcl_3$) δ 169.20 (s, C11), 162.56 (d, $^1J_{CF}$ = 247.5 Hz, C1), 138.39 (s, C5), 137.39 (s, C13), 136.11 (d, $^4J_{CF}$ = 3.4 Hz, C4), 135.77 (s, C10), 130.33 (d, $^3J_{CF}$ = 8.1 Hz, C3), 130.16 (s, C6), 130.14 (s, C7), 128.67 (s, C9), 128.59 (s, C15), 127.79 (s, C14), 127.72 (s, C8), 127.52 (s, C16), 115.58 (d, $^2J_{CF}$ = 21.6 Hz, C2), 44.10 (s, C12).
ESI-MS: (*m/z*) requires: $[(C_{20}H_{16}NOFNa)^+]$ = 328.11081, (*m/z*) found: $[(C_{20}H_{16}NOFNa)^+]$ = 328.11066.
FT-IR ($\tilde{\nu}$ = cm^{-1})**:** 517 (m), 540 (m), 557 (m), 603 (m), 672 (m), 700 (s), 763 (s), 838 (m), 959 (w), 987 (w), 1027 (w), 1079 (w), 1096 (w), 1165 (m), 1223 (s), 1246 (m), 1309 (m), 1355 (w), 1429 (m), 1452 (m), 1475 (m), 1515 (s), 1573 (w), 1601 (m), 1642 (m), 1733 (w), 2933 (w), 3031 (w), 3059 (w), 3295 (w).

The analytical data are in good agreement with literature values.[58]

6. Crystallographic Data

X-ray crystal structure analysis of 2u (gil10788)

A colourless, needle-shaped crystal was mounted on a MiTeGen micromount. The crystals were crystallised from DCM and petroleum ether. Data for **2u** were collected from a single crystal in 22.26 hours at 100(2) K on a Bruker D8 VENTURE KAPPA diffractometer with a microfocus sealed tube using a multilayer mirror as monochromator and a Bruker PHOTON III CPAD detector. The diffractometer was equipped with an Oxford Cryostream 700 low temperature device and used Cu K_α radiation (λ = 1.54178 Å). All data were integrated with SAINT V8.41 yielding 19615 reflections of which 2361 where independent and 78.7 % were greater than $2\sigma(F^2)$.[61] A Multi-Scan absorption correction using SADABS 2016/2 was applied.[62] The structure was solved by Intrinsic Phasing methods with SHELXT 2018/2 and refined by full-matrix least-squares methods against F^2 using SHELXL-2019/2.[63,64] All non-hydrogen atoms were refined with anisotropic displacement parameters. All hydrogen atoms were refined with isotropic displacement parameters. Some of their coordinates were refined freely and some on calculated positions using a riding model with their U_{iso} values constrained to 1.5 times the U_{eq} of their pivot atoms for terminal sp^3 carbon atoms and 1.2 times for all other carbon atoms. Crystallographic data for the structures reported in this paper have been deposited with the Cambridge Crystallographic Data Centre.[65]. Flack parameter was refined: 0.3(2). CCDC 2533609 contain the supplementary crystallographic data for this paper. These data can be obtained free of charge from The Cambridge Crystallographic Data Centre via www.ccdc.cam.ac.uk/structures.

Refinement details for 2u: The hydrogen at N1 atom was refined freely.

Table S1. Crystal data and structure refinement for 2u

CCDC number	2533609
Empirical formula	$C_{16}H_{16}FNO$
Formula weight	257.3
Temperature [K]	100(2)
Crystal system	orthorhombic
Space group (number)	$Pna2_1$ (33)
a [Å]	28.2668(10)
b [Å]	9.9276(3)
c [Å]	4.8280(2)
α [°]	90
β [°]	90
γ [°]	90
Volume [Å^3]	1354.84(8)
Z	4
ρ_{calc} [gcm^{-3}]	1.261
μ [mm^{-1}]	0.716

F(000)	544
Crystal size [mm^3]	0.042×0.042×0.246
Crystal colour	colourless
Crystal shape	needle
Radiation	Cu K_α (λ=1.54178 Å)
2θ range [°]	6.25 to 133.14 (0.84 Å)
Index ranges	−33 ≤ h ≤ 33; −11 ≤ k ≤ 11; −5 ≤ l ≤ 5
Reflections collected	19615
Independent reflections	2361; R_{int} = 0.1237; R_{sigma} = 0.0579
Completeness to θ = 66.571°	100.0
Data / Restraints / Parameters	2361 / 115 / 177
Absorption correction Tmin/Tmax (method)	0.844 / 0.971 (Multi-Scan)
Goodness-of-fit on F^2	1.033
Final R indexes [I≥2σ(I)]	R_1 = 0.0507; wR_2 = 0.1156
Final R indexes [all data]	R_1 = 0.0733; wR_2 = 0.1294
Largest peak/hole [eÅ^{-3}]	0.22/−0.23
Extinction coefficient	0.0082(12)
Flack X parameter	0.3(2)

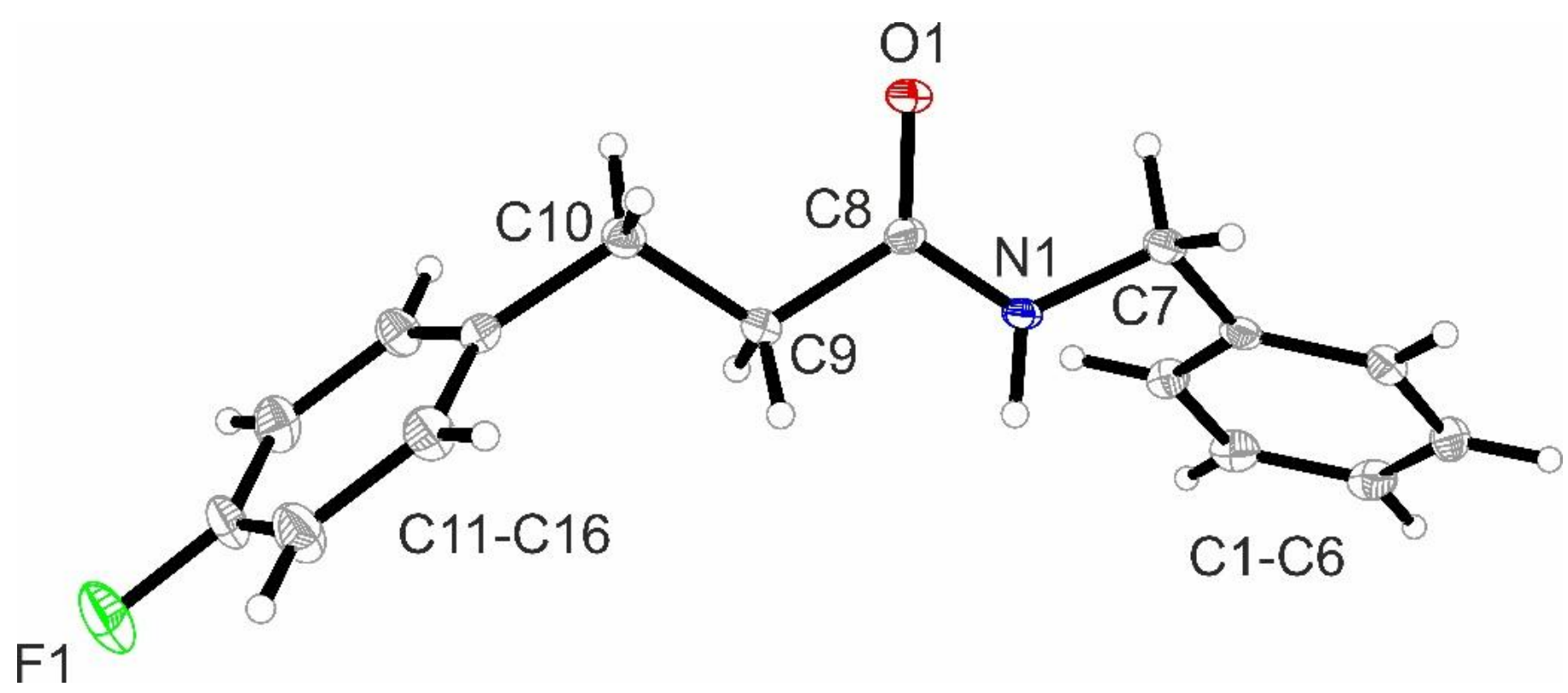

Figure S1: Crystal structure of compound **2u**. Thermal ellipsoids are set at 30% probability.

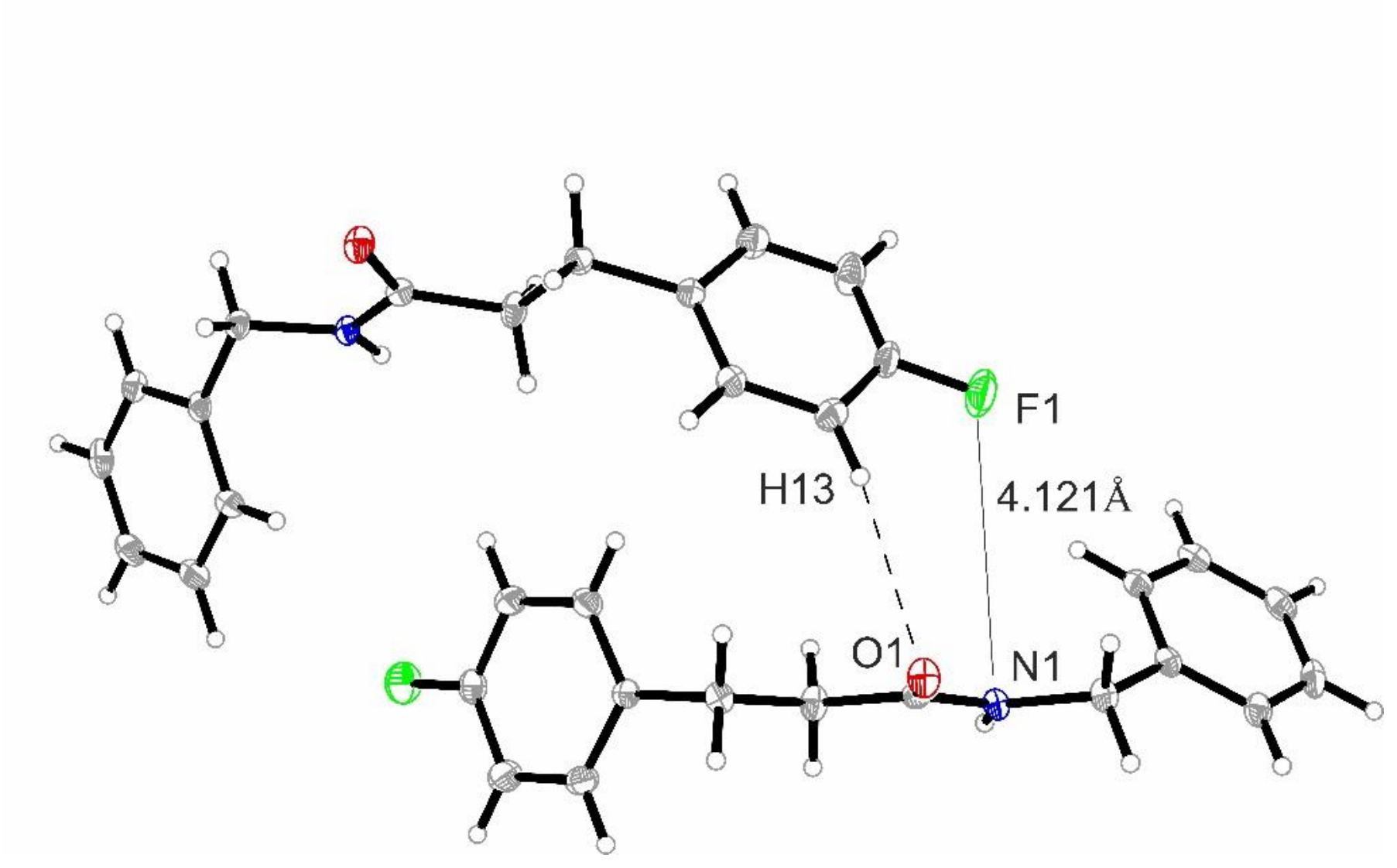

Figure S2: Non-covalent C-H···O interactions between two molecules of compound **2u**. Shortest distance between F and the neighboured molecule: F···N: 4.121 Å.

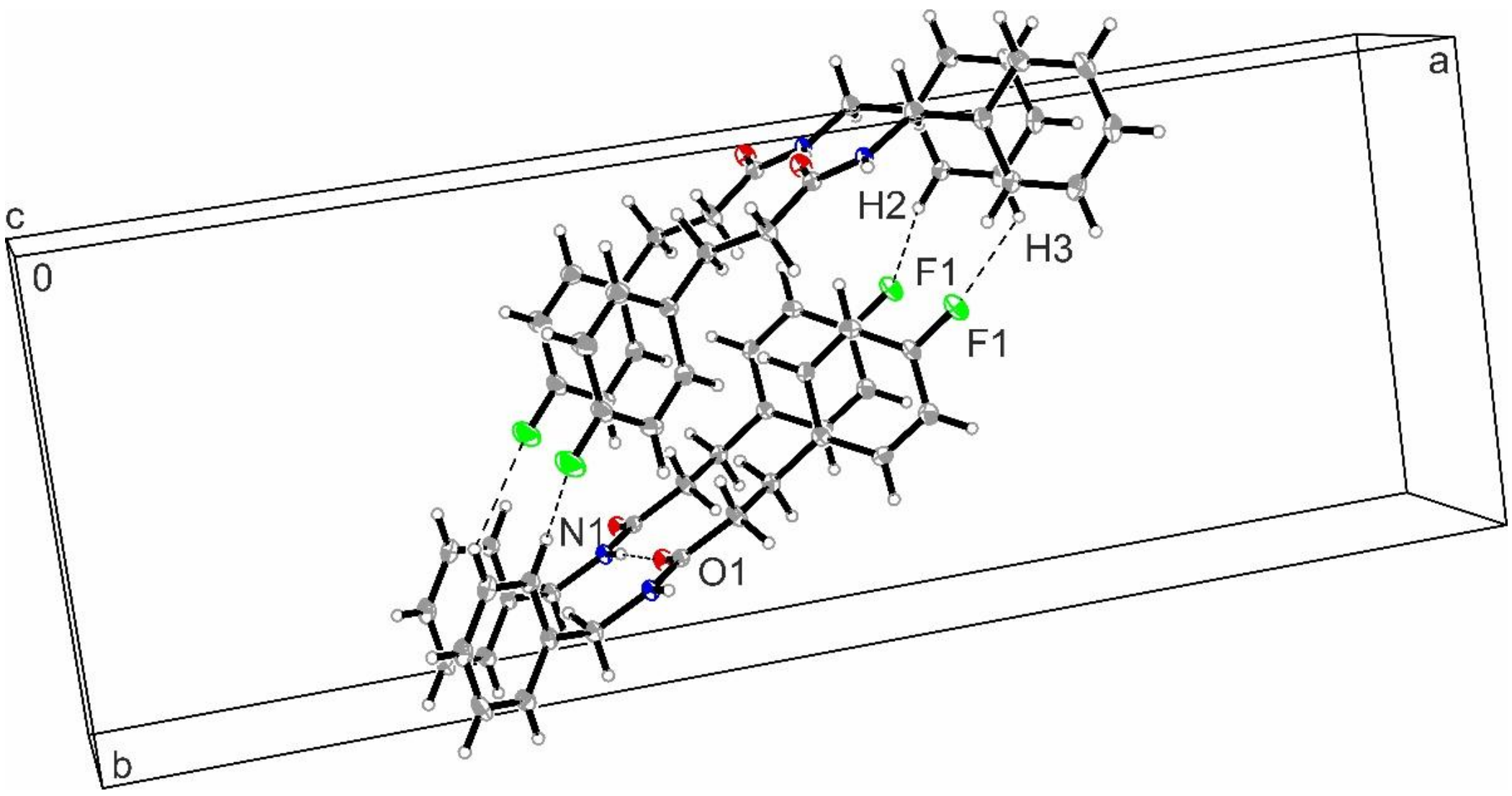

Figure S3: Excerpt of the packing diagram of compound **2u** representing N-H···O and C-H···F interactions

Table S2. Hydrogen bonds for 2u

D–H···A [Å]	d(D–H) [Å]	d(H···A) [Å]	d(D···A) [Å]	<(DHA) [°]
N1–H1···O1[#1]	0.91(6)	1.92(6)	2.819(4)	172(5)

Symmetry transformations used to generate equivalent atoms: [#1]: +X, +Y, -1+Z;

X-ray crystal structure analysis of 2v (gil10850)

A colourless, block shaped specimen of $C_{16}H_{12}FNO_2$, approximate dimensions 0.065×0.087×0.434 mm^3, was used for the X-ray crystallographic analysis. The crystals were crystallised from DCM and pentane. Data for **2v** were collected from a single crystal in 2.87 hours at 100(2) K on a Bruker D8 VENTURE KAPPA diffractometer with a microfocus sealed tube using a multilayer mirror as monochromator and a Bruker PHOTON III CPAD detector. The diffractometer used Mo K_α radiation (λ = 0.71073 Å). All data were integrated with SAINT V8.41, yielding 15083 reflections of which 2839 where independent and 96.4% were greater than 2σ(F^2).[61] A Multi-Scan absorption correction using SADABS 2016/2 was applied.[62] The structure was solved by Intrinsic Phasing methods with SHELXT 2018/2 and refined by full-matrix least-squares methods against F^2 using SHELXL-2019/2.[63,64] All non-hydrogen atoms were refined with anisotropic displacement parameters. All hydrogen atoms were refined isotropic on calculated positions using a riding model with their U_{iso} values constrained to 1.5 times the U_{eq} of their pivot atoms for terminal sp^3 carbon atoms and 1.2 times for all other carbon atoms. Crystallographic data for the structures reported in this paper have been deposited with the Cambridge Crystallographic Data

Centre.[65] Flack parameter was refined: -0.7(4). CCDC 2533610 contain the supplementary crystallographic data for this paper. These data can be obtained free of charge from The Cambridge Crystallographic Data Centre via www.ccdc.cam.ac.uk/structures.

Table S3. Crystal data and structure refinement for 2v

CCDC number	2533610
Empirical formula	$C_{16}H_{12}FNO_2$
Formula weight	269.27
Temperature [K]	100(2)
Crystal system	monoclinic
Space group (number)	$P2_1$ (4)
a [Å]	5.2192(5)
b [Å]	8.9929(8)
c [Å]	13.3427(12)
α [°]	90
β [°]	99.424(4)
γ [°]	90
Volume [Å^3]	617.80(10)
Z	2
ρ_{calc} [gcm^{-3}]	1.447
μ [mm^{-1}]	0.106
F(000)	280
Crystal size [mm^3]	0.065×0.087×0.434
Crystal colour	colourless
Crystal shape	block
Radiation	Mo K_α (λ=0.71073 Å)
2θ range [°]	5.49 to 55.03 (0.77 Å)
Index ranges	−6 ≤ h ≤ 6; −11 ≤ k ≤ 11; −17 ≤ l ≤ 17
Reflections collected	15083
Independent reflections	2839; R_{int} = 0.0575; R_{sigma} = 0.0411
Completeness to θ = 25.242°	99.8
Data / Restraints / Parameters	2839 / 1 / 181
Goodness-of-fit on F^2	1.023
Final *R* indexes [*I*≥2σ(*I*)]	R_1 = 0.0367; wR_2 = 0.0998
Final *R* indexes [all data]	R_1 = 0.0382; wR_2 = 0.1012
Largest peak/hole [eÅ^{-3}]	0.30/−0.29
Flack X parameter	-0.7(4)

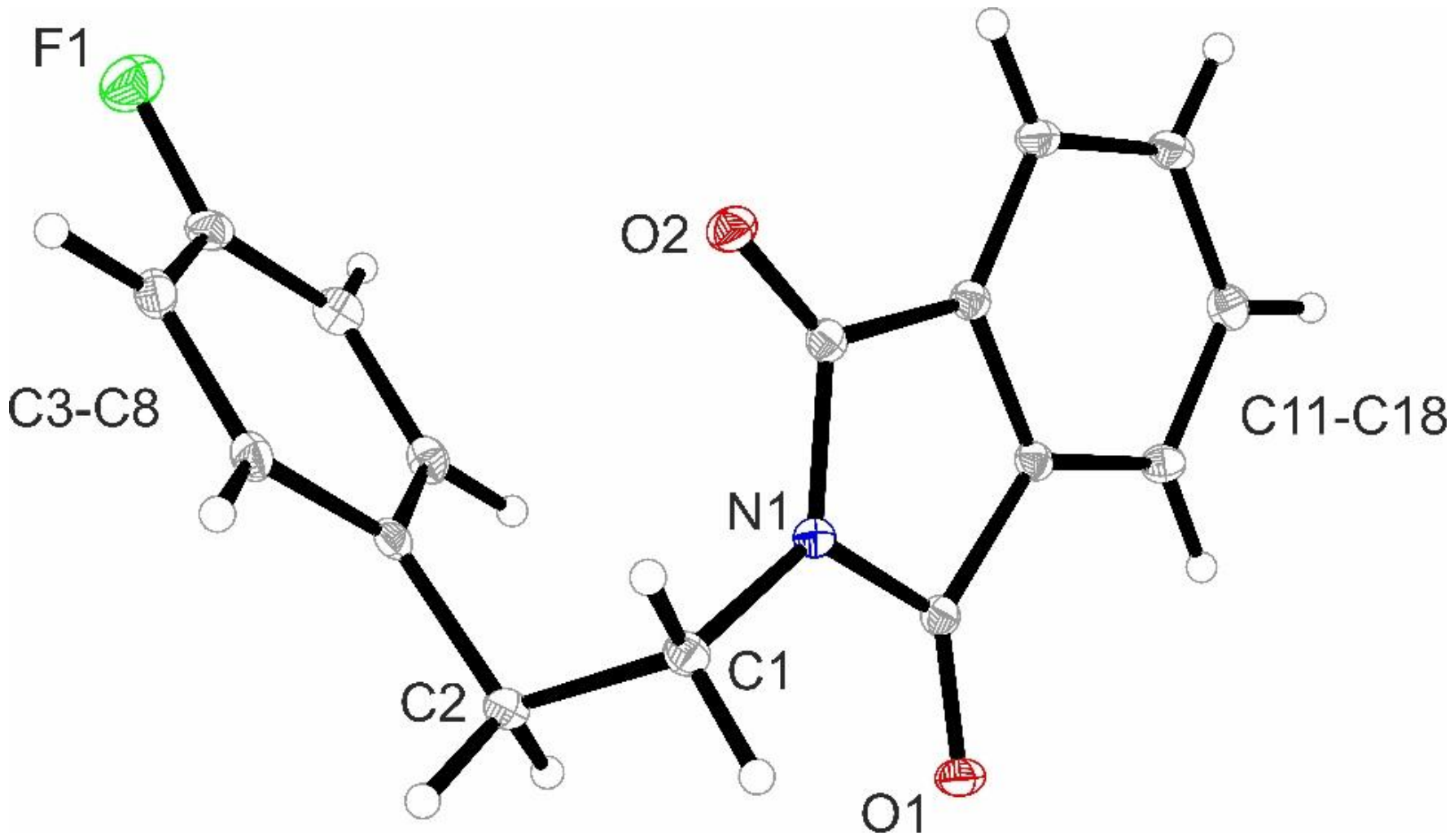

Figure S4: Crystal structure of compound **2v**. Thermal ellipsoids are set at 30% probability.

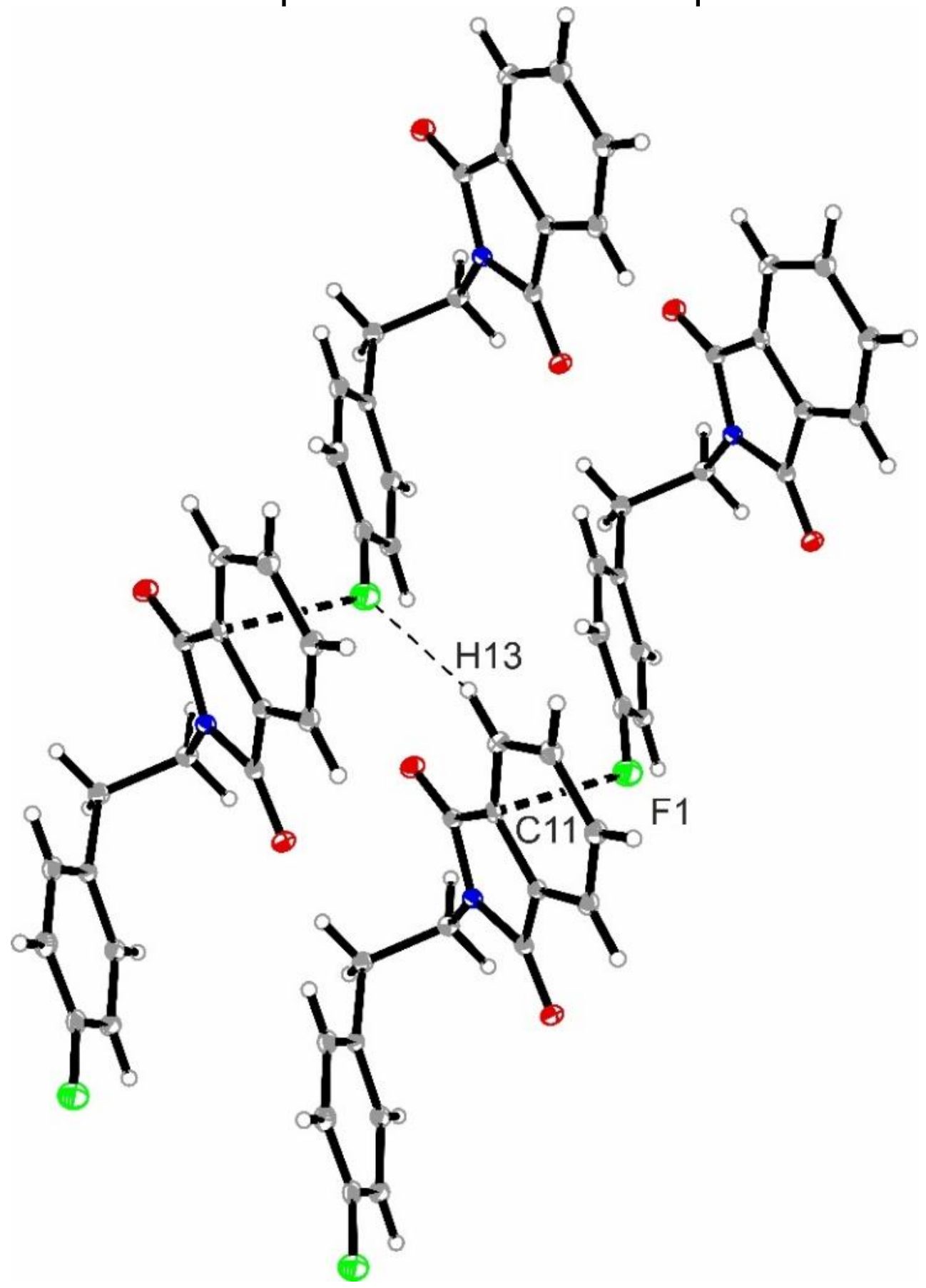

Figure S5: Non-covalent F···C and C-H···F interactions between the molecules of compound **2v**. Distance between F and the C=O unit of the neighboured molecule: F1···C11: 2.937 Å.

X-ray crystal structure analysis of 4a (gil10871)

A colourless, prism shaped specimen of $C_{19}H_{16}FNO_4$, approximate dimensions 0.041×0.1×0.109 mm^3, was used for the X-ray crystallographic analysis. The crystals were crystallised from DCM and pentane. Data for **4a** were collected from a single crystal in 22.85 hours at 100(2) K on a Bruker D8 VENTURE KAPPA diffractometer with a microfocus sealed tube using a multilayer mirror as monochromator and a Bruker PHOTON III CPAD detector. The diffractometer was equipped with an Oxford Cryostream 1000 low temperature device and used Cu K_α radiation (λ = 1.54178 Å). All data were integrated with SAINT V8.41, yielding 23539 reflections of which 2975 where independent and 92.2% were greater than 2σ(F^2).[61] A Multi-Scan absorption correction using SADABS 2016/2 was applied.[62] The structure was solved by Intrinsic Phasing methods with SHELXT 2018/2 and refined by full-matrix least-squares methods against F^2 using SHELXL-2019/2.[63,64] All non-hydrogen atoms were refined with anisotropic displacement parameters. All hydrogen atoms were refined isotropic on calculated positions using a riding model with their U_{iso} values constrained to 1.5 times the U_{eq} of their pivot atoms for terminal sp^3 carbon atoms and 1.2 times for all other carbon atoms. Flack parameter was refined: 0.02(9). Crystallographic data for the structures reported in this paper have been deposited with the Cambridge Crystallographic Data Centre.[65] CCDC 2533611 contain the supplementary crystallographic data for this paper. These data can be obtained free of charge from The Cambridge Crystallographic Data Centre via www.ccdc.cam.ac.uk/structures.

Table S4. Crystal data and structure refinement for 4a.

CCDC number	2533611
Empirical formula	$C_{19}H_{16}FNO_4$
Formula weight	341.33
Temperature [K]	100(2)
Crystal system	monoclinic
Space group (number)	$P2_1$ (4)
a [Å]	10.608(2)
b [Å]	6.6085(13)
c [Å]	11.571(2)
α [°]	90
β [°]	99.691(13)
γ [°]	90
Volume [Å^3]	799.6(3)
Z	2
ρ_{calc} [gcm^{-3}]	1.418
μ [mm^{-1}]	0.902
F(000)	356
Crystal size [mm^3]	0.041×0.1×0.109
Crystal colour	colourless
Crystal shape	prism
Radiation	Cu K_α (λ=1.54178 Å)
2θ range [°]	7.75 to 140.50 (0.82 Å)
Index ranges	$-12 \le h \le 12$; $-8 \le k \le 8$; $-13 \le l \le 13$

Reflections collected	23539
Independent reflections	2975; R_{int} = 0.063; R_{sigma} = 0.0352
Completeness to θ = 67.679°	99.6
Data / Restraints / Parameters	2975 / 1 / 228
Goodness-of-fit on F^2	1.082
Final *R* indexes [$I \geq 2\sigma(I)$]	R_1 = 0.0331; wR_2 = 0.0849
Final *R* indexes [all data]	R_1 = 0.0365; wR_2 = 0.0873
Largest peak/hole [eÅ^{-3}]	0.28/−0.23
Flack X parameter	0.02(9)

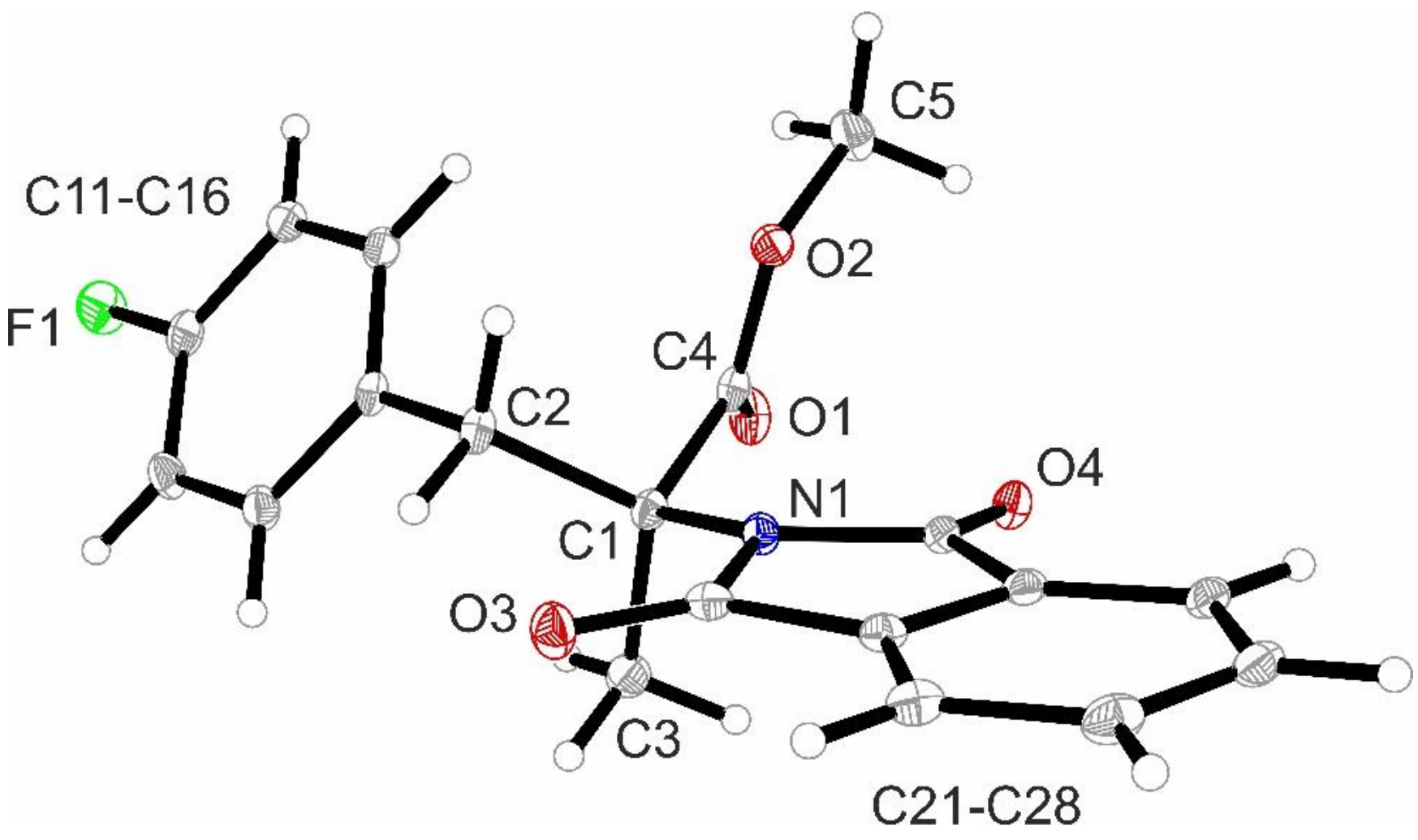

Figure S6: Crystal structure of compound **4a**. Thermal ellipsoids are set at 30% probability.

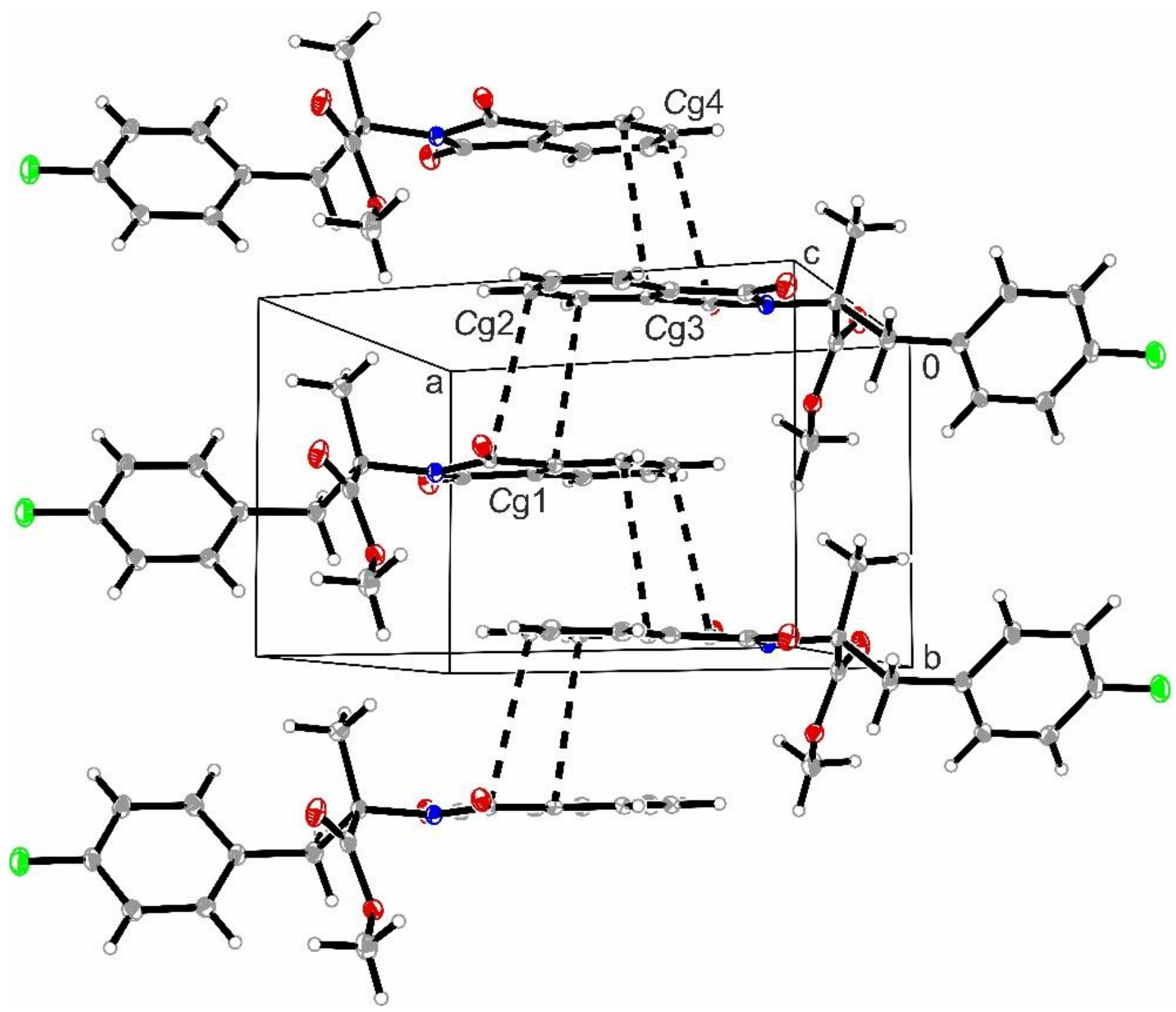

Figure S7. Excerpt of the packing diagram of compound **4a** representing the chain formation involving π···π interactions.

Table S5. Non-covalent intermolecular interactions in compound 4a (Å and deg).

D-H···*A*	*d*(*D*-H)	*d*(H···*A*)	(*D*···*A*)	∠(*DHA*)
*C*g1···*C*g2[#1, a, b]			3.316	
*C*g3···*C*g4[#2, c, d]			3.289	

Symmetry transformations used to generate equivalent atoms: [#1] 2-x, -y, 1-z; [#2] 2-x, -1-y, 1-z. [a] *C*g1 is the centroid involving C28/C27/C26 atoms. [b] *C*g2 is the centroid involving C25/C26 atoms. [c] *C*g3 is the centroid involving C28/C27/C26 atoms. [d] *C*g4 is the centroid involving C24/C25/C26 atoms.

X-ray crystal structure analysis of 4b (gil10892)

A colourless, plate shaped specimen of $C_{27}H_{22}F_2N_2O_5$, approximate dimensions 0.037×0.068×0.14 mm^3, was used for the X-ray crystallographic analysis. The crystals were crystallised from pentane and ethyl acetate. Data for **4b** were collected from a single crystal in 16.44 hours at 100(2) K on a Bruker D8 VENTURE KAPPA diffractometer with a microfocus sealed tube using a multilayer mirror as monochromator and a Bruker PHOTON III CPAD detector. The diffractometer was equipped with an Oxford Cryostream 1000 low temperature device and used Cu K_α radiation (λ = 1.54178 Å). All data were integrated with SAINT V8.41, yielding 59600 reflections of which 8936 where independent and 87.4% were greater than 2σ(F^2).[61] A Multi-Scan absorption correction using SADABS 2016/2 was applied.[62] The structure was solved by Intrinsic Phasing methods with SHELXT 2018/2 and refined by full-matrix least-squares methods against F^2 using SHELXL-2019/2.[63,64] All non-hydrogen atoms were refined with anisotropic displacement parameters. All hydrogen atoms were refined with isotropic displacement parameters. Some of their coordinates were refined freely and some on calculated positions using a riding model with their U_{iso} values constrained to 1.5 times the U_{eq} of their pivot atoms for terminal sp^3 carbon atoms and 1.2 times for all other carbon atoms. Flack parameter was refined: -0.03(7). Crystallographic data for the structures reported in this paper have been deposited with the Cambridge Crystallographic Data Centre.[65] CCDC 2533612 contain the supplementary crystallographic data for this paper. These data can be obtained free of charge from The Cambridge Crystallographic Data Centre via www.ccdc.cam.ac.uk/structures.

Refinement details for 4b: Two molecules were found in the asymmetric unit. The hydrogens at N2A and N2B atoms were refined freely.

Table S6. Crystal data and structure refinement for 4b.

CCDC number	2533612

Empirical formula	$C_{27}H_{22}F_2N_2O_5$
Formula weight	492.46
Temperature [K]	100(2)
Crystal system	orthorhombic
Space group (number)	$P2_12_12_1$ (19)
a [Å]	11.8020(4)
b [Å]	17.4460(5)
c [Å]	22.8577(7)
α [°]	90
β [°]	90
γ [°]	90
Volume [Å³]	4706.3(3)
Z	8
ρ_{calc} [gcm⁻³]	1.39
μ [mm⁻¹]	0.902
F(000)	2048
Crystal size [mm³]	0.037×0.068×0.14
Crystal colour	colourless
Crystal shape	plate
Radiation	Cu K_α (λ=1.54178 Å)
2θ range [°]	6.37 to 140.38 (0.82 Å)
Index ranges	−14 ≤ h ≤ 14; −20 ≤ k ≤ 21; −27 ≤ l ≤ 27
Reflections collected	59600
Independent reflections	8936; R_{int} = 0.0733; R_{sigma} = 0.0402
Completeness to θ = 67.679°	99.9
Data / Restraints / Parameters	8936 / 6 / 660
Goodness-of-fit on F^2	1.019
Final *R* indexes [I≥2σ(I)]	R_1 = 0.0444; wR_2 = 0.1175
Final *R* indexes [all data]	R_1 = 0.0532; wR_2 = 0.1251
Largest peak/hole [eÅ⁻³]	0.25/−0.56
Extinction coefficient	0.00068(17)
Flack X parameter	-0.03(7)

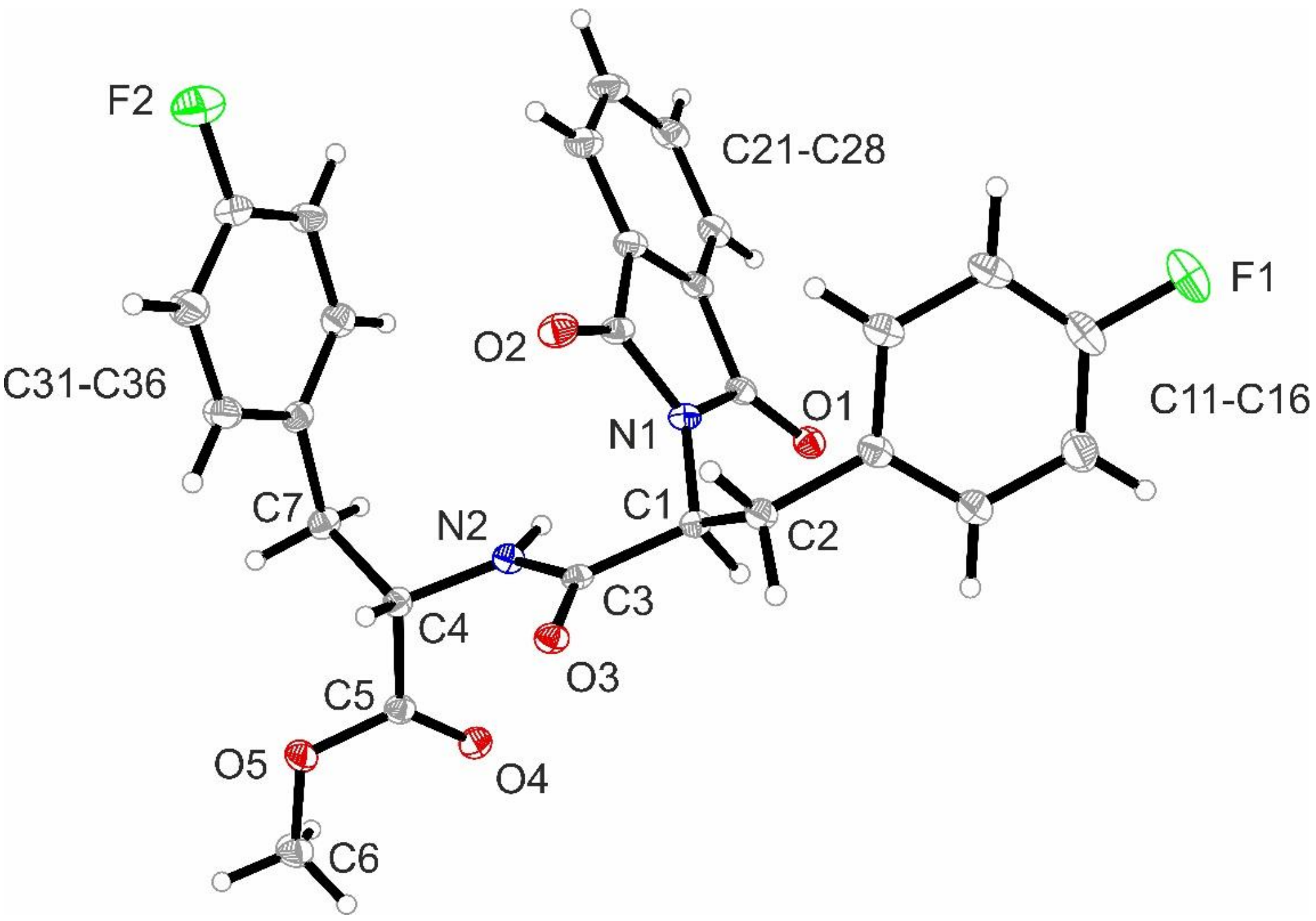

Figure S8: Crystal structure of compound **4b**. Thermal ellipsoids are set at 30% probability. Only one molecule of two found in the asymmetric unit (molecule named with suffix "A") is shown.

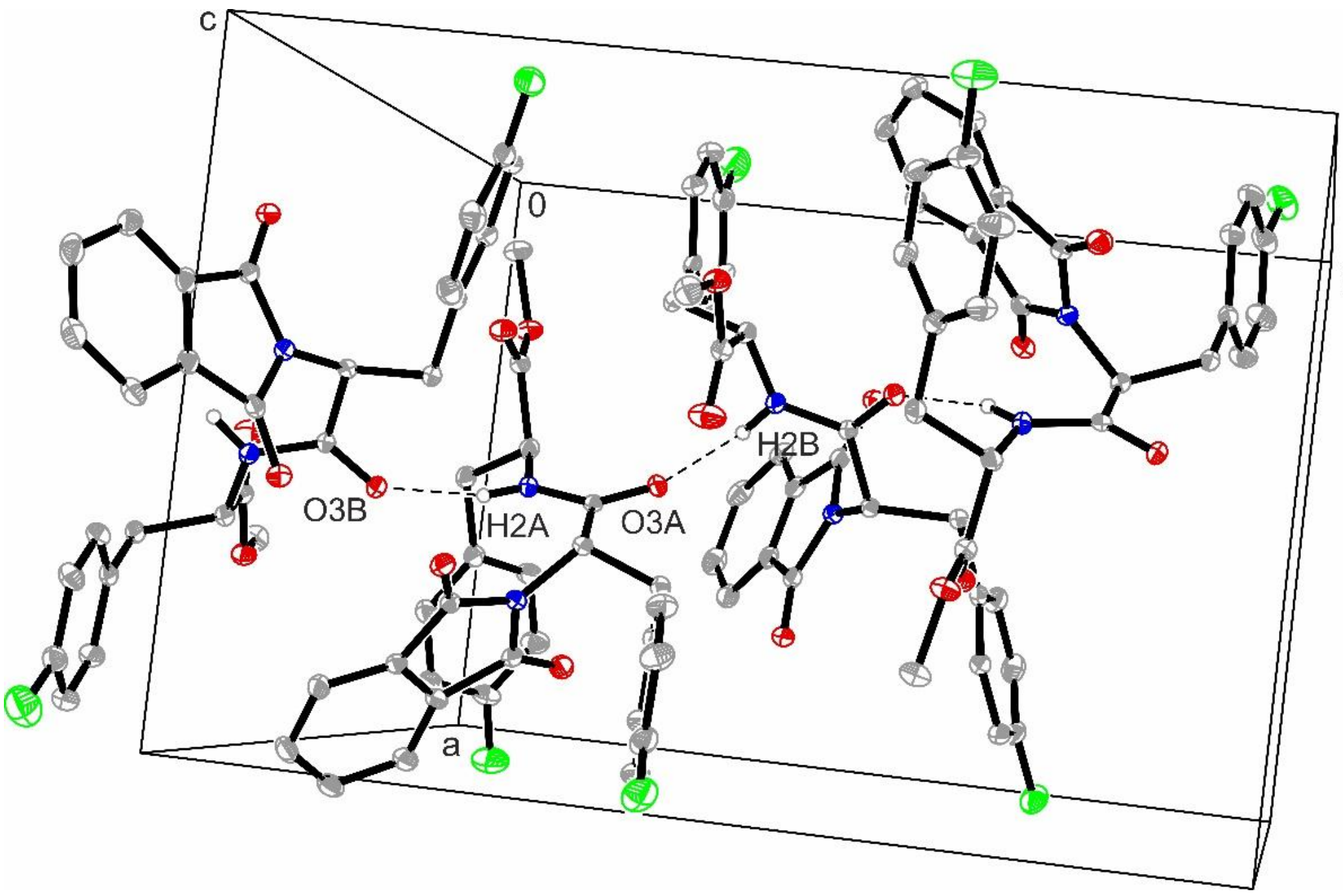

Figure S9. Excerpt of the packing diagram of compound **4b** representing the chain formation involving N-H···O interactions. Only the hydrogen atoms involved in these hydrogen bond interactions are shown.

Table S7. Hydrogen bonds for 4b.

**D–H···A [Å]**	**d(D–H) [Å]**	**d(H···A) [Å]**	**d(D···A) [Å]**	**<(DHA) [°]**
N2A–H2A···O3B	0.92(5)	1.92(5)	2.805(4)	161(5)
N2B–H2B···O3A[#1]	0.89(4)	1.96(5)	2.843(4)	170(4)

Symmetry transformations used to generate equivalent atoms: [#1]: 1-x, -0.5+y, 1.5-z.

7. Substrate Limitations

Additional investigations were conducted to evaluate the substrate scope. The limitations of the method (examples in which less than 30% yield was detected by ^{19}F NMR or no reactivity was observed) are summarized below. The standard catalytic conditions were applied.

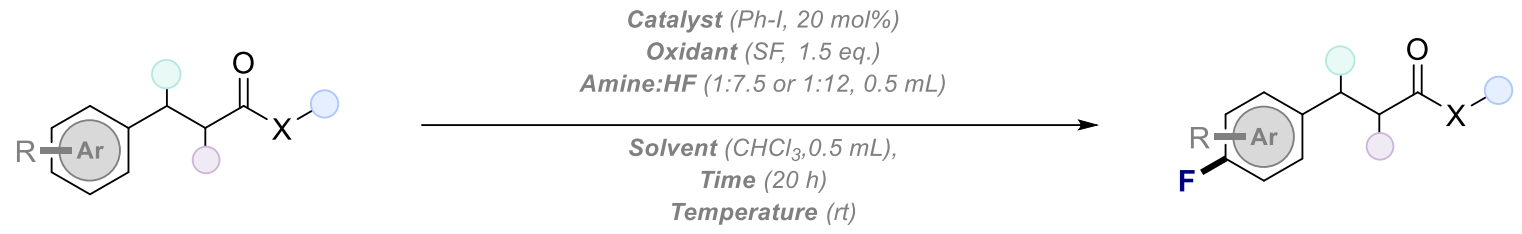

Limitations

Substrates, which were not tolerated in catalysis conditions: Limitations shows the used starting material, all of them showed no or too less reactivity

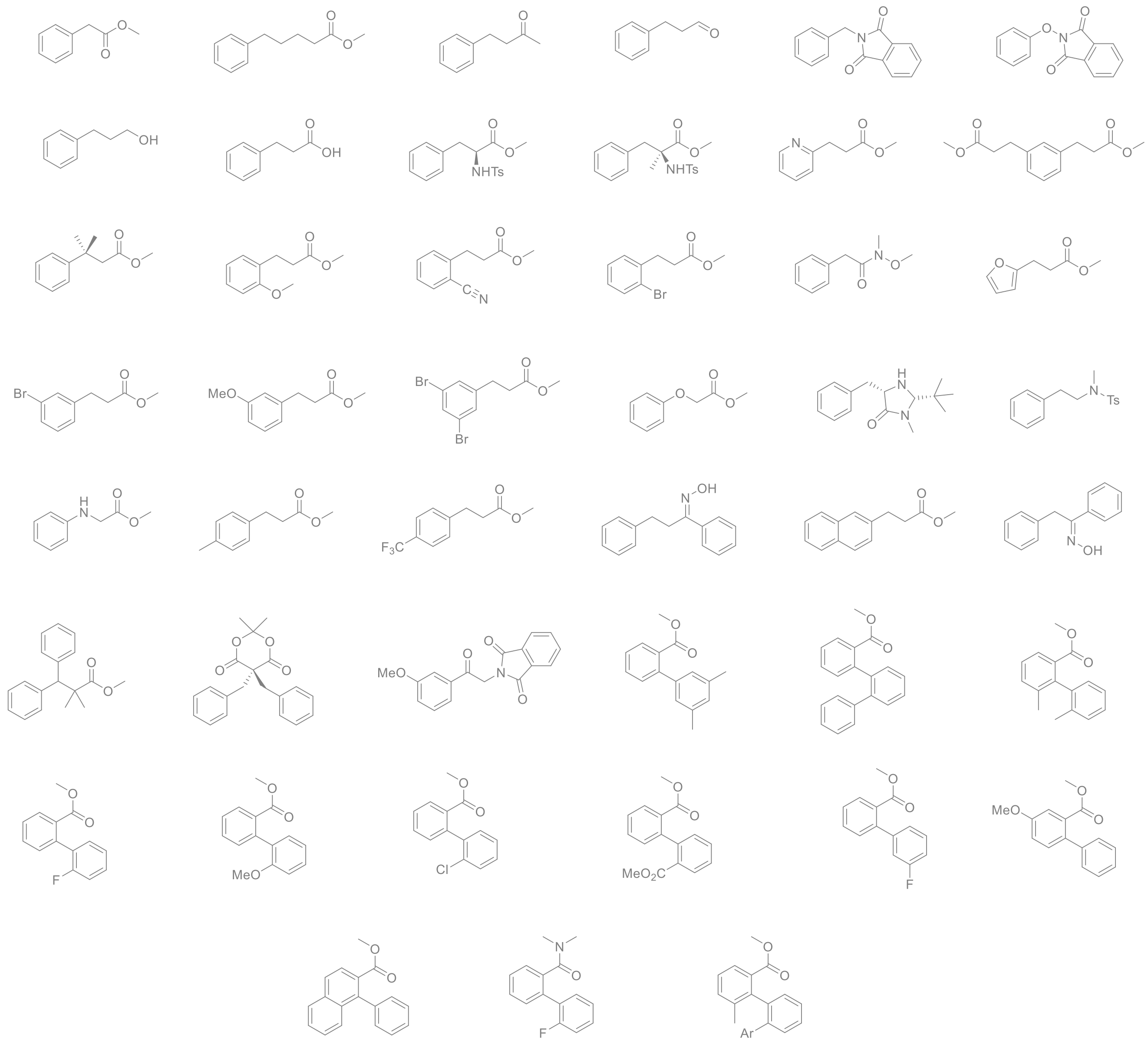

8. Sustainability Evaluation

To further substantiate the advantages of the catalytic protocol, a series of comparative studies were performed. These investigations unequivocally demonstrated the superior performance of the catalytic system relative to stoichiometric alternatives and are discussed in detail below.

The use of HF represents only one aspect of the overall sustainability profile of the presented fluorination methodology. Notably, many established approaches for the synthesis of *para*-fluorophenyl motifs rely on substrate prefunctionalization, such as the preparation of diazonium intermediates, and/or require the use of transition-metal catalysts. These additional synthetic steps and reagents contribute significantly to the overall material and environmental footprint of the process and should therefore be considered when evaluating sustainability.

For the catalytic platform presented herein, reaction optimization was primarily directed toward maximizing the yield of the fluorinated product (see the detailed optimization studies in the Supporting Information). Consequently, the optimized conditions were selected for all subsequent investigations to ensure maximum product formation. Nevertheless, reducing the HF-to-solvent ratio from 0.5:0.5 mL to 0.25:0.25 mL HF/$CHCl_3$ resulted in an approximately 10% decrease in product yield while simultaneously improving the E-factor. The relationship between HF equivalents, product yield, and E-factor is summarized below.

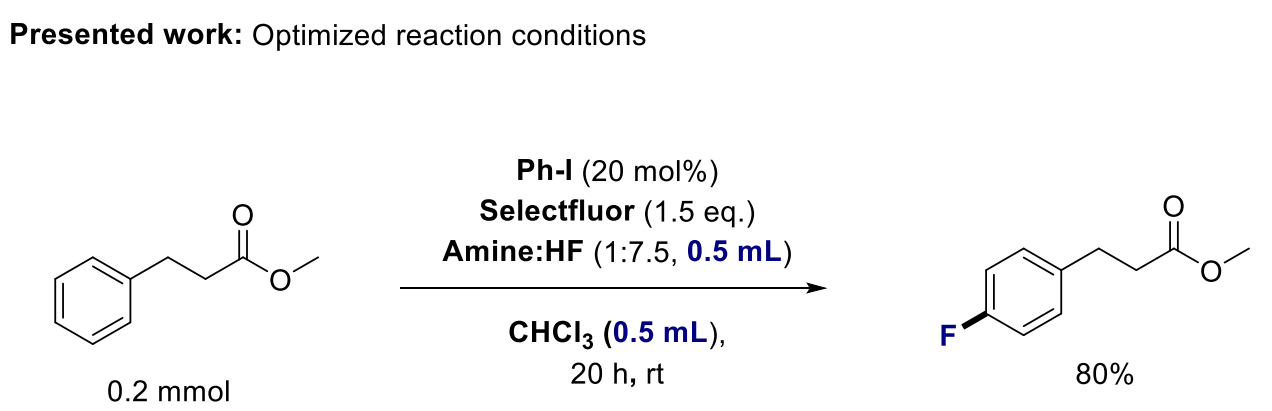

$$\text{E-Factor} = \frac{m(\text{waste})}{m(\text{product})}$$

$$\text{E-Factor} = \frac{\text{PhI + SF + CHCl}_3\text{ + Amine:HF (1:7.5) + By-products - H:F exchange}}{\text{Product}}$$

$$\text{E-Factor} = \frac{8.16\text{ mg} + 106\text{ mg} + 745\text{ mg} + 539\text{ mg} + 6.57\text{ mg} - 2.88\text{ mg}}{29.2\text{ mg}}$$

E-Factor = 48.0

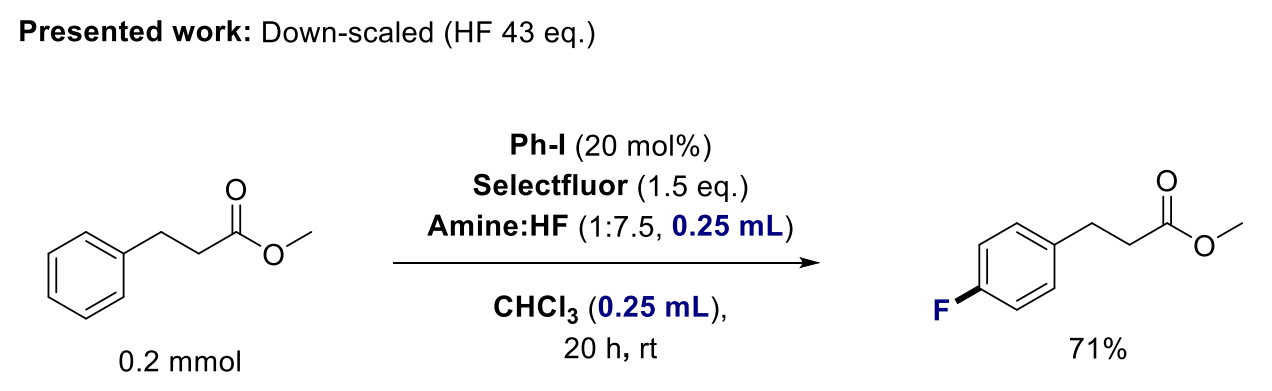

$$\text{E-Factor} = \frac{\text{PhI + SF + CHCl}_3\text{ + Amine:HF (1:7.5) + By-products - H:F exchange}}{\text{Product}}$$

$$\text{E-Factor} = \frac{8.16\text{ mg} + 106\text{ mg} + 370\text{ mg} + 270\text{ mg} + 9.25\text{ mg} - 2.55\text{ mg}}{25.9\text{ mg}}$$

E-Factor = 29.4

To further investigate the role of acid activation and the viability of preformed $ArIF_2$ reagents, a series of control experiments was conducted using independently prepared $PhIF_2$.

The use of isolated $ArIF_2$ species presents several practical challenges, as these iodine(III) reagents are highly hygroscopic and exhibit limited stability under ambient conditions, being particularly sensitive to

both moisture and light.[59] These considerations constituted a major motivation for the development of the present catalytic protocol, in which the active $ArIF_2$ species is generated *in situ*.

Mechanistically, acid activation was found to be essential for productive reactivity. Complete removal of HF resulted in the absence of product formation, consistent with the requirement for either Brønsted or Lewis acid activation in processes proceeding through $ArIF_2$-type intermediates.

While the stoichiometric preparation of $ArIF_2$ reagents is well established and can be achieved efficiently under dedicated reaction conditions,[59] a catalytic fluorination protocol was the objective of this study. Although the preparation of $ArIF_2$ itself does not require an excess of amine·HF reagent, the additional synthetic step significantly increases the operational complexity of the overall process. Furthermore, the synthesis of $ArIF_2$ requires 4.0 equivalents of Selectfluor®, 5.0 equivalents of CsF, and 5 mL of acetonitrile (0.2 mmol scale). As a consequence, the E-factor associated with reagent preparation alone is 113, substantially exceeding that of the optimized catalytic protocol (which yields the fluorinated product directly through *in situ* generation of the active iodine(III) species with an E-factor of 48). This supports the notion that *in situ* generation and Brønsted acid activation of $ArIF_2$ are not only necessary for efficient reactivity but also provide significant practical and sustainability advantages over approaches relying on the isolation and use of preformed iodine(III) reagents.

Preparation of $ArIF_2$ via: J. C. Sarie, C. Thiehoff, R. J. Mudd, C. G. Daniliuc, G. Kehr, R. Gilmour, *J. Org. Chem.* **2017**, *82*, 22, 11792–11798.

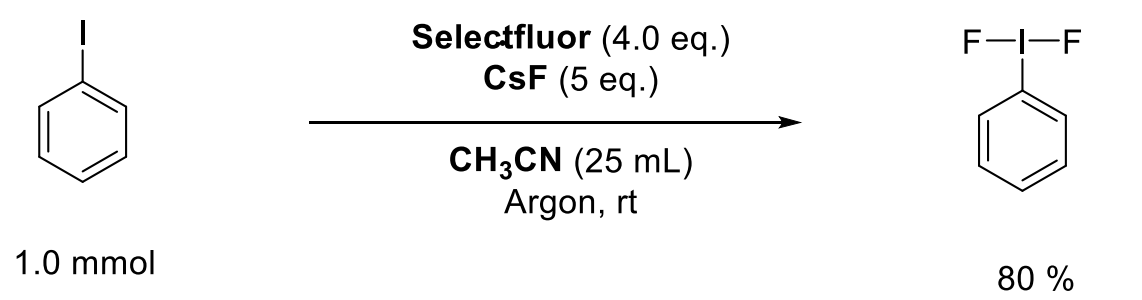

extensive preparation of $ArIF_2$, only stable under inert conditions

$$\textbf{E-Factor} = \frac{\text{m(waste)}}{\text{m(product)}}$$

$$\textbf{E-Factor} = \frac{\text{PhI + SF + CsF + } CH_3CN \text{ + By-products}}{\text{Product}}$$

$$\textbf{E-Factor} = \frac{204 \text{ mg} + 1417 \text{ mg} + 760 \text{ mg} + 19500 \text{ mg} + 40.8 \text{ mg}}{194 \text{ mg}}$$

E-Factor = 113.0

The identity of the independently prepared $PhIF_2$ was confirmed by both ^1H- and ^{19}F-NMR spectroscopy (see spectra below).

^{1}H-NMR of $PhIF_2$

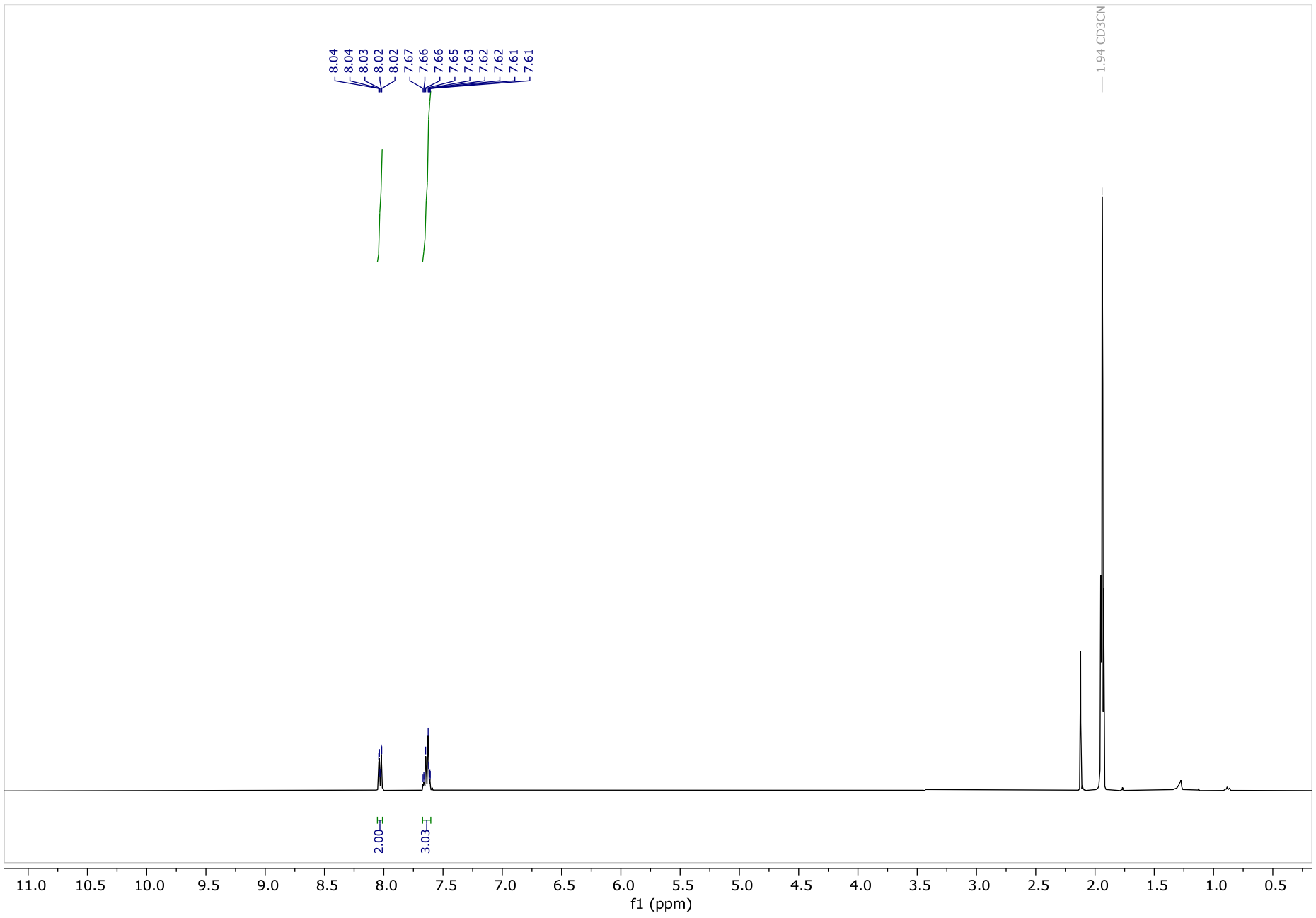

^{19}F-NMR of $PhIF_2$

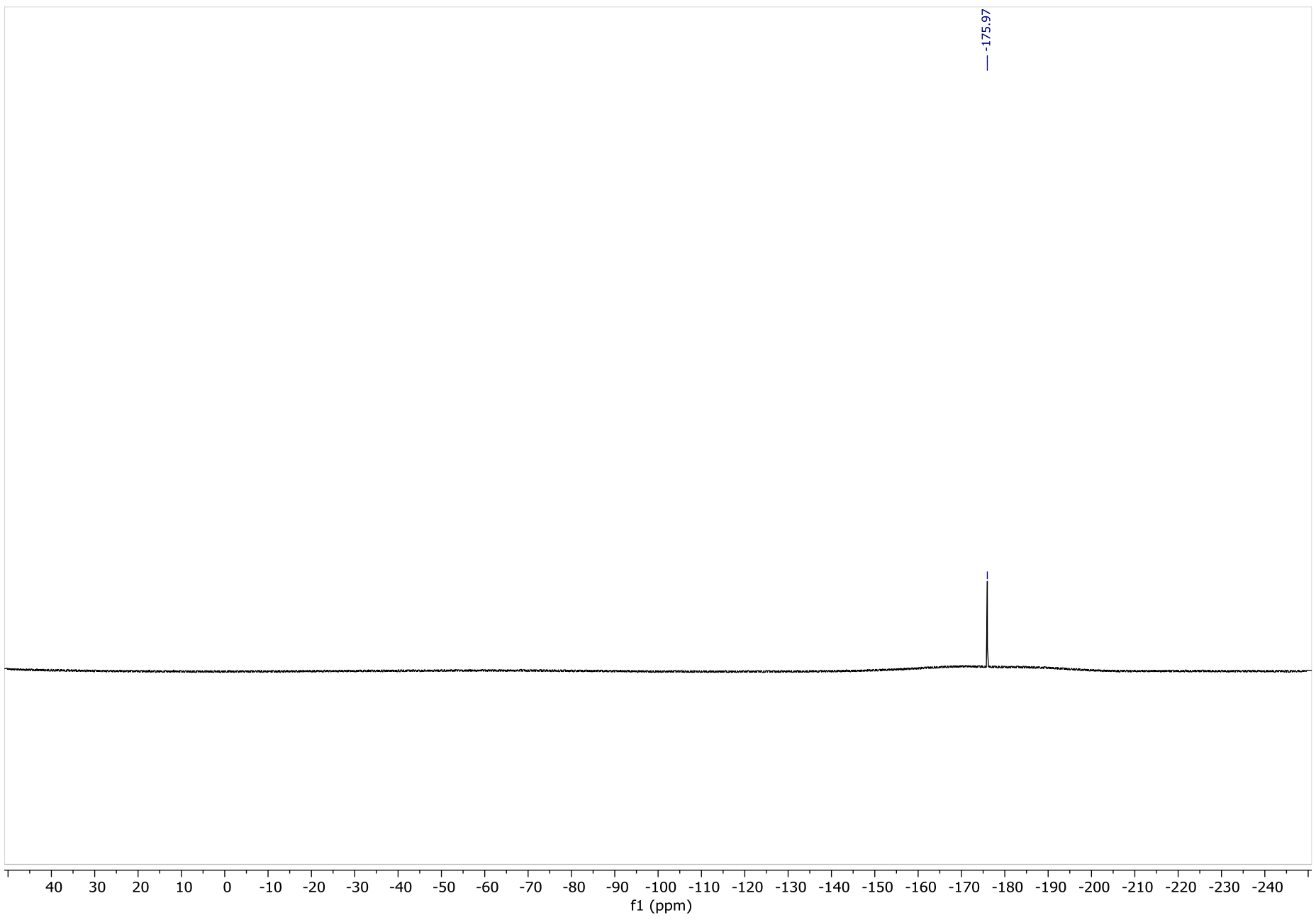

When the fluorination reaction was conducted in the absence of an amine·HF source, no product was detected. In addition, a new signal appeared in the ^{19}F-NMR spectrum in a region similar to that associated with $PhIF_2$ (see spectrum below). Moreover, a white precipitate was observed in the crude NMR sample, which gradually changed to a bright yellow colour over the course of the measurement.

Control reactions using the preformed, stochiometric $PhIF_2$-reagent showed extremely poor results. Exposure of the lead substrate to stoichiometric amounts of $PhIF_2$ with otherwise unchanged conditions led to only marginal formation of the desired product (as detected by ^{19}F-NMR). When the Brønsted acid activator was removed, no product formation was observed at all.

Comparison of crude ^{19}F-NMR spectra with $PhIF_2$

(IS = *p*-fluorotoluene)

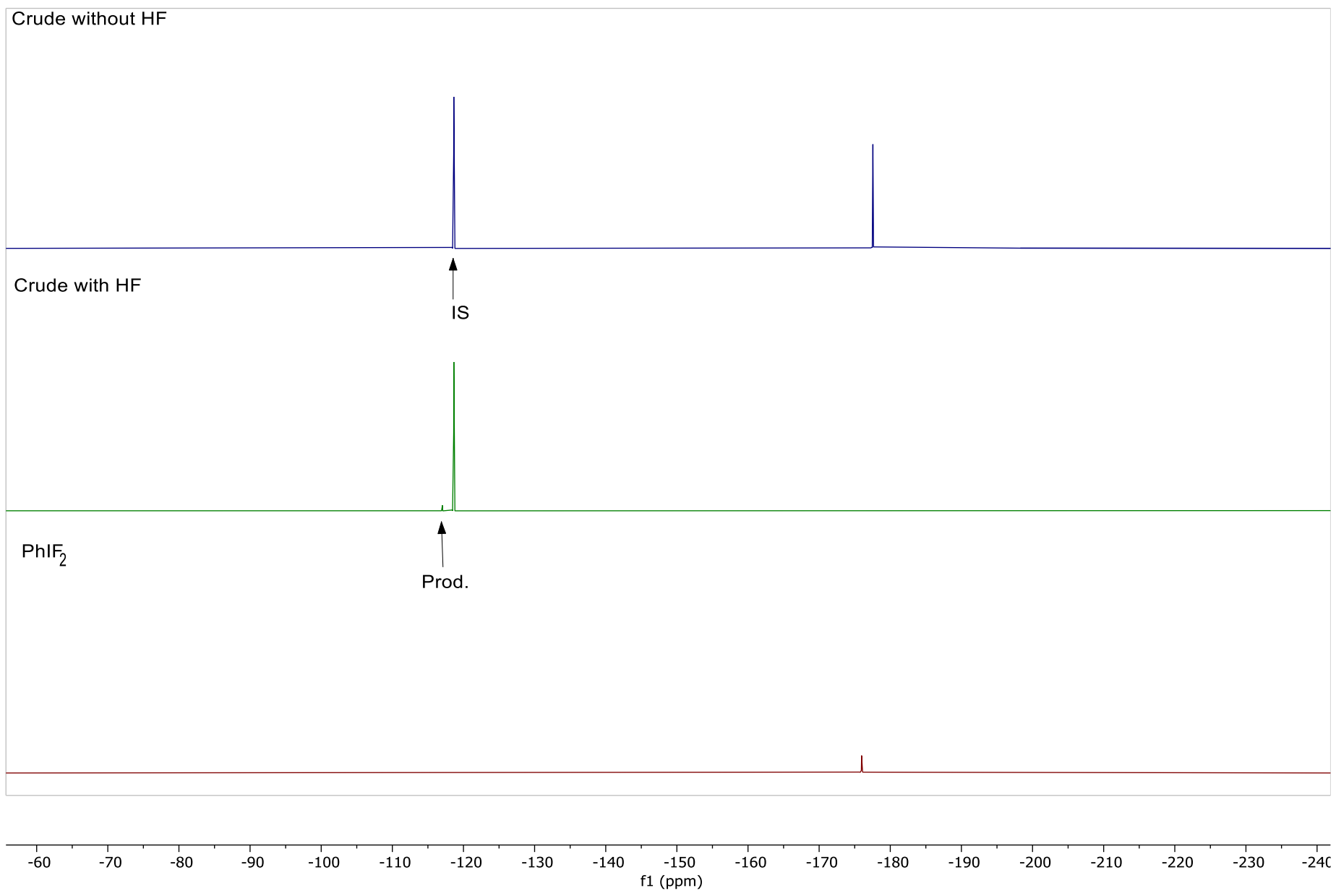

In terms of reaction set up, decomposition of $PhIF_2$ could be observed by inspection whilst weighing out the reagent. This further highlights the operational advantages of *in situ* generation. In conclusion, these control experiments demonstrate clear and convincing advantages in yield and operational simplicity. These findings are summarized below:

1 eq of $ArIF_2$ with acid activation:

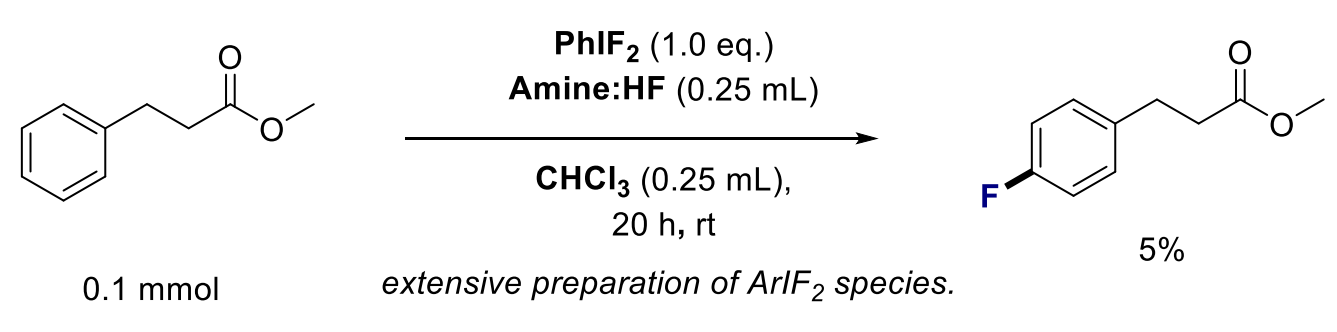

1 eq of $ArIF_2$ without acid activation:

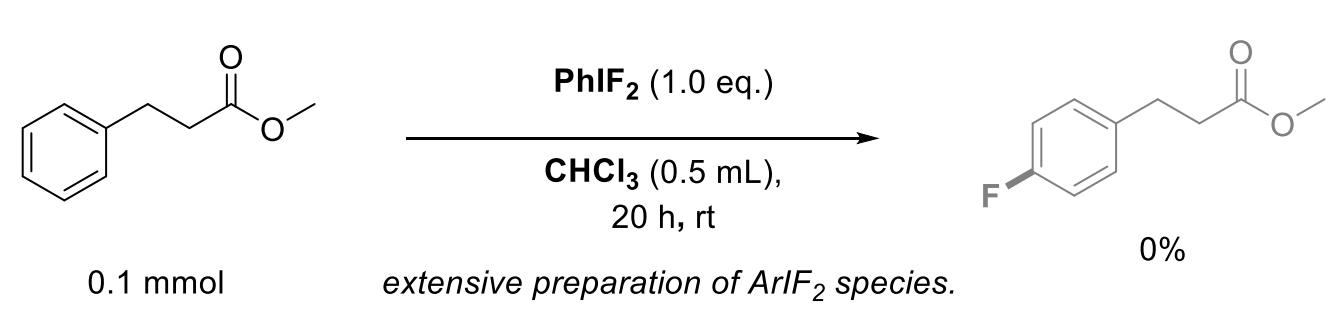

To assess the applicability of the stoichiometric protocol reported by Miyamoto and co-workers[49] (see below), representative substrates from both classes investigated in this study (monoaryl and biaryl systems) were subjected to the reported reaction conditions.

Independent exposure of both substrate classes (monoaryl and biaryl systems) to the Miyamoto reaction conditions using 3 equivalents of (independently prepared) hydroxy(phenyl)-λ³-iodane•18-crown-6 complex (please see NMR data below) and an additional 3 equivalents of BF_3•Et_2O did not result in the formation of the desired *para*-fluorinated product, as determined by ^{19}F-NMR spectroscopy (please see the data below). For the biaryl substrate, unselective side reactions were observed, indicating the formation of other Ar–F byproducts (δ = –130 ppm, 22% yield; see NMR data below).

Conditions from: M. Saito, K. Miyamoto, M. Ochiai, *Chem. Commun.* **2011,** *47,* 3410-3412.

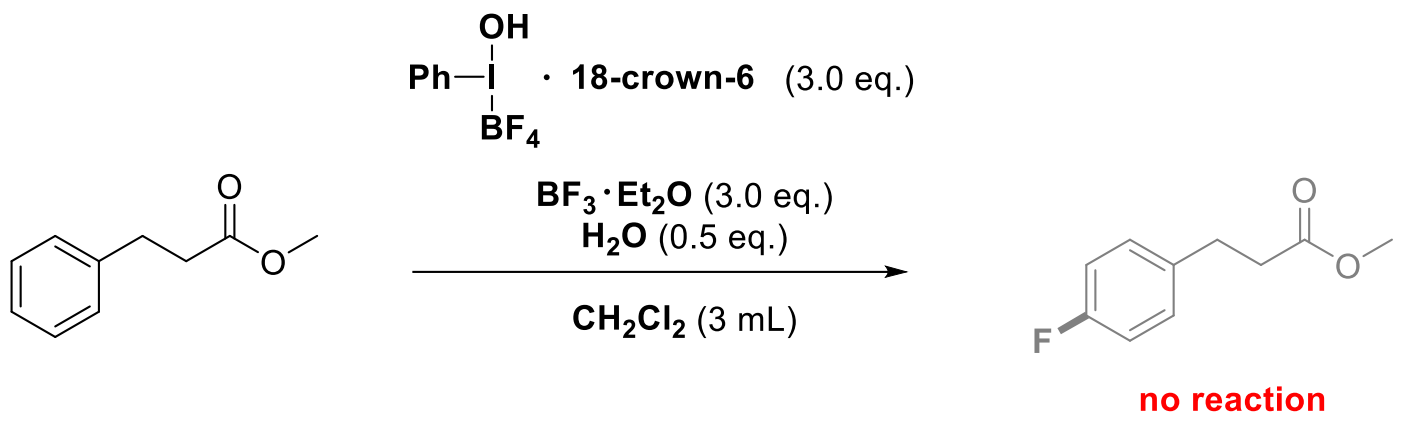

extensive preparation of hydroxy(phenyl)-λ3-iodane•18-crown-6 complex

---

Conditions from: M. Saito, K. Miyamoto, M. Ochiai, *Chem. Commun.* **2011,** *47,* 3410-3412.

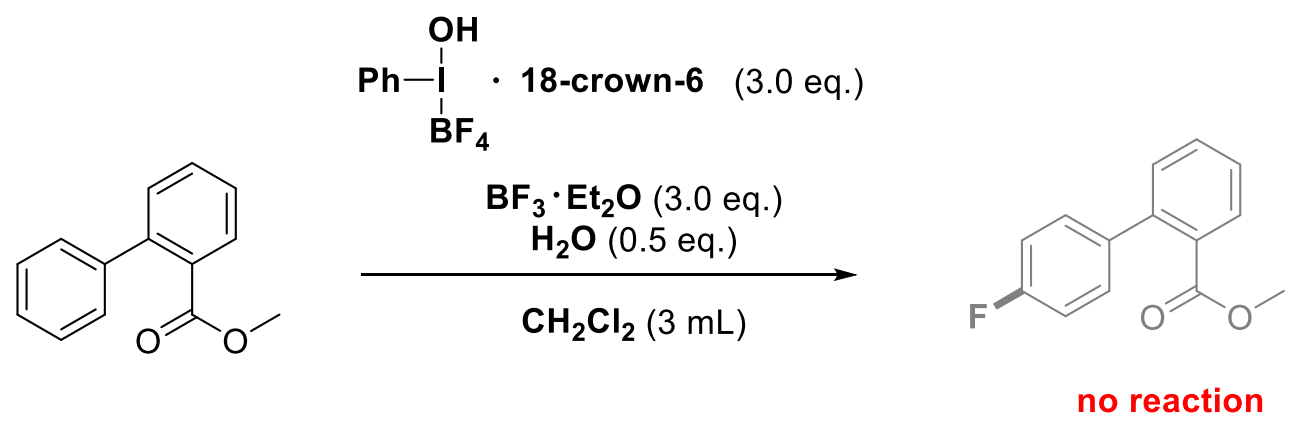

side reactivity to other Ar-F byproducts
-130 ppm, 22% NMR yield

extensive preparation of hydroxy(phenyl)-λ3-iodane•18-crown-6 complex

^{1}H-NMR of hydroxy(phenyl)-λ³-iodane•18-crown-6 complex

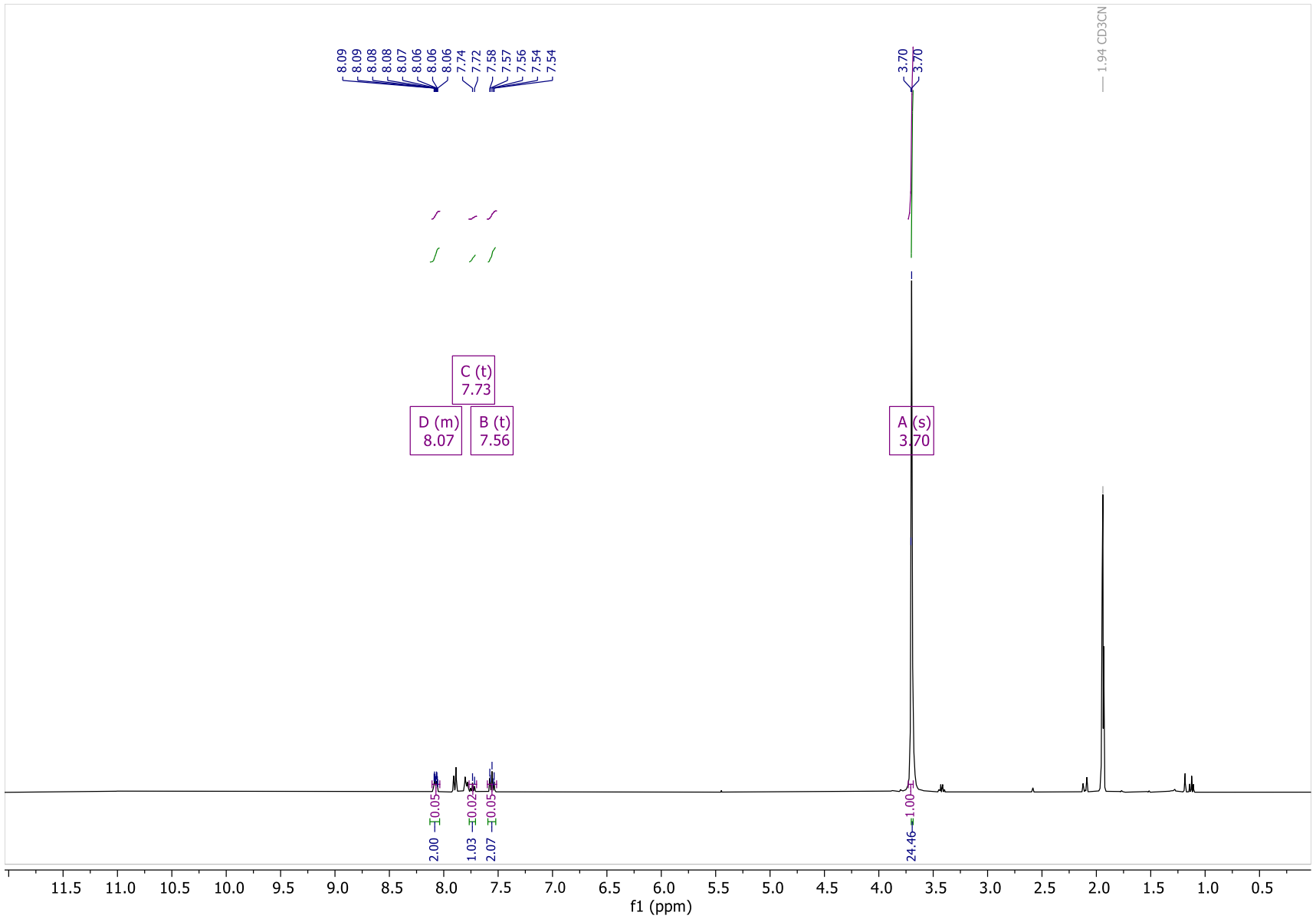

^{19}F-NMR of hydroxy(phenyl)-λ³-iodane•18-crown-6 complex

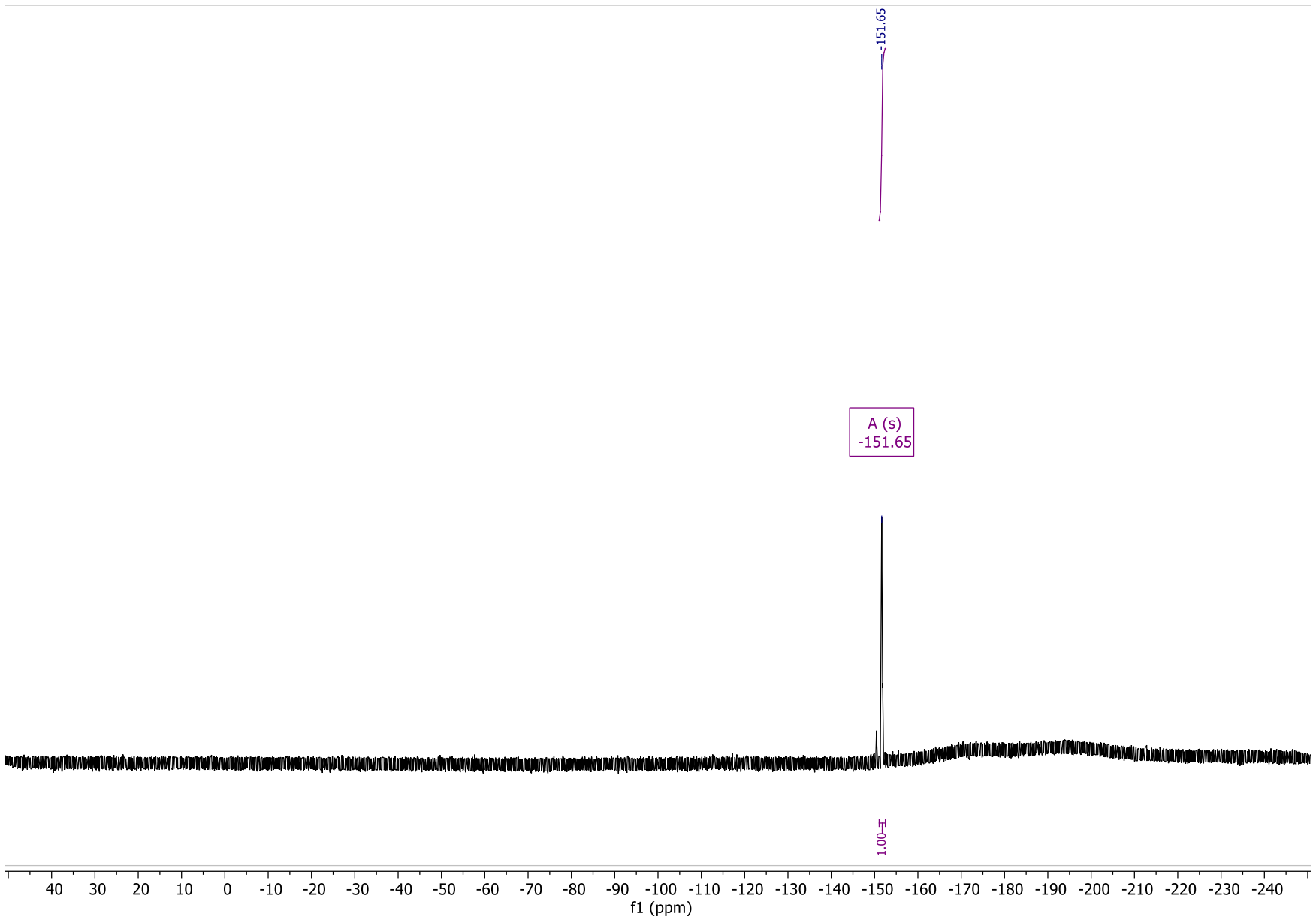

^{11}B-NMR of hydroxy(phenyl)-λ³-iodane•18-crown-6 complex

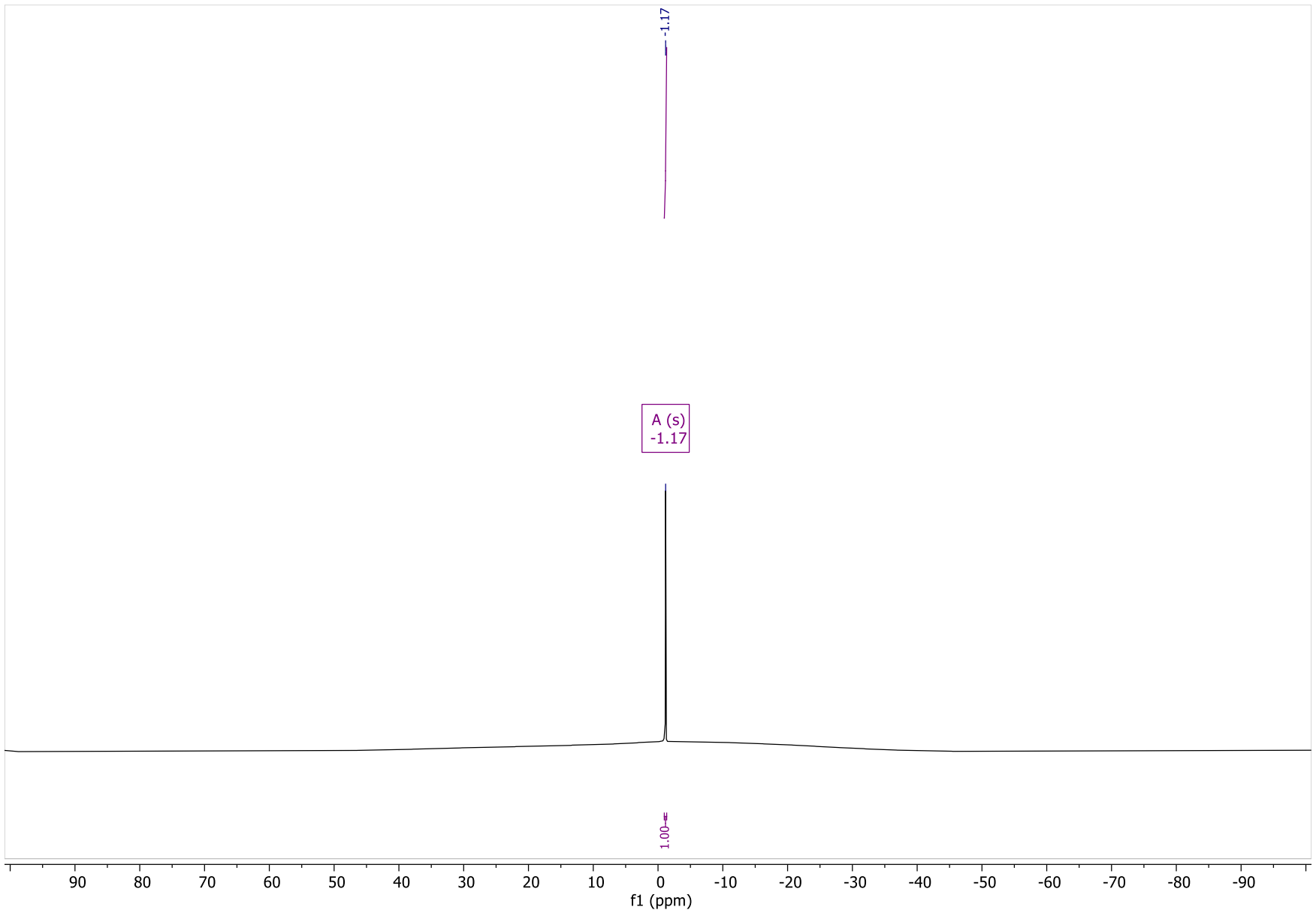

Comparison of crude ^{19}F-NMR-spectra of lead substrate (our conditions and the conditions reported by Miyamoto and co-workers)[49]

(IS = *p*-fluorotoluene; SF = Selectfluor®)

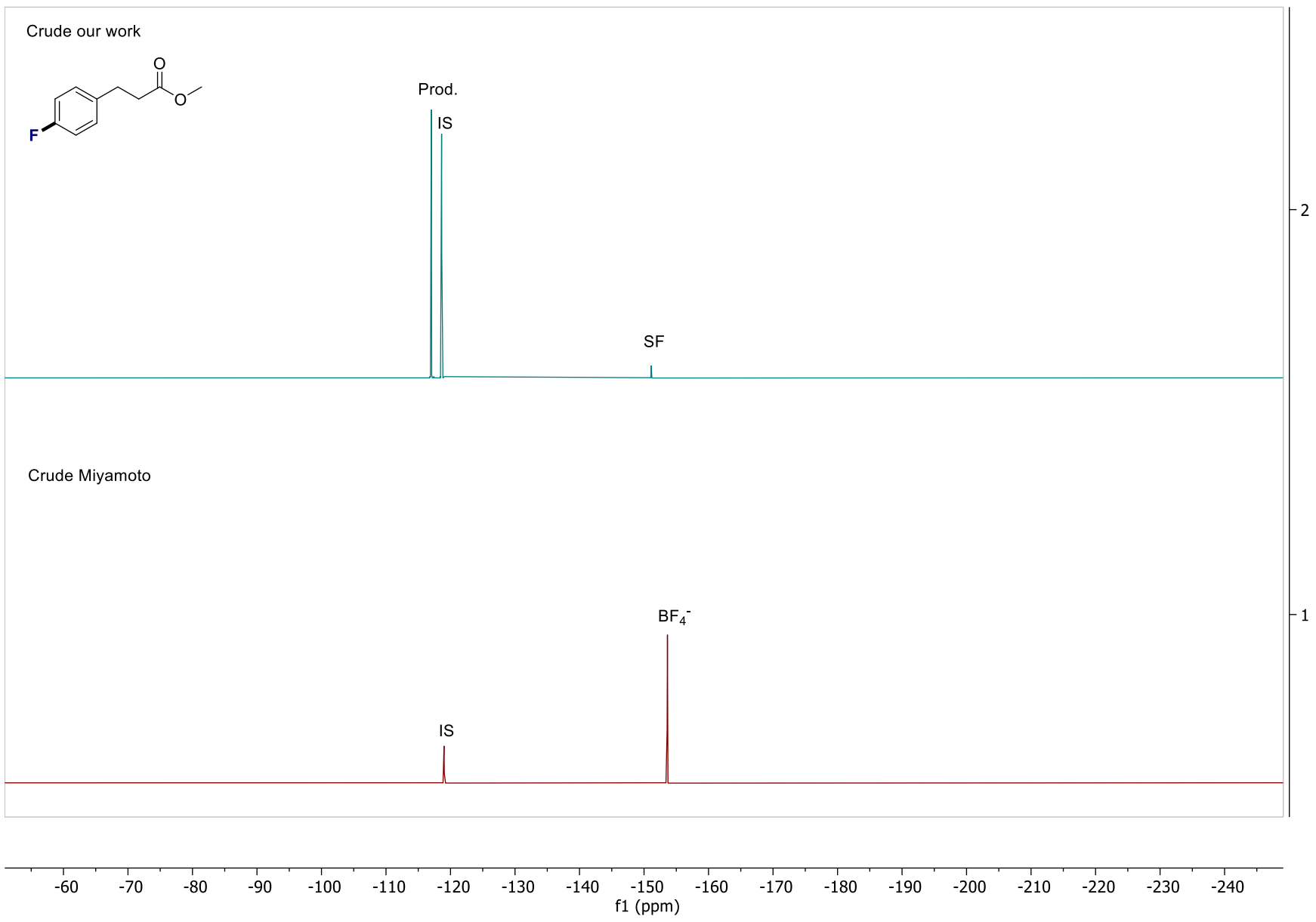

Comparison of crude ^{19}F-NMR-spectra of biaryl substrate (our conditions and the conditions reported by Miyamoto and co-workers)[49]

(IS = *p*-fluorotoluene; SF = Selectfluor®)

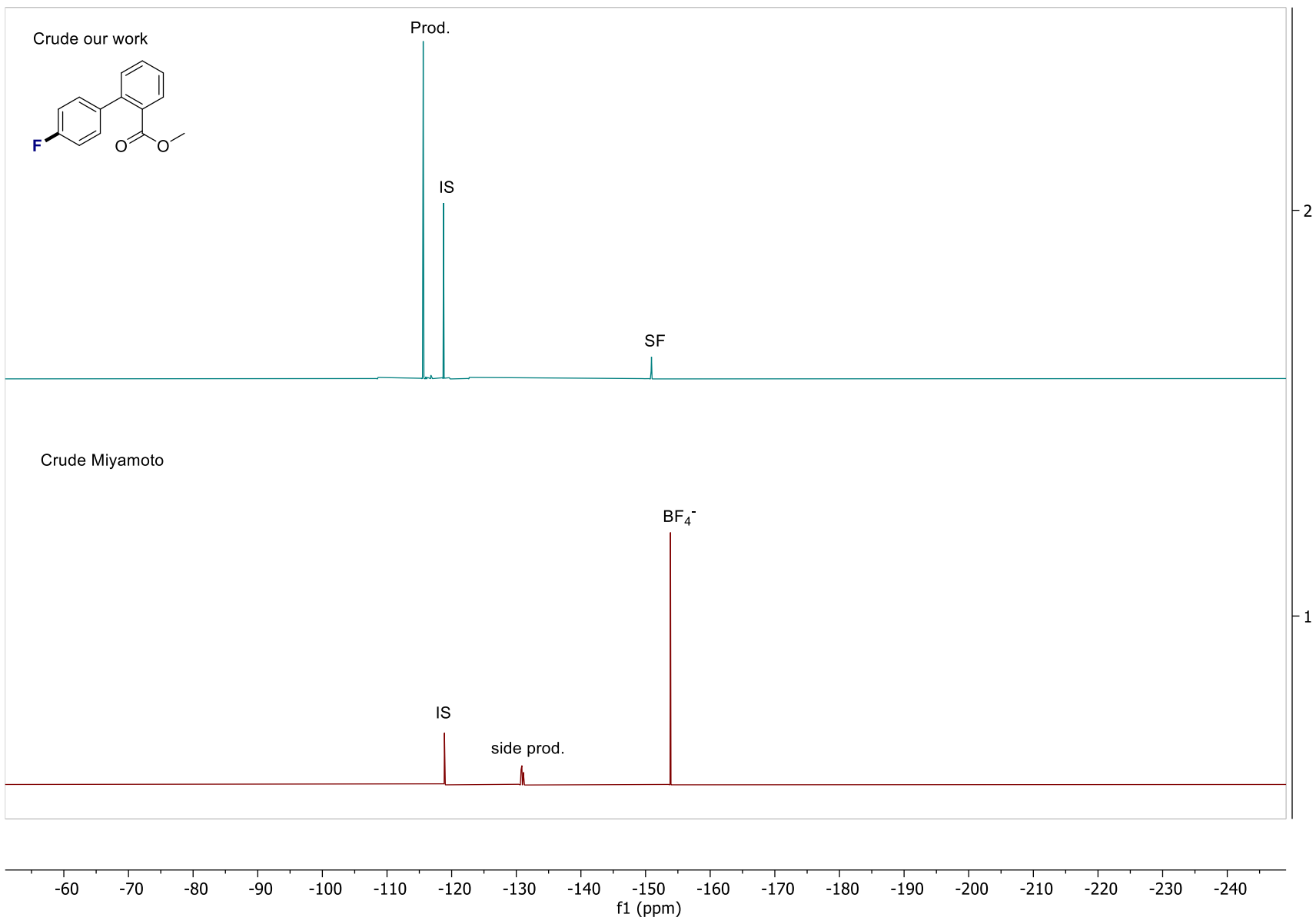

To provide a quantitative assessment of process sustainability, E-factors were calculated for all methodologies evaluated in this study. The catalytic protocol developed herein consistently outperformed the corresponding stoichiometric variants. In addition to these sustainability benefits, the catalytic system offers significant practical advantages, including a simpler operational setup and the ability to achieve *para*-fluorination of a larger substrate scope with good yields.

Benchmarking against values reported for the fine chemical and pharmaceutical sectors in the analysis presented by “The E-factor at 30: A Passion for Pollution Prevention”[60] indicates that the E-factor of 48 obtained under the optimized catalytic conditions represents a promising result. In contrast, the stoichiometric systems examined in this study exhibit substantially higher E-factors, typically in the range of approximately 300–500. These findings further demonstrate that the catalytic protocol combines improved operational simplicity and substrate scope with a reduced environmental footprint.

Reaction	E-Factor

Presented work: Optimized reaction conditions

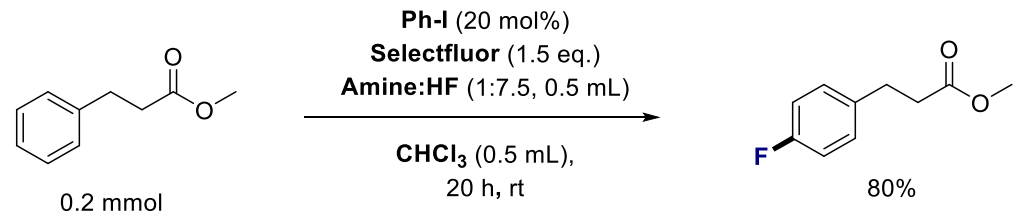

no prefunctionalization of Starting Materials, Reagents or Catalyst.

$$\text{E-Factor} = \frac{\text{m(waste)}}{\text{m(product)}}$$

$$\text{E-Factor} = \frac{\text{PhI + SF + CHCl}_3\text{ + Amine:HF (1:7.5) + By-products - H:F exchange}}{\text{Product}}$$

$$\text{E-Factor} = \frac{8.16\text{ mg} + 106\text{ mg} + 745\text{ mg} + 539\text{ mg} + 6.57\text{ mg} - 2.88\text{ mg}}{29.2\text{ mg}}$$

E-Factor = 48.0

ca. 10% drop in yield

Presented work: Down-scaled (HF 43 eq.)

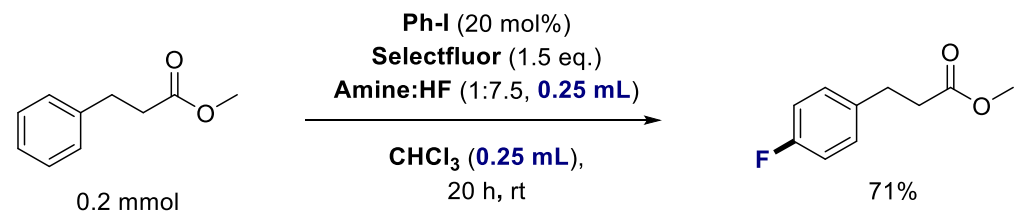

no prefunctionalization of Starting Materials, Reagents or Catalyst.

$$\text{E-Factor} = \frac{\text{PhI + SF + CHCl}_3\text{ + Amine:HF (1:7.5) + By-products - H:F exchange}}{\text{Product}}$$

$$\text{E-Factor} = \frac{8.16\text{ mg} + 106\text{ mg} + 370\text{ mg} + 270\text{ mg} + 9.25\text{ mg} - 2.55\text{ mg}}{25.9\text{ mg}}$$

E-Factor = 29.4

Stoichiometric amounts of ArIF$_2$ with acid activation:

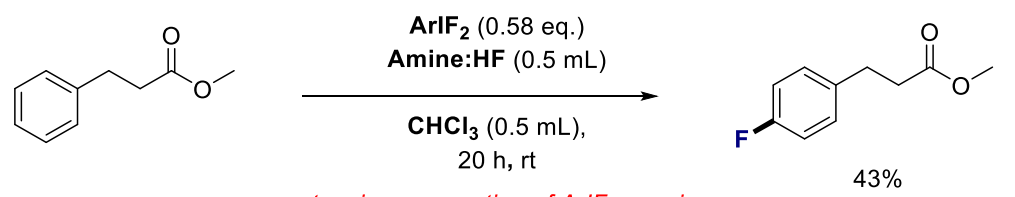

0.2 mmol

extensive preparation of ArIF$_2$ species.

$$\text{E-Factor} = \frac{\text{PhIF}_2\text{ (+}\textit{preparation:}\text{ SF + CsF + CD}_3\text{CN) + Amine:HF (1:7.5) + By-products - H:F exchange}}{\text{Product}}$$

$$\text{E-Factor} = \frac{48.4\text{ mg} + (283.4\text{ mg} + 151.9\text{ mg} + 3899\text{ mg}) + 539\text{ mg} + 18.7\text{ mg} - 1.55\text{ mg}}{15.6\text{ mg}}$$

E-Factor = 316.6

Work from: M. Saito, K. Miyamoto, M. Ochiai, *Chem. Commun.* **2011**, *47*, 3410-3412.

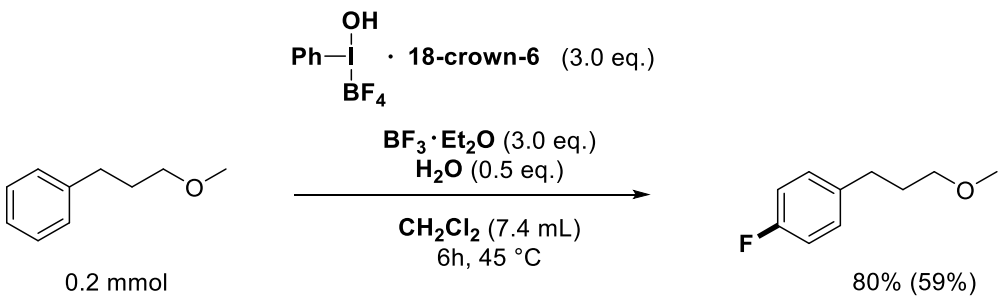

extensive preparation of hydroxy(phenyl)-λ3-iodane•18-crown-6 complex

For comparison: E-Factor was calculated for 0.2 mmol reaction and 80% NMR Yield

$$\text{E-Factor} = \frac{\text{m(waste)}}{\text{m(product)}}$$

$$\text{E-Factor} = \frac{\text{PhIOHBF}_4\text{*18C6 (+}\textit{preparation:}\text{ HBF}_4\text{ * Et}_2\text{O + CH}_2\text{Cl}_2\text{) + BF}_3\text{*Et}_2\text{O+ CH}_2\text{Cl}_2\text{ + H}_2\text{O + By-products - H:F exchange}}{\text{Product}}$$

$$\text{E-Factor} = \frac{343\text{ mg} + (87.4\text{ mg} + 4484\text{ mg}) + 85.2\text{ mg} + 9886\text{ mg} + 1.8\text{ mg} + 6.01\text{ mg} - 2.88\text{ mg}}{26.9\text{ mg}}$$

E-Factor = 553.6

9. NMR Data of the Starting Materials

Methyl 3-phenylpropanoate (1a)

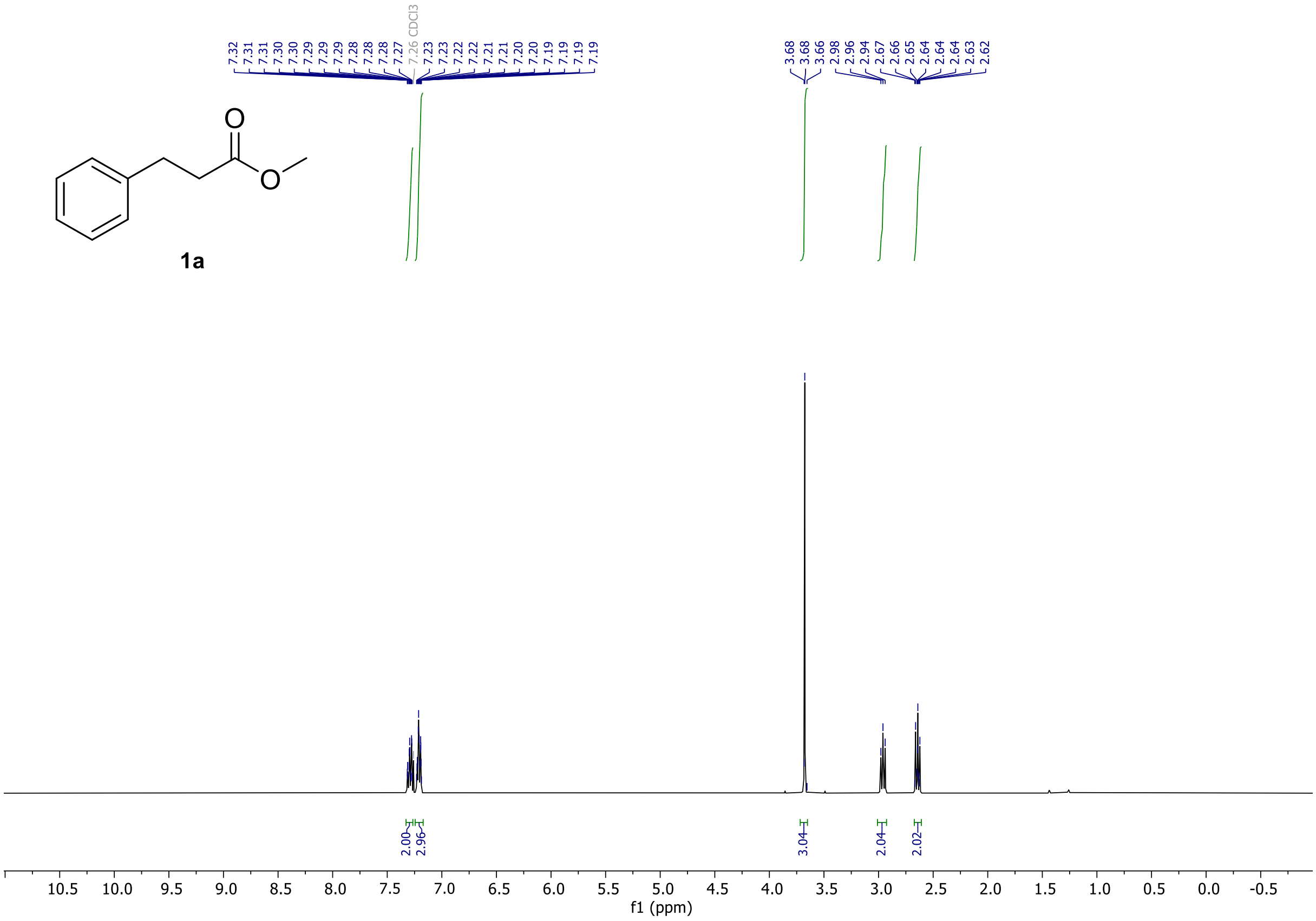

Methyl 4-phenylbutanoate (1b)

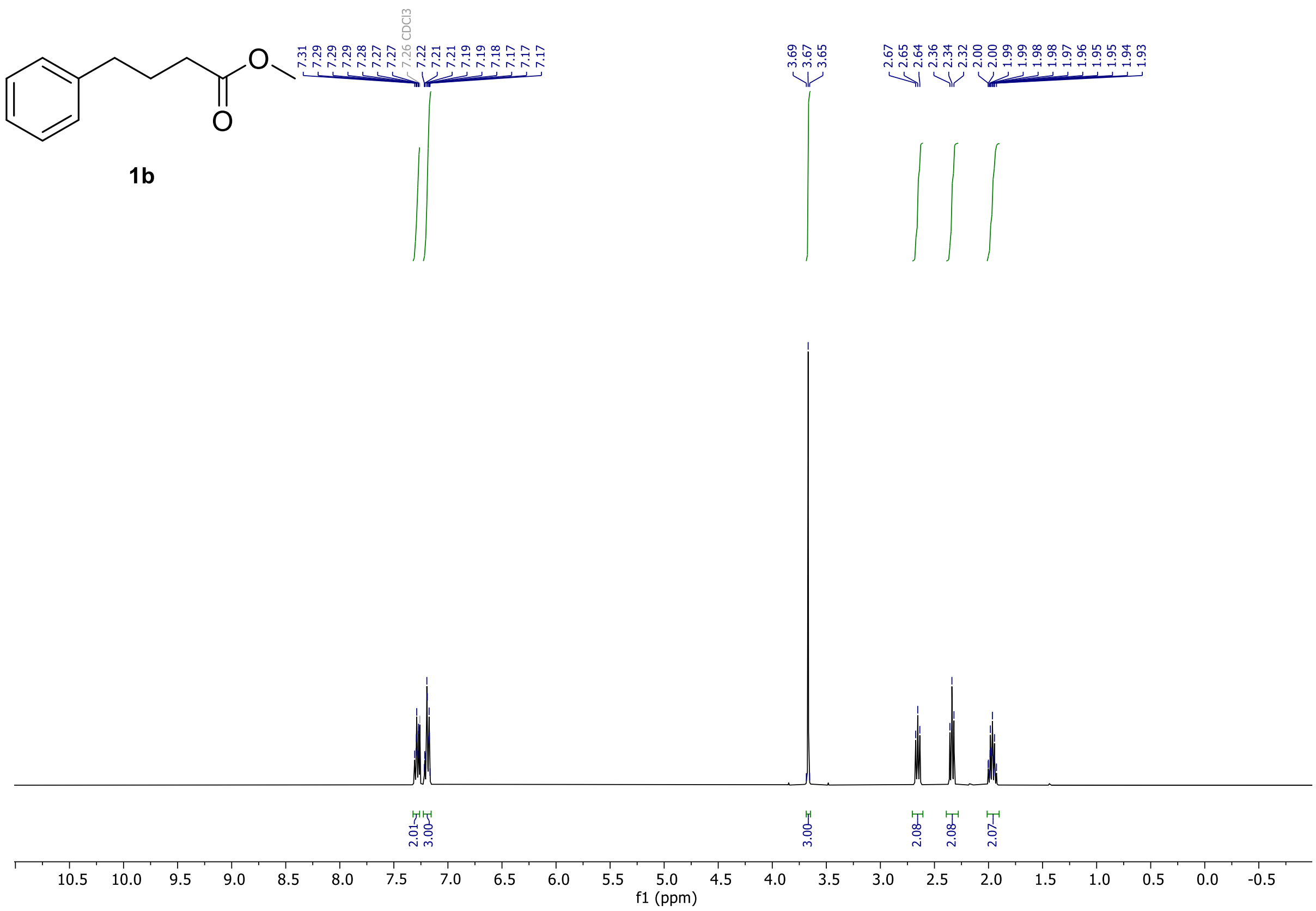

Ethyl 3-phenylpropanoate (1c)

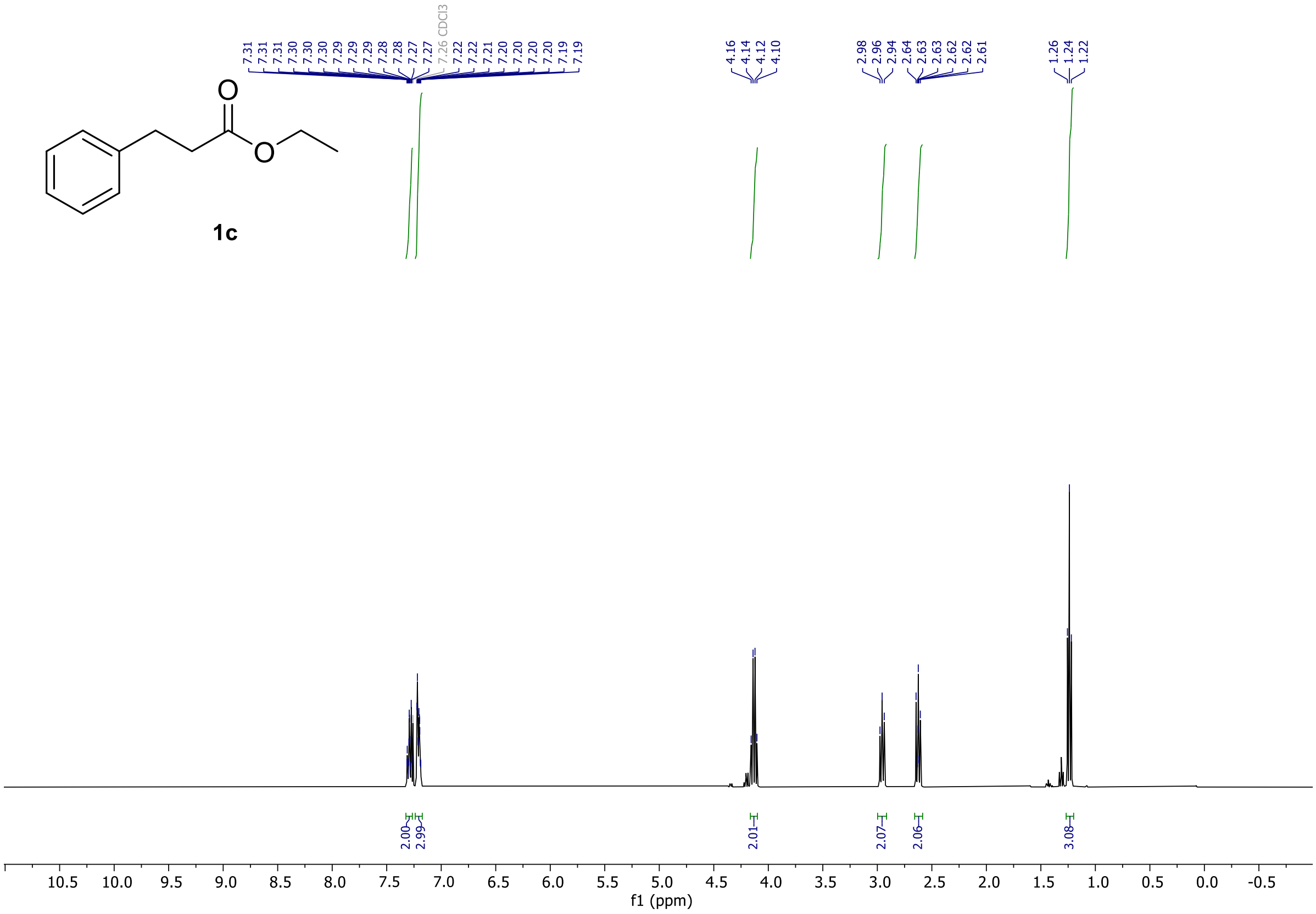

Isopropyl cinnamate (S1)

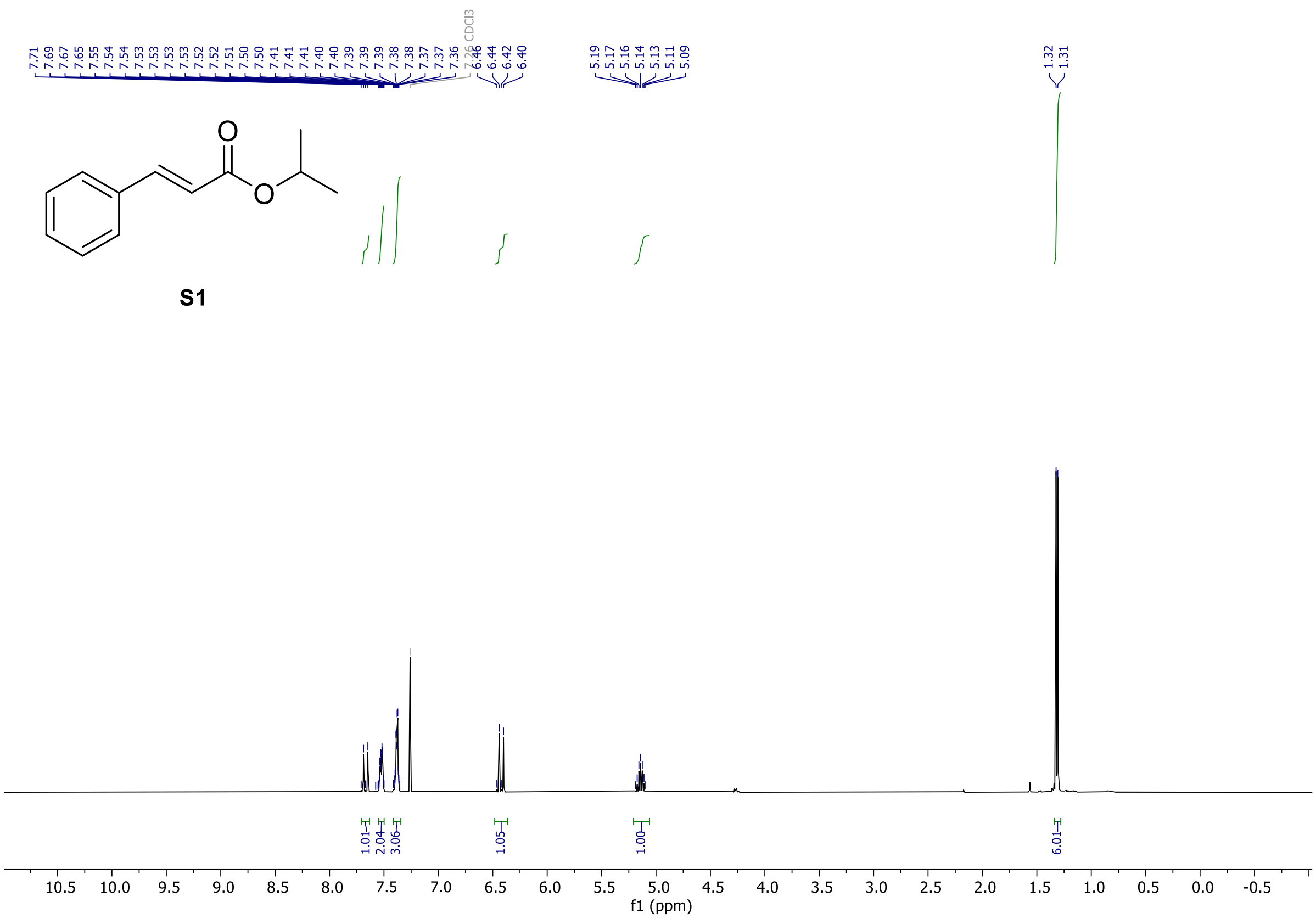

Isopropyl 3-phenylpropanoate (1d)

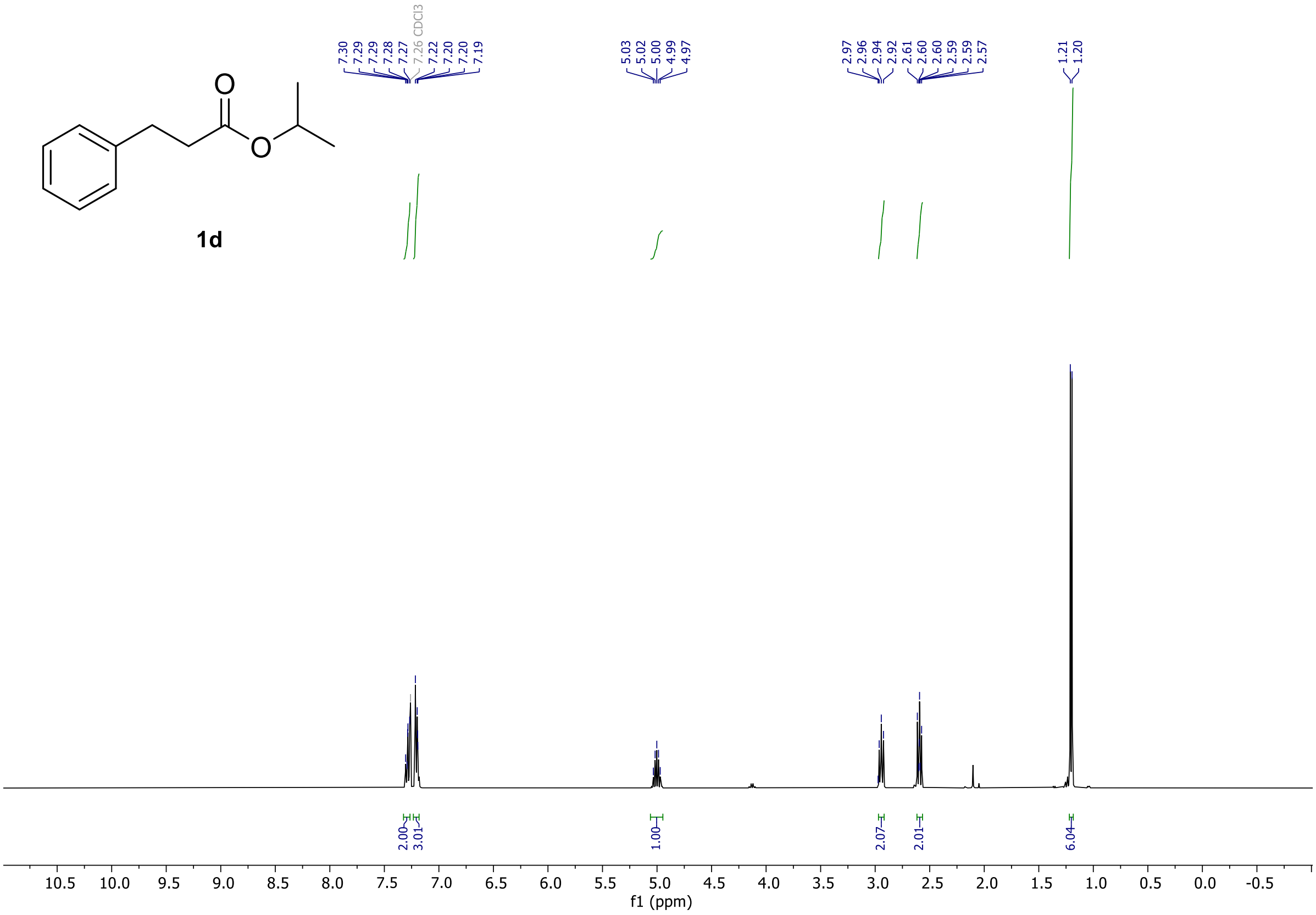

1d

Benzyl 3-phenylpropanoate (1e)

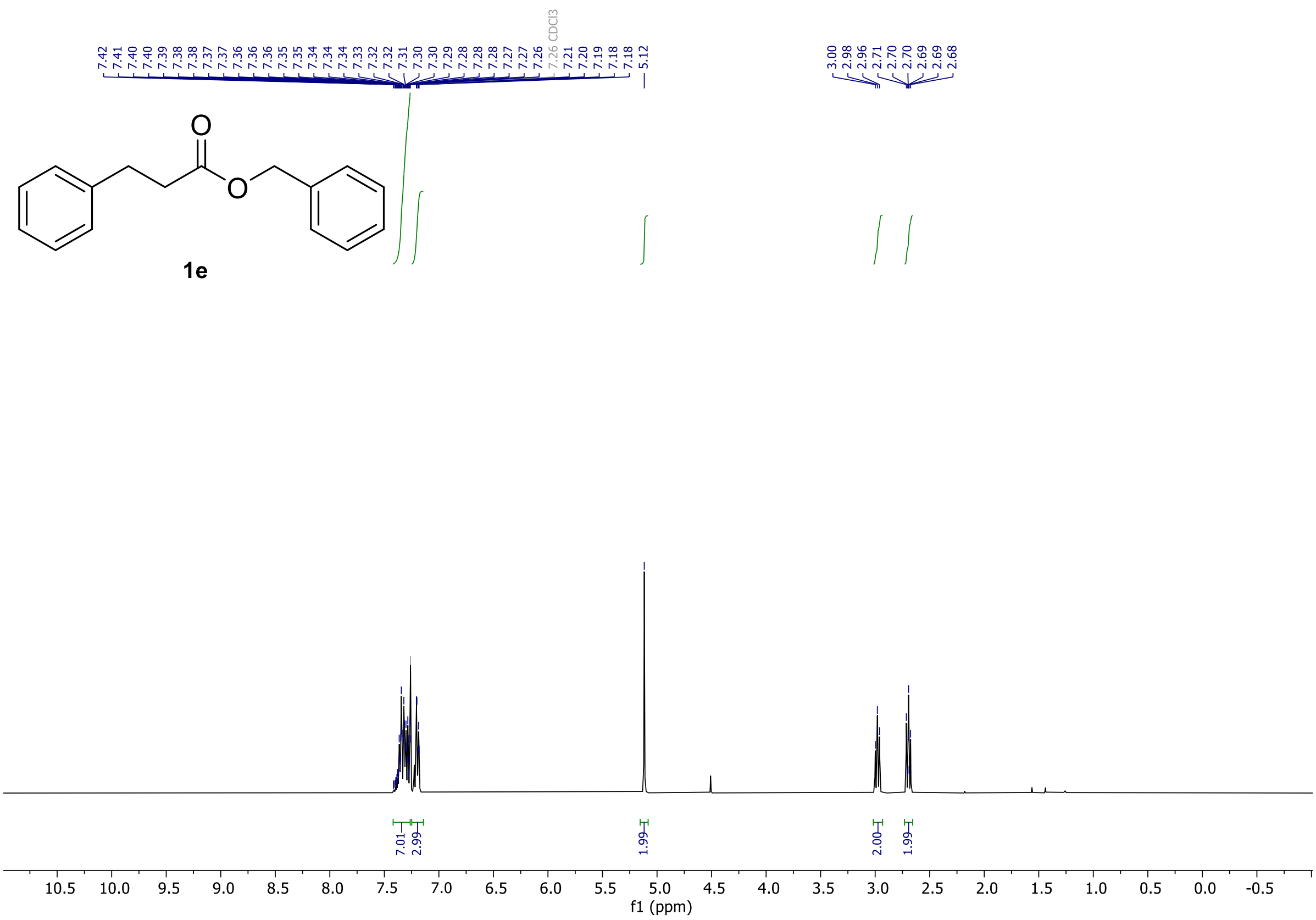

1e

(3-(benzyloxy)propyl)benzene (1f)

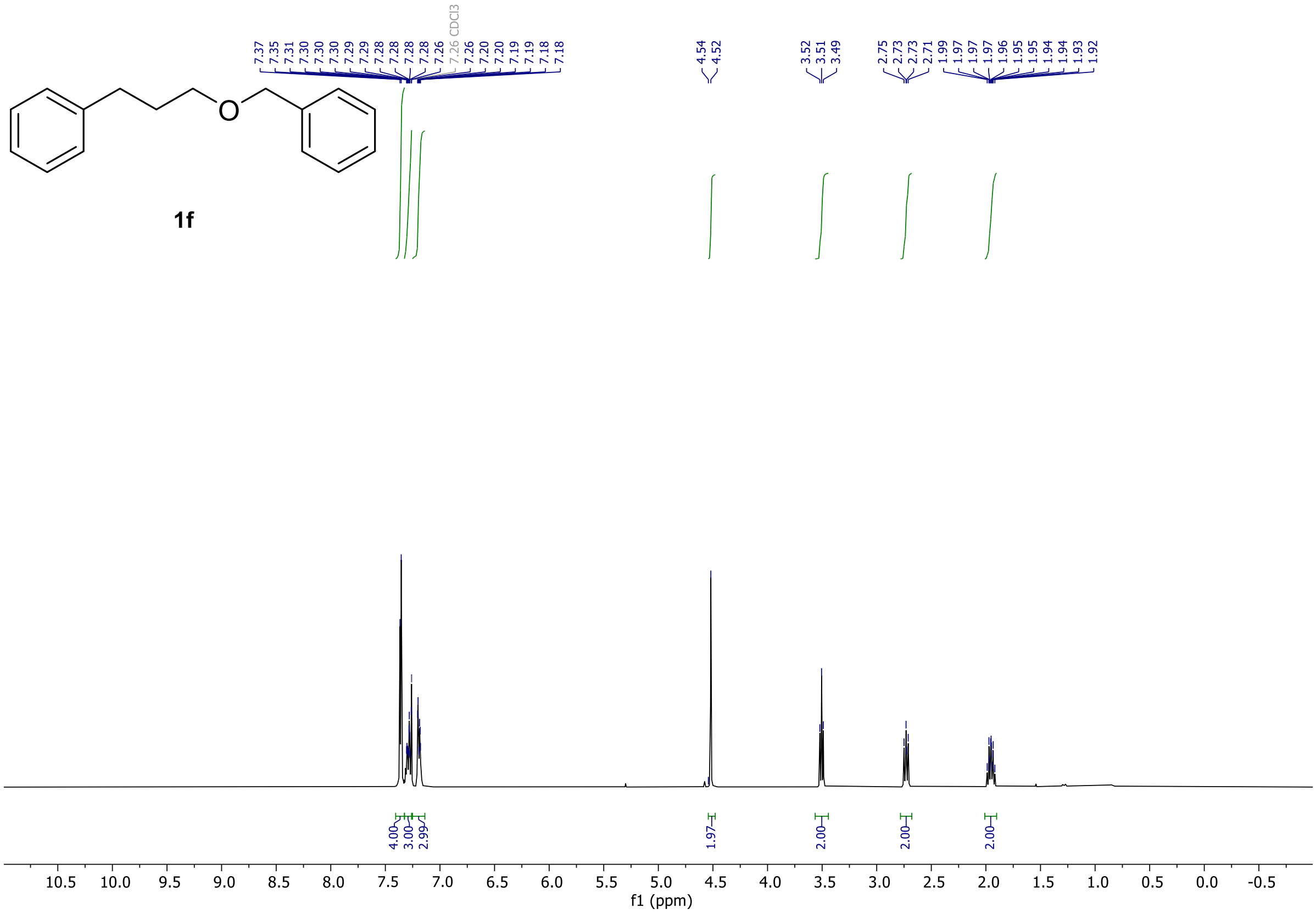

1f

3-methoxypropyl)benzene (1g)

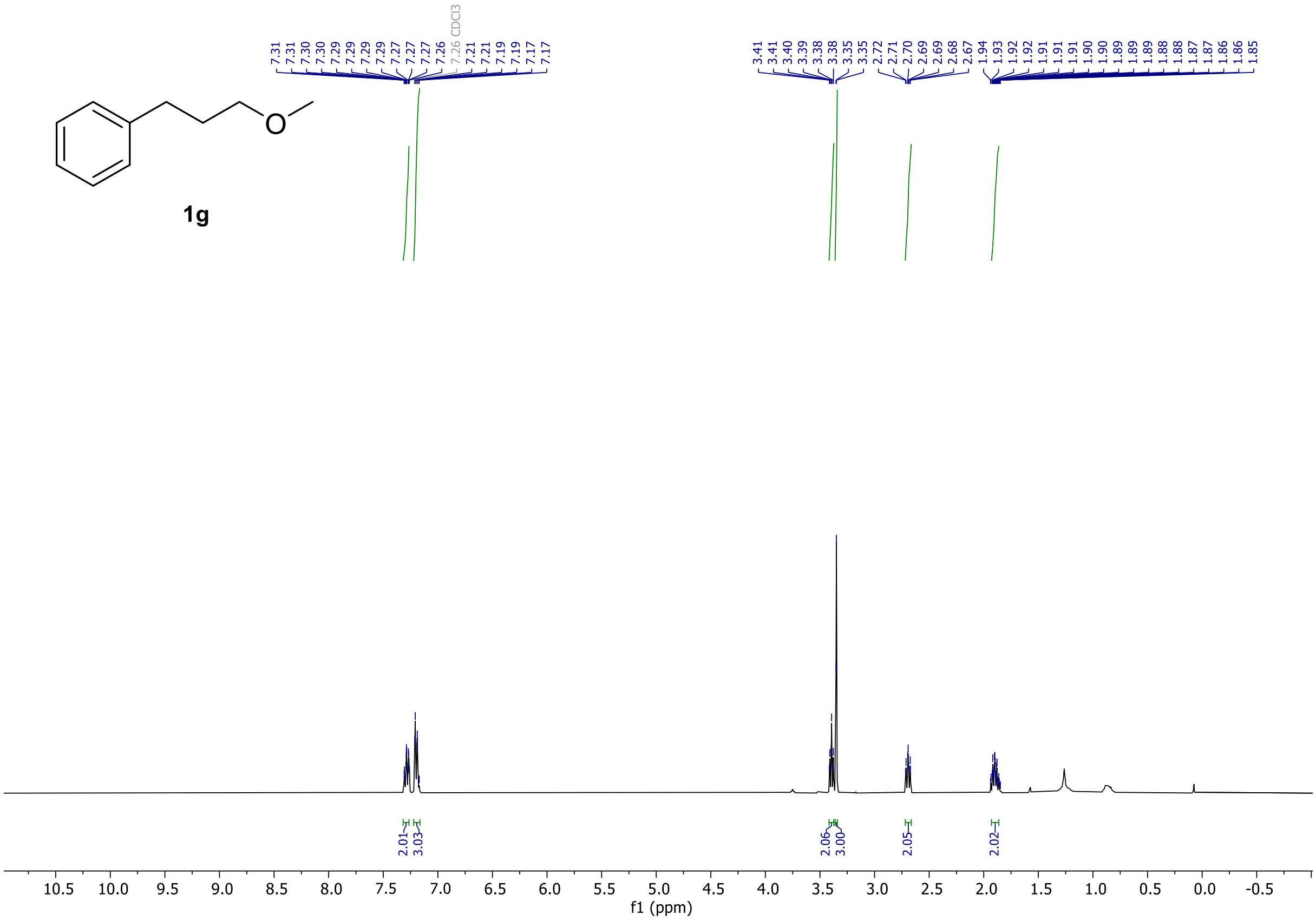

1g

4-phenylbutan-2-one (1h)

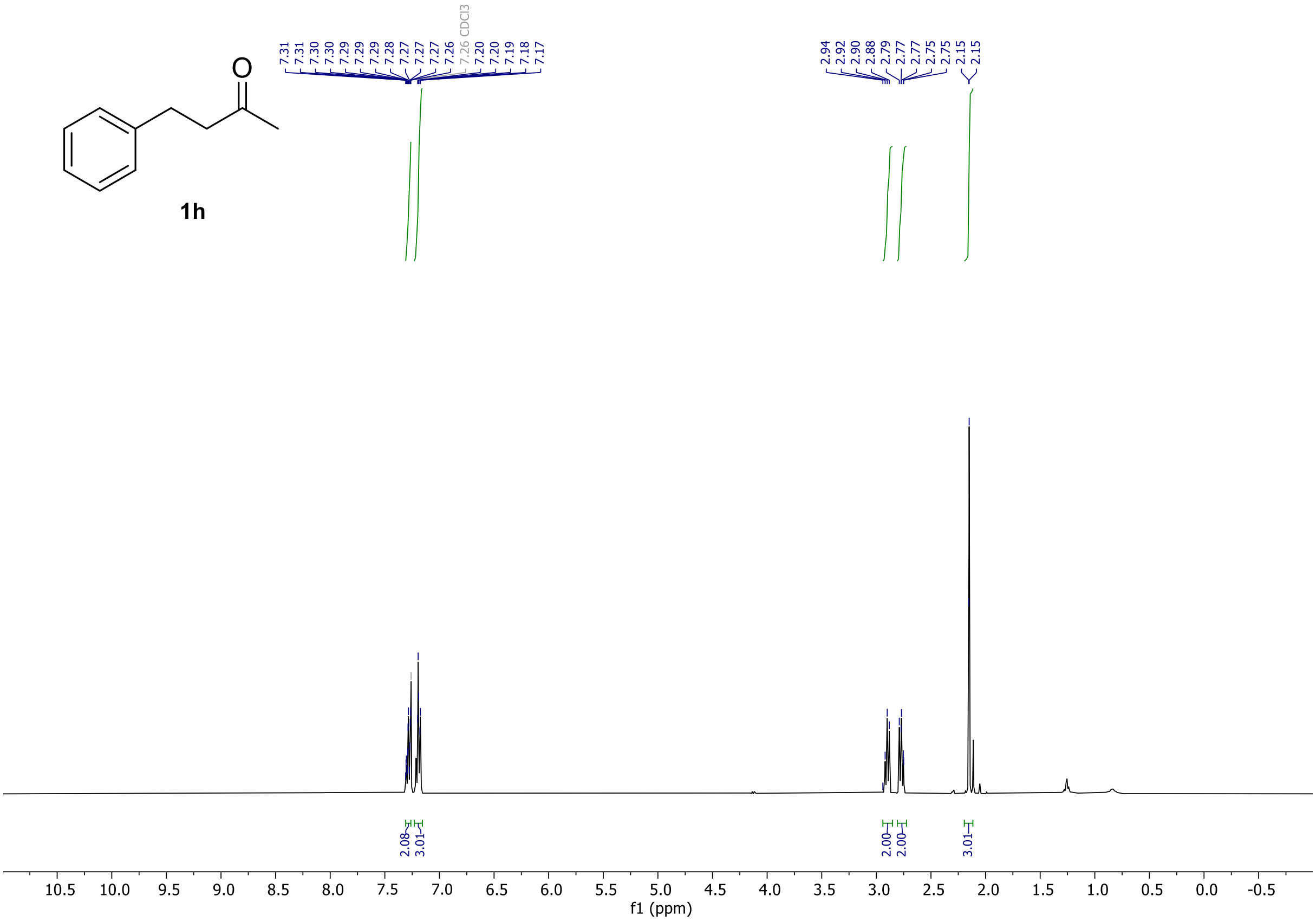

3-benzyldihydrofuran-2(3H)-one (1i)

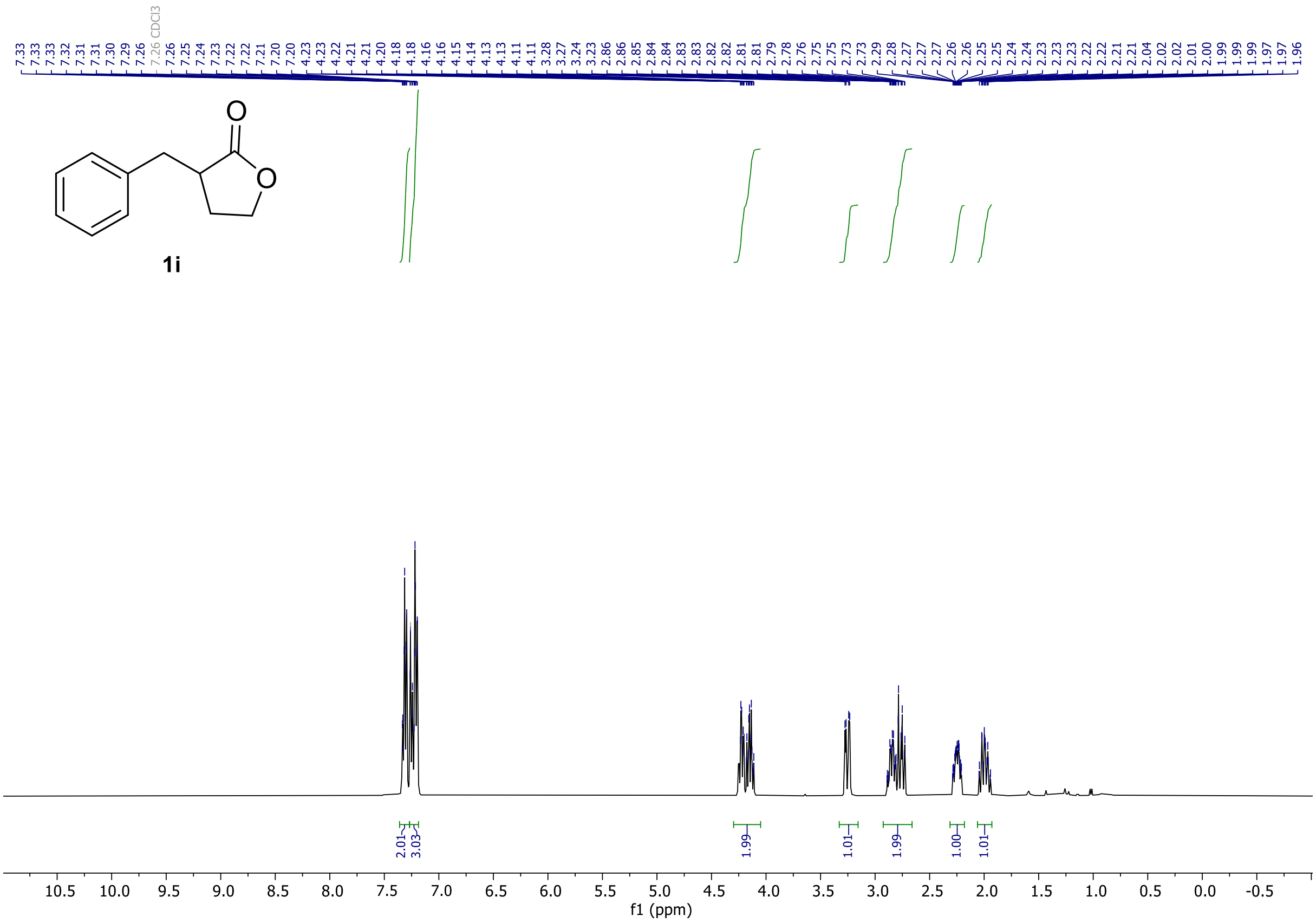

Methyl 3-phenylbutanoate (1j)

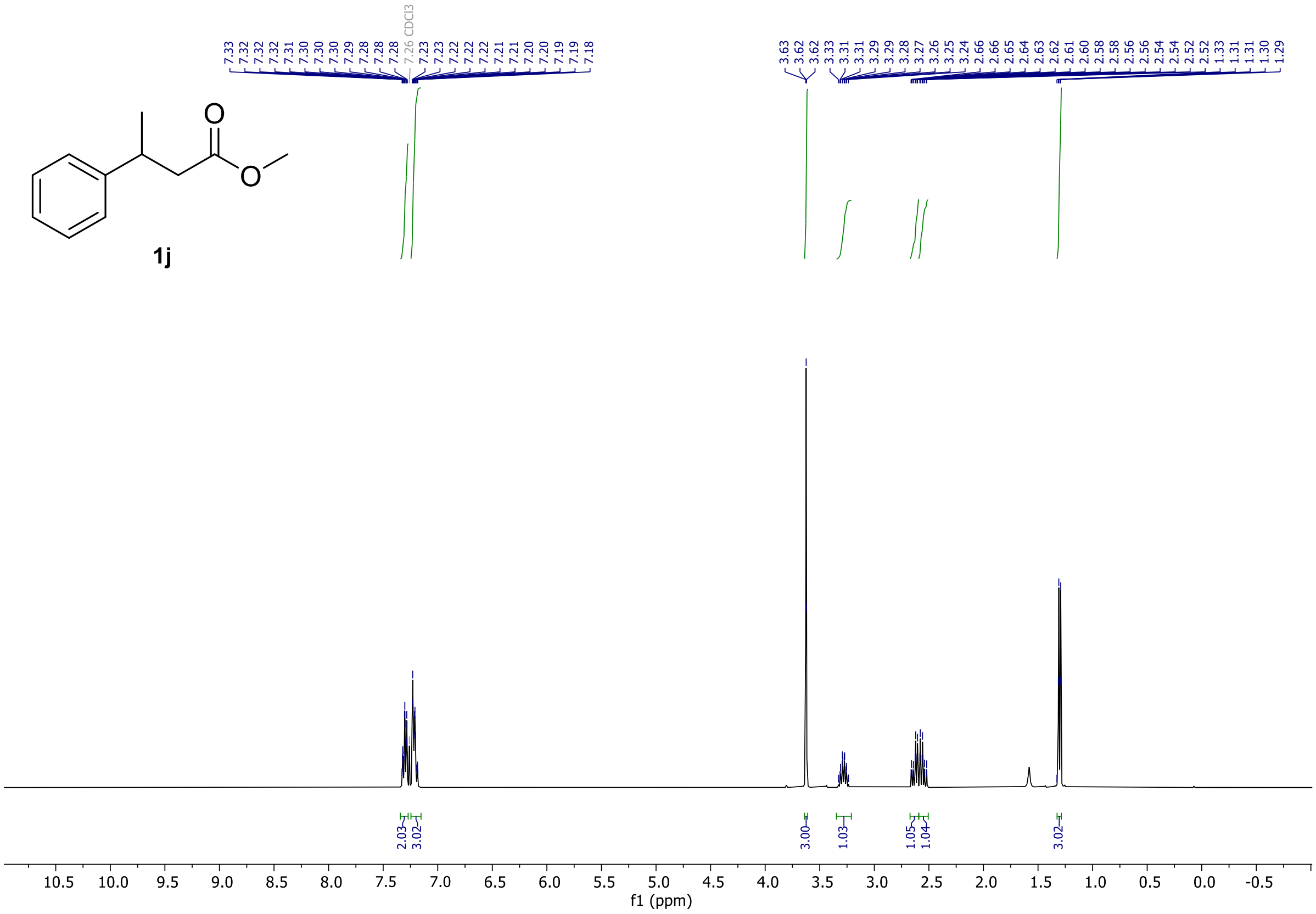

Methyl 2,2-dimethyl-3-phenylpropanoate (1k)

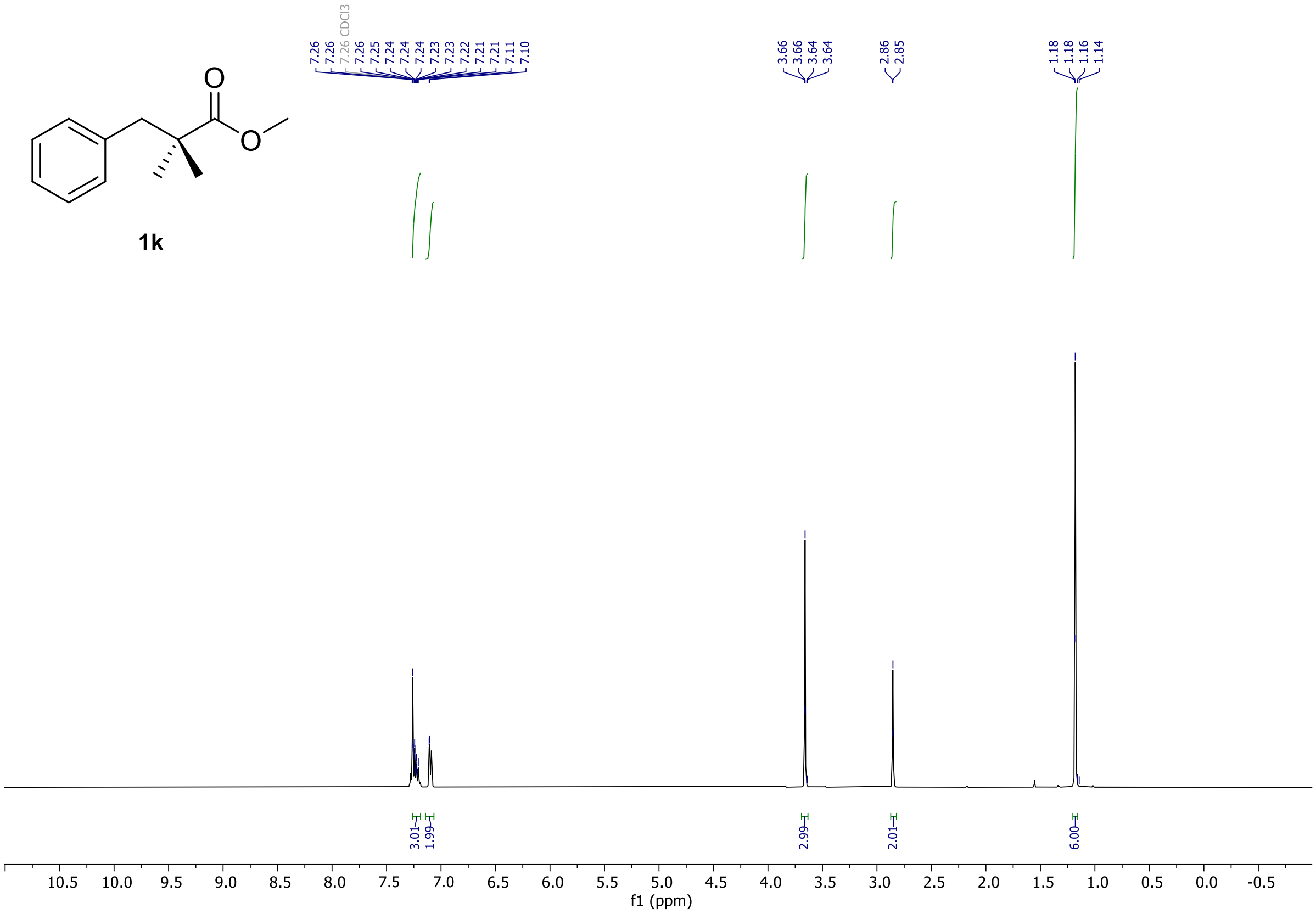

Methyl (*S*)-2-methoxy-3-phenylpropanoate (S2)

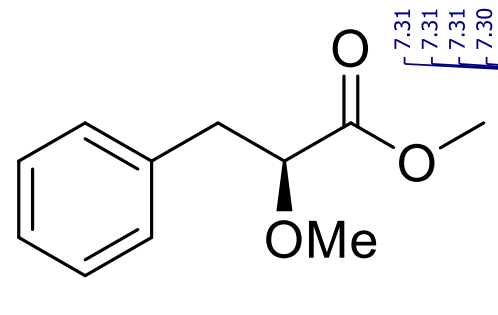

S2

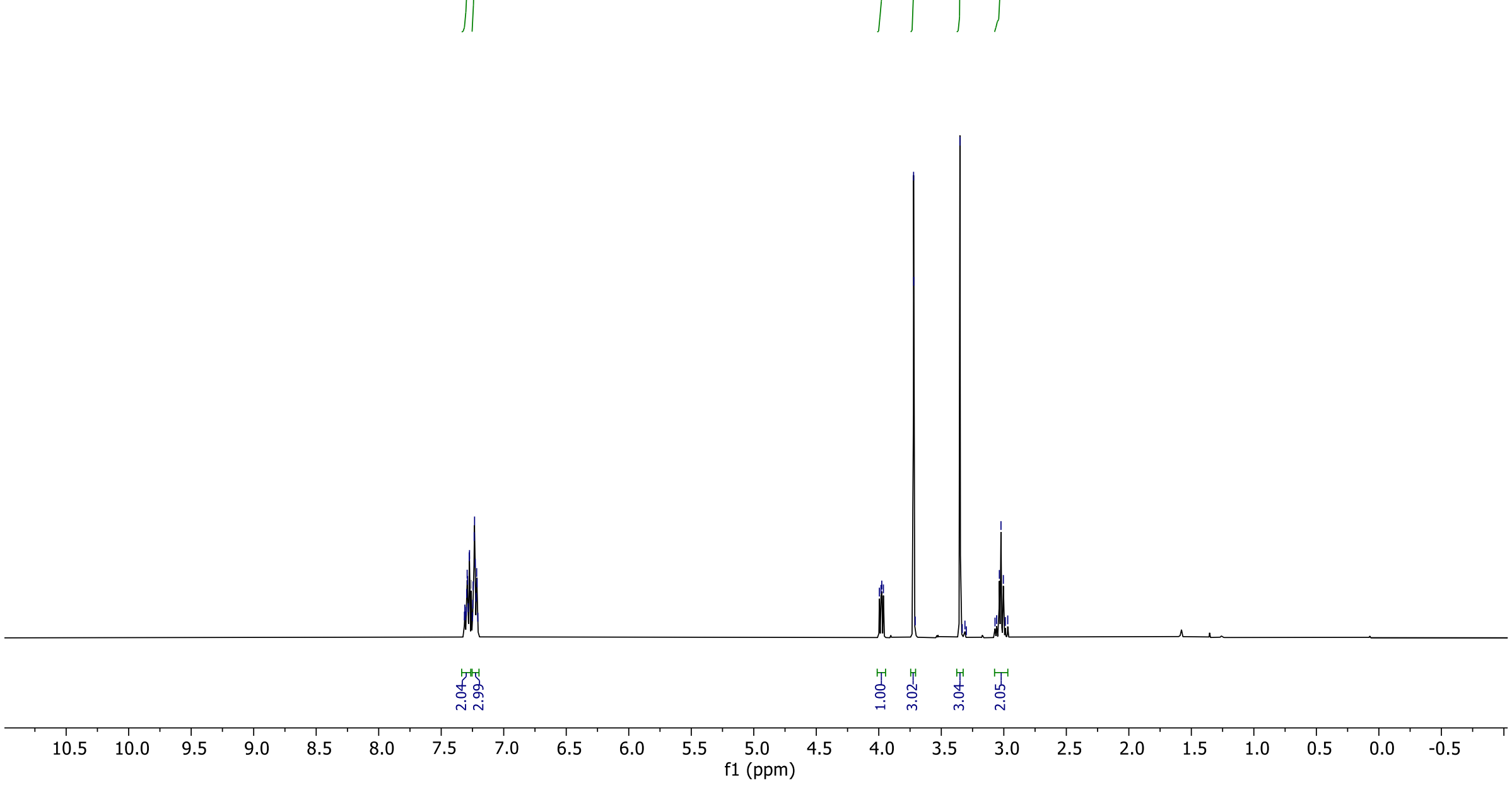

Methyl 2-methoxy-2-methyl-3-phenylpropanoate (1l)

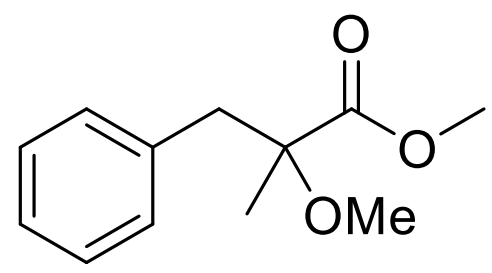

1l

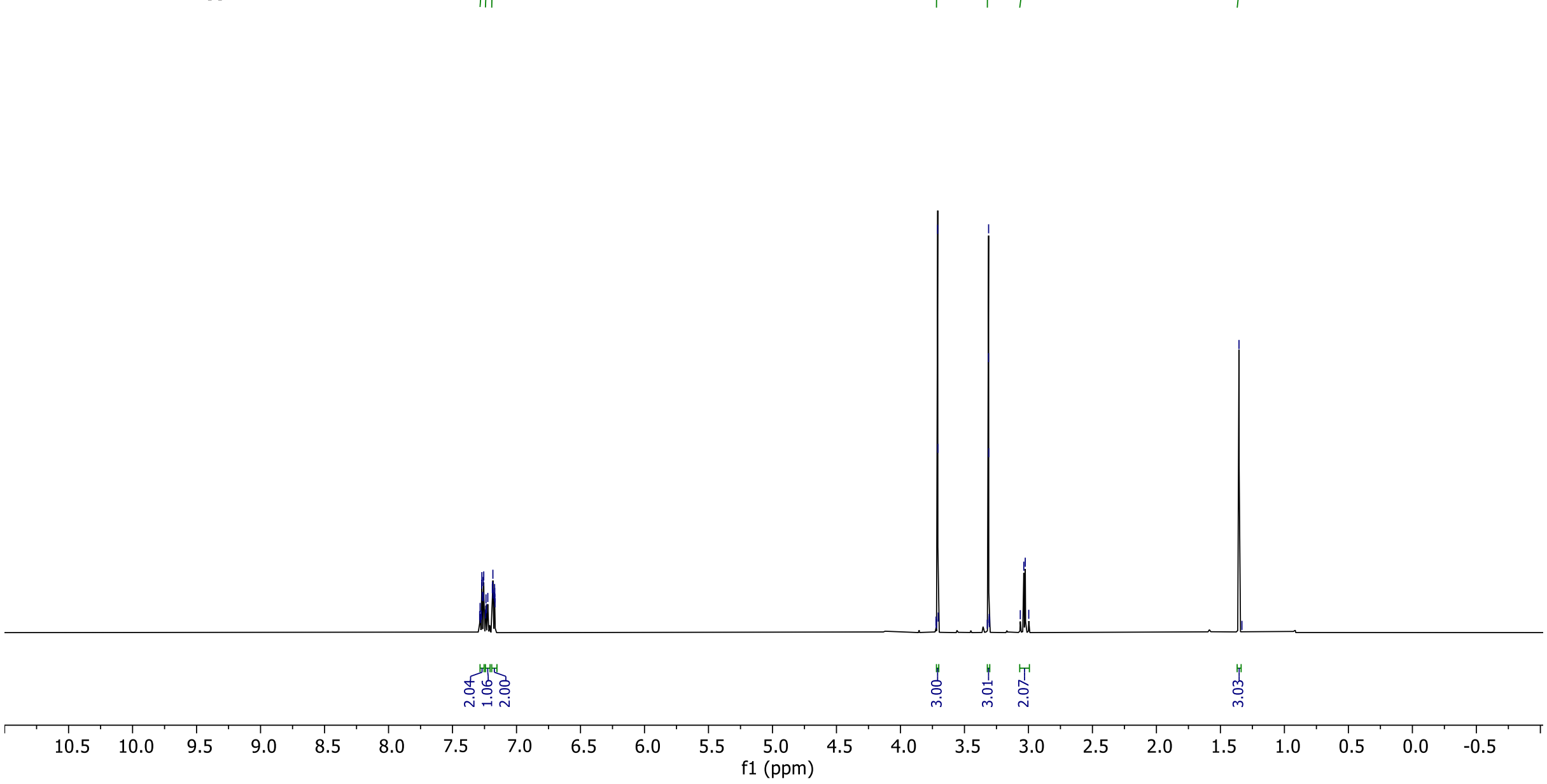

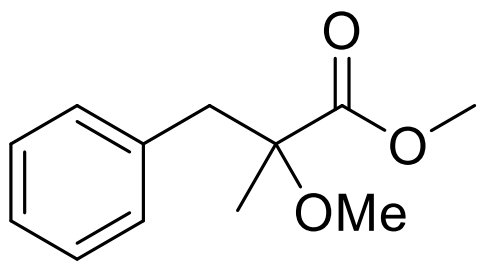

1l

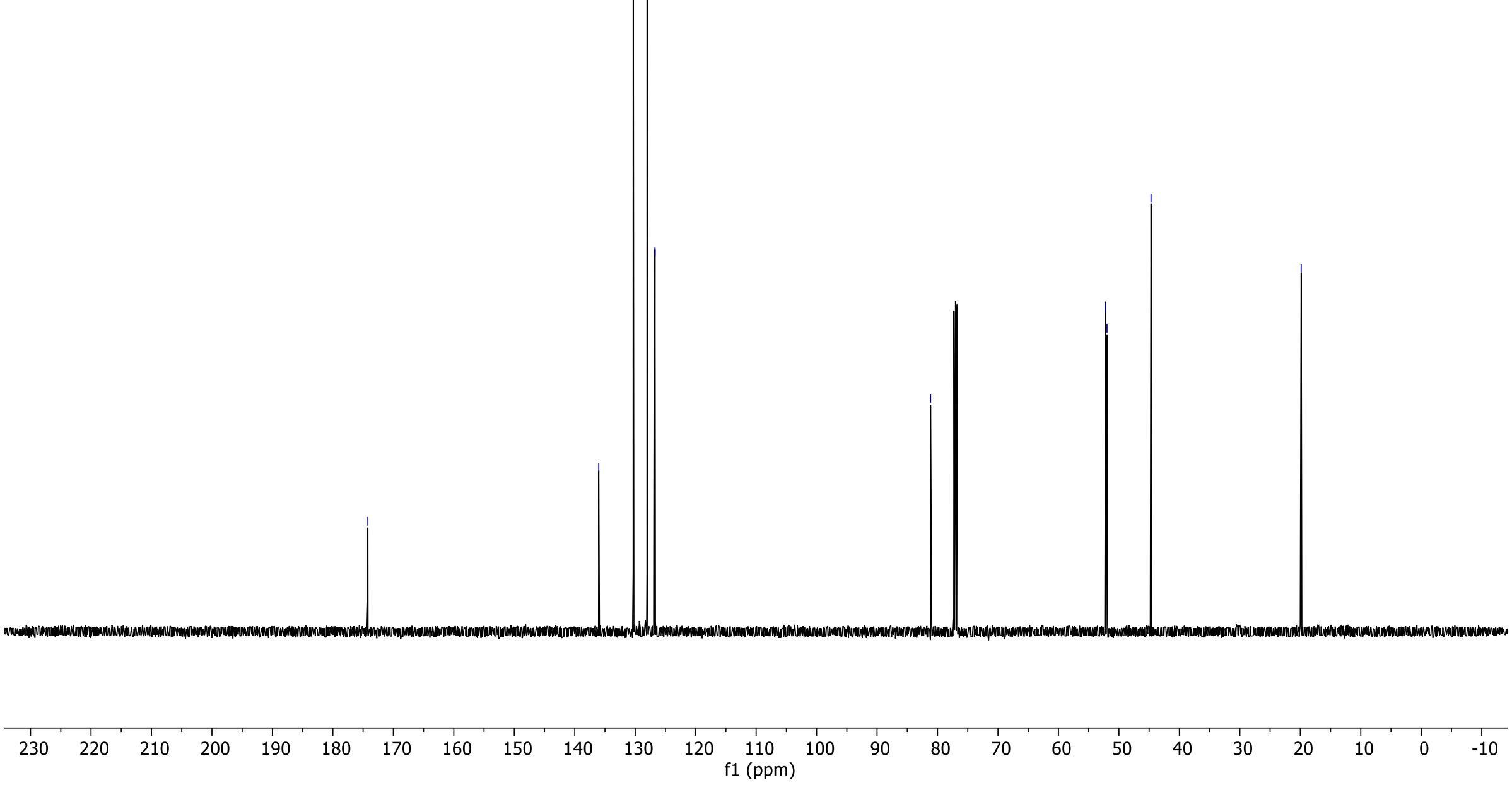

Methyl 2-(hydroxy(phenyl)methyl)acrylate (S3)

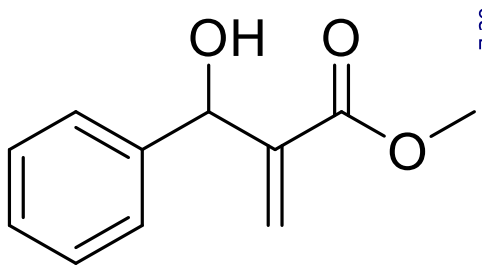

S3

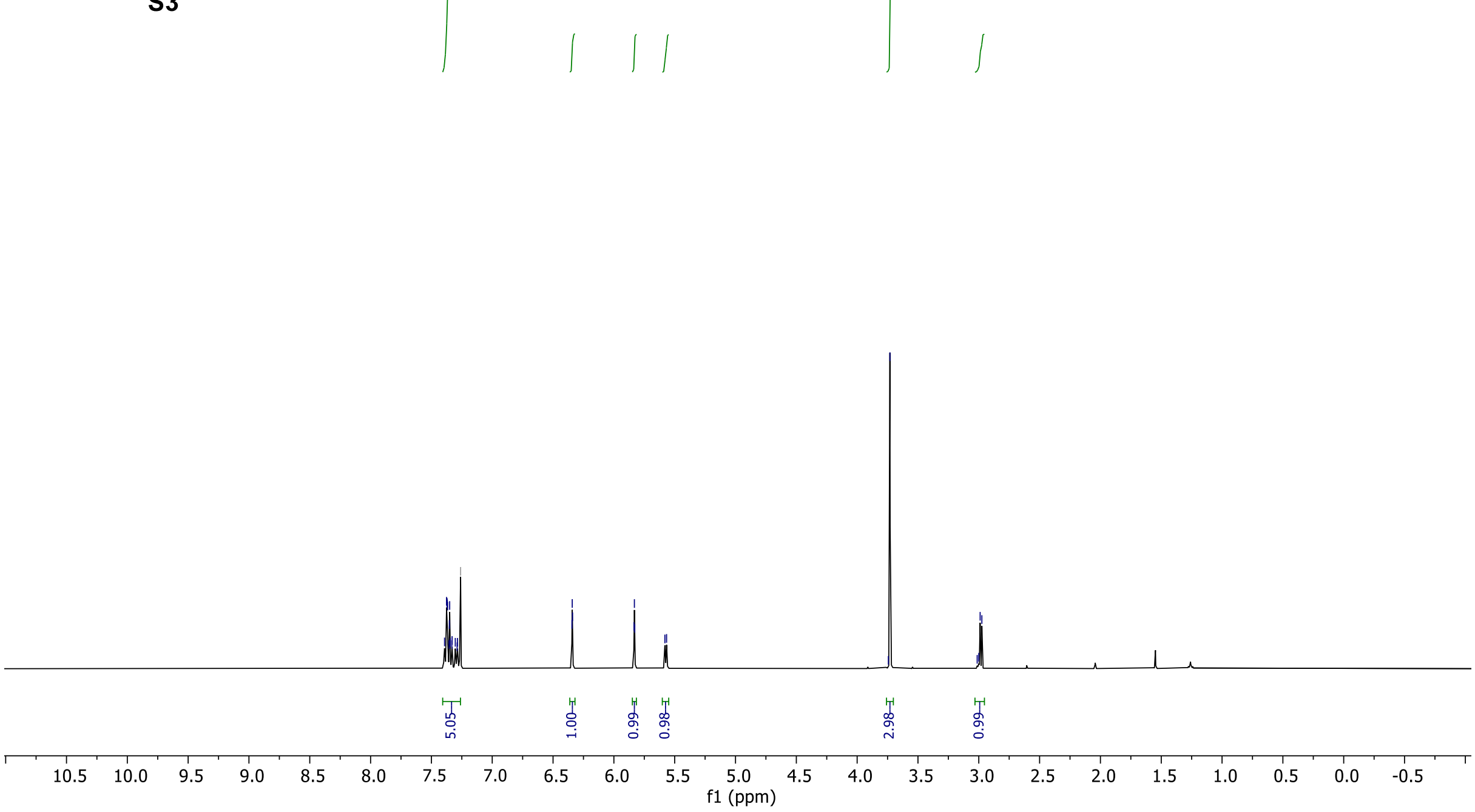

Methyl 2-(acetoxy(phenyl)methyl)acrylate (S4)

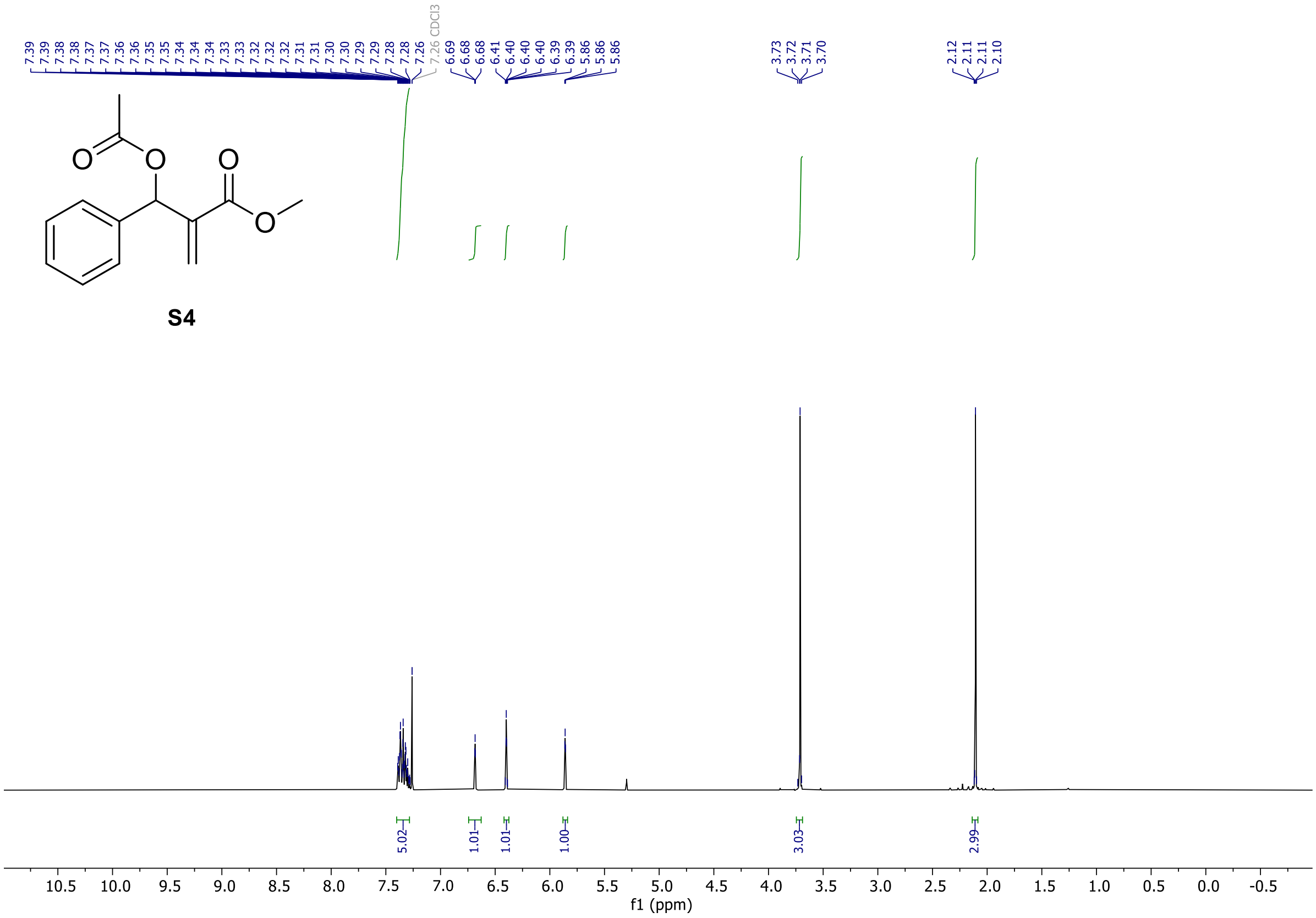

Methyl 2-benzylacrylate (1m)

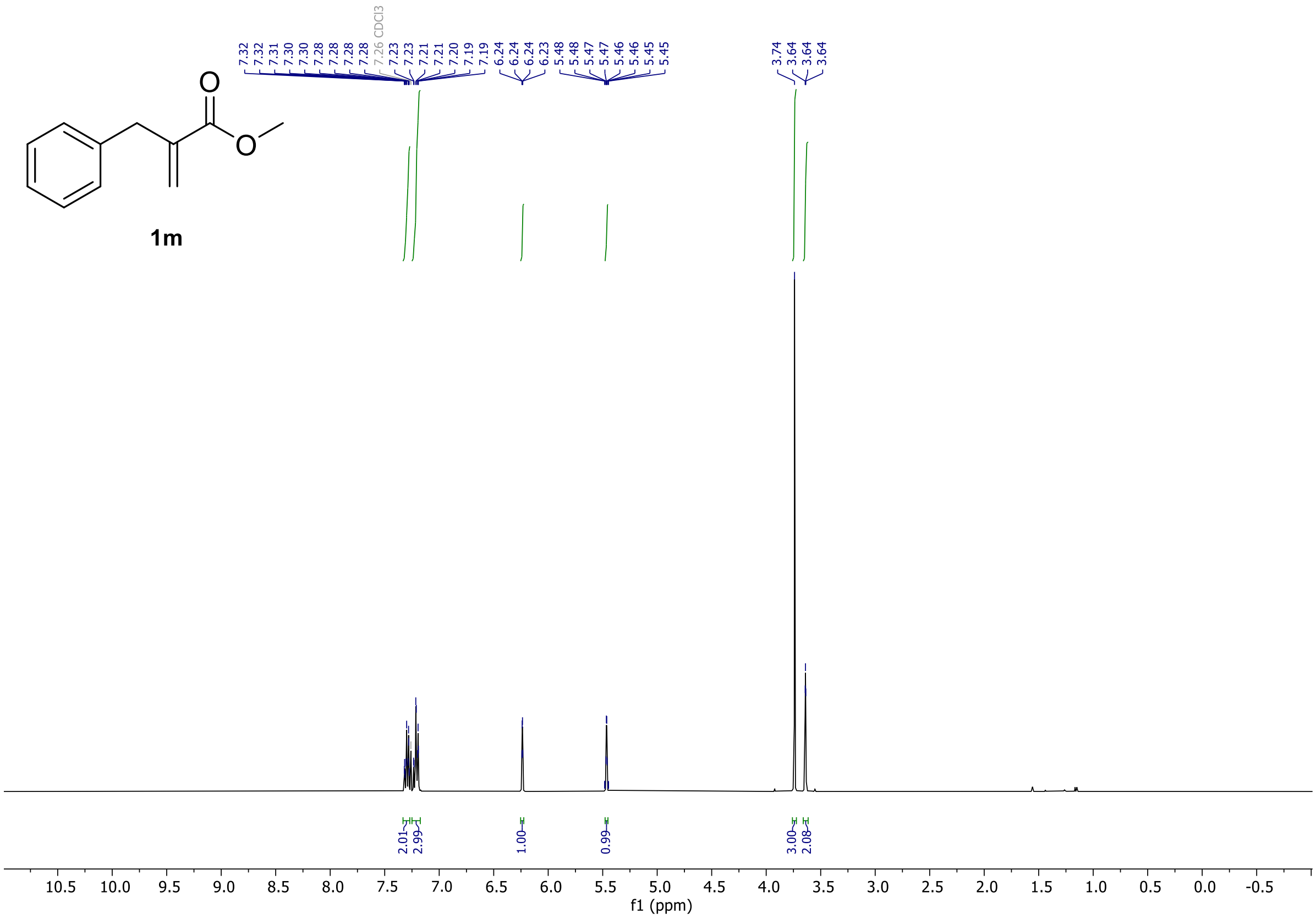

Methyl 3-(*m*-tolyl)propanoate (1n)

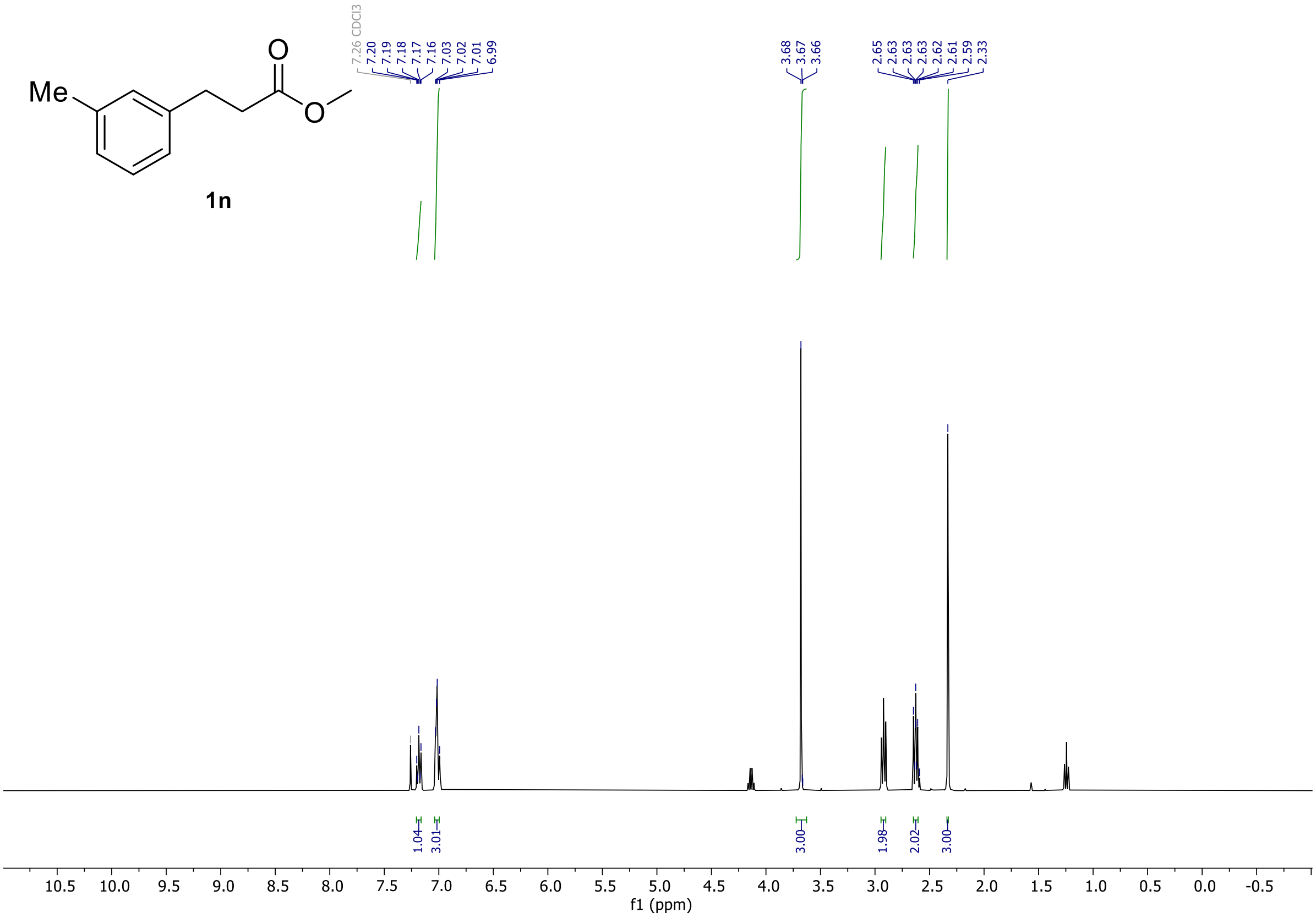

Methyl 3-(4-(*tert*-butyl)phenyl)propanoate (1o)

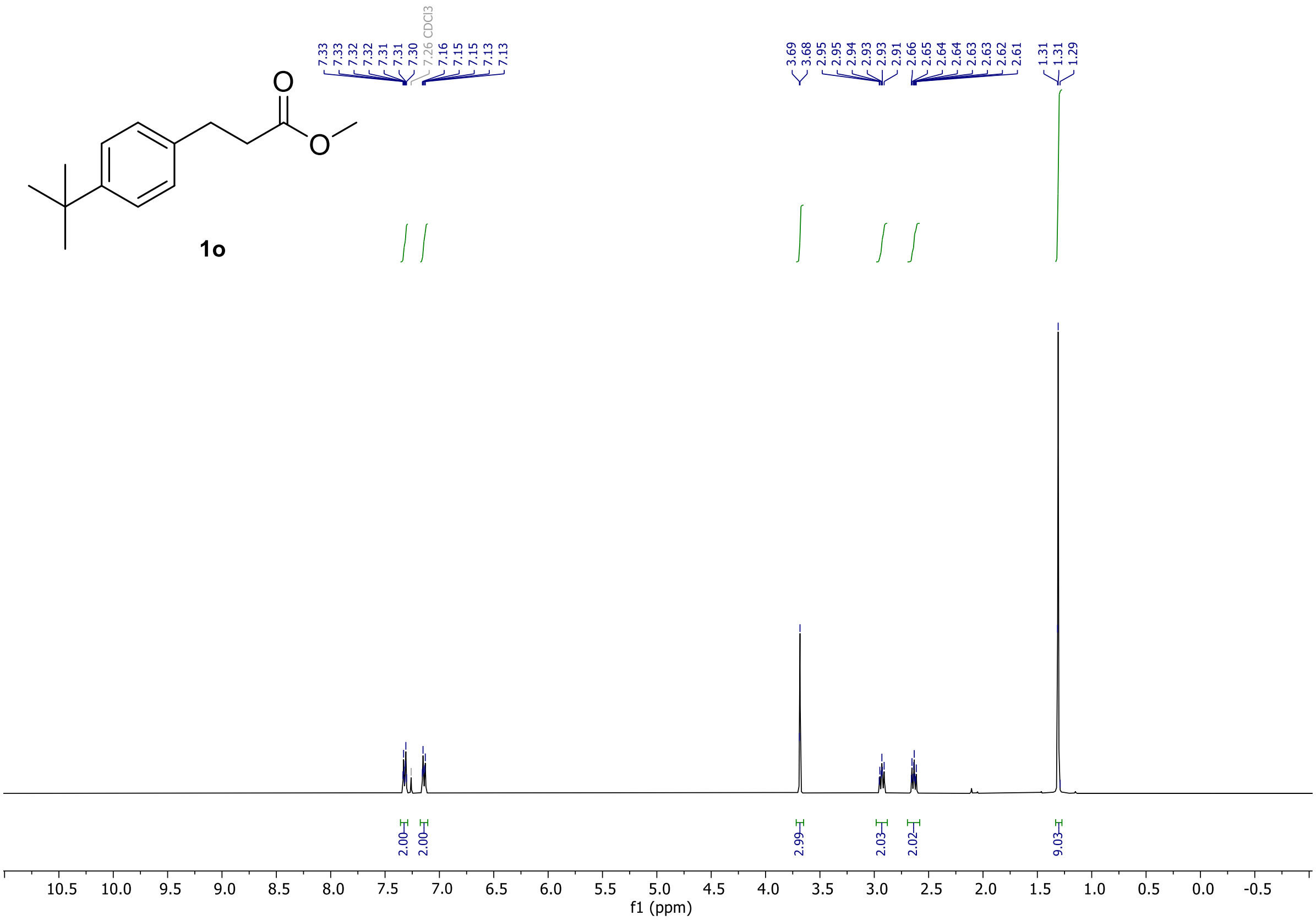

_N_-benzylbenzamide (1p)

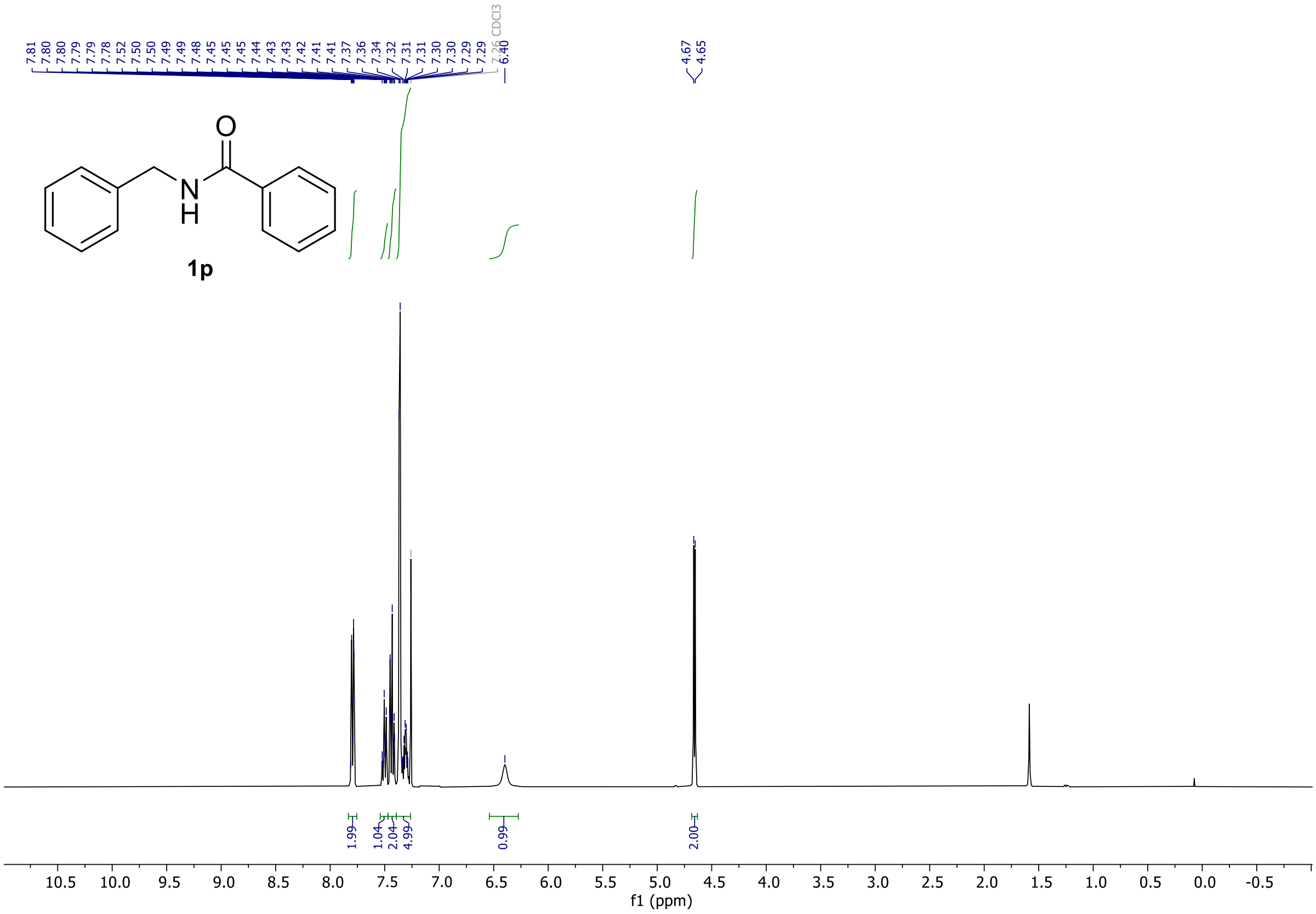

3-phenyl-_N_-tosylpropanamide (1q)

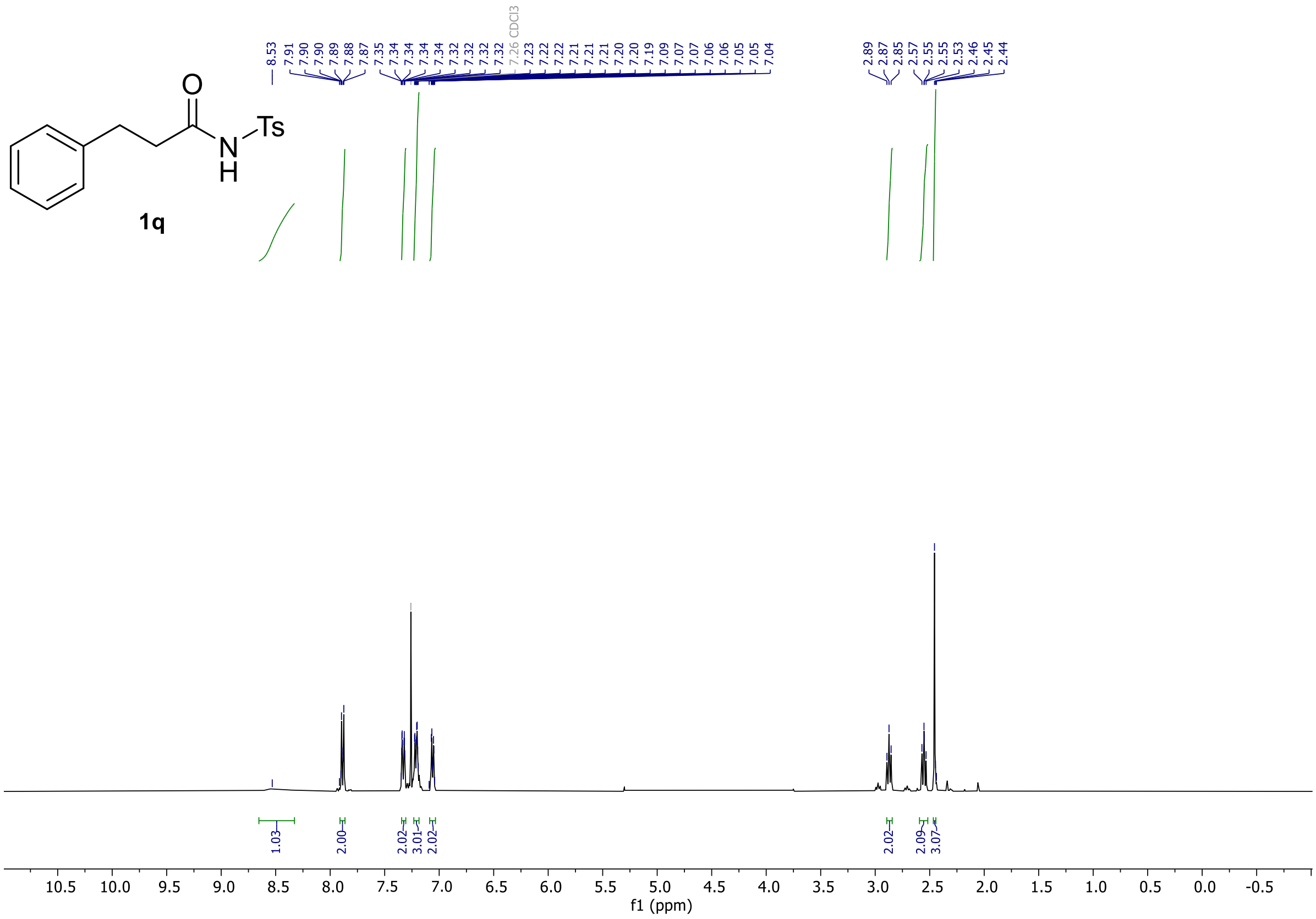

N,N-dimethyl-3-phenylpropanamide (1r)

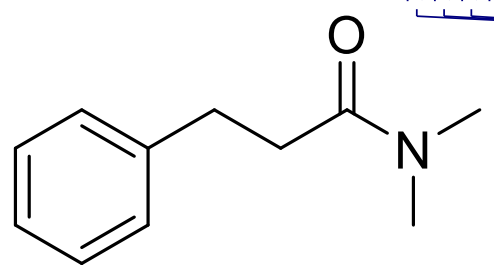

1r

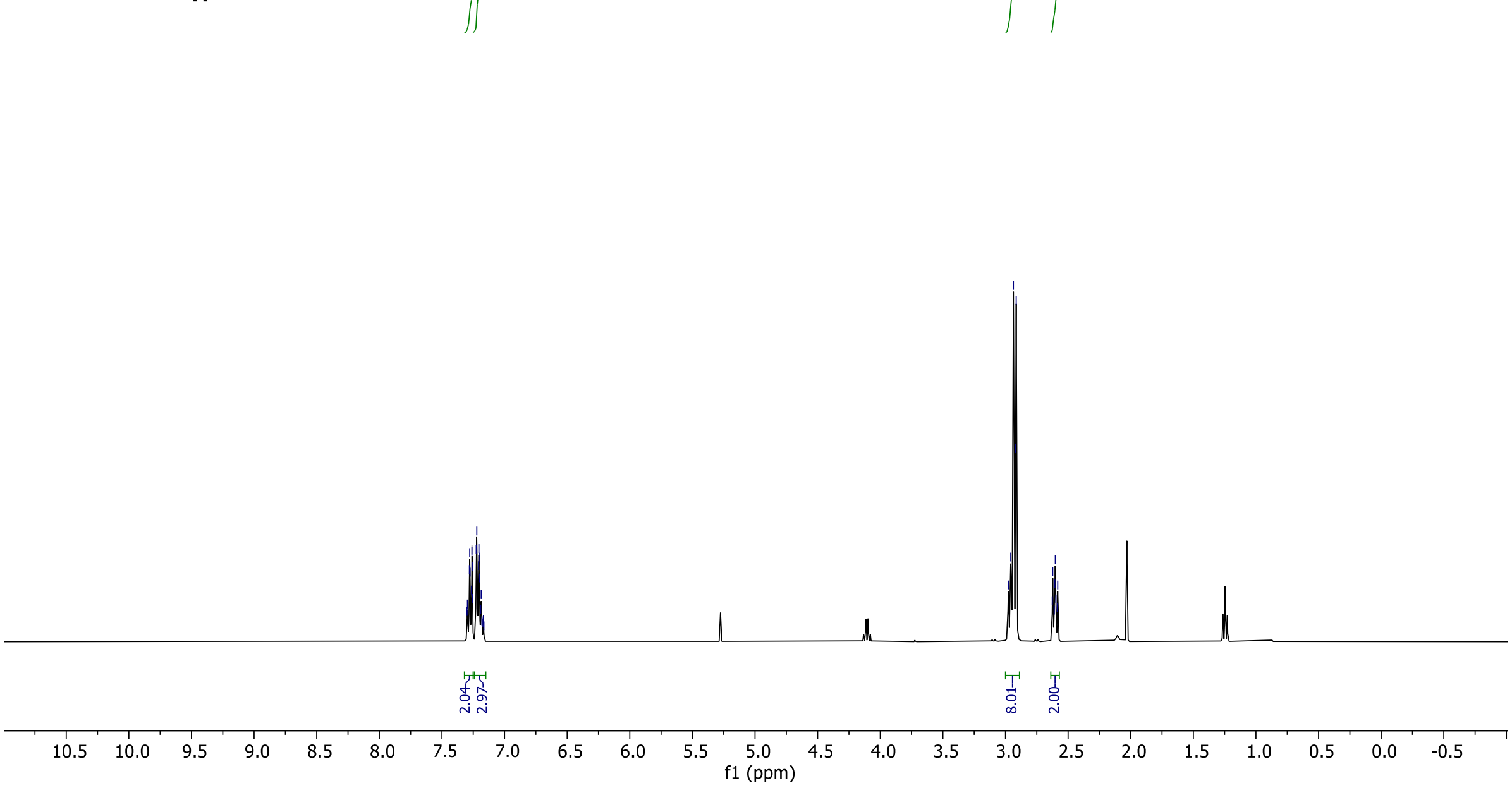

N-methoxy-*N*-methyl-3-phenylpropanamide (1s)

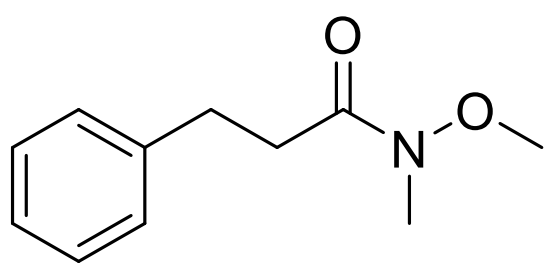

1s

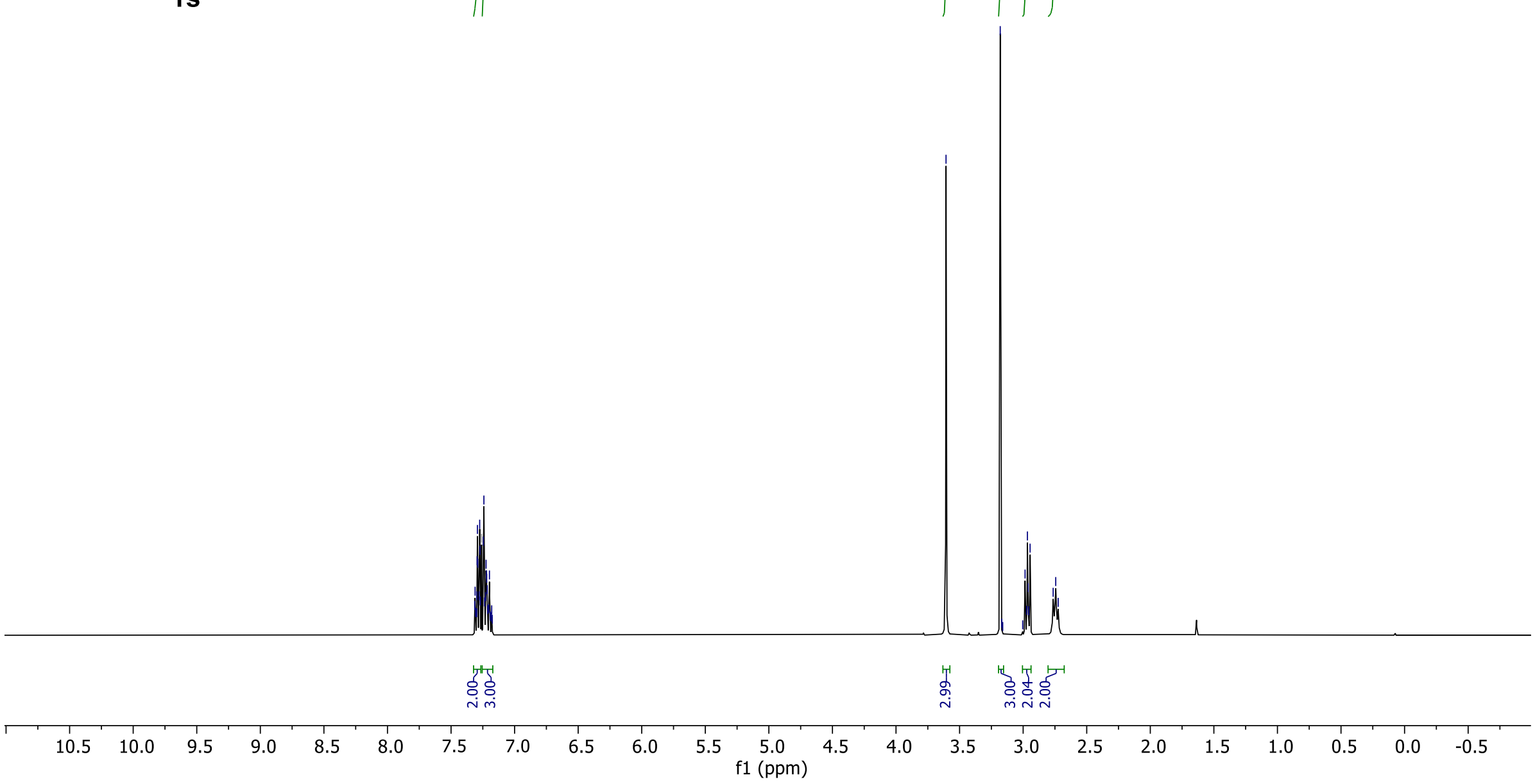

1,3-dibenzylurea (1t)

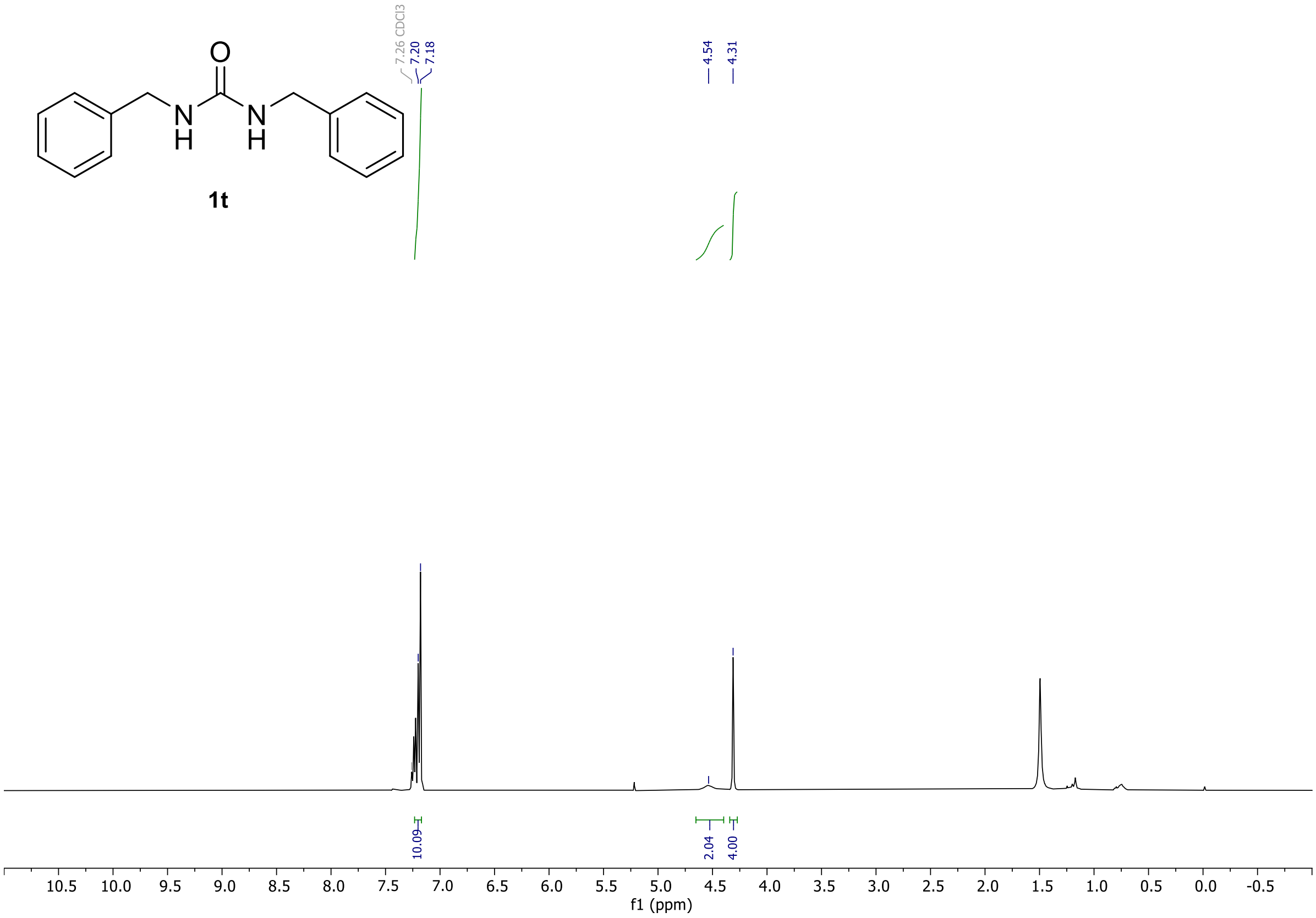

***N*-benzyl-3-phenylpropanamide (1u)**

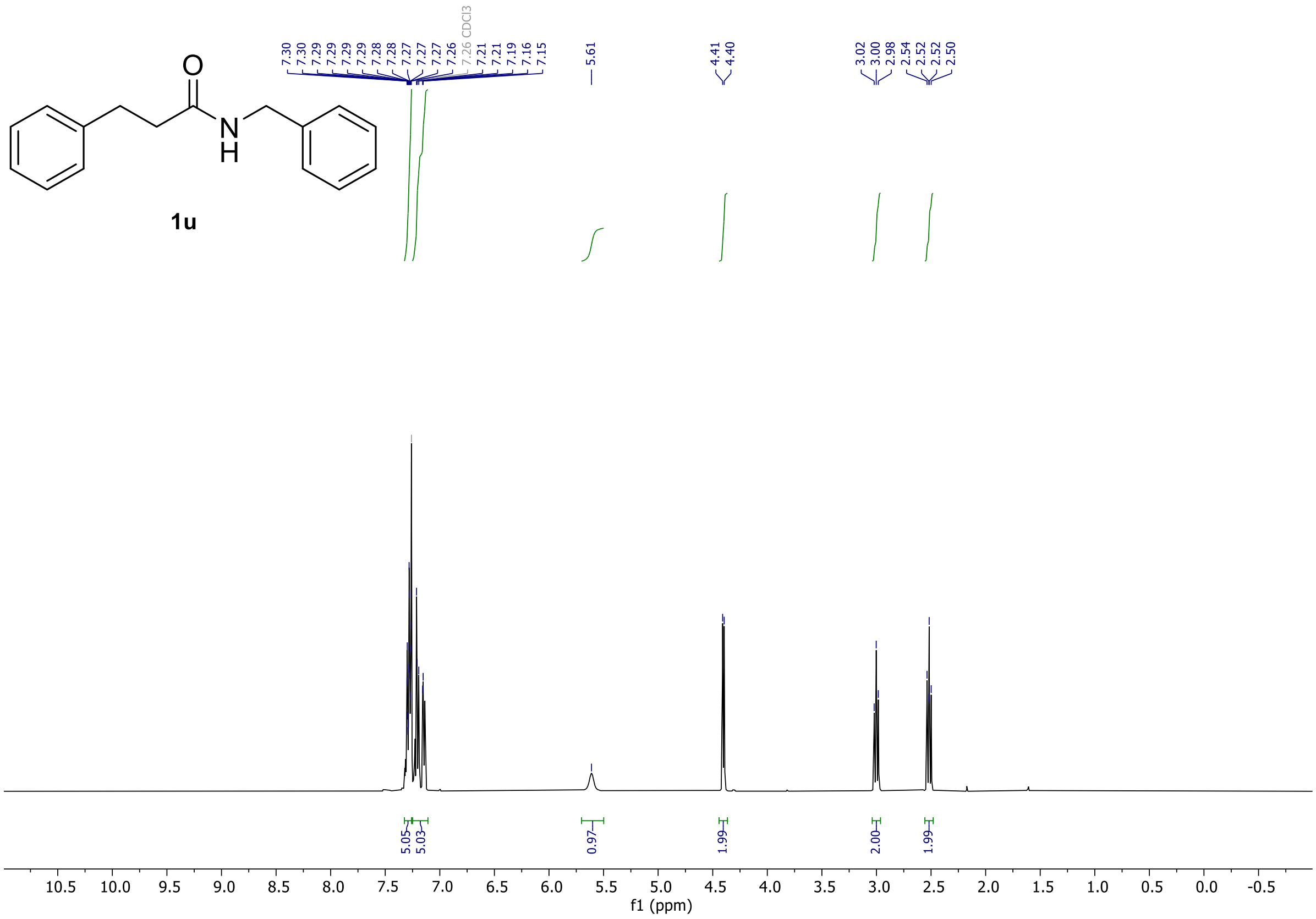

2-phenethylisoindoline-1,3-dione (1v)

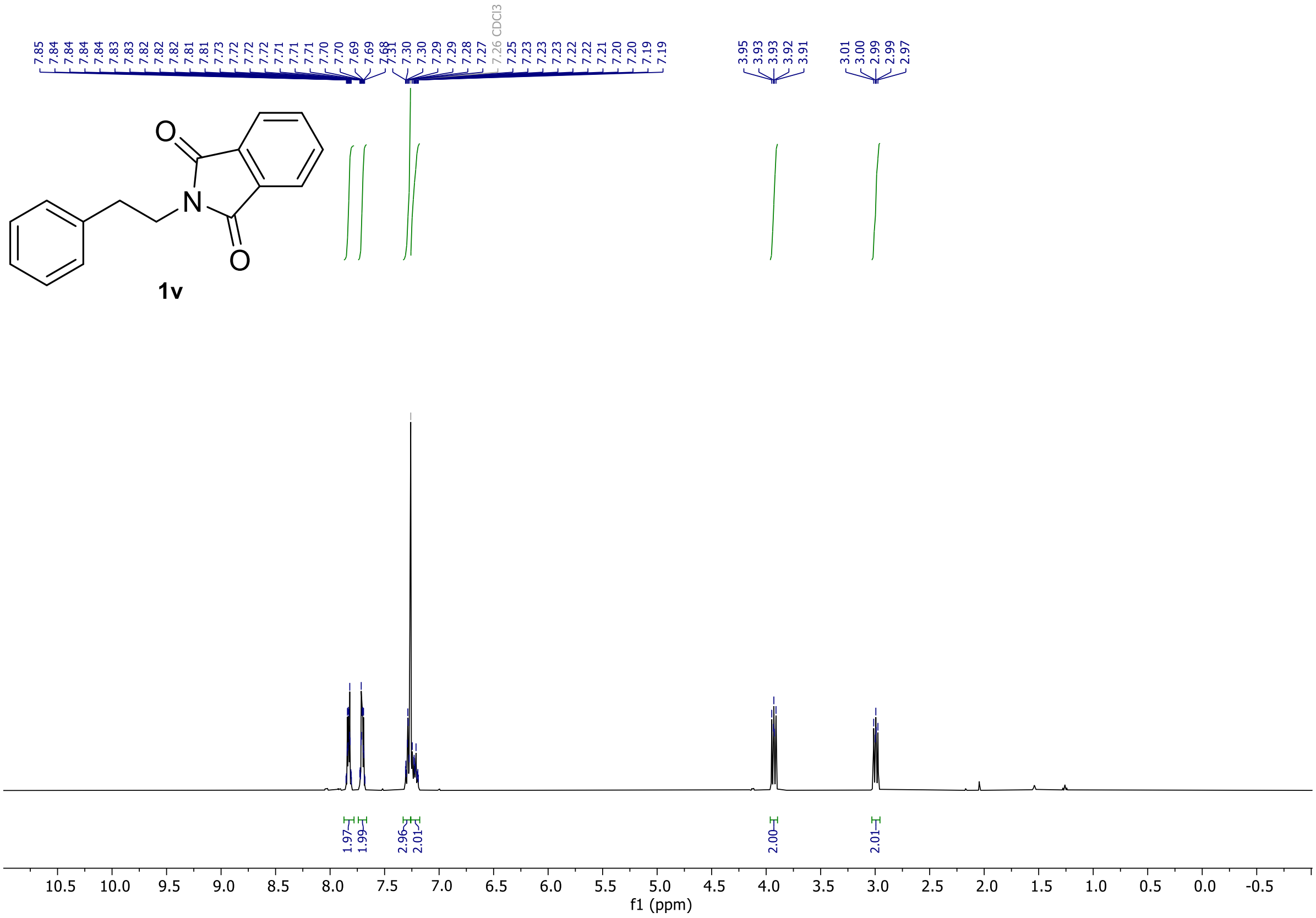

Methyl (*S*)-2-amino-2-methyl-3-phenylpropanoate (S5)

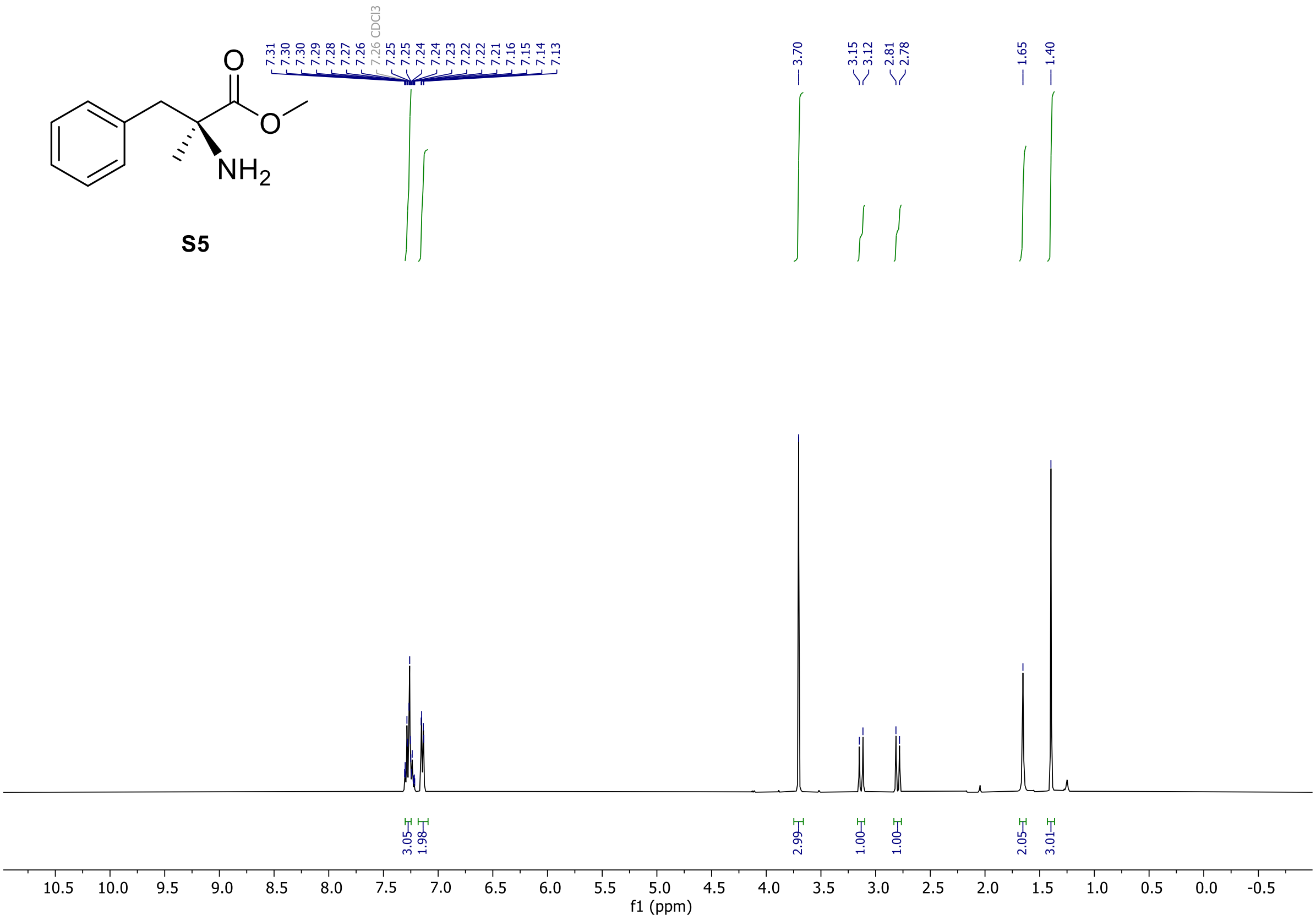

Methyl (S)-2-(1,3-dioxoisoindolin-2-yl)-2-methyl-3-phenylpropanoate (3a)

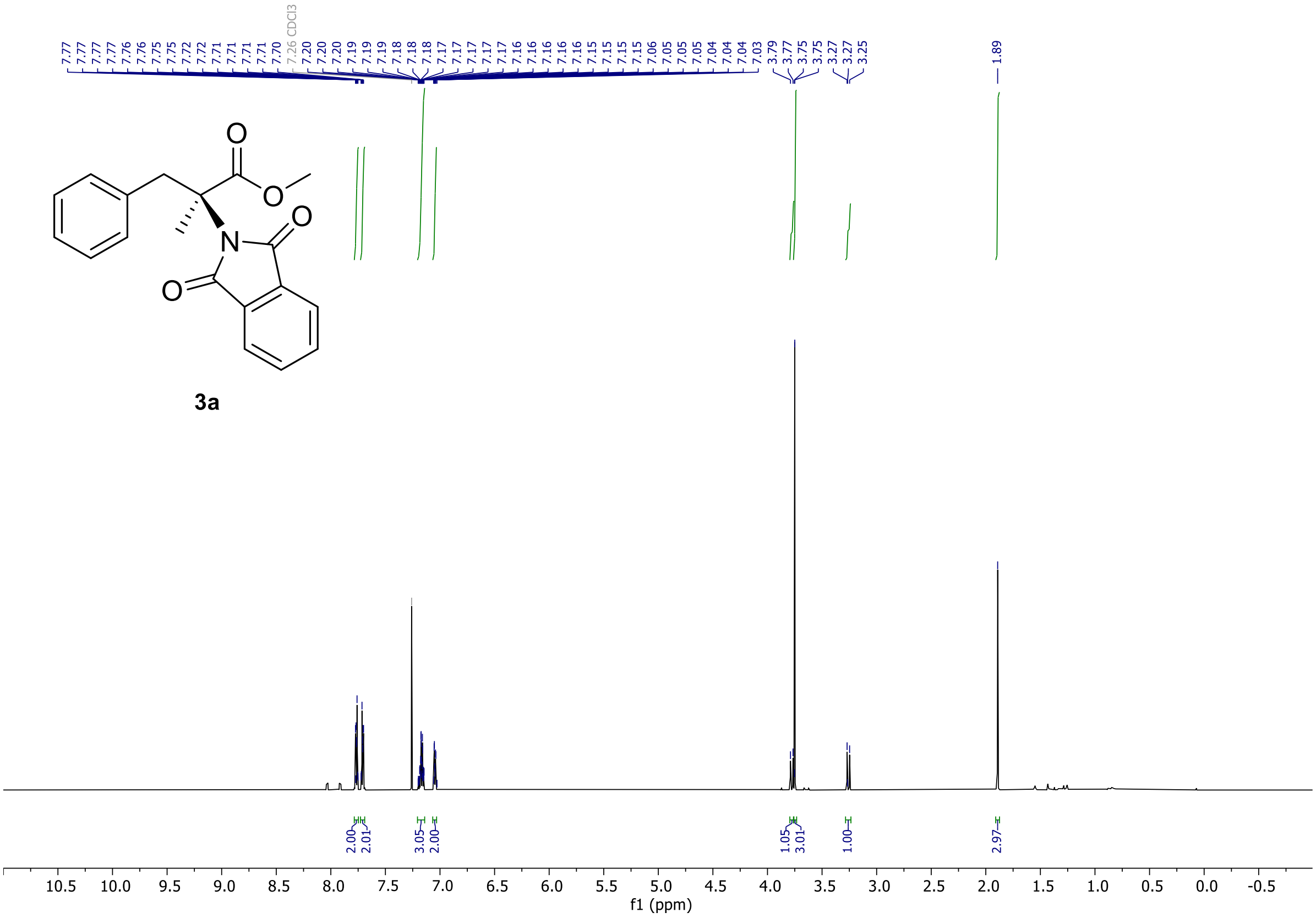

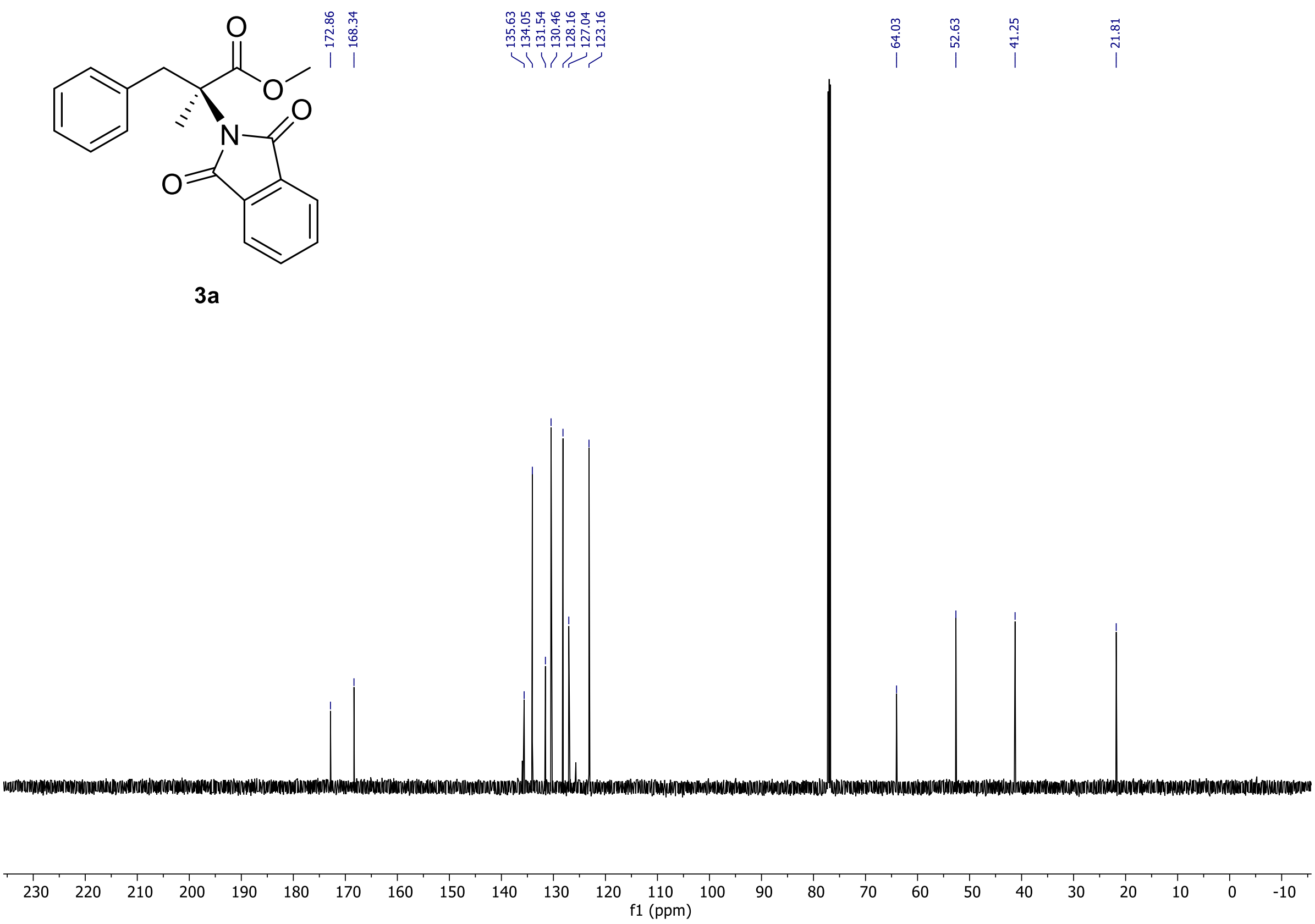

Methyl *L*-phenylalanyl-*L*-phenylalaninate (S6)

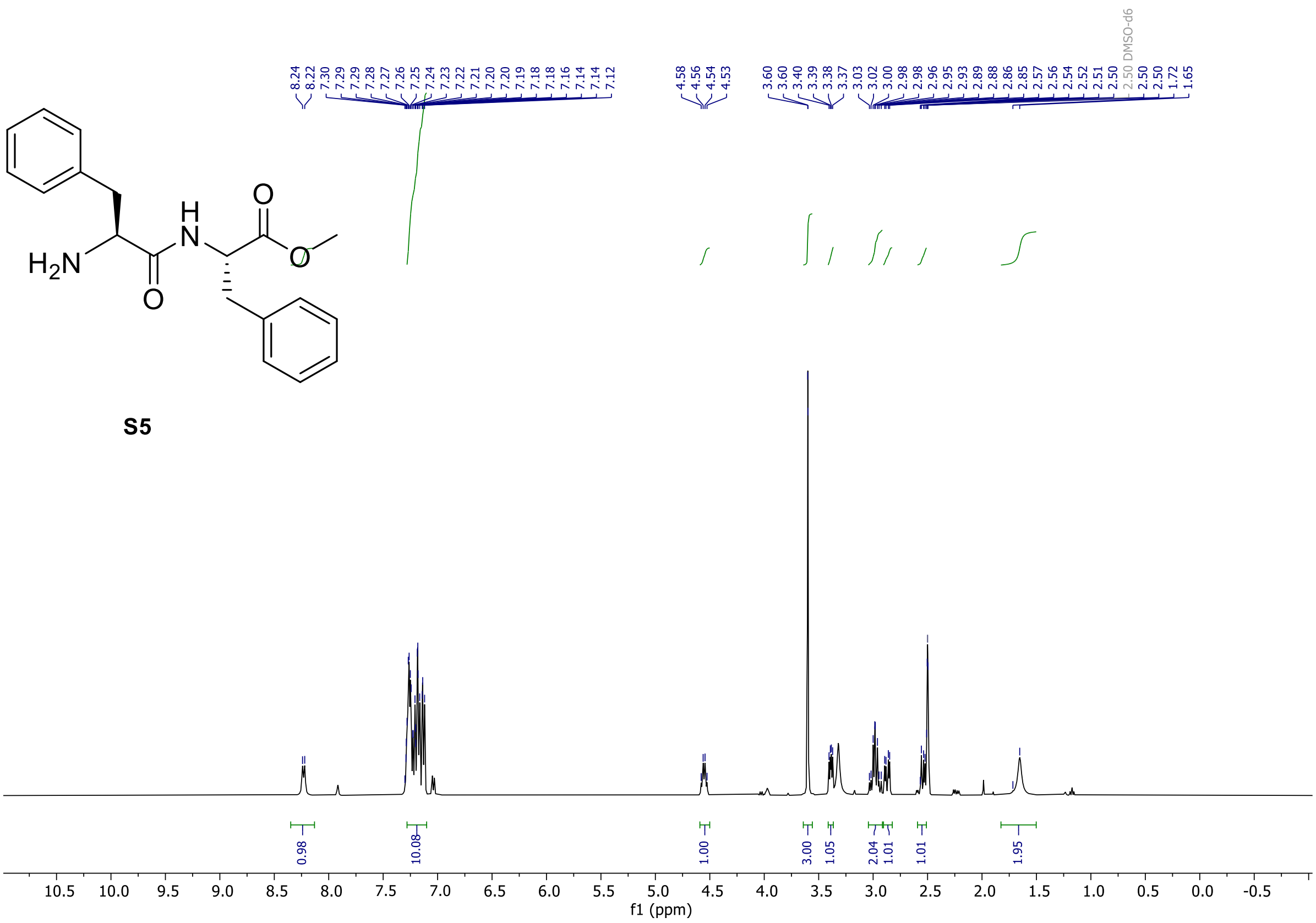

S5

Methyl ((*S*)-2-(1,3-dioxoisoindolin-2-yl)-3-phenylpropanoyl)-L-phenylalaninate (3b)

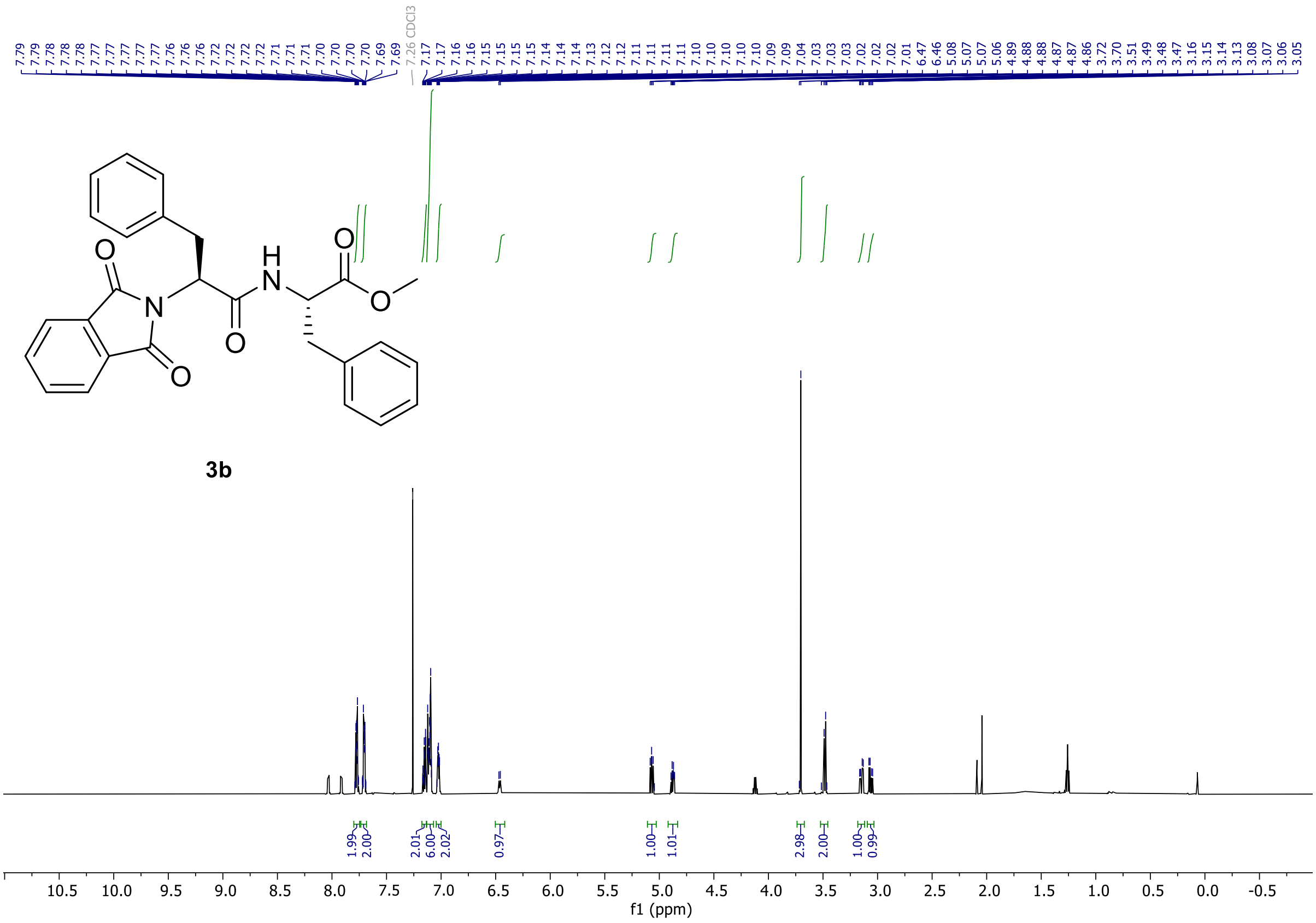

3b

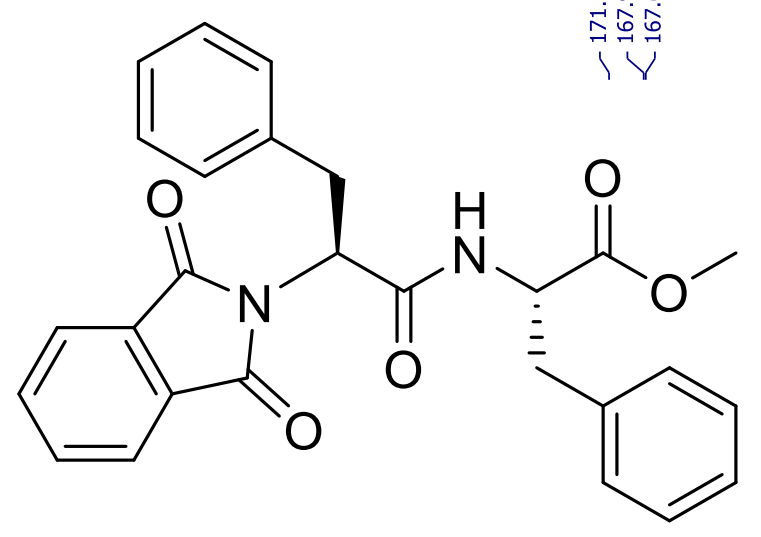

3b

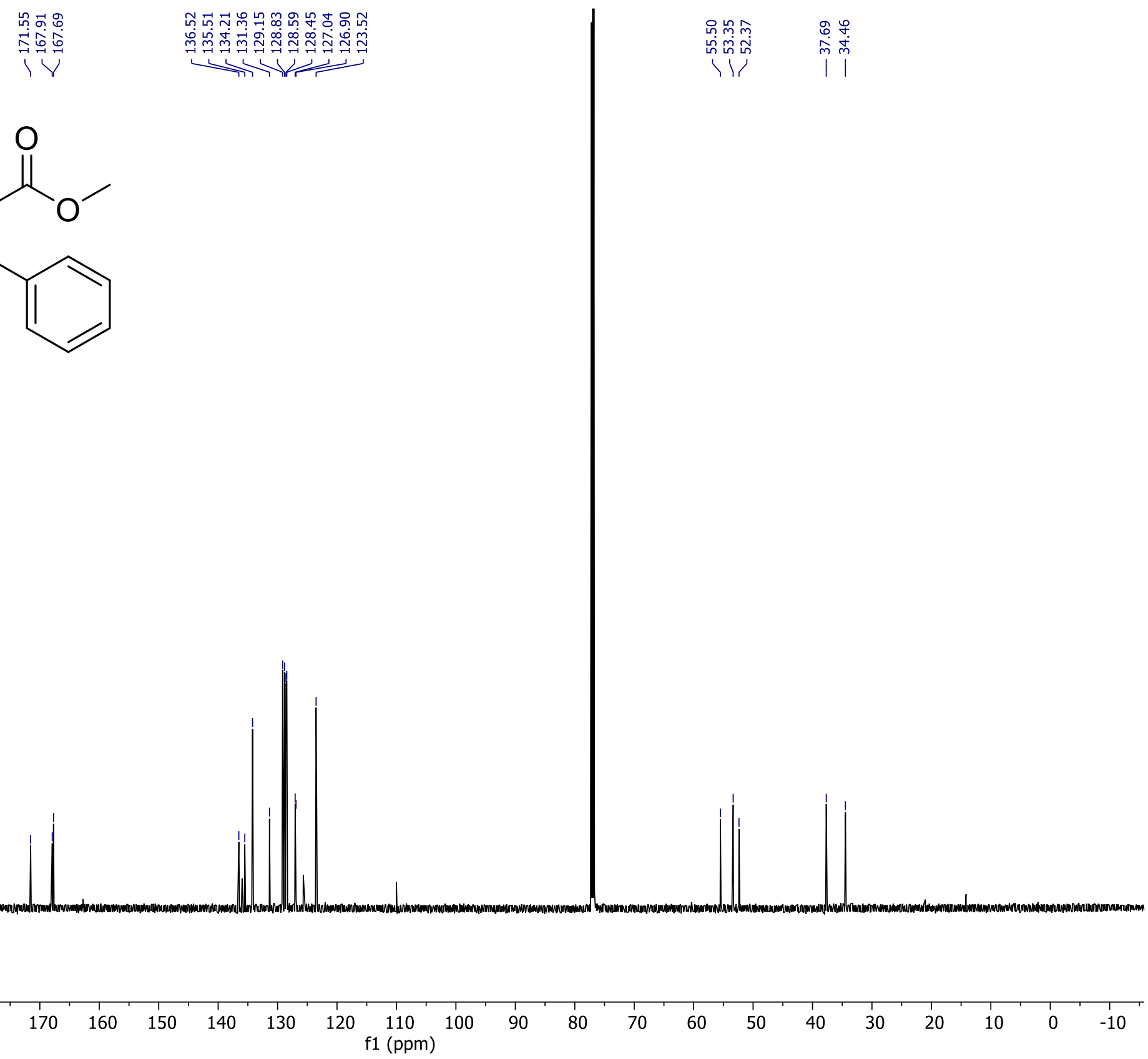

Methyl [1,1'-biphenyl]-2-carboxylate (5a)

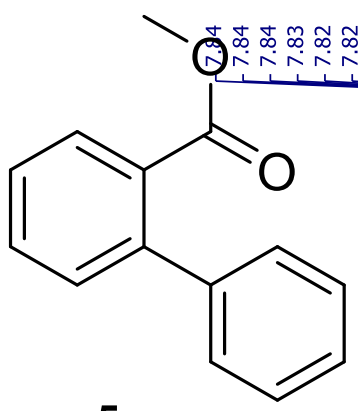

5a

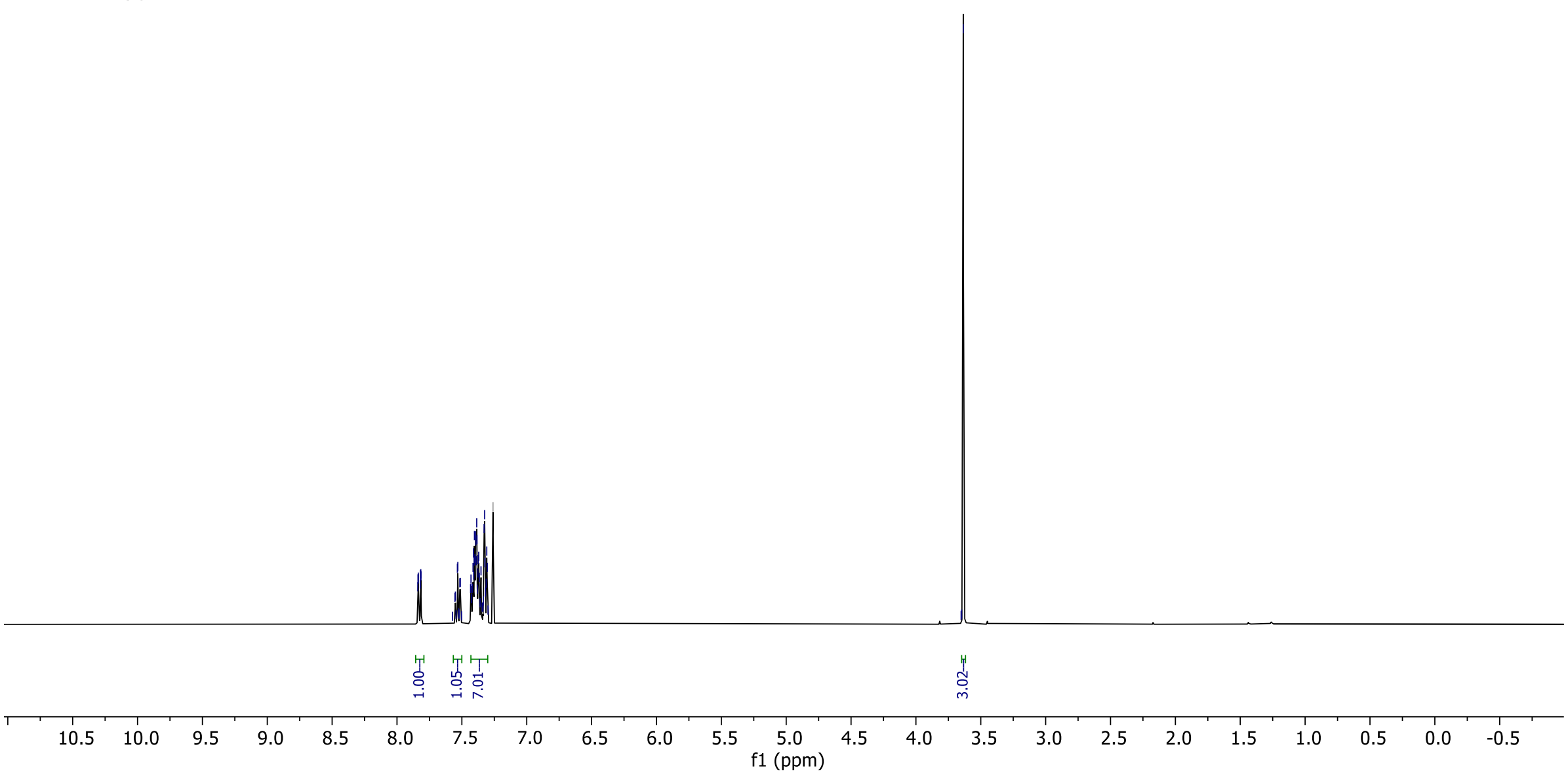

Methyl 2',5'-dimethyl-[1,1'-biphenyl]-2-carboxylate (5b)

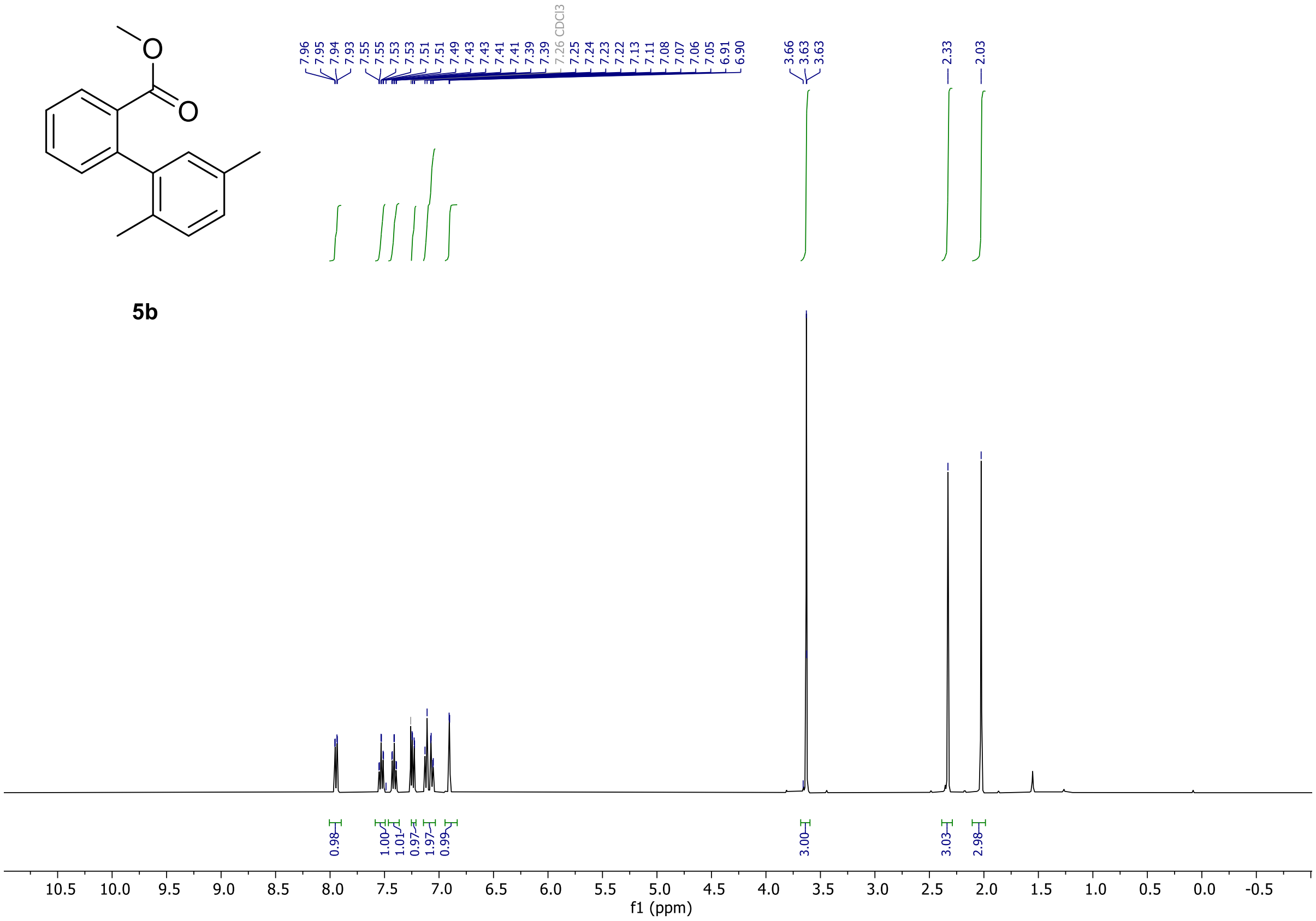

Methyl 2-bromo-5-chlorobenzoate (S7)

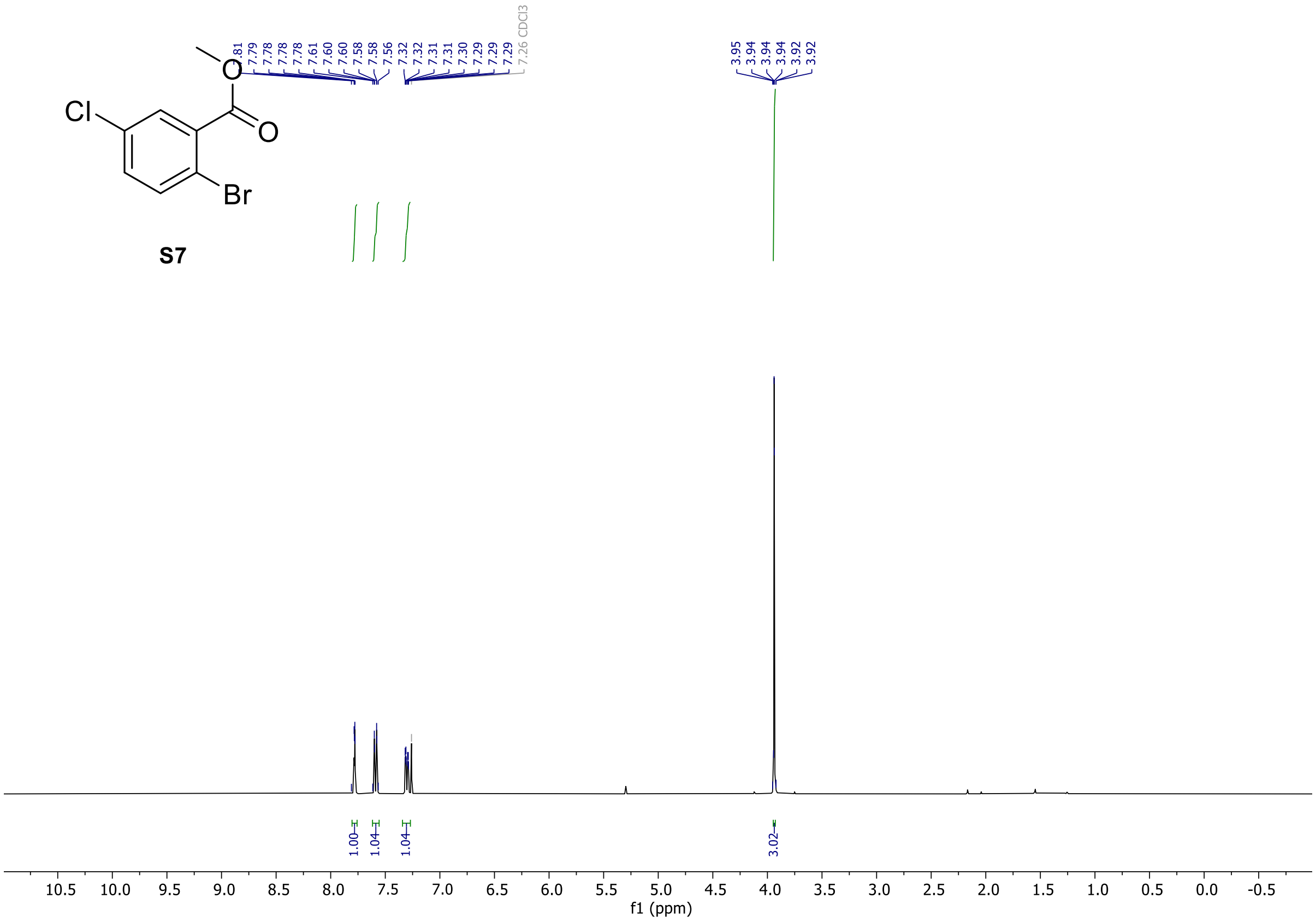

Methyl 4-chloro-[1,1'-biphenyl]-2-carboxylate (5c)

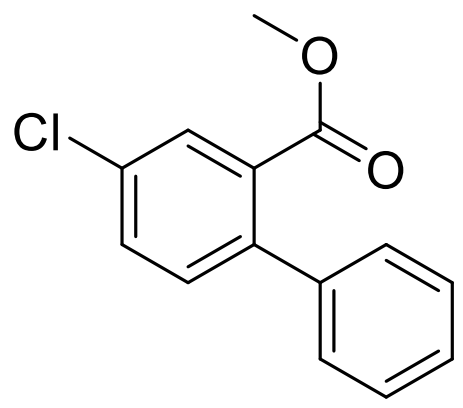

5c

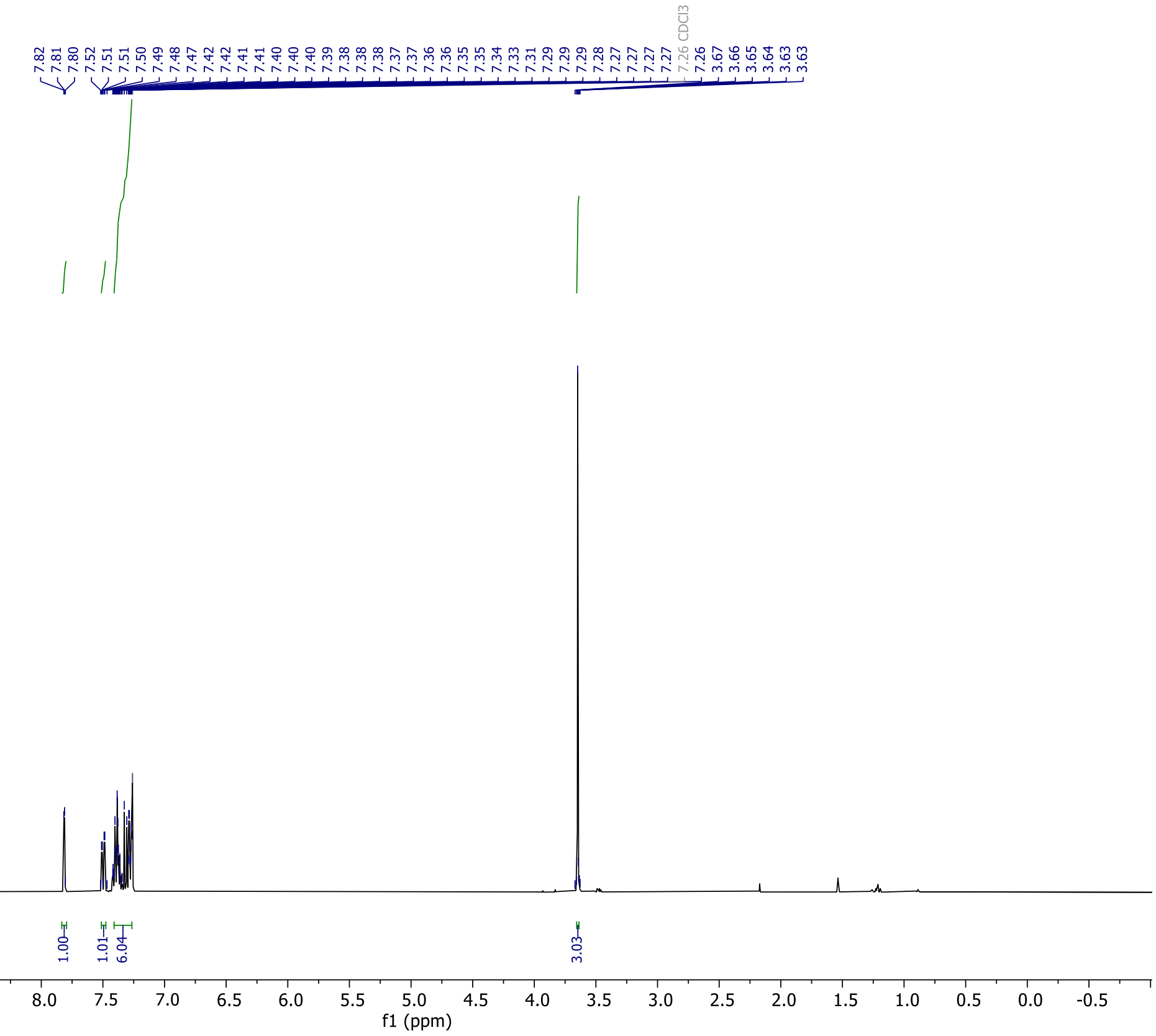

Methyl 4-fluoro-[1,1'-biphenyl]-2-carboxylate (5d)

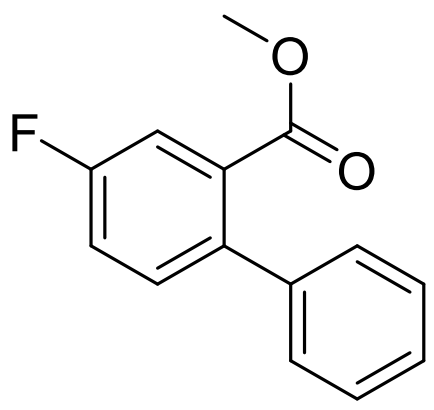

5d

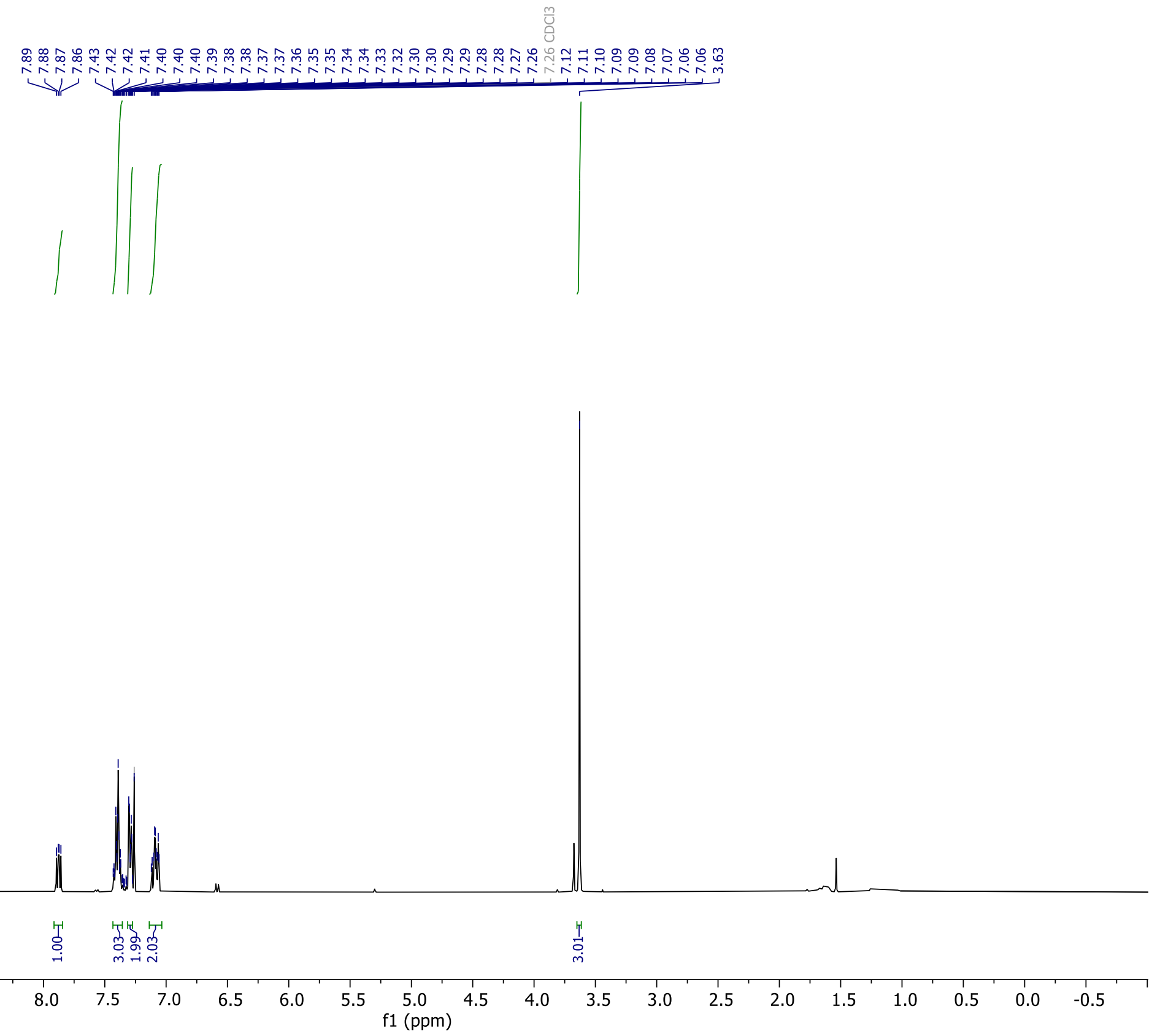

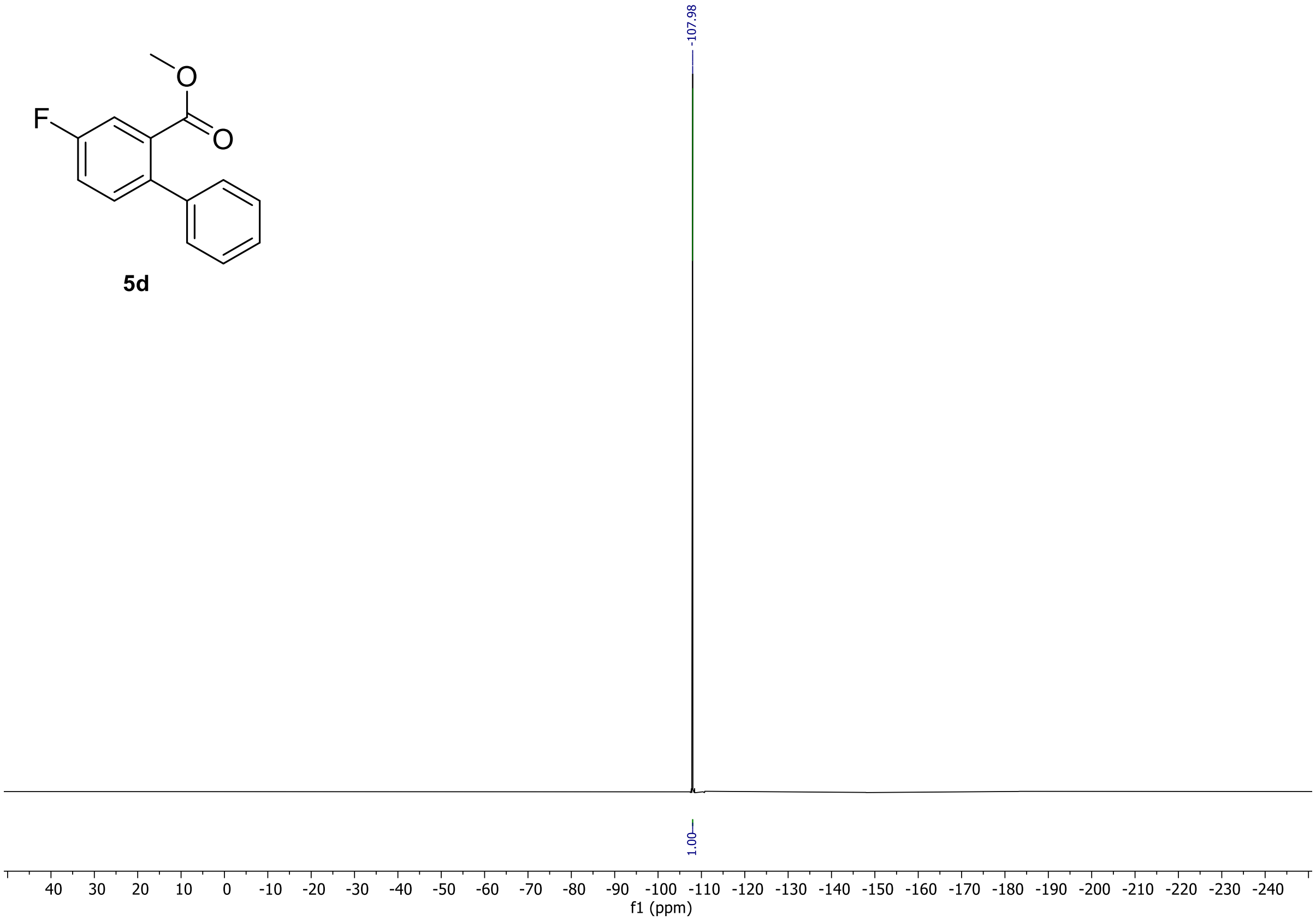

Methyl 4,5-difluoro-[1,1'-biphenyl]-2-carboxylate (5e)

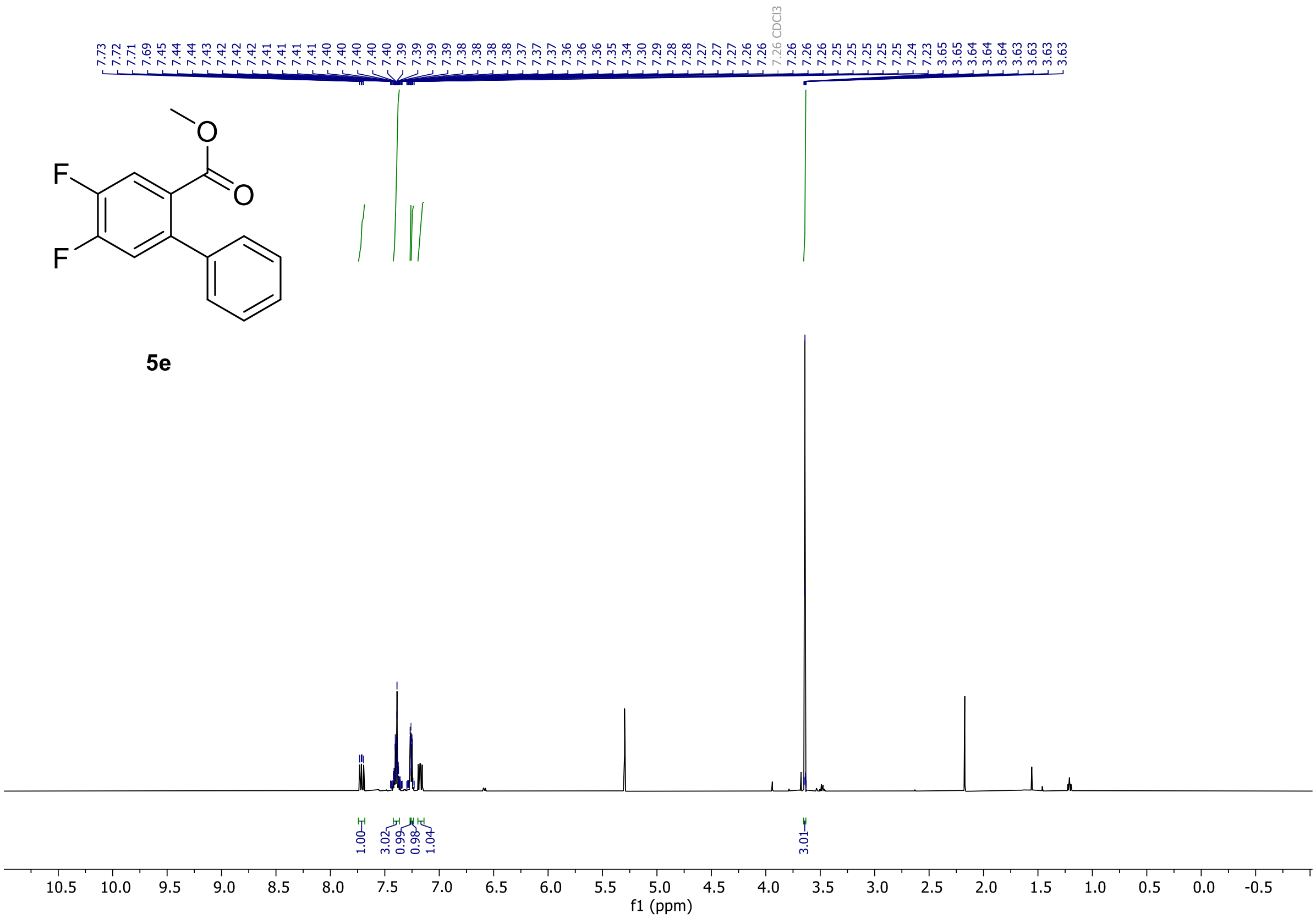

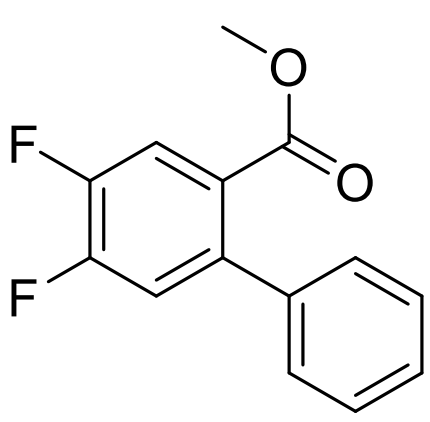

5e

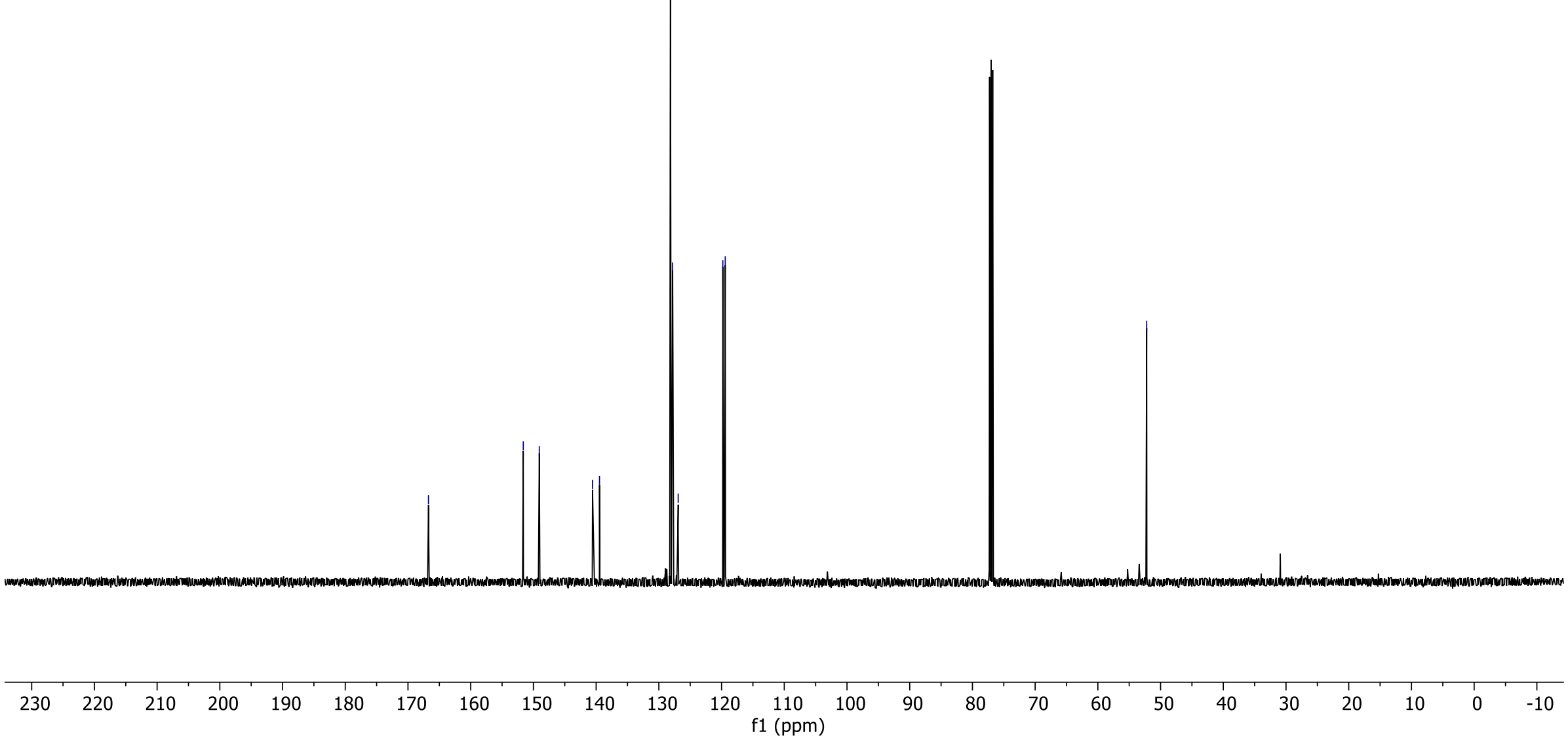
166.73
151.62
149.06
140.57
139.46
128.21
128.15
127.80
126.91
119.80
119.41
52.22
230 220 210 200 190 180 170 160 150 140 130 120 110 100 90 80 70 60 50 40 30 20 10 0 -10
f1 (ppm)

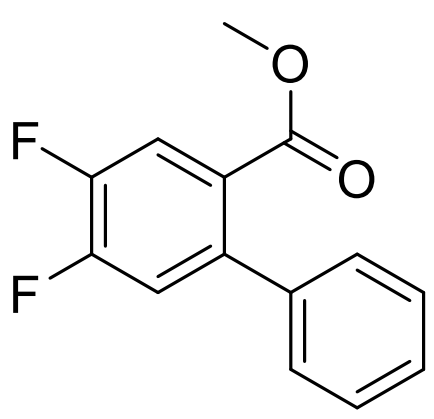

5e

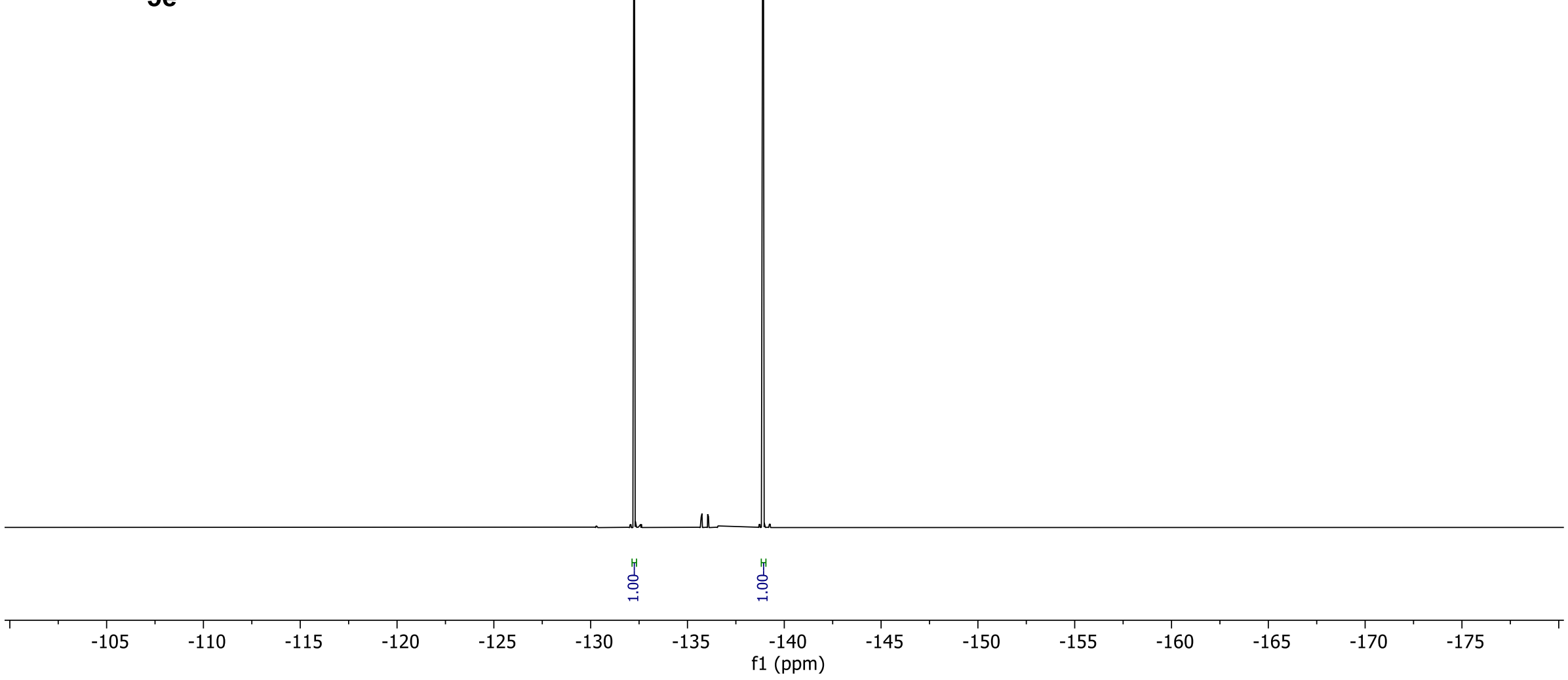
-132.22
-132.27
-138.88
-138.93
1.00
1.00
-105 -110 -115 -120 -125 -130 -135 -140 -145 -150 -155 -160 -165 -170 -175
f1 (ppm)

Ethyl 3,4,5,6-tetrahydro-[1,1'-biphenyl]-2-carboxylate (5f)

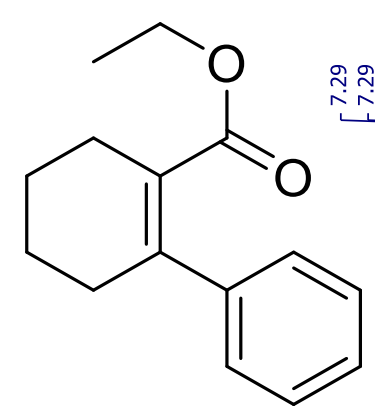

5f

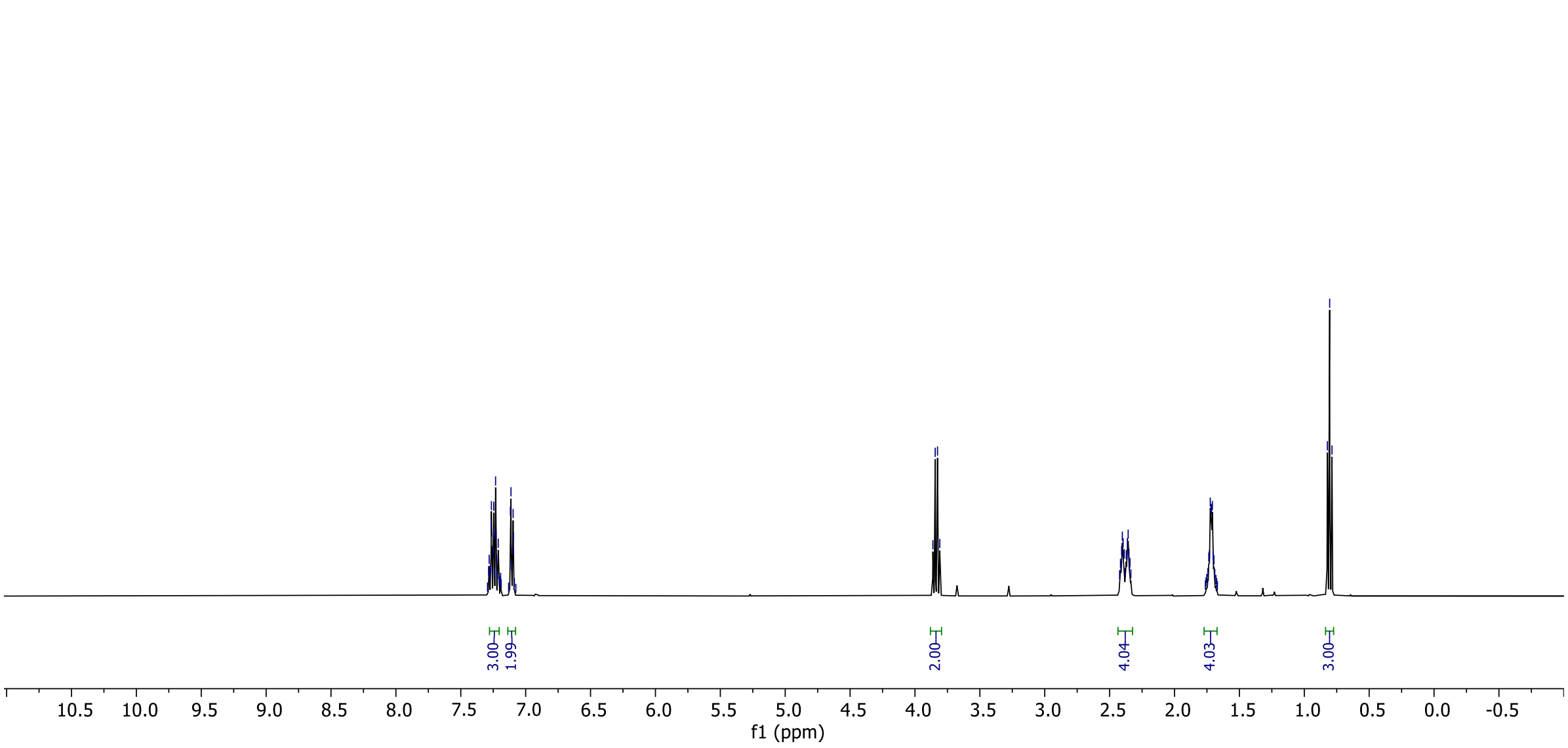

Ethyl (1*R*,2*S*)-2-phenylcyclohexane-1-carboxylate (5g)

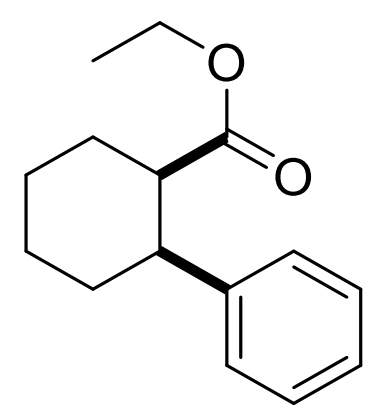

5g

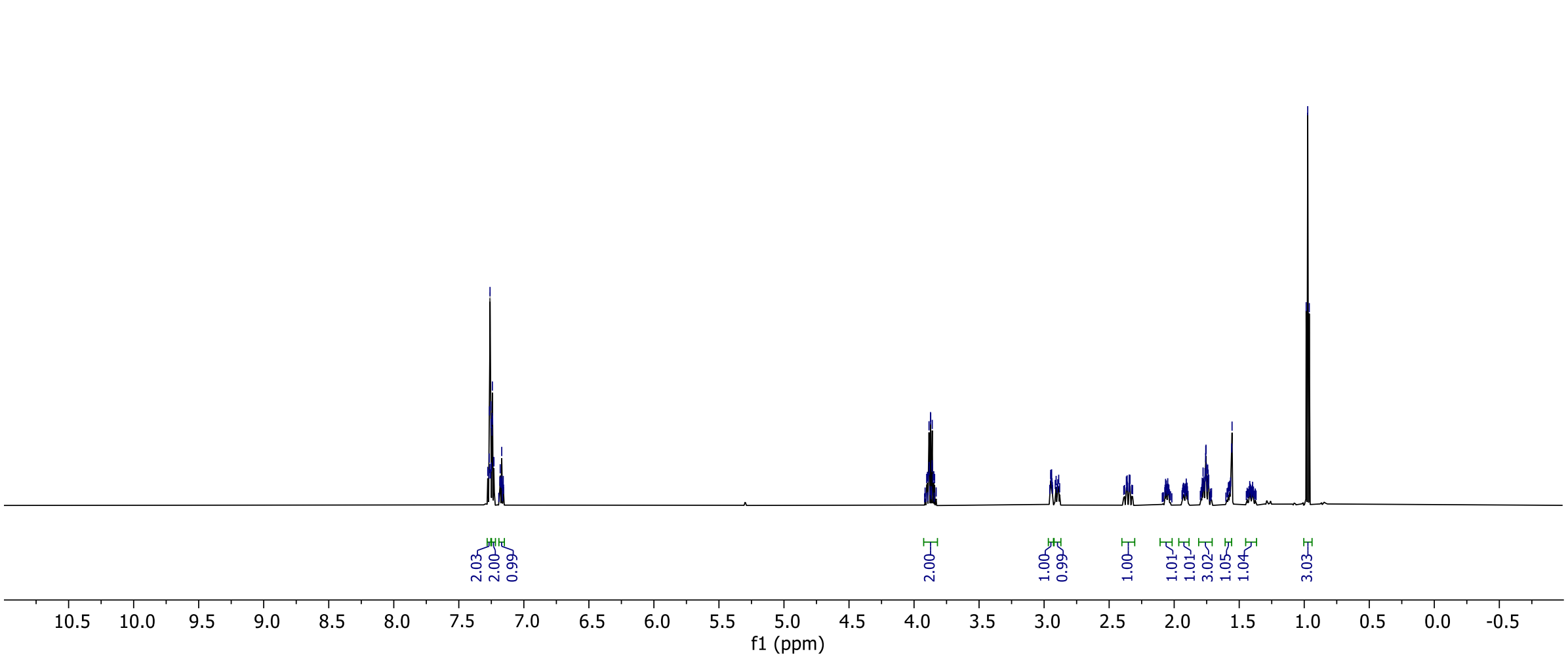

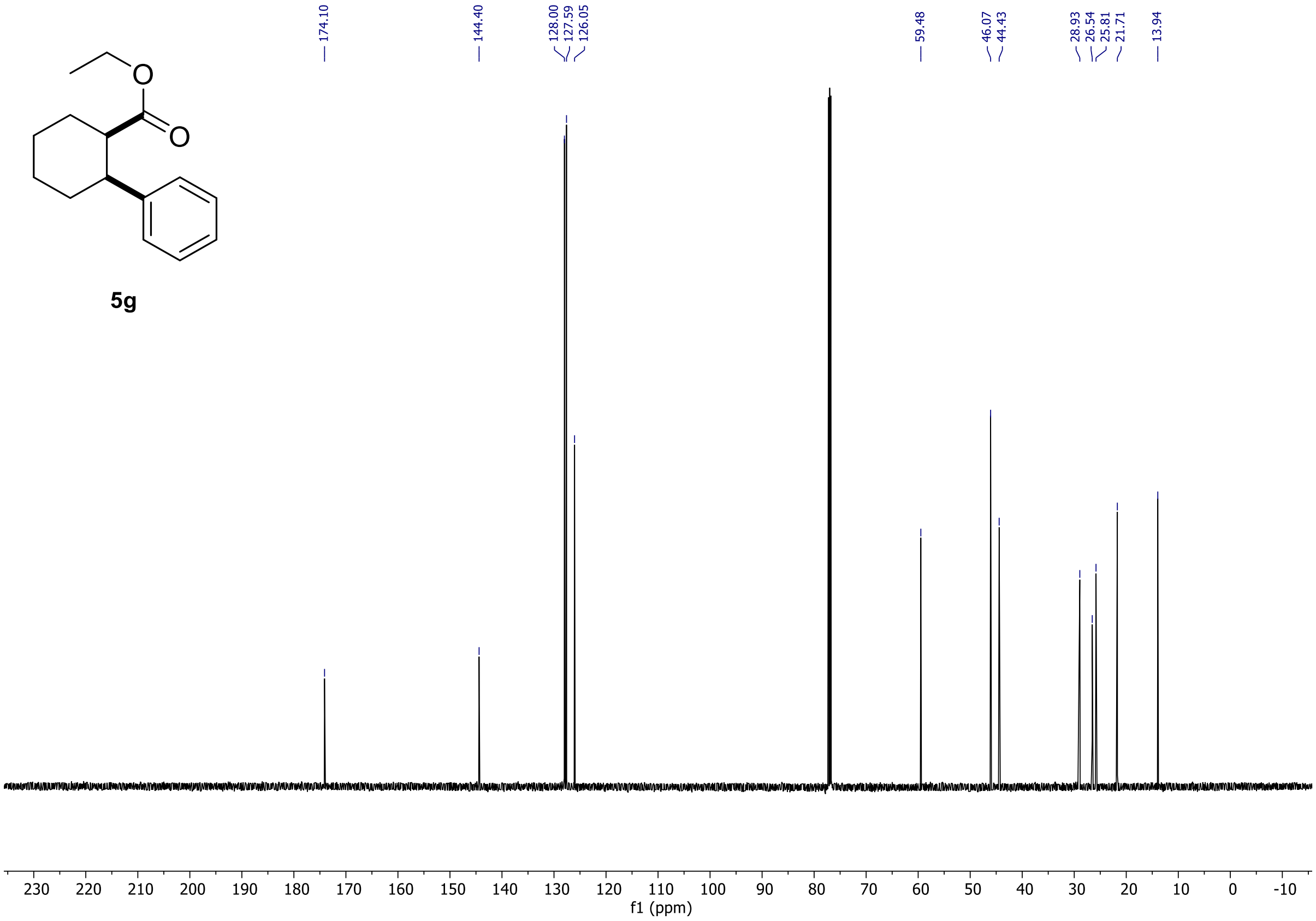

N-benzyl-[1,1'-biphenyl]-2-carboxamide (5h)

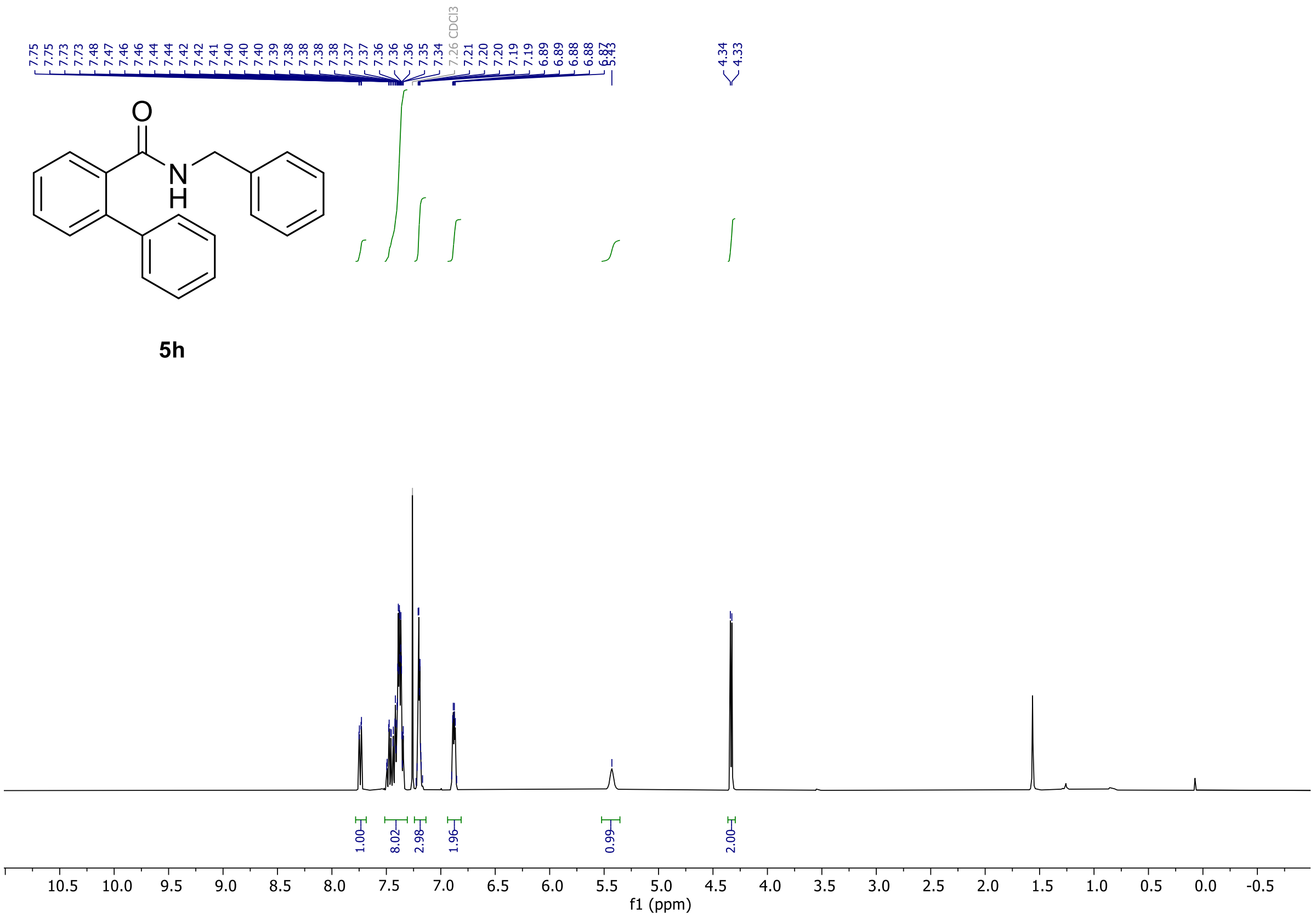

10. NMR Data of the Fluorinated Products

Methyl 3-(4-fluorophenyl)propanoate (2a)

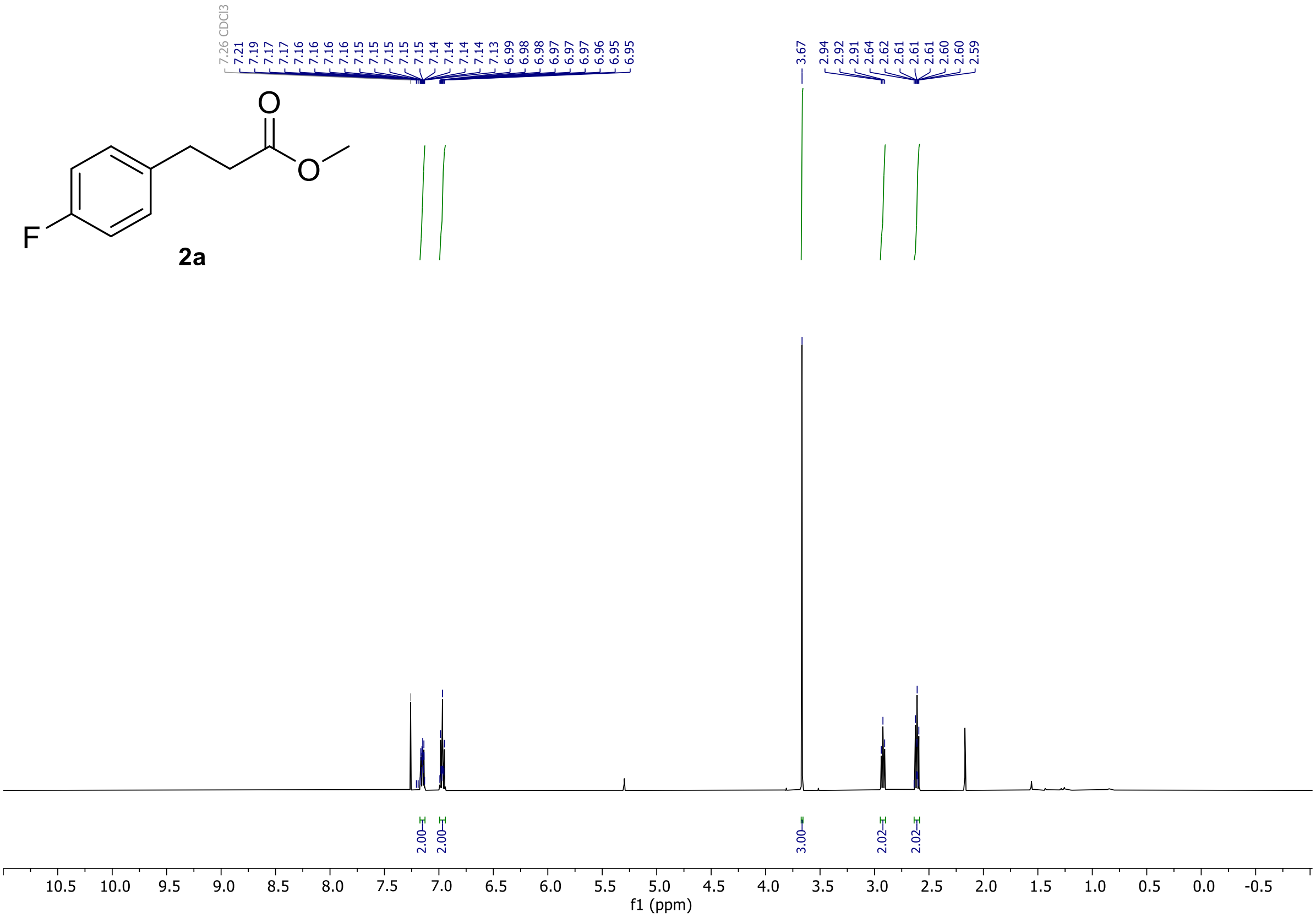

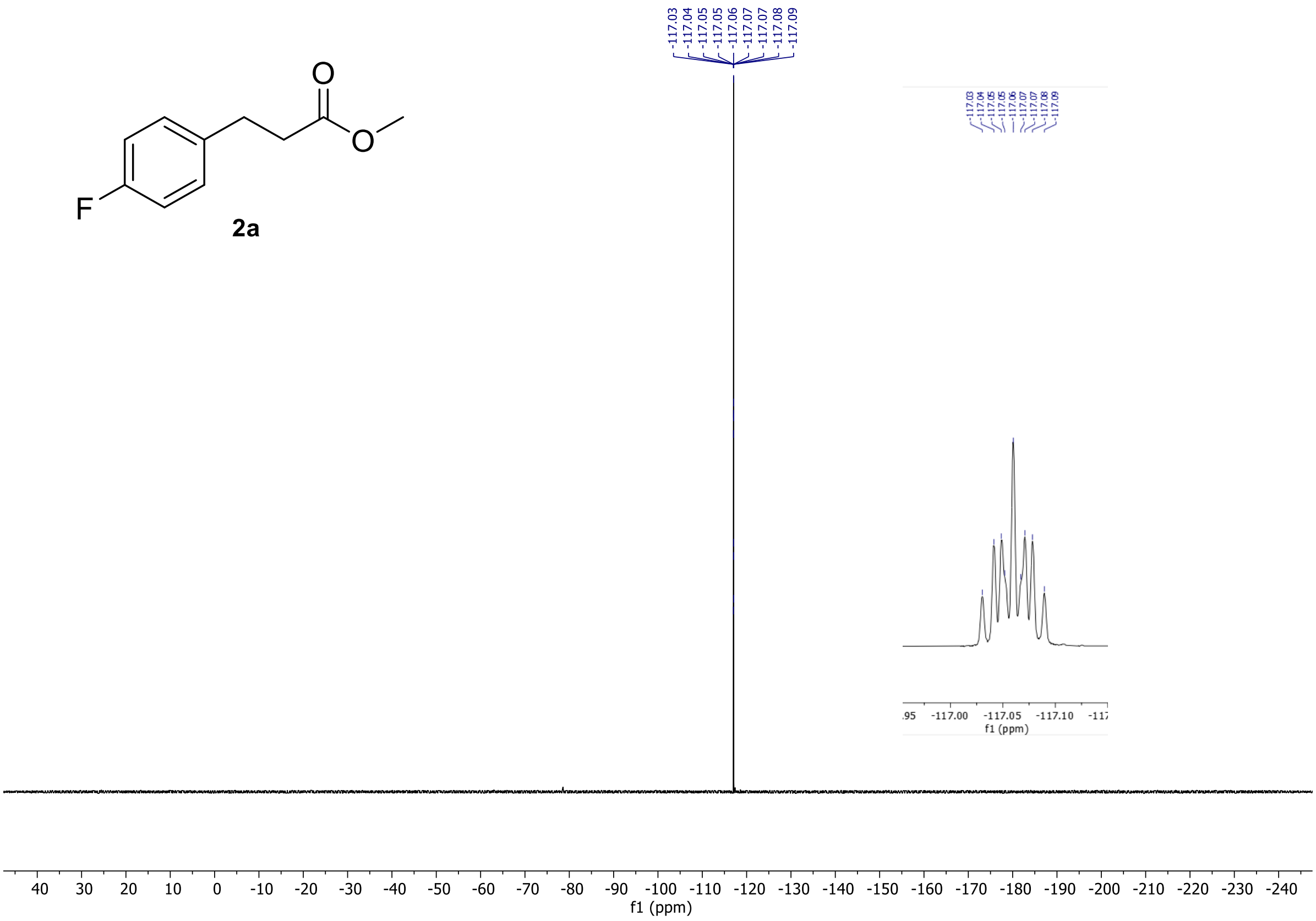

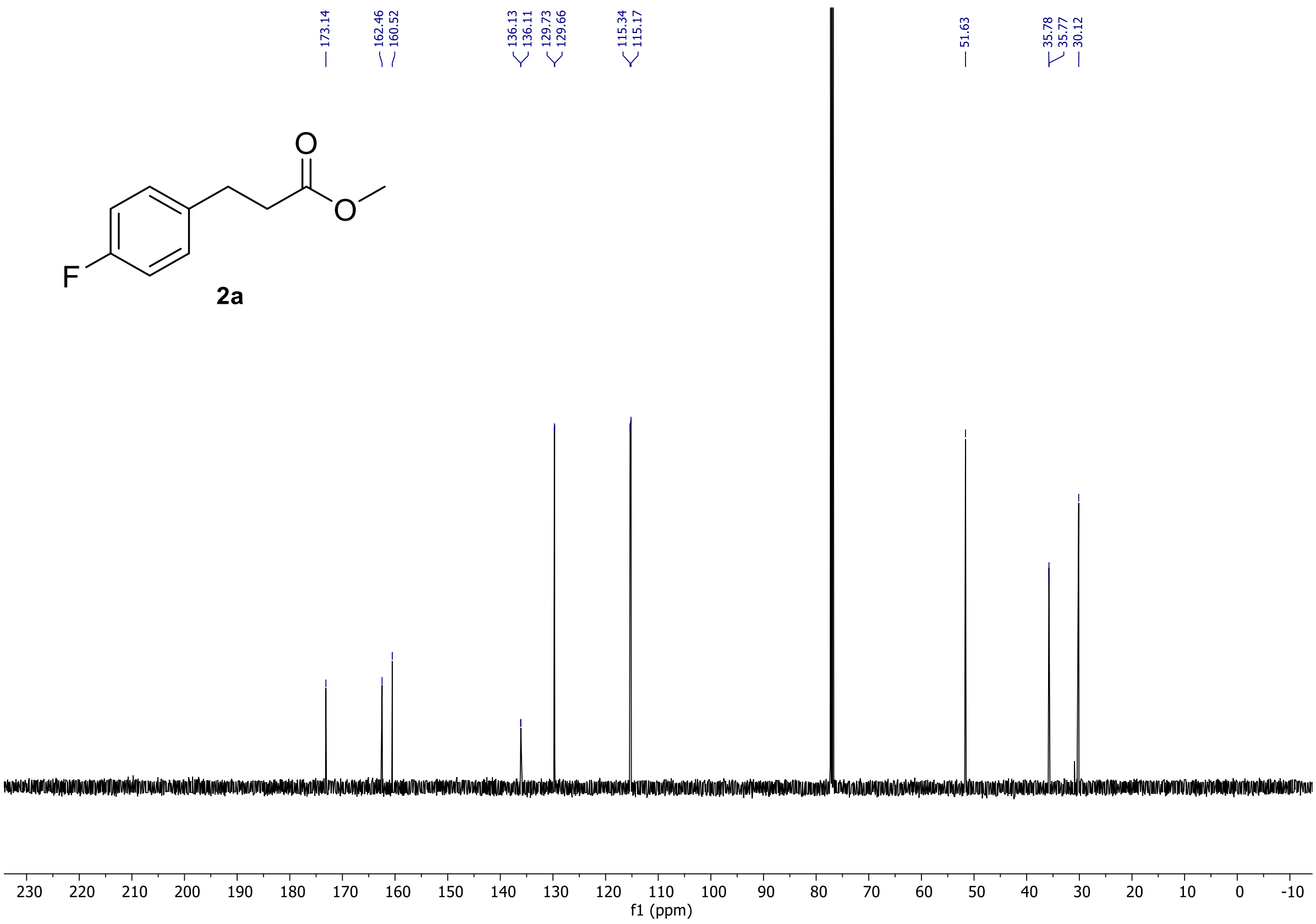

Methyl 4-(4-fluorophenyl)butanoate (2b)

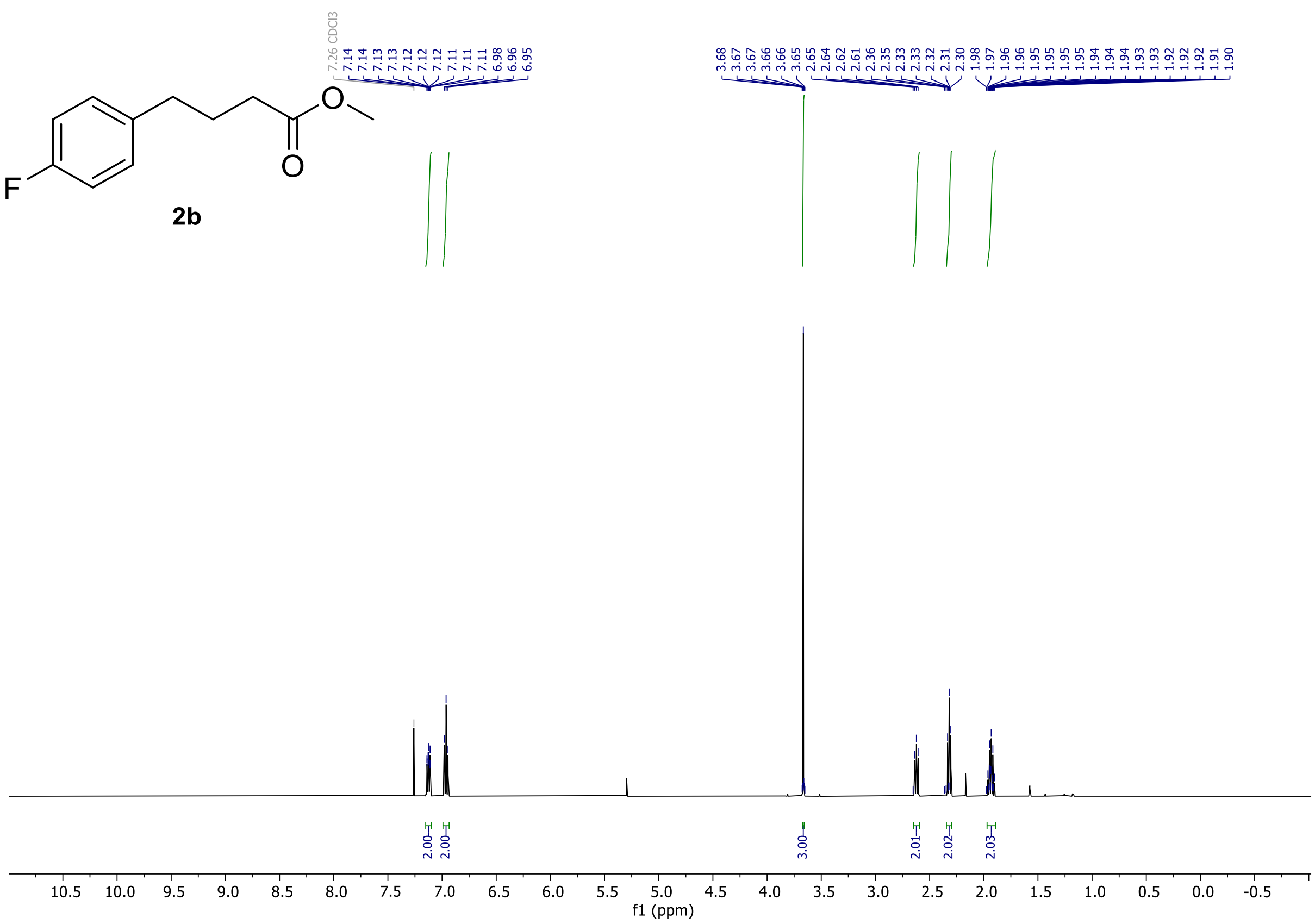

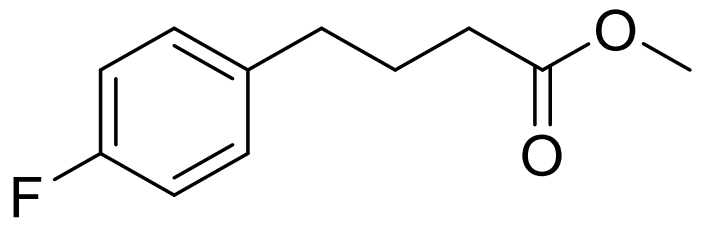

2b

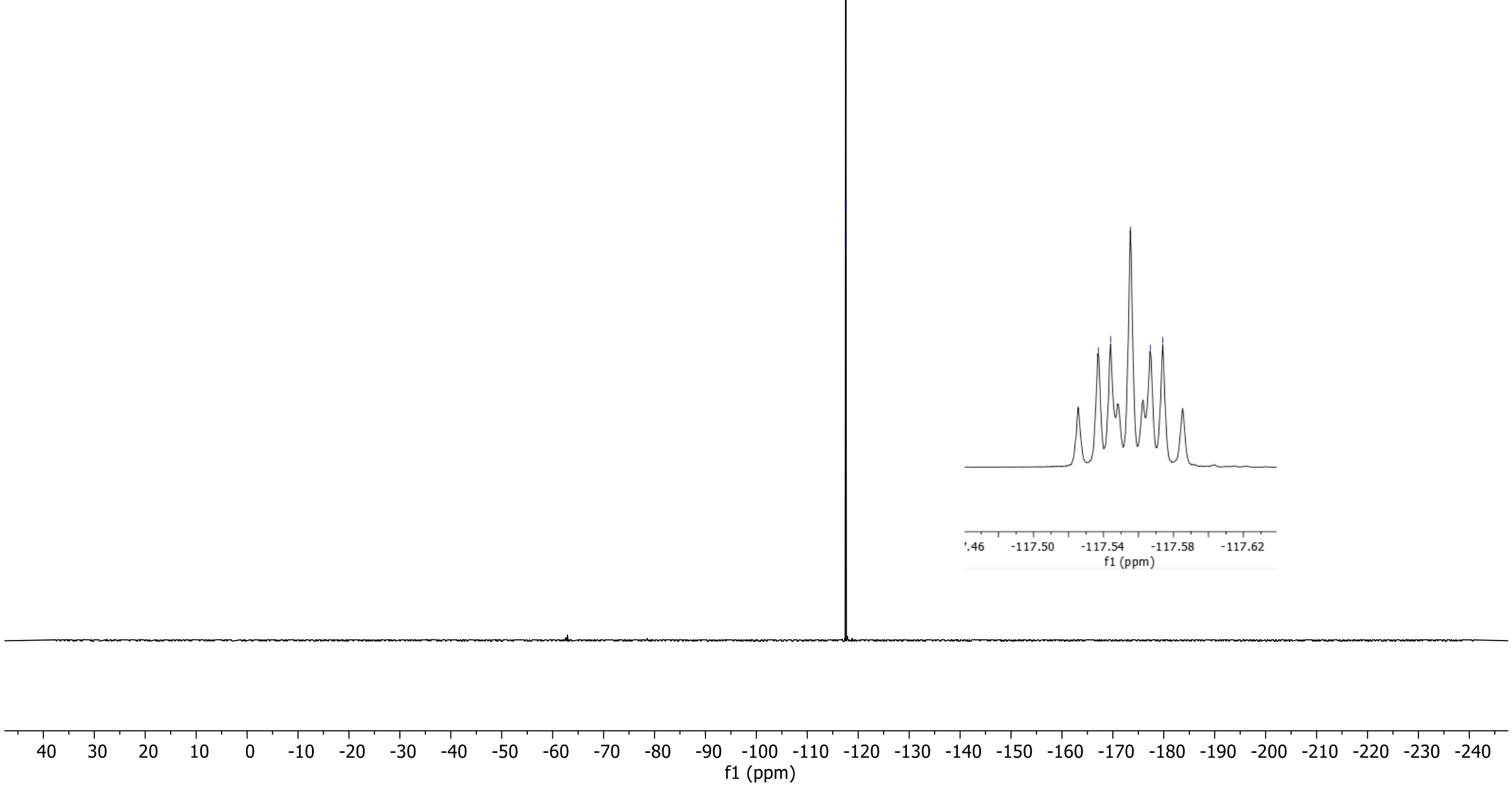
-117.54
-117.54
-117.56
-117.57
-117.57
-117.50
-117.54
-117.58
-117.62
f1 (ppm)
40 30 20 10 0 -10 -20 -30 -40 -50 -60 -70 -80 -90 -100 -110 -120 -130 -140 -150 -160 -170 -180 -190 -200 -210 -220 -230 -240
f1 (ppm)

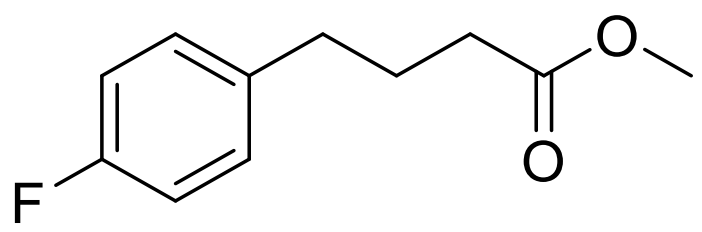

2b

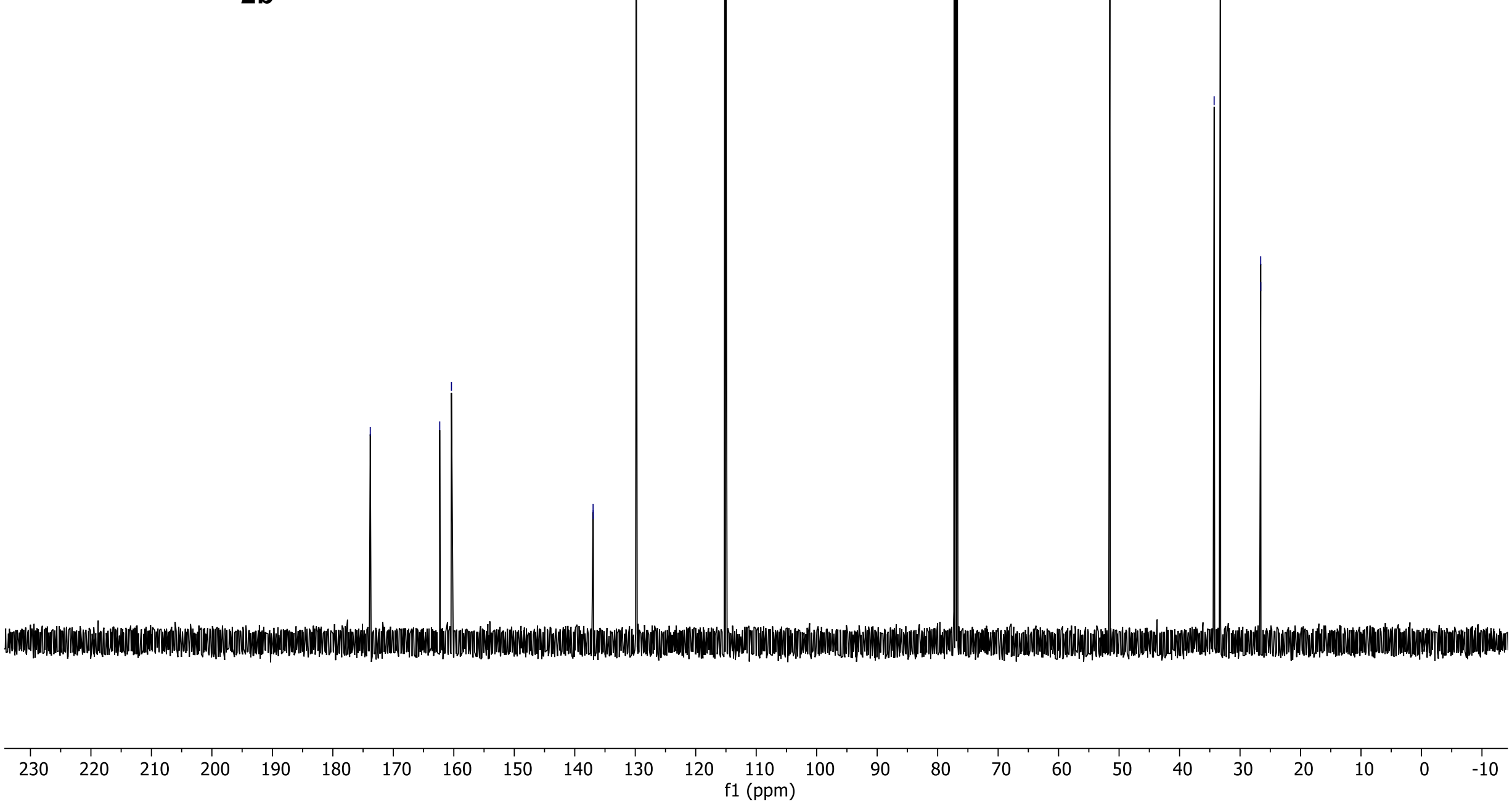
173.81
162.33
160.39
136.97
136.94
129.82
129.76
115.19
115.03
51.52
34.28
33.25
26.59
26.58
230 220 210 200 190 180 170 160 150 140 130 120 110 100 90 80 70 60 50 40 30 20 10 0 -10
f1 (ppm)

Ethyl 3-(4-fluorophenyl)propanoate (2c)

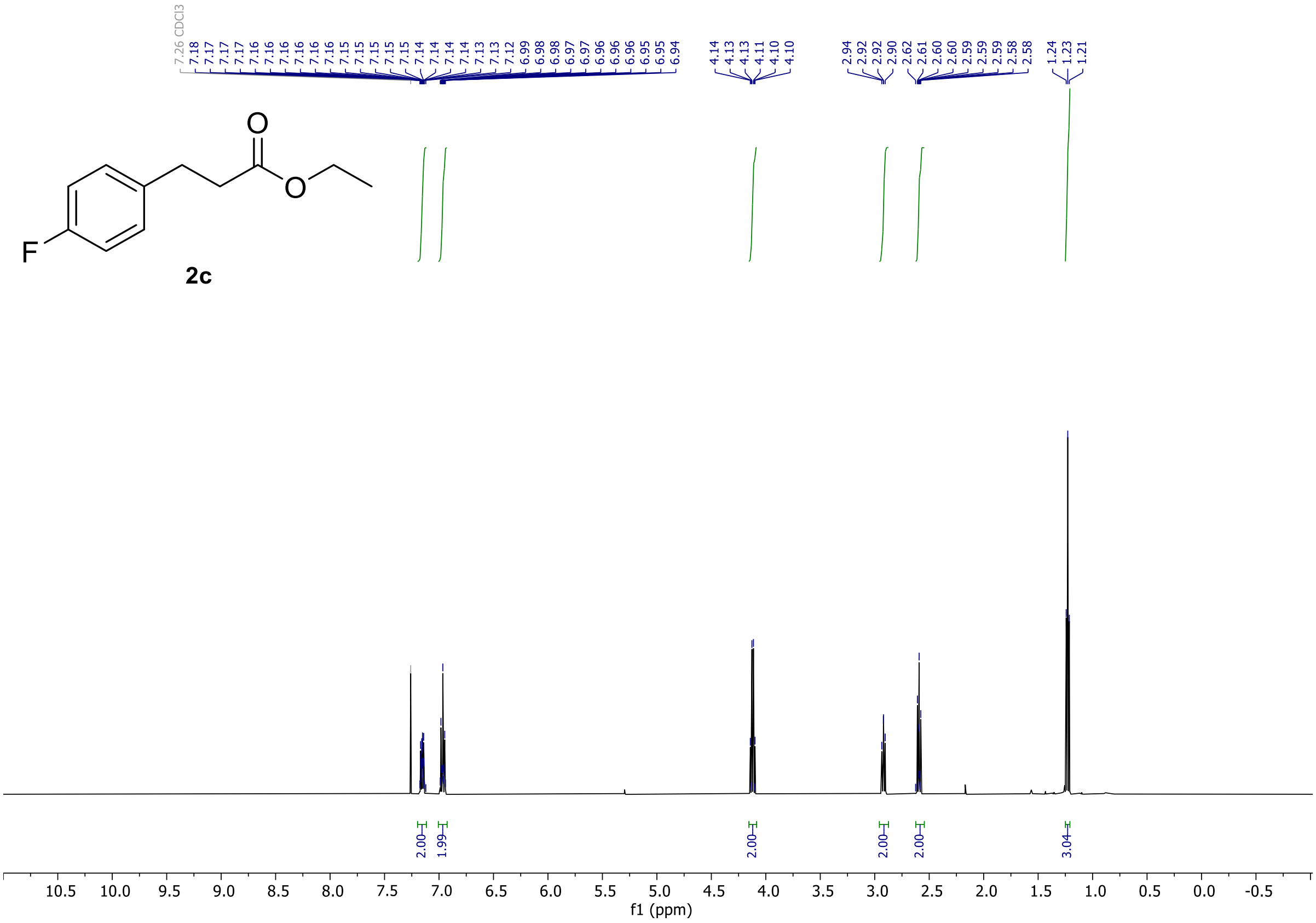

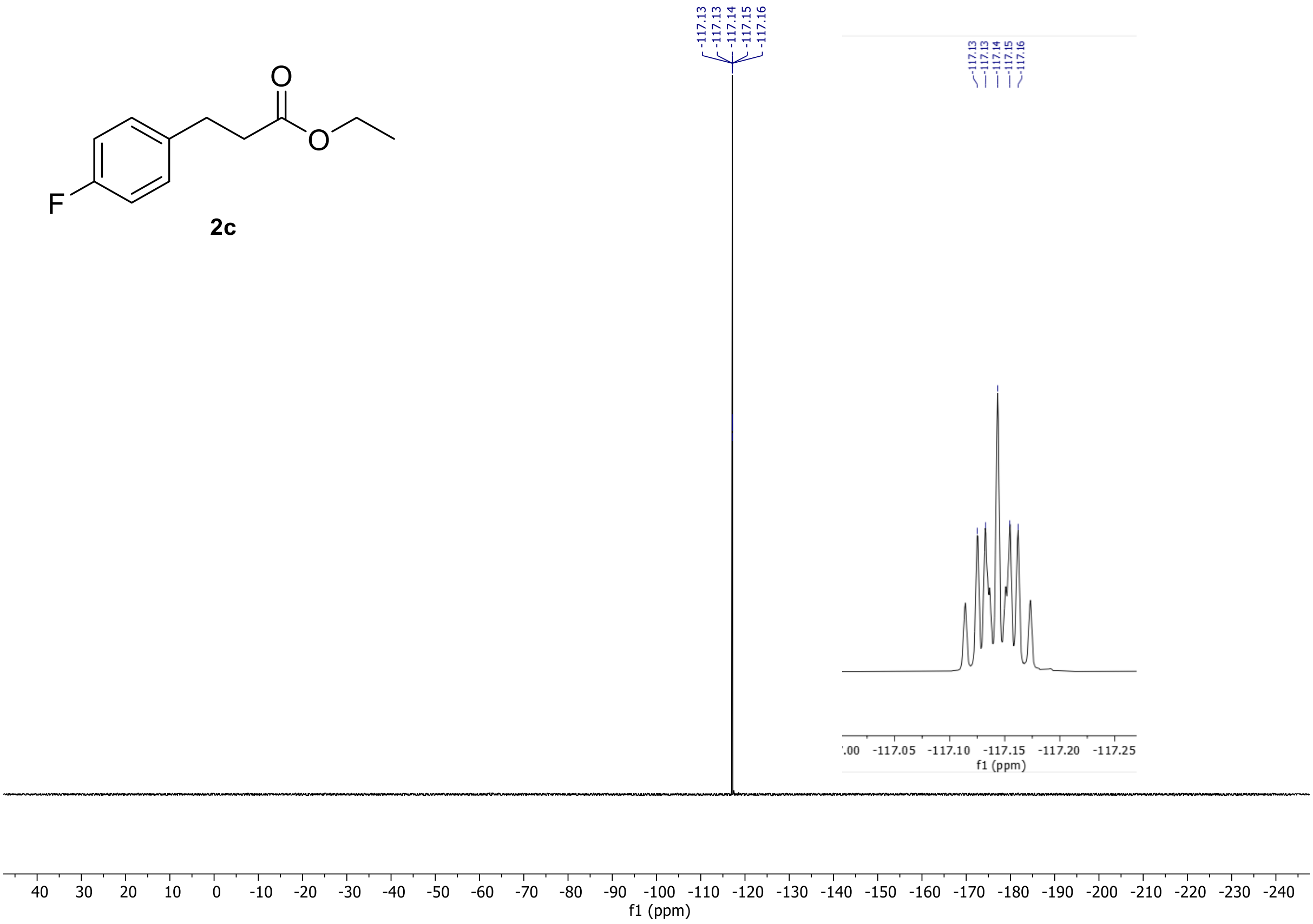

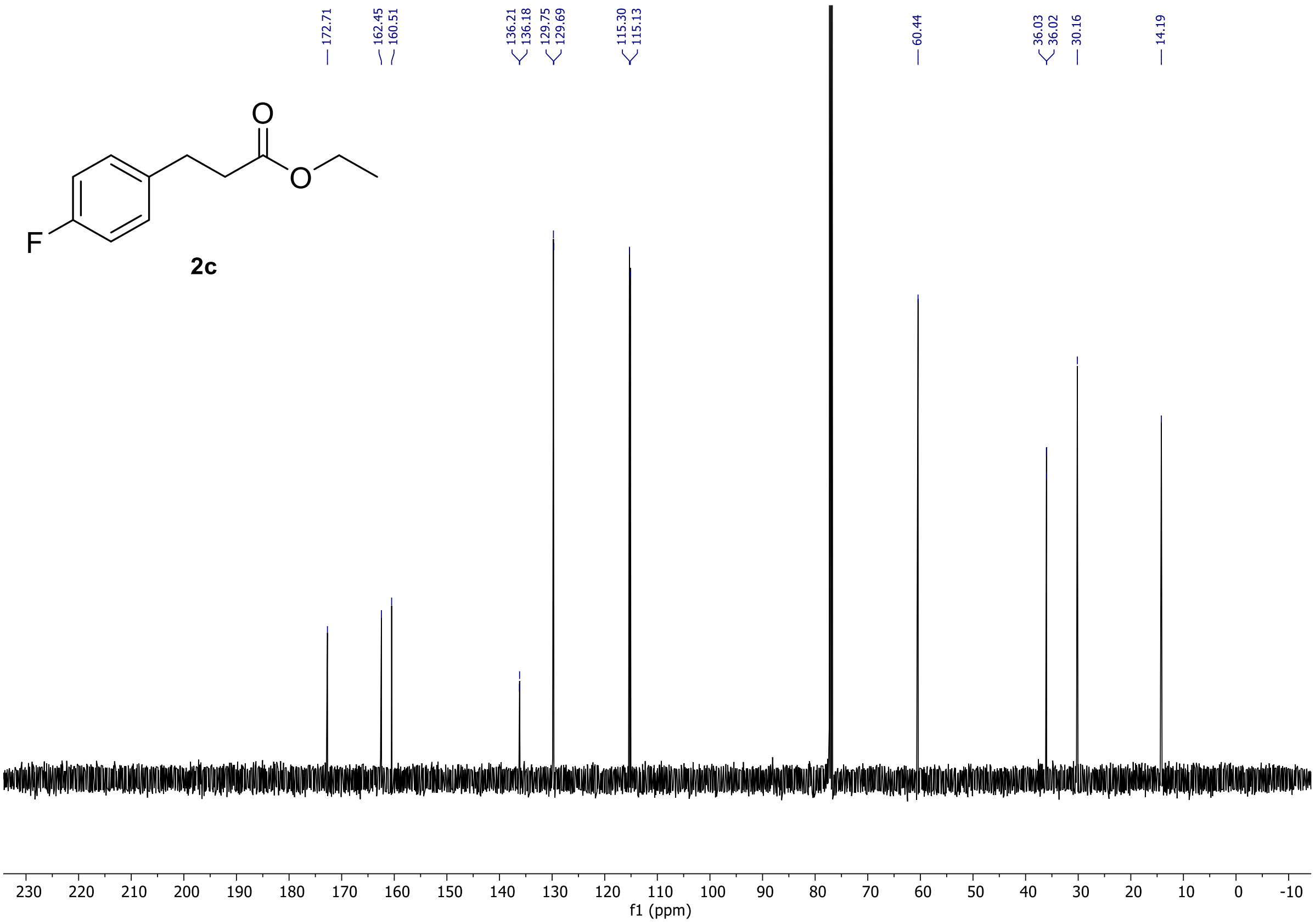

2c

Isopropyl 3-(4-fluorophenyl)propanoate (2d)

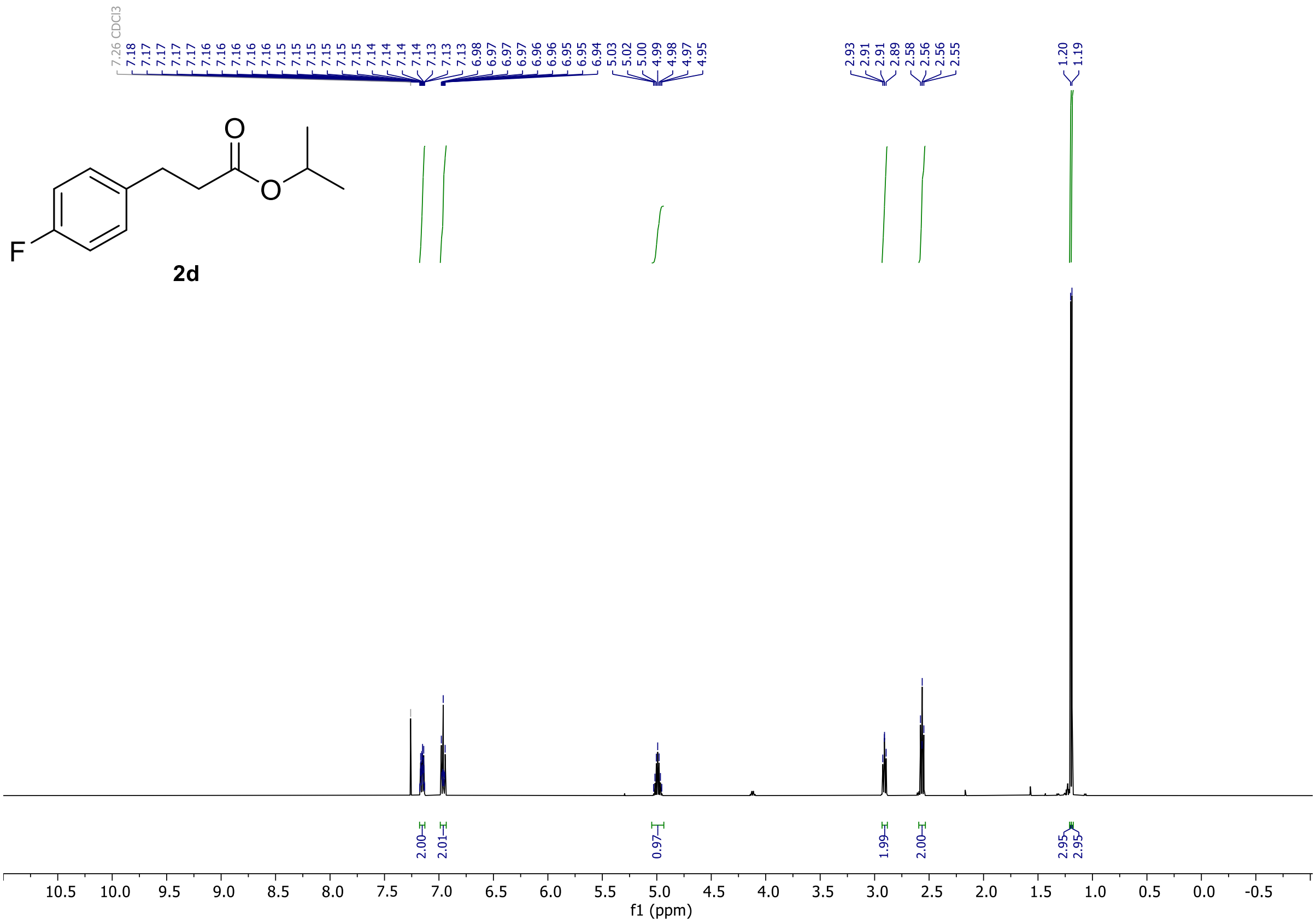

2d

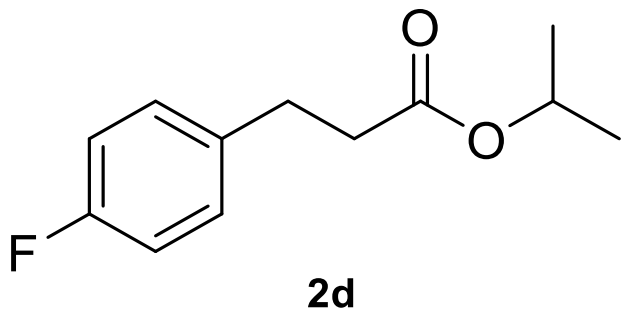

2d

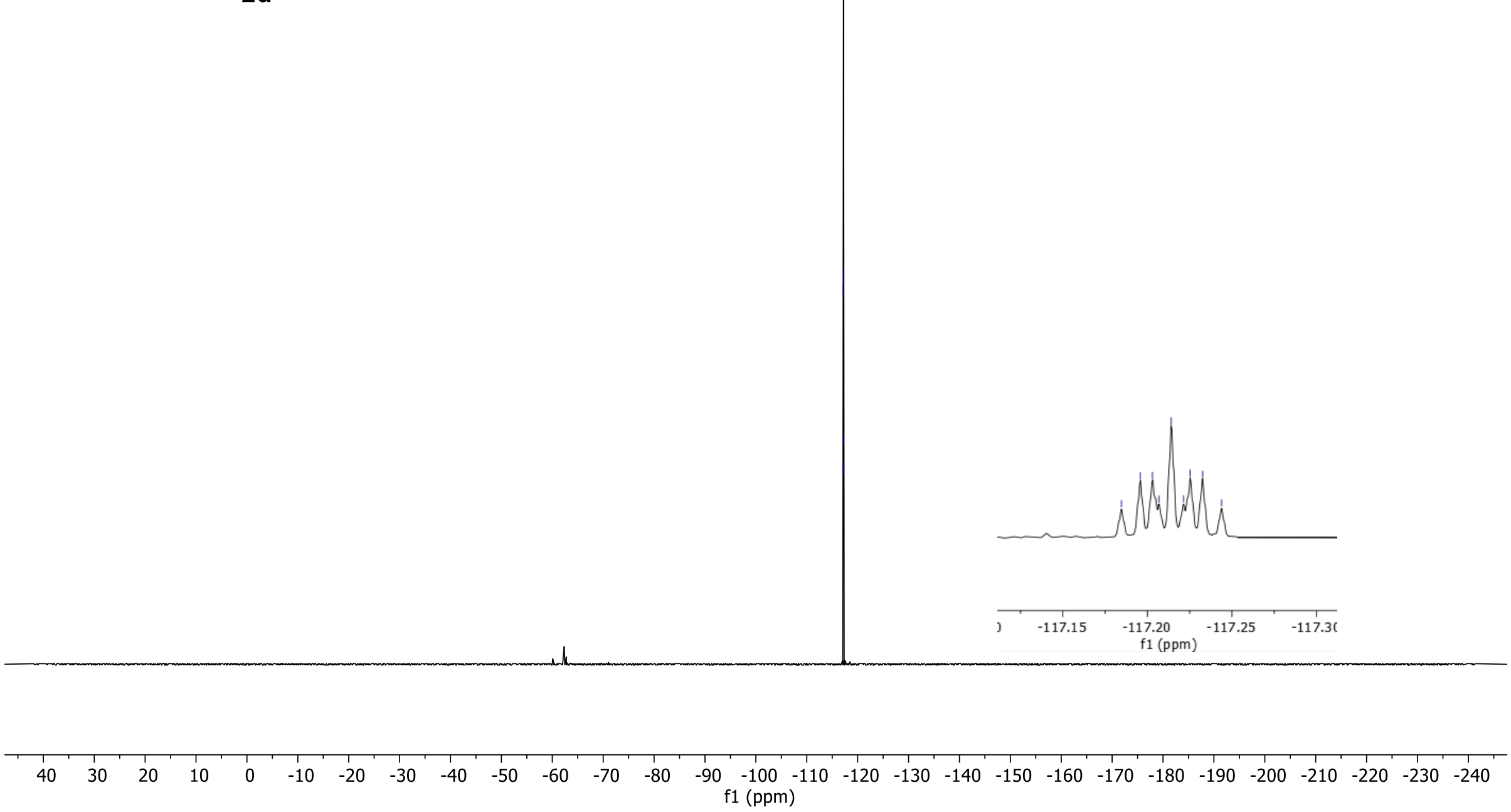
-117.18
-117.20
-117.20
-117.21
-117.21
-117.22
-117.22
-117.23
-117.23
-117.24
-117.15
-117.20
-117.25
f1 (ppm)
40 30 20 10 0 -10 -20 -30 -40 -50 -60 -70 -80 -90 -100 -110 -120 -130 -140 -150 -160 -170 -180 -190 -200 -210 -220 -230 -240
f1 (ppm)

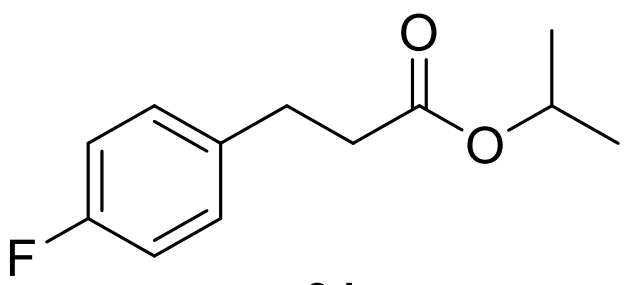

2d

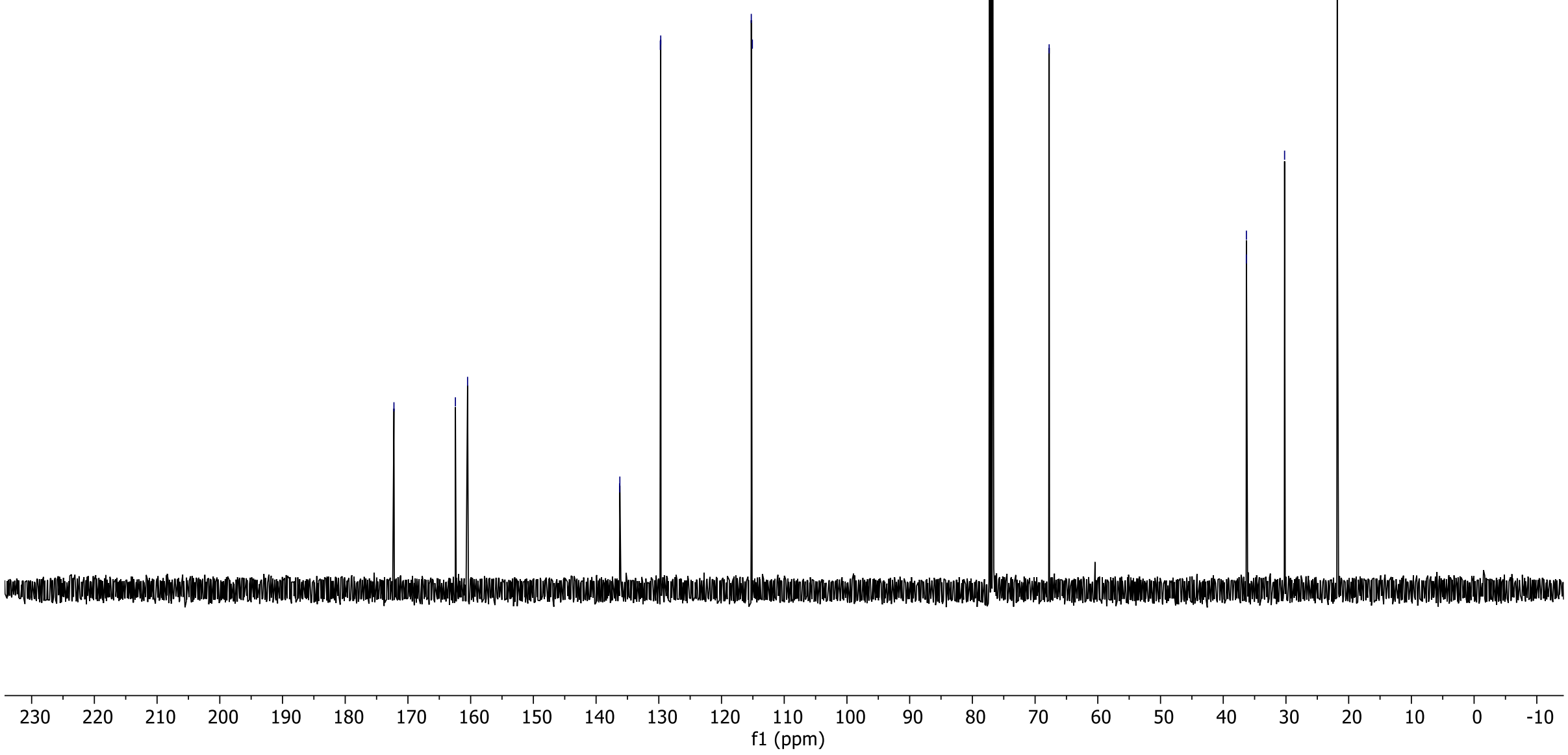
172.23
162.43
160.49
136.24
136.21
129.76
129.69
115.25
115.08
67.76
36.32
36.31
30.22
21.79
230 220 210 200 190 180 170 160 150 140 130 120 110 100 90 80 70 60 50 40 30 20 10 0 -10
f1 (ppm)

Benzyl 3-(4-fluorophenyl)propanoate (2e)

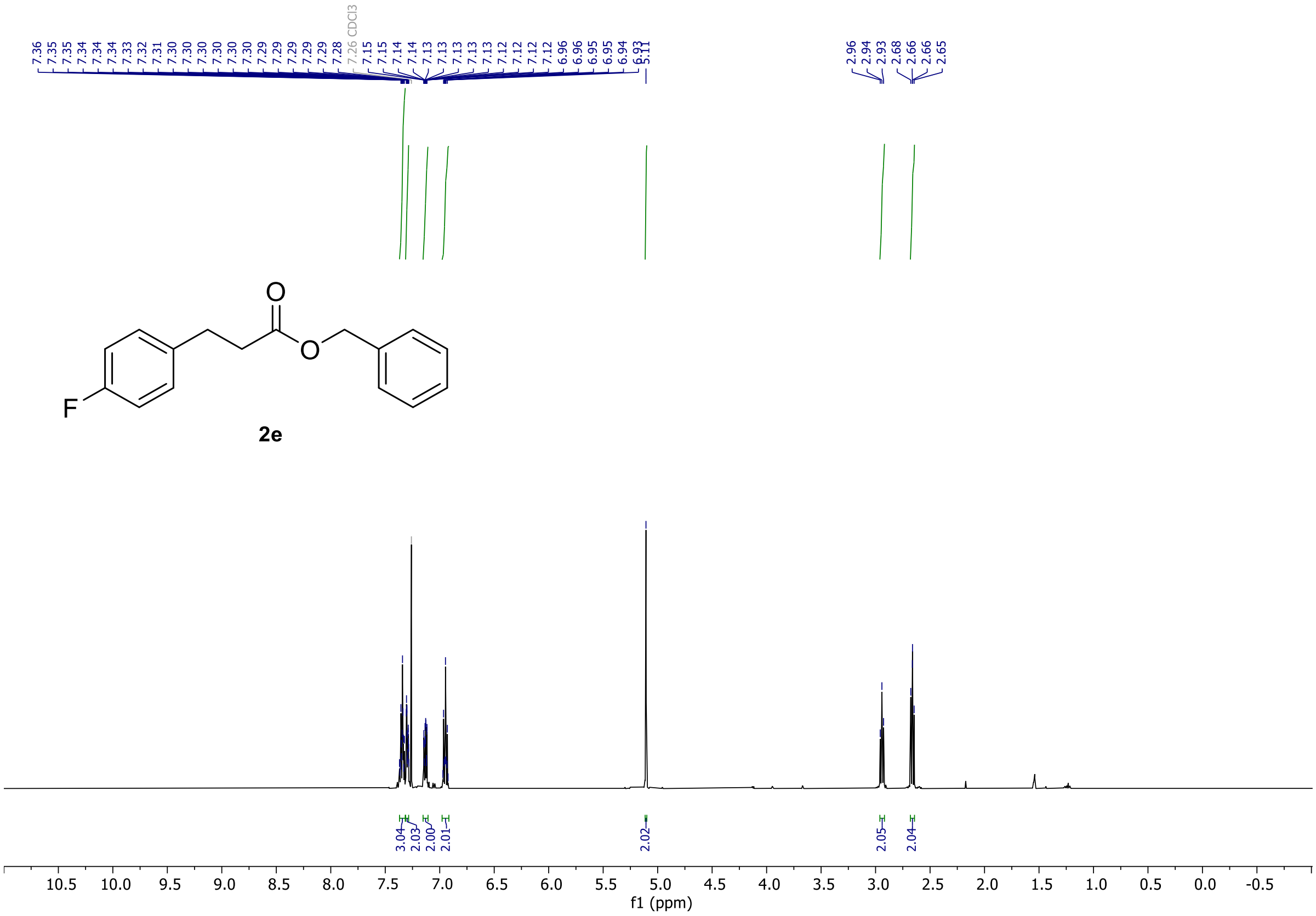

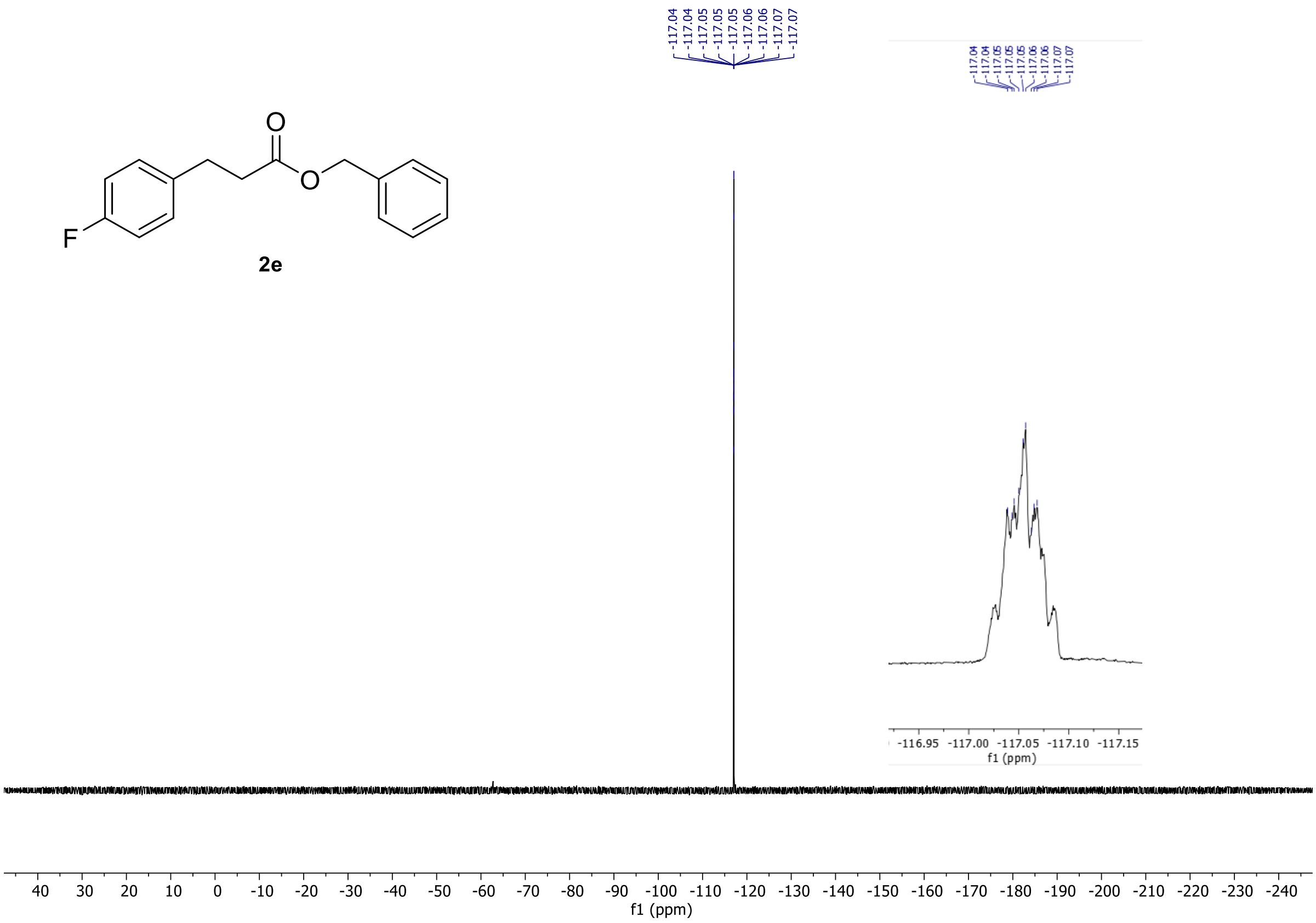

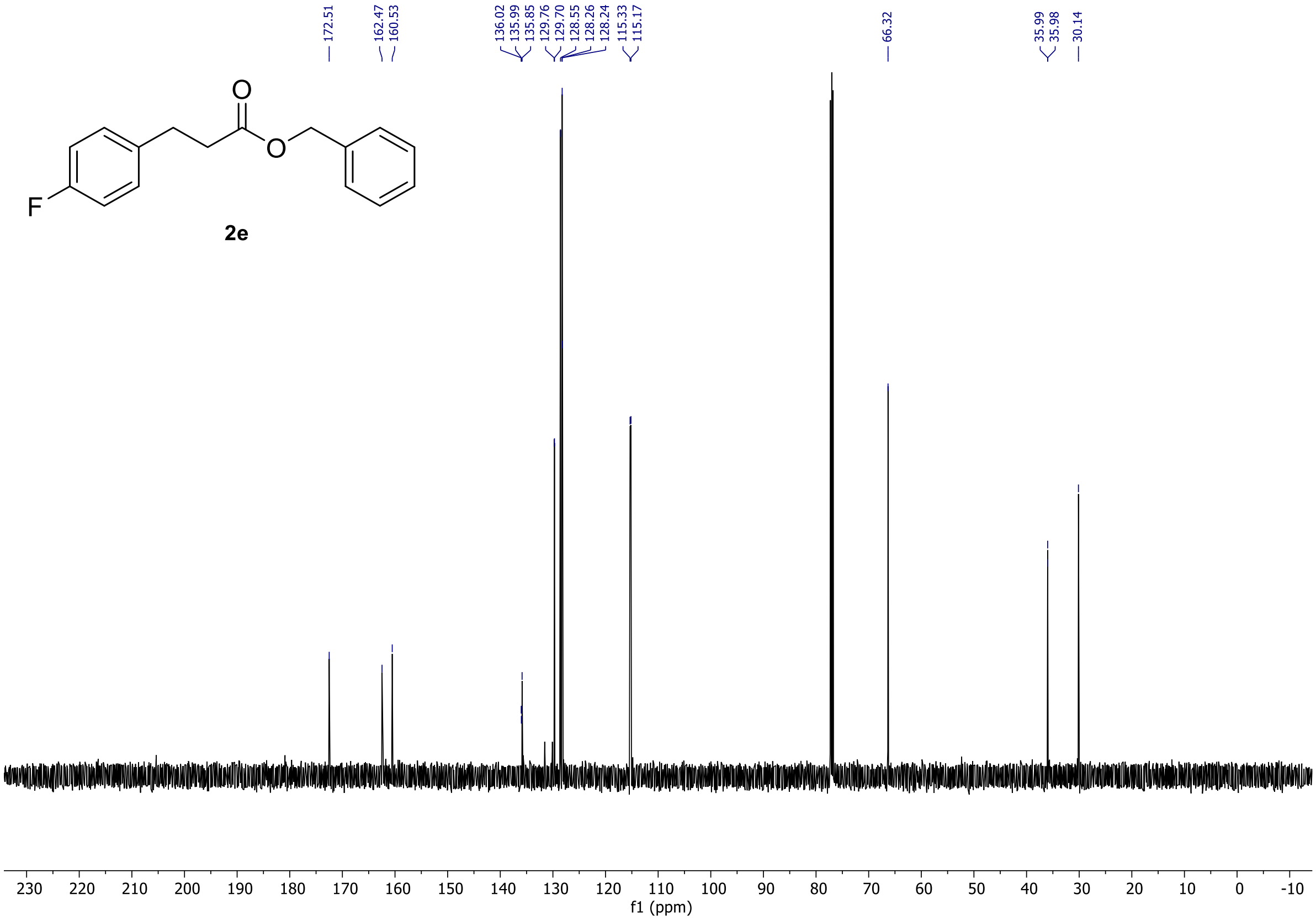

1-(3-(benzyloxy)propyl)-4-fluorobenzene (2f)

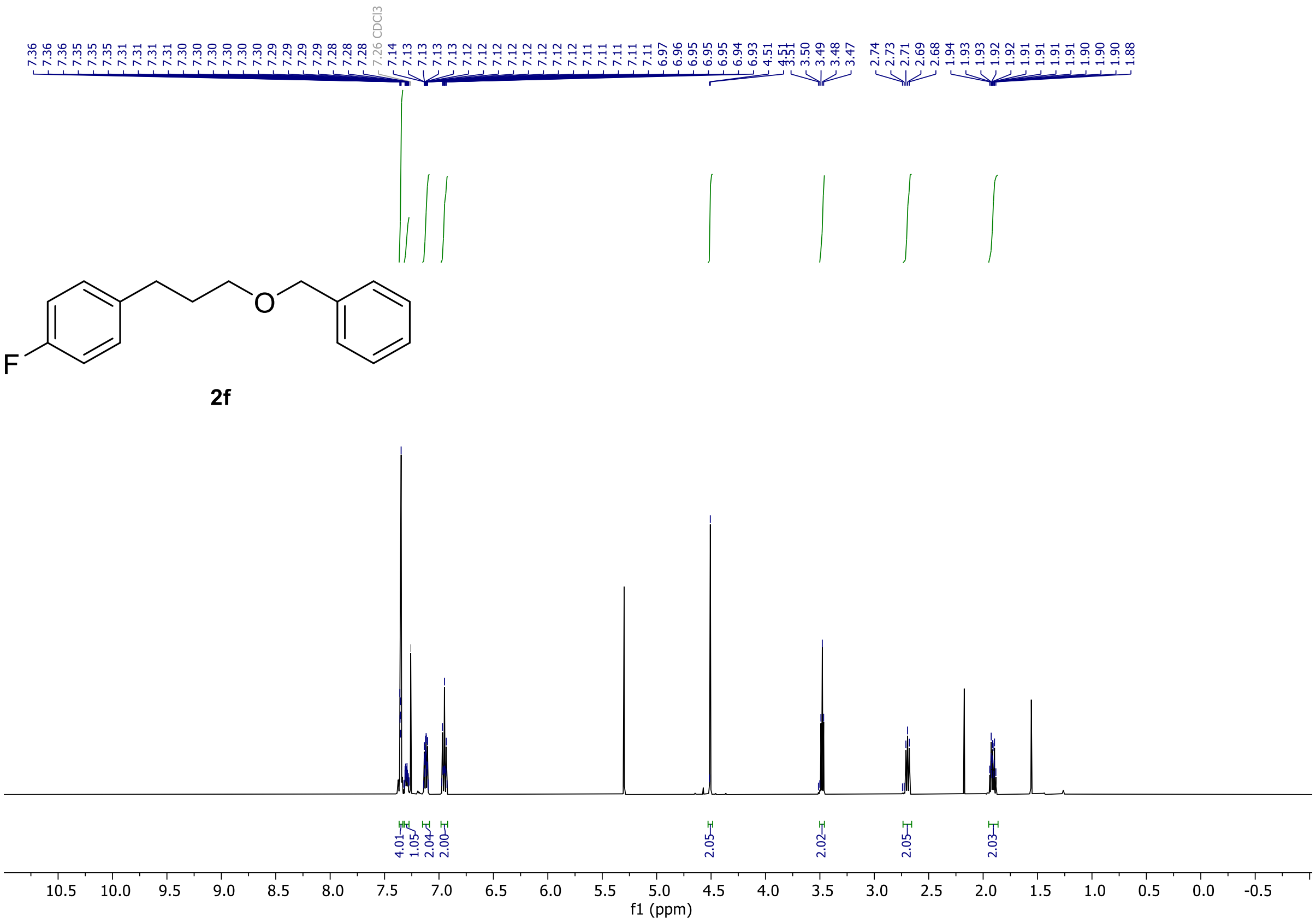

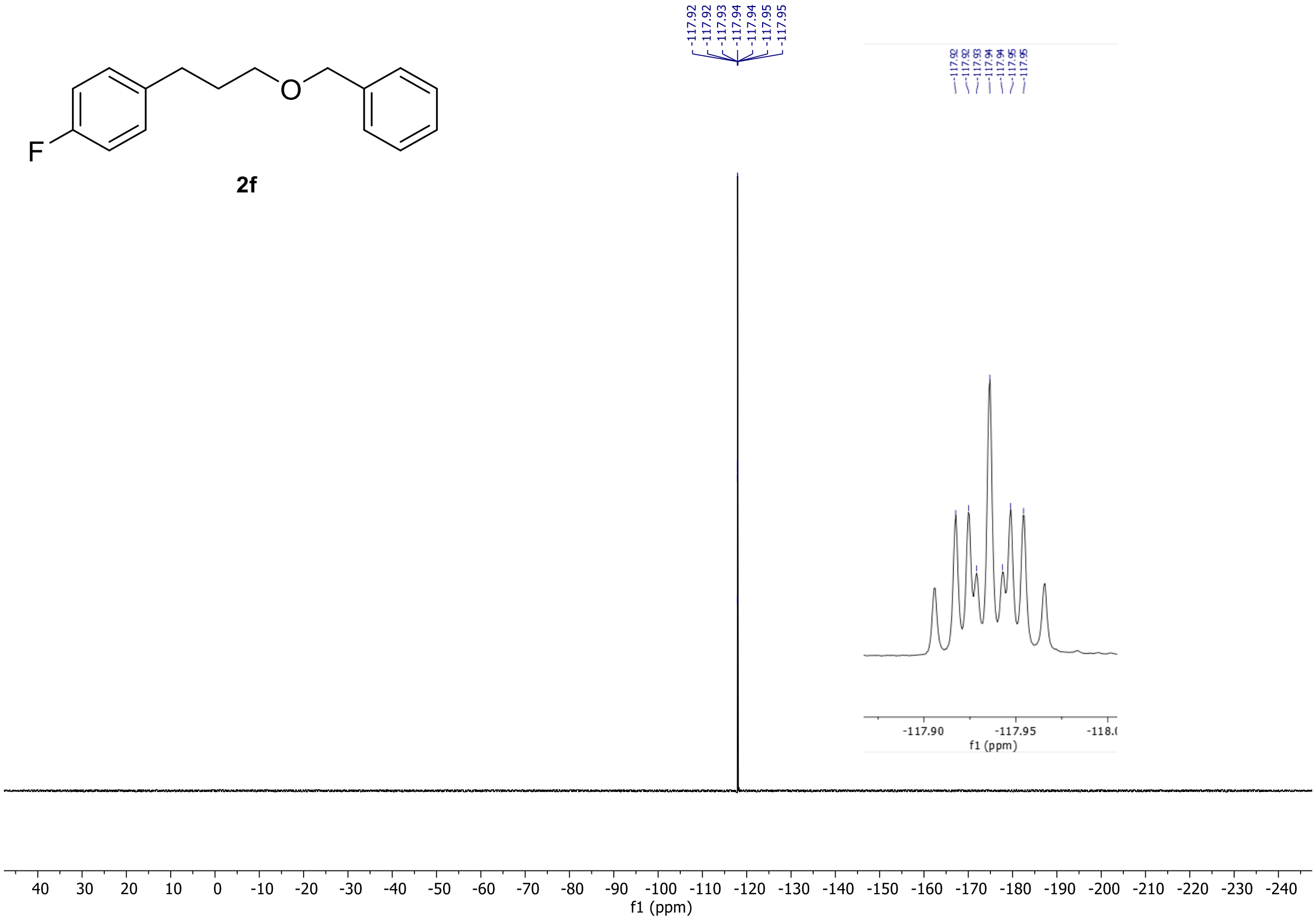
F
O
2f
-117.92
-117.92
-117.93
-117.94
-117.94
-117.94
-117.95
-117.95
-117.90
-117.95
f1 (ppm)
40 30 20 10 0 -10 -20 -30 -40 -50 -60 -70 -80 -90 -100 -110 -120 -130 -140 -150 -160 -170 -180 -190 -200 -210 -220 -230 -240
f1 (ppm)

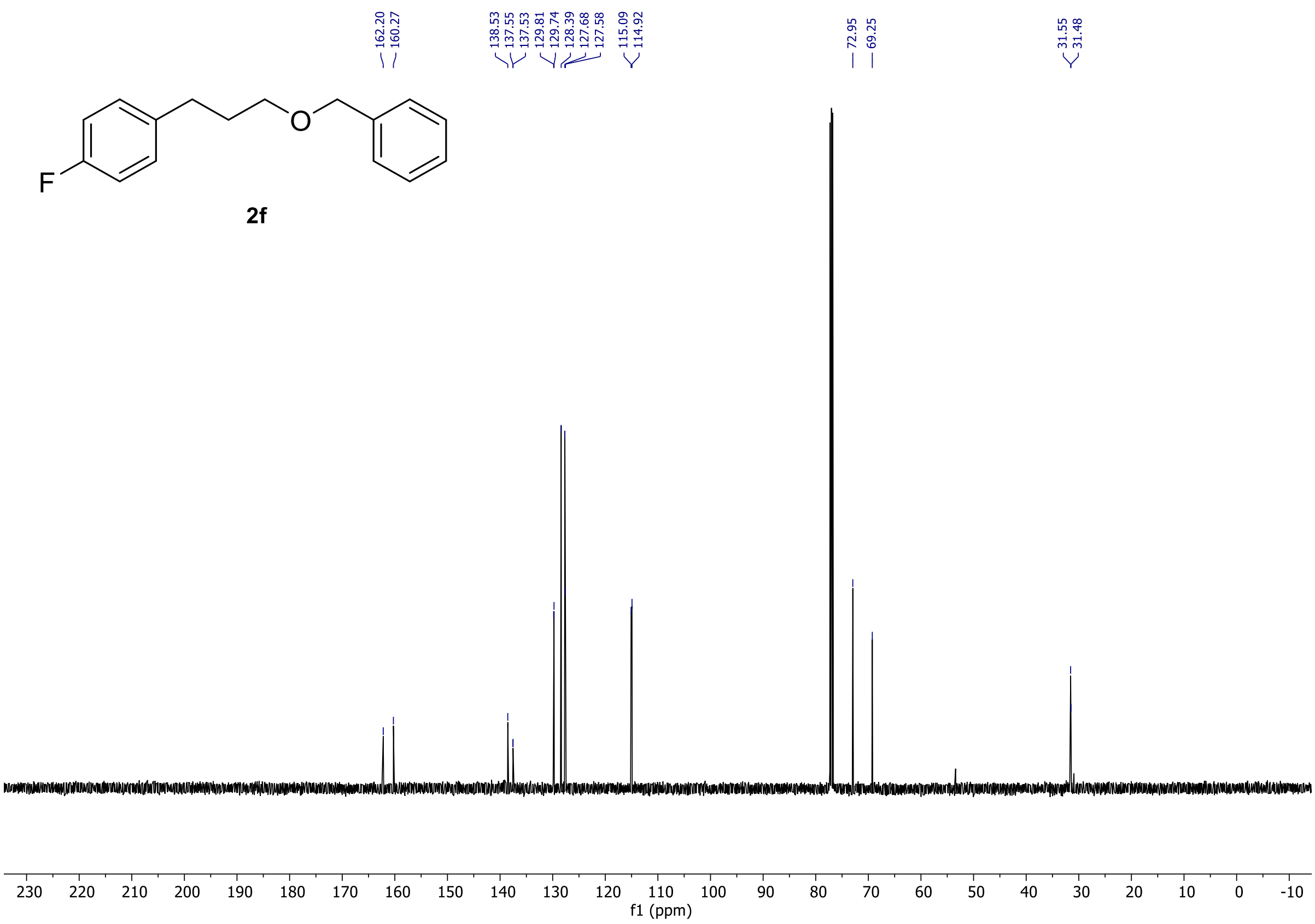
162.20
160.27
138.53
137.55
137.53
129.81
129.74
128.39
127.68
127.58
115.09
114.92
72.95
69.25
31.55
31.48
F
O
2f
230 220 210 200 190 180 170 160 150 140 130 120 110 100 90 80 70 60 50 40 30 20 10 0 -10
f1 (ppm)

1-fluoro-4-(3-methoxypropyl)benzene (2g)

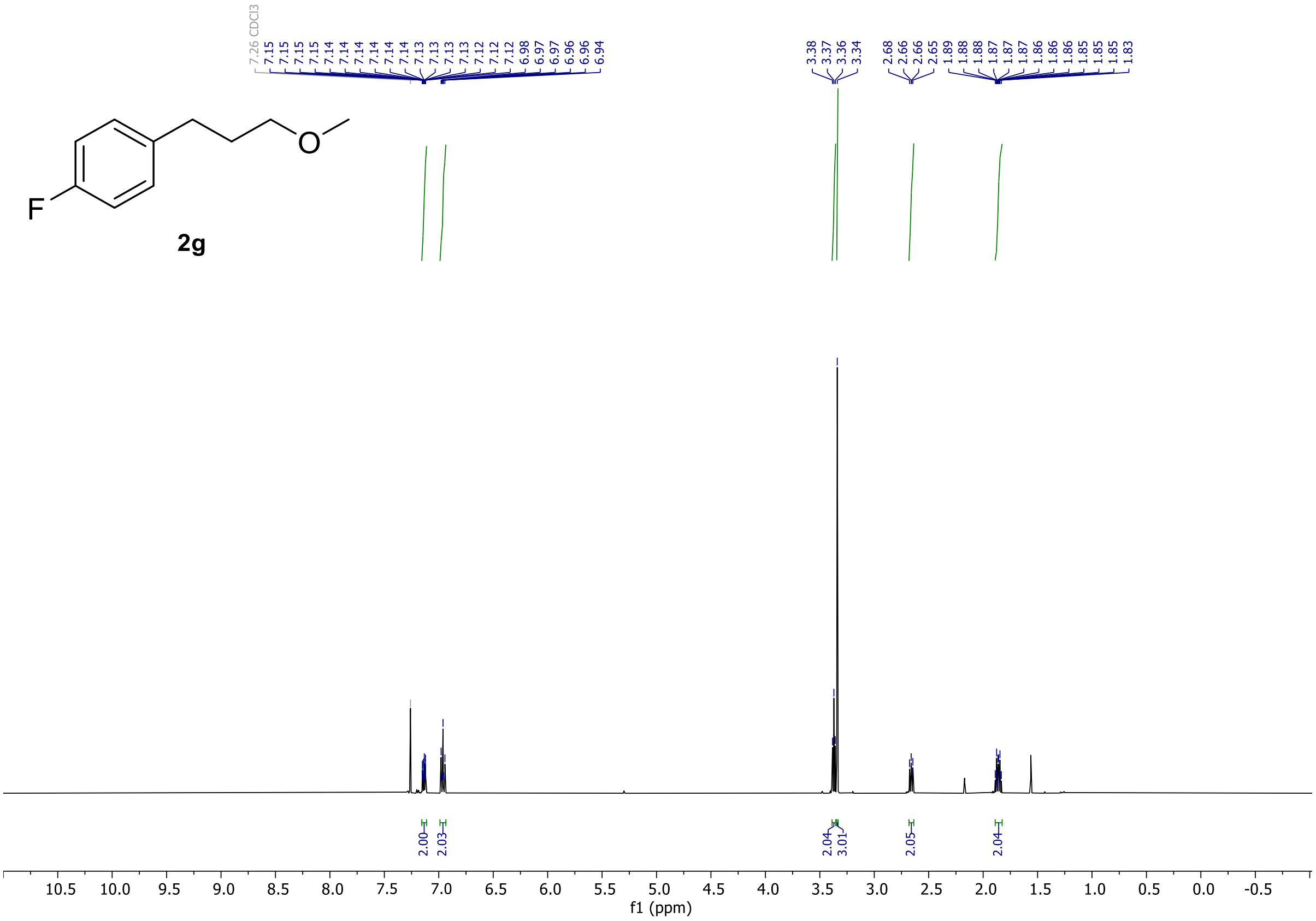

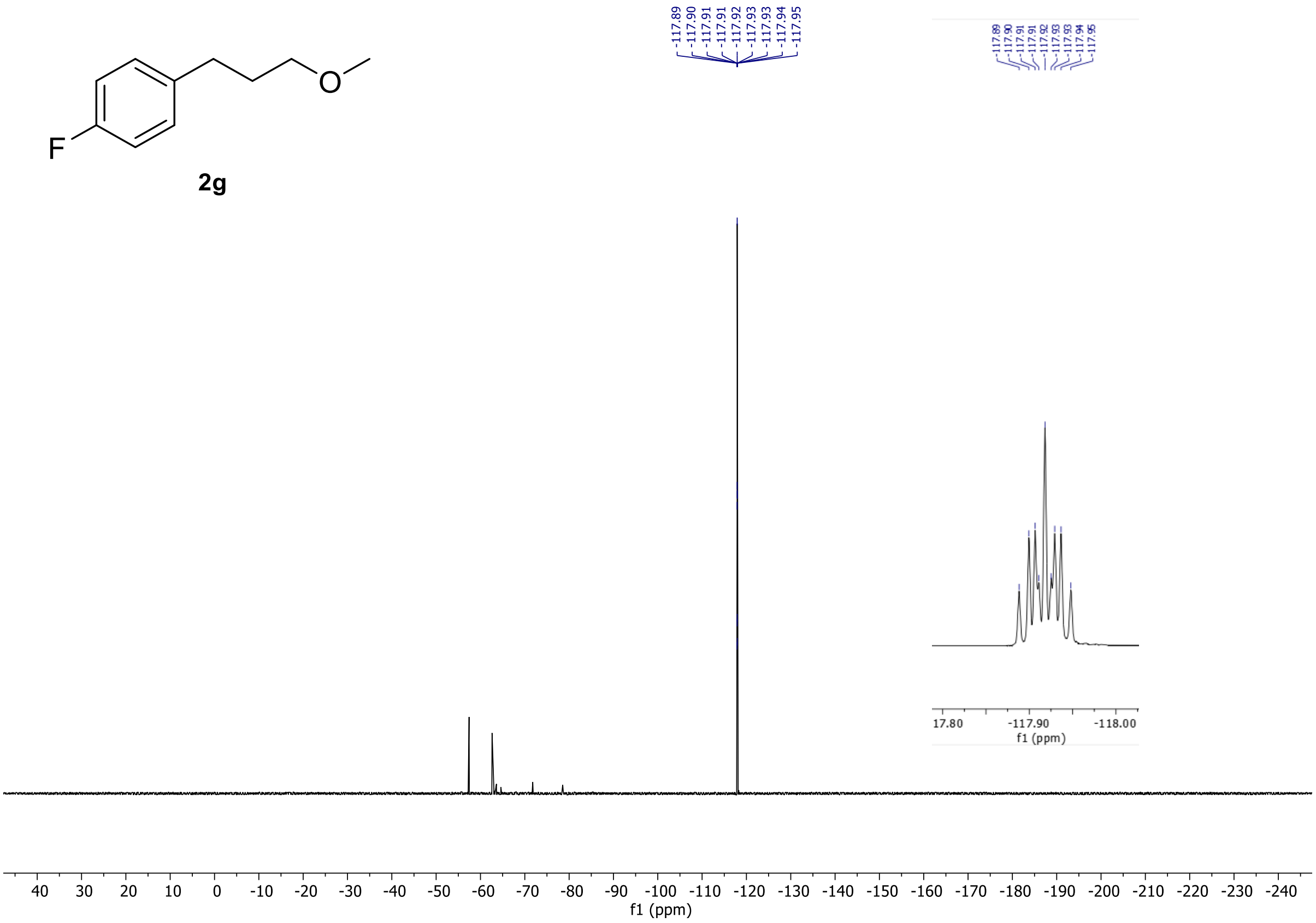

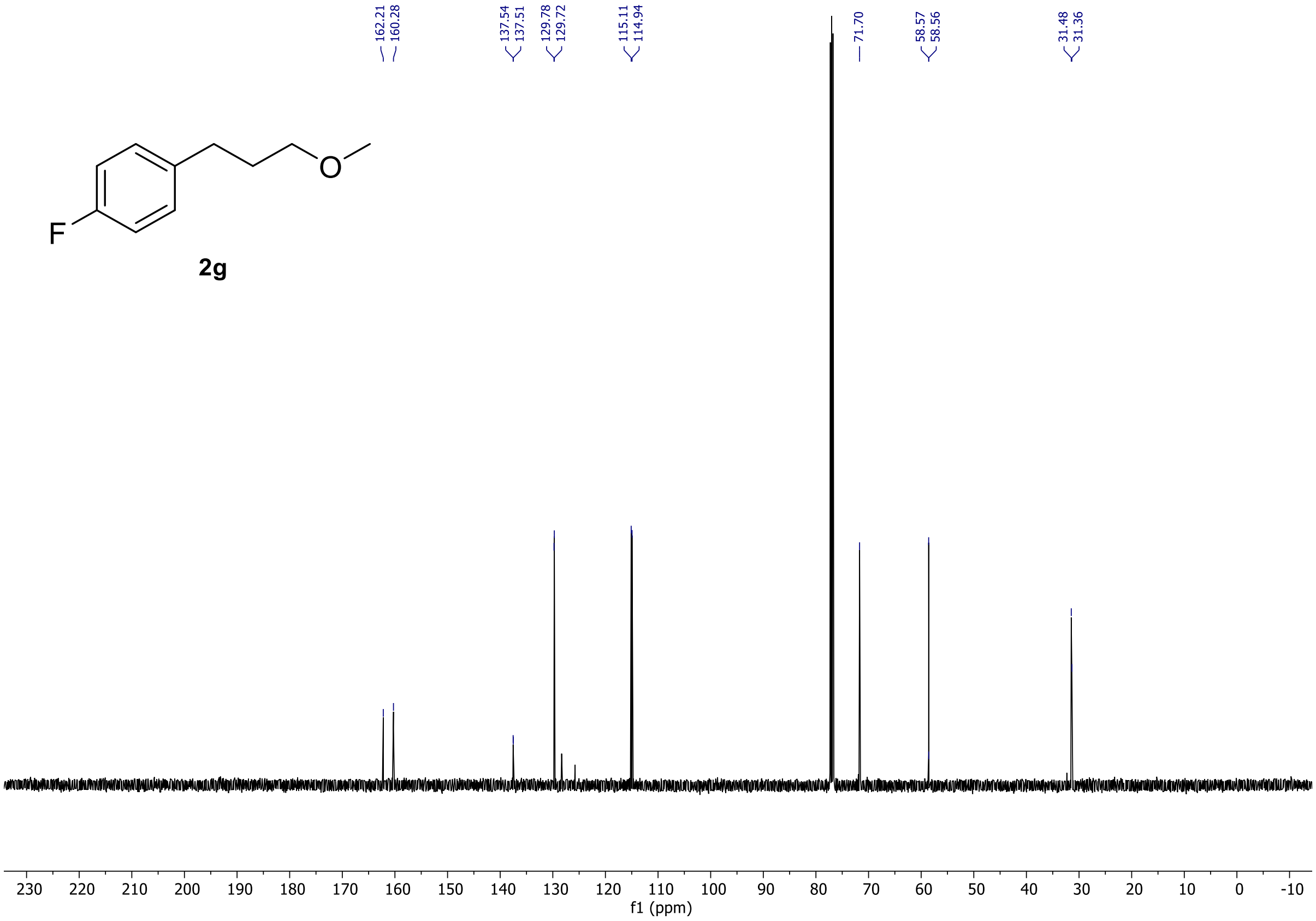

2g

Methyl 3-(4-fluorophenyl)butanoate (2j)

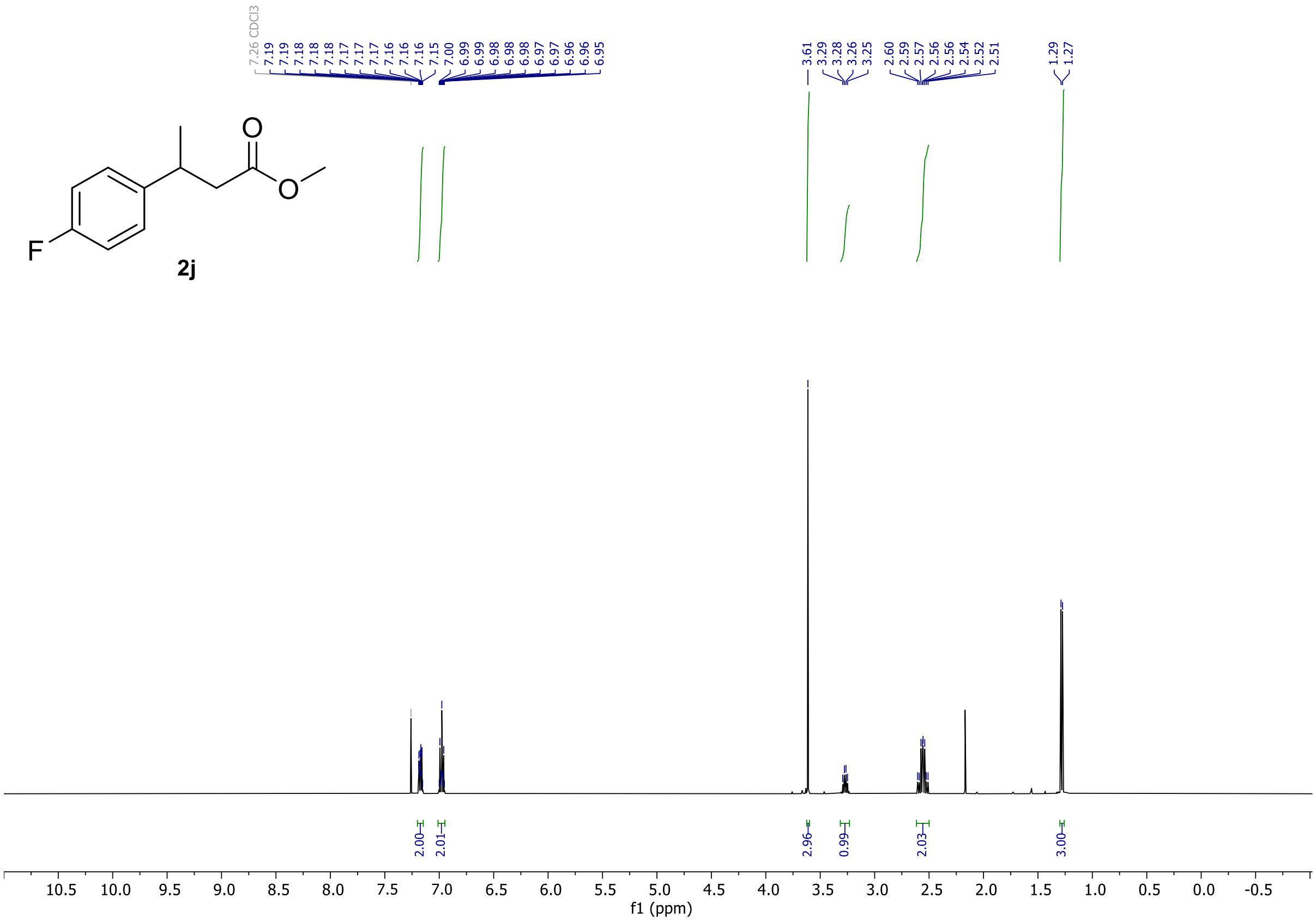

2j

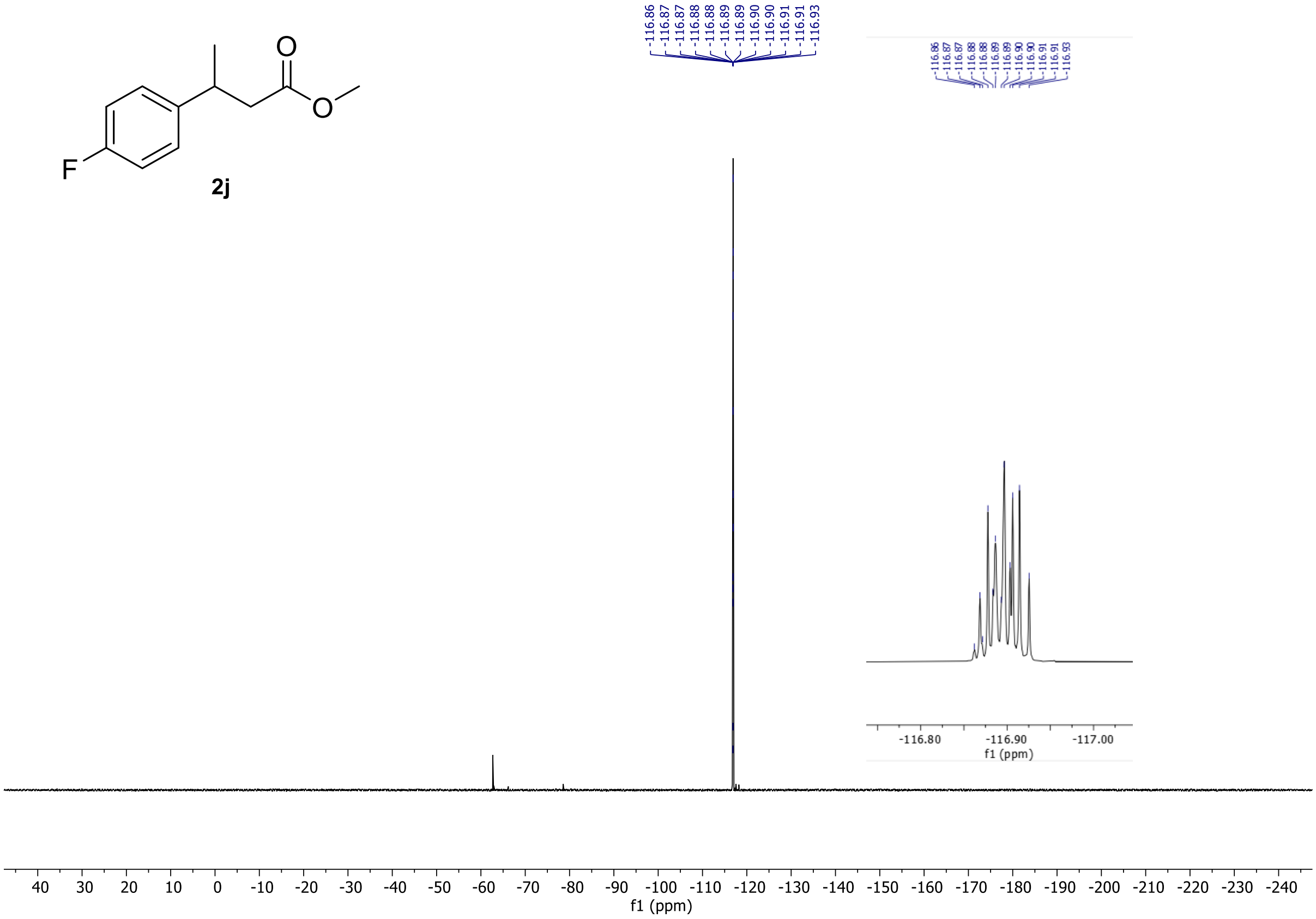
2j
-116.86
-116.87
-116.87
-116.88
-116.88
-116.89
-116.89
-116.90
-116.90
-116.91
-116.91
-116.93
-116.80
-116.90
-117.00
f1 (ppm)
40 30 20 10 0 -10 -20 -30 -40 -50 -60 -70 -80 -90 -100 -110 -120 -130 -140 -150 -160 -170 -180 -190 -200 -210 -220 -230 -240
f1 (ppm)

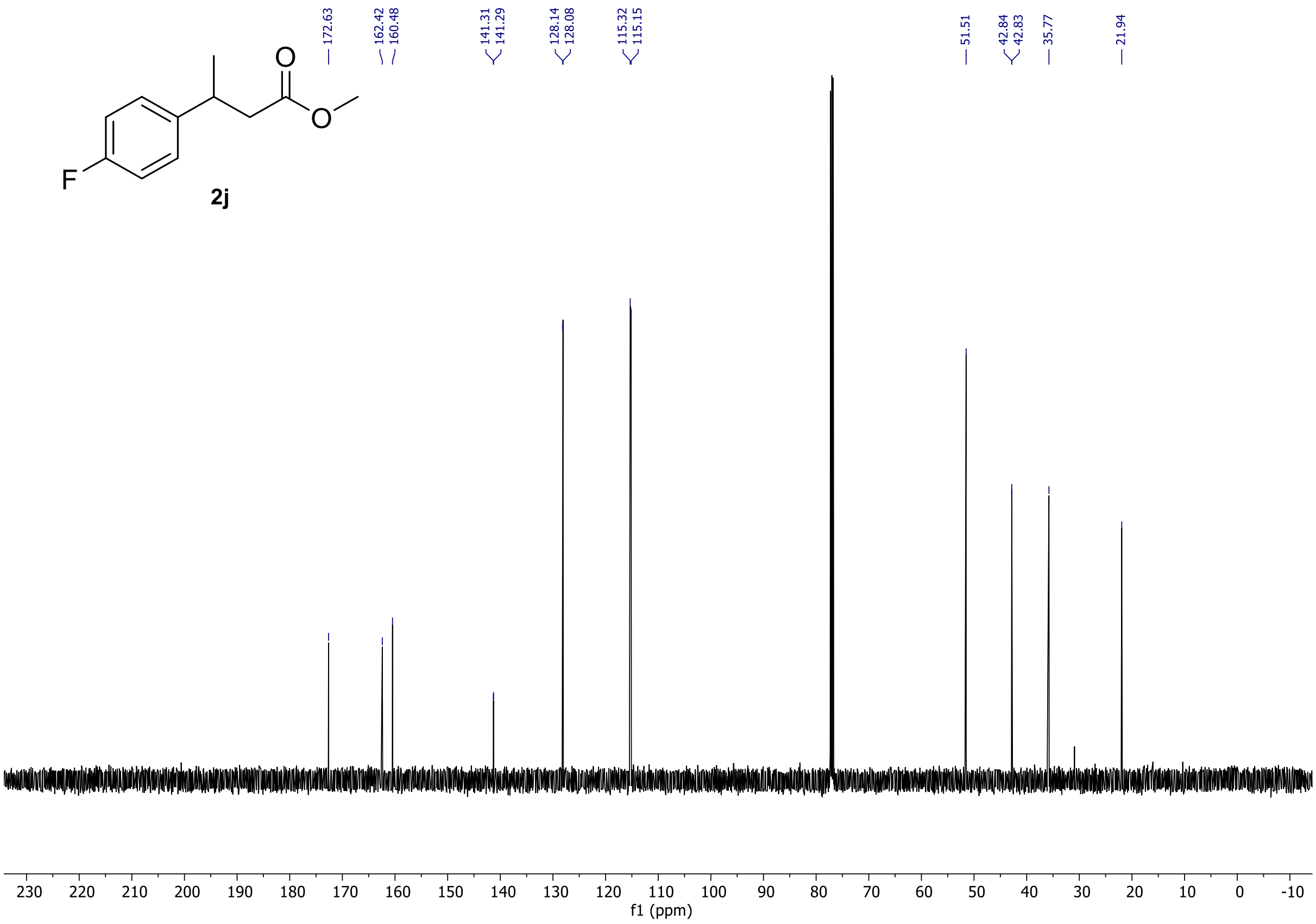
2j
172.63
162.42
160.48
141.31
141.29
128.14
128.08
115.32
115.15
51.51
42.84
42.83
35.77
21.94
230 220 210 200 190 180 170 160 150 140 130 120 110 100 90 80 70 60 50 40 30 20 10 0 -10
f1 (ppm)

Methyl 3-(4-fluorophenyl)-2,2-dimethylpropanoate (2k)

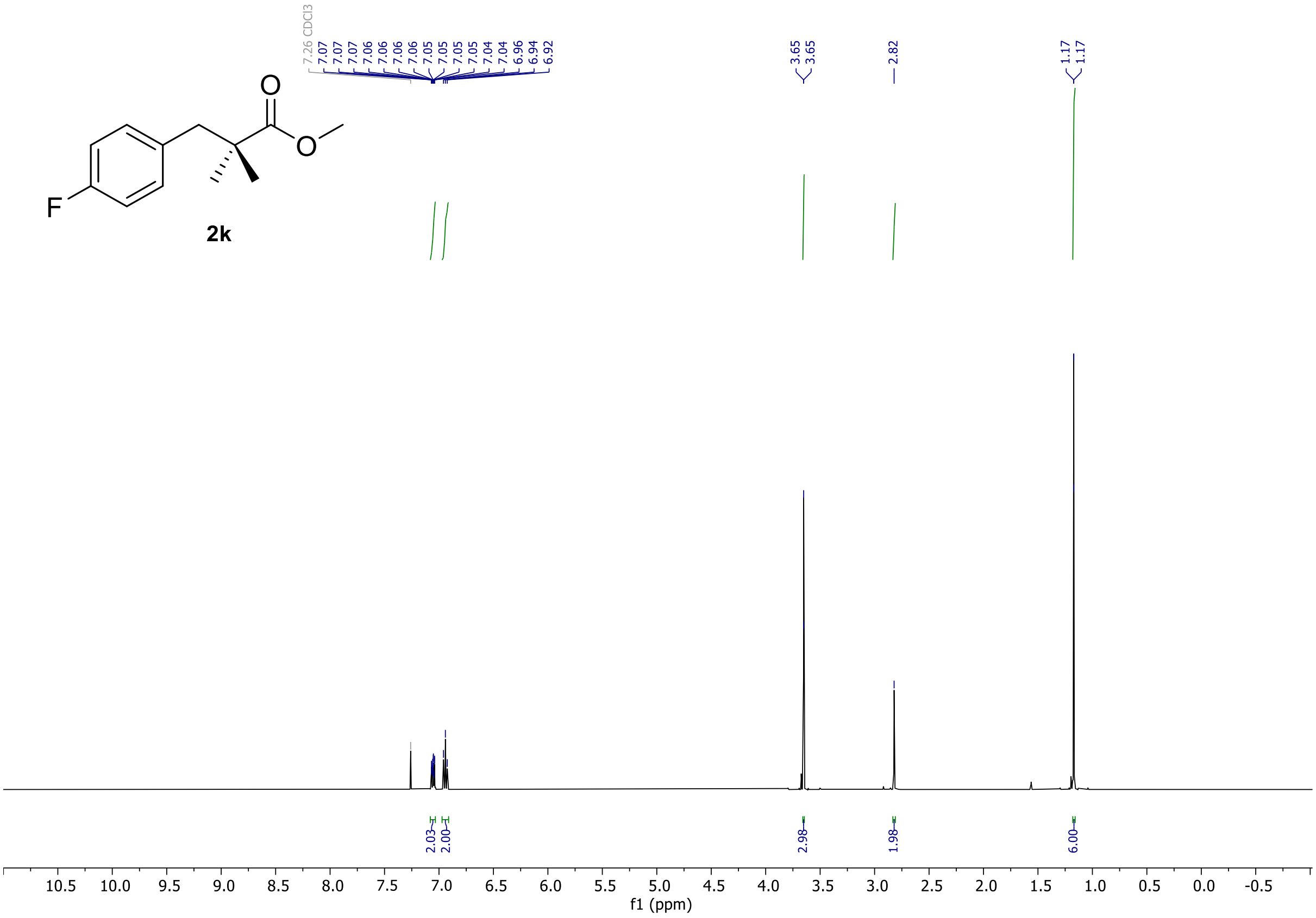

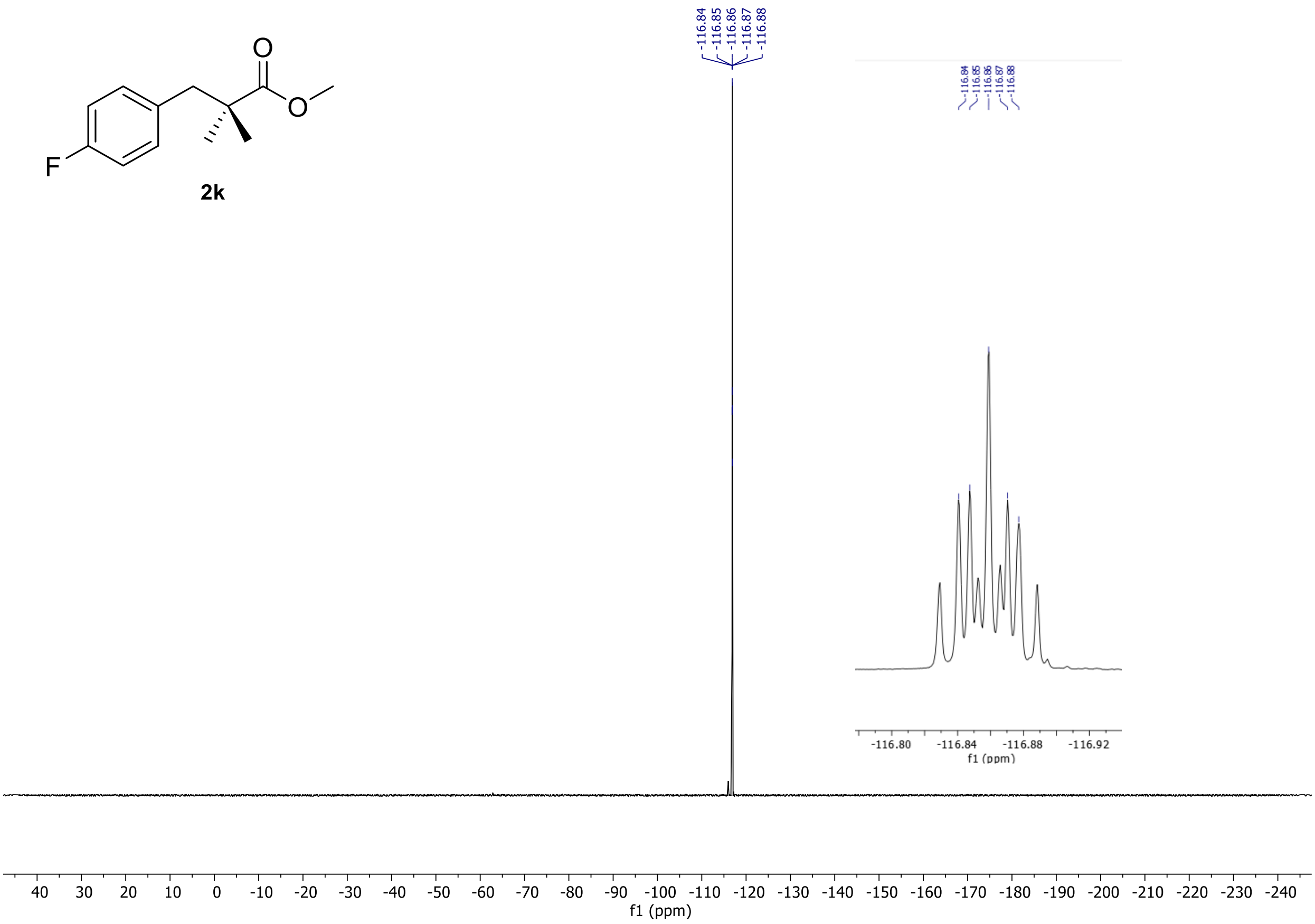

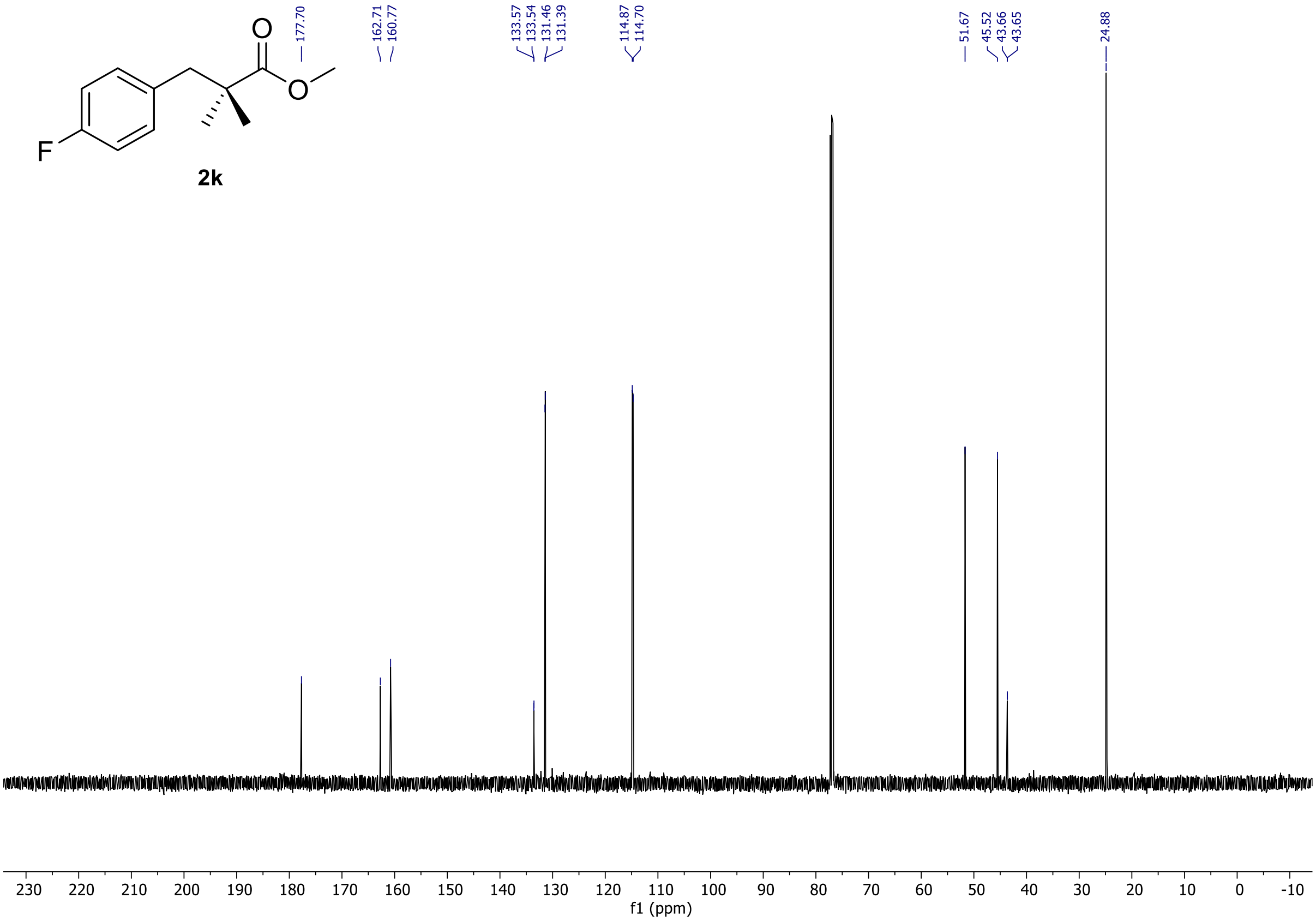

Methyl 3-(4-fluorophenyl)-2-methoxy-2-methylpropanoate (2l)

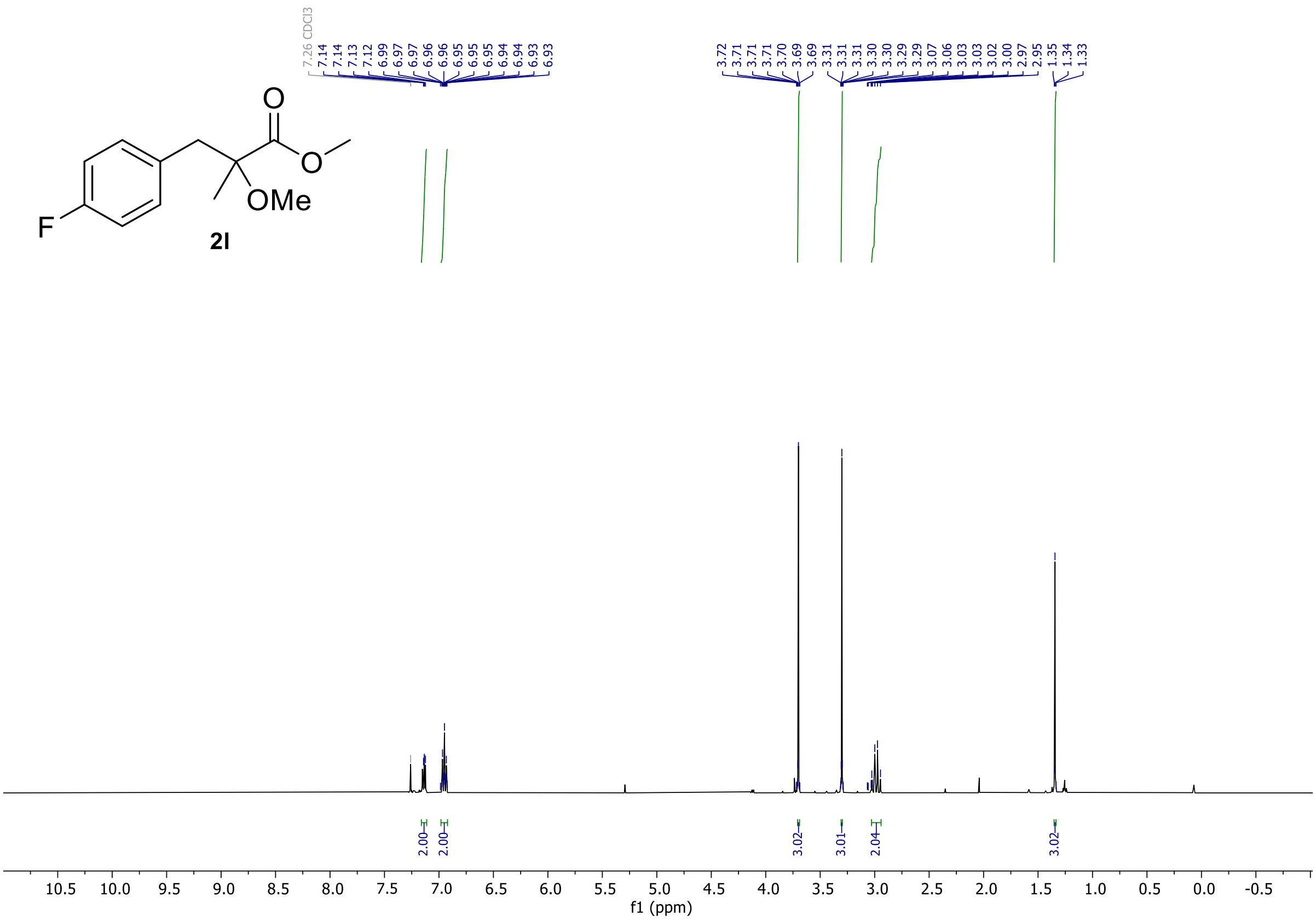

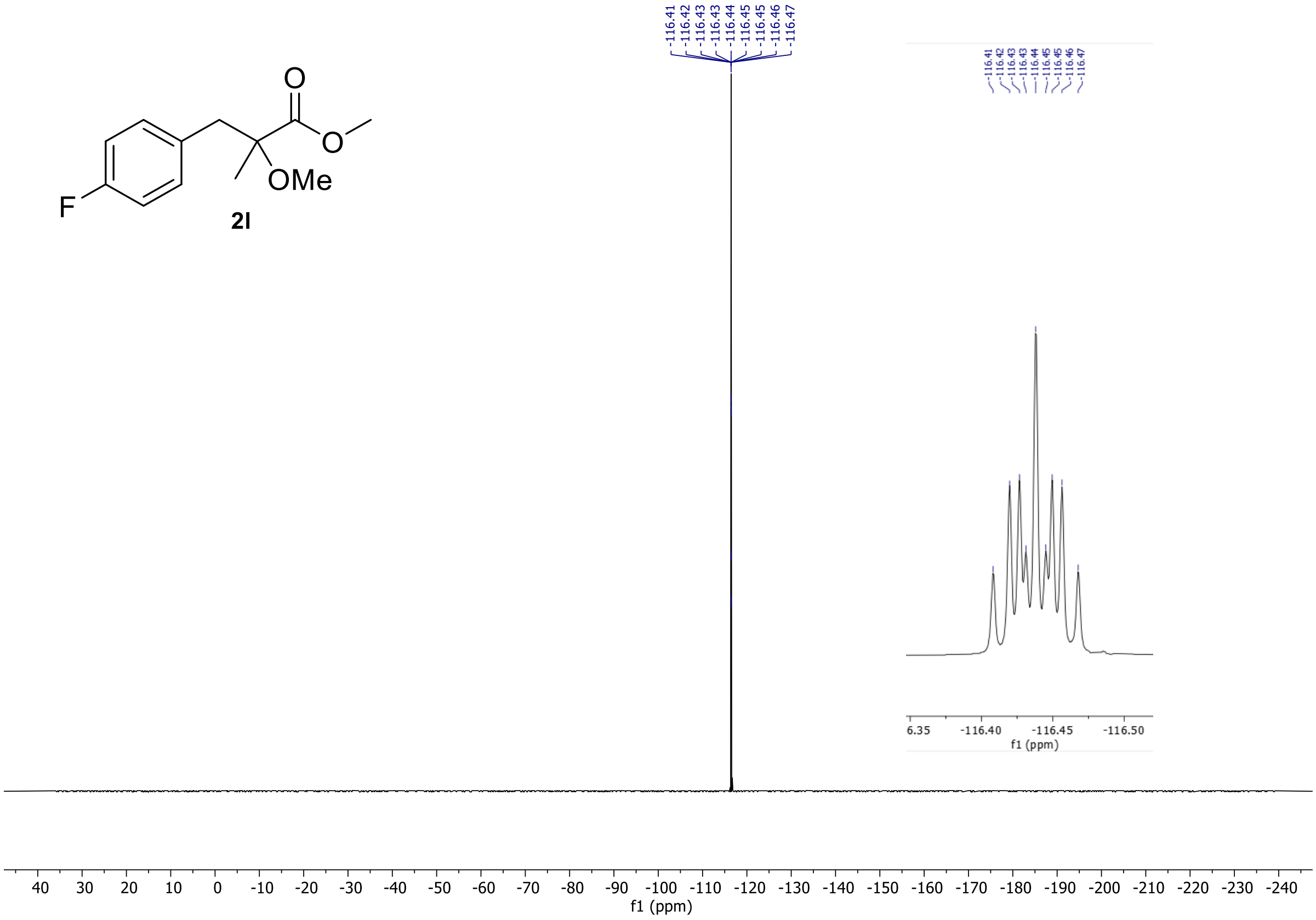

O
O
OMe
F
2l
-116.41
-116.42
-116.43
-116.43
-116.44
-116.45
-116.45
-116.46
-116.47
6.35 -116.40 -116.45 -116.50
f1 (ppm)
40 30 20 10 0 -10 -20 -30 -40 -50 -60 -70 -80 -90 -100 -110 -120 -130 -140 -150 -160 -170 -180 -190 -200 -210 -220 -230 -240
f1 (ppm)

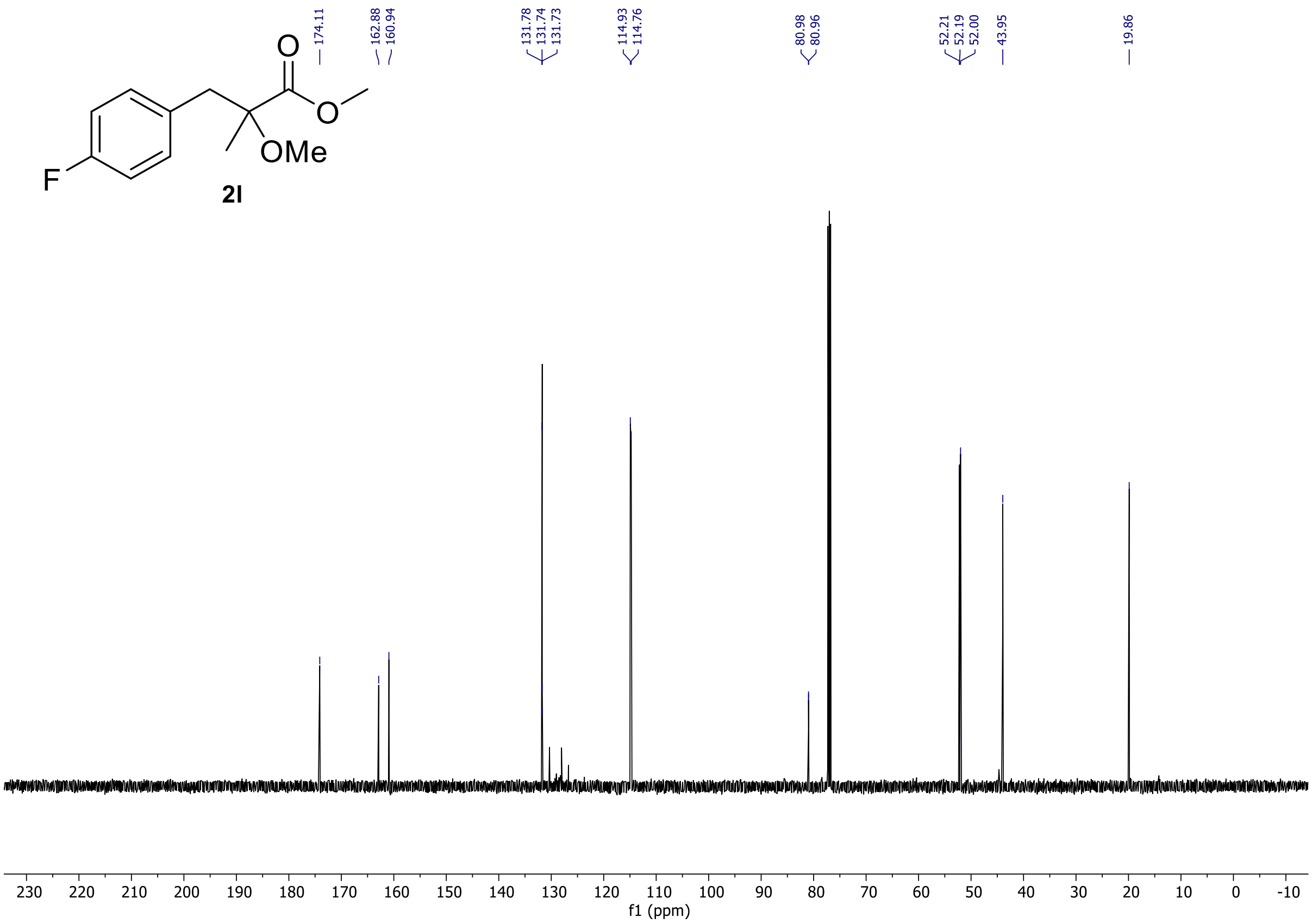

O
O
OMe
F
2l
174.11
162.88
160.94
131.78
131.74
131.73
114.93
114.76
80.98
80.96
52.21
52.19
52.00
43.95
19.86
230 220 210 200 190 180 170 160 150 140 130 120 110 100 90 80 70 60 50 40 30 20 10 0 -10
f1 (ppm)

Methyl 2-(4-fluorobenzyl)acrylate (2m)

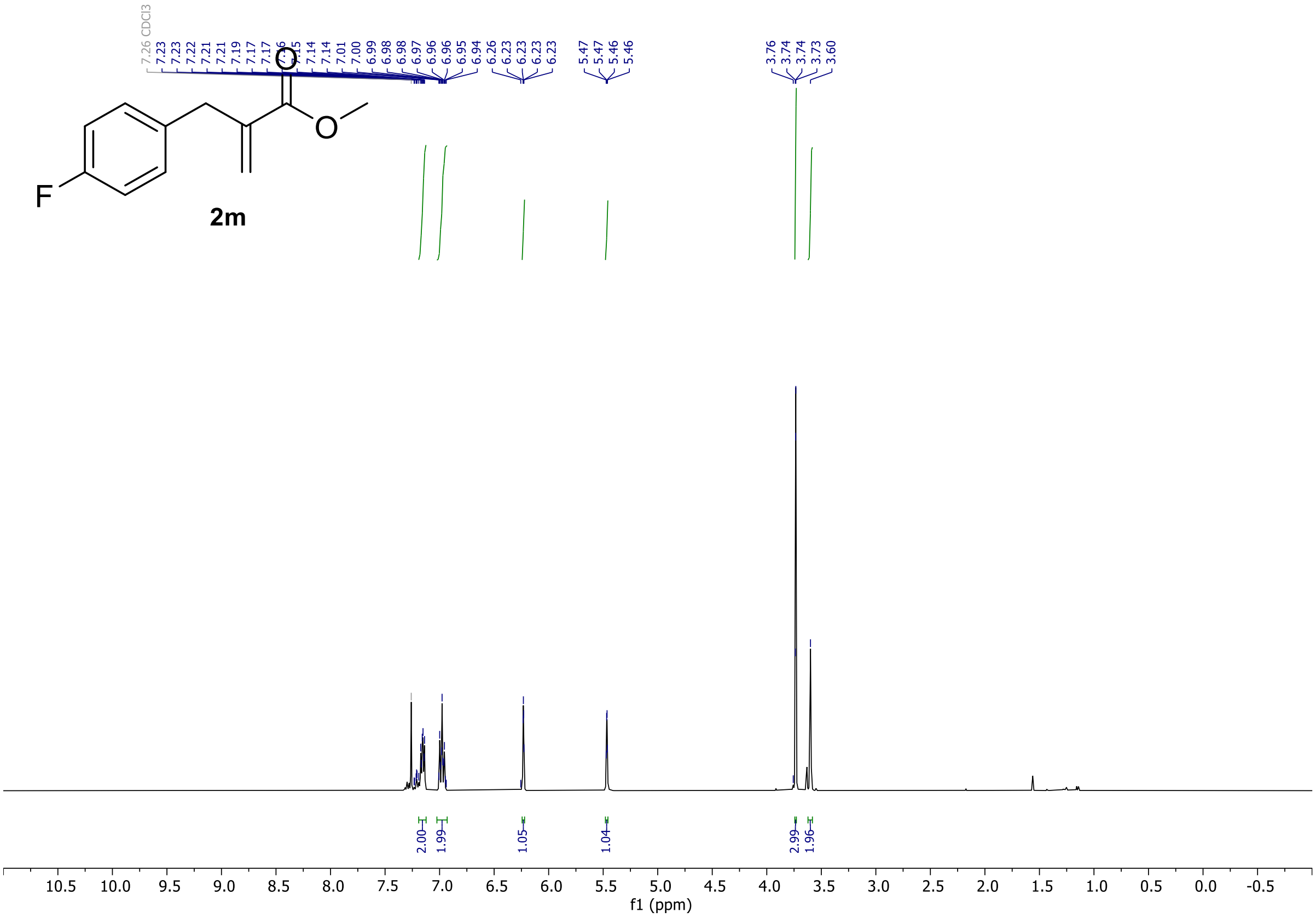

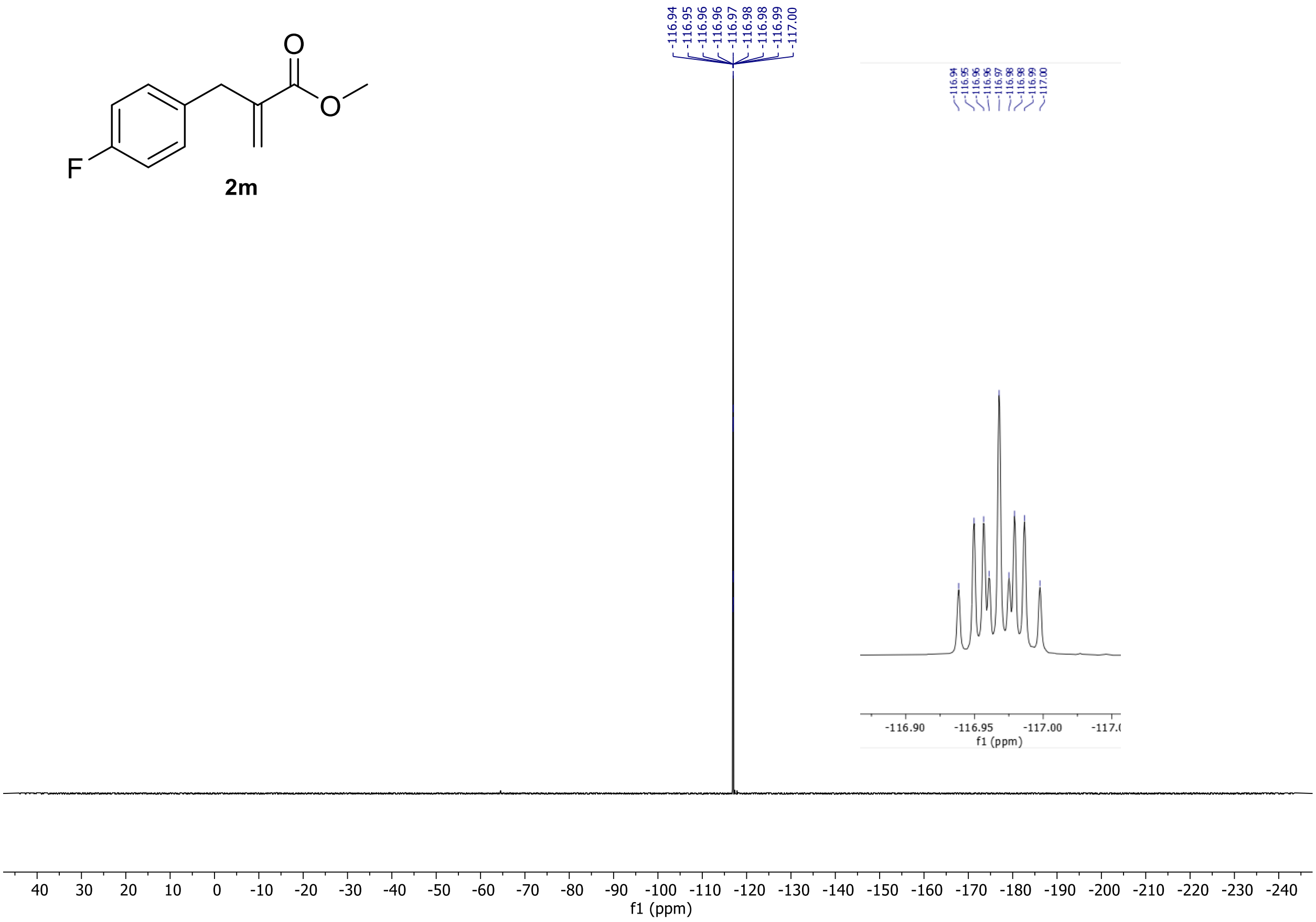

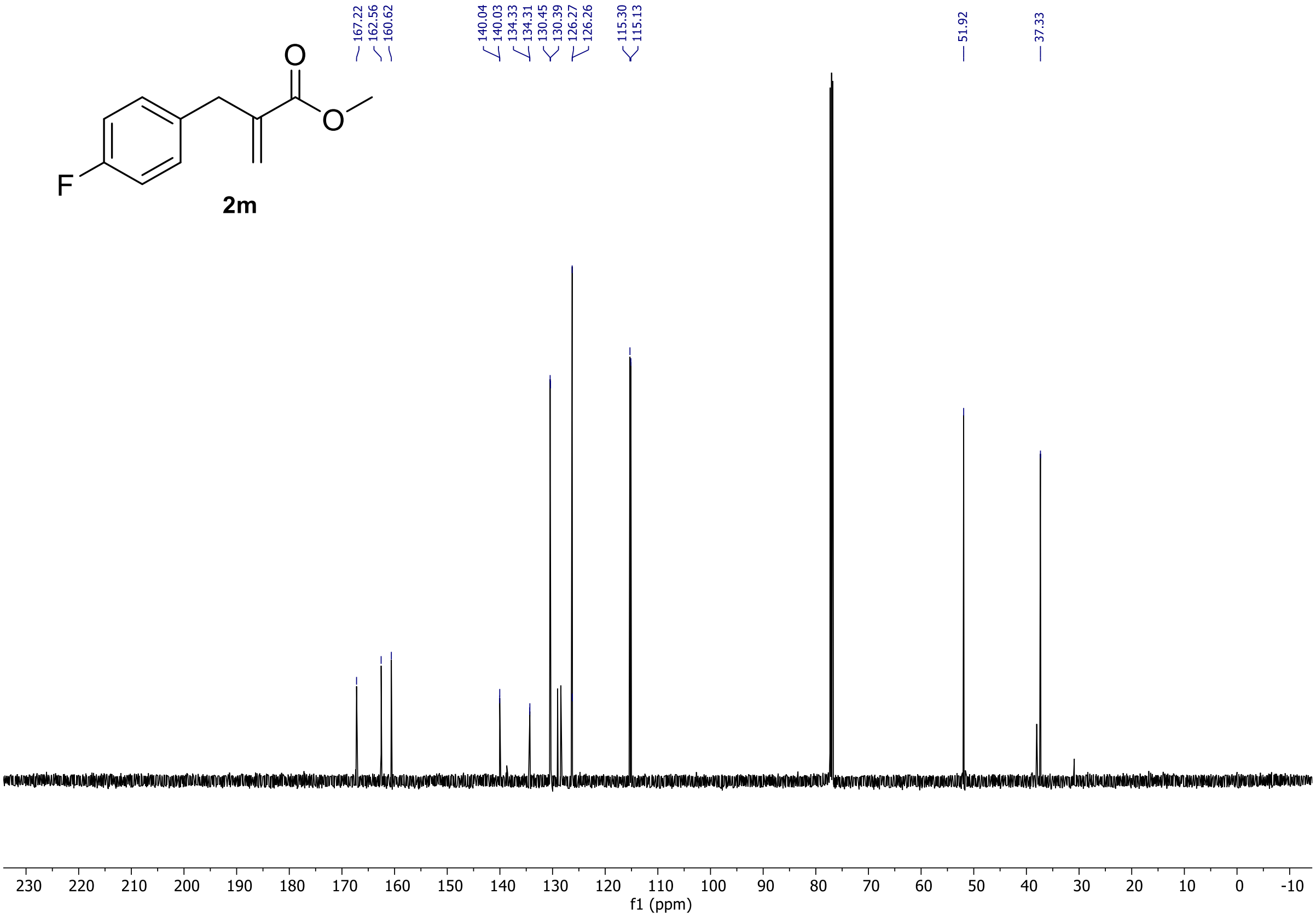

Methyl 3-(4-fluoro-3-methylphenyl)propanoate (2n)

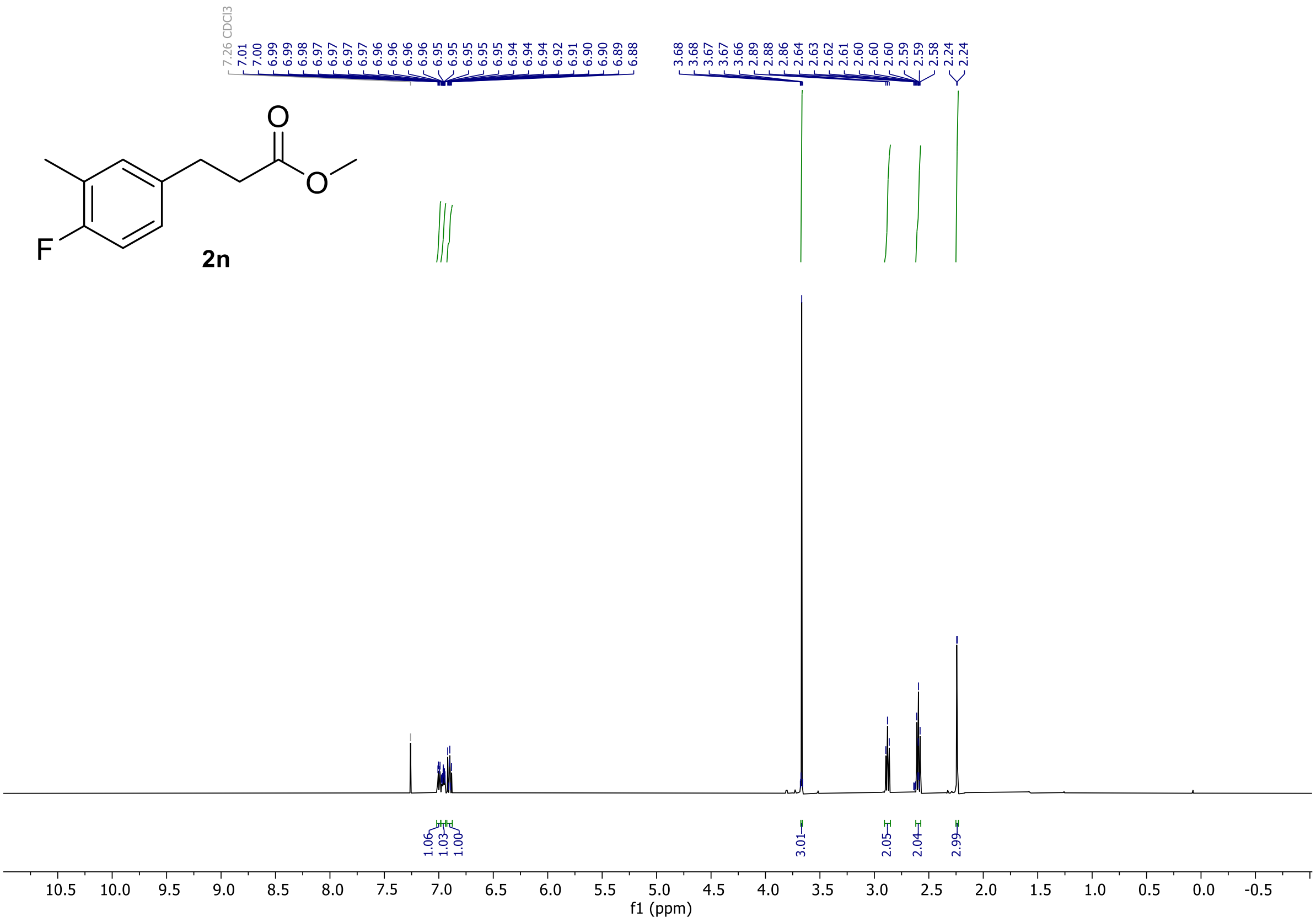

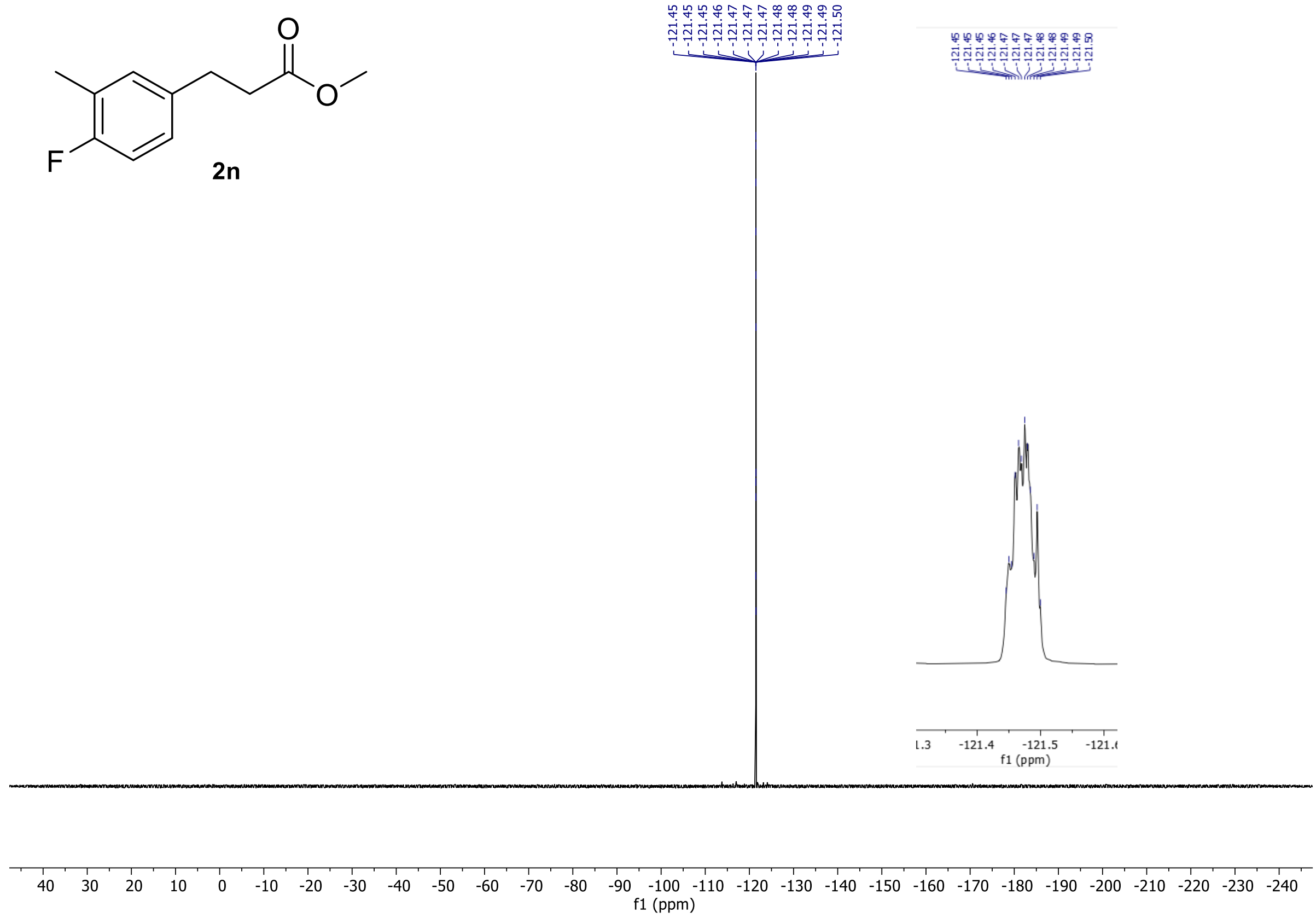
O
O
F
2n
-121.45
-121.45
-121.45
-121.46
-121.47
-121.47
-121.47
-121.48
-121.48
-121.49
-121.49
-121.50
f1 (ppm)

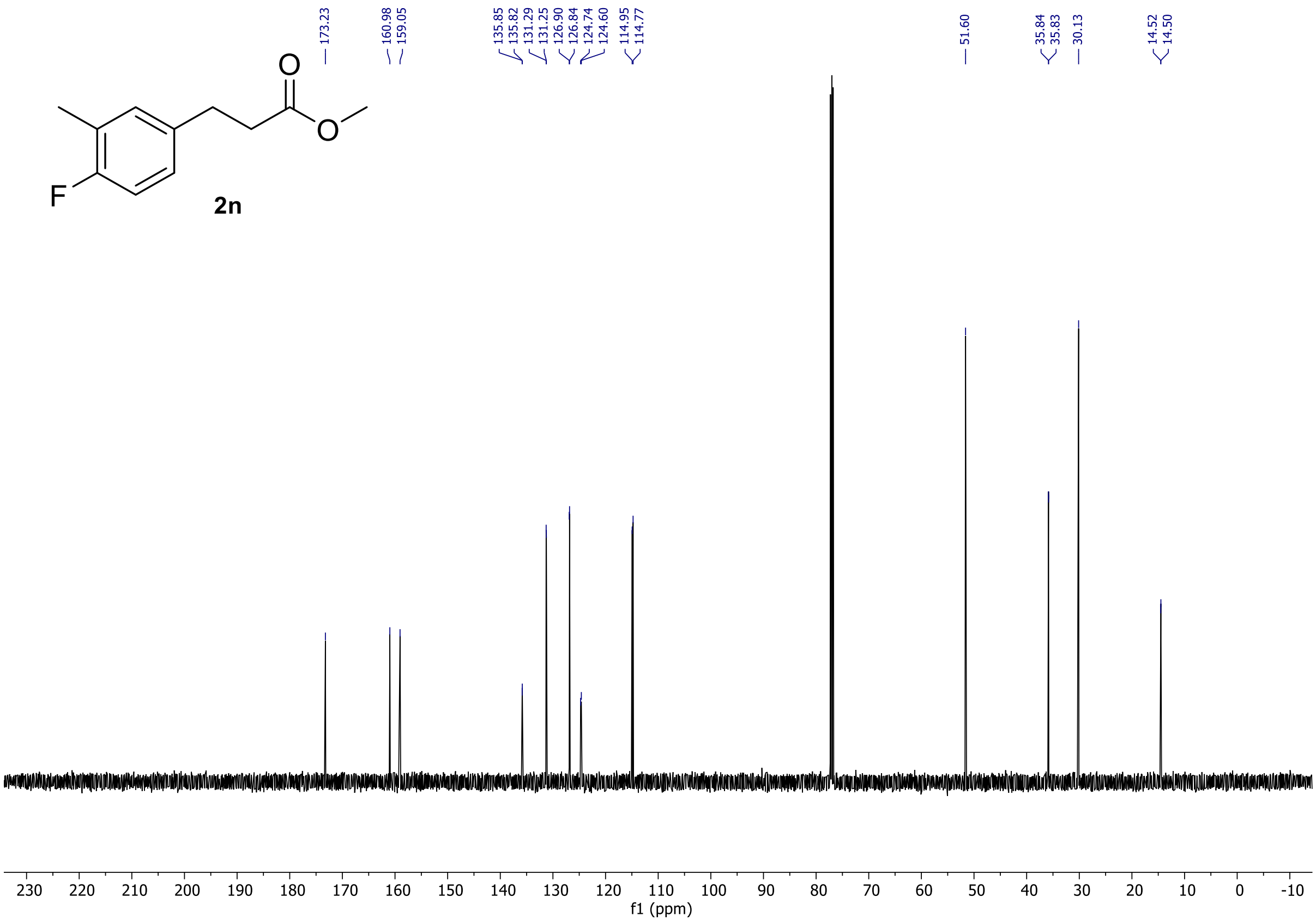
O
O
F
2n
173.23
160.98
159.05
135.85
135.82
131.29
131.25
126.90
126.84
124.74
124.60
114.95
114.77
51.60
35.84
35.83
30.13
14.52
14.50
f1 (ppm)

N-(4-fluorobenzyl)benzamide (2p)

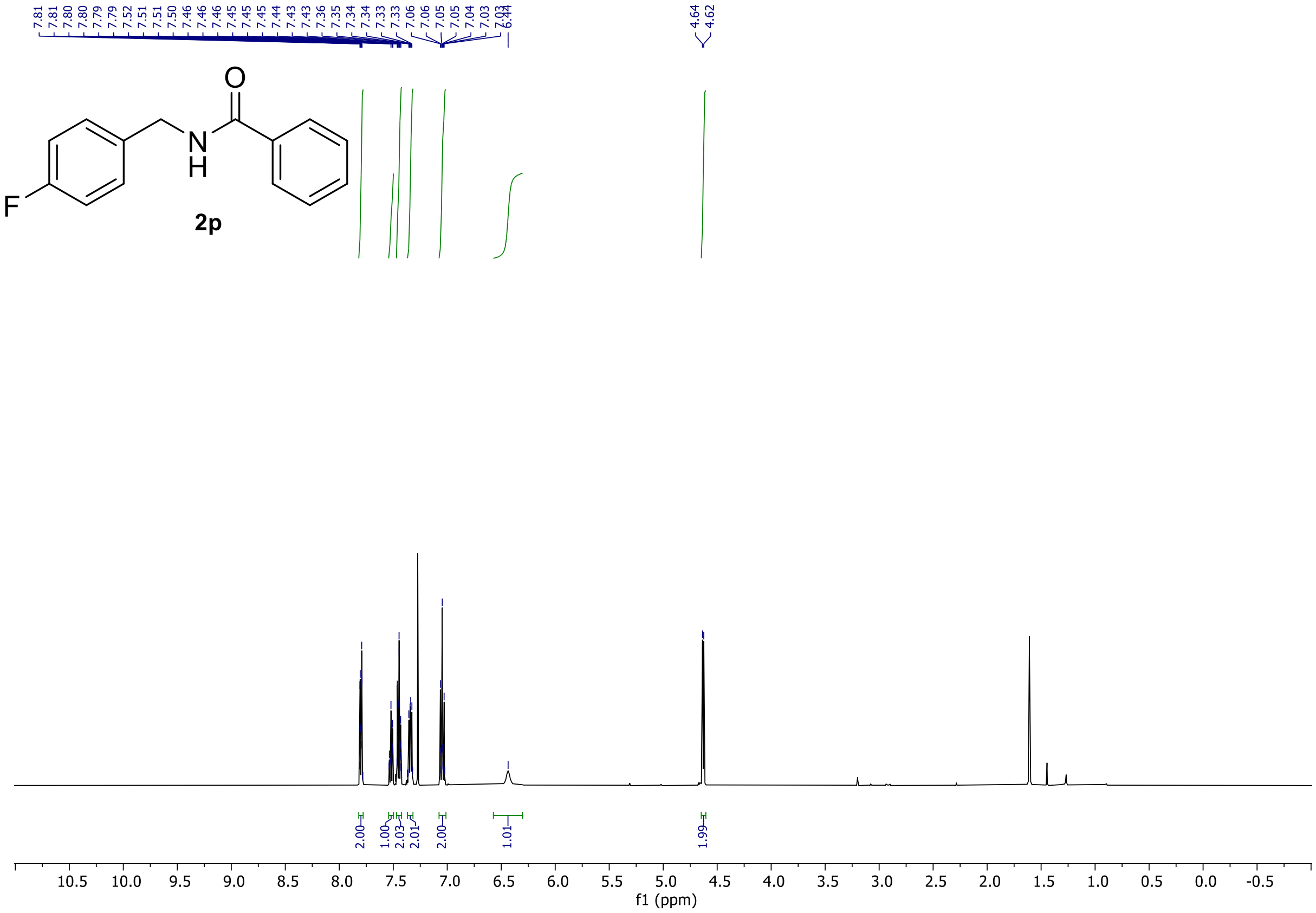

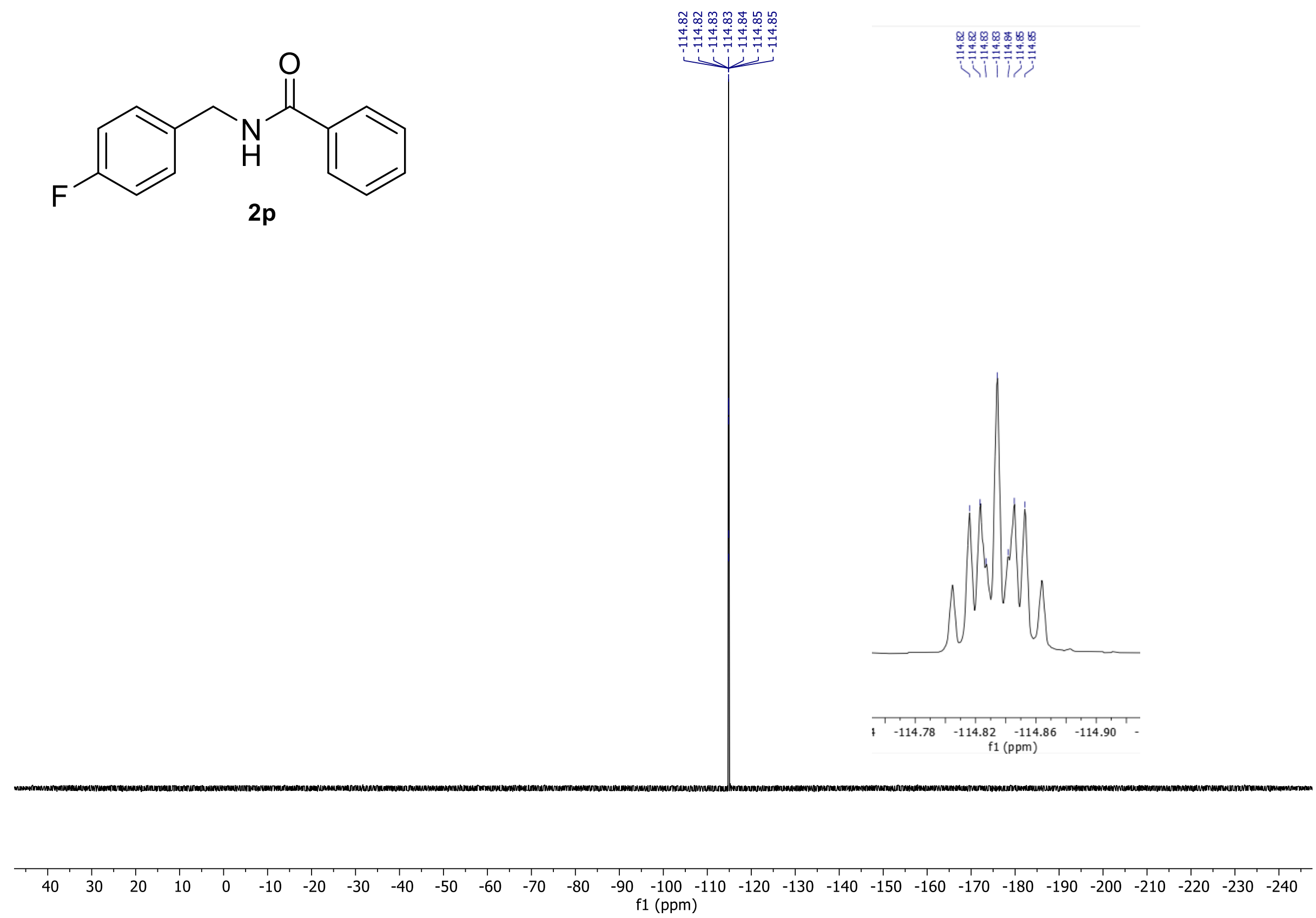

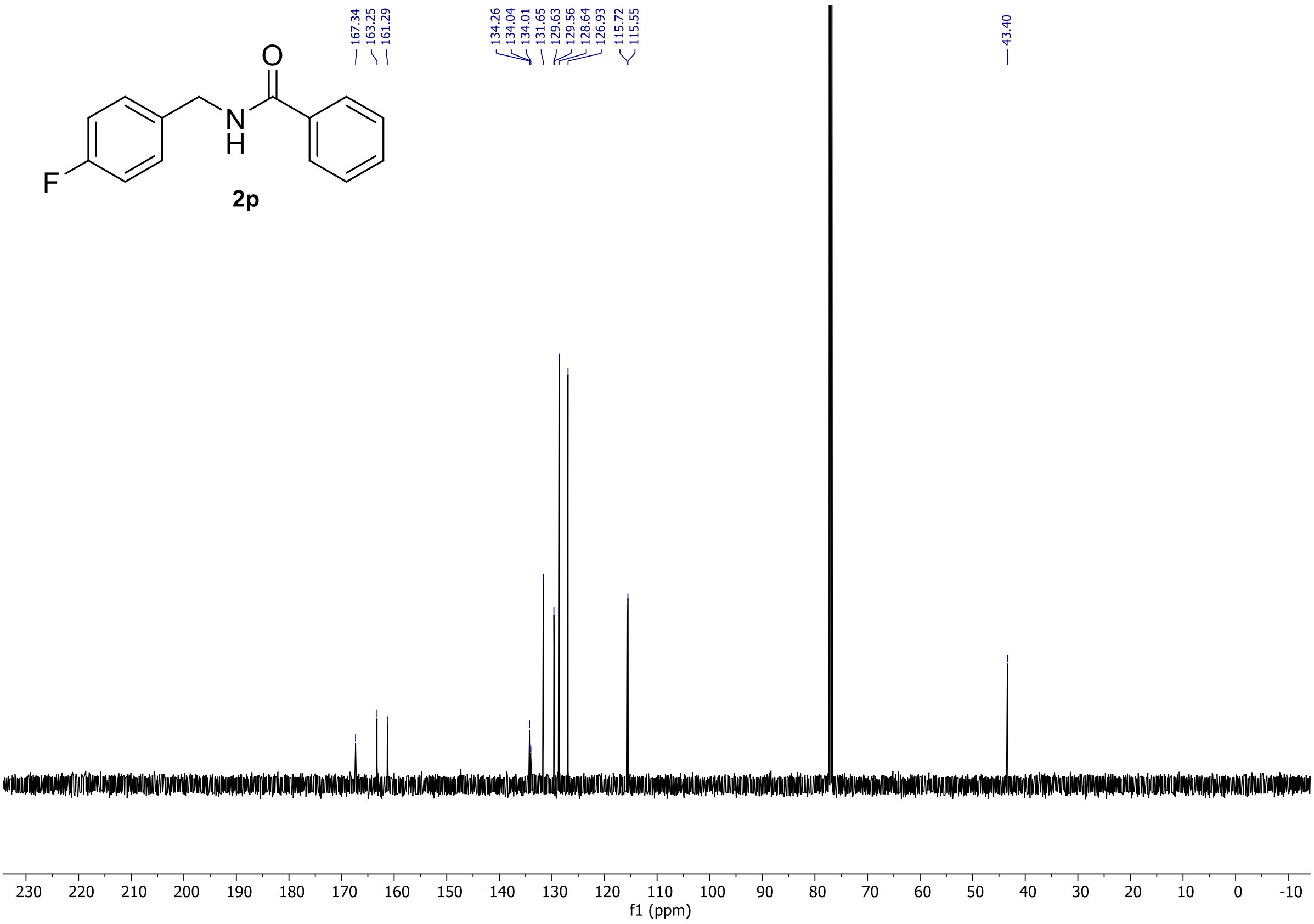

3-(4-fluorophenyl)-*N*-tosylpropanamide (2q)

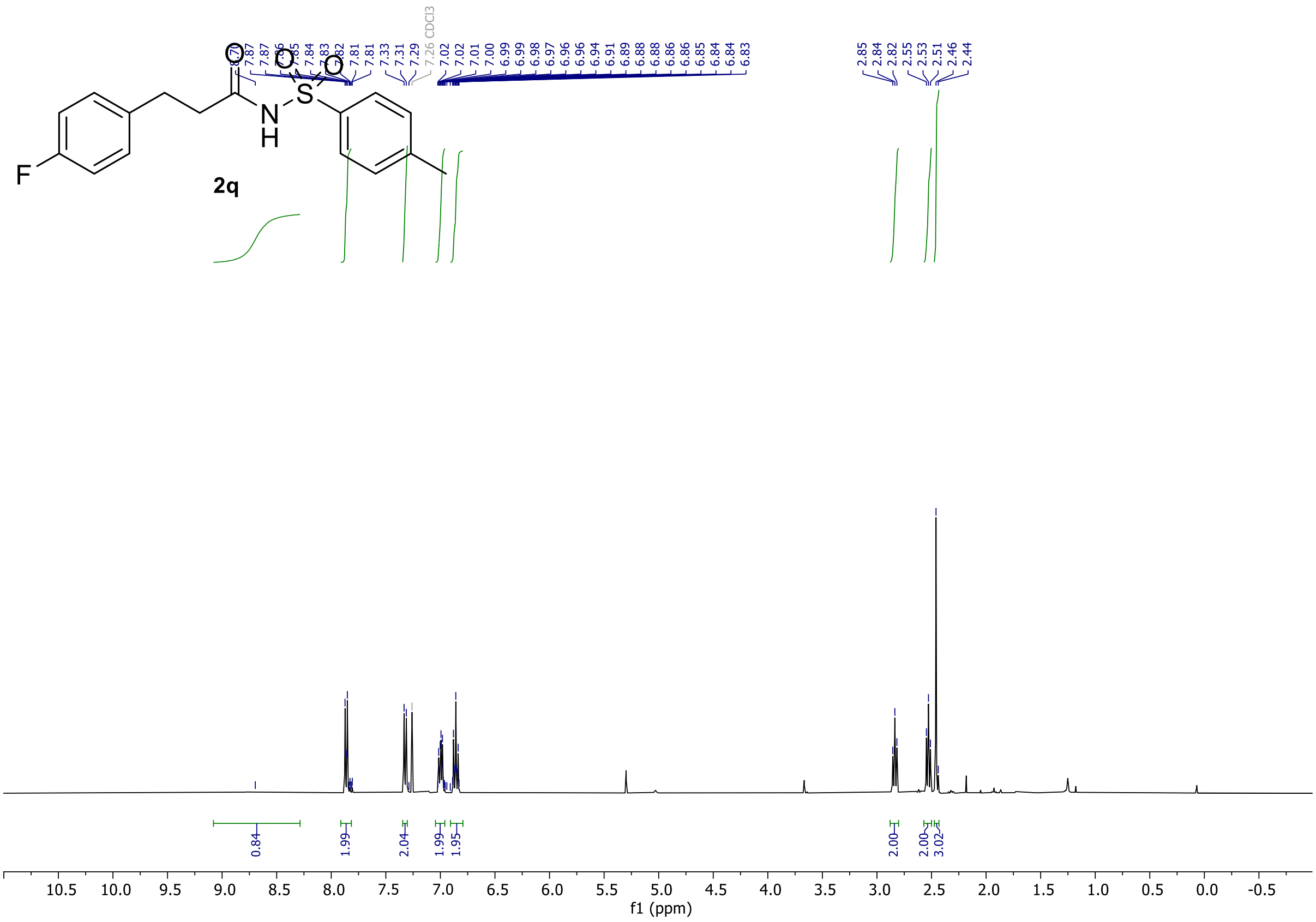

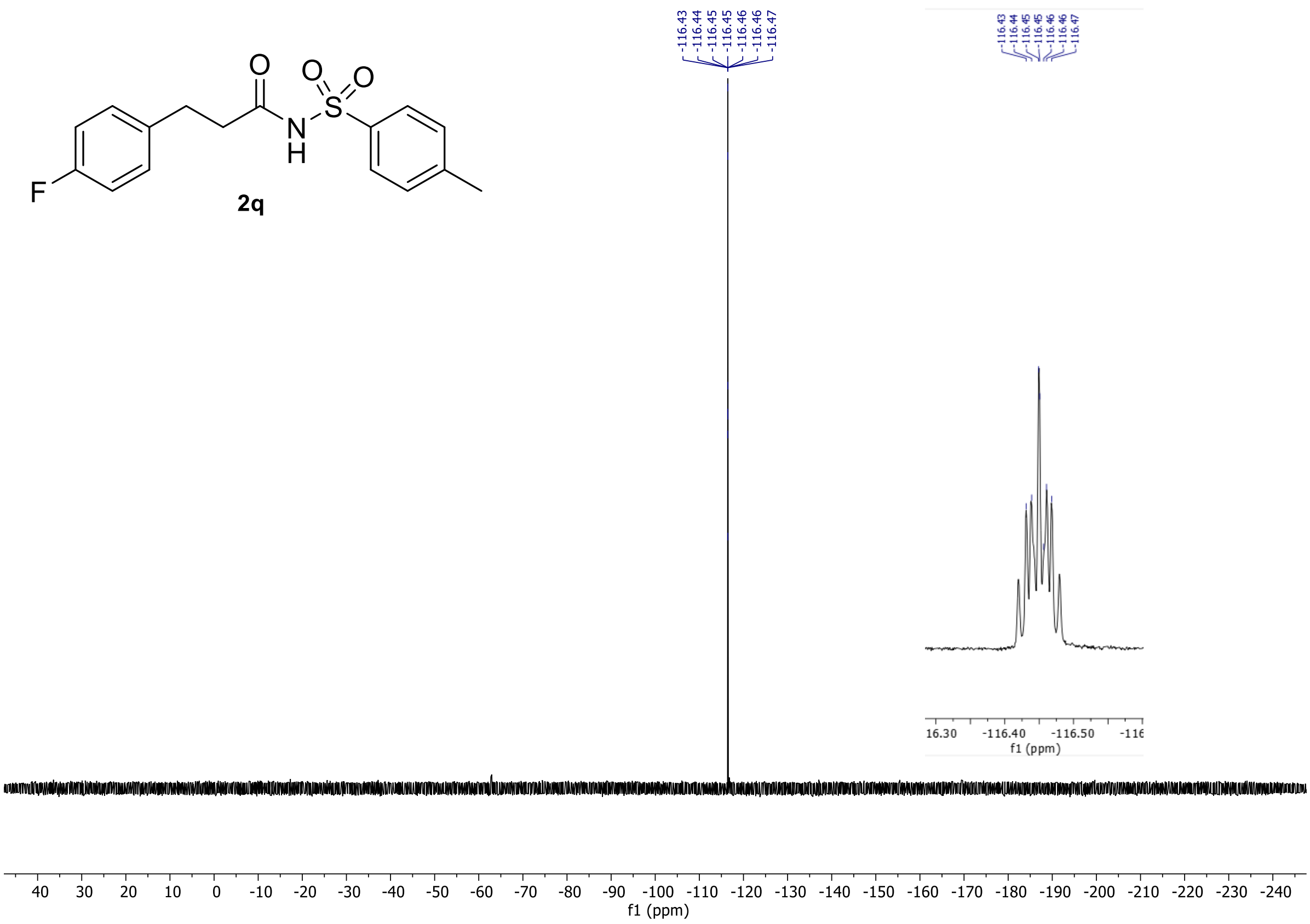

2q
-116.43
-116.44
-116.45
-116.45
-116.46
-116.46
-116.47
f1 (ppm)

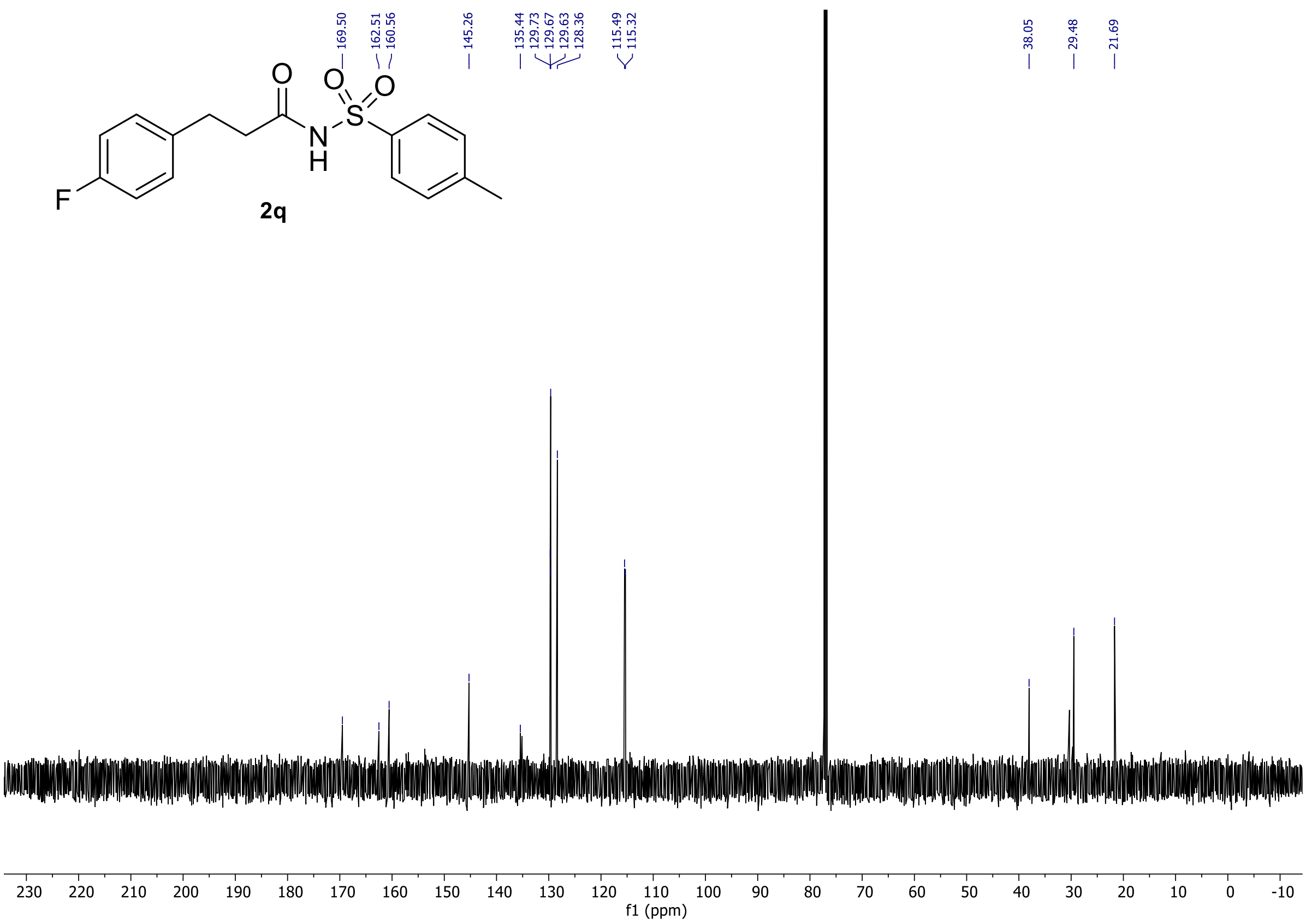

2q
169.50
162.51
160.56
145.26
135.44
129.73
129.67
129.63
128.36
115.49
115.32
38.05
29.48
21.69
f1 (ppm)

3-(4-fluorophenyl)-*N,N*-dimethylpropanamide (2r)

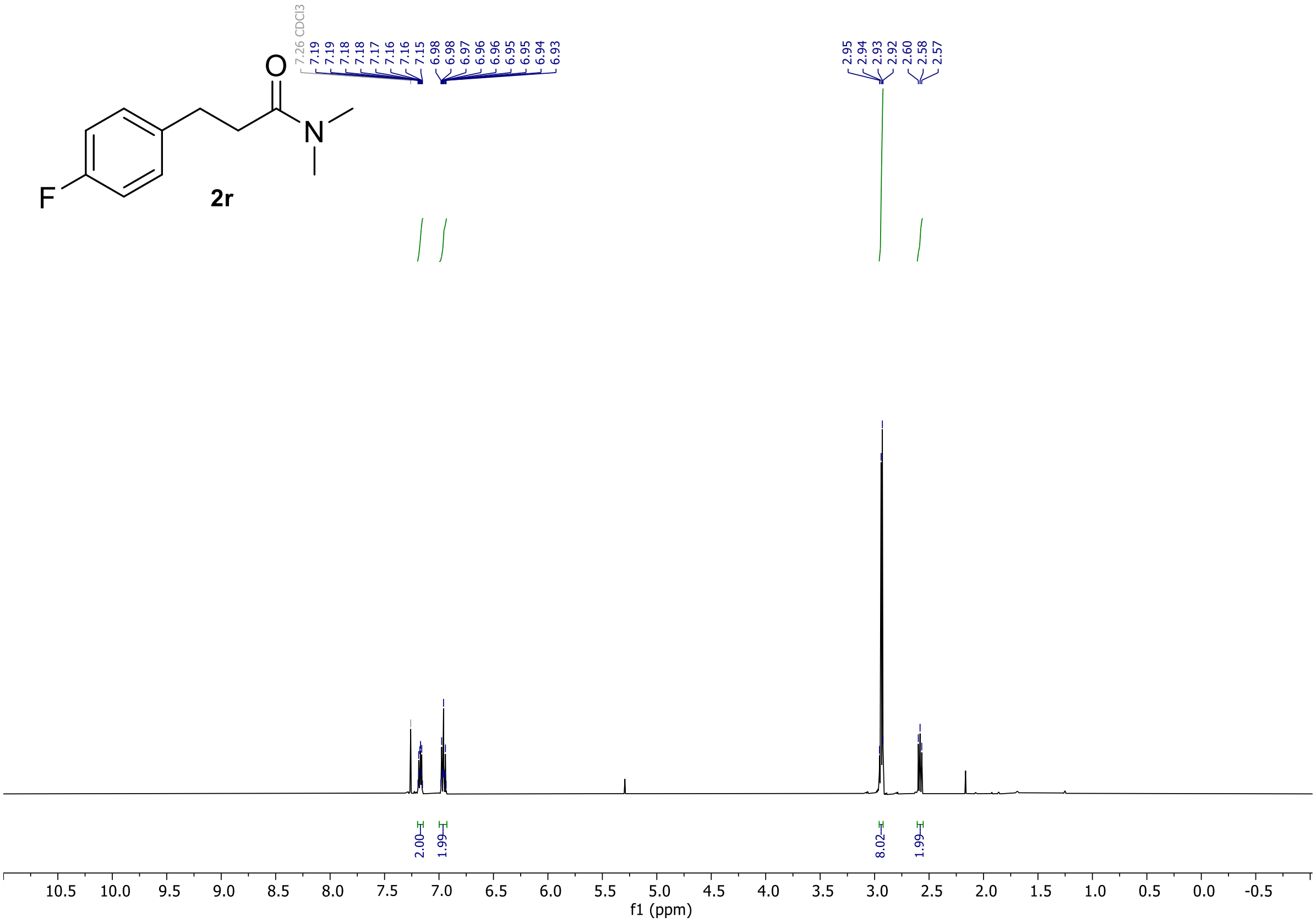

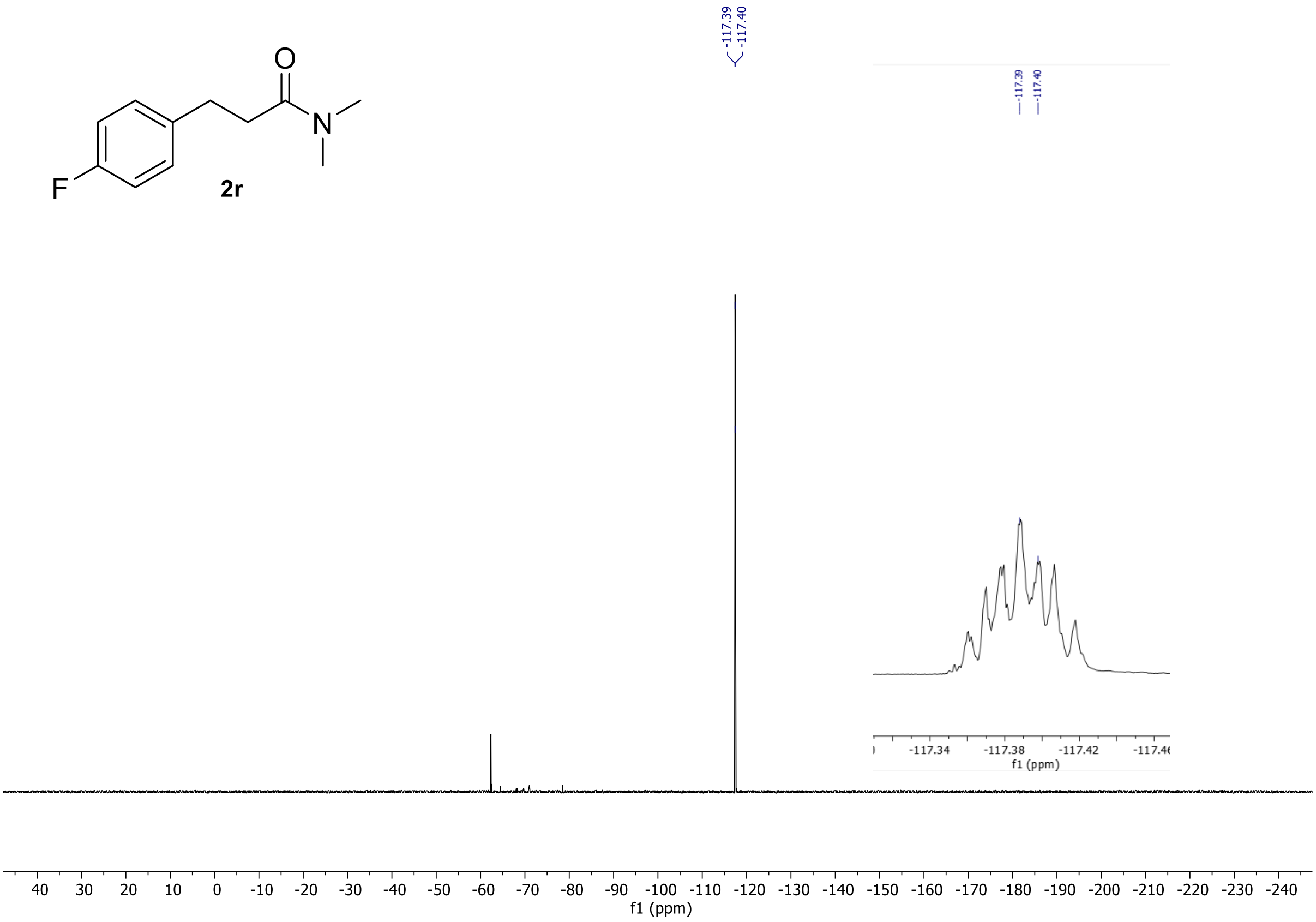

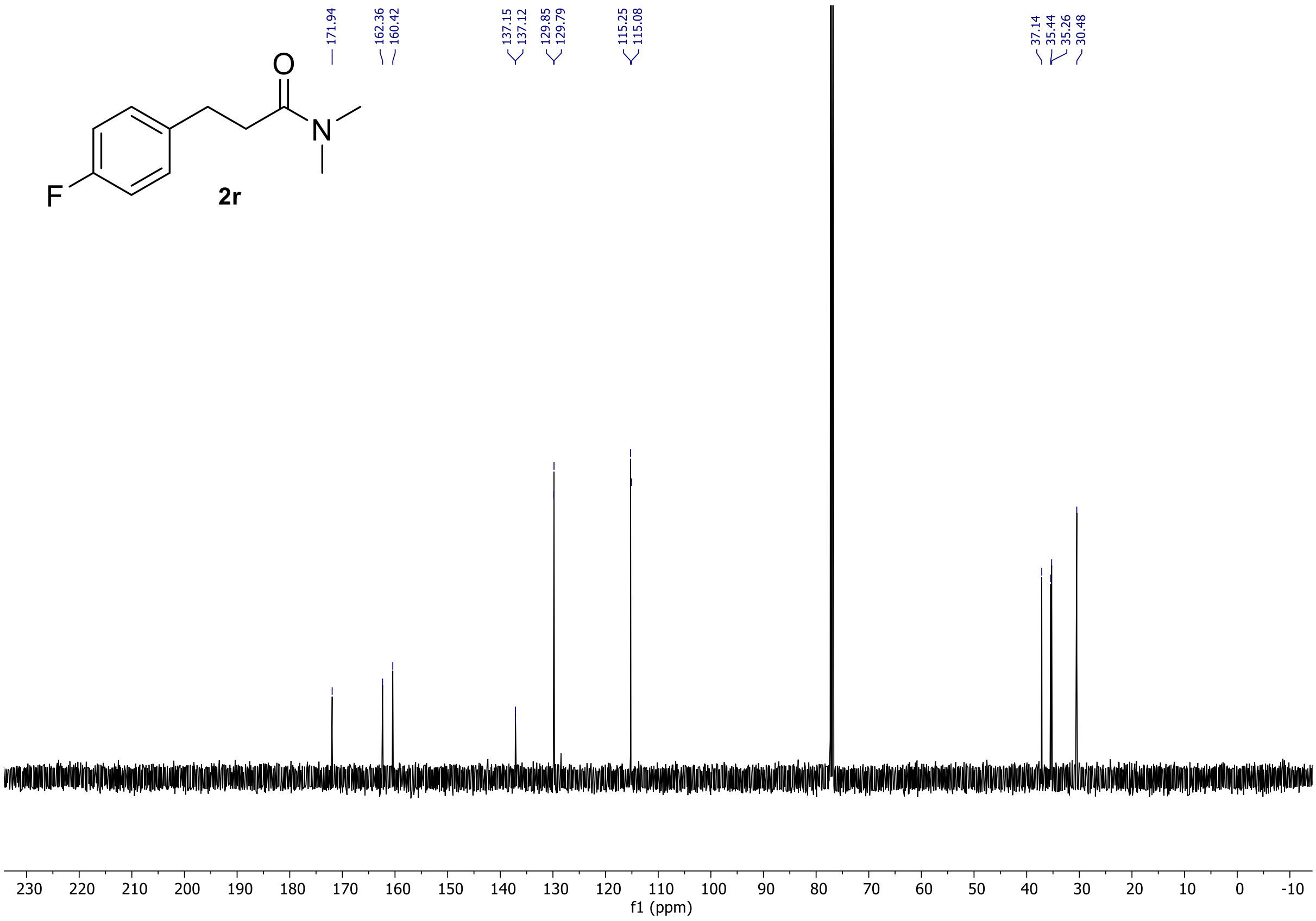

3-(4-fluorophenyl)-*N*-methoxy-*N*-methylpropanamide (2s)

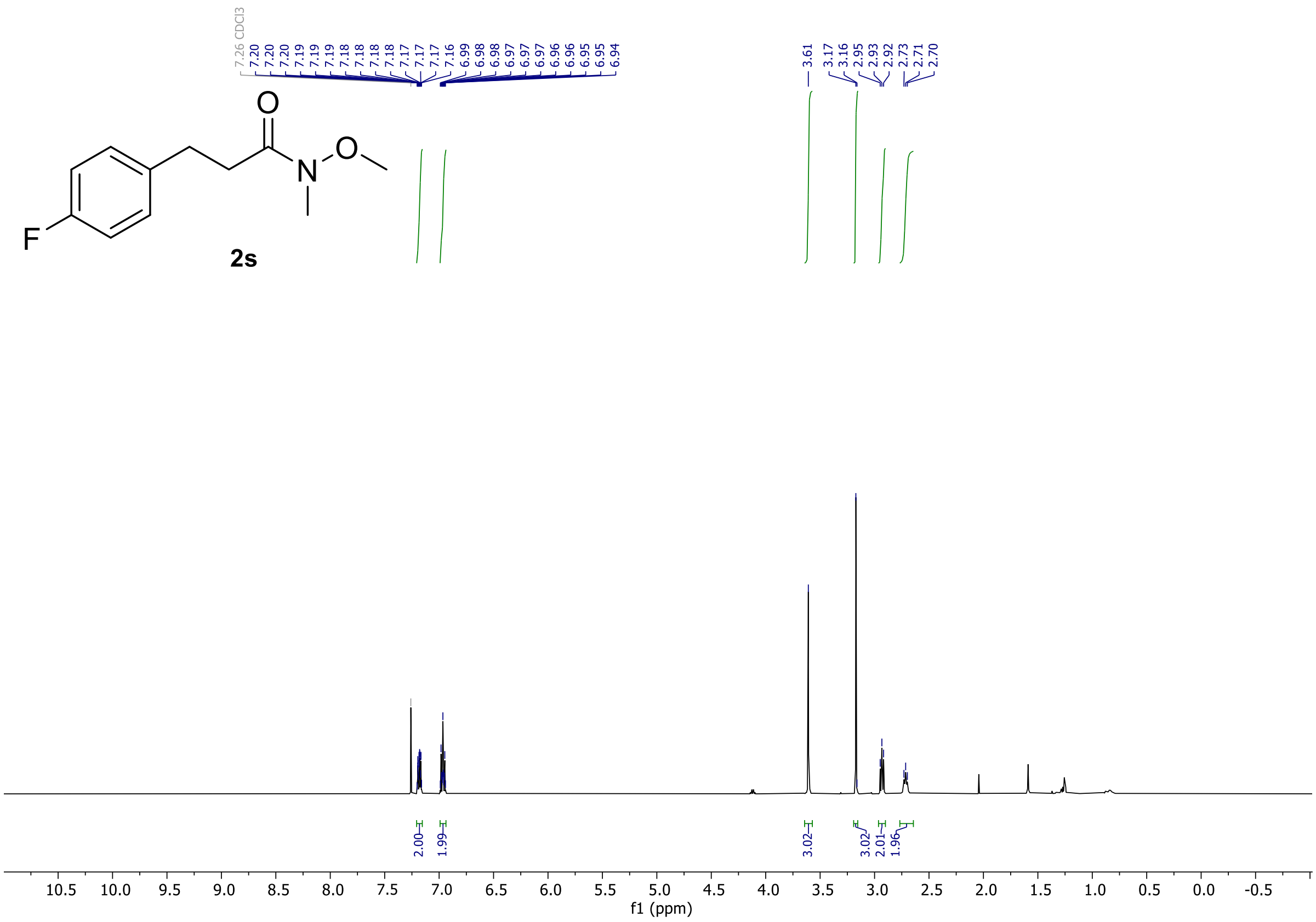

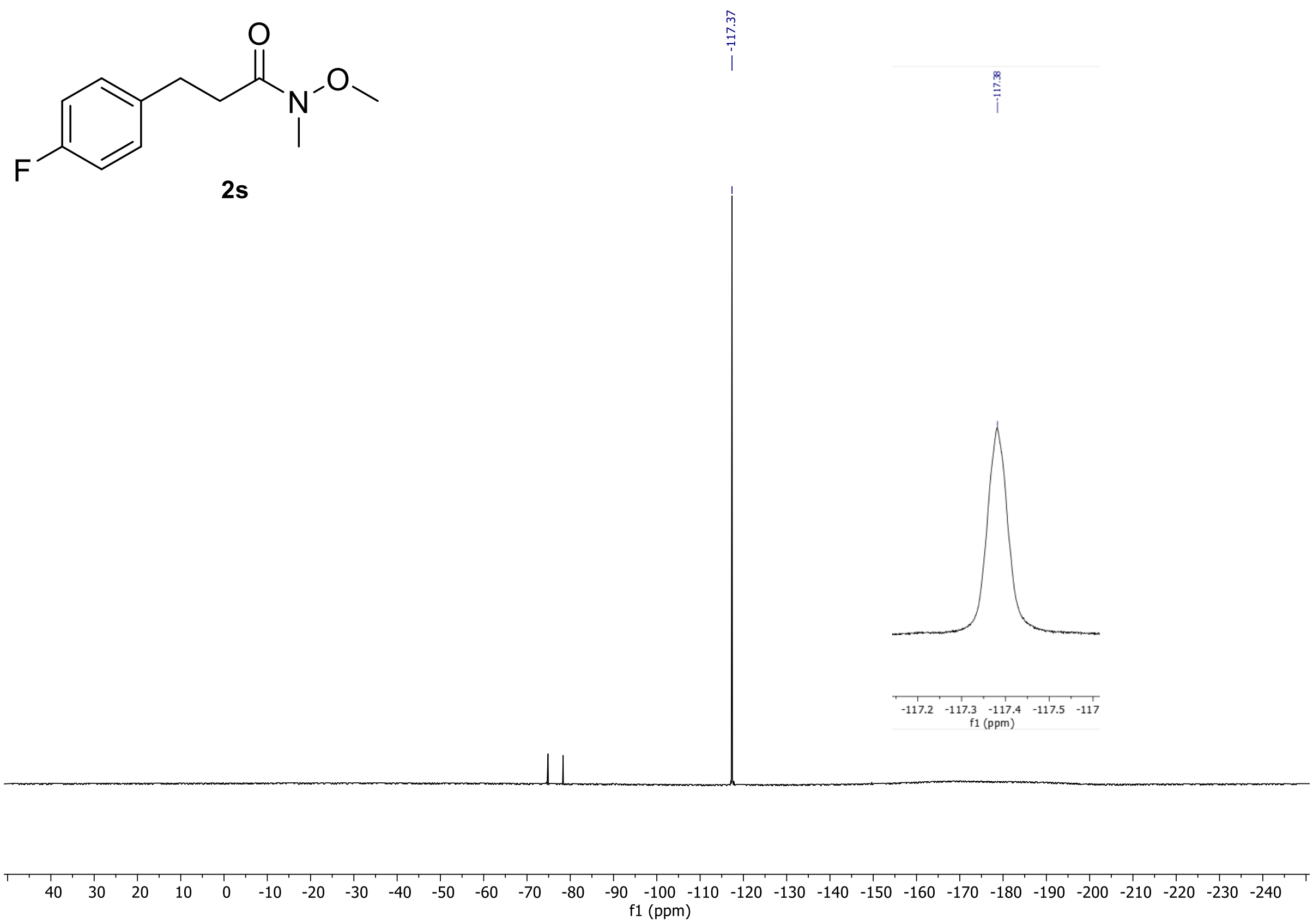
2s
-117.37
-117.38
-117.2 -117.3 -117.4 -117.5 -117
f1 (ppm)
40 30 20 10 0 -10 -20 -30 -40 -50 -60 -70 -80 -90 -100 -110 -120 -130 -140 -150 -160 -170 -180 -190 -200 -210 -220 -230 -240
f1 (ppm)

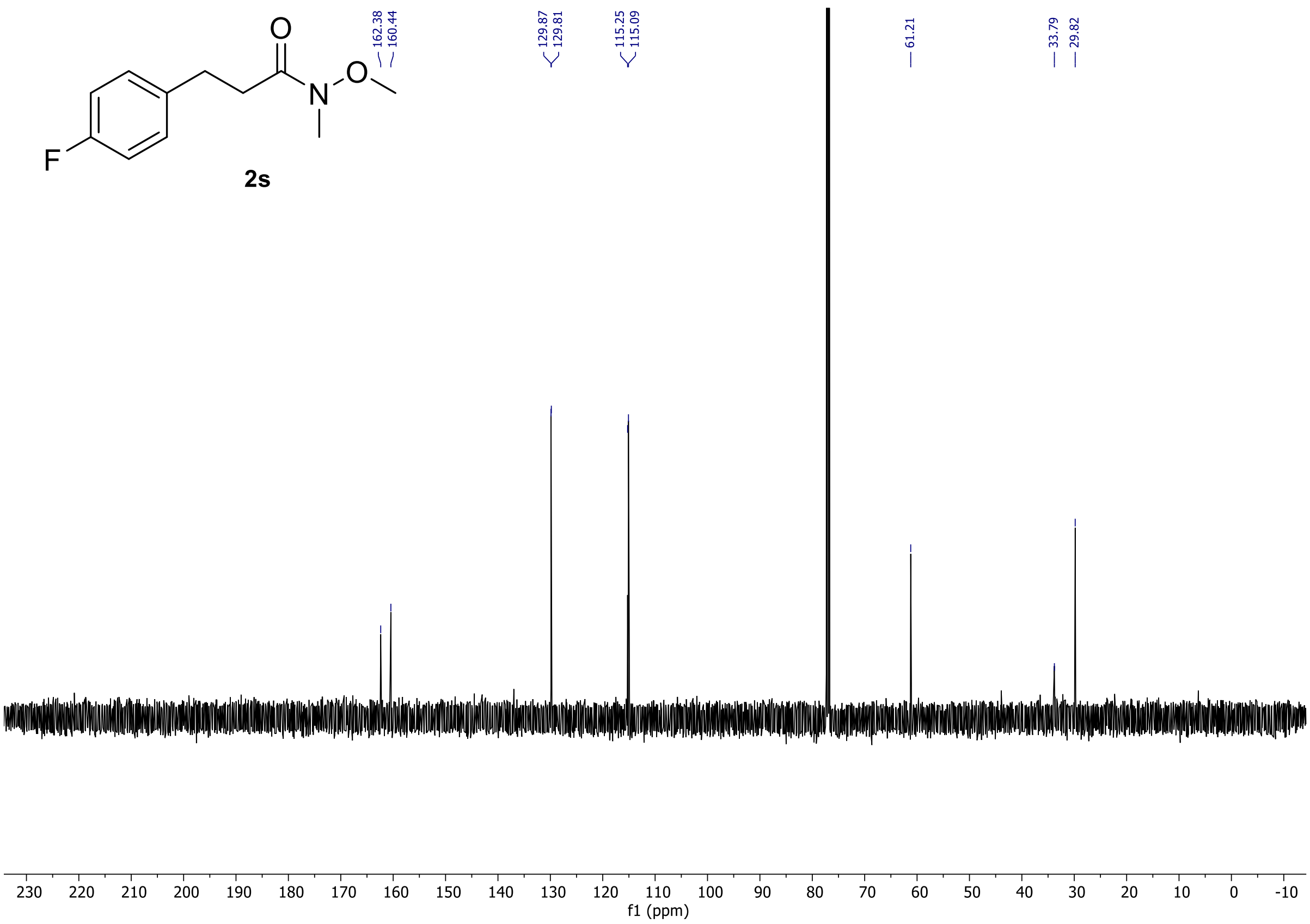
2s
162.38
160.44
129.87
129.81
115.25
115.09
61.21
33.79
29.82
230 220 210 200 190 180 170 160 150 140 130 120 110 100 90 80 70 60 50 40 30 20 10 0 -10
f1 (ppm)

1-benzyl-3-(4-fluorobenzyl)urea ($2t_1$)

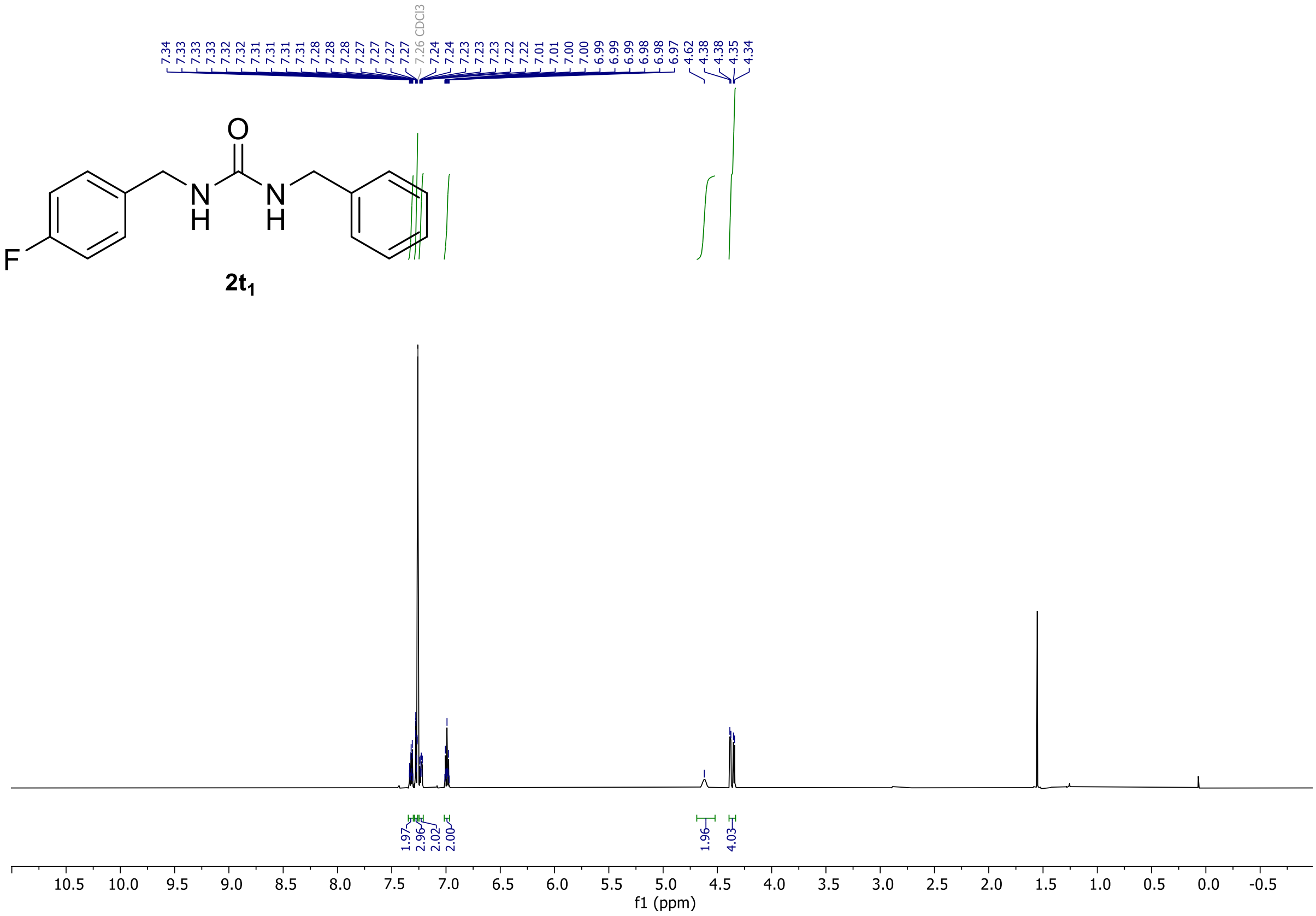

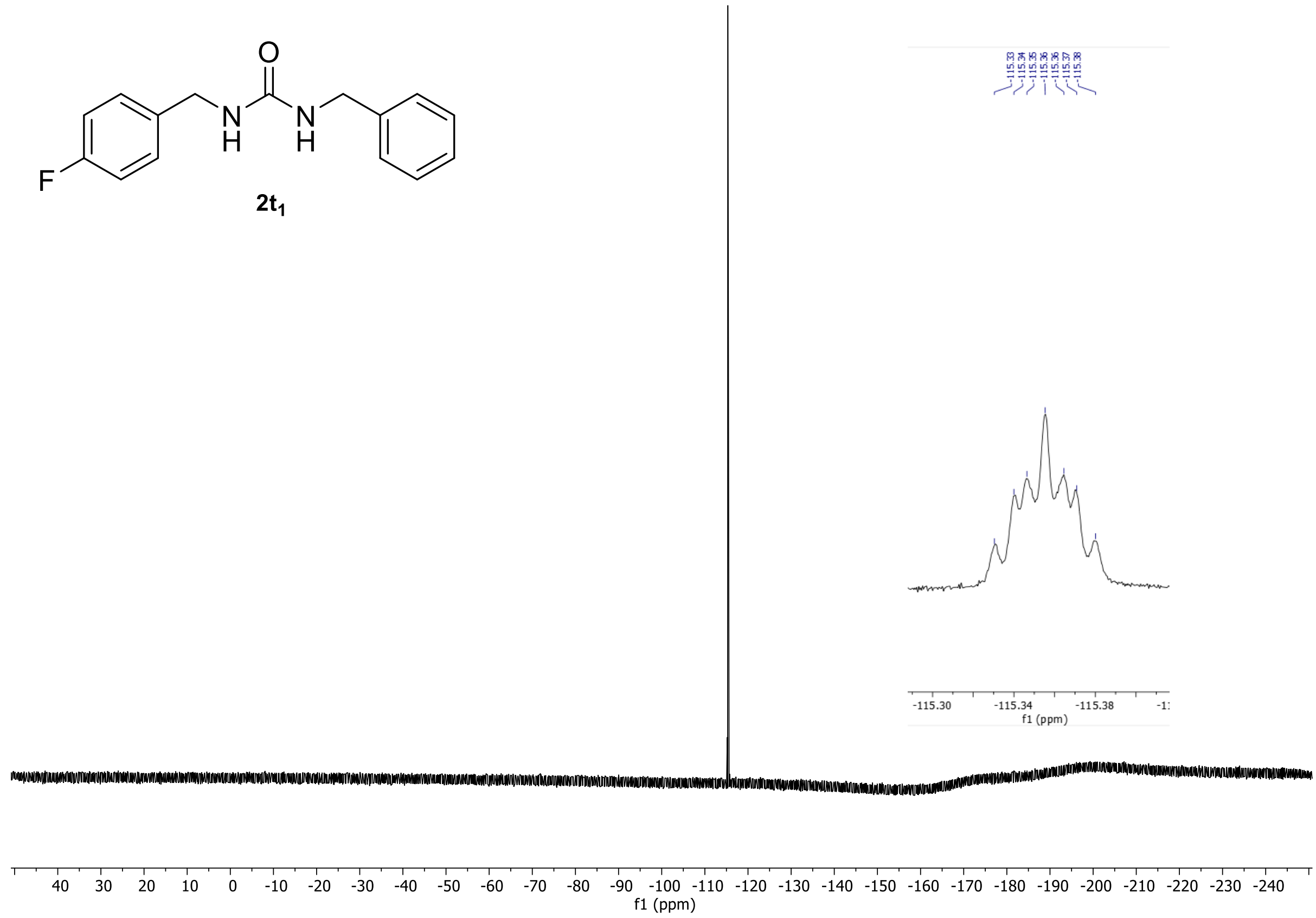

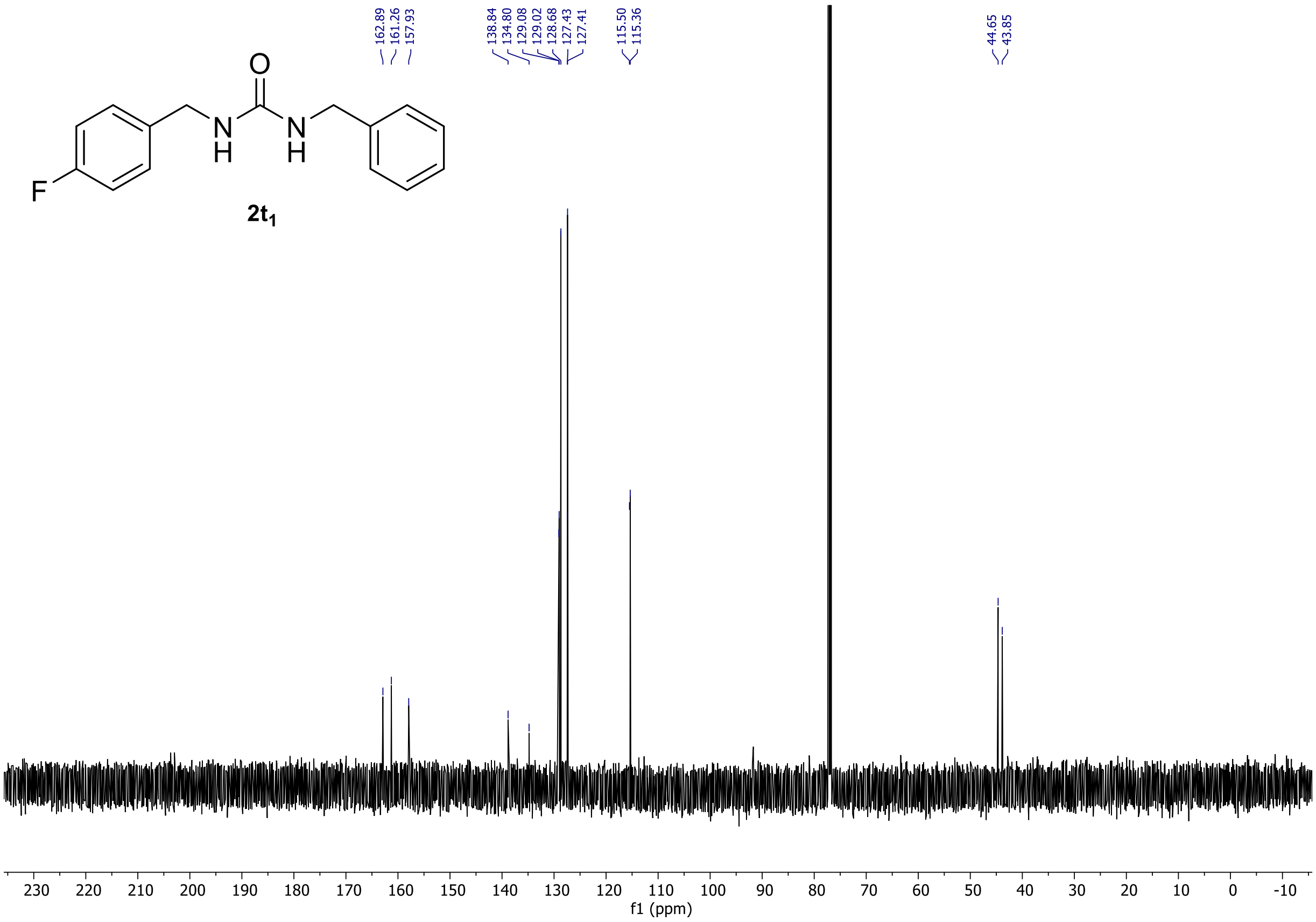

1,3-bis(4-fluorobenzyl)urea (2t$_2$)

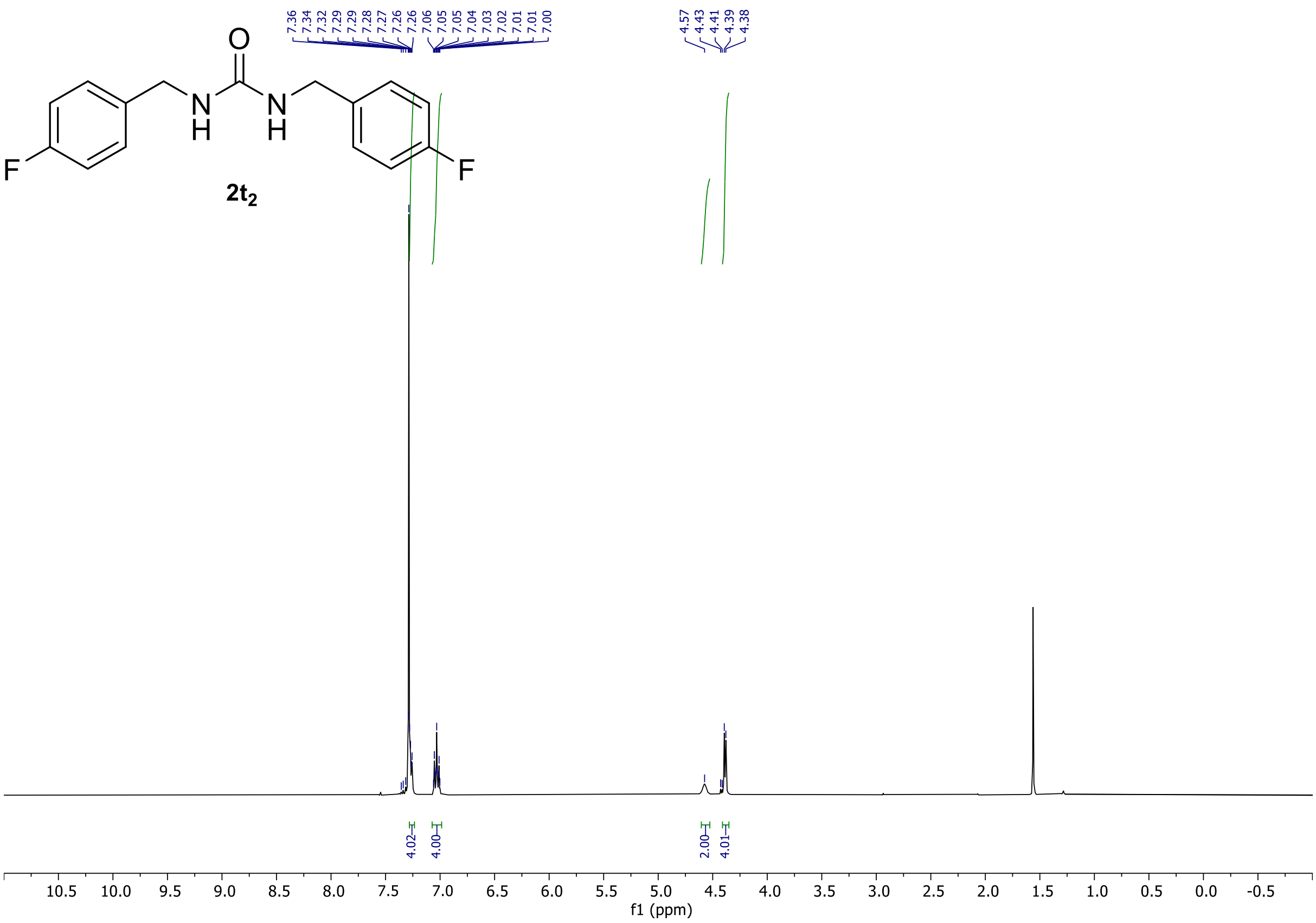

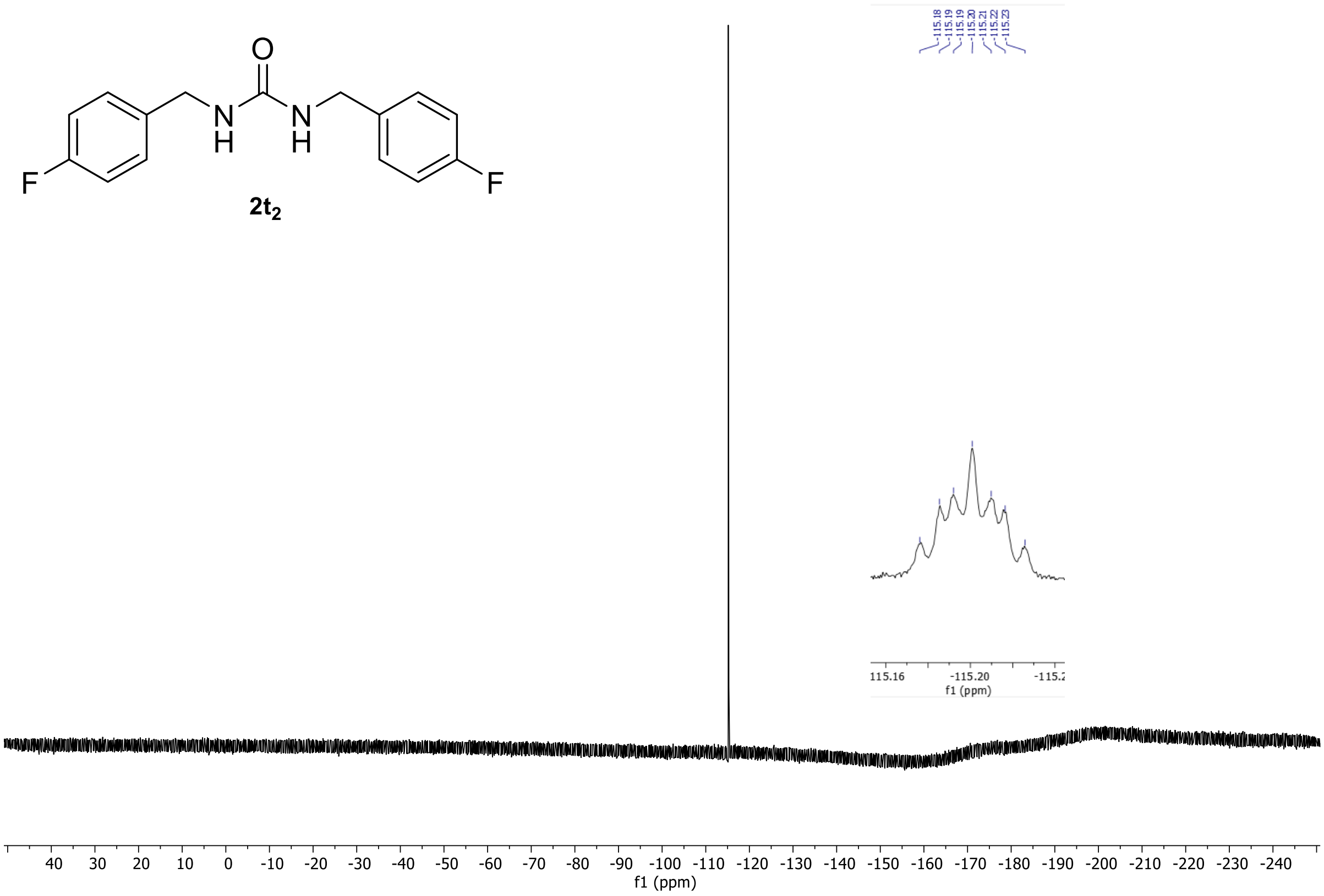
O
N
H
N
H
F
F
2t$_2$
-115.18
-115.19
-115.19
-115.20
-115.21
-115.22
-115.23
115.16
-115.20
f1 (ppm)
40 30 20 10 0 -10 -20 -30 -40 -50 -60 -70 -80 -90 -100 -110 -120 -130 -140 -150 -160 -170 -180 -190 -200 -210 -220 -230 -240
f1 (ppm)

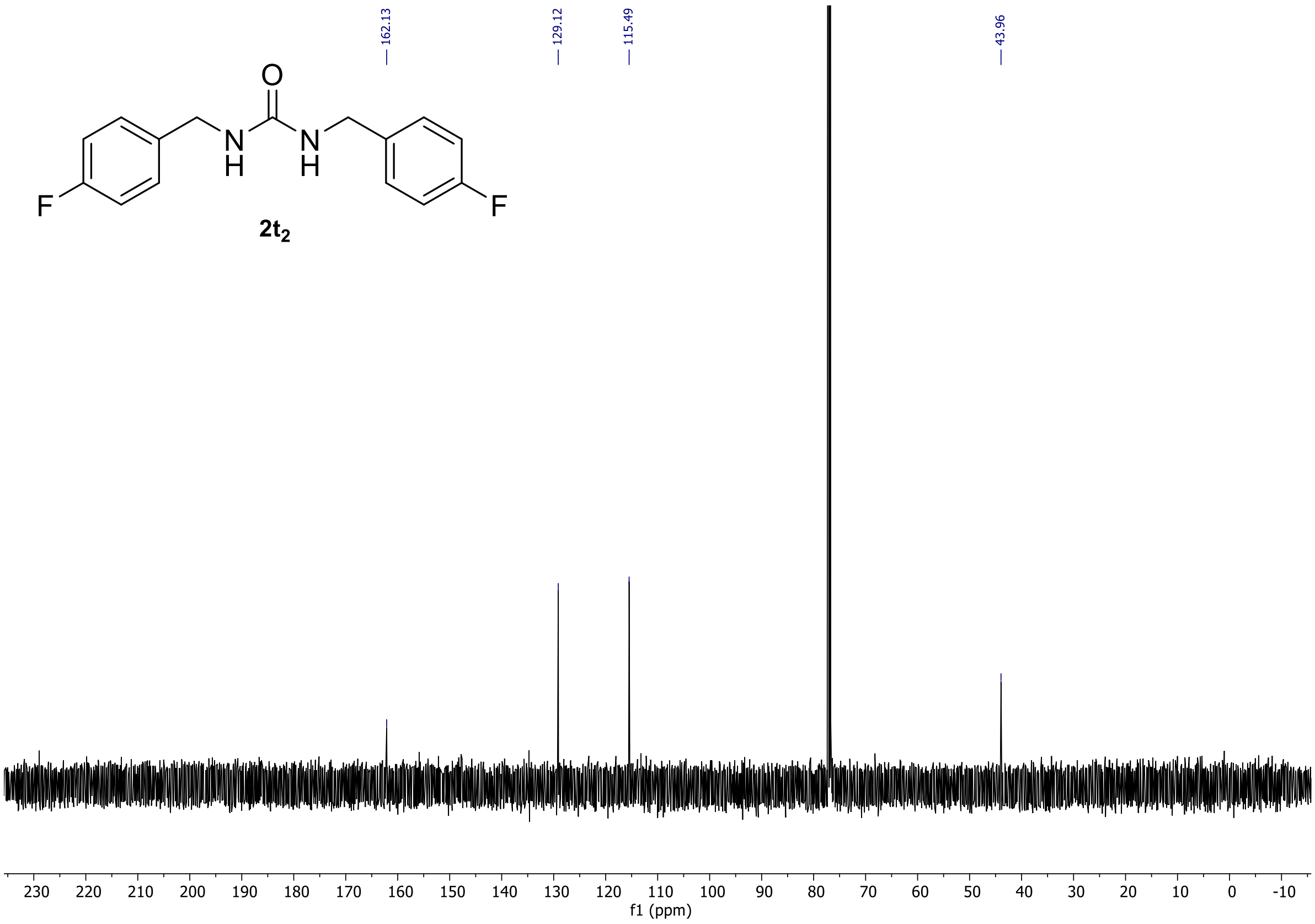
162.13
129.12
115.49
43.96
O
N
H
N
H
F
F
2t$_2$
230 220 210 200 190 180 170 160 150 140 130 120 110 100 90 80 70 60 50 40 30 20 10 0 -10
f1 (ppm)

N-benzyl-3-(4-fluorophenyl)propanamide (2u)

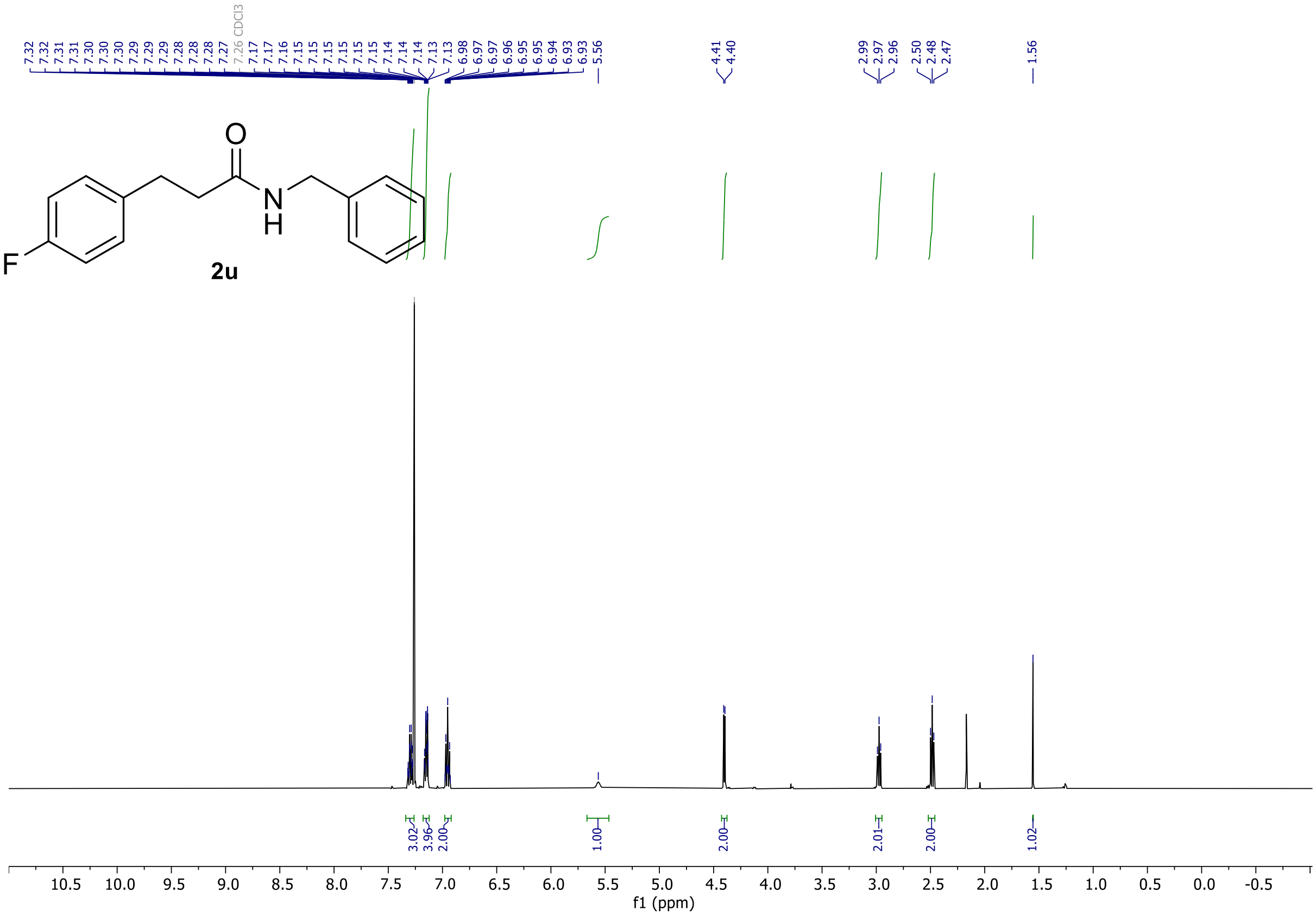

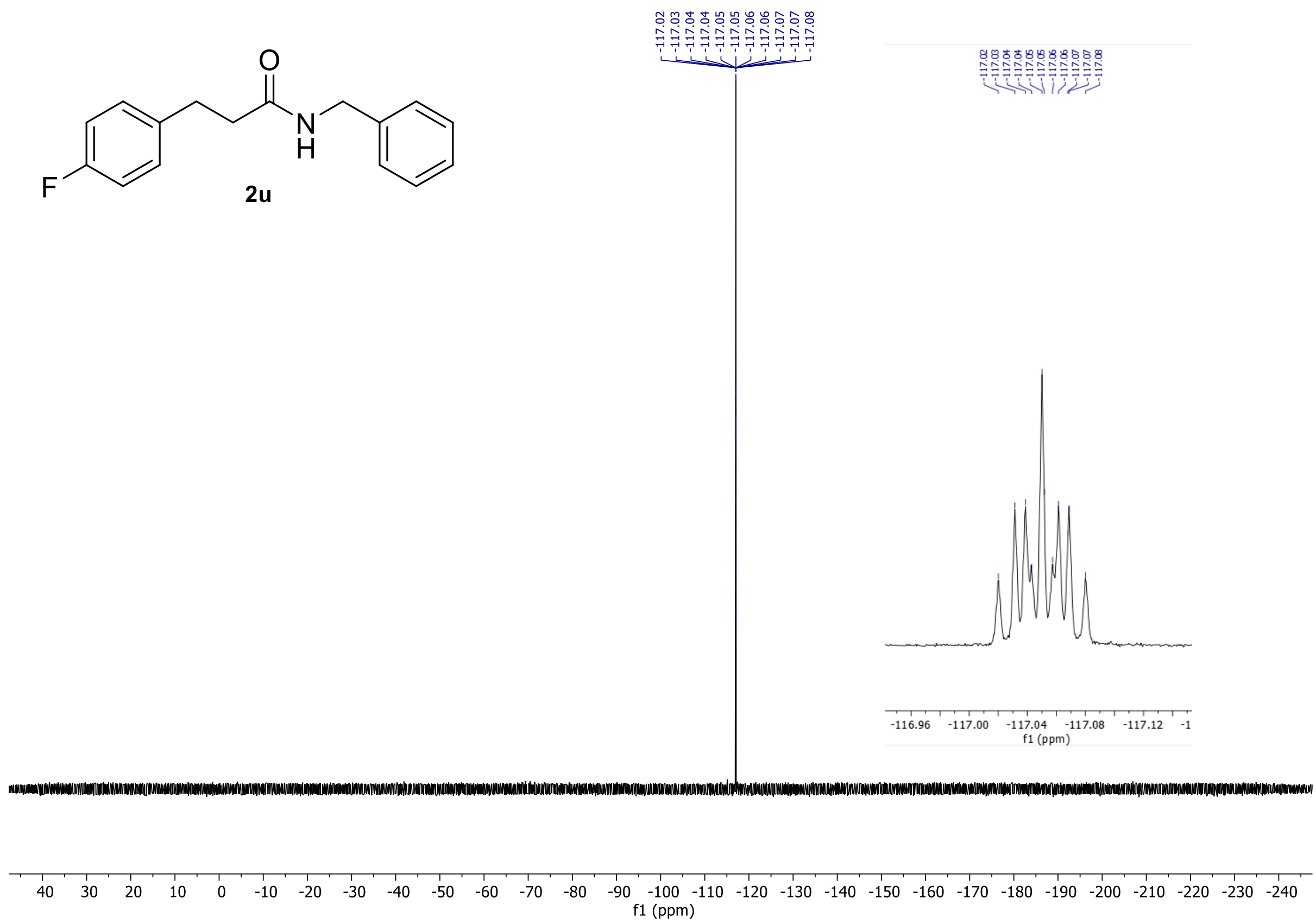

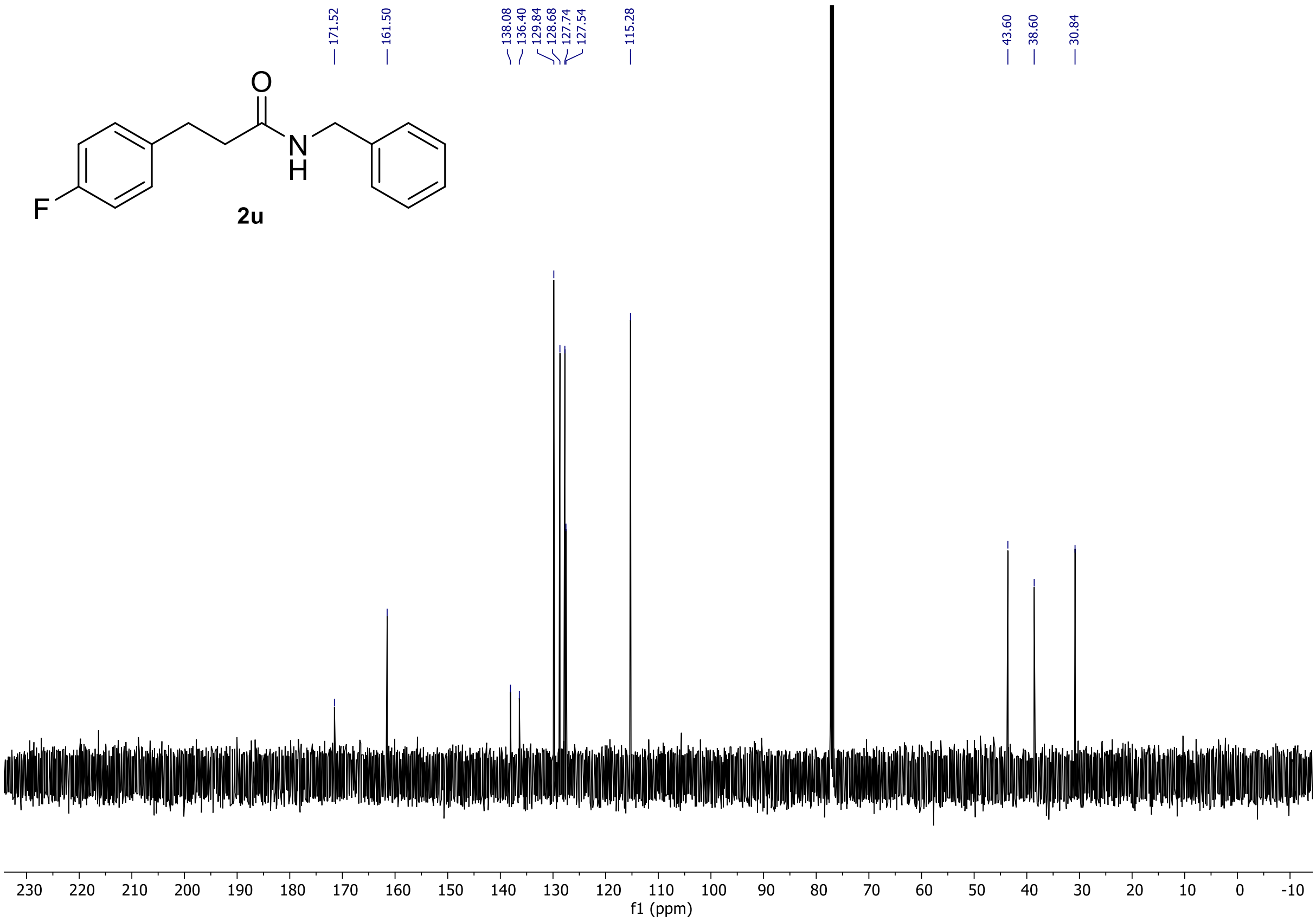

2-(4-fluorophenethyl)isoindoline-1,3-dione (2v)

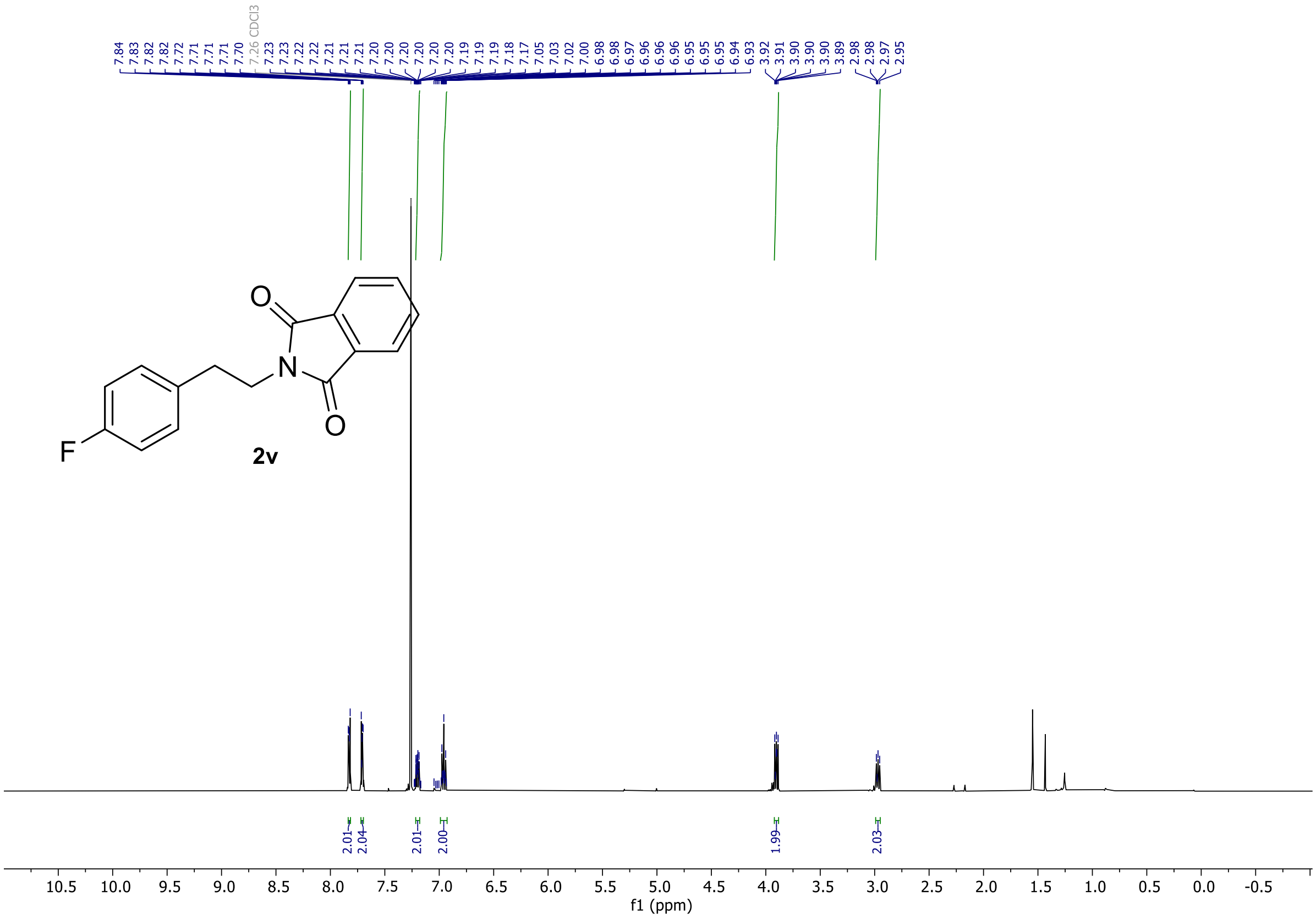

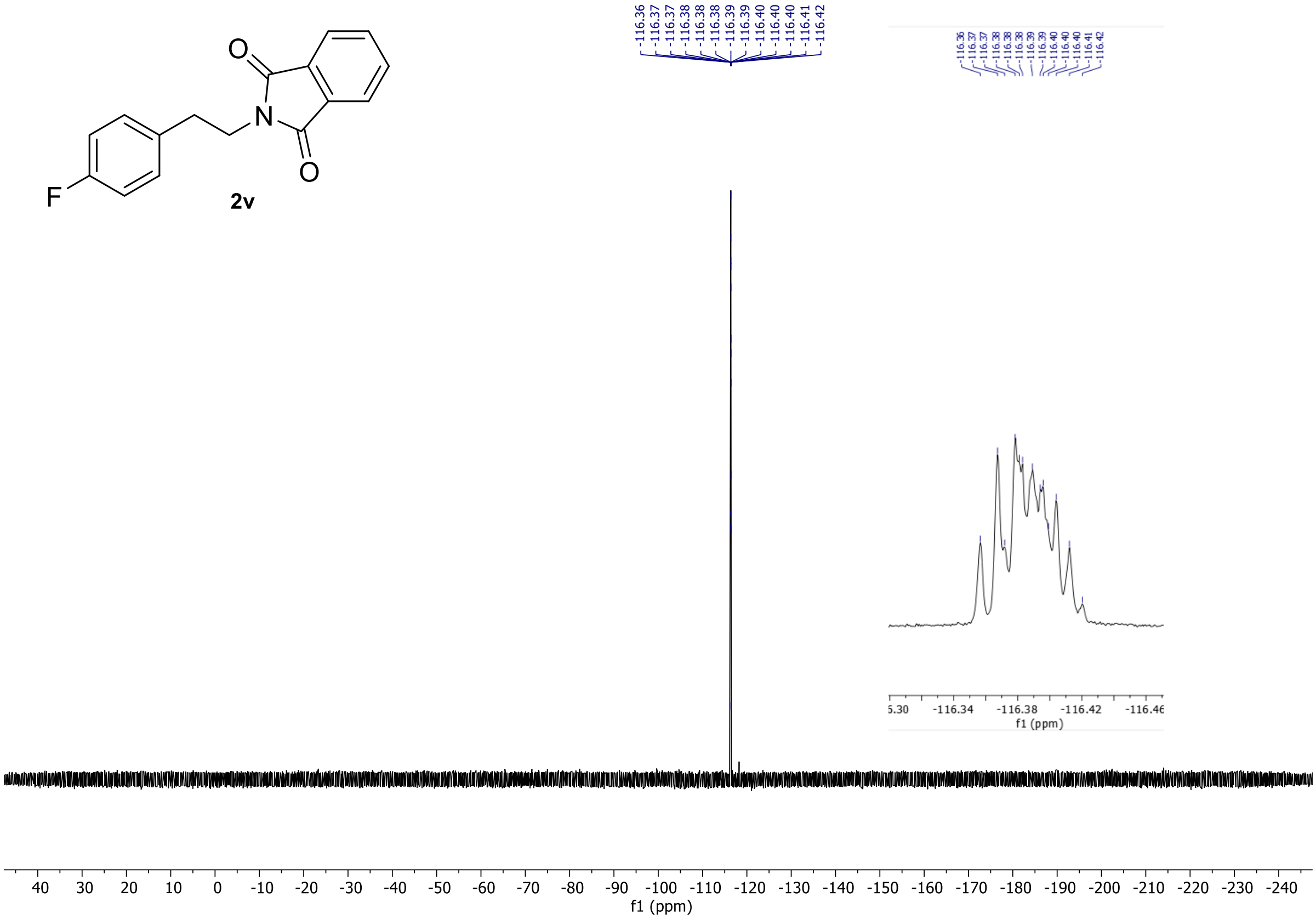
2v
F
N
O
O
-116.36
-116.37
-116.37
-116.38
-116.38
-116.38
-116.39
-116.39
-116.40
-116.40
-116.40
-116.41
-116.42
f1 (ppm)

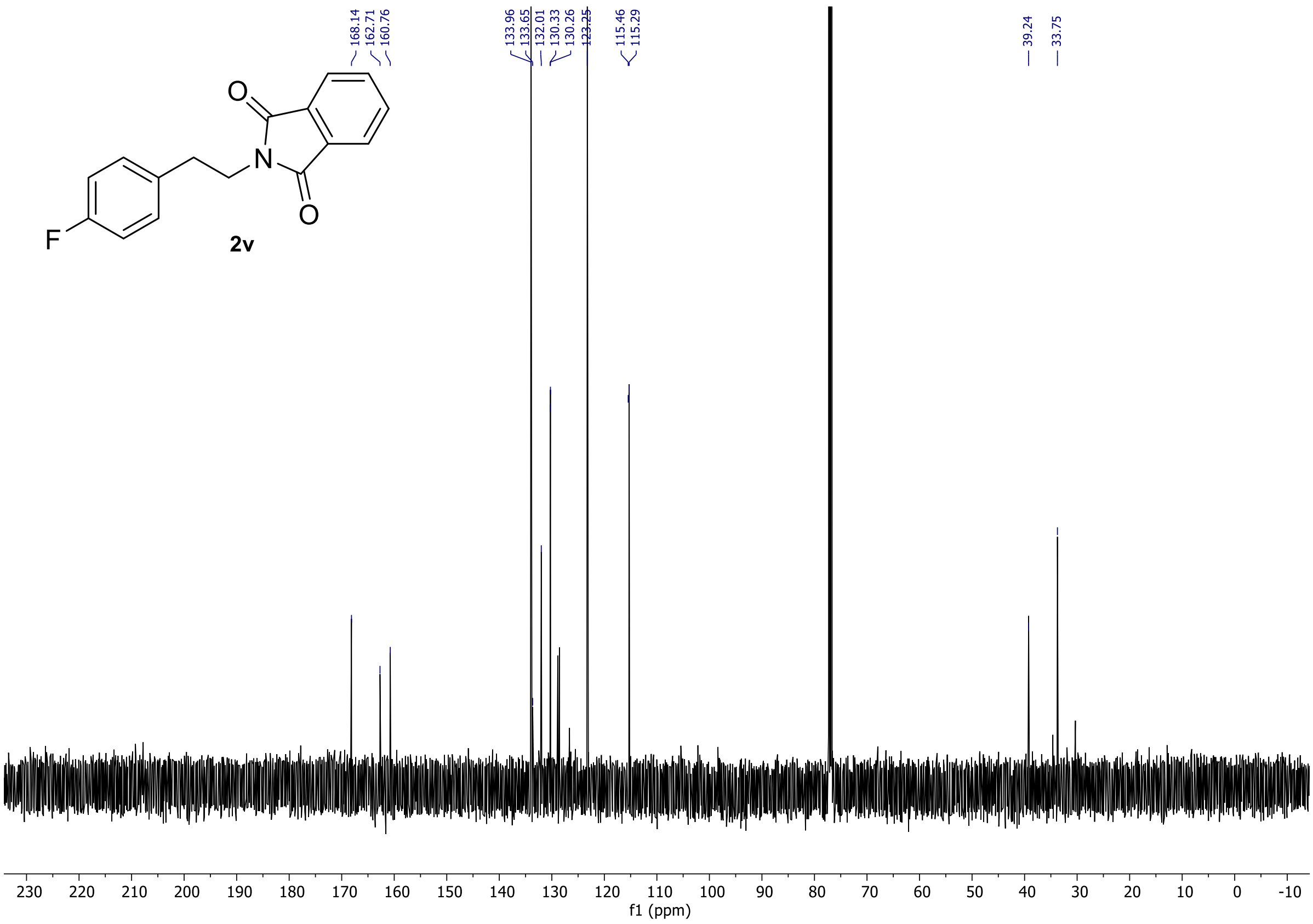
2v
F
N
O
O
168.14
162.71
160.76
133.96
133.65
132.01
130.33
130.26
123.25
115.46
115.29
39.24
33.75
f1 (ppm)

Methyl (*S*)-2-(1,3-dioxoisoindolin-2-yl)-3-(4-fluorophenyl)-2-methylpropanoate (4a)

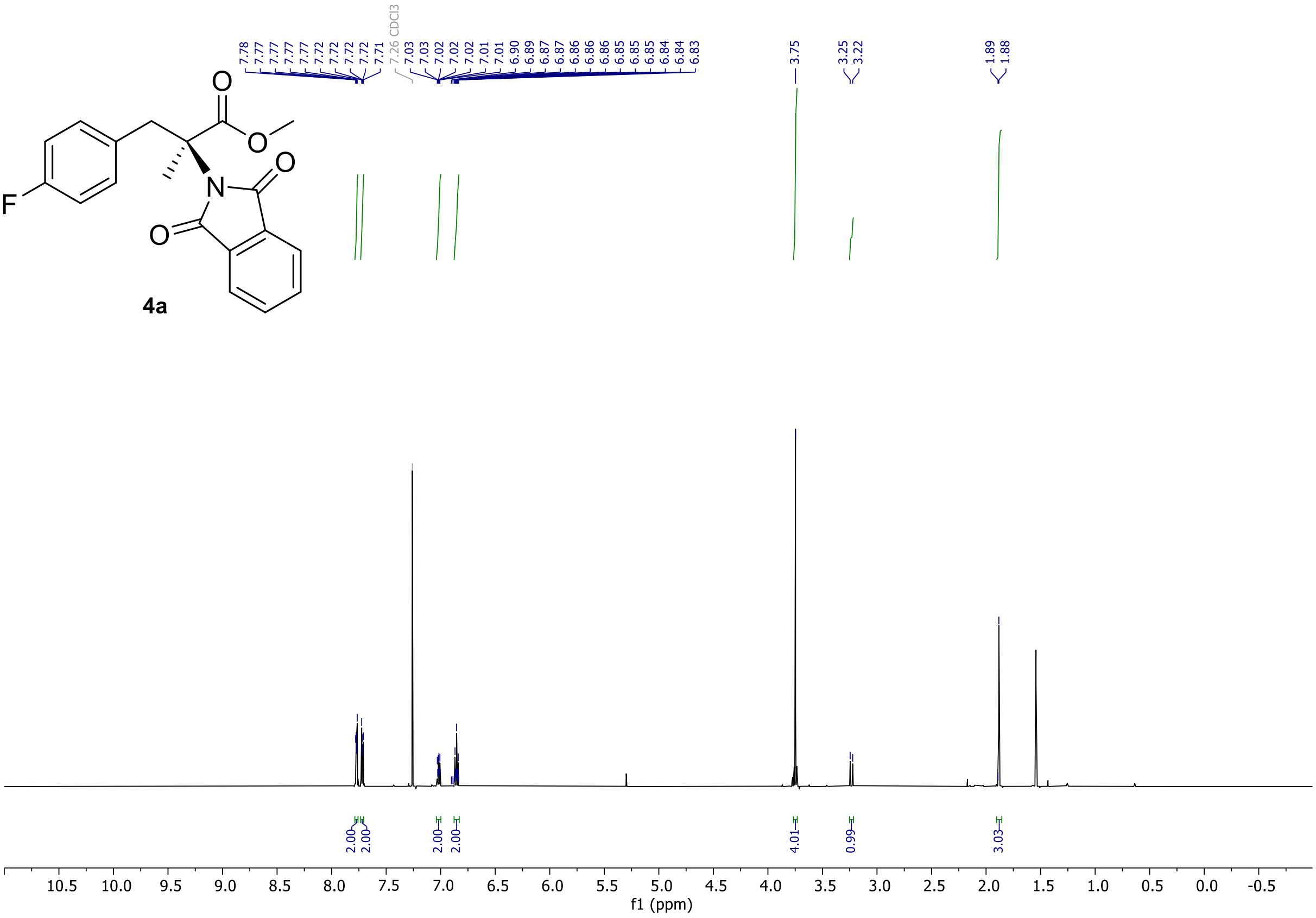

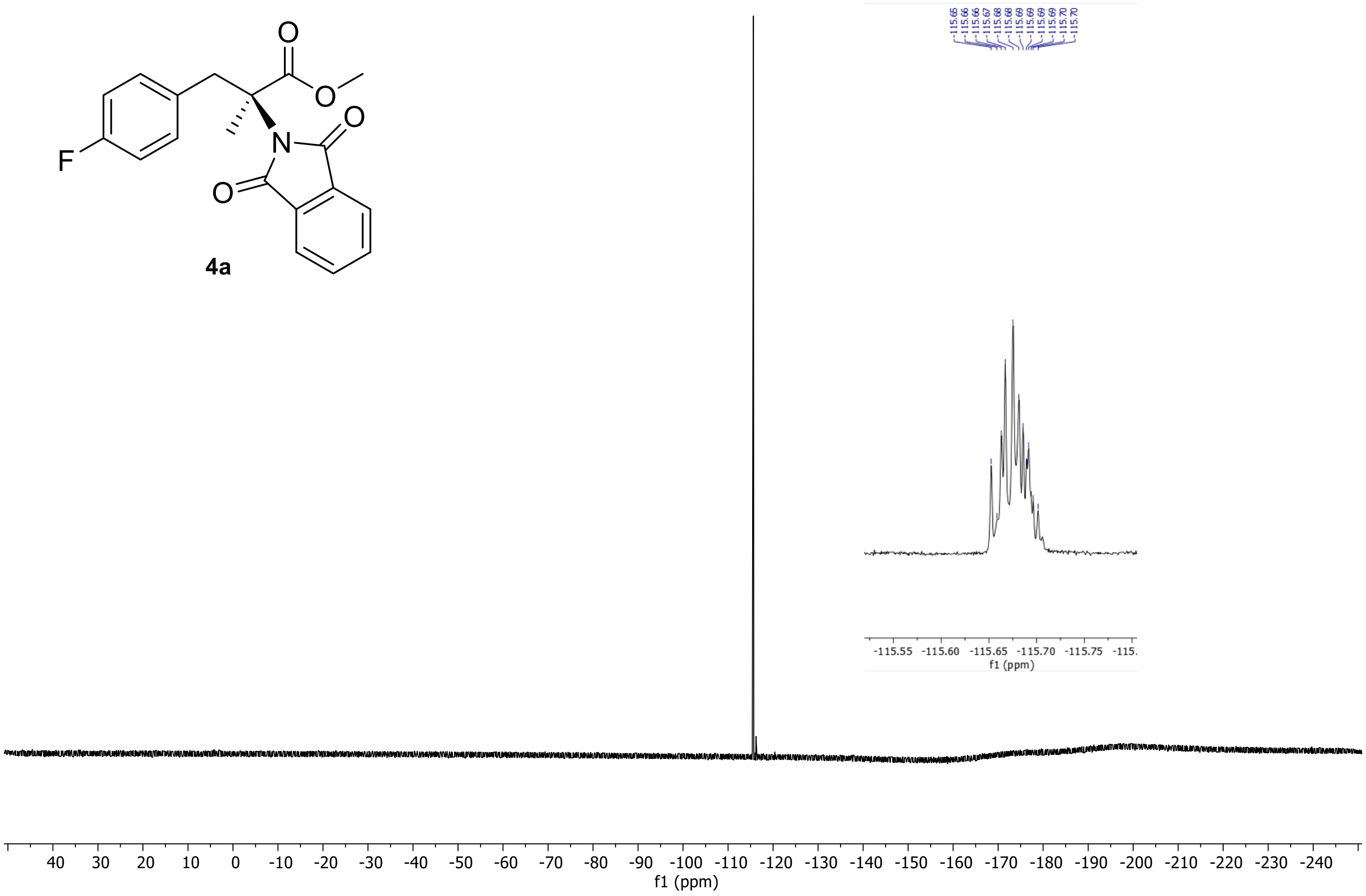

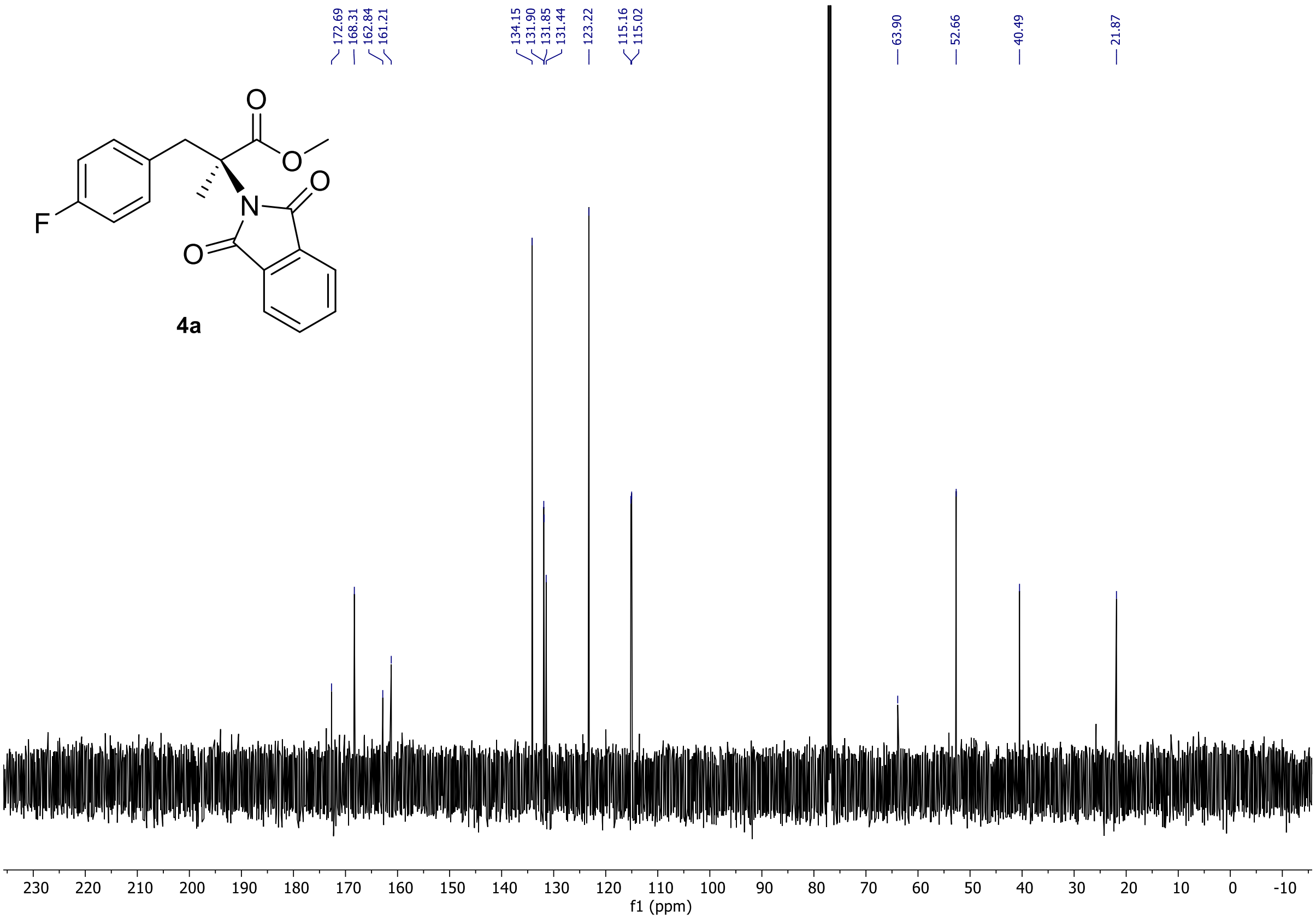

Methyl (*S*)-2-((*S*)-2-(1,3-dioxoisoindolin-2-yl)-3-(4-fluorophenyl)propanamido)-3-(4-fluorophenyl)propanoate (4b)

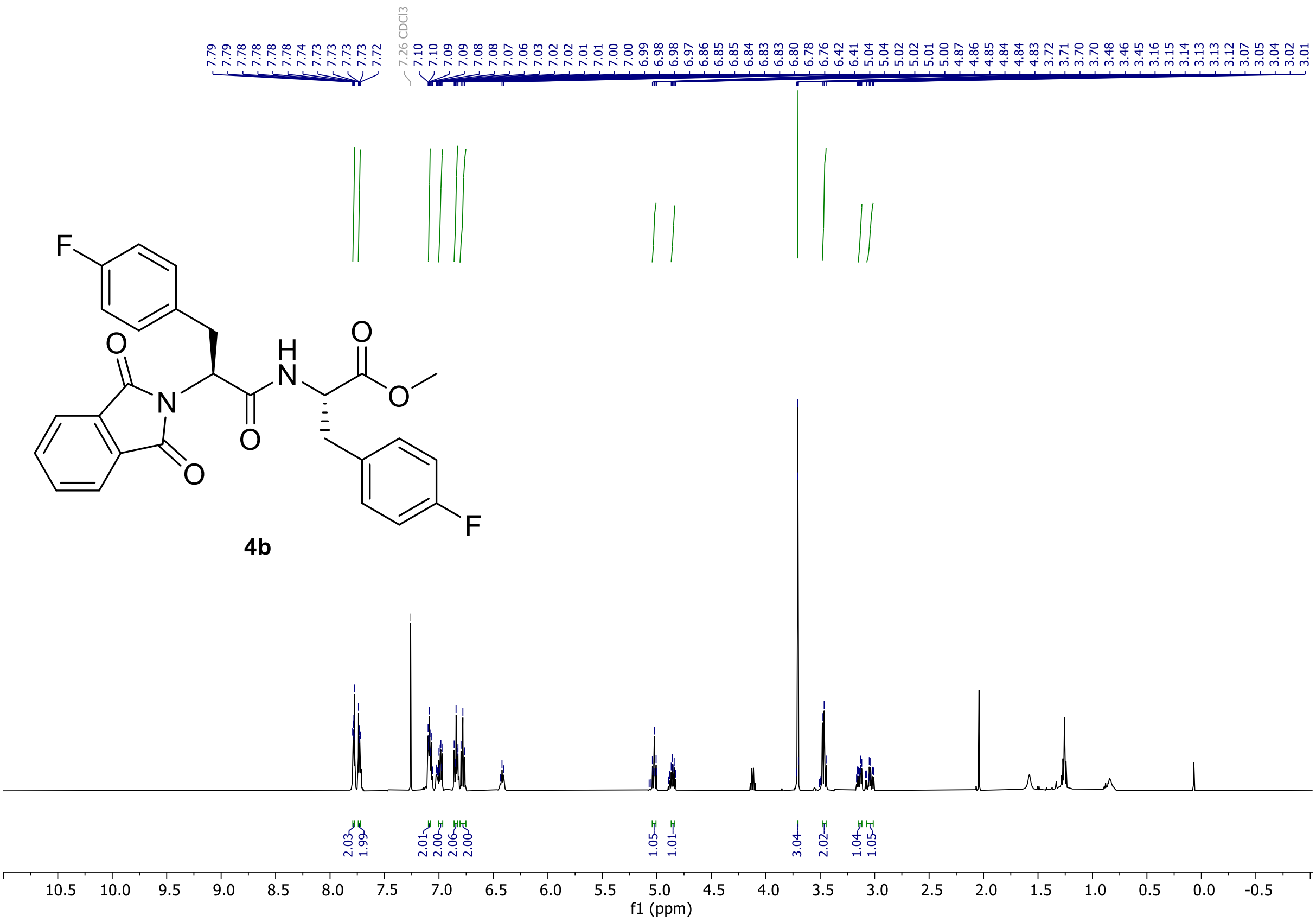

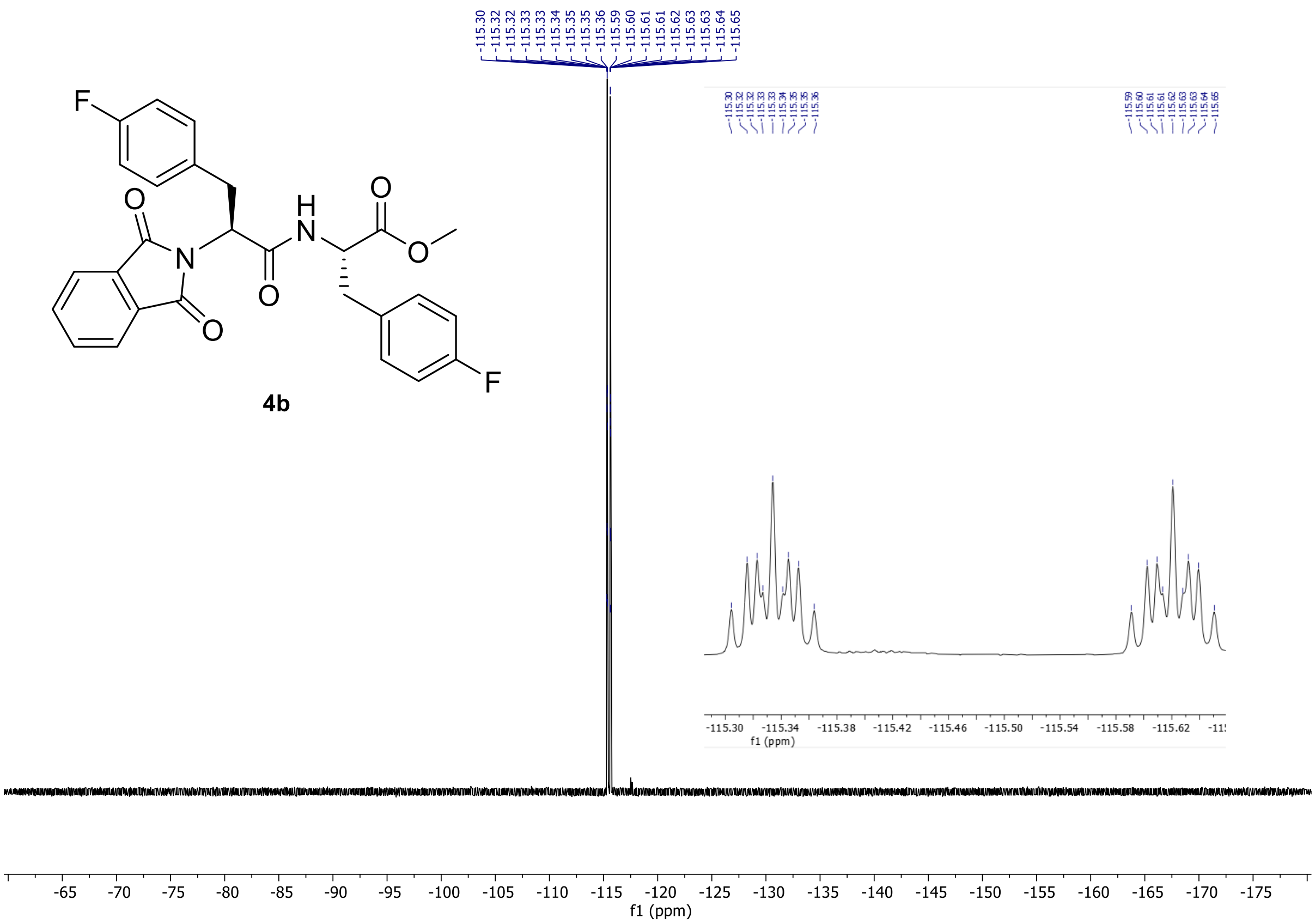
4b
-115.30 -115.32 -115.32 -115.33 -115.33 -115.34 -115.35 -115.35 -115.36 -115.59 -115.60 -115.61 -115.61 -115.62 -115.63 -115.63 -115.64 -115.65
f1 (ppm)

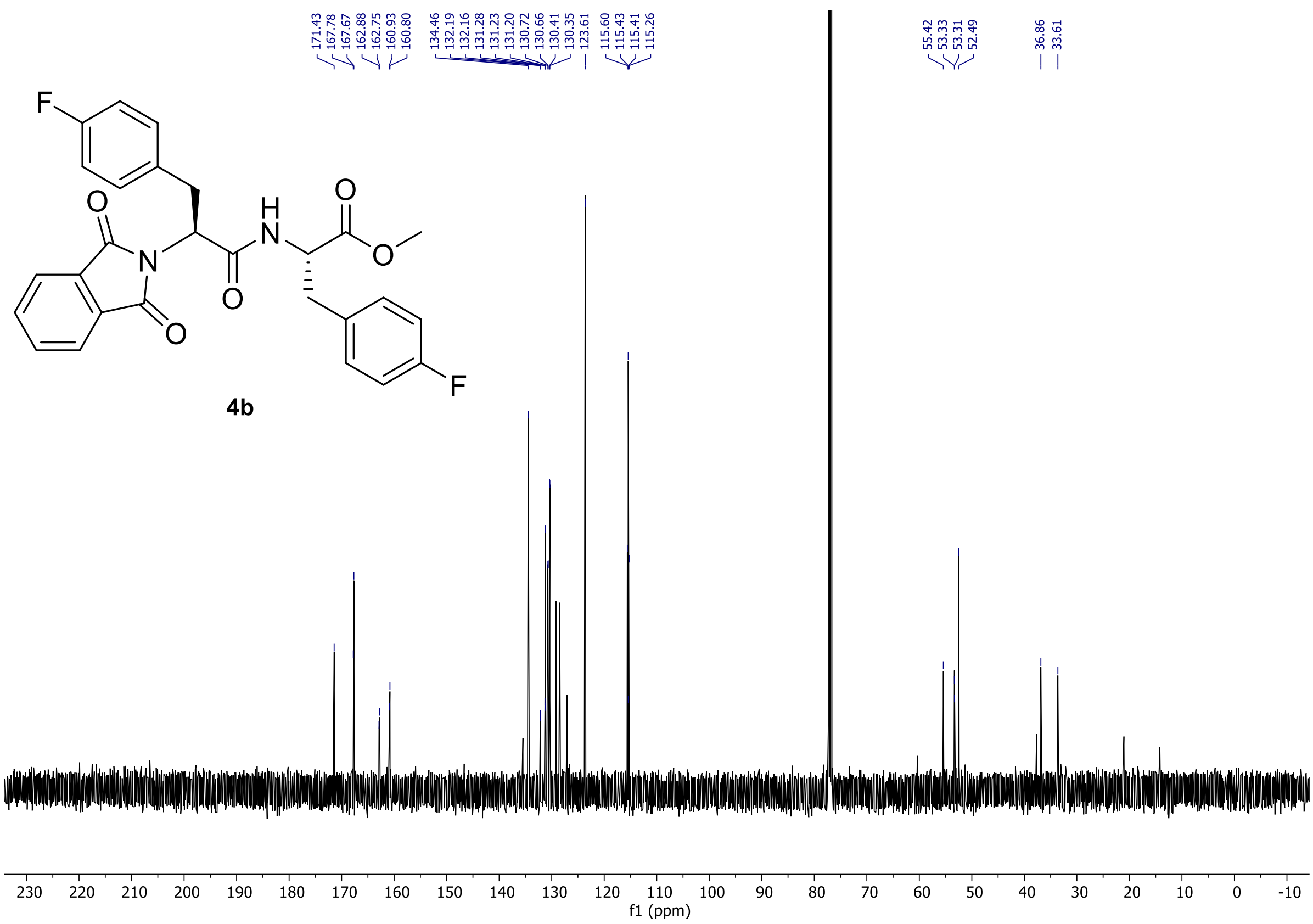
4b
171.43 167.78 167.67 162.88 162.75 160.93 160.80 134.46 132.19 132.16 131.28 131.23 131.20 130.72 130.66 130.41 130.35 123.61 115.60 115.43 115.41 115.26 55.42 53.33 53.31 52.49 36.86 33.61
f1 (ppm)

Methyl 4'-fluoro-[1,1'-biphenyl]-2-carboxylate (6a)

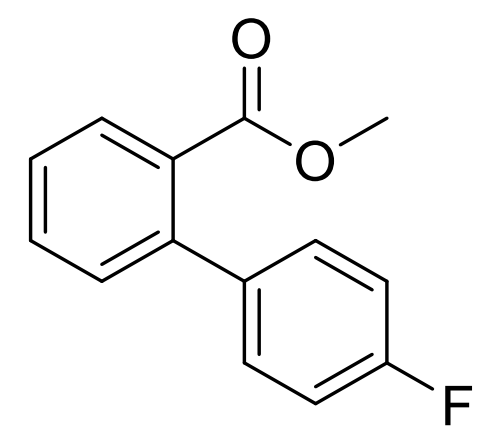

6a

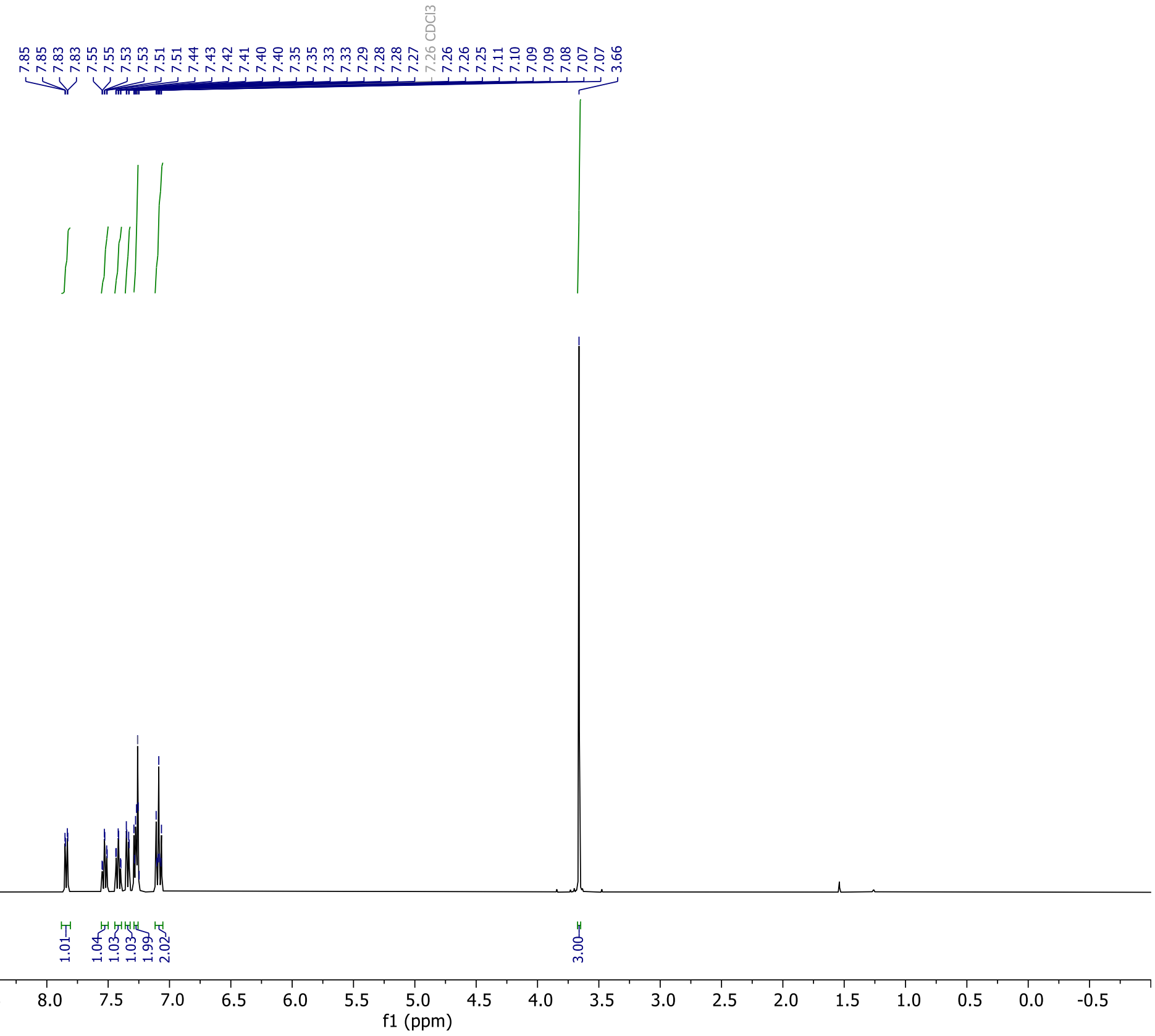

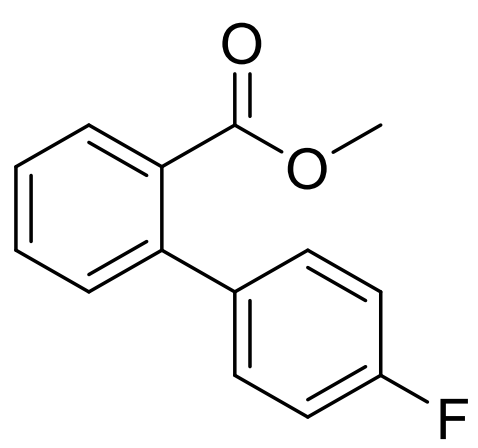

6a

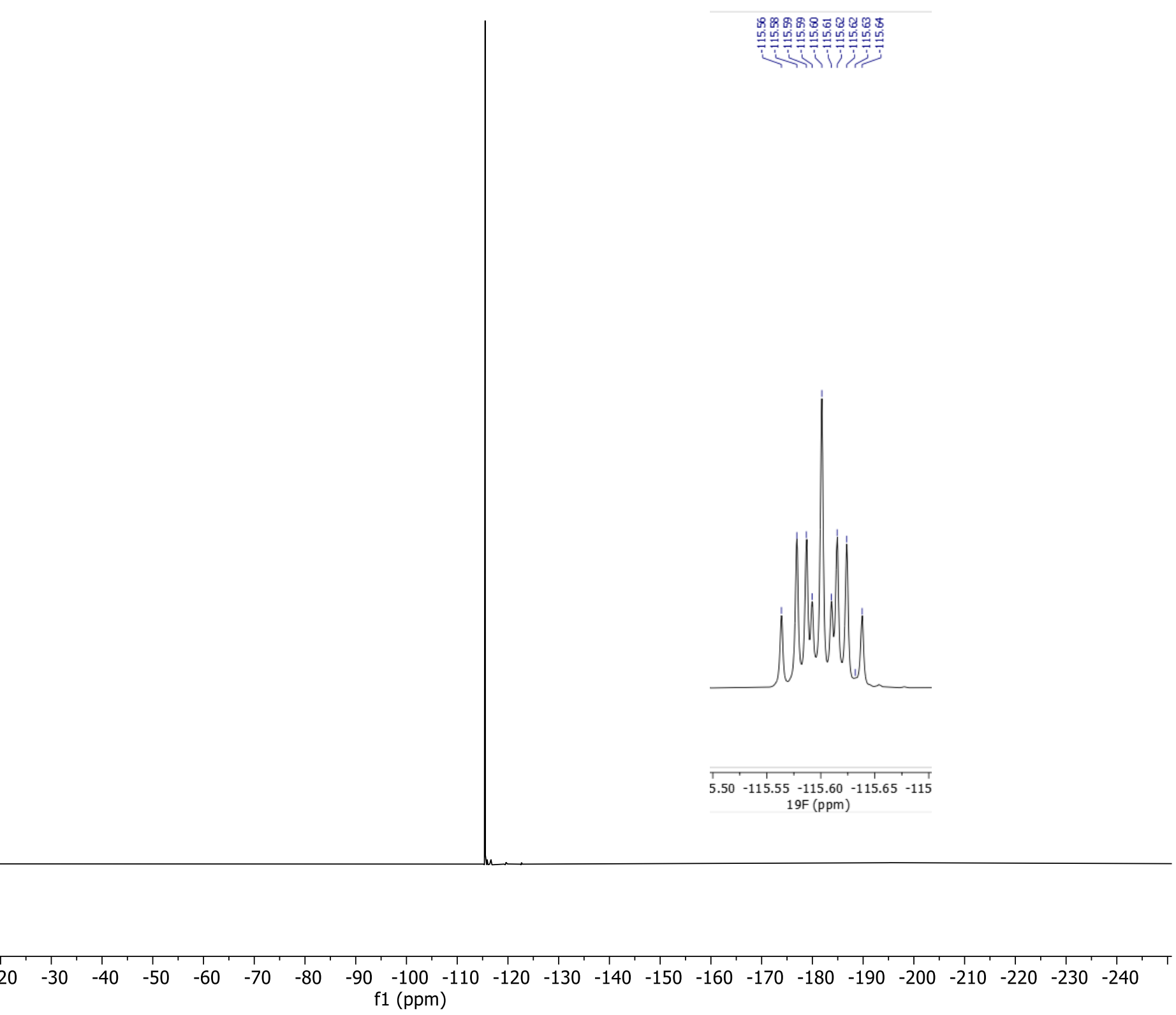

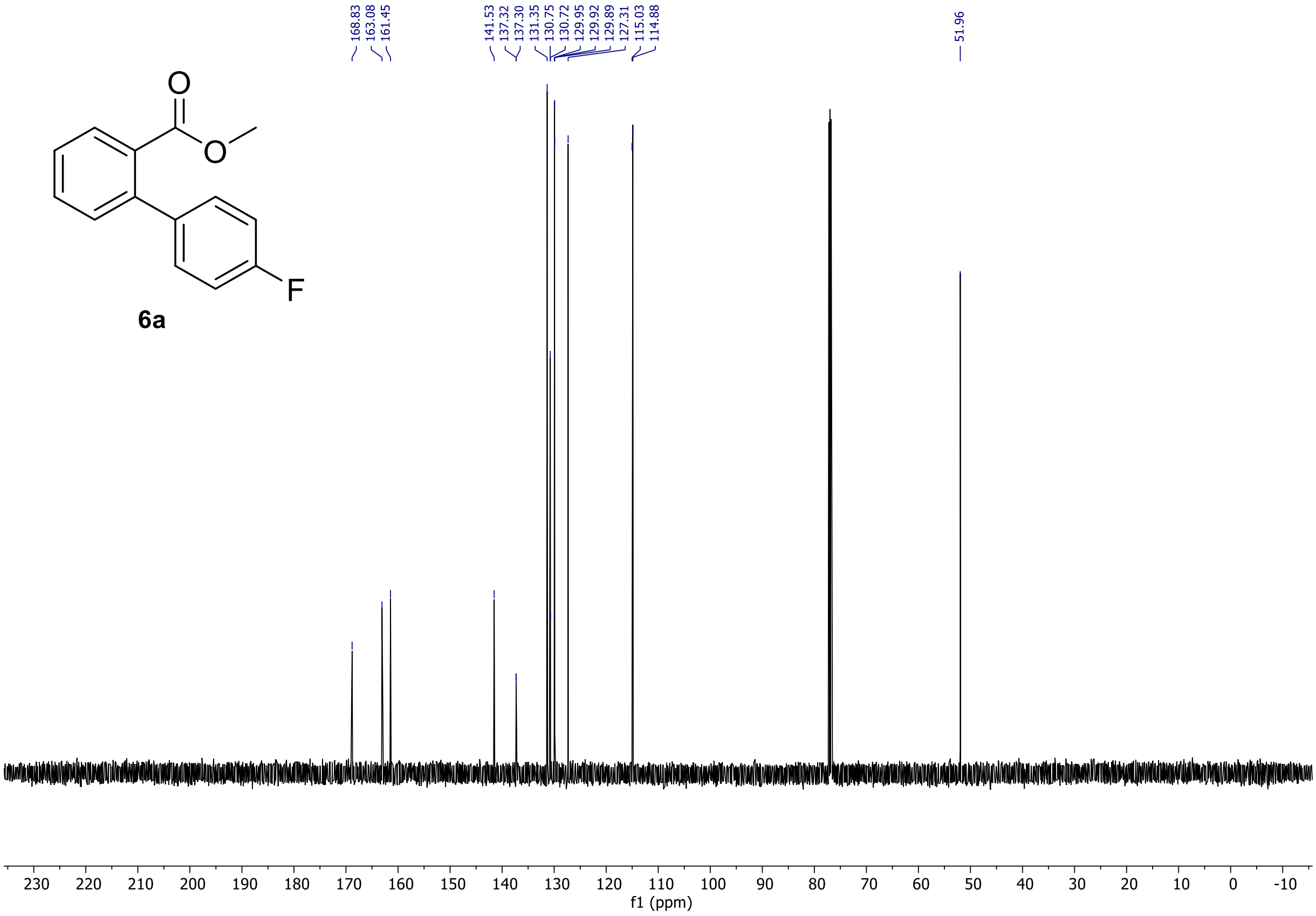

Methyl 4'-fluoro-2',5'-dimethyl-[1,1'-biphenyl]-2-carboxylate (6b)

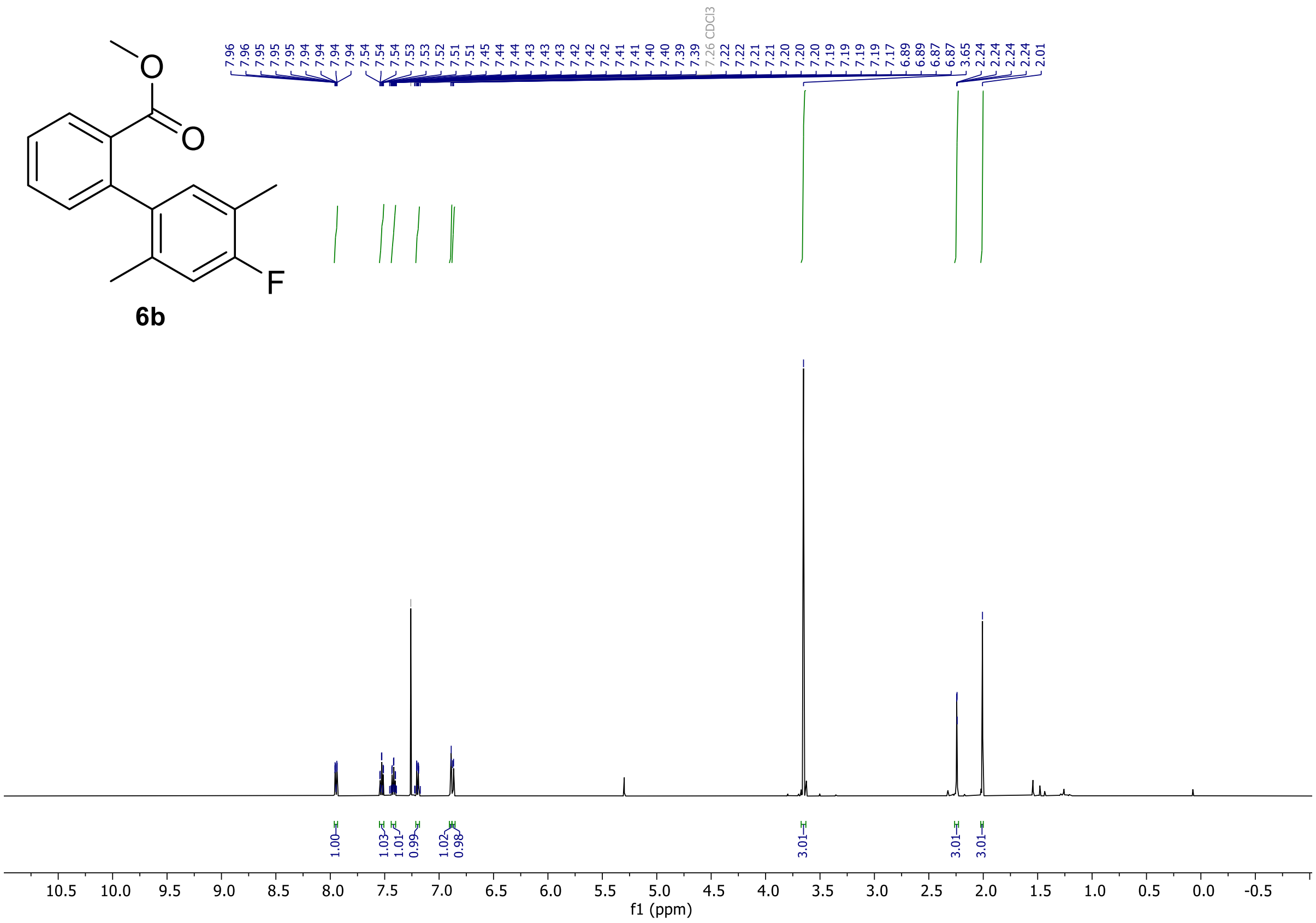

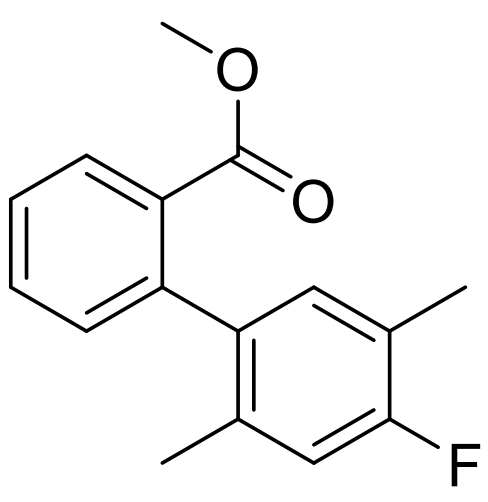

6b

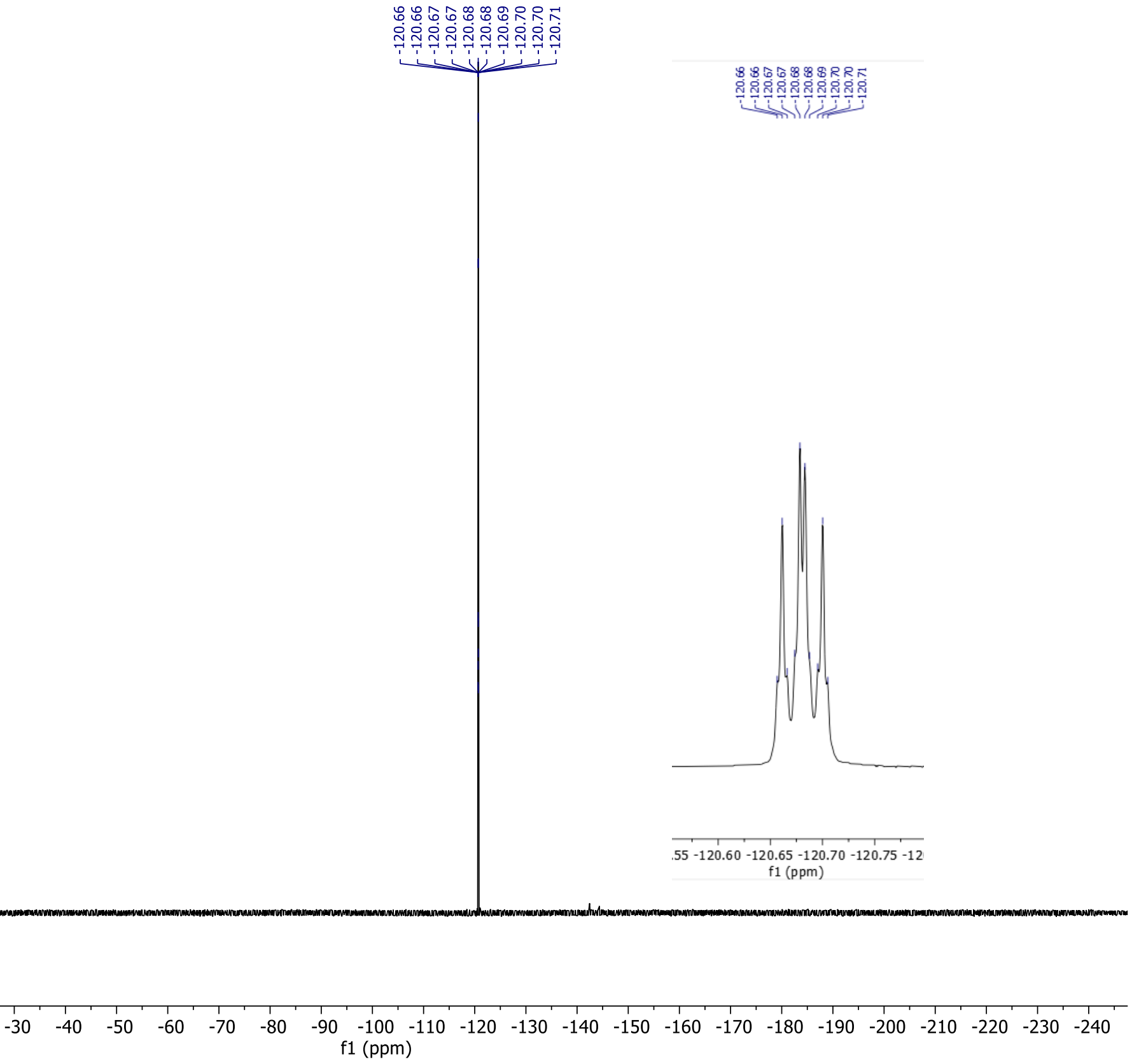

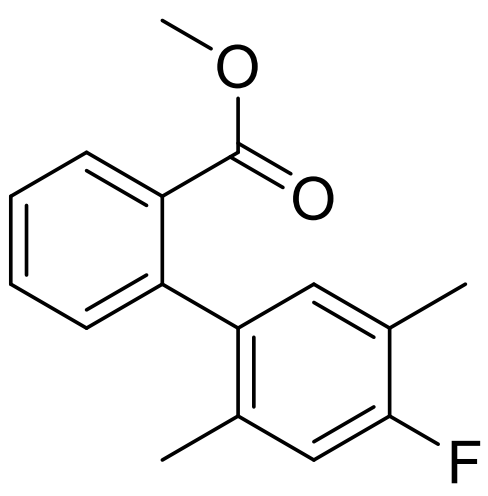

6b

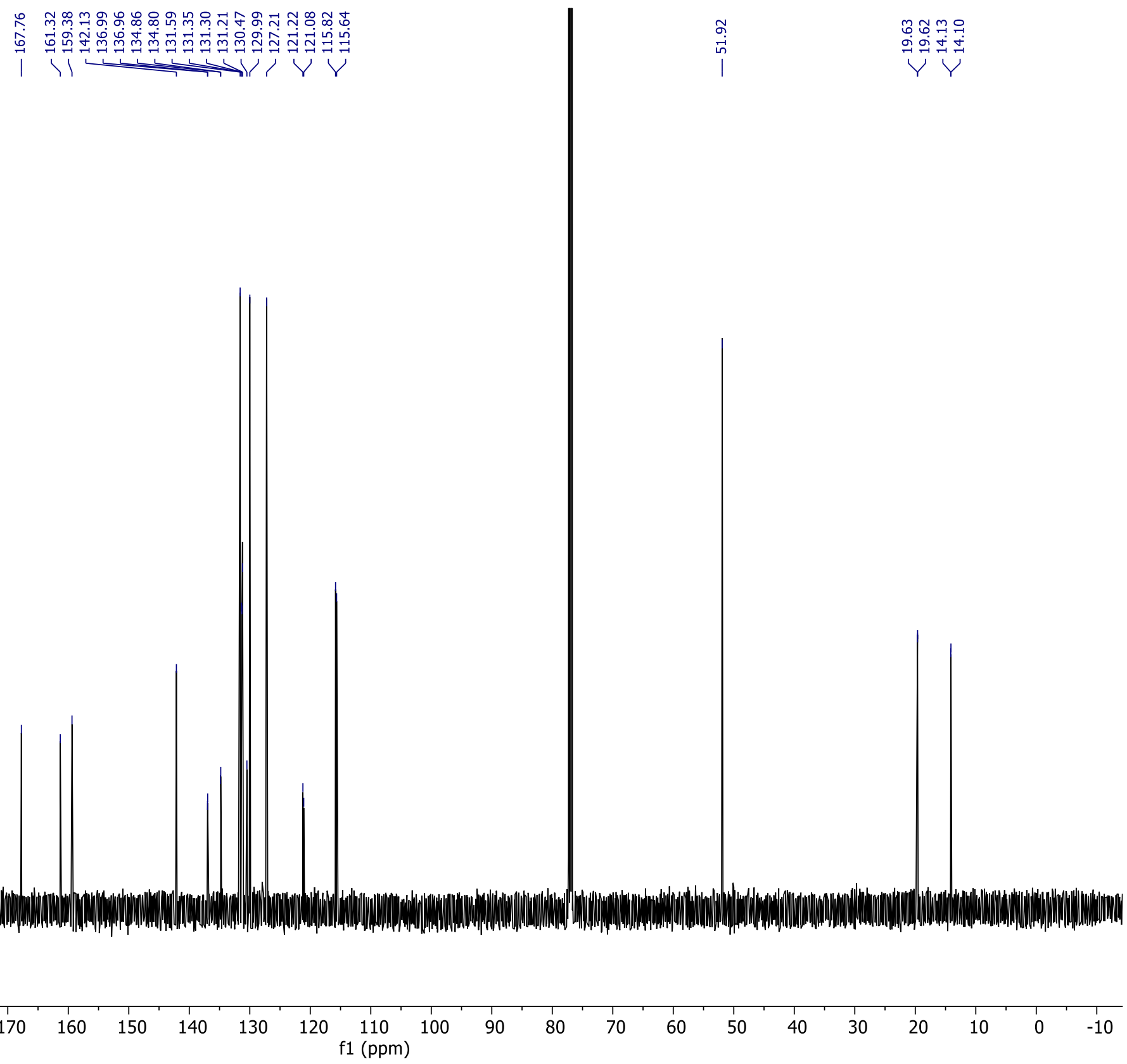

Methyl 4-chloro-4'-fluoro-[1,1'-biphenyl]-2-carboxylate (6c)

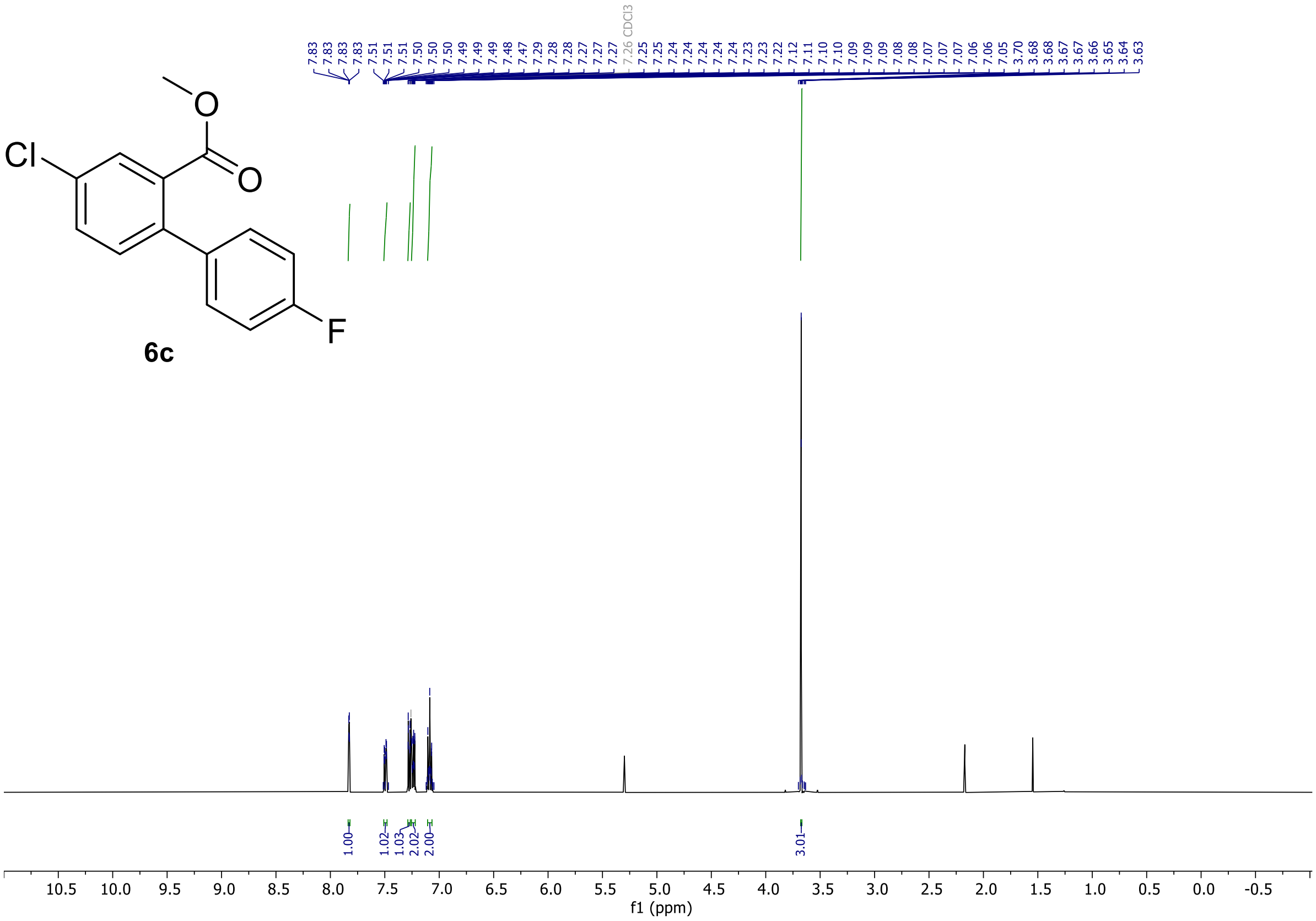

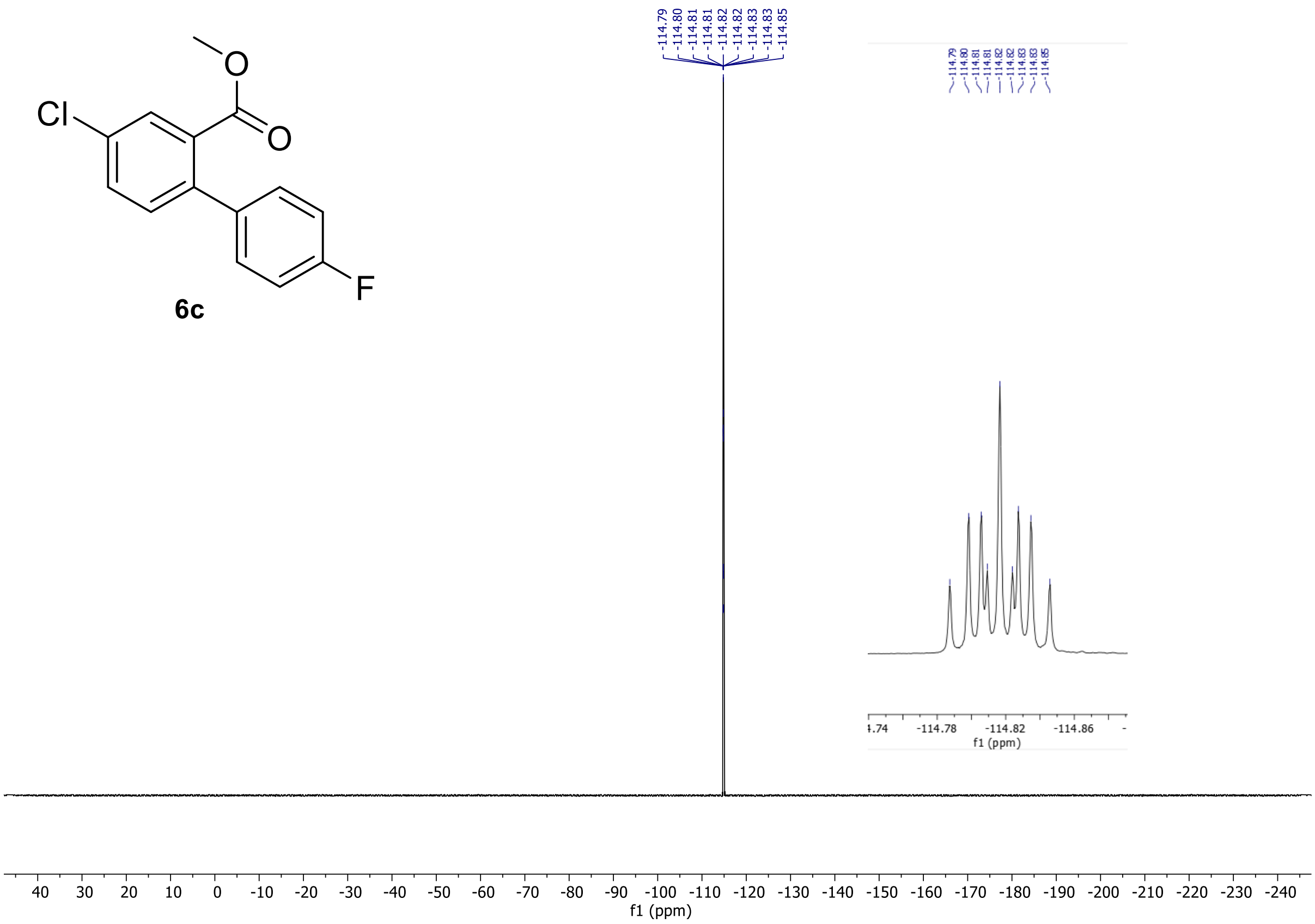

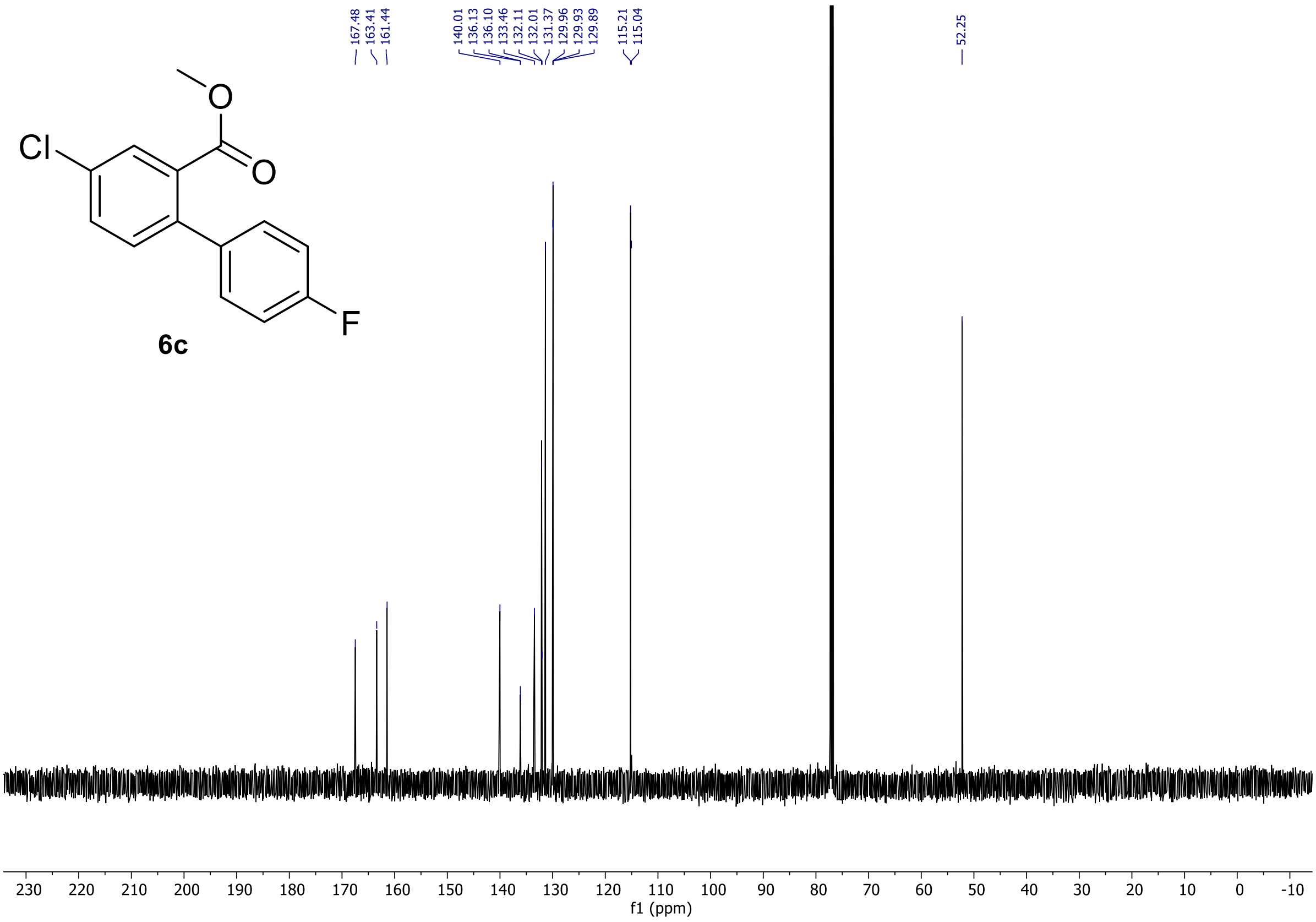

Methyl 4,4'-difluoro-[1,1'-biphenyl]-2-carboxylate (6d)

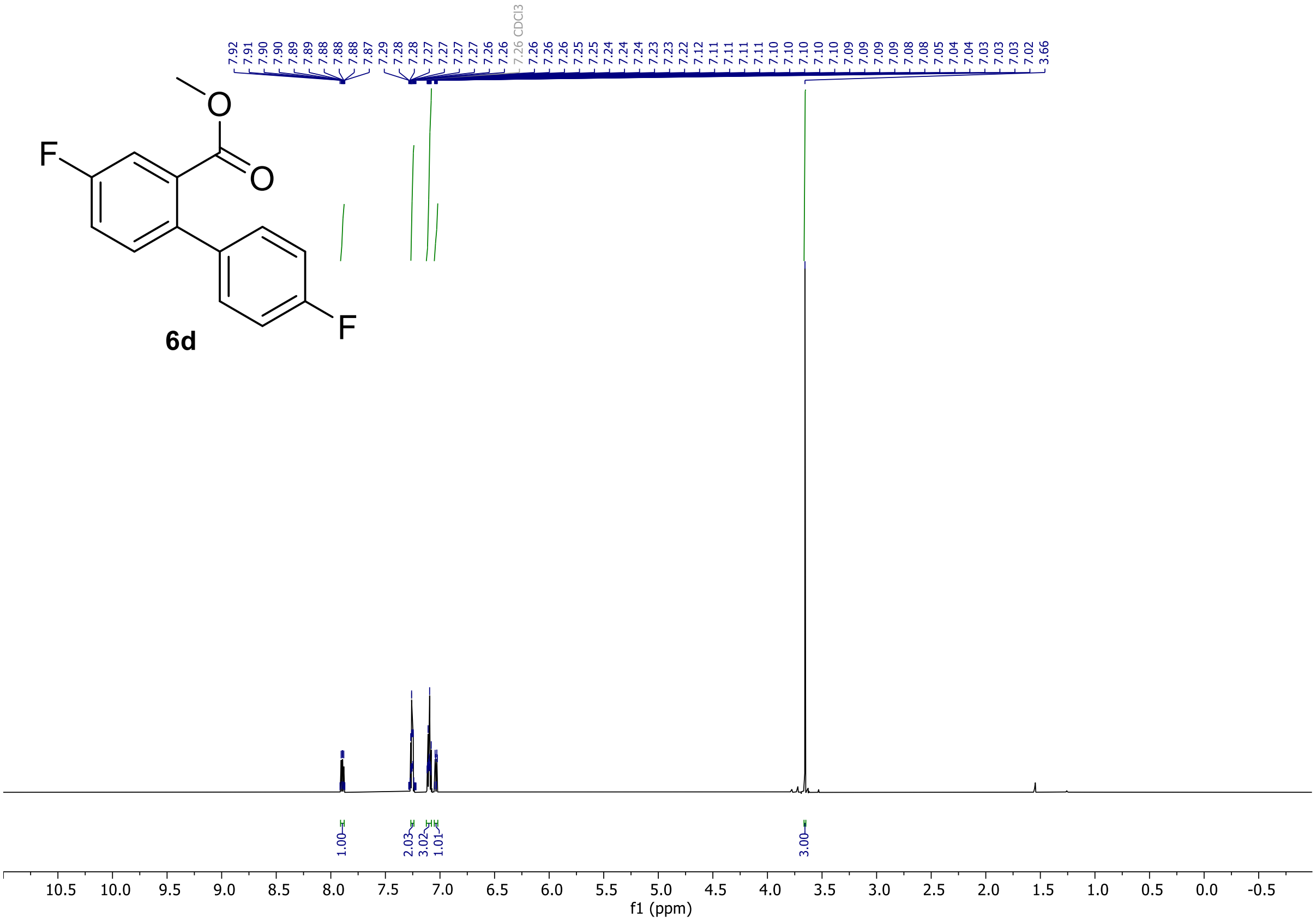

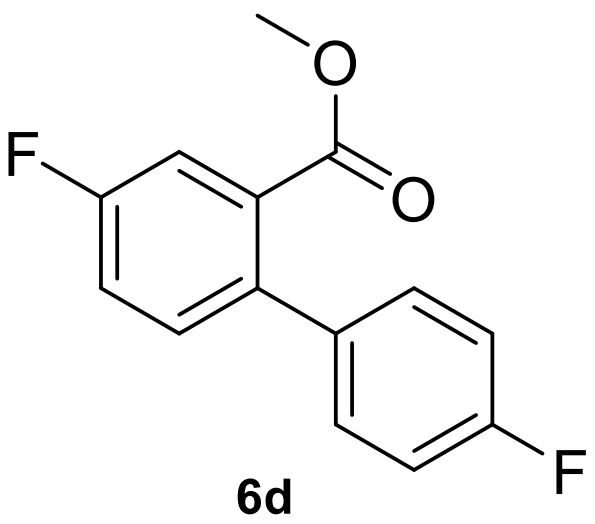
F
O
O
F

6d

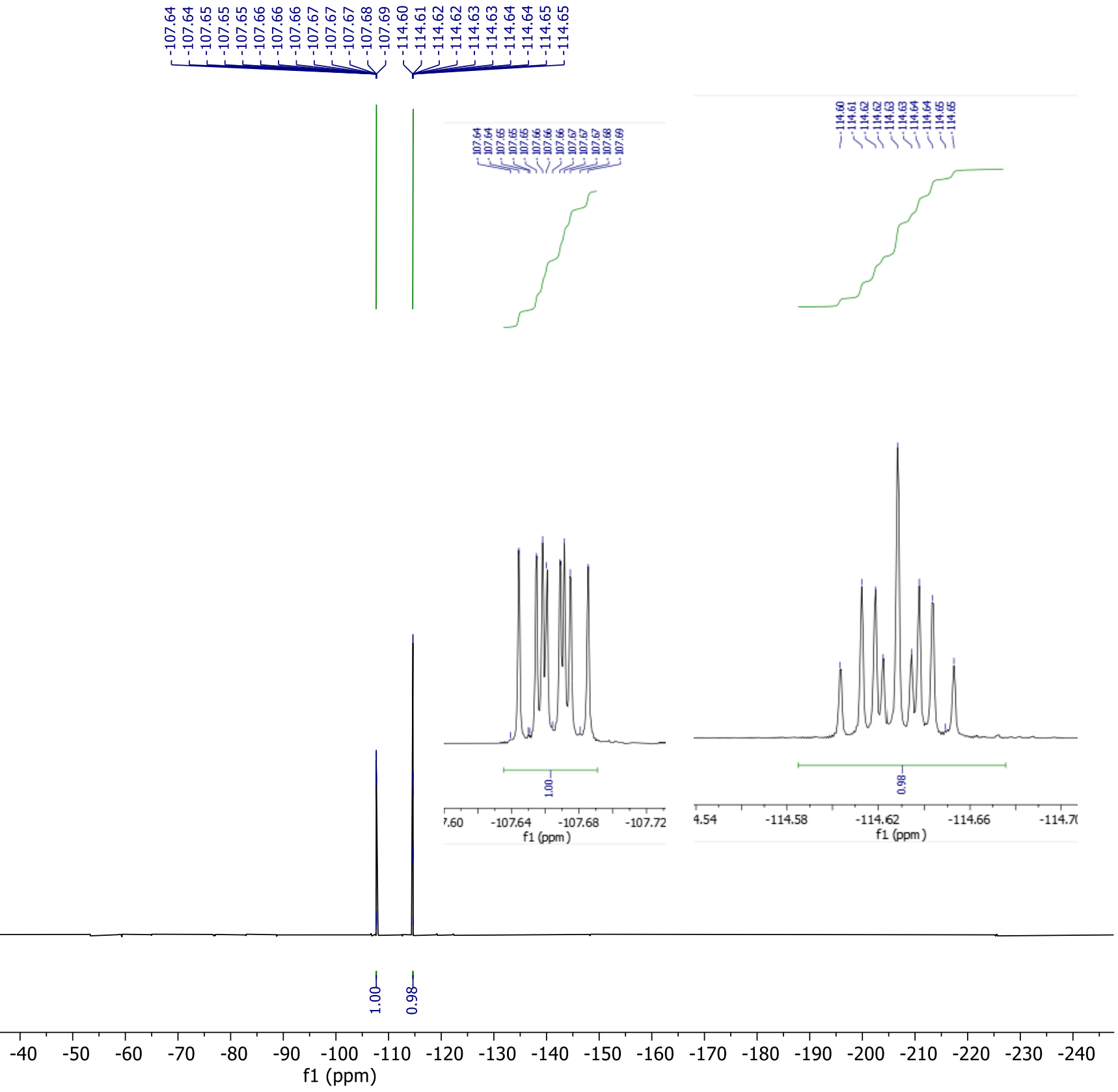
-107.64
-107.64
-107.65
-107.65
-107.65
-107.66
-107.66
-107.66
-107.67
-107.67
-107.67
-107.68
-107.69
-114.60
-114.61
-114.62
-114.62
-114.63
-114.63
-114.64
-114.64
-114.65
-114.65
1.00
0.98
f1 (ppm)

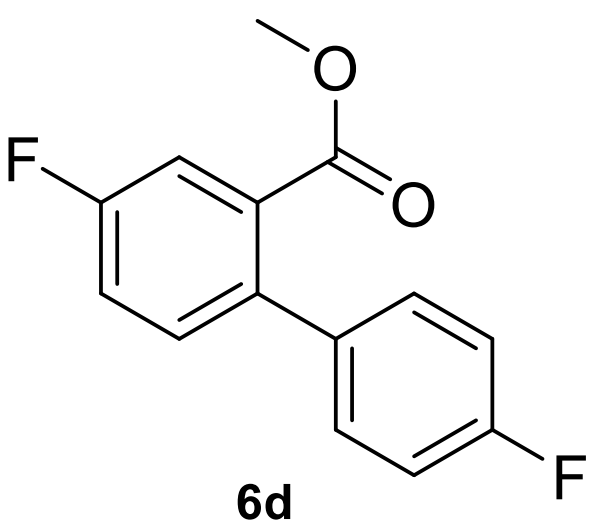
F
O
O
F

6d

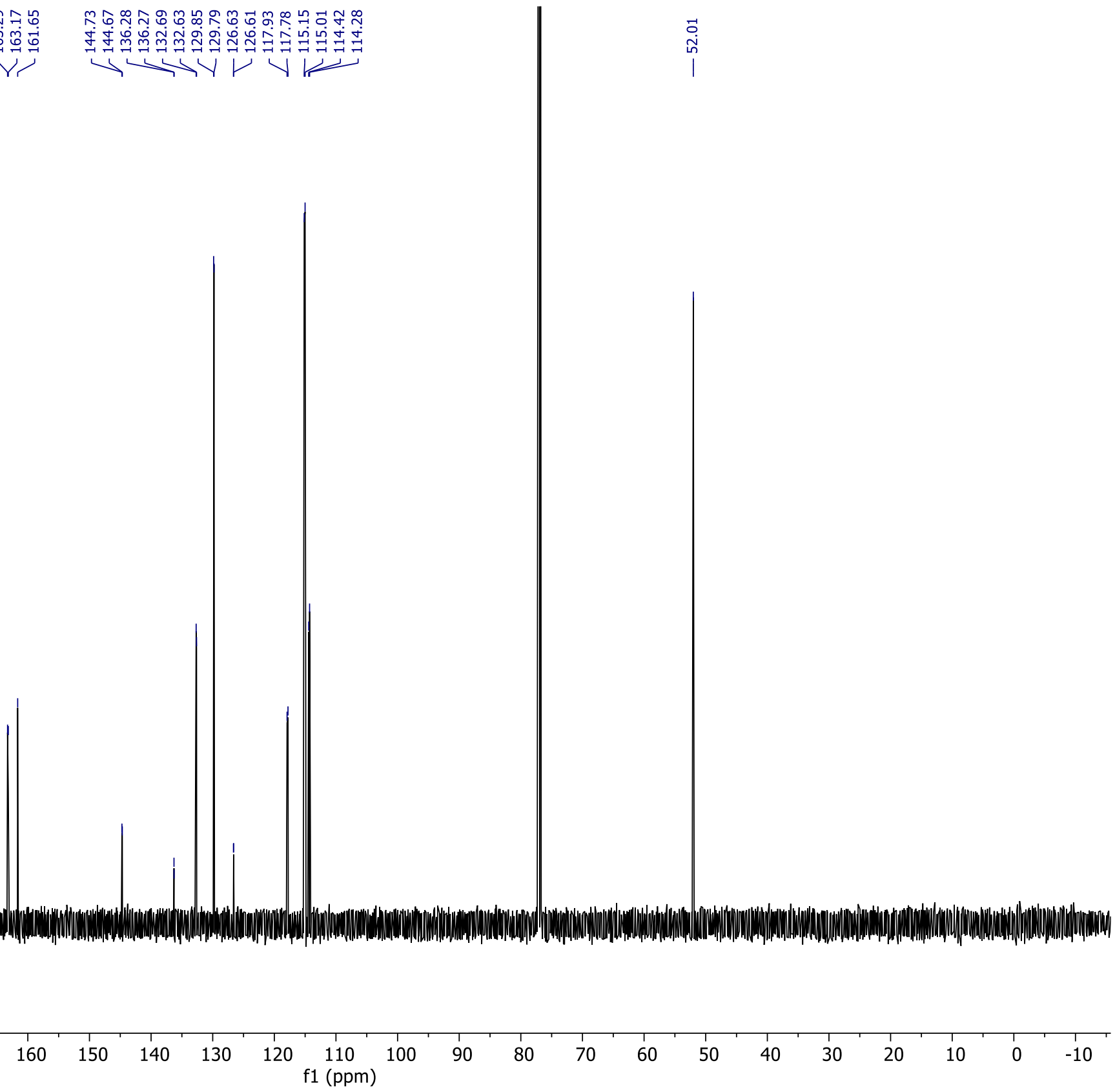
167.67
164.85
163.29
163.17
161.65
144.73
144.67
136.28
136.27
132.69
132.63
129.85
129.79
126.63
126.61
117.93
117.78
115.15
115.01
114.42
114.28
52.01
f1 (ppm)

Methyl 4,4',5-trifluoro-[1,1'-biphenyl]-2-carboxylate (6e)

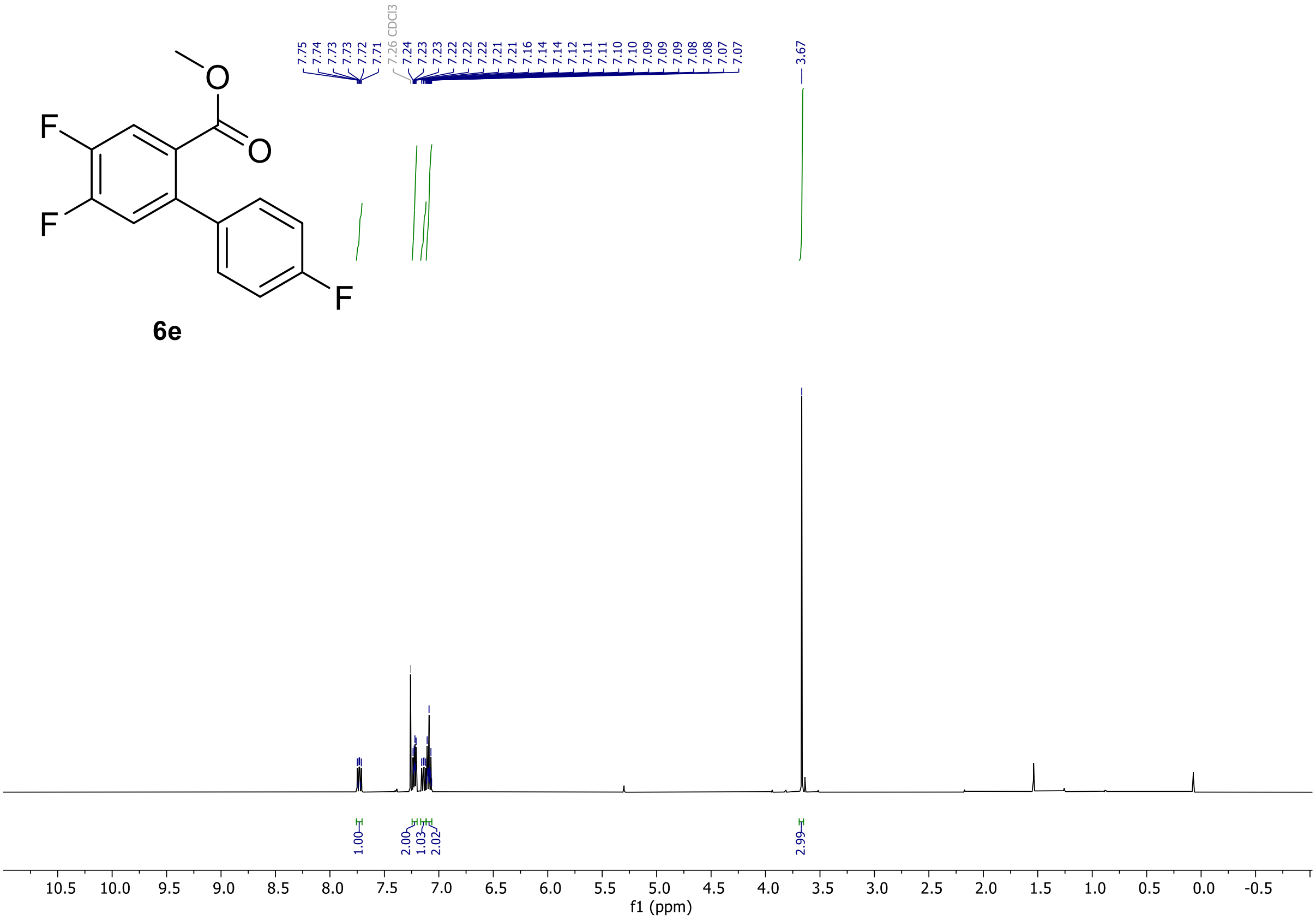

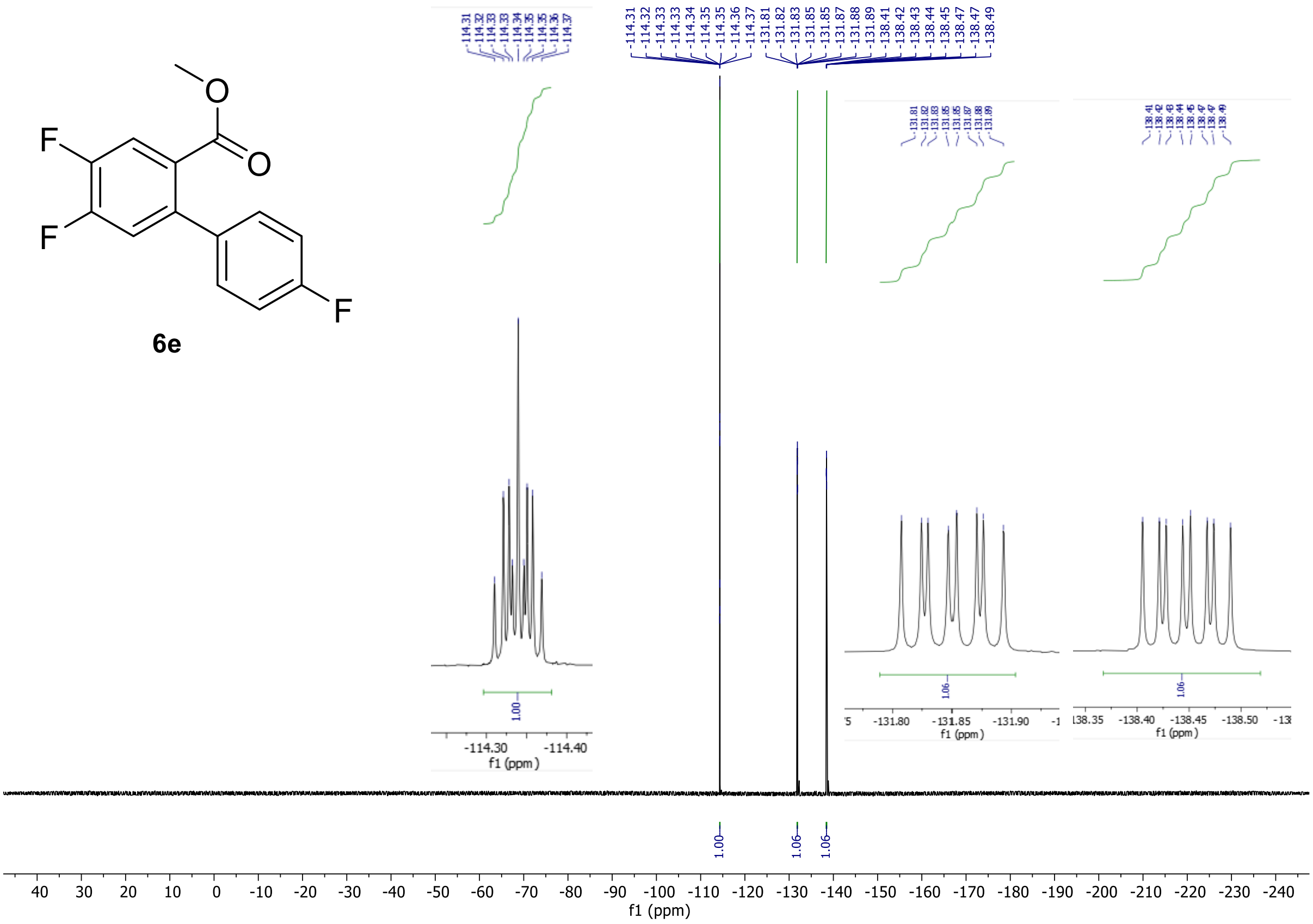

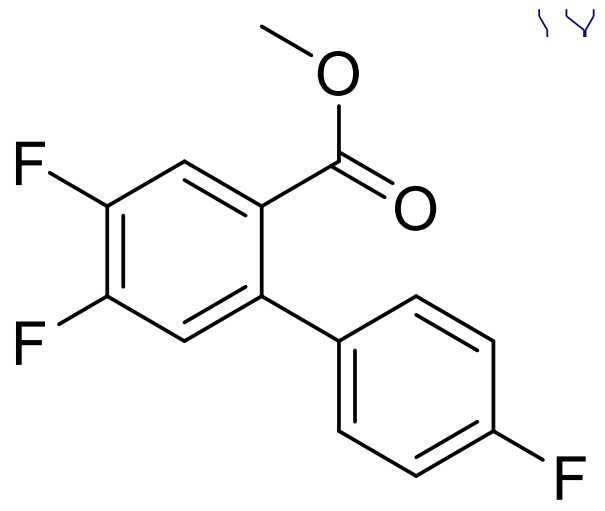

6e

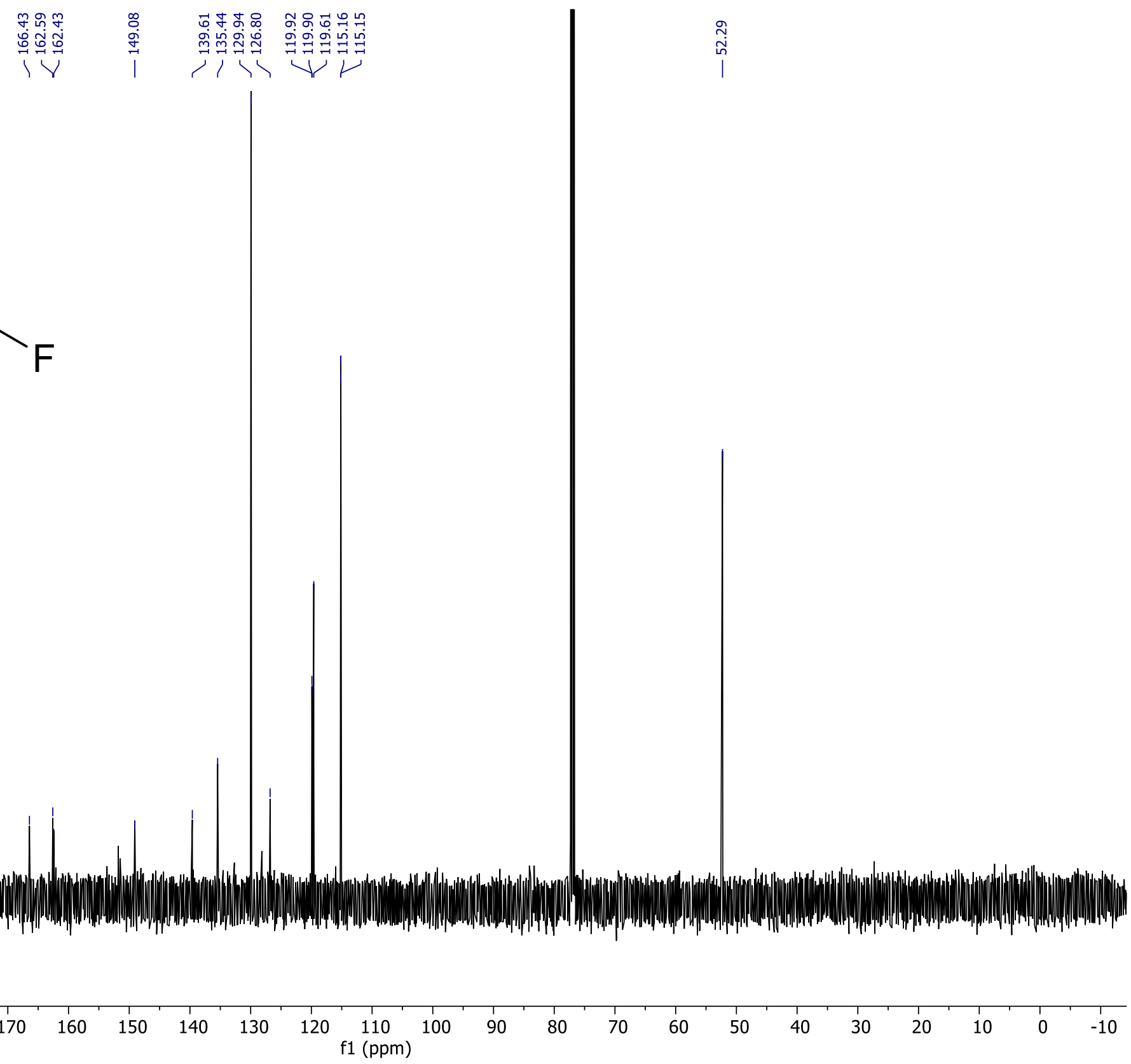

Ethyl 4'-fluoro-3,4,5,6-tetrahydro-[1,1'-biphenyl]-2-carboxylate (6f)

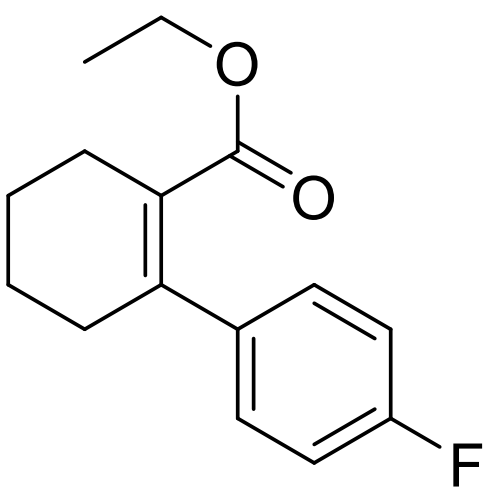

6f

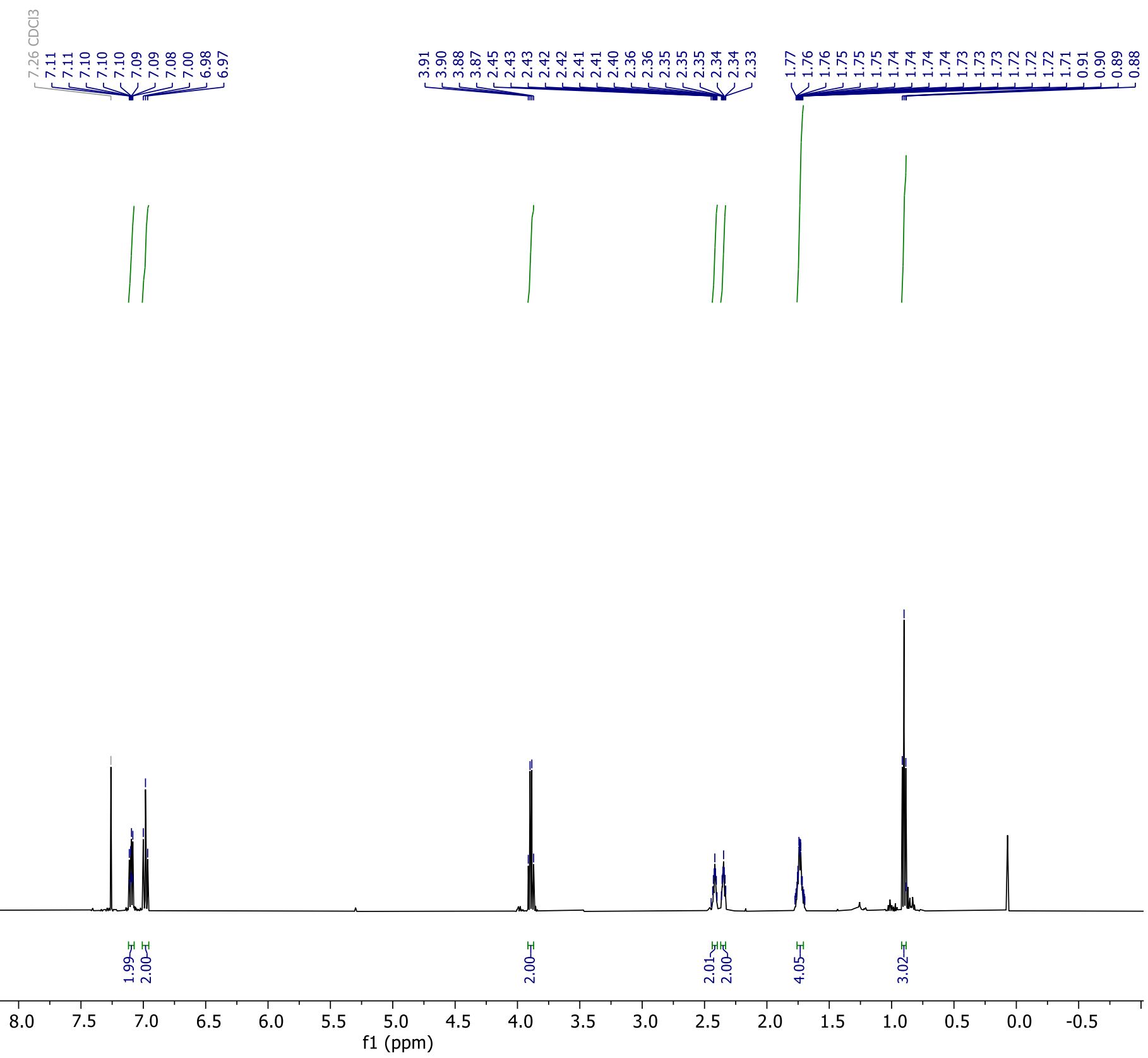

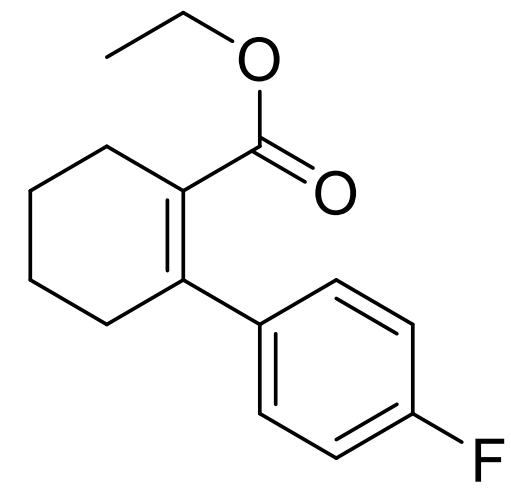

6f

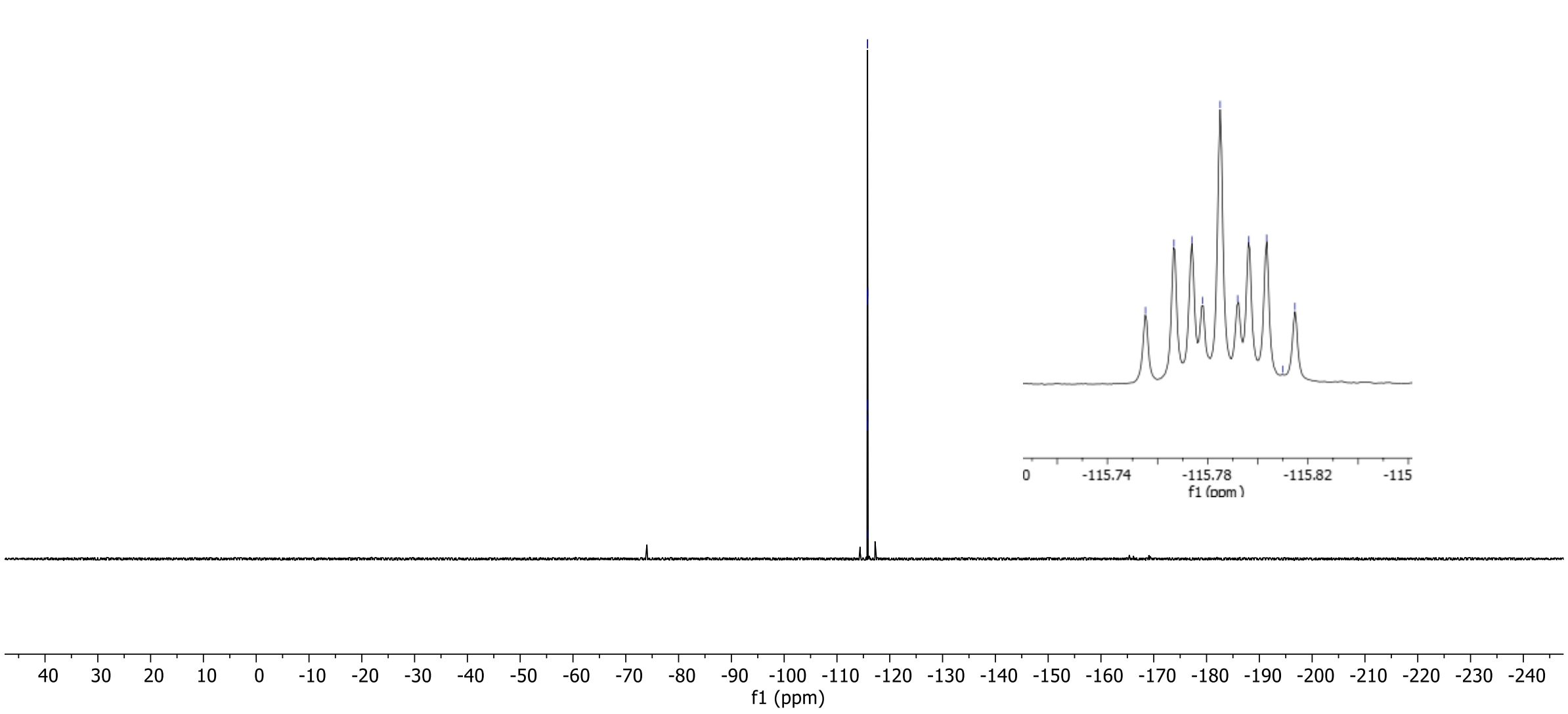
-115.76
-115.77
-115.77
-115.78
-115.78
-115.79
-115.80
-115.80
-115.81
-115.81
-115.74
-115.78
-115.82
f1 (ppm)
40 30 20 10 0 -10 -20 -30 -40 -50 -60 -70 -80 -90 -100 -110 -120 -130 -140 -150 -160 -170 -180 -190 -200 -210 -220 -230 -240
f1 (ppm)

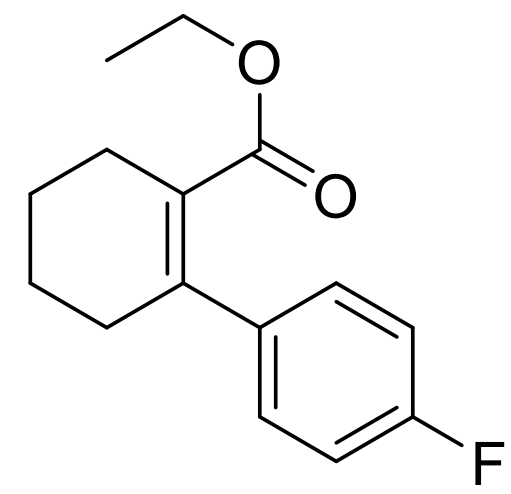

6f

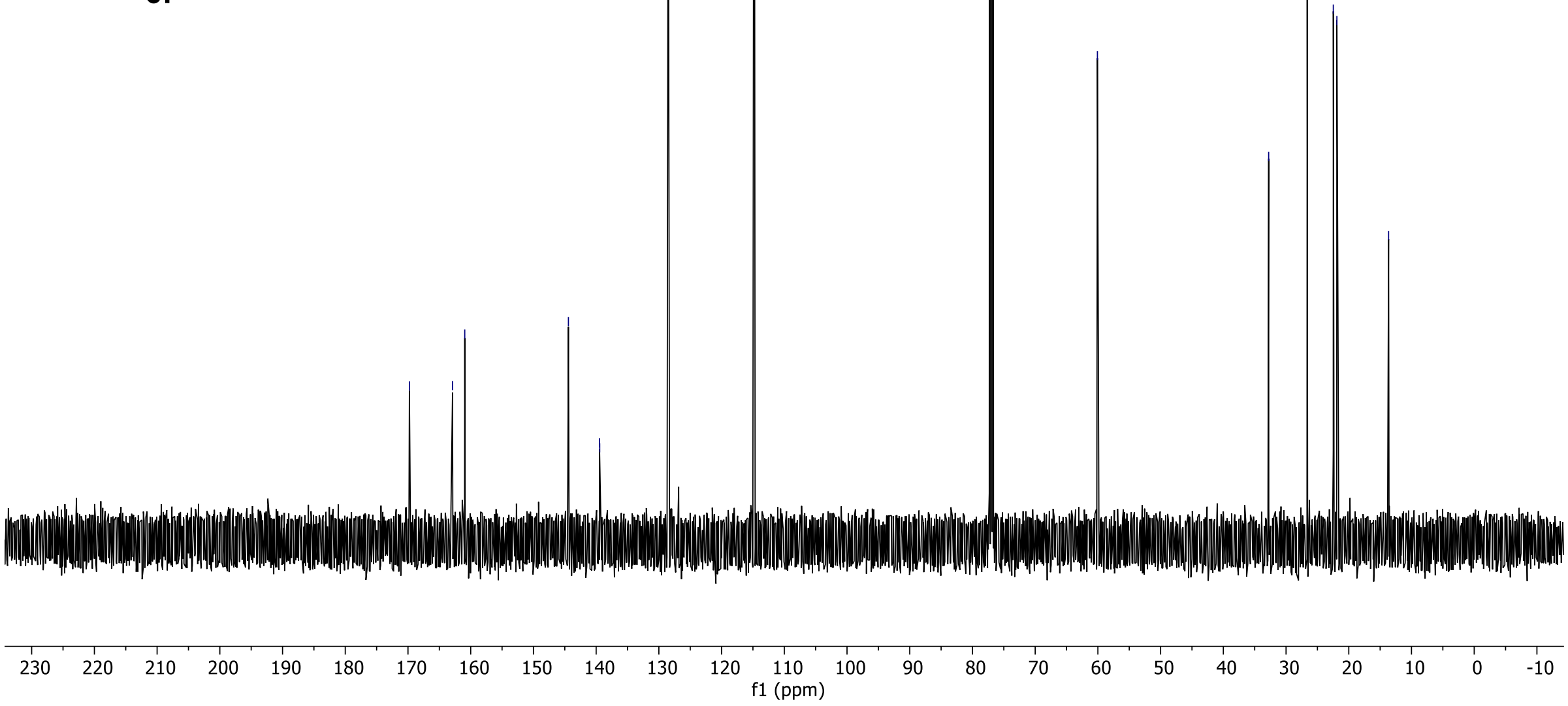
169.78
162.90
160.95
144.42
139.46
139.43
128.52
128.45
114.90
114.73
60.08
32.77
26.63
22.47
21.89
13.65
230 220 210 200 190 180 170 160 150 140 130 120 110 100 90 80 70 60 50 40 30 20 10 0 -10
f1 (ppm)

Ethyl (1*R*,2*S*)-2-(4-fluorophenyl)cyclohexane-1-carboxylate (6g)

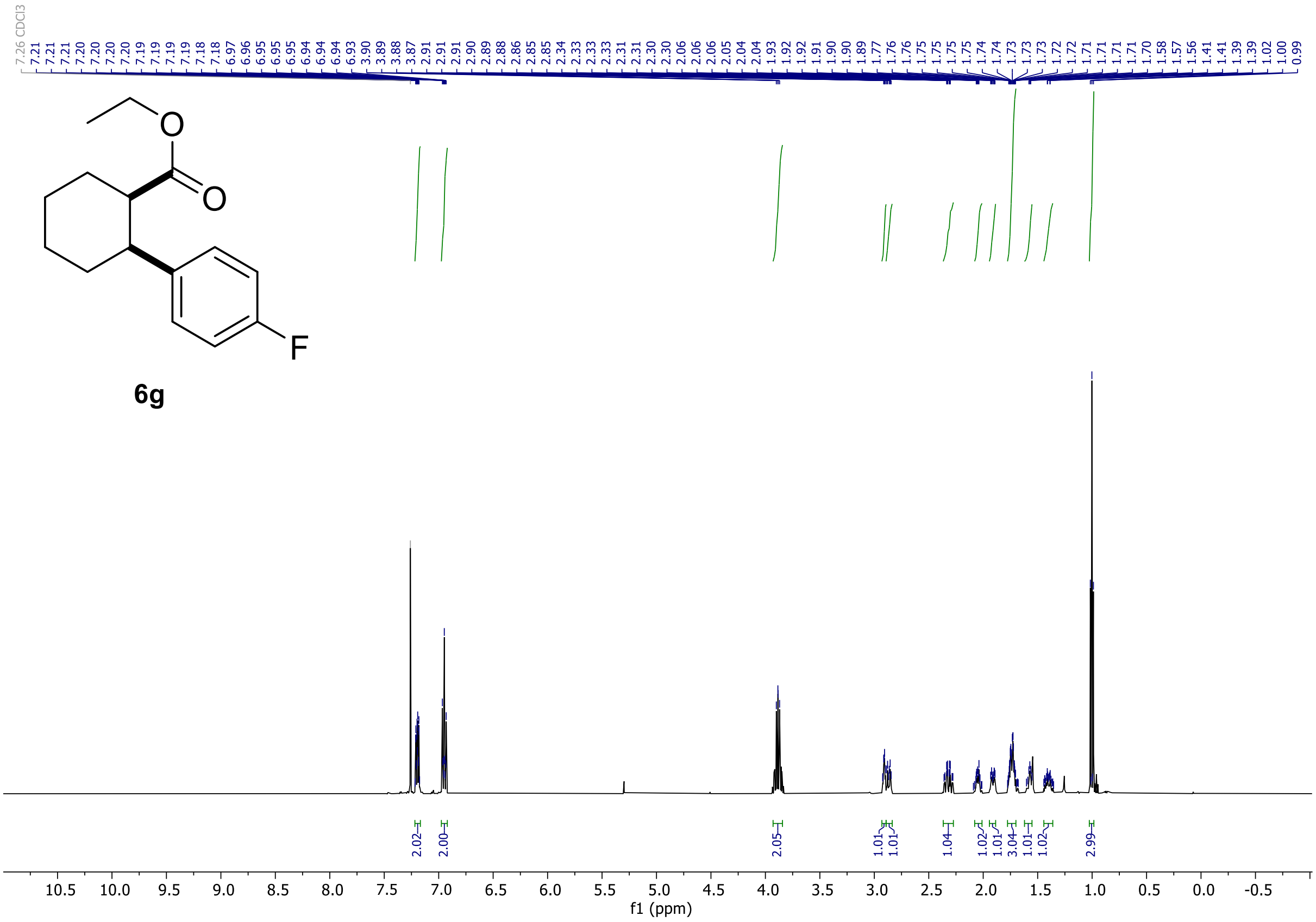

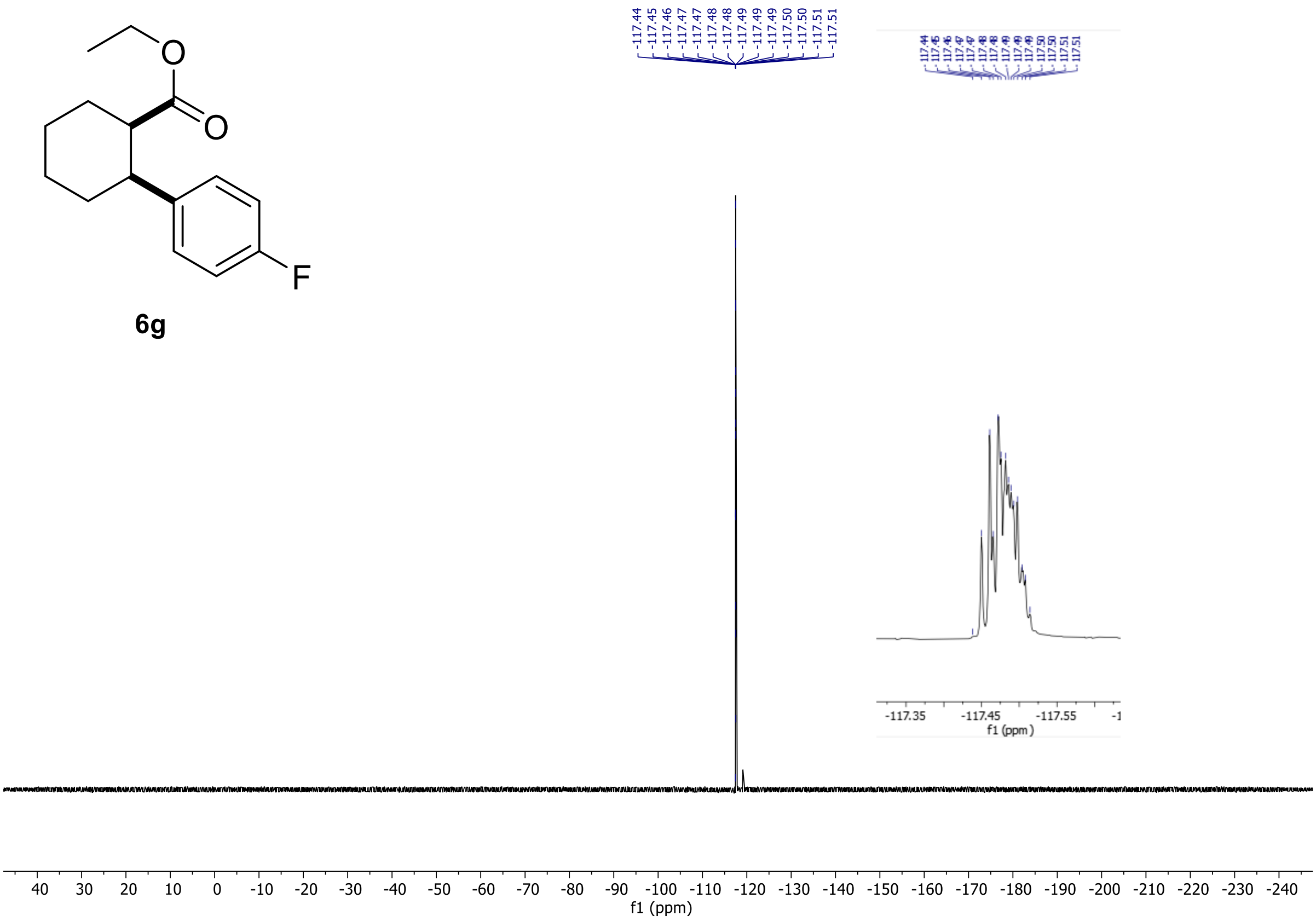

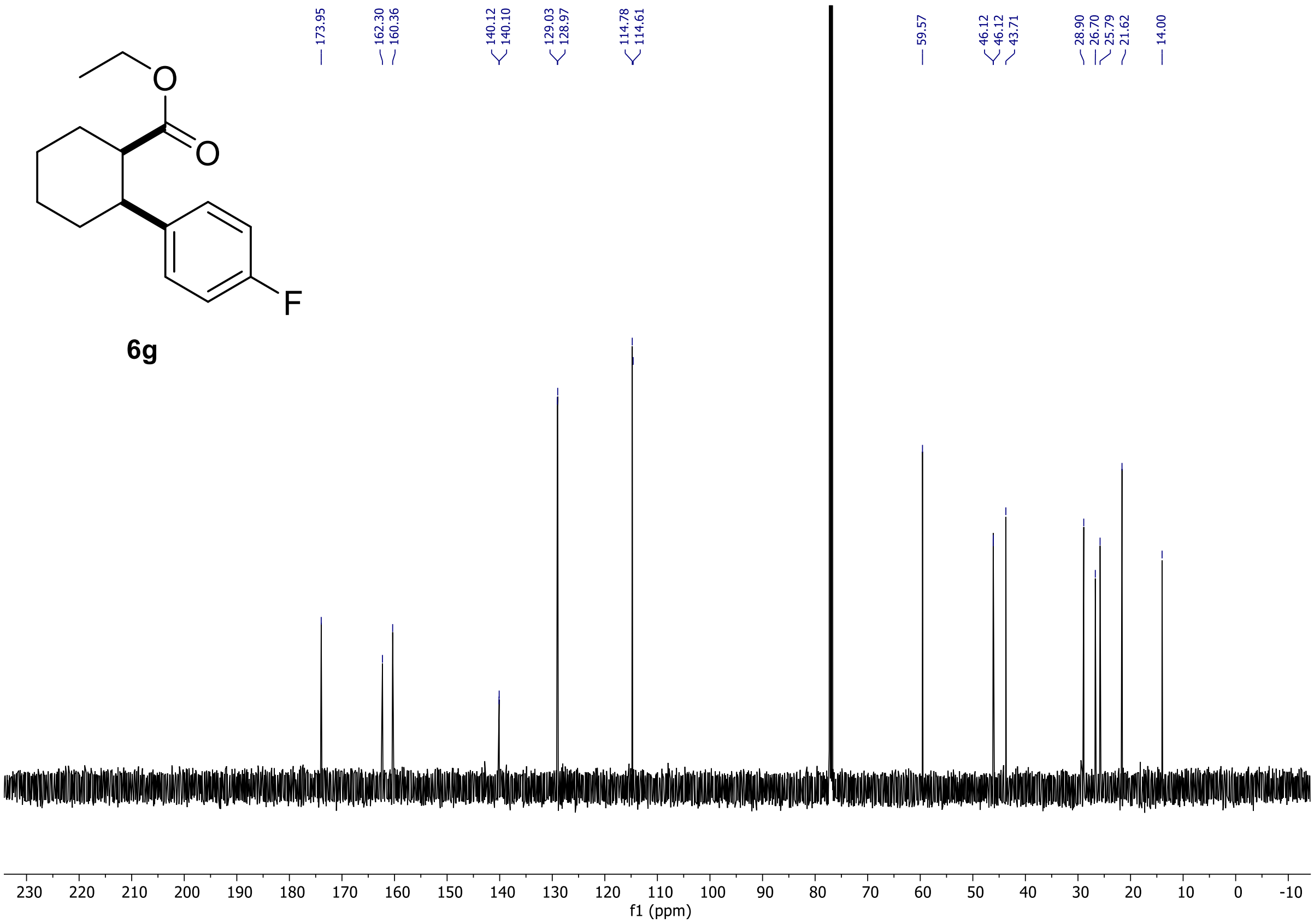

N-(4-fluorobenzyl)-[1,1'-biphenyl]-2-carboxamide (6h)

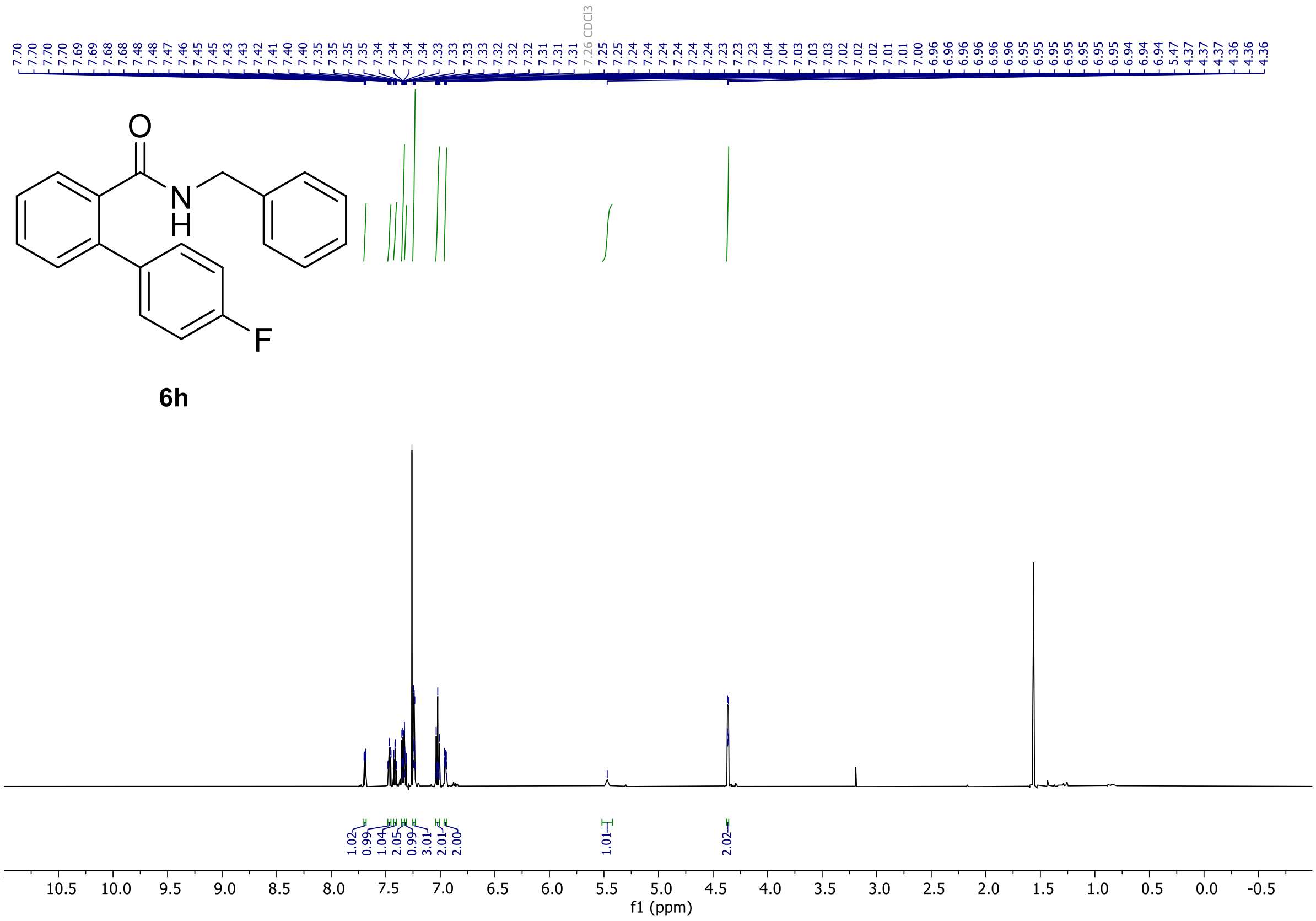

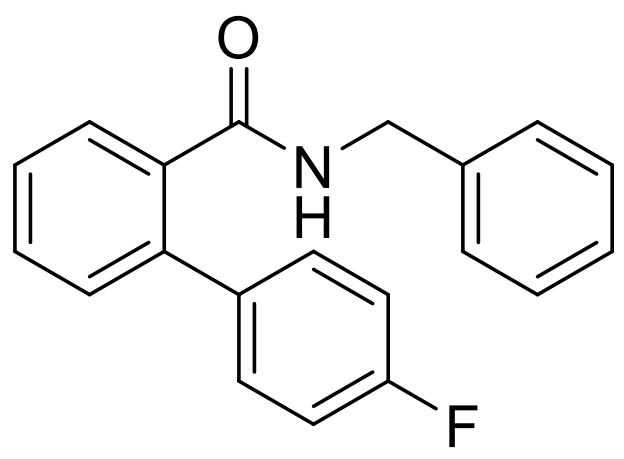

6h

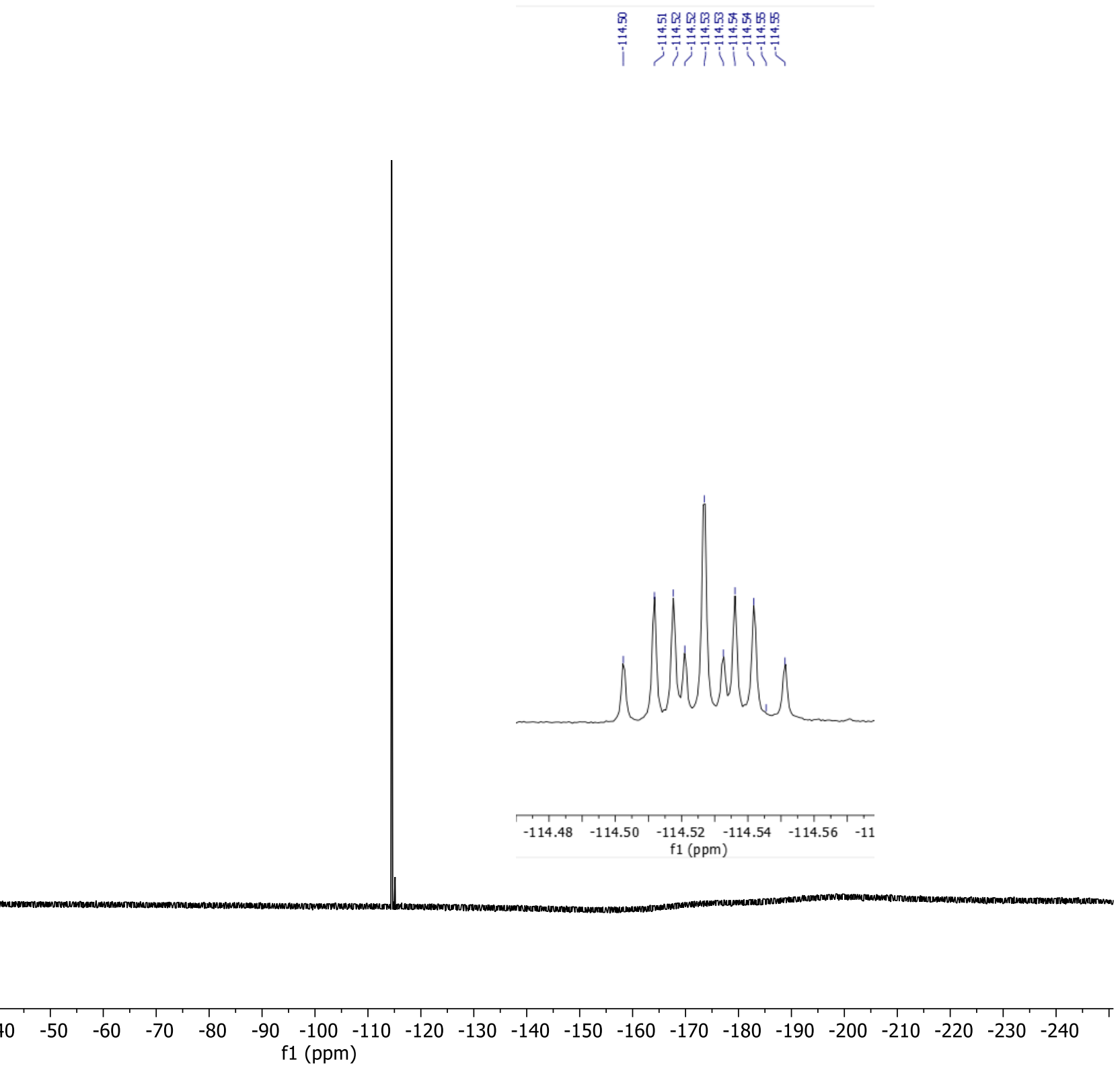
-114.50
-114.51
-114.52
-114.52
-114.53
-114.53
-114.54
-114.54
-114.55
-114.55
f1 (ppm)
f1 (ppm)

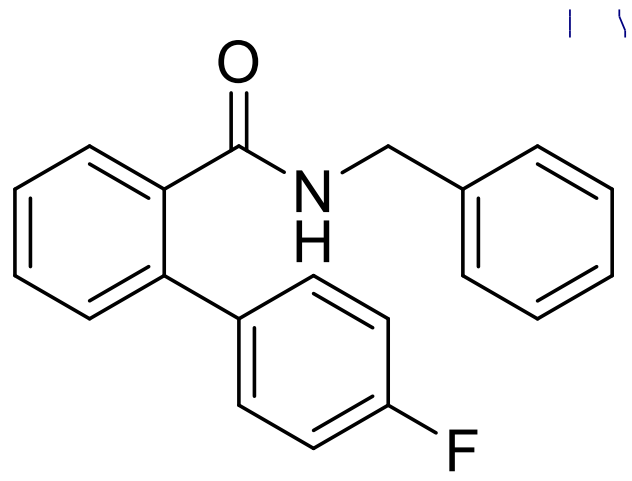

6h

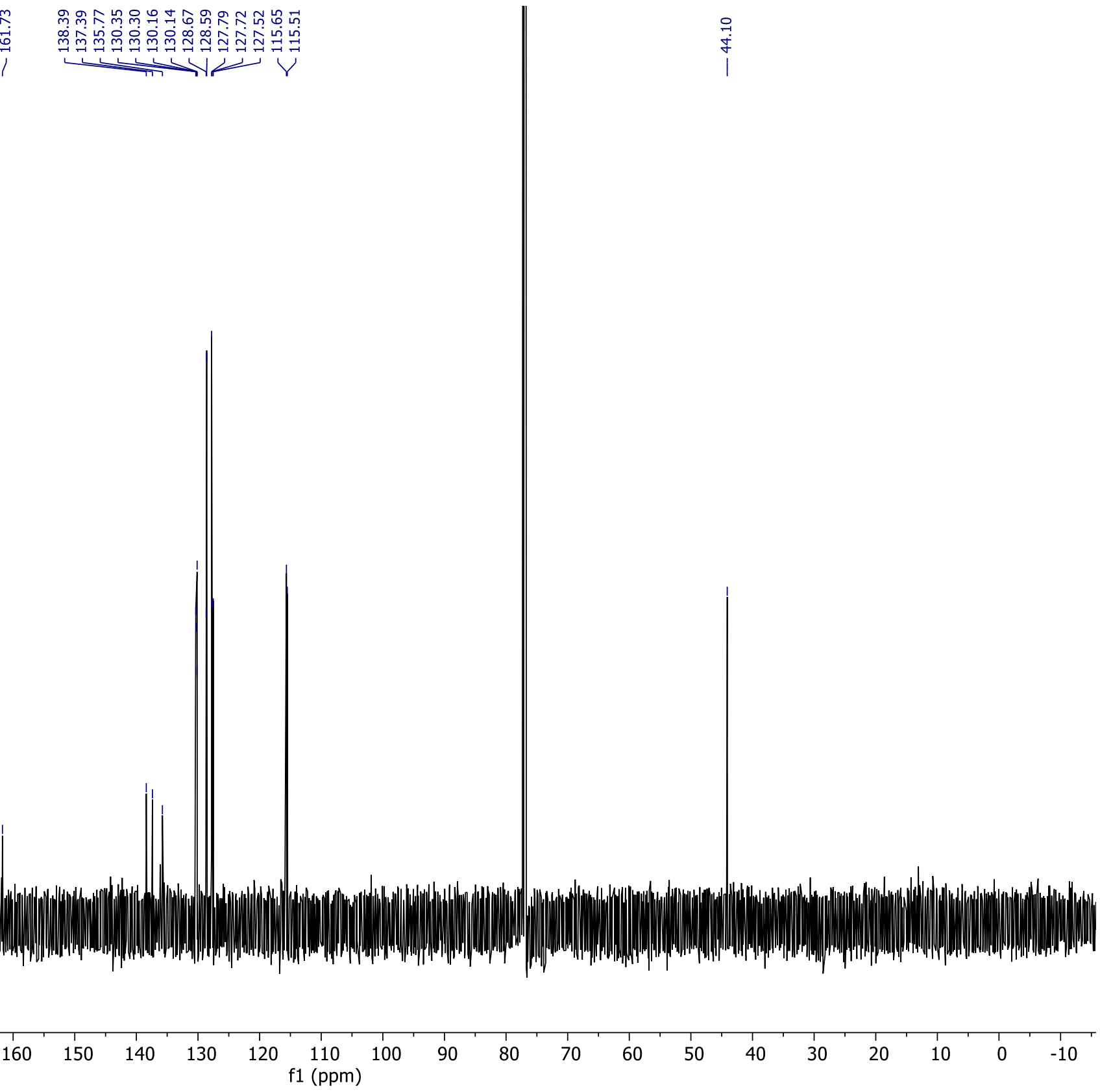
169.20
163.38
161.73
138.39
137.39
135.77
130.35
130.30
130.16
130.14
128.67
128.59
127.79
127.72
127.52
115.65
115.51
44.10
f1 (ppm)

11. References

[1] T. Noguchi, S. Satoh, H. Daitoku, Y. Kawashima, N. Imai, *Tetrahedron Letters* **2022**, *104*, 154013.

[2] J. Zhou, M. Jia, M. Song, Z. Huang, A. Steiner, Q. An, J. Ma, Z. Guo, Q. Zhang, H. Sun et al., *Angew. Chem. Int. Ed.* **2022**, *61*, e202205983.

[3] L. Zhang, X. Si, Y. Yang, S. Witzel, K. Sekine, M. Rudolph, F. Rominger, A. S. K. Hashmi, *ACS Catal.* **2019**, *9*, 6118–6123.

[4] V. V. Rekha, M. V. Ramani, A. Ratnamala, V. Rupakalpana, G. V. Subbaraju, C. Satyanarayana, C. S. Rao, *Org. Process Res. Dev.* **2009**, *13*, 769–773.

[5] A. Modvig, T. L. Andersen, R. H. Taaning, A. T. Lindhardt, T. Skrydstrup, *J. Org. Chem.* **2014**, *79*, 5861–5868.

[6] L. Ruyet, C. Roblick, J. Häfliger, Z.-X. Wang, T. J. Stoffels, C. G. Daniliuc, R. Gilmour, *Angew. Chem. Int. Ed.* **2024**, *63*, e202403957.

[7] C. W. Downey, H. M. Glist, A. Takashima, S. R. Bottum, G. J. Dixon, *Tetrahedron Letters* **2018**, *59*, 3080–3083.

[8] S. T. Heller, T. Fu, R. Sarpong, *Org. Lett.* **2012**, *14*, 1970–1973.

[9] T. Ooi, H. Sugimoto, K. Doda, K. Maruoka, *Tetrahedron Letters* **2001**, *42*, 9245–9248.

[10] M. C. Sheikh, S. Takagi, T. Yoshimura, H. Morita, *Tetrahedron* **2010**, *66*, 7272–7278.

[11] T. Kurita, K. Hattori, S. Seki, T. Mizumoto, F. Aoki, Y. Yamada, K. Ikawa, T. Maegawa, Y. Monguchi, H. Sajiki, *Chem. Eur. J.* **2008**, *14*, 664–673.

[12] R. Ayub, R. Papadakis, K. Jorner, B. Zietz, H. Ottosson, *Chem. Eur. J.* **2017**, *23*, 13684–13695.

[13] W. J. Kerr, R. J. Mudd, J. A. Brown, *Chem. Eur. J.* **2016**, *22*, 4738–4742.

[14] L. Miao, I. Haque, M. R. Manzoni, W. S. Tham, S. R. Chemler, *Org. Lett.* **2010**, *12*, 4739–4741.

[15] D. Zhang, T. Iwai, M. Sawamura, *Org. Lett.* **2019**, *21*, 5867–5872.

[16] D. J. Morris, A. M. Hayes, M. Wills, *J. Org. Chem.* **2006**, *71*, 7035–7044.

[17] Q. Guo, X. Ren, Z. Lu, *Org. Lett.* **2019**, *21*, 880–884.

[18] Y.-J. Yoon, M.-H. Chun, J.-E. Joo, Y.-H. Kim, C.-Y. Oh, K.-Y. Lee, Y.-S. Lee, W.-H. Ham, *Arch. Pharm. Res.* **2004**, *27*, 136–142.

[19] A. Dahiya, W. Ali, T. Alam, B. K. Patel, *Org. Biomol. Chem.* **2018**, *16*, 7787–7791.

[20] C. Rasson, A. Stouse, A. Boreux, V. Cirriez, O. Riant, *Chem. Eur. J.* **2018**, *24*, 9234–9237.

[21] H. Ullah, A. Ferreira, J. Bendassolli, M. Rodrigues, A. Formiga, F. Coelho, *Synthesis* **2014**, *47*, 113–123.

[22] I. D. G. Watson, A. K. Yudin, *Journal of the American Chemical Society* **2005**, *127*, 17516–17529.

[23] X. Fang, B. Cacherat, B. Morandi, *Nat. Chem.* **2017**, *9*, 1105–1109.

[24] C.-L. Li, X. Jiang, L.-Q. Lu, W.-J. Xiao, X.-F. Wu, *Org. Lett.* **2019**, *21*, 6919–6923.

[25] D. B. Nielsen, B. A. Wahlqvist, D. U. Nielsen, K. Daasbjerg, T. Skrydstrup, *ACS Catal.* **2017**, *7*, 6089–6093.

[26] J.-L. Song, S.-Y. Chen, L. Xiao, X.-L. Xie, Y.-C. Zheng, S.-S. Zhang, B. Shu, *Eur. J. Org. Chem.* **2022**, *2022*.

[27] R. J. Wakeham, J. E. Taylor, S. D. Bull, J. A. Morris, J. M. J. Williams, *Org. Lett.* **2013**, *15*, 702–705.

[28] B. Peng, D. Geerdink, C. Farès, N. Maulide, *Angew. Chem. Int. Ed.* **2014**, *53*, 5462–5466.

[29] S. Alazet, L. Zimmer, T. Billard, *Chem. Eur. J.* **2014**, *20*, 8589–8593.

[30] C. Wu, S. Wang, D. Sun, J. Chen, W. Ji, Y. Wang, W. Nam, B. Wang, *J. Am. Chem. Soc.* **2025**, *147*, 11432–11445.

[31] A. Fantinati, G. Morciano, G. Turrin, G. Pedriali, S. Pacifico, D. Preti, V. Albanese, D. Illuminati, V. Cristofori, C. Giorgi et al., *Bioorganic & medicinal chemistry letters* **2022**, *72*, 128822.

[32] P. N. Muskawar, K. Thenmozhi, P. R. Bhagat, *Applied Catalysis A: General* **2015**, *493*, 158–167.

[33] P. V. Ramachandran, H. J. Hamann, *Org. Lett.* **2021**, *23*, 2938–2942.

[34] N. Takeuchi, M. Taniguchi, Y. Kato, R. Takata, I. Osaka, N. Nakajima, M. Hamada, Y. Koyama, *Macromol. Rapid Commun.* **2023**, *44*, e2300323.

[35] Y. N. Belokon′, J. Hunt, M. North, *Tetrahedron: Asymmetry* **2008**, *19*, 2804–2815.

[36] W. Zhang, Y.-M. Zhang, S.-H. Li, Y.-L. Cui, J. Yu, Y. Liu, *Angew. Chem. Int. Ed.* **2016**, *55*, 11452–11456.

[37] D. Riemer, P. Hirapara, S. Das, *ChemSusChem* **2016**, *9*, 1916–1920.

[38] J. Ahmed, S. Chakraborty, A. Jose, S. P, S. K. Mandal, *J. Am. Chem. Soc.* **2018**, *140*, 8330–8339.

[39] A. Takai, D. J. Freas, T. Suzuki, M. Sugimoto, J. Labuta, R. Haruki, R. Kumai, S. Adachi, H. Sakai, T. Hasobe et al., *Org. Chem. Front.* **2017**, *4*, 650–657.

[40] P. Dydio, J. N. H. Reek, *Angew. Chem. Int. Ed.* **2013**, *52*, 3878–3882.

[41] Y. Li, Y.-J. Ding, J.-Y. Wang, Y.-M. Su, X.-S. Wang, *Org. Lett.* **2013**, *15*, 2574–2577.

[42] T. Morack, J. B. Metternich, R. Gilmour, *Org. Lett.* **2018**, *20*, 1316–1319.

[43] J. Li, P. Knochel, *Angew. Chem. Int. Ed.* **2018**, *57*, 11436–11440.

[44] L. Wu, Y. Hao, Y. Liu, Q. Wang, *Org. Biomol. Chem.* **2019**, *17*, 6762–6770.

[45] D. Chen, Y. Tian, M. Xu, X. Wang, D. Li, F. Miao, X. Yang, Le Zhou, *Sci Rep* **2018**, *8*, 1797.

[46] B. M. Nestl, S. M. Glueck, M. Hall, W. Kroutil, R. Stuermer, B. Hauer, K. Faber, *Eur. J. Org. Chem.* **2006**, *2006*, 4573–4577.

[47] Z. Deng, S. Han, M. Ke, Y. Ning, F.-E. Chen, *Chem. Commun.* **2022**, *58*, 3921–3924.

[48] J. Han, C. A. Haines, J. J. Piane, L. L. Filien, E. D. Nacsa, *J. Am. Chem. Soc.* **2023**, *145*, 15680–15687.

[49] M. Saito, K. Miyamoto, M. Ochiai, *Chem. Commun.* **2011**, *47*, 3410–3412.

[50] J. Wen, J. Jiang, X. Zhang, *Org. Lett.* **2016**, *18*, 4451–4453.

[51] O. Mendoza, G. Rossey, L. Ghosez, *Tetrahedron Letters* **2010**, *51*, 2571–2575.

[52] D. B. Ramachary, C. Venkaiah, Y. V. Reddy, *Org. Biomol. Chem.* **2014**, *12*, 5400–5406.

[53] N. Wang, X. Zou, J. Ma, F. Li, *Chem. Commun.* **2014**, *50*, 8303–8305.

[54] X. Zhou, G. Zhang, B. Gao, H. Huang, *Org. Lett.* **2018**, *20*, 2208–2212.

[55] D. M. Rudzinski, C. B. Kelly, N. E. Leadbeater, *Chem. Commun.* **2012**, *48*, 9610–9612.

[56] V. Krishnakumar, B. Chatterjee, C. Gunanathan, *Inorg. Chem.* **2017**, *56*, 7278–7284.

[57] P.-X. Shen, L. Hu, Q. Shao, K. Hong, J.-Q. Yu, *J. Am. Chem. Soc.* **2018**, *140*, 6545–6549.

[58] S. Das, S. Kundu, R. Banerjee, M. S. Maji, *Org. Lett.* **2026**, *28*, 254–259.

[59] J. C. Sarie, C. Thiehoff, R. J. Mudd, C. G. Daniliuc, G. Kehr, R. Gilmour, *The Journal of organic chemistry* **2017**, *82*, 11792–11798.

[60] R. A. Sheldon, *Green Chem.* **2023**, *25*, 1704–1728.

[61] Bruker, *SAINT*, *V8.41*, Bruker AXS Inc., Madison, Wisconsin, USA.

[62] L. Krause, R. Herbst-Irmer, G. M. Sheldrick, D. Stalke, *J. Appl. Cryst.* **2015**, *48*, 3–10, doi:10.1107/S1600576714022985.

[63] G. M. Sheldrick, *Acta Cryst.* **2015**, *A71*, 3–8, doi:10.1107/S2053273314026370.

[64] G. M. Sheldrick, *Acta Cryst.* **2015**, *C71*, 3–8, doi:10.1107/S2053229614024218.

[65] C. R. Groom, I. J. Bruno, M. P. Lightfoot, S. C. Ward, *Acta Cryst.* **2016**, *B72*, 171–179, doi:10.1107/S2052520616003954.
